# Design, Synthesis, and Application of a Type of Chiral Spirocyclic Naphthylamines

**DOI:** 10.1021/jacs.5c12568

**Published:** 2025-09-29

**Authors:** Ronghua Zhang, Minghui Zhu, Jianwei Sun

**Affiliations:** Department of Chemistry and the Hong Kong Branch of Chinese National Engineering Research Centre for Tissue Restoration & Reconstruction, 58207 The Hong Kong University of Science and Technology, Clear Water Bay, Kowloon, Hong Kong SAR 999077, China

## Abstract

A family of new spirocyclic chiral mono- and diamine structures has been designed together with their de novo asymmetric synthesis, featuring a short sequence from simple substrates. This study complements the previous limited exploration of the counterparts of well-established 1,1′-spirobiindane-7,7′-diol (SPINOL) and its modified analogs. The convenient enantioselective synthesis of 2,2′,3,3′-tetrahydro-1,1′-spirobi-[phenalene]-9,9′-diamine (SPHENAM) and 9′-amino-2,2′,3,3′-tetrahydro-1,1′-spirobi­[phenalen]-9-ol (NOSPHEN) represents a notable advantage over the limited spirocyclic precedents of this type, which were uniformly derived from their spiro parents. Two synthetic routes, both featuring catalyst-controlled enantioselective induction, have been designed, leading to the convenient construction of a large library of both *C*_2_-symmetric and non-*C*_2_-symmetric derivatives of 2,2′,3,3′-tetrahydro-1,1′-spirobi­[phenalene]-9,9′-diol (SPHENOL), all with high efficiency and enantiomeric excess. Preliminary studies have demonstrated their superior performance as backbones of diverse effective chiral catalysts and ligands, illustrating their potential in asymmetric synthesis.

## Graphical abstract

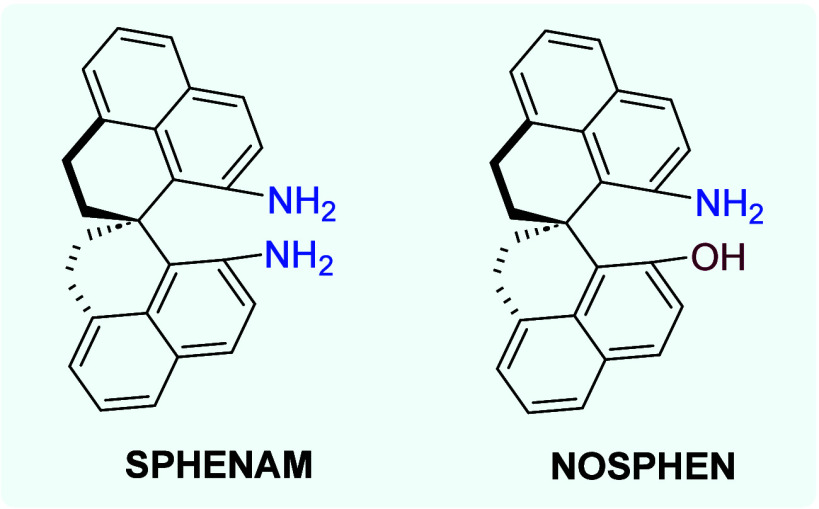

## Introduction

Modern asymmetric synthesis has been substantially advanced by the design of new effective chiral catalysts, particularly those with good accessibility.^1^ The *C*_2_-symmetric axially chiral 1,1′-binaphthyl skeleton (e.g., in BINOL) is an extraordinary example that has been broadly applied in an asymmetric synthesis.^2^ Specifically, the introduction of an amine functionality can further empower this chiral backbone, in view of the exceptional applications of chiral amines both as ligands in an organic synthesis and as pharmacophores in medicinal chemistry.^3^ As a result, BINAM and NOBIN, corresponding to mono- and diamine analogs of BINOL (Figure 1A), have been well-studied regarding their synthesis and application in various contexts.^4,5^

**Figure 1:**
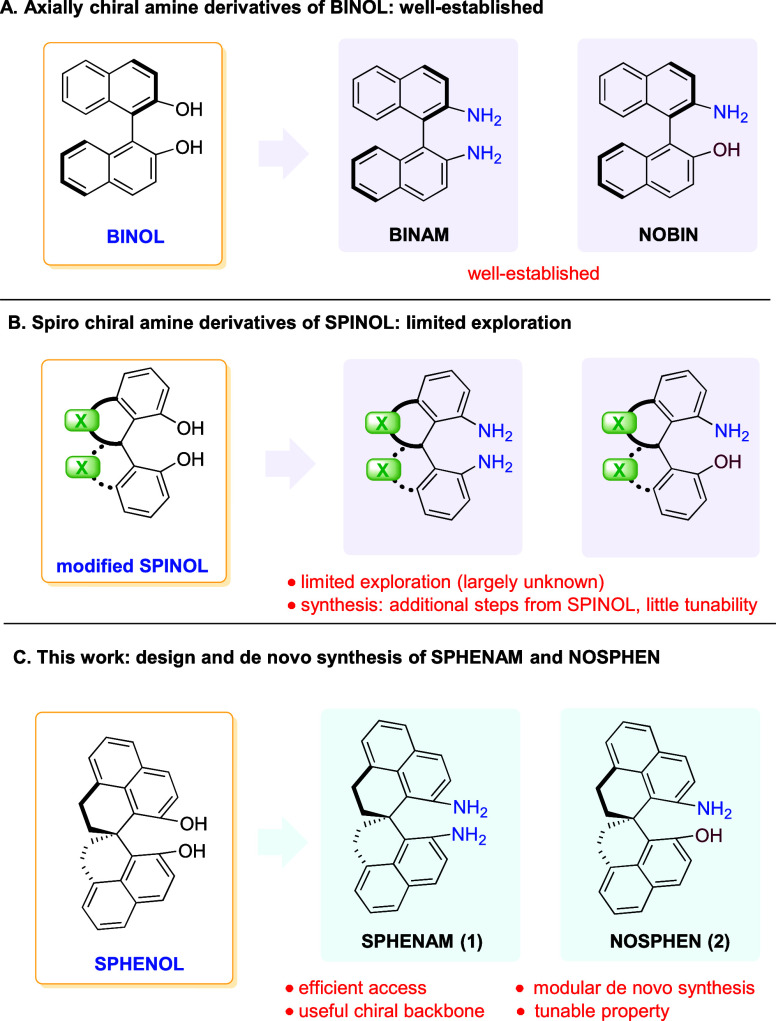
Introduction to the amine derivatives of BINOL, SPINOL, and SPHENOL.

Meanwhile, *C*_2_-symmetric spiro chiral architectures have also gained tremendous success in serving as chiral catalyst backbones.^6^ For example, 1,1′-spirobiindane-7,7′-diol (SPINOL) has been demonstrated as one of the most successful and privileged examples of this family, permitting a myriad of diverse high-performing chiral catalysts to be designed and applied in numerous efficient asymmetric processes that are otherwise less efficient or not as straightforward (Figure 1B).^7^ Moreover, the exceptional performance of SPINOL-derived catalysts has stimulated unfading enthusiasm in modifying this structure into diverse analogs.^8^ However, it is somewhat surprising that, among all the modifications, the introduction of amine functionality directly to the two arene motifs has been very limitedly explored.^9–11^ Compared with BINAM and NOBIN, the performance of the amine counterparts of SPINOL has remained essentially uncovered, although sporadic syntheses have been documented (Figure 1B).^10^ The paucity of studies of these monoamine and diamine derivatives might be partly related to their accessibility. In fact, all the current few examples of this type have been prepared by derivatizations of the SPINOL-based structures, which not only require additional synthetic steps but also have limited flexibility to modify to some degree.^9,10^ Thus, a de novo synthesis without relying on the spiro parents would be expected to offer new opportunities to fully uncover the potential of these molecules and facilitate their applications.

Recently, our laboratory has designed a spirocyclic chiral structure, SPHENOL,^12^ that combines the structural features and advantages of BINOL and SPINOL.^13^ Moreover, its synthesis has also benefited from the efficient intramolecular spirocyclization, owing to the high nucleophilicity of 2-naphthol with outstanding enantioselectivity and regioselectivity.^12^ We envisioned that the amine analogs of SPHENOL would be able to enjoy a similar enantioselective spirocyclization, thus obviating a long sequence via SPHENOL. Here, we present our progress in the design, synthesis, and application of the diamine analog 2,2′,3,3′-tetrahydro-1,1′-spirobi­[phenalene]-9,9′-diamine (SPHENAM) and the monoamine analog 9′-amino-2,2′,3,3′-tetrahydro-1,1′-spirobi­[phenalen]-9-ol (NOSPHEN) (Figure 1C).

## Results and Discussion

We began our study by designing a short sequence for the synthesis of SPHENAM (**1**) from the simple 7-bromo-1-naphthaldehyde **5** (Table 1). A base-promoted aldol condensation with acetone furnished dienone **6**. Further Rh/C-catalyzed hydrogenation followed by Pd-catalyzed amination with benzophenone imine and acidic workup quickly provided the key intermediate **3a**, a linear ketone bearing two 2-naphthylamine units. Next, an enantioselective spirocyclization was expected to proceed by two sequential intramolecular Friedel–Crafts-type cyclizations via the key aza-*ortho*-quinone methide (**aza-** ***o*** **-QM**) intermediate enabled by chiral Bro̷nsted acid catalysis.

**Table 1:**
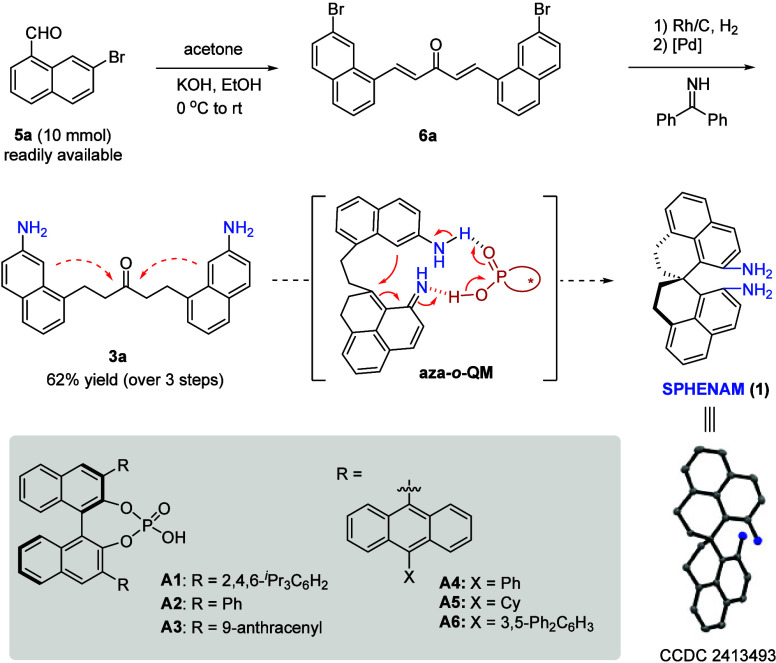
Synthesis of SPHENAM

entry	CPA	yield (%)^a^	ee (%)^a^
1	(*R*)-**A1**	>95%	11%
2	(*R*)-**A2**	>95%	5%
3	(*R*)-**A3**	>95%	18%
4	(*R*)-**A4**	>95%	55%
5	(*R*)-**A5**	>95%	70%
6	(*R*)-**A6**	>95%	78%
7^b^	(*R*)-**A6**	5%	95%
8^c^	(*R*)-**A6**	90%	92%
9^d^	(*R*)-**A6**	90%	91%
10^e^	(*R*)-**A6**	89% (85%)^f^	91% (>99%)^f^

a Reaction scale: **3a** (0.05 mmol), catalyst (10 mol %) in DCM (1.0 mL), 50 °C. The yield was based on analysis of the ^1^H NMR spectrum of the crude reaction mixture using CH_2_Br_2_ as an internal standard. The ee value was determined by chiral HPLC.

b Run in toluene.

c Run in DCM/toluene (v/v = 1:1, 1.0 mL).

d **3a** (3.0 mmol) and (*R*)-**A6** (5 mol %). Isolated yield.

e **3a** (6.5 mmol) and (*R*)-**A6** (2 mol %). Isolated yield.

f The yield and ee in parentheses were after crystallization.

We then evaluated different catalysts for this step. It is encouraging to find that the desired cyclization proceeded to form the desired spiro diamine **1** in high yield with chiral phosphoric acid (CPA) catalysis,^14^ although both the substrate and product have the basic diamine functionality. However, with the well-known BINOL-derived CPAs **A1**–**A3**, the enantioselectivity was rather low (entries 1–3). Nevertheless, further evaluation indicated that other CPAs modified from **A3** with additional substituents were able to improve the enantioselectivity significantly (entries 4–6). Among them, **A6** was identified as the best catalyst, providing the desired SPHENAM in almost quantitative yield and with 78% enantiomeric excess (ee) in refluxing DCM. Notably, no oligomerization or intermolecular imine formation was observed. Brief NMR titration experiments indicated that the high efficiency benefited from the reversible Bro̷nsted acid activation, but not complete protonation, of the amine functionality. Monitoring the reaction progress by NMR did not allow the detection of any intermediates, suggesting that the second cyclization step might be fast.

Replacing the solvent with toluene led to a dramatic decrease in reactivity, although with high enantioselectivity (95% ee, entry 7). Then, a mixture of DCM and toluene was used as the solvent, which resulted in both good yield and enantioselectivity (entry 8). The reaction at a large scale remained very efficient, even with only 2 mol % of catalyst (entry 10). Moreover, the product was crystalline and could be easily enriched to an enantiopure form by simple crystallization almost without loss of material (entry 10). The structure and absolute configuration of SPHENAM were unambiguously confirmed by X-ray crystallography. It is worth noting that, distinct from the amine analogs of SPINOL and those modified SPINOL structures that had to be derived from their spiro parents, our synthesis of SPHENAM features a de novo synthesis that does not rely on the parent SPHENOL. The high efficiency and enantiocontrol indeed benefited from the high nucleophilicity of naphthylamine and the key enantiodetermining transition state featuring bifunctional activation on the well-organized **aza-** ***o*** **-QM** intermediate.^15^

The above protocol could also be applied to the expansion of the chiral spiro diamine library. For example, a range of chiral SPHENAM analogs bearing different substituents at various positions of the naphthalene rings was easily prepared by simply varying the starting materials (Scheme 1). Both *C*_2_-symmetric and non-*C*_2_-symmetric analogs could be made with high chemical efficiency and enantiomeric excess. It is expected that these sterically and electronically different chiral diamines may serve different purposes in asymmetric catalysis.

**Scheme 1:**
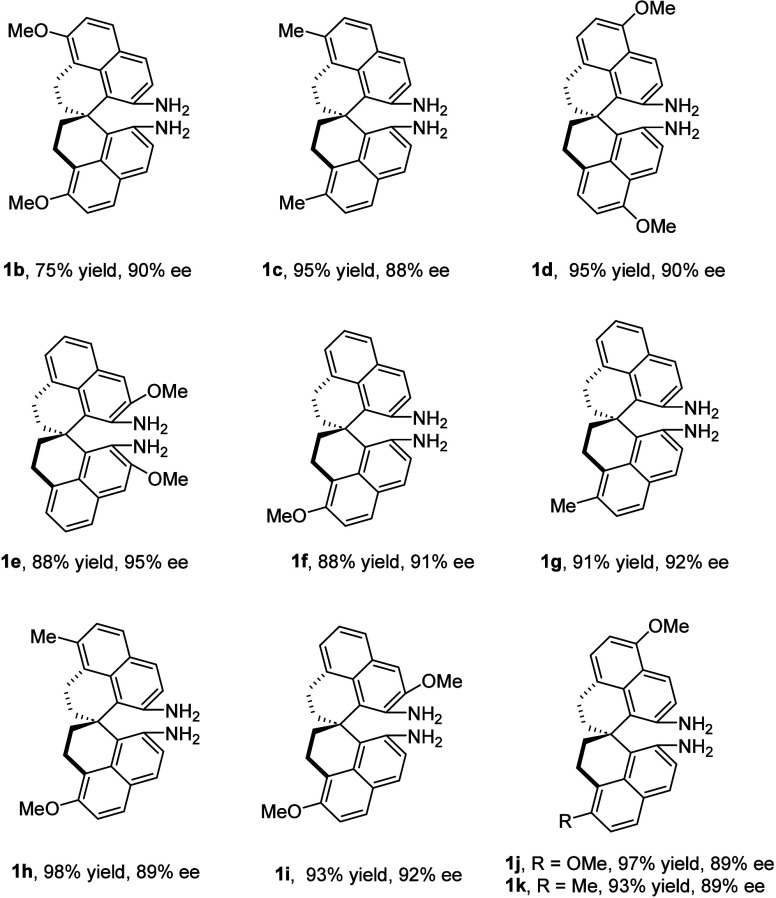
Synthesis of SPHENAM Analogs^a^

a Reaction scale: **3** (0.3 mmol), (*R*)-**A6** (5 mol %), DCM/toluene (v/v = 1:1, 6.0 mL), 50 °C. Isolated yield. The ee value was determined by chiral HPLC analysis.

To further tune the features and permit more structural diversifications, we also investigated an alternative synthetic protocol (Scheme 2). Dienone **6** was subjected to a Rh-catalyzed enantioselective conjugate addition using PhB­(OH)_2_ as a nucleophile and chiral diene **L** as a ligand. The reaction at 5.0 mmol scale provided remarkably efficient access to ketone **7a** in 85% yield as a pure enantiomer and single diastereomer (>99% ee). Next, the amination was easily achieved with no loss of enantiopurity, providing chiral amine **8a** in 89% yield. Finally, in the presence of a racemic phosphoric acid RPA, the spirocyclization of **8a** proceeded with complete diastereocontrol to form diphenyl-substituted SPHENAM **9a** in 82% yield as a single diastereomer. Its structure was also confirmed by X-ray crystallography.

**Scheme 2:**
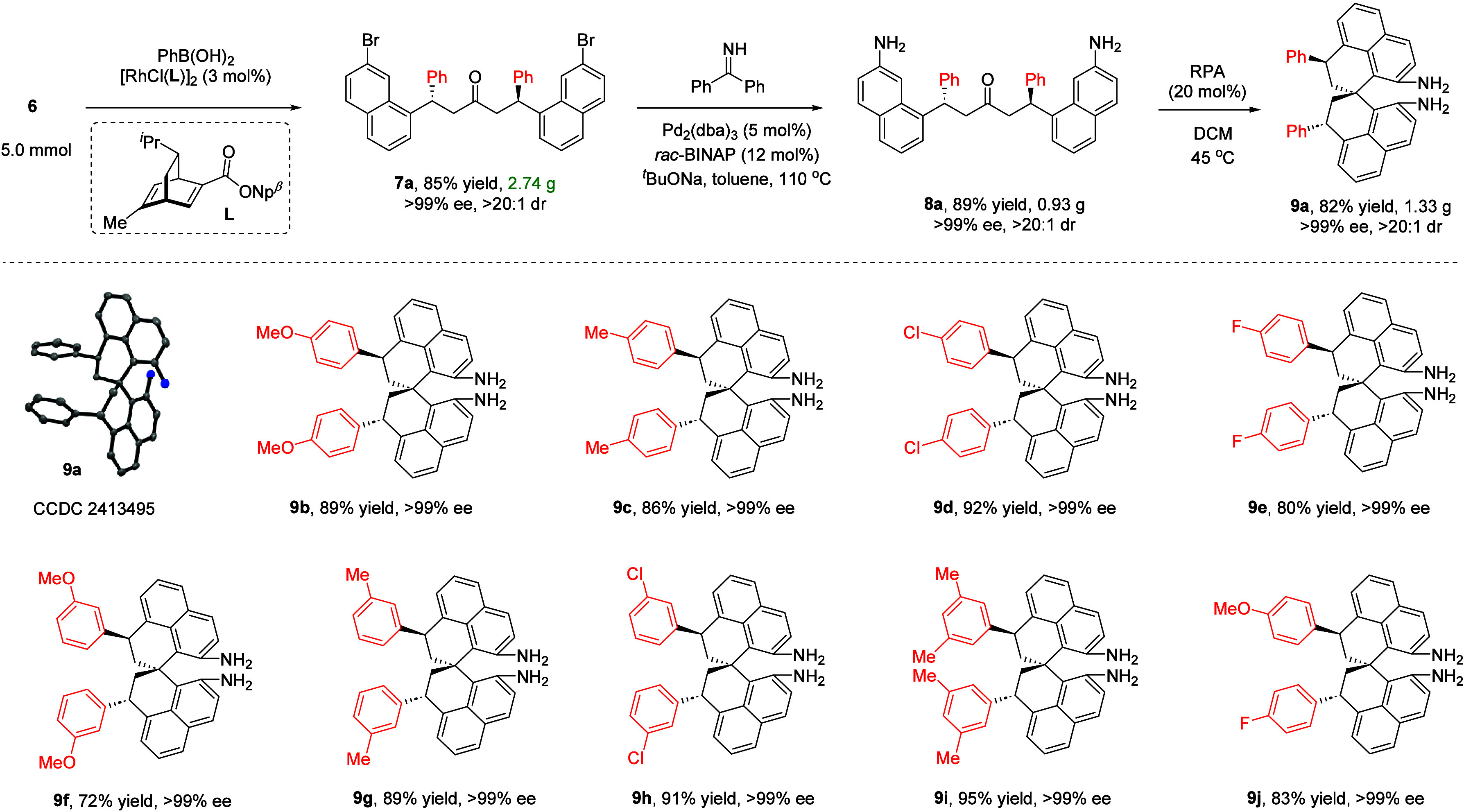
Alternative Synthesis of SPHENAM Analogs^a^

a Reaction conditions: **8** (0.2 mmol), RPA (20 mol %), DCM (2.0 mL). Isolated yield. RPA = (*rac*)-2,2′-biphthyl hydrogenphosphate. The ee value was determined by chiral HPLC analysis.

We also examined the scope of this protocol. A range of chiral SPHENAM analogs bearing two aryl substituents at the saturated linker was obtained with high efficiency and, most importantly, all in enantiopure forms. Notably, although the two aryl groups are the same in most cases, this protocol is also applicable for the incorporation of two different aryl substituents (e.g., **9j**), maintaining high chemical efficiency and stereocontrol.

With the success achieved for the chiral SPHENAM synthesis, we were curious about the non-*C*_*2*_-symmetric NOSPHEN (**2**), the monoamine analog of SPHENOL. It was expected that the selective introduction of only one amine unit to this structure may not be as straightforward as the symmetric diamine since multiple selectivity issues will be encountered. The synthesis started with two sequential aldol condensation steps of acetone initially with 7-hydroxyl-1-naphthaldehyde **10** and then with 7-nitro-1-naphthaldehyde **12**, which afforded unsymmetrical dienone **12** in 77% yield over three steps (Scheme 3A and more details in the Supporting Information). Subsequent hydrogenation catalyzed by PtO_2_ reduced both two C=C bonds and the nitro group in one operation, resulting in key intermediate **4a**. Similar to the synthesis of SPHENAM, the enantioselective spirocyclization was efficiently achieved with **A6** as catalyst, delivering NOSPHEN in 76% yield and 97% ee, which was further enriched to the enantiomerically pure form after simple crystallization without obvious loss of material.

**Scheme 3:**
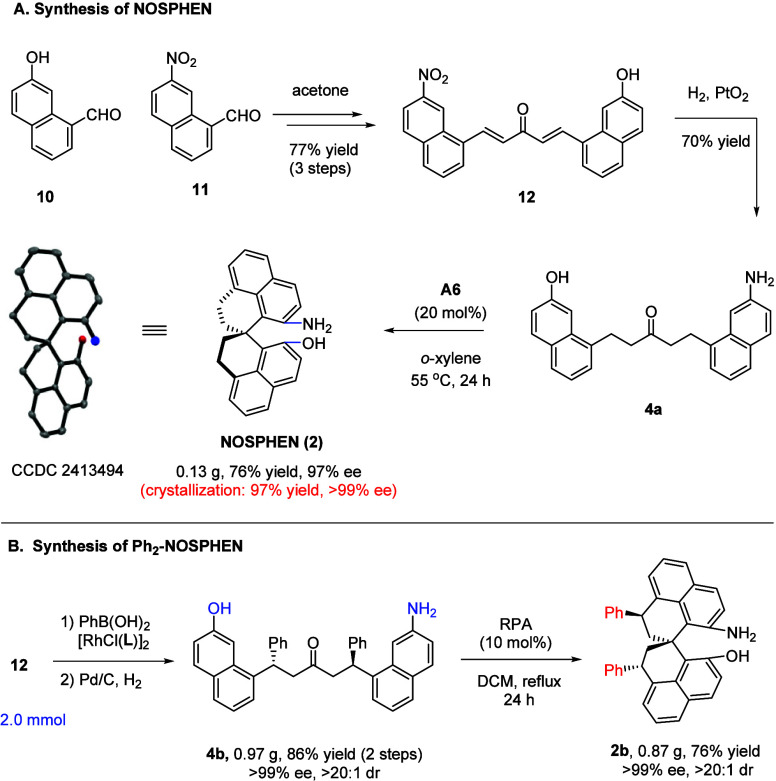
Synthesis of NOSPHEN and Ph_2_-NOSPHEN

A modification of this structure was also demonstrated by following a similar route involving Rh-catalyzed enantioselective conjugate addition followed by substrate-directed spirocyclization. The corresponding diphenyl-substituted NOSPHEN was also obtained in enantiopure form with high overall efficiency (Scheme 3B). SPHENAM and NOSPHEN represent a family of new chiral spiro structures that have been limitedly studied for asymmetric catalysis.

To explore their potential applications, we carried out preliminary studies. These two backbones were employed to install different functionalities. For example, chiral bis­(phosphine) **L1** (or **Ph**_**2**_ **-L1**) could be easily obtained by treating the corresponding diamine SPHENAM (or Ph_2_-SPHENAM) with base and Ph_2_PCl (Scheme 4A). The two amine units can also serve as points to graft urea and thiourea motifs, corresponding to potential organocatalysts **L2** and **L3**. The diamide **L4** was also generated with high efficiency. Moreover, sequential modifications of the amine and alcohol functionalities in NOSPHEN also allowed the synthesis of some useful catalysts and ligands. For example, a phosphite ligand bearing a pyridine motif was easily prepared. Finally, the thiourea/phosphine molecule **L6** was synthesized with good efficiency. In all these cases, the enantiomeric excess of the parents remained intact.

**Scheme 4:**
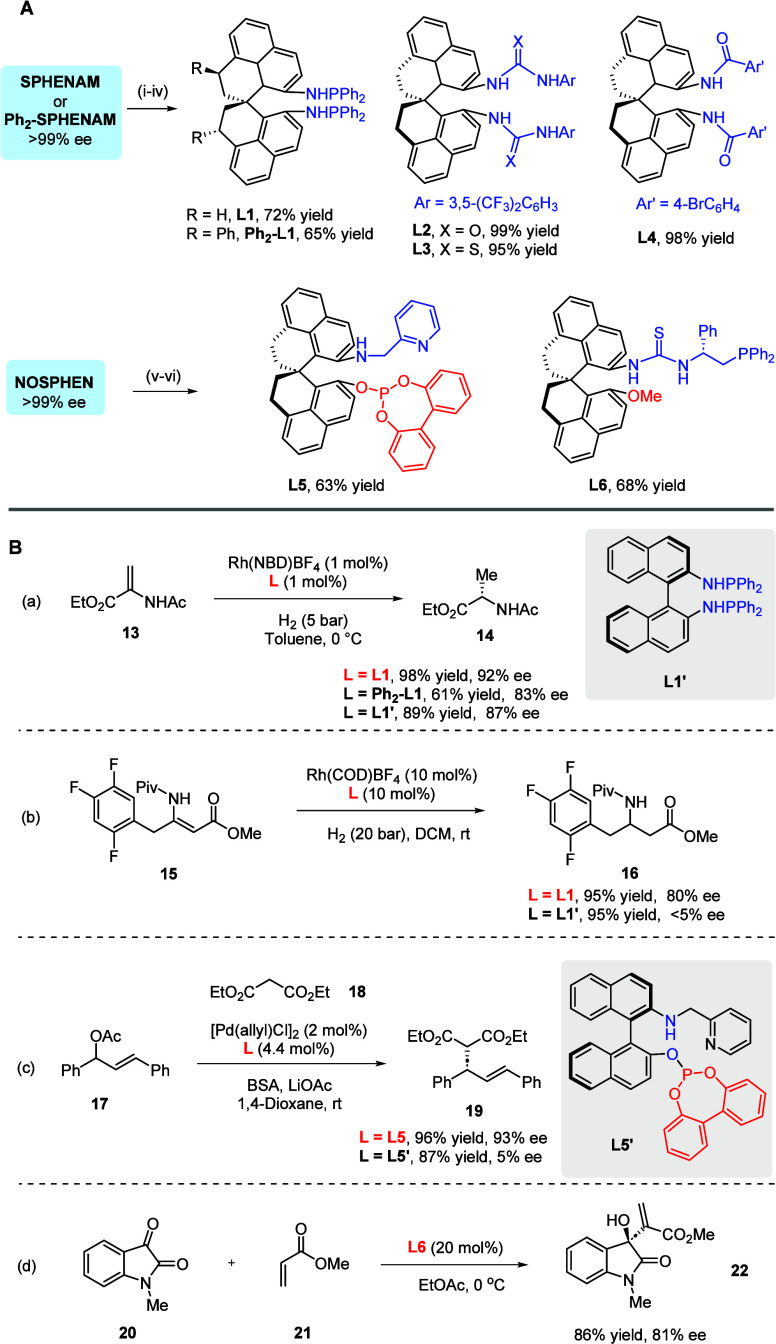
Application of SPHENAM and NOSPHEN Derivatives^a^

a Reaction conditions: (i) ^*n*^BuLi, ClPPh_2_, THF. (ii) 3,5-bis­(trifluoromethyl)­phenyl isocyanate, DCM. (iii) 3,5-bis­(trifuoromethyl)­phenyl isothiocyanate, DCM. (iv) 4-bromobenzoyl chloride, Et_3_N, DCM. (v) (1) 2-pyridinecarboxaldehyde, NaBH­(OAc)_3_, DCE; (2) phosphorochloridite, Et_3_N, DCM. (vi) (1) DEAD, PPh_3_; (2) CSCl_2_, pyridine; (3) (1*R*)-2-diphenylphosphanyl-1-phenylethanamine, pyridine.

Next, potential applications of these derivatives were investigated. For example, with **L1** as ligand, the Rh-catalyzed hydrogenation of enamide **13** provided the enantioenriched α-amino acid derivative **14** in quantitative yield and 92% ee, while **Ph**_**2**_ **-L1** and BINAM-derived **L1**′**** resulted in decreased efficiency and enantioselectivity (Scheme 4B,a). Moreover, the enantioselective hydrogenation of **15** provided efficient access to β-amino ester **16** in 80% ee, a promising intermediate toward the synthesis of an important pharmaceutical ingredient, sitagliptin.^16^ In sharp contrast, **L1**′**** resulted in completely no enantiomeric control under otherwise identical conditions (Scheme 4B,b). The NOSPHEN-derived phosphite **L5** also served as an effective ligand in the Pd-catalyzed asymmetric allylic substitution of **17**, leading to allylic alkylation product **19** in 96% yield with 93% ee. Notably, the NOBIN-derived ligand **L5′** delivered **19** in 87% yield but with a drastically reduced enantioselectivity of 5% ee, suggesting obvious advantages of the new spirocyclic architecture (Scheme 4B,c). Finally, the chiral thiourea **L6** also served as an effective organocatalyst in the asymmetric Morita–Baylis–Hillman (MBH) reaction of *N*-methyl isatin **20** with methyl acrylate **21** (Scheme 4B,d).

Preliminary analysis of the structural properties of SPHENAM and NOSPHEN was based on X-ray crystallography. The dihedral angles of SPHENAM and NOSPHEN are 66.44 and 65.69°, respectively, which are close to those of SPHENOL (68.52°). Similarly, the N···N distance in SPHENAM (3.59 Å) and the N···O distance in NOSPHEN (3.57 Å) are comparable to the O···O distance in SPHENOL (3.50 Å), indicative of a similar three-dimensional architecture and structural rigidity. However, the lack of X-ray data of the corresponding amine derivatives of SPINOL precludes a comprehensive comparison. A correlation between the structure and performance would require further studies.

## Conclusions

We designed a family of new spiro chiral mono- and diamine analogs of SPHENOL as well as their de novo enantioselective synthesis by a short sequence from simple substrates. This study complements the limited exploration of the counterparts of SPINOL and its modified analogs, even though they have been privileged catalyst backbones known for over two decades. Without SPHENOL as a precursor, the convenient enantioselective synthesis of SPHENAM and NOSPHEN also represents a notable advantage over the limited spirocyclic precedents of this type, which were uniformly derived from their spiro parents. With a suitable chiral phosphoric acid catalyst, the facile and well-controlled enantioselective spirocyclization of the linear ketones bearing highly nucleophilic 2-naphthol and 2-naphthylamine served as the key to success and the intrinsic origin of this advantage. An alternative synthetic route featuring Rh-catalyzed diarylation of the enone precursors followed by spirocyclization provided another family of analogs bearing additional substituents on the backbone. Thus, a large library of both *C*_2_-symmetric and non-*C*_2_-symmetric spiro chiral mono- and diamines has been easily prepared, all with high efficiency and enantiomeric excess. Finally, preliminary studies have demonstrated their superior performance as backbones of diverse effective chiral catalysts and ligands in mechanistically unrelated reactions, illustrating their great potential for asymmetric synthesis.

## Supplementary Material



## Notes

Additional experimental details and spectroscopic data of all compounds (PDF)
