# Supplementary material for: Design, Synthesis, and Application of a Type of Chiral Spirocyclic Naphthylamines
Source: J Am Chem Soc. 2025 Sep 29;147(40):36774–80. doi: 10.1021/jacs.5c12568 (PMC12512103; doi:10.1021/jacs.5c12568)
Supplement: Supplementary file 1 [file ja5c12568_si_001.pdf]

Supporting Information

Design, Synthesis, and Application of a Type of Chiral Spirocyclic Naphthylamines

Ronghua Zhang, Minghui Zhu, and Jianwei Sun*

Department of Chemistry and the Hong Kong Branch of Chinese National Engineering Research Centre for Tissue Restoration & Reconstruction, the Hong Kong University of Science and Technology, Clear Water Bay, Kowloon, Hong Kong SAR, China

Table of Contents

I. General Information S-2

II. Preparation of SPHENAMs S-3

III. Preparation of Diaryl SPHENAMs S-38

IV. Preparation of NOSPHEN S-62

V. Preparation of Diphenyl NOSPHEN S-66

VI. Synthesis of SPHENAM Derivatives S-69

VII. Synthesis of NOSPHEN and NOBIN Derivatives S-74

VIII. Application of SPHENAM and NOSPHEN Derivatives S-80

IX. Product Structure Determination S-87

X. References S-93

NMR Spectra and HPLC Traces

I. General Information

Flash column chromatography was performed over silica gel (200-300 mesh) purchased from Qingdao Puke Co., China. All air or moisture sensitive reactions were conducted in oven-dried glassware under nitrogen atmosphere using anhydrous solvents. Anhydrous dichloromethane, toluene, diethyl ether, and tetrahydrofuran were purified by the Innovative® solvent purification system. Chemicals were purchased from commercial suppliers and used without further purification unless otherwise stated. ^{1}H, ^{13}C, ^{19}F NMR, ^{31}P NMR spectra were collected on a Bruker AV 400 MHz NMR spectrometer using residue solvent peaks as an internal standard (^{1}H NMR: $CDCl_3$ at 7.26 ppm, acetone-d_6 at 2.05 ppm, CD_3OD at 3.31 ppm; ^{13}C NMR: $CDCl_3$ at 77.00 ppm, and acetone-d_6 at 29.84 ppm, CD_3OD at 49.00 ppm). Data for ^{1}H NMR are recorded as follows: chemical shift (δ, ppm), multiplicity (s = singlet; d = doublet; t = triplet; q = quartet; p = pentet; sept = septet; m = multiplet; br = broad), coupling constant (Hz), integration. Mass spectra were collected on an Agilent GC/MS 5975C system, a MALDI Micro MX mass spectrometer, or an API QSTAR XL System. IR spectra were recorded on Bruker TENSOR 27 spectrometer and reported in terms of frequency of absorption (cm^{-1}). Optical rotations were measured on JASCO P-2000 polarimeter with $[\alpha]^{D}$ values reported in degrees; concentration (c) is in 10 mg/mL. The enantiomeric excess values were determined by chiral HPLC using an Agilent 1200 LC instrument with Daicel CHIRALPAK® AD-H, CHIRALPAK® IA-3, CHIRALPAK® AD-3, CHIRALCEL® OD-3 and CHIRALCEL® OD-H columns.

II. Preparation of SPHENAMs

1. Synthesis of the Naphthyl Aldehydes 5

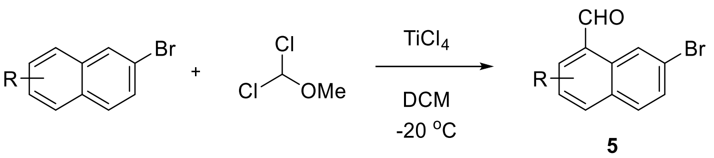

5

At -20 °C under N_2, to a solution of 1,1-dichlorodimethyl ether in anhydrous DCM was added dropwise $TiCl_4$. Then the mixture was stirred at the same temperature for 30 min, after which a solution of 2-bromonaphthalene in anhydrous DCM was added dropwise. The mixture was stirred at -20 °C. Upon completion, the mixture was carefully poured into an aqueous solution of hydrochloric acid at 0 °C. The two layers were separated, and the aqueous layer was extracted with DCM (50 mL × 3). The combined organic layers were washed with a saturated aqueous solution of $NaHCO_3$ (100 mL × 3), brine (100 mL), dried over Na_2SO_4, and filtered. The filtrate was concentrated and purified by silica gel column chromatography followed by recrystallization to afford the pure product.

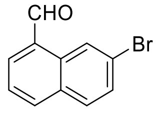

5a

7-Bromo-1-naphthaldehyde (5a) was prepared as a white solid from 2-bromonaphthalene (20.7 g, 100 mmol), 1,1-dichlorodimethyl ether (13.8 g, 120 mmol), $TiCl_4$ (38.0 g, 200 mmol) and anhydrous DCM (100 mL) according to the above procedure (eluent: hexanes/DCM = 1:1) in 62% yield (14.5 g).

^{1}H NMR (400 MHz, $CDCl_3$) δ 10.32 (s, 1H), 9.51 (s, 1H), 8.07 (d, *J* = 8.2 Hz, 1H), 8.01 (d, *J* = 6.9 Hz, 1H), 7.82 – 7.73 (m, 1H), 7.73 – 7.55 (m, 2H).

^{13}C NMR (101 MHz, $CDCl_3$) δ 193.1, 137.7, 135.1, 132.1, 131.2, 130.6, 130.4, 129.8, 127.5, 125.3, 124.1.

HRMS (CI) Calcd for $C_{11}H_7BrO$ (M): 233.9680, Found: 233.9689.

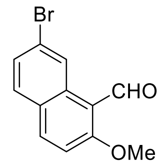

5b

7-Bromo-2-methoxy-1-naphthaldehyde (5b) was prepared as a white solid from 2-bromo-7-methoxynaphthalene (5.90 g, 25 mmol), 1,1-dichlorodimethyl ether (5.70 g, 50 mmol), $TiCl_4$ (9.50 g, 50 mmol) and anhydrous DCM (25 mL) according to the above procedure (eluent: hexanes/DCM = 2:1) in 93% yield (6.11 g).

^{1}H NMR (400 MHz, $CDCl_3$) δ 10.79 (s, 1H), 9.49 (s, 1H), 7.98 (d, *J* = 9.1 Hz, 1H), 7.59 (d, *J* = 8.6 Hz, 1H), 7.54 – 7.43 (m, 1H), 7.27 (d, *J* = 9.2 Hz, 1H), 4.04 (s, 3H).

^{13}C NMR (101 MHz, $CDCl_3$) δ 191.4, 164.3, 137.4, 132.4, 129.6, 128.2, 127.2, 126.8, 125.1, 115.4, 112.7, 56.5.

HRMS (CI) Calcd for $C_{12}H_9BrO_2$ (M): 263.9786, Found: 263.9785.

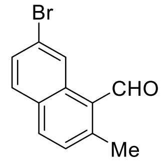

5c

7-Bromo-2-methyl-1-naphthaldehyde (5c) was prepared as a white solid from 2-bromo-7-methylnaphthalene (3.26 g, 15 mmol), 1,1-dichlorodimethyl ether (3.42 g, 30 mmol), $TiCl_4$ (5.70 g, 30 mmol) and anhydrous DCM (30 mL) according to the above procedure (eluent: hexanes/DCM = 2:1) in 71% yield (2.64 g).

^{1}H NMR (400 MHz, $CDCl_3$) δ 10.76 (s, 1H), 9.17 (d, *J* = 2.0 Hz, 1H), 7.80 (d, *J* = 8.4 Hz, 1H), 7.58 (d, *J* = 8.7 Hz, 1H), 7.50 (dd, *J* = 8.7, 1.9 Hz, 1H), 7.26 (d, *J* = 8.4 Hz, 1H), 2.75 (s, 3H).

^{13}**C NMR** (101 MHz, $CDCl_3$) δ 192.5, 144.0, 134.1, 131.8, 130.7, 130.0, 129.5, 129.3, 127.0, 123.6, 99.9, 19.7.

HRMS (CI) Calcd for $C_{12}H_9BrO$ (M): 247.9837, Found: 247.9841.

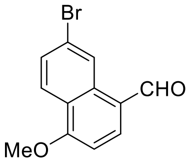

5d

7-Bromo-4-methoxy-1-naphthaldehyde (5d) was prepared as a white solid from 6-bromo-1-methoxynaphthalene (2.16 g, 9.1 mmol), 1,1-dichlorodimethyl ether (1.70 g, 15 mmol), $TiCl_4$ (2.84 g, 15 mmol) and anhydrous DCM (20 mL) according to the above procedure (eluent: hexanes/DCM = 2:1) in 80% yield (1.92 g).

^{1}**H NMR** (400 MHz, $CDCl_3$) δ 10.06 (s, 1H), 9.47 (d, *J* = 2.0 Hz, 1H), 8.09 (d, *J* = 8.9 Hz, 1H), 7.83 (d, *J* = 8.1 Hz, 1H), 7.58 (dd, *J* = 9.0, 2.0 Hz, 1H), 6.86 (d, *J* = 8.1 Hz, 1H), 4.06 (s, 3H).

^{13}**C NMR** (101 MHz, $CDCl_3$) δ 191.8, 160.7, 140.7, 132.5, 129.7, 127.3, 124.8, 124.0, 123.9, 123.8, 103.2, 56.0.

HRMS (CI) Calcd for $C_{12}H_9BrO_2$ (M): 263.9786, Found: 263.9785.

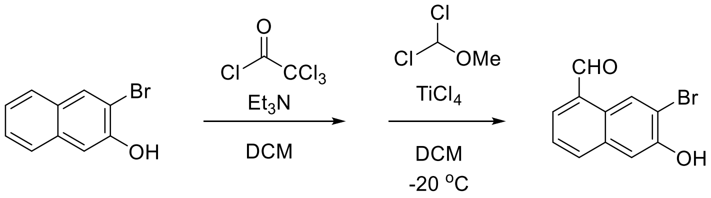

5e

7-Bromo-6-hydroxy-1-naphthaldehyde (5e). To a solution of 3-bromonaphthalen-2-ol (3.55 g, 16 mmol) in anhydrous DCM (20 mL) were added triethylamine (8.08 g, 80 mmol) and trichloroacetyl chloride (4.35 g, 24 mmol). Then the mixture was stirred at room temperature for 4 h. Upon completion, the mixture was filtered through a glass frit and the filter cake was

washed with DCM (10 mL × 3). The filtrate was evaporated and used directly without further purification.

At -20 °C under N_2, to a solution of 1,1-dichlorodimethyl ether (2.76 g, 24 mmol) in anhydrous DCM (20 mL) was added dropwise $TiCl_4$ (4.60 g, 24 mmol). Then the mixture was stirred at the same temperature for 30 min, after which a solution of the above ester intermediate in anhydrous DCM (30 mL) was added dropwise. The mixture was stirred at -20 °C for 6 h. Upon completion, the mixture was carefully poured into an aqueous solution of hydrochloric acid (2.0 M, 50 mL) at 0 °C. The two layers were separated, and the aqueous layer was extracted with DCM (50 mL × 3). The combined organic layers were washed with brine (200 mL), dried over Na_2SO_4, and filtered. The filtrate was evaporated and then re-dissolved in EtOH (50 mL), to which was added K_2CO_3 (4.14 g, 30 mmol). The mixture was stirred vigorously for 1 h. Upon completion, the mixture was carefully poured into an aqueous solution of hydrochloric acid (2.0 M, 100 mL) at 0 °C, which was extracted with EtOAc (50 mL × 3). The combined organic layers were washed with a saturated aqueous solution of $NaHCO_3$ (50 mL × 3), brine (100 mL), and then dried over Na_2SO_4 and filtered. The filtrate was concentrated and purified by silica gel flash chromatography (eluent: hexanes/ EtOAc = 4:1) to afford **5e** as a grey solid in 32% yield (1.28 g).

^{1}H NMR (400 MHz, DMSO-d_6) δ 10.24 (s, 1H), 9.38 (s, 1H), 8.10 (d, J = 8.3 Hz, 1H), 8.00 (d, J = 6.9 Hz, 1H), 7.68 (dd, J = 8.3, 7.1 Hz, 1H), 7.44 (s, 1H).

^{13}C NMR (101 MHz, DMSO-d_6) δ 194.7, 152.6, 135.5, 134.3, 133.6, 129.8, 128.8, 126.1, 124.3, 116.3, 110.8.

HRMS (CI) Calcd for $C_{11}H_7BrO_2$ (M): 249.9629, Found: 249.9625.

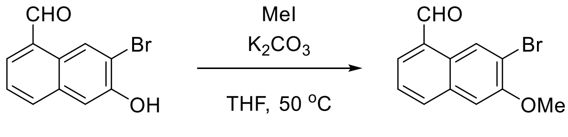

5e **5f**

7-Bromo-6-methoxy-1-naphthaldehyde (5f). To a solution of aldehyde **5e** (590

mg, 2.36 mmol) in THF (10 mL) were added K_2CO_3 (680 mg, 5.0 mmol) and iodomethane (670 mg, 4.72 mmol). The mixture was stirred at 50 °C for 12 h and then filtered through a glass frit and the filter cake was washed with DCM (10 mL × 3). The filtrate was concentrated, and the crude product was purified by silica gel flash chromatography (eluent: hexanes/DCM = 1:1) to afford **5f** as a white solid in 97% yield (602 mg).

^{1}H NMR (400 MHz, $CDCl_3$) δ 10.21 (s, 1H), 9.48 (s, 1H), 7.96 – 7.88 (m, 1H), 7.85 – 7.72 (m, 1H), 7.57 (dd, *J* = 8.3, 7.1 Hz, 1H), 7.14 (s, 1H), 3.99 (s, 3H).

^{13}C NMR (101 MHz, $CDCl_3$) δ 193.3, 154.1, 135.4, 134.2, 133.7, 130.2, 129.8, 125.7, 117.2, 106.8, 56.1.

HRMS (CI) Calcd for $C_{12}H_9BrO_2$ (M): 263.9786, Found: 263.9787.

2. Synthesis of the Symmetric Ketones S3

Step 1: Aldol Condensation

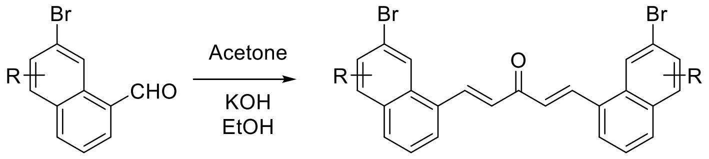

5 **6**

At 0 °C, to a solution of the 7-bromo-1-naphthaldehyde (2.0 equiv) and acetone (1.0 equiv) in absolute EtOH was slowly added KOH (2.0 equiv), which resulted in a yellow suspension. The mixture was stirred at the same temperature. Upon completion (~ 12 h), the mixture was filtered through glass frit, and the yellow filter cake was washed with water (20 mL × 3) followed by EtOH (20 mL × 3). This yellow solid was dried under vacuum to afford the desired dienone, which was used directly for the next step without further purification.

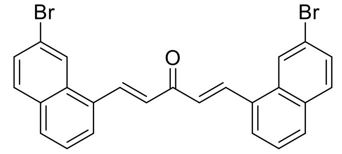

6a

(1*E*,4*E*)-1,5-Bis(7-bromonaphthalen-1-yl)penta-1,4-dien-3-one (6a) was prepared as a white solid from 7-bromo-1-naphthaldehyde **5a** (2.34 g, 10.0 mmol), KOH (560 mg, 10.0 mmol), acetone (290 mg, 5.0 mmol) and absolute EtOH (20 mL) according to the above procedure in 91% yield (2.23 g). This yellow solid was used directly for the next step without further purification.

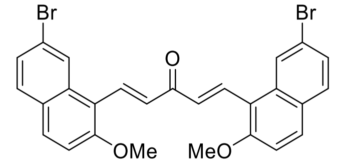

6b

(1*E*,4*E*)-1,5-Bis(7-bromo-2-methoxynaphthalen-1-yl)penta-1,4-dien-3-one (6b) was prepared as a yellow solid from 7-bromo-2-methoxy-1-naphthaldehyde **5b** (528 mg, 2.0 mmol), KOH (112 mg, 2.0 mmol), acetone (58.1 mg, 1.0 mmol) and absolute EtOH (10 mL) according to the General procedure in 92% yield (506 mg). This yellow solid was used directly for the next step without further purification.

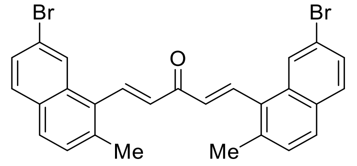

6c

(1*E*,4*E*)-1,5-Bis(7-bromo-2-methylnaphthalen-1-yl)penta-1,4-dien-3-one (6c) was prepared as a yellow solid from 7-bromo-2-methyl-1-naphthaldehyde **5c** (496 mg, 2.0 mmol), KOH (112 mg, 2.0 mmol), acetone (58.1 mg, 1.0 mmol) and absolute EtOH (10 mL) according to the General procedure in 96% yield (495 mg). This yellow solid was used directly for the next step without further

purification.

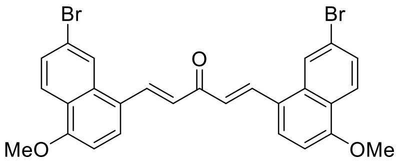

6d

(1*E*,4*E*)-1,5-Bis(7-bromo-4-methoxynaphthalen-1-yl)penta-1,4-dien-3-one (6d) was prepared as a yellow solid from 7-bromo-4-methoxy-1-naphthaldehyde **5d** (792 mg, 3.0 mmol), KOH (118 mg, 3.0 mmol), acetone (87.2 mg, 1.5 mmol) and absolute EtOH (15 mL) according to the above procedure in 67% yield (553 mg). This yellow solid was used directly for the next step without further purification.

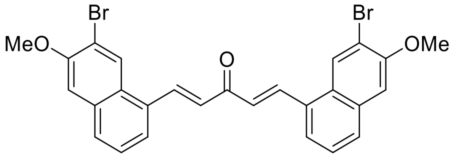

6e

(1*E*,4*E*)-1,5-Bis(7-bromo-6-methoxynaphthalen-1-yl)penta-1,4-dien-3-one (6e) was prepared as a yellow solid from 7-bromo-6-methoxy-1-naphthaldehyde **5f** (528 mg, 2.0 mmol), KOH (112 mg, 2.0 mmol), acetone (58.1 mg, 1.0 mmol) and absolute EtOH (10 mL) according to the above procedure in 97% yield (536 mg). This yellow solid was used directly for the next step without further purification.

Step 2: Hydrogenation

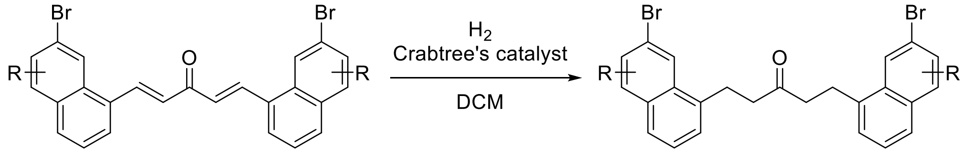

To a solution of the dienone in DCM was added the Crabtree's catalyst (1 mol%).

The mixture was transferred into a Parr® autoclave, which was evaluated and refilled with hydrogen gas for three times and finally pressurized to 15 bar. The mixture was stirred at room temperature for 12 h, and the hydrogen gas was released carefully in a fume hood. The mixture was concentrated, and the crude product was purified by silica gel flash chromatography to afford the desired product.

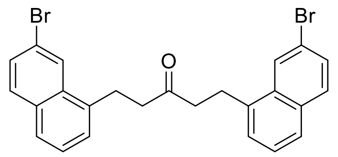

S3a

1,5-Bis(7-bromonaphthalen-1-yl)pentan-3-one (S3a) was prepared as a white solid from **6a** (4.90 g, 10.0 mmol), the Crabtree's catalyst (80.2 mg, 0.1 mmol), and DCM (30 mL) according to the above procedure (eluent: hexanes/DCM = 2:1) in 79% yield (3.90 g).

^{1}H NMR (400 MHz, $CDCl_3$) δ 8.10 (s, 2H), 7.75 – 7.62 (m, 4H), 7.58 – 7.51 (m, 2H), 7.41 – 7.34 (m, 2H), 7.34 – 7.27 (m, 2H), 3.30 (t, *J* = 7.6 Hz, 4H), 2.82 (t, *J* = 7.6 Hz, 4H).

^{13}C NMR (101 MHz, $CDCl_3$) δ 208.6, 136.1, 132.7, 132.2, 130.5, 128.9, 127.0, 126.8, 126.0, 125.7, 43.4, 26.4.

HRMS (CI) Calcd for $C_{25}H_{20}BrNaO$ (M + Na^+): 518.9753, Found: 518.9756.

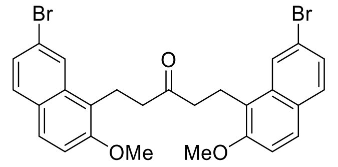

S3b

1,5-Bis(7-bromo-2-methoxynaphthalen-1-yl)pentan-3-one (S3b) was prepared as a white solid from **6b** (825 mg, 1.5 mmol), the Crabtree's catalyst (12 mg, 0.015 mmol), and DCM (15 mL) according to the above procedure (eluent: hexanes/DCM = 2:1) in 82% yield (673 mg).

^{1}H NMR (400 MHz, $CDCl_3$) δ 8.07 (s, 2H), 7.69 (d, *J* = 9.0 Hz, 2H), 7.63 (d, *J* = 8.7 Hz, 2H), 7.39 (dd, *J* = 8.7, 1.8 Hz, 2H), 7.26 (d, *J* = 8.9 Hz, 2H), 3.94 (s, 6H), 3.31 (t, *J* = 8.0 Hz, 4H), 2.71 (t, *J* = 8.0 Hz, 4H).

^{13}C NMR (101 MHz, $CDCl_3$) δ 210.1, 155.0, 133.9, 130.2, 127.9, 127.4, 126.6, 125.0, 121.3, 121.1, 113.1, 56.2, 42.3, 19.4.

HRMS (ES+) Calcd for $C_{27}H_{24}Br_2NaO_3$ (M + Na^+): 578.9964, Found: 578.9971.

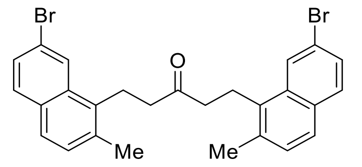

S3c

1,5-Bis(7-bromo-2-methylnaphthalen-1-yl)pentan-3-one (S3c) was prepared as a white solid from **6c** (777 mg, 1.5 mmol), the Crabtree's catalyst (12 mg, 0.015 mmol), and DCM (15 mL) according to the above procedure (eluent: hexanes/DCM = 2:1) in 69% yield (536 mg).

^{1}H NMR (400 MHz, $CDCl_3$) δ 8.07 (s, 2H), 7.63 (d, *J* = 8.7 Hz, 2H), 7.57 (d, *J* = 8.4 Hz, 2H), 7.50 – 7.42 (m, 2H), 7.28 (d, *J* = 8.4 Hz, 2H), 3.30 (t, *J* = 8.0 Hz, 4H), 2.66 (t, *J* = 8.0 Hz, 4H), 2.46 (s, 6H).

^{13}C NMR (101 MHz, $CDCl_3$) δ 209.0, 134.3, 133.1, 132.9, 130.8, 130.3, 129.6, 127.9, 126.3, 125.4, 120.5, 42.4, 22.3, 20.1.

HRMS (ES+) Calcd for $C_{27}H_{24}Br_2NaO$ (M + Na^+): 547.0066, Found: 547.0073.

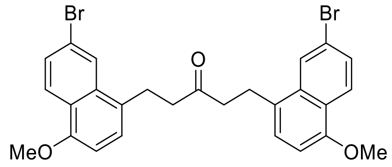

S3d

1,5-Bis(7-bromo-4-methoxynaphthalen-1-yl)pentan-3-one (S3d) was prepared as a white solid from **6d** (550 mg, 1.0 mmol), the Crabtree's catalyst (8.0 mg, 0.01 mmol), and DCM (10 mL) according to the above procedure (eluent: hexanes/DCM = 2:1) in 80% yield (438 mg).

^{1}H NMR (400 MHz, $CDCl_3$) δ 8.14 (d, *J* = 9.0 Hz, 2H), 8.02 (d, *J* = 2.0 Hz, 2H), 7.52 (dd, *J* = 9.0, 1.9 Hz, 2H), 7.17 (d, *J* = 7.9 Hz, 2H), 6.67 (d, *J* = 7.9 Hz, 2H), 3.94 (s, 6H), 3.19 (t, *J* = 7.6 Hz, 4H), 2.76 (t, *J* = 7.6 Hz, 4H).

^{13}C NMR (101 MHz, $CDCl_3$) δ 209.0, 154.2, 133.5, 128.0, 127.7, 127.0, 125.0, 124.7, 124.2, 121.1, 103.7, 55.3, 43.4, 25.9.

HRMS (ES+) Calcd for $C_{27}H_{24}Br_2NaO_3$ (M + Na^+): 578.9964, Found: 578.9971.

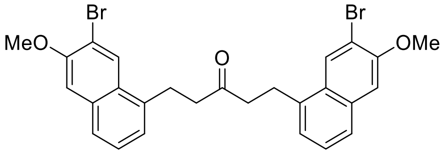

S3e

1,5-Bis(7-bromo-6-methoxynaphthalen-1-yl)pentan-3-one (S3e) was prepared as a white solid from **6e** (550 mg, 1.0 mmol), the Crabtree's catalyst (8.0 mg, 0.01 mmol), and DCM (10 mL) according to the above procedure (eluent: hexanes/DCM = 2:1) in 86% yield (475 mg).

^{1}H NMR (400 MHz, $CDCl_3$) δ 8.15 (s, 2H), 7.63 – 7.51 (m, 2H), 7.38 – 7.29 (m, 2H), 7.21 – 7.08 (m, 4H), 3.99 (s, 6H), 3.28 (t, *J* = 7.6 Hz, 4H), 2.81 (t, *J* = 7.6 Hz, 4H).

^{13}C NMR (101 MHz, $CDCl_3$) δ 208.8, 153.2, 136.1, 134.1, 128.2, 127.6, 126.6, 125.6, 124.6, 113.6, 107.5, 56.2, 43.6, 26.5.

HRMS (ES+) Calcd for $C_{27}H_{24}Br_2NaO_3$ (M + Na^+): 578.9964, Found: 578.9968.

3. Synthesis of the Unsymmetrical Ketones S3

Step 1: Synthesis of the Enones S5

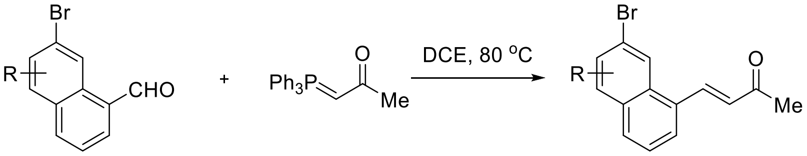

5 **S5**

A sealed tube charged with the aldehyde (1.0 equiv),

(acetylmethylene)triphenylphosphorane (2.0 equiv), and DCE was heated at 80 °C for 12 h before it was cooled to room temperature. The mixture was concentrated and the crude product was purified by silica gel flash chromatography (eluent: hexanes/DCM = 1:1) to afford the enone as a yellow solid.

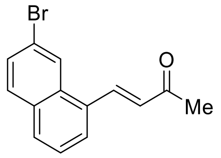

S5a

(*E*)-4-(7-Bromonaphthalen-1-yl)but-3-en-2-one (**S5a)** was prepared as a yellow solid from 7-bromo-1-naphthaldehyde **5a** (1.17 g, 5.0 mmol), (acetylmethylene)triphenylphosphorane (3.78 g, 10.0 mmol) and DCE (10 mL) according to the above procedure in 96% yield (1.31 g). This yellow solid was used directly for the next step.

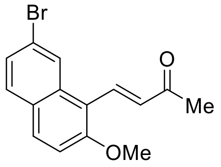

S5b

(*E*)-4-(7-Bromo-2-methoxynaphthalen-1-yl)but-3-en-2-one (S5b) was prepared as a yellow solid from 7-bromo-2-methoxy-1-naphthaldehyde **5b** (1.25 g, 5.0 mmol), (acetylmethylene)triphenylphosphorane (3.78 g, 10.0 mmol) and DCE (10 mL) according to the General procedure in 92% yield (1.32 g). This yellow solid was used directly for the next step.

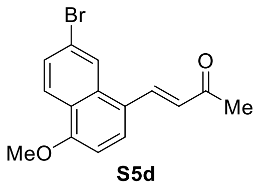

S5d

(*E*)-4-(7-Bromo-4-methoxynaphthalen-1-yl)but-3-en-2-one (S5d) was

prepared as a yellow solid from 7-bromo-2-methyl-1-naphthaldehyde **5d** (1.25 g, 5.0 mmol), (acetylmethylene)triphenylphosphorane (3.78 g, 10.0 mmol) and DCE (10 mL) according to the above procedure in 92% yield (1.32 g). This yellow solid was used directly for the next step.

Step 2: Aldol Condensation

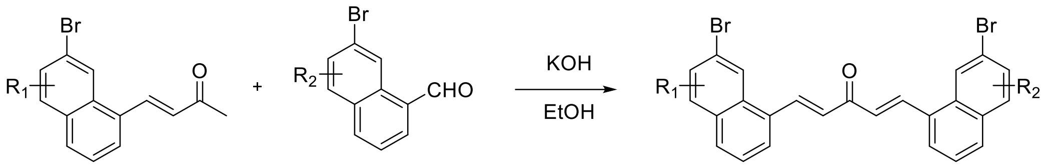

A mixture of the enone (1.0 equiv) and the aldehyde (1.0 equiv), and KOH (1.0 equiv) in EtOH was stirred at room temperature. Upon completion (~ 12 h), the mixture was filtered through glass frit, and the yellow filter cake was washed with water (20 mL × 3) followed by EtOH (20 mL × 3). This yellow solid was dried under vacuum to afford the unsymmetrical dienone. This yellow solid was used directly for the next step without further purification.

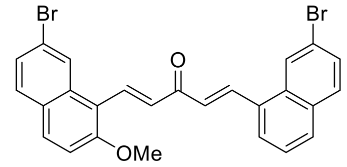

6f

(1*E*,4*E*)-1-(7-Bromo-2-methoxynaphthalen-1-yl)-5-(7-bromonaphthalen-1-yl)penta-1,4-dien-3-one (6f) was prepared as a yellow solid from (*E*)-4-(7-bromo-2-methoxynaphthalen-1-yl)but-3-en-2-one **S5b** (304 mg, 1.0 mmol), 7-bromo-1-naphthaldehyde **5a** (234 mg, 1.0 mmol), KOH (56.1 mg, 1.0 mmol) and absolute EtOH (5.0 mL) according to the above procedure in 82% yield (426 mg). This yellow solid was used directly for the next step without further purification.

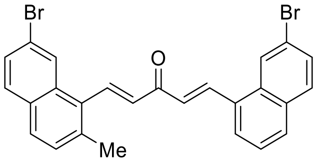

6g

(1*E*,4*E*)-1-(7-Bromo-2-methylnaphthalen-1-yl)-5-(7-bromonaphthalen-1-yl)penta-1,4-dien-3-one (**6g)** was prepared as a yellow solid from (*E*)-4-(7-bromonaphthalen-1-yl)but-3-en-2-one **S5a** (274 mg, 1.0 mmol), 7-bromo-2-methyl-1-naphthaldehyde **5c** (248 mg, 1.0 mmol), KOH (56.1 mg, 1.0 mmol) and absolute EtOH (5.0 mL) according to the above procedure in 70% yield (350 mg). This yellow solid was used directly for the next step without further purification.

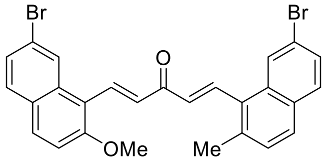

6h

(1*E*,4*E*)-1-(7-Bromo-2-methoxynaphthalen-1-yl)-5-(7-bromo-2-methylnaph thalen-1-yl)penta-1,4-dien-3-one (6h) was prepared as a yellow solid from (*E*)-4-(7-bromo-2-methoxynaphthalen-1-yl)but-3-en-2-one **S5b** (304 mg, 1.0 mmol), 7-bromo-2-methyl-1-naphthaldehyde **5c** (248 mg, 1.0 mmol), KOH (56.1 mg, 1.0 mmol) and absolute EtOH (5.0 mL) according to the above procedure in 93% yield (497 mg). This yellow solid was used directly for the next step without further purification.

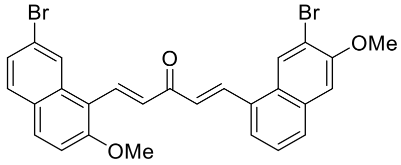

6i

(1*E*,4*E*)-1-(7-Bromo-2-methoxynaphthalen-1-yl)-5-(7-bromo-6-methoxy naphthalen-1-yl)penta-1,4-dien-3-one (6i) was prepared as a yellow solid from

(*E*)-4-(7-bromo-2-methoxynaphthalen-1-yl)but-3-en-2-one **S5b** (304 mg, 1.0 mmol), 7-bromo-6-methoxy-1-naphthaldehyde **5f** (264 mg, 1.0 mmol), KOH (56.1 mg, 1.0 mmol) and absolute EtOH (5.0 mL) according to the above procedure in 70% yield (488 mg). This yellow solid was used directly for the next step without further purification.

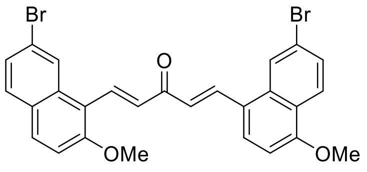

6j

(1*E*,4*E*)-1-(7-Bromo-2-methoxynaphthalen-1-yl)-5-(7-bromo-4-methoxynaph thalen-1-yl)penta-1,4-dien-3-one (6j) was prepared as a yellow solid from (*E*)-4-(7-bromo-2-methoxynaphthalen-1-yl)but-3-en-2-one **S5b** (456 mg, 1.5 mmol), 7-bromo-4-methoxy-1-naphthaldehyde **5d** (396 mg, 1.5 mmol), KOH (84.1 mg, 1.5 mmol) and absolute EtOH (7.5 mL) according to the above procedure in 84% yield (699 mg). This yellow solid was used directly for the next step without further purification.

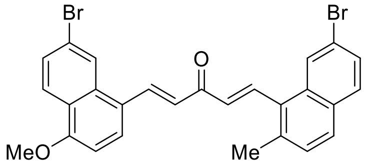

6k

(1*E*,4*E*)-1-(7-Bromo-2-methylnaphthalen-1-yl)-5-(7-bromo-4-methoxynaph thalen-1-yl)penta-1,4-dien-3-one (**6k)** was prepared as a yellow solid from (*E*)-4-(7-bromo-2-methylnaphthalen-1-yl)but-3-en-2-one **S5c** (304 mg, 1.0 mmol), 7-bromo-2-methyl-1-naphthaldehyde **5d** (248 mg, 1.0 mmol), KOH (56.1 mg, 1.0 mmol) and absolute EtOH (5.0 mL) according to the above procedure in 87% yield (465 mg). This yellow solid was used directly for the next step without further purification.

Step 3: Hydrogenation

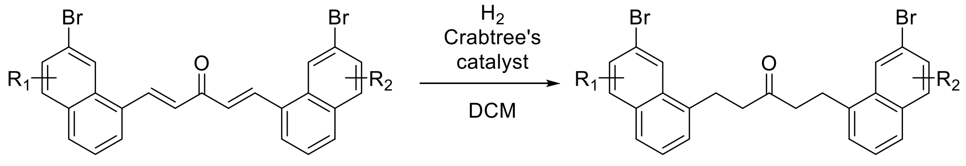

Next, the dienone was dissolved in DCM, to which was added the Crabtree's catalyst (1-2 mol%). The mixture was transferred into a Parr® autoclave, which was flushed with hydrogen gas three times and finally pressurized to 15 bar. The mixture was stirred at room temperature for 12 h, and the hydrogen gas was released carefully in a fume hood. The mixture was concentrated, and the crude product was purified by silica gel flash chromatography to afford the desired product**.**

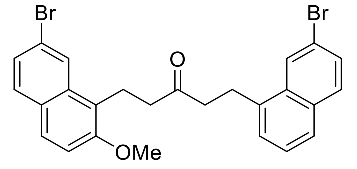

S3f

1-(7-Bromo-2-methoxynaphthalen-1-yl)-5-(7-bromonaphthalen-1-yl)pentan-3-one (S3f) was prepared as a white solid from **6f** (416 mg, 0.8 mmol), the Crabtree's catalyst (12.8 mg, 0.016 mmol), and DCM (8.0 mL) according to the above procedure (eluent: hexanes/DCM = 2:1) in 88% yield (371 mg).

^{1}H NMR (400 MHz, $CDCl_3$) δ 8.14 (d, *J* = 1.8 Hz, 1H), 8.04 (d, *J* = 1.8 Hz, 1H), 7.71 – 7.64 (m, 3H), 7.61 (d, *J* = 8.7 Hz, 1H), 7.56 – 7.51 (m, 1H), 7.44 – 7.36 (m, 2H), 7.35 – 7.32 (m, 1H), 7.22 (d, *J* = 9.0 Hz, 1H), 3.91 (s, 3H), 3.35 – 3.24 (m, 4H), 2.84 (t, *J* = 7.7 Hz, 2H), 2.67 (t, *J* = 8.2 Hz, 2H).

^{13}C NMR (101 MHz, $CDCl_3$) δ 209.3, 154.9, 136.3, 133.8, 132.7, 132.1, 130.4, 130.2, 128.9, 127.9, 127.3, 126.9, 126.7, 126.5, 126.0, 125.7, 124.9, 121.1, 121.0, 120.2, 113.0, 56.1, 43.1, 42.5, 26.5, 19.2.

HRMS (ES+) Calcd for $C_{26}H_{22}Br_2NaO_2$ (M + Na^+): 548.9858, Found: 548.9864.

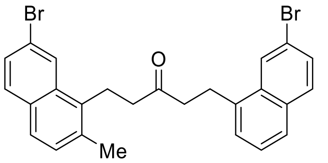

S3g

1-(7-Bromo-2-methylnaphthalen-1-yl)-5-(7-bromonaphthalen-1-yl)pentan-3-one (S3g) was prepared as a white solid from **6g** (352 mg, 0.7 mmol), the Crabtree's catalyst (5.6 mg, 0.007 mmol), and DCM (7.0 mL) according to the above procedure (eluent: hexanes/DCM = 2:1) in 53% yield (189 mg).

^{1}H NMR (400 MHz, $CDCl_3$) δ 8.16 – 8.02 (m, 2H), 7.73 – 7.61 (m, 3H), 7.61 – 7.51 (m, 2H), 7.51 – 7.44 (m, 1H), 7.44 – 7.37 (m, 1H), 7.37 – 7.31 (m, 1H), 7.31 – 7.20 (m, 1H), 3.38 – 3.21 (m, 4H), 2.83 (t, *J* = 7.6 Hz, 2H), 2.65 (t, *J* = 8.0 Hz, 2H), 2.43 (s, 3H).

^{13}C NMR (101 MHz, $CDCl_3$) δ 208.8, 136.1, 134.3, 133.1, 133.0, 132.7, 132.2, 130.9, 130.5, 130.3, 129.6, 128.9, 127.9, 127.0, 126.8, 126.3, 126.0, 125.7, 125.4, 120.5, 120.3, 43.3, 42.6, 26.5, 22.2, 20.1.

HRMS (ES+) Calcd for $C_{26}H_{22}Br_2NaO$ (M + Na$^+$): 532.9909, Found: 532.9910.

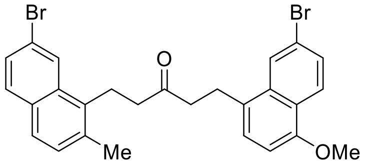

S3h

1-(7-Bromo-2-methylnaphthalen-1-yl)-5-(7-bromo-4-methoxynaphthalen-1-yl)pentan-3-one (S3h) was prepared as a white solid from **6h** (534 mg, 1.0 mmol), the Crabtree's catalyst (16.0 mg, 0.02 mmol), and DCM (10 mL) according to the above procedure (eluent: hexanes/DCM = 2:1) in 73% yield (390 mg).

^{1}H NMR (400 MHz, $CDCl_3$) δ 8.16 (d, *J* = 8.9 Hz, 1H), 8.04 (d, *J* = 8.3 Hz, 1H), 7.65 (d, *J* = 8.7 Hz, 1H), 7.60 – 7.45 (m, 3H), 7.28 – 7.22 (m, 2H), 6.73 (d, *J* = 7.9 Hz, 1H), 3.96 (s, 3H), 3.32 – 3.19 (m, 4H), 2.81 (t, *J* = 7.5 Hz, 2H), 2.65 (t, *J* = 8.1 Hz, 3H), 2.42 (s, 3H).

^{13}**C NMR** (101 MHz, $CDCl_3$) δ 209.2, 154.4, 134.4, 133.6, 133.2, 133.0, 130.9, 130.3, 129.6, 128.2, 128.0, 127.8, 127.2, 126.3, 125.6, 125.5, 124.8, 124.4, 121.3, 120.5, 103.8, 55.5, 43.5, 42.7, 26.3, 22.3, 20.1.

HRMS (ES+) Calcd for $C_{27}H_{24}Br_2NaO_2$ (M + Na^+): 563.0015, Found: 563.0019.

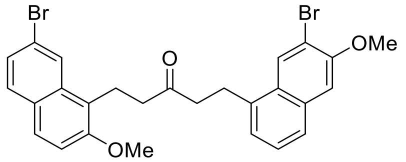

S3i

1-(7-Bromo-2-methoxynaphthalen-1-yl)-5-(7-bromo-6-methoxynaphthalen-1-yl)pentan-3-one (**S3i**) was prepared as a white solid from **6i** (554 mg, 0.83 mmol), the Crabtree's catalyst (12.8 mg, 0.016 mmol), and DCM (8.0 mL) according to the above procedure (eluent: hexanes/DCM = 2:1) in 74% yield (340 mg).

^{1}**H NMR** (400 MHz, $CDCl_3$) δ 8.19 (s, 1H), 8.03 (s, 1H), 7.67 (d, *J* = 9.0 Hz, 1H), 7.64 – 7.55 (m, 2H), 7.42 – 7.33 (m, 2H), 7.26 – 7.18 (m, 2H), 7.15 (s, 1H), 3.99 (s, 3H), 3.91 (s, 3H), 3.34 – 3.23 (m, 4H), 2.85 (t, *J* = 7.7 Hz, 2H), 2.67 (t, *J* = 8.0 Hz, 1H).

^{13}**C NMR** (101 MHz, $CDCl_3$) δ 209.4, 154.9, 153.2, 136.3, 134.1, 133.8, 130.2, 128.2, 127.9, 127.6, 127.3, 126.6, 126.5, 125.5, 124.9, 124.5, 121.1, 121.0, 113.5, 113.0, 107.4, 56.1, 56.1, 43.3, 42.5, 26.6, 19.2.

HRMS (ES+) Calcd for $C_{27}H_{24}Br_2NaO_3$ (M + Na^+): 578.9964, Found: 578.9973.

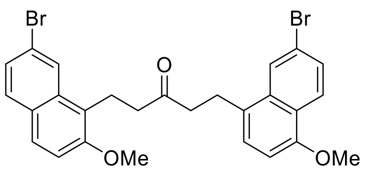

S3j

1-(7-Bromo-2-methoxynaphthalen-1-yl)-5-(7-bromo-4-methoxynaphthalen-1-yl)pentan-3-one (S3j) was prepared as a white solid from **6j** (550 mg, 1.0 mmol), the Crabtree's catalyst (16.0 mg, 0.02 mmol), and DCM (10 mL) according to the

above procedure (eluent: hexanes/DCM = 2:1) in 90% yield (497 mg).

^{1}H NMR (400 MHz, $CDCl_3$) δ 8.11 (d, *J* = 9.0 Hz, 1H), 8.07 – 7.97 (m, 2H), 7.61 (d, *J* = 9.0 Hz, 1H), 7.56 (d, *J* = 8.7 Hz, 1H), 7.52 – 7.44 (m, 1H), 7.41 – 7.33 (m, 1H), 7.21 – 7.09 (m, 2H), 6.66 (d, *J* = 7.9 Hz, 1H), 3.92 (s, 3H), 3.89 (s, 3H), 3.26 (t, *J* = 8.0 Hz, 2H), 3.18 (t, *J* = 7.7 Hz, 2H), 2.75 (t, *J* = 7.7 Hz, 2H), 2.63 (t, *J* = 8.0 Hz, 2H).

^{13}C NMR (101 MHz, $CDCl_3$) δ 209.3, 154.7, 154.0, 133.6, 133.4, 130.0, 127.75, 127.69, 127.62, 127.1, 126.7, 126.2, 125.3, 124.7, 124.5, 124.0, 120.9, 120.93, 120.86, 112.8, 103.5, 55.9, 55.2, 43.0, 42.3, 25.8, 19.0.

HRMS (ES+) Calcd for $C_{27}H_{24}Br_2NaO_3$ (M + Na^+): 578.9964, Found: 578.9973.

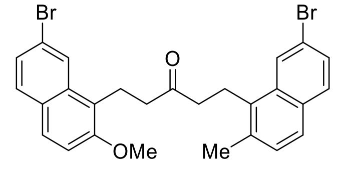

S3k

1-(7-Bromo-2-methoxynaphthalen-1-yl)-5-(7-bromo-2-methylnaphthalen-1-yl)pentan-3-one (S3k) was prepared as a white solid from **6k** (741 mg, 1.4 mmol), the Crabtree's catalyst (21.6 mg, 0.027 mmol), and DCM (8.0 mL) according to the above procedure (eluent: hexanes/DCM = 2:1) in 73% yield (542 mg).

^{1}H NMR (400 MHz, $CDCl_3$) δ 8.10 – 8.04 (m, 2H), 7.71 – 7.54 (m, 1H), 7.49 – 7.45 (m, 1H), 7.40 – 7.37 (m, 1H), 7.29 (d, *J* = 8.4 Hz, 1H), 7.24 (d, *J* = 9.1 Hz, 1H), 3.93 (s, 3H), 3.35 – 3.26 (m, 4H), 2.73 – 2.64 (m, 4H), 2.46 (s, 3H).

^{13}C NMR (101 MHz, $CDCl_3$) δ 209.5, 154.9, 134.3, 133.8, 133.3, 133.0, 130.8, 130.2, 130.2, 129.6, 127.9, 127.9, 127.3, 126.5, 126.2, 125.4, 124.9, 121.1, 120.9, 120.4, 113.0, 56.1, 42.4, 42.2, 22.3, 20.1, 19.3.

HRMS (ES+) Calcd for $C_{27}H_{24}Br_2NaO_2$ (M + Na^+): 563.0015, Found: 563.0018.

4. Synthesis of the Diamines 3

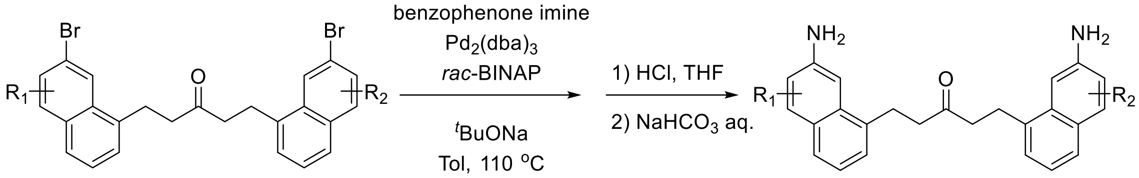

S3 **3**

At room temperature, a 100-mL round-bottom flask equipped with a stir bar was charged with the ketone (1.0 equiv), benzophenone imine (2.5 equiv), $Pd_2(dba)_3$ (2.5 mol%), *rac*-BINAP (6 mol%), ^{t}BuONa (3.0 equiv), and toluene. The flask was evacuated and refilled with N_2 for 5 times. Then, the mixture was stirred under N_2 at 110 °C for 24 h. After cooling to room temperature, the mixture was evaporated and purified by silica gel flash chromatography (eluent: hexanes/EtOAc = 10:1) to afford the desired product as a yellow foam. The yellow foam was dissolved in THF (50 mL) and treated with an aqueous solution of HCl (50 mL, 1.0 M). The mixture was stirred vigorously at room temperature. Upon completion (~ 1 h), the mixture was diluted with EtOAc (50 mL) and carefully poured into a saturated aqueous solution of $NaHCO_3$ at 0 °C. The mixture was extracted with EtOAc (30 mL × 3), washed with brine, dried over Na_2SO_4, and filtered. The filtrate was concentrated and purified by silica gel column chromatography to afford the desired product.

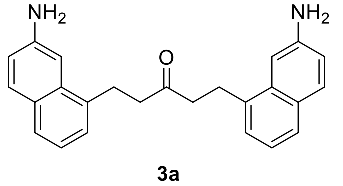

3a

1,5-Bis(7-aminonaphthalen-1-yl)pentan-3-one (3a) was prepared as a yellow foam from **S3a** (4.94 g, 10.0 mmol), benzophenone imine (4.53 g, 25.0 mmol), $Pd_2(dba)_3$ (229 mg, 0.25 mol), *rac*-BINAP (376 mg, 0.6 mmol), ^{t}BuONa (2.88 g, 30 mmol), and toluene (50 mL) according to the above procedure (eluent: DCM/EtOAc = 5:1) in 87% yield (3.20 g).

^{1}**H NMR** (400 MHz, $CDCl_3$) δ 7.66 (d, *J* = 8.7 Hz, 2H), 7.58 (d, *J* = 8.0 Hz, 2H), 7.23 – 7.18 (m, 2H), 7.18 – 7.11 (m, 2H), 7.05 (s, 1H), 6.92 (d, *J* = 8.7 Hz, 1H), 3.74 (s, 4H), 3.24 (t, *J* = 7.8 Hz, 4H), 2.80 (t, *J* = 7.8 Hz, 4H).

^{13}**C NMR** (101 MHz, $CDCl_3$) δ 210.1, 144.4, 134.5, 132.9, 130.2, 128.1, 126.7, 126.3, 122.0, 117.7, 104.5, 43.4, 26.9.

HRMS (ES+) Calcd for $C_{25}H_{24}N_2NaO$ (M + Na^+): 391.1781, Found: 391.1782.

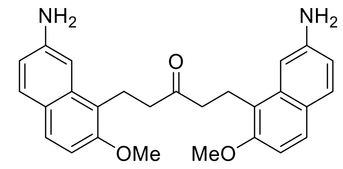

3b

1,5-Bis(7-amino-2-methoxynaphthalen-1-yl)pentan-3-one (3b) was prepared as a yellow foam from **S3b** (554 mg, 1.0 mmol), benzophenone imine (467 mg, 2.5 mmol), $Pd_2(dba)_3$ (45.7 mg, 0.05 mol), *rac*-BINAP (74.6 mg, 0.12 mmol), ^{t}BuONa (288 mg, 3.0 mmol), and toluene (10 mL) according to the above procedure (eluent: DCM/EtOAc = 5:1) in 53% yield (227 mg).

^{1}**H NMR** (400 MHz, acetone-d_6) δ 7.54 (dd, *J* = 8.8, 3.1 Hz, 4H), 7.04 (d, *J* = 2.1 Hz, 2H), 6.99 (d, *J* = 8.9 Hz, 2H), 6.86 (dd, *J* = 8.7, 2.1 Hz, 2H), 4.92 (s, 4H), 3.87 (s, 7H), 3.21 (t, *J* = 8.2 Hz, 4H), 2.69 (t, *J* = 8.2 Hz, 4H).

^{13}**C NMR** (101 MHz, acetone-d_6) δ 210.6, 155.6, 147.8, 135.5, 130.4, 128.4, 127.7, 124.1, 119.8, 116.7, 109.4, 102.9, 56.5, 42.7, 20.2.

HRMS (ES+) Calcd for $C_{27}H_{28}N_2NaO_3$ (M + Na^+): 451.1992, Found: 451.1996.

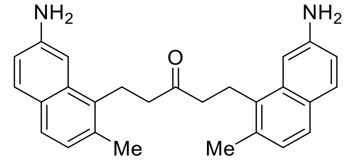

3c

1,5-Bis(7-amino-2-methylnaphthalen-1-yl)pentan-3-one (3c) was prepared as a yellow foam from **S3c** (518 mg, 1.0 mmol), benzophenone imine (467 mg, 2.5 mmol), $Pd_2(dba)_3$ (45.7 mg, 0.05 mol), *rac*-BINAP (74.6 mg, 0.12 mmol), ^{t}BuONa

(288 mg, 3.0 mmol), and toluene (10 mL) according to the above procedure (eluent: DCM/EtOAc = 5:1) in 75% yield (296 mg).

^{1}H NMR (400 MHz, acetone-*d*$_6$) δ 7.55 (d, *J* = 8.7 Hz, 2H), 7.43 (d, *J* = 8.3 Hz, 2H), 7.10 (s, 2H), 6.98 – 6.90 (m, 4H), 4.88 (s, 4H), 3.24 – 3.17 (m, 4H), 2.76 – 2.67 (m, 4H), 2.39 (s, 6H).

^{13}C NMR (101 MHz, acetone-*d*$_6$) δ 210.1, 147.5, 134.6, 133.6, 132.3, 130.4, 127.3, 126.9, 125.5, 117.8, 103.8, 42.6, 23.4, 20.2.

HRMS (ES+) Calcd for $C_{27}H_{28}N_2NaO$ (M + Na$^+$): 419.2094, Found: 419.2099.

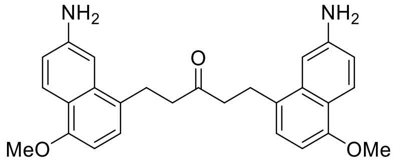

3d

1,5-Bis(7-amino-4-methoxynaphthalen-1-yl)pentan-3-one (3d) was prepared as a yellow foam from **S3d** (554 mg, 1.0 mmol), benzophenone imine (467 mg, 2.5 mmol), $Pd_2(dba)_3$ (45.7 mg, 0.05 mol), *rac*-BINAP (74.6 mg, 0.12 mmol), ^{t}BuONa (288 mg, 3.0 mmol), and toluene (10 mL) according to the above procedure (eluent: DCM/EtOAc = 5:1) in 67% yield (286 mg).

^{1}H NMR (400 MHz, methanol-*d*$_4$) δ 7.99 (d, *J* = 8.9 Hz, 2H), 7.02 – 6.97 (m, 2H), 6.97 – 6.87 (m, 4H), 6.41 (d, *J* = 7.8 Hz, 2H), 3.85 (s, 6H), 3.02 (t, *J* = 7.6 Hz, 4H), 2.68 (t, *J* = 7.6 Hz, 4H).

^{13}C NMR (101 MHz, methanol-*d*$_4$) δ 213.5, 155.8, 147.5, 135.4, 127.8, 127.2, 124.7, 120.9, 118.0, 106.0, 101.1, 55.7, 44.4, 27.7.

HRMS (ES+) Calcd for $C_{27}H_{28}N_2NaO_3$ (M + Na$^+$): 451.1992, Found: 451.1999.

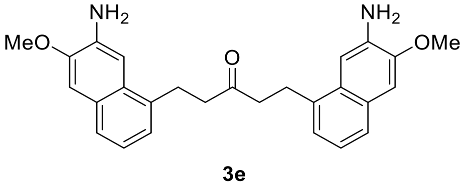

3e

1,5-Bis(7-amino-6-methoxynaphthalen-1-yl)pentan-3-one (3e) was prepared

as a yellow foam from **S3e** (554 mg, 1.0 mmol), benzophenone imine (467 mg, 2.5 mmol), $Pd_2(dba)_3$ (45.7 mg, 0.05 mol), *rac*-BINAP (74.6 mg, 0.12 mmol), ^{t}BuONa (288 mg, 3.0 mmol), and toluene (10 mL) according to the above procedure (eluent: DCM/EtOAc = 5:1) in 57% yield (245 mg).

^{1}H NMR (400 MHz, acetone-d_6) δ 7.55 – 7.42 (m, 2H), 7.19 – 7.13 (m, 4H), 7.11 – 6.95 (m, 4H), 4.83 (s, 4H), 3.95 (s, 6H), 3.15 (t, *J* = 7.2 Hz, 4H), 2.84 (t, *J* = 7.2 Hz, 4H).

^{13}C NMR (101 MHz, acetone-d_6) δ 209.6, 149.1, 139.3, 135.0, 129.3, 129.2, 126.2, 124.5, 122.4, 106.8, 104.3, 55.8, 43.8, 27.9.

HRMS (ES+) Calcd for $C_{27}H_{28}N_2NaO_3$ (M + Na$^+$): 451.1992, Found: 451.1996.

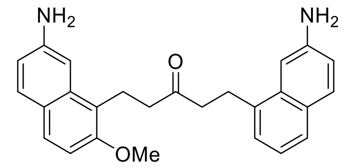

3f

1-(7-Amino-2-methoxynaphthalen-1-yl)-5-(7-aminonaphthalen-1-yl)pentan-3-one (3f) was prepared as a yellow foam from **S3f** (524 mg, 1.0 mmol), benzophenone imine (467 mg, 2.5 mmol), $Pd_2(dba)_3$ (45.7 mg, 0.05 mol), *rac*-BINAP (74.6 mg, 0.12 mmol), ^{t}BuONa (288 mg, 3.0 mmol), and toluene (10 mL) according to the above procedure (eluent: DCM/EtOAc = 5:1) in 54% yield (214 mg).

^{1}H NMR (400 MHz, acetone-d_6) δ 7.61 (d, *J* = 8.7 Hz, 1H), 7.58 – 7.48 (m, 3H), 7.19 (d, *J* = 6.9 Hz, 1H), 7.14 (d, *J* = 2.2 Hz, 1H), 7.08 – 6.96 (m, 4H), 6.85 (dd, *J* = 8.7, 2.2 Hz, 1H), 4.96 (s, 2H), 4.91 (s, 2H), 3.87 (s, 3H), 3.23 – 3.11 (m, 4H), 2.91 – 2.85 (m, 2H), 2.72 – 2.58 (m, 2H).

^{13}C NMR (101 MHz, acetone-d_6) δ 210.1, 155.6, 147.8, 147.4, 135.5, 135.4, 134.4, 130.5, 130.4, 128.43, 128.41, 127.3, 126.7, 124.1, 121.9, 119.8, 118.7, 116.7, 109.4, 104.0, 102.9, 56.5, 43.4, 42.9, 27.8, 20.2.

HRMS (ES+) Calcd for $C_{26}H_{26}N_2NaO_2$ (M + Na$^+$): 421.1886, Found: 421.1891.

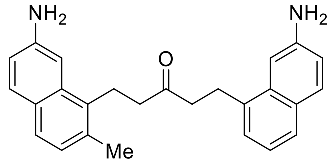

3g

1-(7-Amino-2-methylnaphthalen-1-yl)-5-(7-aminonaphthalen-1-yl)pentan-3-one (3g) was prepared as a yellow foam from **S3g** (254 mg, 0.5 mmol), benzophenone imine (234 mg, 1.3 mmol), $Pd_2(dba)_3$ (22.9 mg, 0.025 mol), *rac*-BINAP (37.3 mg, 0.06 mmol), ^{t}BuONa (115 mg, 1.2 mmol), and toluene (5.0 mL) according to the above procedure (eluent: DCM/EtOAc = 5:1) in 70% yield (134 mg).

^{1}H NMR (400 MHz, $CDCl_3$) δ 7.71 – 7.54 (m, 3H), 7.49 (d, *J* = 8.3 Hz, 1H), 7.29 – 7.12 (m, 2H), 7.12 – 6.98 (m, 3H), 6.97 – 6.79 (m, 2H), 3.88 (s, 4H), 3.34 – 3.14 (m, 4H), 2.83 (t, *J* = 7.8 Hz, 2H), 2.66 (t, *J* = 8.1 Hz, 2H).

^{13}C NMR (101 MHz, $CDCl_3$) δ 210.4, 144.5, 144.4, 134.5, 133.4, 133.1, 132.9, 131.5, 130.2, 130.0, 128.1, 126.9, 126.7, 126.4, 126.2, 125.7, 122.0, 117.8, 116.8, 104.4, 104.3, 43.4, 42.4, 27.0, 22.5, 20.1.

HRMS (ES+) Calcd for $C_{26}H_{26}N_2NaO$ (M + Na$^+$): 405.1932, Found: 405.1939.

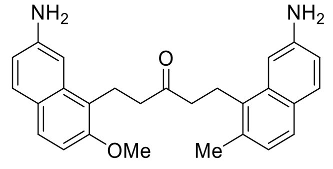

3h

1-(7-Amino-2-methoxynaphthalen-1-yl)-5-(7-amino-2-methylnaphthalen-1-yl)pentan-3-one (3h) was prepared as a yellow foam from **S3h** (542 mg, 1.0 mmol), benzophenone imine (467 mg, 2.5 mmol), $Pd_2(dba)_3$ (45.7 mg, 0.05 mol), *rac*-BINAP (74.6 mg, 0.12 mmol), ^{t}BuONa (288 mg, 3.0 mmol), and toluene (10 mL) according to the above procedure (eluent: DCM/EtOAc = 5:1) in 70% yield (286 mg).

^{1}H NMR (400 MHz, $CDCl_3$) δ 7.65 – 7.55 (m, 3H), 7.52 – 7.45 (m, 1H), 7.10 – 6.96 (m, 4H), 6.91 – 6.85 (m, 1H), 6.84 – 6.76 (m, 1H), 3.90 (s, 7H), 3.33 – 3.19 (m, 4H), 2.77 – 2.63 (m, 4H), 2.42 (s, 3H).

^{13}C NMR (101 MHz, $CDCl_3$) δ 211.2, 154.9, 144.9, 144.5, 134.1, 133.4, 133.2, 131.7, 130.0, 127.7, 126.9, 126.1, 125.7, 123.6, 119.4, 116.8, 115.7, 109.4, 104.4, 103.6, 56.1, 42.5, 42.3, 22.6, 20.1, 19.6.

HRMS (ES+) Calcd for $C_{27}H_{28}N_2NaO_2$ (M + Na^+): 451.1992, Found: 451.1996.

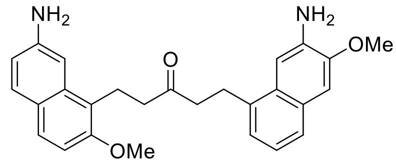

3i

1-(7-Amino-2-methoxynaphthalen-1-yl)-5-(7-amino-6-methoxynaphthalen-1-yl)pentan-3-one (3i) was prepared as a yellow foam from **S3i**(554 mg, 1.0 mmol), benzophenone imine (467 mg, 2.5 mmol), $Pd_2(dba)_3$ (45.7 mg, 0.05 mol), *rac*-BINAP (74.6 mg, 0.12 mmol), ^{t}BuONa (288 mg, 3.0 mmol), and toluene (10 mL) according to the above procedure (eluent: DCM/EtOAc = 5:1) in 46% yield (195 mg).

^{1}H NMR (400 MHz, acetone-d_6) δ 7.54 (d, J = 8.8 Hz, 2H), 7.52 – 7.46 (m, 1H), 7.17 (d, J = 13.4 Hz, 2H), 7.11 – 7.02 (m, 2H), 7.01 – 6.96 (m, 2H), 6.85 (dd, J = 8.7, 2.1 Hz, 1H), 4.90 (s, 2H), 4.85 (s, 2H), 3.95 (s, 3H), 3.87 (s, 3H), 3.21 – 3.11 (m, 4H), 2.85 (t, J = 7.4 Hz, 1H), 2.66 (t, J = 8.2 Hz, 2H).

^{13}C NMR (101 MHz, acetone-d_6) δ 210.0, 155.6, 149.1, 147.8, 139.3, 135.5, 135.1, 130.4, 129.3, 129.2, 128.4, 126.2, 124.6, 124.1, 122.5, 119.8, 116.7, 109.4, 106.8, 104.4, 102.9, 56.5, 55.8, 43.6, 42.9, 28.0, 20.2.

HRMS (ES+) Calcd for $C_{27}H_{29}N_2O_3$ (M + H^+): 429.2173, Found: 429.2173.

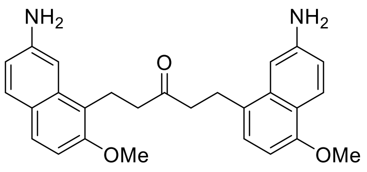

3j

1-(7-Amino-2-methoxynaphthalen-1-yl)-5-(7-amino-4-methoxynaphthalen-1-yl)pentan-3-one (3j) was prepared as a yellow foam from **S3j** (554 mg, 1.0 mmol), benzophenone imine (467 mg, 2.5 mmol), $Pd_2(dba)_3$ (45.7 mg, 0.05 mol), *rac*-BINAP (74.6 mg, 0.12 mmol), ^{t}BuONa (288 mg, 3.0 mmol), and toluene (10 mL) according to the above procedure (eluent: DCM/EtOAc = 5:1) in 62% yield (267 mg).

^{1}H NMR (400 MHz, methanol-d_4) δ 7.98 (d, *J* = 8.9 Hz, 1H), 7.52 – 7.44 (m, 2H), 7.03 (d, *J* = 2.2 Hz, 1H), 6.98 – 6.87 (m, 4H), 6.81 (dd, *J* = 8.7, 2.1 Hz, 1H), 6.42 (d, *J* = 7.8 Hz, 1H), 3.84 (s, 3H), 3.78 (s, 3H), 3.11 (t, *J* = 7.9 Hz, 2H), 3.04 (t, *J* = 7.6 Hz, 2H), 2.70 (t, *J* = 7.6 Hz, 2H), 2.55 (t, *J* = 7.9 Hz, 2H).

^{13}C NMR (101 MHz, methanol-d_4) δ 214.0, 156.1, 155.9, 147.3, 135.6, 135.4, 130.8, 128.8, 127.9, 127.2, 125.2, 124.8, 120.9, 120.3, 118.0, 117.2, 110.1, 106.0, 104.7, 101.1, 56.6, 55.7, 44.2, 43.6, 27.9, 20.5.

HRMS (ES+) Calcd for $C_{27}H_{28}N_2NaO_3$ (M + Na^+): 451.1992, Found: 451.1999.

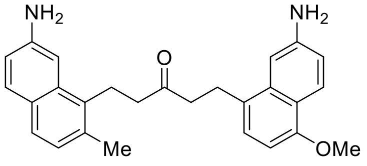

3k

1-(7-Amino-2-methylnaphthalen-1-yl)-5-(7-amino-4-methoxynaphthalen-1-yl)pentan-3-one (3k) was prepared as a yellow foam from **S3k** (538 mg, 1.0 mmol), benzophenone imine (467 mg, 2.5 mmol), $Pd_2(dba)_3$ (45.7 mg, 0.05 mol), *rac*-BINAP (74.6 mg, 0.12 mmol), ^{t}BuONa (288 mg, 3.0 mmol), and toluene (10 mL) according to the above procedure (eluent: DCM/EtOAc = 5:1) in 74% yield (303 mg).

^{1}**H NMR** (400 MHz, $CDCl_3$) δ 8.13 (d, *J* = 8.9 Hz, 1H), 7.61 (d, *J* = 8.6 Hz, 1H), 7.48 (d, *J* = 8.3 Hz, 1H), 7.13 (d, *J* = 7.8 Hz, 1H), 7.08 – 6.97 (m, 3H), 6.91 (dd, *J* = 8.9, 2.3 Hz, 1H), 6.86 (dd, *J* = 8.6, 2.2 Hz, 1H), 6.51 (d, *J* = 7.8 Hz, 1H), 3.94 (s, 7H), 3.27 – 3.15 (m, 4H), 2.81 (t, *J* = 7.7 Hz, 2H), 2.63 (t, *J* = 7.6 Hz, 2H).

^{13}**C NMR** (101 MHz, $CDCl_3$) δ 210.7, 154.5, 144.9, 144.5, 133.9, 133.4, 133.2, 131.6, 130.0, 126.9, 126.6, 126.4, 126.2, 125.7, 124.2, 119.8, 116.8, 116.7, 104.7, 104.3, 100.4, 55.3, 43.5, 42.5, 26.8, 22.5, 20.1.

HRMS (ES+) Calcd for $C_{27}H_{28}N_2NaO_2$ ($M + Na^+$): 435.2043, Found: 435.2049.

III. Synthesis of SPHENAMs

1. General Procedure A: Synthesis of the SPHENAMs 1

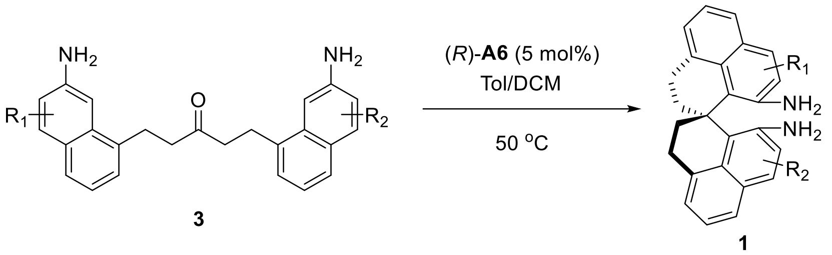

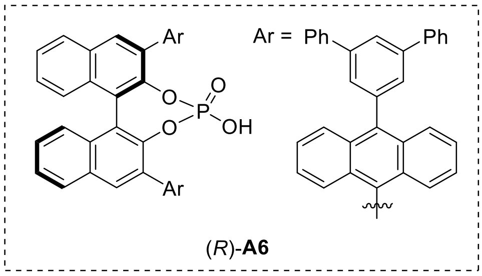

At room temperature, to a solution of the linear diamine substrate **3** (0.3 mmol) in toluene (3.0 mL) and DCM (3.0 mL) was added (*R*)-**A6** (17.3 mg, 15 μmol). The mixture was stirred at 50 °C for 24 h. The reaction progress was monitored by thin layer chromatography. Upon completion, the mixture was cooled to room temperature, and directly subjected to silica gel flash chromatography (eluent: hexanes/DCM = 1:1) to afford the product.

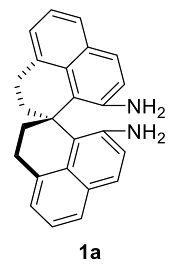

1a

(*S*)-2,2',3,3'-Tetrahydro-1,1'-spirobi[phenalene]-9,9'-diamine (1a) was prepared as a white solid from **3a** (110 mg, 0.3 mmol), (*R*)-**A6** (17.3 mg, 15 μmol), DCM (3.0 mL), and toluene (3.0 mL) according to the General Procedure A (eluent: hexanes/DCM = 1:1) in 91% yield (95.6 mg, 90% ee).

$[\alpha]_D^{20}$: -177.5 (c = 1.0, CH_2Cl_2). HPLC analysis of the product: Daicel

CHIRALPAK AD-H column; 20% *i*-PrOH in hexanes; 1.0 mL/min; retention times: 14.6 min (minor), 15.7 min (major).

^{1}H NMR (400 MHz, $CDCl_3$) δ 7.64 – 7.56 (m, 4H), 7.30 – 7.11 (m, 4H), 6.79 (d, *J* = 8.7 Hz, 2H), 3.53 (s, 4H), 3.41 – 3.25 (m, 2H), 3.12 – 2.97 (m, 2H), 2.55 – 2.29 (m, 4H).

^{13}C NMR (101 MHz, $CDCl_3$) δ 140.8, 133.2, 130.4, 128.9, 128.3, 126.5, 125.2, 121.9, 119.5, 117.6, 40.2, 27.9, 26.4.

HRMS (ES+) Calcd for $C_{25}H_{23}N_2$ (M + H$^+$): 351.1856, Found: 351.1863.

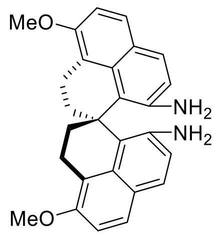

1b

(*S*)-4,4'-Dimethoxy-2,2',3,3'-tetrahydro-1,1'-spirobi[phenalene]-9,9'-diamine (1b) was prepared as a white solid from **3b** (128 mg, 0.3 mmol), (*R*)-**A6** (17.3 mg, 15 μmol), DCM (3.0 mL), and toluene (3.0 mL) according to the General Procedure A (eluent: hexanes/DCM = 1:1) in 75% yield (92.1 mg, 90% ee).

$[\alpha]_D^{20}$: -463.2 (*c* = 1.0, CH_2Cl_2). HPLC analysis of the product: Daicel CHIRALCEL OD-H column; 20% *i*-PrOH in hexanes; 1.0 mL/min; retention times: 12.6 min (minor), 31.2 min (major).

^{1}H NMR (400 MHz, $CDCl_3$) δ 7.61 (d, *J* = 9.2 Hz, 2H), 7.52 (d, *J* = 8.7 Hz, 2H), 7.06 (d, *J* = 8.8 Hz, 2H), 6.65 (d, *J* = 8.7 Hz, 2H), 3.96 (s, 6H), 3.64 (s, 4H), 3.40 – 3.25 (m, 2H), 2.96 – 2.80 (m, 2H), 2.40 – 2.20 (m, 4H).

^{13}C NMR (101 MHz, $CDCl_3$) δ 153.7, 141.2, 131.4, 128.2, 127.1, 124.4, 118.6, 117.5, 116.4, 108.8, 56.2, 39.1, 27.2, 18.7.

HRMS (ES+) Calcd for $C_{27}H_{27}N_2O_2$ (M + H$^+$): 411.2067, Found: 411.2070.

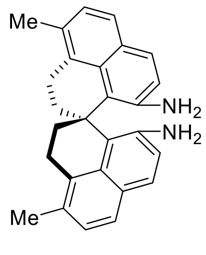

1c

(*S*)-4,4'-Dimethyl-2,2',3,3'-tetrahydro-1,1'-spirobi[phenalene]-9,9'-diamine (1c) was prepared as a white solid from **3c** (120 mg, 0.3 mmol), (*R*)-**A6** (17.3 mg, 15 μmol), DCM (3.0 mL), and toluene (3.0 mL) according to the General Procedure A (eluent: hexanes/DCM = 1:1) in 95% yield (108 mg, 88% ee).

$[\alpha]_D^{20}$: -431.4 (*c* = 1.0, CH_2Cl_2). HPLC analysis of the product: Daicel CHIRALPAK AD-H column; 20% *i*-PrOH in hexanes; 1.0 mL/min; retention times: 18.4 min (minor), 27.5 min (major).

^{1}H NMR (400 MHz, $CDCl_3$) δ 7.60 – 7.50 (m, 4H), 7.19 – 7.12 (m, 2H), 6.74 (d, *J* = 8.7 Hz, 2H), 3.61 (s, 4H), 3.20 – 2.98 (m, 4H), 2.48 (s, 6H), 2.45 – 2.35 (m, 4H).

^{13}C NMR (101 MHz, $CDCl_3$) δ 141.0, 132.4, 130.5, 130.3, 128.2, 127.7, 126.0, 125.10, 118.5, 117.3, 39.2, 27.4, 22.8, 20.3.

HRMS (ES+) Calcd for $C_{27}H_{27}N_2$ (M + H$^+$): 379.2169, Found: 379.2171.

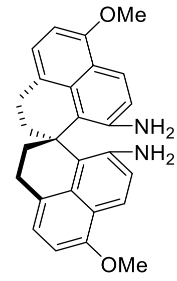

1d

(*S*)-6,6'-Dimethoxy-2,2',3,3'-tetrahydro-1,1'-spirobi[phenalene]-9,9'-diamine (1d) was prepared as a white solid from **3d** (128 mg, 0.3 mmol), (*R*)-**A6** (17.3 mg, 15 μmol), DCM (3.0 mL), and toluene (3.0 mL) according to the General Procedure A (eluent: hexanes/DCM = 1:1) in 95% yield (118 mg, 90% ee).

$[\alpha]_D^{20}$: -148.5 (*c* = 1.0, CH_2Cl_2). HPLC analysis of the product: Daicel CHIRALPAK AD-H column; 30% *i*-PrOH in hexanes; 1.0 mL/min; retention

times: 20.2 min (major), 31.0 min (minor).

^{1}H NMR (400 MHz, $CDCl_3$) δ 8.10 (d, *J* = 9.0 Hz, 2H), 7.18 (d, *J* = 7.8 Hz, 2H), 6.77 (d, *J* = 9.0 Hz, 1H), 6.64 (d, *J* = 7.8 Hz, 2H), 4.01 (s, 5H), 3.44 (s, 4H), 3.33 – 3.20 (m, 2H), 3.04 – 2.88 (m, 2H), 2.52 – 2.33 (m, 4H).

^{13}C NMR (101 MHz, $CDCl_3$) δ 154.2, 141.4, 131.2, 125.3, 124.6, 122.0, 120.2, 118.5, 117.8, 100.5, 55.4, 40.0, 28.1, 25.8.

HRMS (ES+) Calcd for $C_{27}H_{27}N_2O_2$ (M + H$^+$): 411.2067, Found: 411.2068.

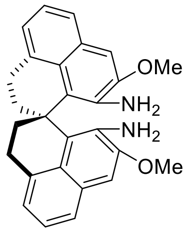

1e

(*S*)-8,8'-Dimethoxy-2,2',3,3'-tetrahydro-1,1'-spirobi[phenalene]-9,9'-diamine (1e) was prepared as a white solid from **1e** (128 mg, 0.3 mmol), (*R*)-**A6** (17.3 mg, 15 μmol), DCM (3.0 mL), and toluene (3.0 mL) according to the General Procedure A (eluent: hexanes/DCM = 1:1) in 88% yield (109 mg, 95% ee).

$[\alpha]_D^{20}$: -224.4 (*c* = 0.5, CH_2Cl_2). HPLC analysis of the product: Daicel CHIRALPAK AD-H column; 30% *i*-PrOH in hexanes; 1.0 mL/min; retention times: 6.2 min (minor), 9.5 min (major).

^{1}H NMR (400 MHz, $CDCl_3$) δ 7.70 – 7.56 (m, 2H), 7.32 – 7.26 (m, 2H), 7.25 – 7.18 (m, 2H), 7.09 (s, 2H), 3.97 (s, 4H), 3.94 (s, 6H), 3.48 – 3.24 (m, 2H), 3.18 – 2.95 (m, 2H), 2.67 – 2.51 (m, 2H), 2.51 – 2.31 (m, 2H).

^{13}C NMR (101 MHz, $CDCl_3$) δ 147.3, 133.5, 133.0, 128.6, 125.4, 125.1, 122.9, 122.2, 117.2, 104.2, 55.2, 40.7, 27.5, 26.3.

HRMS (ES+) Calcd for $C_{27}H_{27}N_2O_2$ (M + H$^+$): 411.2067, Found: 411.2072.

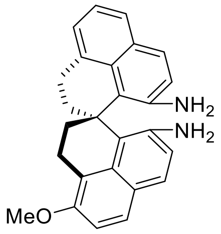

1f

(*S*)-4-Methoxy-2,2',3,3'-tetrahydro-1,1'-spirobi[phenalene]-9,9'-diamine (1f) was prepared as a white solid from **3f** (120 mg, 0.3 mmol), (*R*)-**A6** (17.3 mg, 15 μmol), DCM (3.0 mL), and toluene (3.0 mL) according to the General Procedure A (eluent: hexanes/DCM = 1:1) in 88% yield (101 mg, 91% ee).

$[\alpha]_D^{20}$: -291.2 (*c* = 0.6, CH_2Cl_2). HPLC analysis of the product: Daicel CHIRALPAK AD-H column; 30% *i*-PrOH in hexanes; 1.0 mL/min; retention times: 17.8 min (minor), 22.7 min (major).

^{1}H NMR (400 MHz, $CDCl_3$) δ 7.66 – 7.57 (m, 3H), 7.54 (d, *J* = 8.7 Hz, 1H), 7.30 – 7.17 (m, 2H), 7.09 (d, *J* = 8.8 Hz, 1H), 6.79 (d, *J* = 8.7 Hz, 1H), 6.64 (d, *J* = 8.6 Hz, 1H), 3.98 (s, 3H), 3.55 – 3.26 (m, 6H), 3.09 – 2.98 (m, 1H), 2.97 – 2.83 (m, 1H), 2.45 – 2.30 (m, 4H).

^{13}C NMR (101 MHz, $CDCl_3$) δ 153.7, 141.1, 140.9, 133.3, 131.3, 130.3, 128.9, 128.2, 128.1, 127.1, 126.4, 125.1, 124.2, 121.8, 119.4, 118.3, 117.8, 117.4, 116.0, 108.7, 56.1, 39.6, 27.9, 27.1, 26.3, 18.7.

HRMS (ES+) Calcd for $C_{26}H_{25}N_2O$ (M + H$^+$): 381.1961, Found: 381.1967.

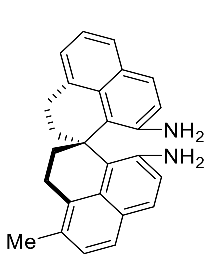

1g

(*S*)-4-Methyl-2,2',3,3'-tetrahydro-1,1'-spirobi[phenalene]-9,9'-diamine (1g) was prepared as a white solid from **3g** (110 mg, 0.3 mmol), (*R*)-**A6** (17.3 mg, 15 μmol), DCM (3.0 mL), and toluene (3.0 mL) according to the General Procedure

A (eluent: hexanes/DCM = 1:1) in 91% yield (99.6 mg, 92% ee).

$[\alpha]_D^{20}$: -314.2 (c = 0.5, CH_2Cl_2). HPLC analysis of the product: Daicel CHIRALPAK AD-3 column; 20% *i*-PrOH in hexanes; 1.0 mL/min; retention times: 18.8 min (minor), 23.4 min (major).

^{1}H NMR (400 MHz, $CDCl_3$) δ 7.64 – 7.58 (m, 2H), 7.58 – 7.49 (m, 2H), 7.26 – 7.17 (m, 2H), 7.15 (d, *J* = 8.2 Hz, 1H), 6.80 (d, *J* = 8.7 Hz, 1H), 6.72 (d, *J* = 8.7 Hz, 1H), 3.58 (s, 5H), 3.40 – 3.29 (m, 1H), 3.17 – 2.95 (m, 4H), 2.47 – 2.37 (m, 7H).

^{13}C NMR (101 MHz, $CDCl_3$) δ 140.90, 140.89, 133.3, 132.4, 130.5, 130.4, 130.3, 129.0, 128.3, 128.23, 128.22, 127.7, 126.5, 126.0, 125.2, 121.9, 119.5, 118.6, 117.8, 117.0, 39.7, 27.9, 27.5, 26.5, 22.8, 20.3.

HRMS (ES+) Calcd for $C_{26}H_{25}N_2$ (M + H^+): 365.2012, Found: 365.2015.

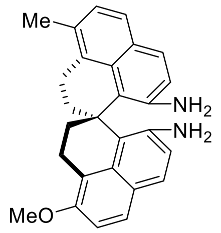

1h

(*S*)-4-Methoxy-4'-methyl-2,2',3,3'-tetrahydro-1,1'-spirobi[phenalene]-9,9'-diamine (1h) was prepared as a white solid from **3h** (124 mg, 0.3 mmol), (*R*)-**A6** (17.3 mg, 15 μmol), DCM (3.0 mL), and toluene (3.0 mL) according to the General Procedure A (eluent: hexanes/DCM = 1:1) in 98% yield (116 mg, 89% ee).

$[\alpha]_D^{20}$: -385.8 (c = 1.0, CH_2Cl_2). HPLC analysis of the product: Daicel CHIRALPAK AD-H column; 20% *i*-PrOH in hexanes; 1.0 mL/min; retention times: 25.4 min (minor), 35.7 min (major).

^{1}H NMR (400 MHz, $CDCl_3$) δ 7.66 – 7.60 (m, 1H), 7.59 – 7.51 (m, 3H), 7.16 (d, *J* = 8.2 Hz, 1H), 7.09 (d, *J* = 8.9 Hz, 1H), 6.73 (d, *J* = 8.6 Hz, 1H), 6.66 (d, *J* = 8.7 Hz, 1H), 3.98 (s, 3H), 3.63 (s, 4H), 3.45 – 3.35 (m, 1H), 3.18 – 2.99 (m, 2H), 2.98 – 2.84 (m, 1H), 2.48 (s, 3H), 2.44 – 2.31 (m, 4H).

^{13}**C NMR** (101 MHz, $CDCl_3$) δ 153.7, 141.2, 140.9, 132.3, 131.3, 130.5, 130.4, 128.2, 128.1, 127.7, 127.1, 125.9, 125.1, 124.3, 118.48, 118.45, 117.5, 117.3, 116.3, 108.8, 56.1, 39.1, 27.5, 27.1, 22.7, 20.3, 18.8.

HRMS (ES+) Calcd for $C_{27}H_{27}N_2O$ (M + H^+): 395.2118, Found: 395.2121.

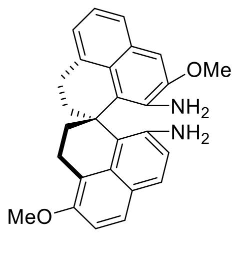

1i

(*R*)-4,8'-Dimethoxy-2,2',3,3'-tetrahydro-1,1'-spirobi[phenalene]-9,9'-diamine (1i) was prepared as a white solid from **3i** (128 mg, 0.3 mmol), (*R*)-**A6** (17.3 mg, 15 μmol), DCM (3.0 mL), and toluene (3.0 mL) according to the General Procedure A (eluent: hexanes/DCM = 1:1) in 93% yield (114 mg, 92% ee).

$[\alpha]_D^{20}$: -259.2 (c = 1.0, CH_2Cl_2). HPLC analysis of the product: Daicel CHIRALPAK AD-H column; 20% *i*-PrOH in hexanes; 1.0 mL/min; retention times: 16.7 min (minor), 60.0 min (major).

^{1}**H NMR** (400 MHz, $CDCl_3$) δ 7.72 – 7.52 (m, 3H), 7.30 – 7.23 (m, 1H), 7.20 (d, *J* = 6.8 Hz, 1H), 7.11 (d, *J* = 8.9 Hz, 1H), 7.08 (s, 1H), 6.64 (d, *J* = 8.6 Hz, 1H), 4.11 – 3.51 (m, 10H), 3.47 – 3.29 (m, 2H), 3.11 – 3.00 (m, 1H), 3.00 – 2.87 (m, 1H), 2.52 – 2.35 (m, 4H).

^{13}**C NMR** (101 MHz, $CDCl_3$) δ 153.7, 147.3, 141.1, 133.5, 133.1, 131.3, 128.6, 128.1, 127.1, 125.4, 125.0, 124.2, 122.9, 122.2, 118.3, 117.6, 117.4, 115.8, 108.7, 104.2, 56.1, 55.2, 39.9, 28.0, 26.6, 26.3, 18.7.

HRMS (ES+) Calcd for $C_{27}H_{27}N_2O_2$ (M + H^+): 411.2067, Found: 411.2068.

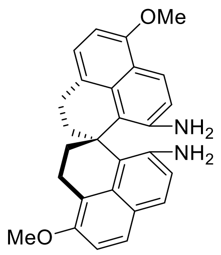

1j

(*S*)-4,6'-Dimethoxy-2,2',3,3'-tetrahydro-1,1'-spirobi[phenalene]-9,9'-diamine (1j) was prepared as a white solid from **3j** (129 mg, 0.3 mmol), (*R*)-**A6** (17.3 mg, 15 μmol), DCM (3.0 mL), and toluene (3.0 mL) according to the General Procedure A (eluent: hexanes/DCM = 1:1) in 97% yield (120 mg, 89% ee).

$[\alpha]_D^{20}$: -227.5 (c = 1.0, CH_2Cl_2). HPLC analysis of the product: Daicel CHIRALPAK AD-H column; 30% *i*-PrOH in hexanes; 1.0 mL/min; retention times: 19.9 min (major), 24.2 min (minor).

^{1}H NMR (400 MHz, $CDCl_3$) δ 8.11 (d, J = 8.9 Hz, 1H), 7.63 (d, J = 8.8 Hz, 1H), 7.54 (d, J = 8.7 Hz, 1H), 7.18 (d, J = 7.7 Hz, 1H), 7.09 (d, J = 8.8 Hz, 1H), 6.79 (d, J = 8.9 Hz, 1H), 6.69 – 6.53 (m, 2H), 4.01 (s, 3H), 3.98 (s, 3H), 3.59 (s, 4H), 3.44 – 3.22 (m, 2H), 3.04 – 2.81 (m, 2H), 2.46 – 2.28 (m, 4H).

^{13}C NMR (101 MHz, $CDCl_3$) δ 154.2, 153.7, 141.5, 141.1, 131.3, 131.2, 128.1, 127.1, 125.5, 124.6, 124.2, 121.9, 120.3, 118.4, 118.3, 118.0, 117.4, 116.1, 108.7, 100.5, 56.1, 55.4, 39.5, 28.3, 27.0, 25.7, 18.7.

HRMS (ES+) Calcd for $C_{27}H_{27}N_2O_2$ (M + H$^+$): 411.2067, Found: 411.2071.

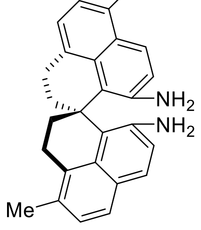

1k

(*S*)-6'-Methoxy-4-methyl-2,2',3,3'-tetrahydro-1,1'-spirobi[phenalene]-9,9'-diamine (1k) was prepared as a white solid from **3k** (124 mg, 0.3 mmol), (*R*)-**A6** (17.3 mg, 15 μmol), DCM (3.0 mL), and toluene (3.0 mL) according to the

General Procedure A (eluent: hexanes/DCM = 1:1) in 93% yield (110 mg, 89% ee).

$[\alpha]_D^{20}$: -229.7 (c = 1.0, CH_2Cl_2). HPLC analysis of the product: Daicel CHIRALPAK AD-3 column; 20% *i*-PrOH in hexanes; 1.0 mL/min; retention times: 20.3 min (major), 29.7 min (minor).

^{1}H NMR (400 MHz, $CDCl_3$) δ 8.08 (d, J = 8.9 Hz, 1H), 7.60 – 7.48 (m, 2H), 7.20 – 7.09 (m, 2H), 6.79 (d, J = 9.0 Hz, 1H), 6.71 (d, J = 8.6 Hz, 1H), 6.63 (d, J = 7.7 Hz, 1H), 3.99 (s, 3H), 3.57 (s, 4H), 3.35 – 3.20 (m, 1H), 3.18 – 2.92 (m, 3H), 2.52 – 2.31 (m, 7H).

^{13}C NMR (101 MHz, $CDCl_3$) δ 154.3, 141.6, 140.8, 132.4, 131.2, 130.5, 130.3, 128.3, 127.7, 125.9, 125.4, 125.1, 124.6, 122.0, 120.3, 118.6, 118.5, 118.0, 117.2, 100.6, 55.4, 39.6, 28.2, 27.4, 25.9, 22.8, 20.3.

HRMS (ES+) Calcd for $C_{27}H_{27}N_2O$ (M + H^+): 395.2118, Found: 395.2125.

2. Gram-scale Synthesis of the SPHENAM 1a

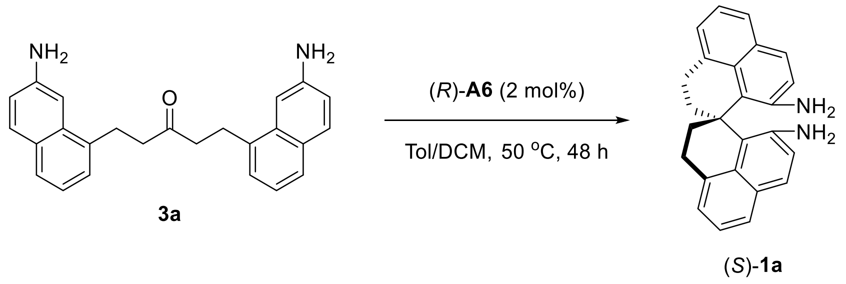

At room temperature, to a solution of **3a** (2.40 g, 6.52 mmol) in toluene (65 mL) and DCM (65 mL) was added (*R*)-**A6** (151 mg, 0.13 mmol). The mixture was stirred at 50 °C for 48 h before it was cooled to room temperature. The mixture was evaporated and subjected to silica gel flash chromatography (eluent: hexanes/DCM = 1:1) to afford the product (*S*)-**1a** as a white solid in 89% yield (2.30 g, 90% ee), which was recrystallized from hexanes/DCM (v/v = 10:1) to give enantiopure (*S*)-**1a** as a white solid in 85% yield (1.95 g, >99% ee).

III. Preparation of Diaryl SPHENAMs

1. Gram-Scale Synthesis of the Chiral Ketone 7a

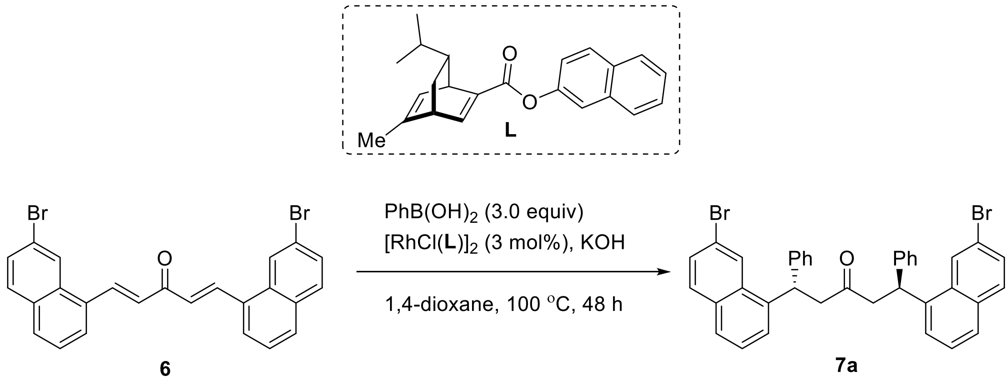

(1*S*,5*S*)-1,5-Bis(7-bromonaphthalen-1-yl)-1,5-diphenylpentan-3-one (7a). At room temperature, a round-bottom flask equipped with a stir bar was charged with **6** (2.45 g, 5.0 mmol), $[RhCl(\mathbf{L})]_2$ (141 mg, 0.15 mmol), an aqueous solution of KOH (1.0 M, 0.4 mL), phenyl boronic acid (1.83 g, 15 mmol), and 1,4-dioxane (50 mL). The flask was evacuated and refilled with N_2 for 5 times. Then, the mixture was stirred under N_2 at 100 °C for 48 h. After cooling to room temperature, the mixture was concentrated and the residue was purified by silica gel flash chromatography (eluent: hexanes/DCM = 3:1) to afford the desired product **7a** as a white solid in 85% yield (2.74 g, >99% ee).

$[\alpha]_D^{20}$: -21.1 (c = 1.0, CH_2Cl_2). HPLC analysis of the product: Daicel CHIRALPAK AD-H column; 5% *i*-PrOH in hexanes; 1.0 mL/min; retention times: 21.2 min (minor), 25.9 min (major).

^{1}H NMR (400 MHz, $CDCl_3$) δ 8.22 (s, 2H), 7.64 (d, J = 8.5 Hz, 4H), 7.51 – 7.42 (m, 2H), 7.37 – 7.28 (m, 2H), 7.25 – 7.06 (m, 12H), 5.34 – 5.08 (m, 2H), 3.35 – 3.03 (m, 4H).

^{13}C NMR (101 MHz, $CDCl_3$) δ 206.1, 143.1, 138.2, 132.6, 132.4, 130.4, 129.0, 128.7, 127.7, 127.2, 126.6, 126.2, 125.6, 124.9, 120.6, 49.9, 41.1.

HRMS (ES+) Calcd for $C_{37}H_{28}Br_2ONa$ (M + Na^+): 671.0379, Found: 671.0391.

2. General Procedure B: Synthesis of the Chiral Ketones 7

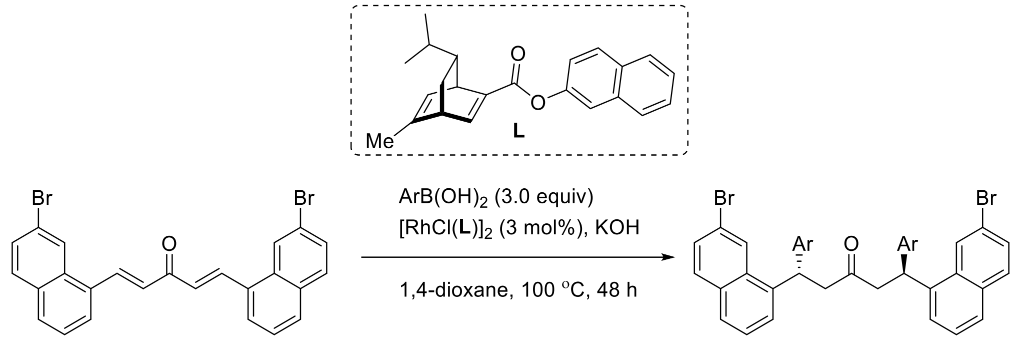

At room temperature, a 25-mL round-bottom flask equipped with a stir bar was charged with **6** (294 mg, 0.6 mmol), [RhCl(**L**)]$_2$ (16.9 mg, 0.018 mmol), an aqueous solution of KOH (1.0 M, 48 μL), aryl boronic acid (2.4 mmol), and 1,4-dioxane (6.0 mL). The flask was evacuated and refilled with N_2 for 5 times. Then, the mixture was stirred under N_2 at 100 °C for 48 h. After cooling to room temperature, the mixture was concentrated, and the residue was purified by silica gel flash chromatography to afford the desired product **7** as a white solid.

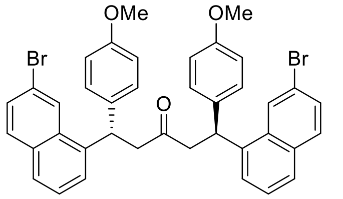

7b

(1*S*,5*S*)-1,5-Bis(7-bromonaphthalen-1-yl)-1,5-bis(4-methoxyphenyl)pentan-3-one (7b) was prepared as a white solid from **6** (294 mg, 0.6 mmol), [RhCl(**L**)]$_2$ (16.9 mg, 0.018 mmol), an aqueous solution of KOH (1.0 M, 48 μL), 4-methoxyphenyl boronic acid (365 mg, 2.4 mmol), and 1,4-dioxane (6.0 mL) according to the General Procedure B (eluent: hexanes/DCM = 3:1) in 82% yield (347 mg, >99% ee).

$[\alpha]_D^{20}$: -44.8 (c = 1.0, CH_2Cl_2). HPLC analysis of the product: Daicel CHIRALPAK AD-H column; 15% *i*-PrOH in hexanes; 1.0 mL/min; retention times: 28.6 min (minor), 37.4 min (major).

^{1}H NMR (400 MHz, $CDCl_3$) δ 8.24 (s, 2H), 7.72 – 7.59 (m, 4H), 7.56 – 7.45 (m, 2H), 7.41 – 7.30 (m, 2H), 7.24 – 7.14 (m, 2H), 7.13 – 7.00 (m, 4H), 6.81 – 6.63 (m, 4H), 5.22 (t, *J* = 7.3 Hz, 2H), 3.74 (s, 6H), 3.31 – 3.06 (m, 4H).

^{13}C NMR (101 MHz, $CDCl_3$) δ 206.5, 158.1, 138.6, 135.1, 132.6, 132.4, 130.4, 128.9, 128.7, 127.1, 126.2, 125.6, 124.7, 120.5, 114.0, 55.1, 50.1, 40.3.

HRMS (ES-) Calcd for $C_{39}H_{22}Br_2NaO_3$ (M + Na^+): 731.0590, Found: 731.0598.

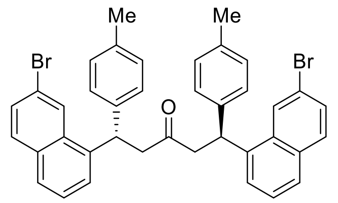

7c

(1*S*,5*S*)-1,5-Bis(7-bromonaphthalen-1-yl)-1,5-di-p-tolylpentan-3-one (7c) was prepared as a white solid from **6** (294 mg, 0.6 mmol), $[RhCl(\mathbf{L})]_2$ (16.9 mg, 0.018 mmol), an aqueous solution of KOH (1.0 M, 48 μL), 4-methylphenyl boronic acid (326 mg, 2.4 mmol), and 1,4-dioxane (6.0 mL) according to the General Procedure B (eluent: hexanes/DCM = 3:1) in 75% yield (302 mg, >99% ee).

$[\alpha]_D^{20}$: -60.2 (*c* = 1.0, CH_2Cl_2). HPLC analysis of the product: Daicel CHIRALPAK AD-H column; 2% *i*-PrOH in hexanes; 1.0 mL/min; retention times: 39.8 min (minor), 44.1 min (major).

^{1}H NMR (400 MHz, $CDCl_3$) δ 8.25 (s, 2H), 7.70 – 7.62 (m, 4H), 7.49 (dd, *J* = 8.7, 1.9 Hz, 2H), 7.37 – 7.29 (m, 2H), 7.23 – 7.18 (m, 2H), 7.07 – 6.98 (m, 8H), 5.23 (t, *J* = 7.4 Hz, 2H), 3.35 – 3.10 (m, 4H).

^{13}C NMR (101 MHz, $CDCl_3$) δ 206.4, 140.1, 138.5, 136.1, 132.6, 132.4, 130.4, 129.4, 128.9, 127.6, 127.1, 126.2, 125.6, 124.8, 120.5, 50.0, 40.7, 21.0.

HRMS (ES+) Calcd for $C_{39}H_{32}Br_2ONa$ (M + Na^+): 699.0692, Found: 699.0707.

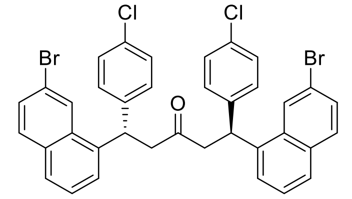

7d

(1*S*,5*S*)-1,5-Bis(7-bromonaphthalen-1-yl)-1,5-bis(4-chlorophenyl)pentan-3-one (7d) was prepared as a white solid from **6** (294 mg, 0.6 mmol), [RhCl(**L**)]$_2$ (16.9 mg, 0.018 mmol), an aqueous solution of KOH (1.0 M, 48 μL), 4-chlorophenyl boronic acid (374 mg, 2.4 mmol), and 1,4-dioxane (6.0 mL) according to the General Procedure B (eluent: hexanes/DCM = 3:1) in 61% yield (260 mg, >99% ee).

$[\alpha]_D^{20}$: -30.6 (c = 1.0, CH_2Cl_2). HPLC analysis of the product: Daicel CHIRALPAK AD-H column; 5% *i*-PrOH in hexanes; 1.0 mL/min; retention times: 37.5 min (minor), 43.7 min (major).

^{1}H NMR (400 MHz, $CDCl_3$) δ 8.16 (s, 2H), 7.72 – 7.63 (m, 4H), 7.55 – 7.48 (m, 2H), 7.40 – 7.33 (m, 2H), 7.22 – 7.13 (m, 6H), 7.10 – 7.02 (m, 4H), 5.25 (t, J = 7.3 Hz, 2H), 3.35 – 3.10 (m, 4H).

^{13}C NMR (101 MHz, $CDCl_3$) δ 205.5, 141.6, 137.7, 132.5, 132.4, 132.4, 130.5, 129.2, 129.0, 128.8, 127.5, 126.0, 125.6, 124.9, 120.8, 49.7, 40.3.

HRMS (ES+) Calcd for $C_{37}H_{26}Br_2Cl_2ONa$ (M + Na^+): 738.9599, Found: 738.9602.

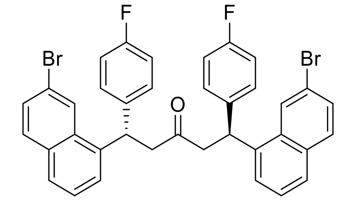

7e

(1*S*,5*S*)-1,5-Bis(7-bromonaphthalen-1-yl)-1,5-bis(4-fluorophenyl)pentan-3-one (7e) was prepared as a white solid from **6** (294 mg, 0.6 mmol), [RhCl(**L**)]$_2$ (16.9 mg, 0.018 mmol), an aqueous solution of KOH (1.0 M, 48 μL), 4-fluorophenyl boronic acid (336 mg, 2.4 mmol), and 1,4-dioxane (6.0 mL)

according to the General Procedure B (eluent: hexanes/DCM = 3:1) in 35% yield (239 mg, >99% ee).

$[\alpha]_D^{20}$: -12.9 (c = 0.5, CH_2Cl_2). HPLC analysis of the product: Daicel CHIRALPAK AD-H column; 10% *i*-PrOH in hexanes; 1.0 mL/min; retention times: 16.9 min (minor), 24.9 min (major).

^{1}H NMR (400 MHz, $CDCl_3$) δ 8.20 (s, 2H), 7.72 – 7.64 (m, 4H), 7.54 – 7.48 (m, 2H), 7.41 – 7.34 (m, 2H), 7.25 – 7.19 (m, 2H), 7.15 – 7.06 (m, 4H), 6.94 – 6.83 (m, 4H), 5.28 (t, J = 7.3 Hz, 2H), 3.33 – 3.14 (m, 4H).

^{13}C NMR (101 MHz, $CDCl_3$) δ 205.7, 161.4 (d, J = 245.0 Hz), 138.8, 138.7, 138.0, 132.4 (d, J = 3.4 Hz), 130.5, 129.1 (d, J = 8.2 Hz), 129.1, 127.4, 126.0, 125.6, 124.8, 120.7, 115.5 (d, J = 21.2 Hz), 49.9, 40.2.

^{19}F NMR (376 MHz, $CDCl_3$) δ -116.0.

HRMS (ES+) Calcd for $C_{37}H_{26}Br_2F_2ONa$ (M + Na^+): 707.0190, Found: 707.0200.

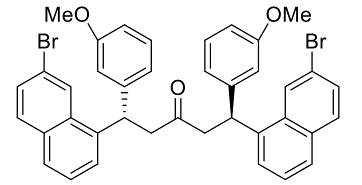

7f

(1*S*,5*S*)-1,5-Bis(7-bromonaphthalen-1-yl)-1,5-bis(3-methoxyphenyl)pentan-3-one (7f) was prepared as a white solid from **6** (294 mg, 0.6 mmol), [RhCl(**L**)]$_2$ (16.9 mg, 0.018 mmol), an aqueous solution of KOH (1.0 M, 48 μL), 3-methoxyphenyl boronic acid (365 mg, 2.4 mmol), and 1,4-dioxane (6.0 mL) according to the General Procedure B (eluent: hexanes/DCM = 3:1) in 80% yield (340 mg, >99% ee).

$[\alpha]_D^{20}$: -19.7 (c = 1.0, CH_2Cl_2). HPLC analysis of the product: Daicel CHIRALPAK IA-3 column; 10% *i*-PrOH in hexanes; 1.0 mL/min; retention times: 16.4 min (minor), 17.6 min (major).

^{1}H NMR (400 MHz, $CDCl_3$) δ 8.24 (s, 2H), 7.70 – 7.60 (m, 4H), 7.54 – 7.42 (m, 2H), 7.37 – 7.29 (m, 2H), 7.23 – 7.10 (m, 4H), 6.80 – 6.73 (m, 2H), 6.73 – 6.64 (m, 4H), 5.24 (t, *J* = 7.3 Hz, 2H), 3.70 (s, 6H), 3.34 – 3.12 (m, 4H).

^{13}C NMR (101 MHz, $CDCl_3$) δ 206.1, 159.7, 144.8, 138.0, 132.6, 132.4, 130.4, 129.7, 129.0, 127.2, 126.2, 125.6, 124.8, 120.5, 120.1, 113.9, 111.6, 55.1, 49.8, 41.1.

HRMS (ES-) Calcd for $C_{39}H_{22}Br_2NaO_3$ (M + Na^+): 731.0590, Found: 731.0606.

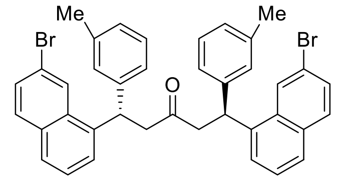

7g

(1*S*,5*S*)-1,5-Bis(7-bromonaphthalen-1-yl)-1,5-di-m-tolylpentan-3-one (7g) was prepared as a white solid from **6** (294 mg, 0.6 mmol), [RhCl(**L**)]$_2$ (16.9 mg, 0.018 mmol), an aqueous solution of KOH (1.0 M, 48 μL), 3-methylphenyl boronic acid (324 mg, 2.4 mmol), and 1,4-dioxane (6.0 mL) according to the General Procedure B (eluent: hexanes/DCM = 3:1) in 91% yield (366 mg, >99% ee).

$[\alpha]_D^{20}$: -35.3 (*c* = 1.0, CH_2Cl_2). HPLC analysis of the product: Daicel CHIRALPAK AD-H column; 20% *i*-PrOH in hexanes; 1.0 mL/min; retention times: 26.0 min (minor), 28.0 min (major).

^{1}H NMR (400 MHz, $CDCl_3$) δ 8.27 (s, 2H), 7.70 – 7.63 (m, 4H), 7.53 – 7.47 (m, 2H), 7.39 – 7.32 (m, 2H), 7.25 – 7.19 (m, 2H), 7.17 – 7.10 (m, 2H), 7.00 – 6.93 (m, 6H), 5.25 (t, *J* = 7.3 Hz, 2H), 3.34 – 3.14 (m, 4H), 2.26 (s, 6H).

^{13}C NMR (101 MHz, $CDCl_3$) δ 206.2, 143.0, 138.3, 132.6, 132.4, 130.4, 128.9, 128.53, 128.47, 127.4, 127.1, 126.2, 125.6, 124.8, 124.7, 120.5, 49.9, 41.0, 21.5.

HRMS (ES+) Calcd for $C_{39}H_{32}Br_2ONa$ (M + Na^+): 699.0692, Found: 699.0707.

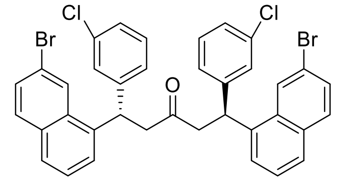

7h

(1*S*,5*S*)-1,5-Bis(7-bromonaphthalen-1-yl)-1,5-bis(3-chlorophenyl)pentan-3-one (7h) was prepared as a white solid from **6** (294 mg, 0.6 mmol), [RhCl(**L**)]$_2$ (16.9 mg, 0.018 mmol), an aqueous solution of KOH (1.0 M, 48 μL), 3-chlorophenyl boronic acid (374 mg, 2.4 mmol), and 1,4-dioxane (6.0 mL) according to the General Procedure B (eluent: hexanes/DCM = 3:1) in 63% yield (270 mg, >99% ee).

$[\alpha]_D^{20}$: -55.1 (c = 1.0, CH_2Cl_2). HPLC analysis of the product: Daicel CHIRALPAK AD-H column; 5% *i*-PrOH in hexanes; 1.0 mL/min; retention times: 19.3 min (minor), 20.9 min (major).

^{1}H NMR (400 MHz, $CDCl_3$) δ 8.17 (s, 2H), 7.73 – 7.65 (m, 4H), 7.55 – 7.47 (m, 2H), 7.38 (t, *J* = 7.8 Hz, 2H), 7.22 (d, *J* = 7.2 Hz, 2H), 7.18 – 6.99 (m, 8H), 5.25 (t, *J* = 7.3 Hz, 2H), 3.24 (d, *J* = 7.3 Hz, 4H).

^{13}C NMR (101 MHz, $CDCl_3$) δ 205.2, 145.2, 137.3, 134.5, 132.5, 132.5, 130.5, 129.9, 129.2, 127.6, 127.6, 127.0, 126.1, 125.9, 125.6, 125.0, 120.8, 49.6, 40.6.

HRMS (ES+) Calcd for $C_{37}H_{26}Br_2Cl_2ONa$ (M + Na$^+$): 738.9599, Found: 738.9620.

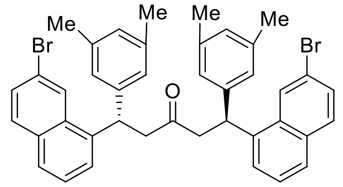

7i

(1*S*,5*S*)-1,5-Bis(7-bromonaphthalen-1-yl)-1,5-bis(3,5-dimethylphenyl)pentan-3-one (7i) was prepared as a white solid from **6** (294 mg, 0.6 mmol), [RhCl(**L**)]$_2$ (16.9 mg, 0.018 mmol), an aqueous solution of KOH (1.0 M, 48 μL), 3,5-dimethylphenyl boronic acid (360 mg, 2.4 mmol), and 1,4-dioxane (6.0 mL)

according to the General Procedure B (eluent: hexanes/DCM = 3:1) in 82% yield (345 mg, >99% ee).

$[\alpha]_D^{20}$: -35.3 (c = 1.0, CH_2Cl_2). HPLC analysis of the product: Daicel CHIRALPAK AD-H column; 2% *i*-PrOH in hexanes; 1.0 mL/min; retention times: 12.6 min (minor), 14.9 min (major).

^{1}H NMR (400 MHz, $CDCl_3$) δ 8.28 (s, 2H), 7.71 – 7.59 (m, 4H), 7.57 – 7.44 (m, 2H), 7.42 – 7.28 (m, 2H), 7.24 – 7.13 (m, 2H), 6.89 – 6.60 (m, 6H), 5.20 (t, *J* = 7.4 Hz, 2H), 3.23 (d, *J* = 7.3 Hz, 4H), 2.22 (s, 12H).

^{13}C NMR (101 MHz, $CDCl_3$) δ 206.3, 143.0, 138.4, 138.1, 132.7, 132.4, 130.3, 128.9, 128.4, 127.0, 126.2, 125.6, 125.5, 124.8, 120.5, 49.9, 41.0, 21.3.

HRMS (ES+) Calcd for $C_{41}H_{36}Br_2ONa$ (M + Na^+): 727.1005, Found: 727.1024.

3. Synthesis of the Chiral Ketones 7j

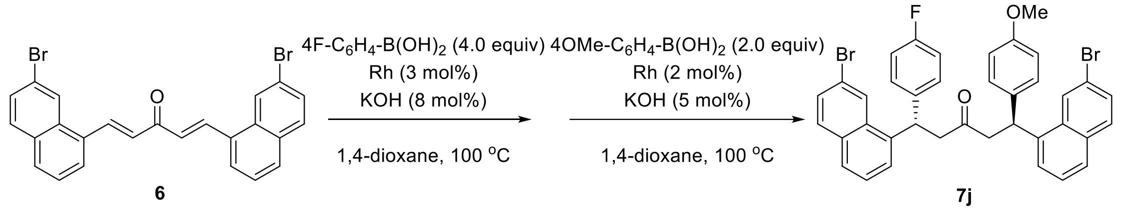

(1*S*,5*S*)-1,5-Bis(7-bromonaphthalen-1-yl)-1-(4-fluorophenyl)-5-(4-methoxyphenyl)pentan-3-one (7j). At room temperature, a 25-mL round-bottom flask equipped with a stir bar was charged with **6** (294 mg, 0.6 mmol), $[RhCl(\mathbf{L})]_2$ (16.9 mg, 0.018 mmol), an aqueous solution of KOH (1.0 M, 48 μL), 4-fluorobenzene boronic acid (336 mg, 2.4 mmol), and 1,4-dioxane (6.0 mL). The flask was evacuated and refilled with N_2 for 5 times. Then, the mixture was stirred under N_2 at 100 °C for 48 h. After cooling to room temperature, the mixture was concentrated, and the residue was purified by silica gel flash chromatography (eluent: hexanes/DCM = 3:1) to afford the enone in 26% yield (149 mg).

At room temperature, a 25-mL round-bottom flask equipped with a stir bar was charged with the above enone (236 mg, 0.4 mmol), $[RhCl(\mathbf{L})]_2$ (7.5 mg, 0.008

mmol), an aqueous solution of KOH (1.0 M, 20 μL), 4-methoxyphenyl boronic acid (0.8 mmol), and 1,4-dioxane (4.0 mL). The flask was evacuated and refilled with N_2 for 5 times. Then, the mixture was stirred under N_2 at 100 °C for 48 h. After cooling to room temperature, the mixture was concentrated and the residue was purified by silica gel flash chromatography (eluent: hexanes/DCM = 3:1) to afford the product **7j** as a white solid in 79% yield (219 mg, >99% ee). $[\alpha]_D^{20}$: -40.7 (c = 0.5, CH_2Cl_2). HPLC analysis of the product: Daicel CHIRALCEL OD-H column; 10% *i*-PrOH in hexanes; 1.0 mL/min; retention times: 24.8 min (minor), 52.5 min (major).

^{1}H NMR (400 MHz, $CDCl_3$) δ 8.36 – 8.19 (m, 2H), 7.71 – 7.61 (m, 4H), 7.57 – 7.46 (m, 2H), 7.44 – 7.34 (m, 2H), 7.33 – 7.28 (m, 1H), 7.24 – 7.02 (m, 7H), 6.86 – 6.70 (m, 2H), 5.37 – 5.17 (m, 2H), 3.74 (s, 3H), 3.36 – 3.09 (m, 4H).

^{13}C NMR (101 MHz, $CDCl_3$) δ 205.9, 161.2 (d, J = 245.3 Hz), 158.0, 138.79, 138.76, 138.5, 138.0, 134.9, 132.4, 132.33, 132.32, 130.3 (d, J = 2.8 Hz), 129.1 (d, J = 7.9 Hz), 128.9, 128.8, 128.5, 127.2, 127.0, 126.1, 126.0, 125.5, 125.5, 124.7, 124.6, 120.5, 120.4, 115.3 (d, J = 21.3 Hz), 113.9, 55.0, 49.8, 49.8, 40.2, 40.0.

^{19}F NMR (376 MHz, $CDCl_3$) δ -115.8.

HRMS (ES+) Calcd for $C_{38}H_{29}Br_2FO_2Na$ (M + Na^+): 719.0390, Found: 719.0406.

4. Gram-scale Synthesis of the Chiral Diphenyl Diamine 8a

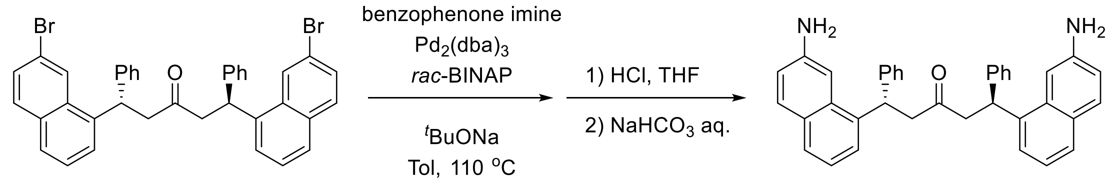

7a
>99% ee

8a

(1*S*,5*S*)-1,5-Bis(7-aminonaphthalen-1-yl)-1,5-diphenylpentan-3-one (8a). At room temperature, a 100-mL round-bottom flask equipped with a stir bar was charged with **7a** (>99% ee, 1.28 g, 2.0 mmol), benzophenone imine (1.12 g, 3.0 mmol), $Pd_2(dba)_3$ (90.0 mg, 0.1 mol), *rac*-BINAP (155 mg, 0.25 mmol), ^{t}BuONa

(720 mg, 7.5 mmol), and toluene (20 mL). The flask was evacuated and refilled with N_2 for 5 times. Then, the mixture was stirred under N_2 at 110 °C for 24 h. After cooling to room temperature, the mixture was evaporated and purified by silica gel flash chromatography (eluent: hexanes/EtOAc = 10:1) to afford the coupling product as a yellow foam.

The yellow foam was dissolved in THF (20 mL) and treated with an aqueous solution of HCl (1.0 M, 10 mL). The mixture was stirred vigorously at room temperature. Upon completion (~ 1 h), the mixture was diluted with EtOAc (20 mL) and carefully poured into a saturated aqueous solution of $NaHCO_3$ at 0 °C. The mixture was extracted with EtOAc (10 mL × 3), washed with brine, dried over Na_2SO_4 and filtered. The filtrate was concentrated and purified by silica gel column chromatography (eluent: DCM/EtOAc = 5:1) to afford the desired product (930 mg).

$[\alpha]_D^{20}$: -29.4 (c = 1.0, CH_2Cl_2).

^{1}H NMR (400 MHz, $CDCl_3$) δ 7.64 (d, J = 8.6 Hz, 2H), 7.58 (d, J = 7.8 Hz, 2H), 7.25 – 7.03 (m, 16H), 6.88 (dd, J = 8.7, 2.2 Hz, 2H), 5.30 – 5.14 (m, 2H), 3.78 (s, 4H), 3.39 – 3.03 (m, 4H).

^{13}C NMR (101 MHz, $CDCl_3$) δ 207.2, 144.4, 143.6, 136.8, 132.8, 130.1, 128.5, 128.3, 127.9, 127.0, 126.3, 124.8, 121.7, 117.7, 104.8, 49.7, 41.1.

HRMS (ES-) Calcd for $C_{37}H_{31}N_2O^-$ (M - H$^+$): 519.2442, Found: 519.2435.

5. General Procedure C: Synthesis of the Chiral Diaryl Diamines 8

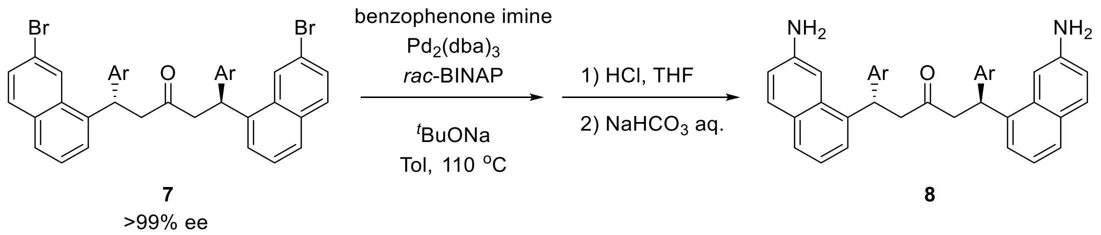

At room temperature, a 25-mL round-bottom flask equipped with a stir bar was charged with **7** (>99% ee, 0.4 mmol), benzophenone imine (224 mg, 1.2 mmol),

$Pd_2(dba)_3$ (18.3 mg, 0.02 mol), *rac*-BINAP (31.1 mg, 0.05 mmol), ^{t}BuONa (144 mg, 1.5 mmol), and toluene (4.0 mL). The flask was evacuated and refilled with N_2 for 5 times. Then, the mixture was stirred under N_2 at 110 °C for 24 h. After cooling to room temperature, the mixture was evaporated and purified by silica gel flash chromatography (eluent: hexanes/EtOAc = 10:1) to afford the product as a yellow foam.

The yellow foam was dissolved in THF (5.0 mL) and treated with an aqueous solution of HCl (1.0 M, 5.0 mL). The mixture was stirred vigorously at room temperature. Upon completion (~ 1 h), the mixture was diluted with EtOAc (20 mL) and carefully poured into a saturated aqueous solution of $NaHCO_3$ at 0 °C. The mixture was extracted with EtOAc (10 mL × 3), washed with brine, dried over Na_2SO_4 and filtered. The filtrate was concentrated and purified by silica gel column chromatography (eluent: DCM/EtOAc = 5:1) to afford the desired product **8** as a yellow foam.

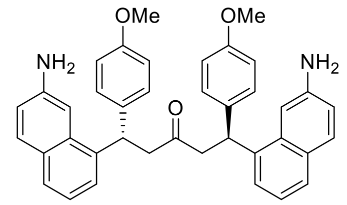

8b

(1*S*,5*S*)-1,5-Bis(7-aminonaphthalen-1-yl)-1,5-bis(4-methoxyphenyl)pentan-3-one (8b) was prepared as a yellow foam from **7b** (>99% ee, 282 mg, 0.4 mmol), benzophenone imine (224 mg, 1.2 mmol), $Pd_2(dba)_3$ (18.3 mg, 0.02 mol), *rac*-BINAP (31.1 mg, 0.05 mmol), ^{t}BuONa (144 mg, 1.5 mmol), and toluene (4.0 mL) according to the General Procedure C (eluent: DCM/EtOAc = 5:1) in 82% yield (189 mg).

$[\alpha]_D^{20}$: -71.2 (*c* = 0.5, CH_2Cl_2).

^{1}H NMR (400 MHz, $CDCl_3$) δ 7.63 (d, *J* = 8.6 Hz, 2H), 7.56 (d, *J* = 7.8 Hz, 2H), 7.19 – 7.02 (m, 10H), 6.88 (dd, *J* = 8.6, 2.2 Hz, 2H), 6.78 – 6.68 (m, 4H), 5.22 – 5.11 (m, 2H), 3.80 (s, 4H), 3.74 (s, 6H), 3.32 – 3.05 (m, 4H).

^{13}**C NMR** (101 MHz, $CDCl_3$) δ 207.5, 157.9, 144.4, 137.3, 135.6, 132.7, 130.1, 128.9, 128.3, 126.9, 124.7, 121.7, 117.7, 113.9, 104.8, 55.1, 49.9, 40.3.

HRMS (ES-) Calcd for $C_{39}H_{35}N_2O_3^-$ (M - H^+): 579.2653, Found: 579.2651.

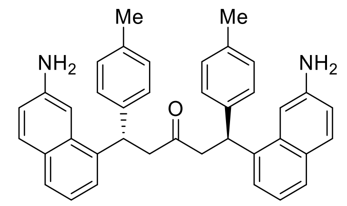

8c

(1*S*,5*S*)-1,5-Bis(7-aminonaphthalen-1-yl)-1,5-di-p-tolylpentan-3-one (8c) was prepared as a yellow foam from **7c** (>99% ee, 270 mg, 0.4 mmol), benzophenone imine (224 mg, 1.2 mmol), $Pd_2(dba)_3$ (18.3 mg, 0.02 mol), *rac*-BINAP (31.1 mg, 0.05 mmol), ^{t}BuONa (144 mg, 1.5 mmol), and toluene (4.0 mL) according to the General Procedure C (eluent: DCM/EtOAc = 5:1) in 86% yield 186 mg).

$[\alpha]_D^{20}$: -81.5 (c = 1.0, CH_2Cl_2).

^{1}**H NMR** (400 MHz, $CDCl_3$) δ 7.63 (d, J = 8.6 Hz, 2H), 7.57 (d, J = 7.0 Hz, 2H), 7.16 (d, J = 2.1 Hz, 2H), 7.13 – 6.98 (m, 14H), 6.88 (dd, J = 8.6, 2.2 Hz, 2H), 5.28 – 5.10 (m, 2H), 3.79 (s, 4H), 3.35 – 3.00 (m, 4H), 2.28 (s, 6H).

^{13}**C NMR** (101 MHz, $CDCl_3$) δ 207.4, 144.4, 140.5, 137.1, 135.7, 132.8, 130.1, 129.2, 128.4, 127.8, 126.9, 124.8, 121.7, 117.7, 104.9, 49.8, 40.7, 20.9.

HRMS (ES-) Calcd for $C_{39}H_{35}N_2O^-$ (M - H^+): 547.2755, Found: 547.2751.

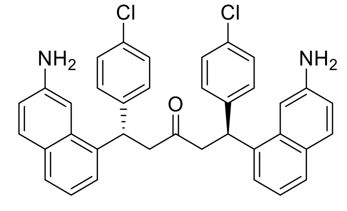

8d

(1*S*,5*S*)-1,5-Bis(7-aminonaphthalen-1-yl)-1,5-bis(4-chlorophenyl)pentan-3-one (8d) was prepared as a yellow foam from **7d** (>99% ee, 286 mg, 0.4 mmol), benzophenone imine (224 mg, 1.2 mmol), $Pd_2(dba)_3$ (18.3 mg, 0.02 mol), *rac*-

BINAP (31.1 mg, 0.05 mmol), ^{t}BuONa (144 mg, 1.5 mmol), and toluene (4.0 mL) according to the General Procedure C (eluent: DCM/EtOAc = 5:1) in 64% yield (152 mg).

$[\alpha]_D^{20}$: -94.2 (c = 1.0, CH_2Cl_2).

^{1}H NMR (400 MHz, $CDCl_3$) δ 7.65 (d, J = 8.7 Hz, 2H), 7.61 (d, J = 8.0 Hz, 2H), 7.19 – 7.10 (m, 6H), 7.10 – 7.01 (m, 8H), 6.92 – 6.84 (m, 2H), 5.20 (t, J = 7.2 Hz, 2H), 3.79 (s, 4H), 3.29 – 3.08 (m, 4H).

^{13}C NMR (101 MHz, $CDCl_3$) δ 206.6, 144.6, 142.1, 136.2, 132.6, 131.9, 130.2, 129.2, 128.5, 128.3, 127.2, 124.7, 121.6, 117.8, 104.4, 49.5, 40.3.

HRMS (ES-) Calcd for $C_{37}H_{29}Cl_2N_2O^-$ (M - H$^+$): 587.1662, Found: 587.1653.

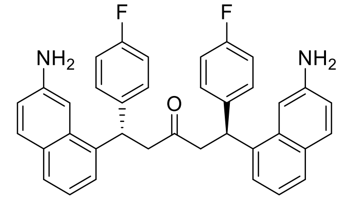

8e

(1*S*,5*S*)-1,5-Bis(7-aminonaphthalen-1-yl)-1,5-bis(4-fluorophenyl)pentan-3-one (8e) was prepared as a yellow foam from **7e** (>99% ee, 273 mg, 0.4 mmol), benzophenone imine (224 mg, 1.2 mmol), $Pd_2(dba)_3$ (18.3 mg, 0.02 mol), *rac*-BINAP (31.1 mg, 0.05 mmol), ^{t}BuONa (144 mg, 1.5 mmol), and toluene (4.0 mL) according to the General Procedure C (eluent: DCM/EtOAc = 5:1) in 90% yield (216 mg).

$[\alpha]_D^{20}$: -22.7 (c = 1.0, CH_2Cl_2).

^{1}H NMR (400 MHz, $CDCl_3$) δ 7.75 – 7.45 (m, 4H), 7.23 – 6.99 (m, 10H), 6.99 – 6.74 (m, 6H), 5.23 (t, J = 7.3 Hz, 2H), 3.80 (s, 4H), 3.35 – 3.00 (m, 4H).

^{13}C NMR (101 MHz, $CDCl_3$) δ 206.9, 161.2 (d, J = 244.9 Hz), 144.6, 139.2, 136.6, 132.7, 130.2, 129.2 (d, J = 7.8 Hz), 128.3, 127.2, 124.7, 121.6, 117.8, 115.2 (d, J = 21.1 Hz), 104.5, 49.8, 40.2.

^{19}F NMR (376 MHz, $CDCl_3$) δ -116.6.

HRMS (ES-) Calcd for $C_{37}H_{29}F_2N_2O^-$ (M - H$^+$): 555.2253, Found: 555.2250.

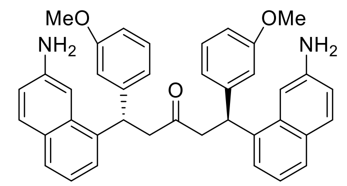

8f

(1S,5S)-1,5-Bis(7-aminonaphthalen-1-yl)-1,5-bis(3-methoxyphenyl)pentan-3-one (8f) was prepared as a yellow foam from **7e** (>99% ee, 282 mg, 0.4 mmol), benzophenone imine (224 mg, 1.2 mmol), $Pd_2(dba)_3$ (18.3 mg, 0.02 mol), *rac*-BINAP (31.1 mg, 0.05 mmol), ^{t}BuONa (144 mg, 1.5 mmol), and toluene (4.0 mL) according to the General Procedure C (eluent: DCM/EtOAc = 5:1) in 76% yield (175 mg).

$[\alpha]_D^{20}$: -45.1 (c = 1.0, CH_2Cl_2).

^{1}**H NMR** (400 MHz, $CDCl_3$) δ 7.64 (d, J = 8.7 Hz, 2H), 7.61 – 7.52 (m, 2H), 7.21 – 7.03 (m, 8H), 6.89 – 6.83 (m, 2H), 6.83 – 6.78 (m, 4H), 6.76 – 6.66 (m, 2H), 5.24 (t, J = 7.2 Hz, 2H), 3.79 (s, 4H), 3.70 (s, 6H), 3.34 – 3.08 (m, 4H).

^{13}**C NMR** (101 MHz, $CDCl_3$) δ 207.1, 159.5, 145.3, 144.5, 136.6, 132.8, 130.0, 129.4, 128.2, 126.9, 124.7, 121.6, 120.3, 117.6, 114.1, 111.2, 104.6, 54.9, 49.6, 41.0.

HRMS (ES-) Calcd for $C_{39}H_{35}N_2O_3^-$ (M - H^+): 579.2653, Found: 579.2647.

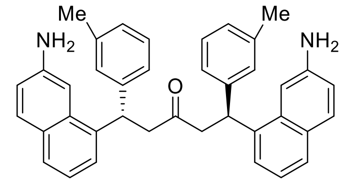

8g

(1*S*,5*S*)-1,5-Bis(7-aminonaphthalen-1-yl)-1,5-di-m-tolylpentan-3-one (8g) was prepared as a yellow foam from **7g** (>99% ee, 270 mg, 0.4 mmol), benzophenone imine (224 mg, 1.2 mmol), $Pd_2(dba)_3$ (18.3 mg, 0.02 mol), *rac*-BINAP (31.1 mg, 0.05 mmol), ^{t}BuONa (144 mg, 1.5 mmol), and toluene (4.0 mL) according to the General Procedure C (eluent: DCM/EtOAc = 5:1) in 73% yield (161 mg).

$[\alpha]_D^{20}$: -43.2 (c = 1.0, CH_2Cl_2).

^{1}**H NMR** (400 MHz, $CDCl_3$) δ 7.64 (d, *J* = 8.7 Hz, 2H), 7.57 (d, *J* = 7.9 Hz, 2H), 7.17 (s, 2H), 7.15 – 7.03 (m, 6H), 7.02 – 6.94 (m, 6H), 6.88 (dd, *J* = 8.6, 2.2 Hz, 2H), 5.24 – 5.07 (m, 2H), 3.80 (s, 4H), 3.37 – 3.00 (m, 4H), 2.25 (s, 6H).

^{13}**C NMR** (101 MHz, $CDCl_3$) δ 207.2, 144.4, 143.5, 138.0, 137.0, 132.8, 130.1, 128.8, 128.4, 128.3, 127.1, 126.9, 124.9, 121.8, 117.7, 104.9, 49.8, 41.0, 21.5.

HRMS (ES-) Calcd for $C_{39}H_{35}N_2O^-$ (M - H^+): 547.2755, Found: 547.2748.

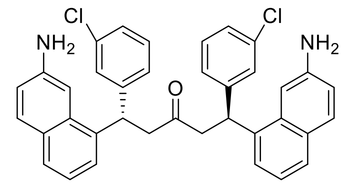

8h

(1*S*,5*S*)-1,5-Bis(7-aminonaphthalen-1-yl)-1,5-bis(3-chlorophenyl)pentan-3-one (8h) was prepared as a yellow foam from **7h** (>99% ee, 286 mg, 0.4 mmol), benzophenone imine (224 mg, 1.2 mmol), $Pd_2(dba)_3$ (18.3 mg, 0.02 mol), *rac*-BINAP (31.1 mg, 0.05 mmol), ^{t}BuONa (144 mg, 1.5 mmol), and toluene (4.0 mL) according to the General Procedure C (eluent: DCM/EtOAc = 5:1) in 72% yield (166 mg).

$[\alpha]_D^{20}$: -66.9 (*c* = 1.0, CH_2Cl_2).

^{1}**H NMR** (400 MHz, $CDCl_3$) δ 7.81 – 7.38 (m, 4H), 7.23 – 6.95 (m, 14H), 6.94 – 6.75 (m, 2H), 5.38 – 4.86 (m, 2H), 3.79 (s, 4H), 3.41 – 2.89 (m, 4H).

^{13}**C NMR** (101 MHz, $CDCl_3$) δ 206.3, 145.8, 144.6, 135.8, 134.2, 132.7, 130.2, 129.7, 128.3, 127.8, 127.3, 126.5, 126.1, 124.7, 121.6, 117.8, 104.4, 49.4, 40.6.

HRMS (ES-) Calcd for $C_{37}H_{29}Cl_2N_2O^-$ (M - H^+): 587.1662, Found: 587.1658.

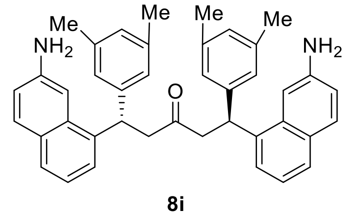

8i

(1*S*,5*S*)-1,5-Bis(7-aminonaphthalen-1-yl)-1,5-bis(3,5-dimethylphenyl)pentan-

3-one (8i) was prepared as a yellow foam from **7i** (>99% ee, 281 mg, 0.4 mmol), benzophenone imine (224 mg, 1.2 mmol), $Pd_2(dba)_3$ (18.3 mg, 0.02 mol), *rac*-BINAP (31.1 mg, 0.05 mmol), ^{t}BuONa (144 mg, 1.5 mmol), and toluene (4.0 mL) according to the General Procedure C (eluent: DCM/EtOAc = 5:1) in 77% yield (177 mg).

$[\alpha]_D^{20}$: -50.8 (c = 1.0, CH_2Cl_2).

^{1}H NMR (400 MHz, $CDCl_3$) δ 7.67 (d, J = 8.7 Hz, 2H), 7.65 – 7.58 (m, 2H), 7.25 – 7.21 (m, 2H), 7.21 – 7.12 (m, 4H), 6.94 – 6.80 (m, 8H), 5.25 (t, J = 7.3 Hz, 2H), 3.77 (s, 4H), 3.38 – 3.08 (m, 4H), 2.29 (s, 12H).

^{13}C NMR (101 MHz, $CDCl_3$) δ 207.3, 144.4, 143.5, 137.8, 136.9, 132.8, 130.0, 128.2, 128.0, 126.8, 125.7, 124.7, 121.6, 117.6, 104.7, 49.8, 40.9, 21.3.

HRMS (ES-) Calcd for $C_{41}H_{39}N_2O^-$ (M - H^+): 575.3068, Found: 575.3061.

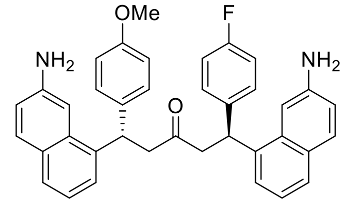

8j

(1*S*,5*S*)-1,5-Bis(7-aminonaphthalen-1-yl)-1-(4-fluorophenyl)-5-(4-methoxyphenyl)pentan-3-one (8j) was prepared as a yellow foam from **7j** (>99% ee, 278 mg, 0.4 mmol), benzophenone imine (224 mg, 1.2 mmol), $Pd_2(dba)_3$ (18.3 mg, 0.02 mol), *rac*-BINAP (31.1 mg, 0.05 mmol), ^{t}BuONa (144 mg, 1.5 mmol), and toluene (4.0 mL) according to the General Procedure C (eluent: DCM/EtOAc = 5:1) in 74% yield (166 mg).

$[\alpha]_D^{20}$: -48.3 (c = 0.3, CH_2Cl_2).

^{1}H NMR (400 MHz, $CDCl_3$) δ 7.74 – 7.54 (m, 4H), 7.22 – 6.98 (m, 10H), 6.93 – 6.82 (m, 4H), 6.77 (d, J = 8.5 Hz, 2H), 5.28 – 5.14 (m, 2H), 3.91 – 3.56 (m, 7H), 3.33 – 3.03 (m, 4H).

^{13}C NMR (101 MHz, $CDCl_3$) δ 207.2, 161.2 (d, *J* = 244.5 Hz), 157.9, 144.6, 144.5, 139.3, 139.2, 137.1, 136.7, 135.5, 132.7, 132.6, 130.1 (d, *J* = 3.6 Hz), 129.3 (d, *J* = 7.9 Hz), 128.8, 128.27, 128.24, 127.1, 126.9, 124.7, 124.6, 121.6, 117.7, 117.7, 115.1 (d, *J* = 21.2 Hz), 113.8, 104.7, 104.5, 55.1, 49.8, 40.3, 40.1.

^{19}F NMR (376 MHz, $CDCl_3$) δ -116.7.

HRMS (ES-) Calcd for $C_{38}H_{31}FN_2O_2^-$ (M - H$^+$): 567.2453, Found: 567.2447.

6. Gram-Scale Synthesis of the Diphenyl SPHENAM 9a

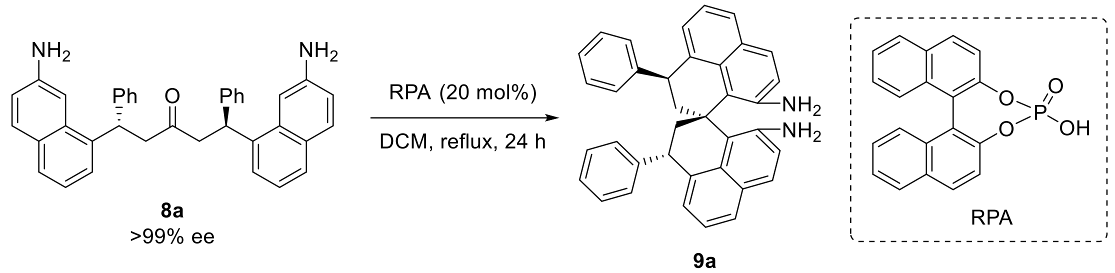

(1*S*,3*S*,3'S)-3,3'-Diphenyl-2,2',3,3'-tetrahydro-1,1'-spirobi[phenalene]-9,9'-diamine (9a). At room temperature, to a solution of the chiral diaryl diamine **8a** (1.69 g, 3.25 mmol) in DCM (33 mL) was added RPA (224 mg, 0.65 mmol). The mixture was heated to reflux for 24 h. The reaction progress was monitored by thin layer chromatography. Upon completion, the mixture was cooled to room temperature, evaporated and subjected to silica gel flash chromatography (eluent: hexanes/DCM = 1:1) to afford the desired product **9a** in 82% yield (1.33 g).

$[\alpha]_D^{20}$: +301.6 (*c* = 1.0, CH_2Cl_2).

^{1}H NMR (400 MHz, $CDCl_3$) δ 7.70 – 7.62 (m, 4H), 7.24 – 7.18 (m, 2H), 7.17 – 7.08 (m, 6H), 7.00 (d, *J* = 7.0 Hz, 2H), 6.85 (d, *J* = 8.7 Hz, 2H), 6.81 – 6.71 (m, 4H), 4.64 (t, *J* = 5.9 Hz, 2H), 3.72 (s, 4H), 3.07 (dd, *J* = 13.7, 5.7 Hz, 2H), 2.25 (dd, *J* = 13.8, 6.2 Hz, 2H).

^{13}C NMR (101 MHz, $CDCl_3$) δ 146.2, 141.9, 135.5, 130.8, 129.1, 128.7, 128.6, 128.0, 126.89, 126.88, 125.5, 122.0, 120.1, 117.4, 43.3, 42.3, 41.8.

HRMS (ES-) Calcd for $C_{37}H_{39}N_2^-$ (M - H$^+$): 501.2335, Found: 501.2336.

7. General Procedure D: Synthesis of the Diaryl SPHENAMs 9

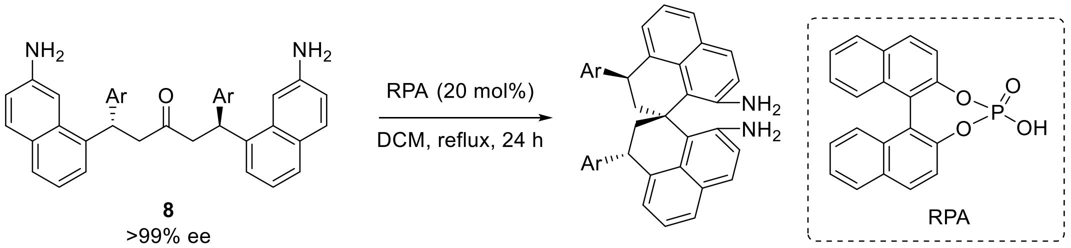

At room temperature, to a solution of the chiral diaryl diamine **8** (>99% ee, 0.2 mmol) in DCM (2.0 mL) were added RPA (13.6 mg, 20 mol%). The mixture was heated to reflux for 24 h. The reaction progress was monitored by thin layer chromatography. Upon completion, the mixture was cooled to room temperature and directly subjected to silica gel flash chromatography (eluent: hexanes/DCM = 1:1) to afford the desired product **9**.

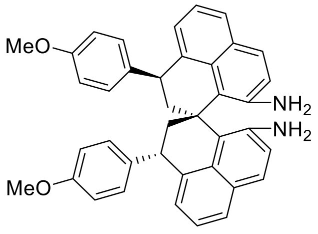

9b

(1*S*,3*S*,3'*S*)-3,3'-Bis(4-methoxyphenyl)-2,2',3,3'-tetrahydro-1,1'-spirobi[phenalene]-9,9'-diamine (9b) was prepared as a white foam from **8b** (>99% ee, 116 mg, 0.2 mmol), RPA (13.8 mg, 0.04 mmol), and DCM (2.0 mL) according to the General Procedure D (eluent: DCM/hexanes = 1:1) in 89% yield (99.8 mg).

$[\alpha]_D^{20}$: +249.8 (c = 1.0, CH_2Cl_2).

^{1}H NMR (400 MHz, $CDCl_3$) δ 7.70 – 7.59 (m, 4H), 7.24 – 7.11 (m, 2H), 7.03 – 6.96 (m, 2H), 6.83 (d, J = 8.7 Hz, 2H), 6.68 (s, 8H), 4.57 (t, J = 5.9 Hz, 2H), 3.78 (s, 6H), 3.75 (s, 4H), 2.99 (dd, J = 13.8, 5.7 Hz, 2H), 2.20 (dd, J = 13.8, 6.2 Hz, 2H).

^{13}C NMR (101 MHz, $CDCl_3$) δ 157.5, 141.9, 138.5, 135.9, 130.8, 129.6, 129.1, 128.6, 126.9, 126.8, 122.0, 120.1, 117.5, 113.3, 55.2, 42.5, 42.4, 41.8.

HRMS (ES-) Calcd for $C_{39}H_{33}N_2O_2^-$ (M - H^+): 561.2548, Found: 561.2542.

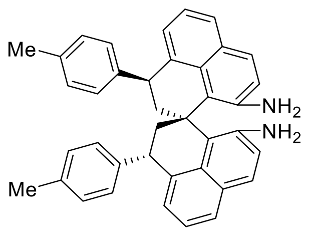

9c

(1*S*,3*S*,3'*S*)-3,3'-Di-*p*-tolyl-2,2',3,3'-tetrahydro-1,1'-spirobi[phenalene]-9,9'-diamine (9c) was prepared as a white foam from **8c** (>99% ee, 110 mg, 0.2 mmol), RPA (13.8 mg, 0.04 mmol), and DCM (2.0 mL) according to the General Procedure D (eluent: DCM/hexanes = 1:1) in 86% yield (91.2 mg).

$[\alpha]_D^{20}$: +293.2 (c = 0.7, CH_2Cl_2).

^{1}H NMR (400 MHz, $CDCl_3$) δ 7.70 – 7.60 (m, 4H), 7.24 – 7.16 (m, 2H), 7.03 – 6.98 (m, 2H), 6.95 (d, J = 7.8 Hz, 4H), 6.84 (d, J = 8.7 Hz, 2H), 6.71 – 6.57 (m, 4H), 4.60 (t, J = 6.0 Hz, 2H), 3.77 (s, 4H), 3.02 (dd, J = 13.7, 5.7 Hz, 2H), 2.35 (s, 6H), 2.22 (dd, J = 13.8, 6.3 Hz, 2H).

^{13}C NMR (101 MHz, $CDCl_3$) δ 143.2, 141.9, 135.8, 134.9, 130.8, 129.1, 128.57, 128.55 (2C), 128.52, 126.8, 122.0, 120.1, 117.5, 42.9, 42.5, 41.8, 20.9.

HRMS (ES-) Calcd for $C_{39}H_{33}N_2^-$ (M - H^+): 529.2649, Found: 529.2643.

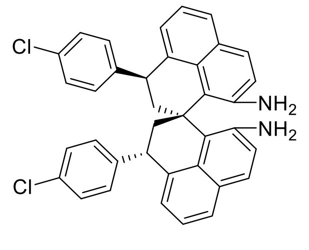

9d

(1*S*,3*S*,3'*S*)-3,3'-Bis(4-chlorophenyl)-2,2',3,3'-tetrahydro-1,1'-spirobi[phena lene]-9,9'-diamine (9d) was prepared as a white foam from **8e** (>99% ee, 112 mg, 0.2 mmol), RPA (13.8 mg, 0.04 mmol), and DCM (2.0 mL) according to the General Procedure D (eluent: DCM/hexanes = 1:1) in 92% yield (104.7 mg).

[α]$_D^{20}$: +302.3 (c = 1.0, CH_2Cl_2).

^{1}H NMR (400 MHz, $CDCl_3$) δ 7.75 – 7.56 (m, 4H), 7.21 (t, J = 7.6 Hz, 2H), 7.14 – 7.04 (m, 4H), 6.99 (d, J = 7.0 Hz, 2H), 6.84 (d, J = 8.7 Hz, 2H), 6.70 – 6.51 (m, 4H), 4.57 (t, J = 5.7 Hz, 2H), 3.69 (s, 4H), 2.97 (dd, J = 14.0, 6.2 Hz, 2H), 2.25 (dd, J = 14.0, 5.2 Hz, 2H).

^{13}C NMR (101 MHz, $CDCl_3$) δ 145.2, 141.7, 134.4, 131.2, 130.6, 129.9, 129.0, 128.8, 128.1, 127.2, 122.1, 120.2, 117.0, 42.9, 41.3, 40.5.

HRMS (ES-) Calcd for $C_{37}H_{27}Cl_2N_2^-$ (M - H$^+$): 569.1557, Found: 569.1551.

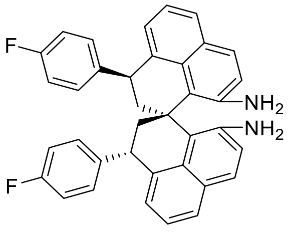

9e

(1*S*,3*S*,3'*S*)-3,3'-Bis(4-fluorophenyl)-2,2',3,3'-tetrahydro-1,1'-spirobi[phenalene]-9,9'-diamine (9e) was prepared as a white foam from **8d** (>99% ee, 107 mg, 0.2 mmol), RPA (13.8 mg, 0.04 mmol), and DCM (2.0 mL) according to the General Procedure D (eluent: DCM/hexanes = 1:1) in 80% yield (85.7 mg).

[α]$_D^{20}$: +261.5 (c = 1.0, CH_2Cl_2).

^{1}H NMR (400 MHz, $CDCl_3$) δ 7.72 – 7.59 (m, 4H), 7.21 (t, J = 7.6 Hz, 2H), 6.99 (d, J = 7.0 Hz, 2H), 6.88 – 6.76 (m, 6H), 6.76 – 6.58 (m, 4H), 4.60 (t, J = 5.8 Hz, 2H), 3.70 (s, 4H), 3.01 (dd, J = 13.9, 5.9 Hz, 2H), 2.23 (dd, J = 13.9, 5.7 Hz, 2H).

^{13}C NMR (101 MHz, $CDCl_3$) δ 161.0 (d, J = 243.8 Hz), 142.2 (d, J = 3.1 Hz), 141.9, 135.0, 130.7, 130.0 (d, J = 7.7 Hz), 129.0, 128.7, 127.1 (d, J = 5.6 Hz), 122.1, 120.2, 117.1, 114.7 (d, J = 21.1 Hz), 42.7, 41.6, 41.5.

^{19}F NMR (376 MHz, $CDCl_3$) δ -117.8.

HRMS (ES-) Calcd for $C_{37}H_{27}F_2N_2^-$ (M - H$^+$): 537.2148, Found: 537.2143.

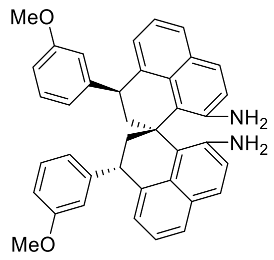

9f

(1*S*,3*S*,3'*S*)-3,3'-Bis(3-methoxyphenyl)-2,2',3,3'-tetrahydro-1,1'-spirobi[phenalene]-9,9'-diamine (9f) was prepared as a white foam from **8f** (>99% ee, 116 mg, 0.2 mmol), RPA (13.8 mg, 0.04 mmol), and DCM (2.0 mL) according to the General Procedure D (eluent: DCM/hexanes = 1:1) in 72% yield (80.4 mg).

$[\alpha]_D^{20}$: +310.0 (c = 1.0, CH_2Cl_2).

^{1}H NMR (400 MHz, $CDCl_3$) δ 7.75 – 7.57 (m, 4H), 7.22 (t, J = 7.5 Hz, 2H), 7.12 – 6.98 (m, 4H), 6.85 (d, J = 8.7 Hz, 2H), 6.76 – 6.66 (m, 2H), 6.52 – 6.27 (m, 4H), 4.65 (t, J = 6.0 Hz, 2H), 3.80 (d, J = 35.0 Hz, 4H), 3.67 (s, 6H), 3.13 (dd, J = 13.7, 5.8 Hz, 2H), 2.29 (dd, J = 13.7, 6.3 Hz, 2H).

^{13}C NMR (101 MHz, $CDCl_3$) δ 159.2, 148.0, 141.9, 135.4, 130.7, 129.1, 128.7, 128.6, 126.9, 122.0, 121.3, 120.1, 117.2, 114.2, 111.2, 54.9, 43.4, 42.5, 41.8.

HRMS (ES-) Calcd for $C_{39}H_{33}N_2O_2^-$ (M - H$^+$): 561.2548, Found: 561.2543.

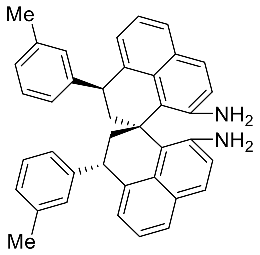

9g

(1*S*,3*S*,3'*S*)-3,3'-Di-m-tolyl-2,2',3,3'-tetrahydro-1,1'-spirobi[phenalene]-9,9'-diamine (9g) was prepared as a white foam from **8g** (>99% ee, 116 mg, 0.2 mmol), RPA (13.8 mg, 0.04 mmol), and DCM (2.0 mL) according to the General Procedure D (eluent: DCM/hexanes = 1:1) in 89% yield (99.8 mg).

$[\alpha]_D^{20}$: +306.7 (c = 1.0, CH_2Cl_2).

^{1}**H NMR** (400 MHz, $CDCl_3$) δ 7.76 – 7.56 (m, 4H), 7.24 – 7.15 (m, 2H), 7.11 – 6.92 (m, 6H), 6.92 – 6.80 (m, 2H), 6.76 – 6.53 (m, 4H), 4.63 (t, *J* = 6.2 Hz, 2H), 3.85 (s, 4H), 3.12 (dd, *J* = 13.6, 5.6 Hz, 2H), 2.21 (s, 8H).

^{13}**C NMR** (101 MHz, $CDCl_3$) δ 146.0, 142.1, 137.3, 135.9, 130.8, 129.3, 129.1, 128.7, 127.7, 126.8, 126.7, 126.3, 126.0, 122.0, 120.0, 117.3, 44.0, 43.1, 42.2, 21.3.

HRMS (ES-) Calcd for $C_{39}H_{33}N_2^-$ (M - H^+): 529.2649, Found: 529.2646.

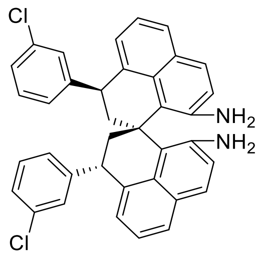

9h

(1*S*,3*S*,3'*S*)-3,3'-Bis(3-chlorophenyl)-2,2',3,3'-tetrahydro-1,1'-spirobi[phenalene]-9,9'-diamine (9h) was prepared as a white foam from **8h** (>99% ee, 104 mg, 0.2 mmol), RPA (13.8 mg, 0.04 mmol), and DCM (2.0 mL) according to the General Procedure D (eluent: DCM/hexanes = 1:1) in 91% yield (91.7 mg).

$[\alpha]_D^{20}$: +290.3 (*c* = 1.0, CH_2Cl_2).

^{1}**H NMR** (400 MHz, $CDCl_3$) δ 7.74 – 7.62 (m, 4H), 7.23 (t, *J* = 7.6 Hz, 2H), 7.19 – 7.13 (m, 2H), 7.11 – 7.03 (m, 2H), 7.00 (d, *J* = 7.1 Hz, 2H), 6.85 (d, *J* = 8.7 Hz, 2H), 6.82 – 6.61 (m, 4H), 4.62 (t, *J* = 6.0 Hz, 2H), 3.74 (s, 4H), 3.13 – 3.00 (m, 2H), 2.29 – 2.12 (m, 2H).

^{13}**C NMR** (101 MHz, $CDCl_3$) δ 148.5, 142.0, 134.4, 133.9, 130.6, 129.16, 129.15, 128.8, 128.6, 127.3, 127.1, 126.9, 126.0, 122.1, 120.2, 116.8, 43.1, 42.3, 41.6.

HRMS (ES-) Calcd for $C_{37}H_{27}Cl_2N_2^-$ (M - H^+): 569.1557, Found: 569.1557.

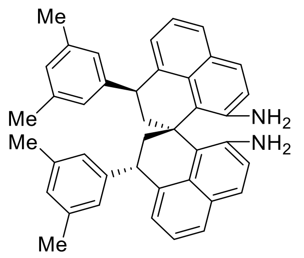

9i

(1*S*,3*S*,3'*S*)-3,3'-Bis(3,5-dimethylphenyl)-2,2',3,3'-tetrahydro-1,1'-spirobi[phenalene]-9,9'-diamine (9i) was prepared as a white foam from **8i** (>99% ee, 115 mg, 0.2 mmol), RPA (13.8 mg, 0.04 mmol), and DCM (2.0 mL) according to the General Procedure D (eluent: DCM/hexanes = 1:1) in 95% yield (105.6 mg).

$[\alpha]_D^{20}$: +314.9 (c = 1.0, CH_2Cl_2).

^{1}H NMR (400 MHz, $CDCl_3$) δ 7.70 (d, J = 8.7 Hz, 4H), 7.23 (d, J = 7.7 Hz, 2H), 7.04 (d, J = 7.0 Hz, 2H), 6.97 – 6.77 (m, 4H), 6.67 – 6.42 (m, 4H), 4.64 (t, J = 6.4 Hz, 2H), 3.86 (s, 4H), 3.16 (dd, J = 13.5, 5.4 Hz, 2H), 2.32 – 2.03 (m, 14H).

^{13}C NMR (101 MHz, $CDCl_3$) δ 145.9, 142.2, 137.1, 136.2, 130.8, 129.3, 128.6, 127.2, 126.7, 126.6, 126.5, 121.9, 119.9, 117.2, 44.9, 42.9, 42.3, 21.1.

HRMS (ES-) Calcd for $C_{41}H_{37}N_2^-$ (M - H^+): 557.2962, Found: 557.2964.

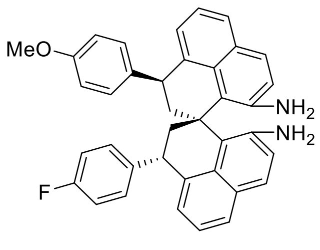

9j

(1*S*,3*S*,3'*S*)-3-(4-fluorophenyl)-3'-(4-methoxyphenyl)-2,2',3,3'-tetrahydro-1,1'-spirobi[phenalene]-9,9'-diamine (9j) was prepared as a white foam from **8j** (>99% ee, 117 mg, 0.2 mmol), RPA (13.8 mg, 0.04 mmol), and DCM (2.0 mL) according to the General Procedure D (eluent: DCM/hexanes = 1:1) in 83% yield (93.6 mg).

$[\alpha]_D^{20}$: +323.1 (c = 1.0, CH_2Cl_2).

^{1}**H NMR** (400 MHz, $CDCl_3$) δ 7.72 – 7.57 (m, 4H), 7.25 – 7.15 (m, 2H), 7.08 – 6.94 (m, 2H), 6.89 – 6.76 (m, 4H), 6.76 – 6.53 (m, 6H), 4.66 – 4.51 (m, 2H), 3.79 (s, 7H), 3.06 – 2.92 (m, 2H), 2.29 – 2.15 (m, 2H).

^{13}**C NMR** (101 MHz, $CDCl_3$) δ 160.9 (d, *J* = 243.7 Hz), 157.5, 142.10, 142.08, 141.9, 141.8, 138.6, 135.6, 135.2, 130.7, 130.0 (d, *J* = 7.3 Hz), 129.5, 129.1 (d, *J* = 1.9 Hz), 128.7, 128.6, 127.1, 126.9, 122.1, 122.0, 120.2, 120.1, 117.3, 117.3, 114.6 (d, *J* = 21.0 Hz), 113.3, 55.2, 42.7, 42.6, 42.0, 41.9, 41.6.

^{19}**F NMR** (376 MHz, $CDCl_3$) δ -118.0.

HRMS (ES-) Calcd for $C_{38}H_{30}FN_2O^-$ (M - H^+): 549.2348, Found: 549.2347.

IV. Preparation of NOSPHEN

1. Synthesis of the 7-Nitro-1-Naphthaldehyde 11

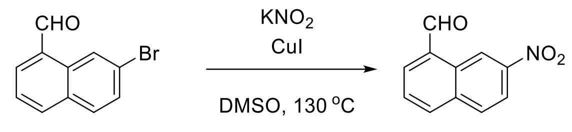

7-Nitro-1-naphthaldehyde (11). To a solution of 7-bromo-1-naphthaldehyde **5** (7.02 g, 30 mmol) in DMSO (100 mL) were added KNO_2 (7.65 g, 90 mmol) and CuI (8.55 g, 45 mmol). Then the mixture was stirred at 130 °C for 24 h. Next, the solvent was removed by vacuum distillation, and the residue was purified by silica gel column chromatography (eluent: hexanes/DCM = 1:1) to afford the product **11** as a yellow solid in 63% yield (3.80 g).

^{1}H NMR (400 MHz, $CDCl_3$) δ 10.38 (s, 1H), 10.17 (d, *J* = 2.2 Hz, 1H), 8.33 (dd, *J* = 9.0, 2.3 Hz, 1H), 8.20 (d, *J* = 8.3 Hz, 1H), 8.14 (dd, *J* = 7.1, 1.3 Hz, 1H), 8.05 (d, *J* = 9.0 Hz, 1H), 7.87 (dd, *J* = 8.3, 7.1 Hz, 1H).

^{13}C NMR (101 MHz, $CDCl_3$) δ 192.6, 147.7, 138.2, 136.0, 134.7, 132.6, 129.9, 129.0, 128.6, 121.9, 120.6.

HRMS (CI) Calcd for $C_{11}H_7NO_3$ (M): 201.0426, Found: 201.0430.

2. Synthesis of the Enone S10

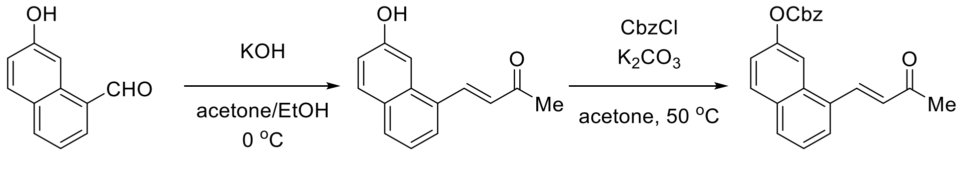

(*E*)-Benzyl (8-(3-oxobut-1-en-1-yl)naphthalen-2-yl) carbonate (S9). At 0 °C, to a solution of KOH (4.50 g, 80 mmol) in absolute EtOH (100 mL) and acetone (100 mL) was slowly added 7- hydroxy-1-naphthaldehyde **10** (6.88 g, 40 mmol). The mixture was stirred at 0 °C for additional 2 h and then carefully poured into an aqueous solution of hydrochloric acid (2.0 M, 50 mL) at 0 °C. The

mixture was extracted with EtOAc (30 mL × 3), washed with brine, dried over Na_2SO_4 and filtered. The filtrate was concentrated and purified by silica gel column chromatography (eluent: hexanes/EtOAc = 4:1) to afford the desired enone **S9** as a yellow solid in 87% yield (7.38 g).

Next, the enone **S9** (1.06 g, 5.0 mmol) was dissolved in acetone (60 mL), to which were added K_2CO_3 (1.38 g, 10 mmol) and benzyl chloroformate (1.70 g, 10 mmol). Then, the mixture was stirred at 50 °C for 12 h before it was filtered through a glass frit and the filter cake was washed with DCM (10 mL × 3). The filtrate was concentrated, and the crude product was purified by silica gel flash chromatography (eluent: hexanes/DCM = 2:1) to afford the desired enone **S10** as a white solid in 97% yield (1.68 g).

^{1}H NMR (400 MHz, $CDCl_3$) δ 8.21 (d, *J* = 16.0 Hz, 1H), 7.95 (s, 1H), 7.90 (d, *J* = 8.7 Hz, 2H), 7.78 (d, *J* = 7.2 Hz, 1H), 7.51 – 7.45 (m, 3H), 7.45 – 7.34 (m, 4H), 6.79 (d, *J* = 16.0 Hz, 1H), 5.33 (s, 2H), 2.46 (s, 3H).

^{13}C NMR (101 MHz, $CDCl_3$) δ 198.0, 153.6, 149.5, 139.4, 134.5, 131.9, 131.7, 131.6, 130.44, 130.38, 129.8, 128.8, 128.7, 128.6, 125.8, 125.5, 121.0, 114.1, 70.5, 27.8.

HRMS (ES+) Calcd for $C_{22}H_{18}NaO_4^+$ (M + Na^+): 369.1097, Found: 369.1103.

3. Synthesis of the Dienone 12

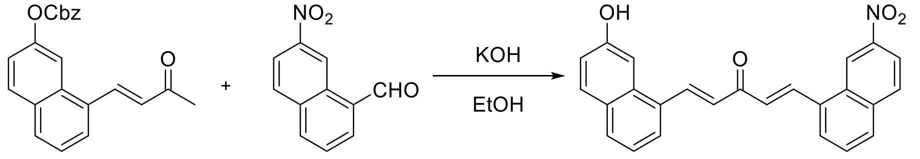

S10 11 12

(1*E*,4*E*)-1-(7-Hydroxynaphthalen-1-yl)-5-(7-nitronaphthalen-1-yl)penta-1,4-dien-3-one (12). At room temperature, to a solution of the enone **S10** (1.38 g, 4.0 mmol) and 7-nitro-1-naphthaldehyde **11** (804 mg, 4.0 mmol) in absolute EtOH (20 mL) was slowly added KOH (448 mg, 8.0 mmol). The mixture was stirred at room temperature for 24 h and then cooled to 0 °C, to which was added an aqueous solution of hydrochloric acid (2.0 M, 5.0 mL). The mixture was then

stirred vigorously for 10 min before it was filtered through glass frit, and the red filter cake was washed with water (10 mL × 3) and MeOH (10 mL × 3). This red solid was dried under vacuum to afford dienone **12** in 89% yield (1.41 g).

^{1}H NMR (400 MHz, DMSO-*d*$_6$) δ 10.13 (s, 1H), 9.14 (s, 1H), 8.60 (d, *J* = 15.7 Hz, 1H), 8.50 (d, *J* = 15.7 Hz, 1H), 8.35 – 8.12 (m, 4H), 8.02 (d, *J* = 7.3 Hz, 1H), 7.91 (d, *J* = 8.1 Hz, 1H), 7.89 – 7.80 (m, 2H), 7.64 – 7.44 (m, 3H), 7.36 (t, *J* = 7.7 Hz, 1H), 7.24 – 7.09 (m, 1H).

^{13}C NMR (101 MHz, DMSO-*d*$_6$) δ 188.3, 156.6, 145.7, 139.9, 137.7, 135.9, 134.2, 133.2, 130.9, 130.9, 130.6, 130.5, 130.1, 129.8, 129.7, 129.4, 128.2, 127.4, 127.3, 125.9, 122.4, 120.3, 119.5, 119.0, 104.7.

HRMS (ES+) Calcd for $C_{25}H_{17}NNaO_4^+$ (M + Na$^+$): 418.1050, Found: 418.1053.

4. Synthesis of the Ketone 4a

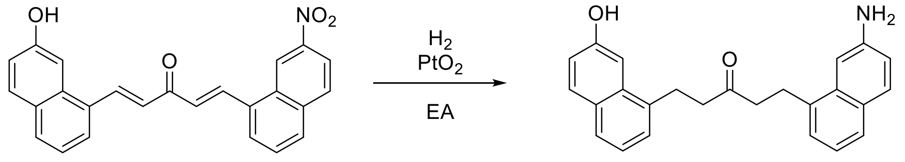

12 4a

1-(7-Aminonaphthalen-1-yl)-5-(7-hydroxynaphthalen-1-yl)pentan-3-one (4a). At room temperature, a flask charged with the dienone **12** (395 mg, 1.0 mmol), EtOAc (10 mL), PtO_2 (40.0 mg, 10 wt%) was evacuated and refilled with hydrogen gas using balloon. The mixture was stirred for 24 h and PtO_2 was recycled by centrifugation and decanting. The resulting solution was concentrated and the crude product was purified by silica gel flash chromatography (eluent: hexanes/EtOAc = 5:1 → 3:1) to afford the ketone **4a** as a pink foam in 70% yield (258 mg).

^{1}H NMR (400 MHz, methanol-*d*$_4$) δ 7.70 – 7.59 (m, 1H), 7.58 – 7.49 (m, 2H), 7.49 – 7.39 (m, 1H), 7.25 – 7.18 (m, 1H), 7.12 – 6.89 (m, 7H), 3.19 – 3.00 (m, 4H), 2.73 – 2.59 (m, 4H).

^{13}**C NMR** (101 MHz, methanol-d_4) δ 212.4, 156.6, 146.8, 136.3, 135.6, 134.4, 134.4, 131.5, 130.9, 130.1, 129.5, 127.62, 127.60, 127.3, 127.1, 123.6, 122.7, 119.2, 118.7, 106.2, 105.7, 44.09, 44.06, 28.0, 27.9.

HRMS (ES-) Calcd for $C_{25}H_{22}NO_2^-$ (M - H$^+$): 368.1656, Found: 368.1649.

5. Synthesis of the NOSPHEN 2a

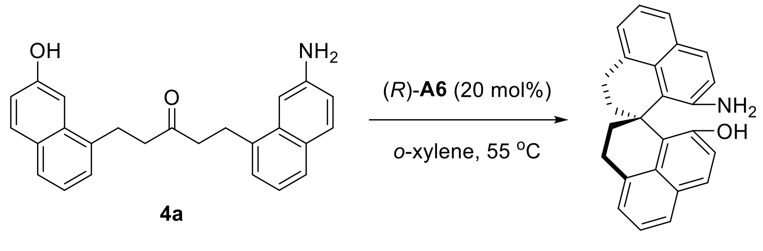

(*S*)-9'-Amino-2,2',3,3'-tetrahydro-1,1'-spirobi[phenalen]-9-ol (2a). At room temperature, to a solution of **4a** (185 mg, 0.5 mmol) in *o*-xylene (10 mL) was added (*R*)-**A6** (116 mg, 0.1 mmol). The mixture was stirred at 55 °C for 24 h before it was cooled to room temperature and evaporated. The crude product was directly subjected to silica gel flash chromatography (eluent: hexanes/DCM = 1:1) to afford the product (*S*)-**2a** as a white solid in 76% yield (133 mg, 98% ee).

$[\alpha]_D^{20}$: -339.6 (c = 0.6, CH_2Cl_2). HPLC analysis of the product: Daicel CHIRALPAK AD-H column; 20% *i*-PrOH in hexanes; 1.0 mL/min; retention times: 12.0 min (minor), 15.9 min (major).

^{1}**H NMR** (400 MHz, $CDCl_3$) δ 7.73 – 7.49 (m, 4H), 7.33 – 7.17 (m, 4H), 7.02 (d, J = 8.8 Hz, 1H), 6.76 (d, J = 8.7 Hz, 1H), 5.33 (s, 1H), 3.58 (s, 2H), 3.36 – 3.19 (m, 2H), 3.12 – 2.94 (m, 2H), 2.51 – 2.38 (m, 2H), 2.36 – 2.14 (m, 2H).

^{13}**C NMR** (101 MHz, $CDCl_3$) δ 151.4, 141.6, 133.3, 132.9, 130.5, 129.9, 129.7, 129.6, 129.0, 128.9, 126.7, 126.6, 125.7, 125.3, 123.1, 122.2, 120.3, 119.5, 118.6, 113.9, 39.5, 30.8, 27.4, 26.3, 26.2.

HRMS (ES-) Calcd for $C_{25}H_{20}NO^-$ (M - H$^+$): 350.1550, Found: 350.1544.

V. Preparation of Diphenyl NOSPHEN

1. Synthesis of the Chiral Diaryl Ketone S12

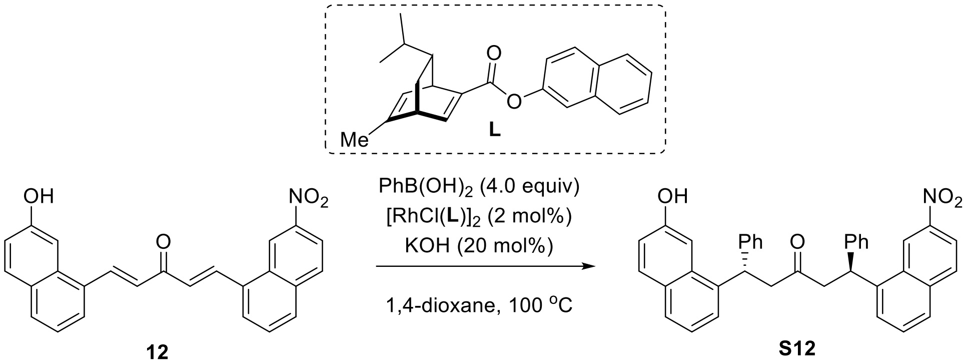

(1*S*,5*S*)-1-(7-Hydroxynaphthalen-1-yl)-5-(7-nitronaphthalen-1-yl)-1,5-diphenylpentan-3-one (S12). At room temperature, a 25-mL round-bottom flask equipped with a stir bar was charged with **12** (1.19 g, 3.0 mmol), [RhCl(**L**)]$_2$ (56.4 mg, 0.06 mmol), an aqueous solution of KOH (1.0 M, 0.6 mL), phenyl boronic acid (1.46 g, 12 mmol), and 1,4-dioxane (30 mL). The flask was evacuated and refilled with N_2 for 5 times. Then, the mixture was stirred under N_2 at 100 °C for 36 h. After cooling to room temperature, the mixture was concentrated and the residue was purified by silica gel flash chromatography to afford the product **S12** as a yellow foam in 92% yield (1.52 g, >99% ee).

$[\alpha]_D^{20}$: -16.8 (c = 1.0, CH_2Cl_2). HPLC analysis of the product: Daicel CHIRALCEL OD-3 column; 30% *i*-PrOH in hexanes; 1.0 mL/min; retention times: 11.9 min (minor), 37.4 min (major).

^{1}H NMR (400 MHz, $CDCl_3$) δ 9.07 (s, 1H), 8.12 (dd, J = 9.0, 2.2 Hz, 1H), 7.85 (d, J = 9.0 Hz, 1H), 7.73 (d, J = 8.2 Hz, 1H), 7.67 (d, J = 8.8 Hz, 1H), 7.62 (dd, J = 7.0, 2.3 Hz, 1H), 7.46 (t, J = 7.8 Hz, 1H), 7.37 (d, J = 2.4 Hz, 1H), 7.25 – 7.09 (m, 12H), 7.00 (dd, J = 8.8, 2.4 Hz, 1H), 6.07 (s, 1H), 5.36 (t, J = 7.3 Hz, 1H), 5.22 (t, J = 7.4 Hz, 1H), 3.42 – 3.08 (m, 4H).

^{13}C NMR (101 MHz, $CDCl_3$) δ 207.1, 154.0, 145.4, 143.1, 143.0, 141.4, 137.1, 136.6, 132.6, 130.7, 130.3, 130.2, 129.3, 129.1, 128.8, 128.6, 127.9, 127.6, 127.2, 127.1, 126.8, 126.5, 125.5, 124.7, 122.7, 120.9, 118.9, 117.5, 105.9, 49.9, 49.7, 41.7, 41.3.

HRMS (ES+) Calcd for $C_{37}H_{29}NNaO_4^+$ (M + Na+): 574.1989, Found: 574.1998.

2. Synthesis of the Chiral Diaryl Ketone 4b

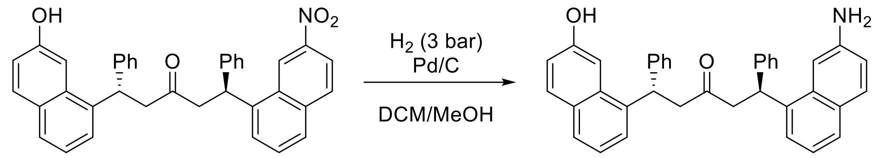

(1*S*,5*S*)-1-(7-Aminonaphthalen-1-yl)-5-(7-hydroxynaphthalen-1-yl)-1,5-diphenylpentan-3-one (4b). To a solution of **S12** (>99% ee, 1.10 g, 2.0 mmol) in DCM (5.0 mL) was added the Pd/C (110 mg, 10 wt%). The mixture was transferred into a Parr® autoclave, which was valuated and refilled with hydrogen gas for three times and finally pressurized to 3 bar. The mixture was stirred at room temperature for 5 h, and the hydrogen gas was released carefully in a fume hood. The mixture was filtered through a pad of celite and the filter cake was washed with EtOAc (20 mL × 3). The filtrate was concentrated, and the crude product was purified by silica gel flash chromatography (eluent: hexanes/EtOAc = 3:1) to afford the ketone **4b** as a yellow foam in 93% yield (970 mg).

$[\alpha]_D^{20}$: -34.1 (c = 1.0, CH_2Cl_2).

^{1}H NMR (400 MHz, $CDCl_3$) δ 7.71 – 7.50 (m, 4H), 7.34 – 7.30 (m, 1H), 7.24 – 6.97 (m, 16H), 6.89 – 6.84 (m, 1H), 5.26 – 5.09 (m, 2H), 3.90 (s, 2H), 3.30 – 3.08 (m, 4H).

^{13}C NMR (101 MHz, $CDCl_3$) δ 208.2, 154.1, 144.2, 143.4, 143.2, 137.5, 136.9, 132.7, 132.6, 130.6, 130.2, 129.2, 128.5, 128.5, 127.9, 127.0, 127.0, 126.3, 126.3, 125.0, 124.8, 122.7, 121.9, 117.9, 117.6, 105.9, 105.3, 49.8, 49.6, 41.2, 41.2.

HRMS (ES+) Calcd for $C_{37}H_{31}NNaO_2^+$ (M + Na+): 544.2250, Found: 544.2247.

3. Synthesis of the Diphenyl NOSPHEN 2b

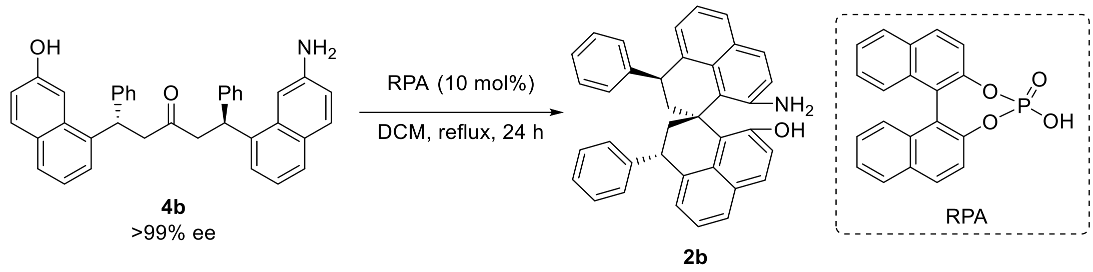

(1*S*,3*S*,3'*S*)-9'-Amino-3,3'-diphenyl-2,2',3,3'-tetrahydro-1,1'-spirobi[phenalen]-9-ol (2b). At room temperature, to a solution of **4b** (>99% ee, 1.19 g, 2.28 mmol) in DCM (9.0 mL) was added RPA (79.3 mg, 0.23 mmol, 10 mol%). The mixture was refluxed for 24 h. The reaction progress was monitored by thin layer chromatography. Upon completion, the mixture was cooled to room temperature, evaporated and subjected to silica gel flash chromatography (eluent: hexanes/DCM = 1:1) to afford the product **2b** as a yellow foam in 76% yield (870 mg).

$[\alpha]_D^{20}$: +188.9 (c = 1.0, CH_2Cl_2).

^{1}H NMR (400 MHz, $CDCl_3$) δ 7.81 – 7.74 (m, 2H), 7.70 (d, J = 8.7 Hz, 1H), 7.61 (d, J = 8.1 Hz, 1H), 7.43 – 7.35 (m, 1H), 7.34 – 7.28 (m, 1H), 7.16 – 7.03 (m, 8H), 6.96 – 6.84 (m, 3H), 6.74 – 6.60 (m, 3H), 6.04 (s, 1H), 4.83 (dd, J = 7.0, 2.7 Hz, 1H), 4.25 (dd, J = 10.9, 4.0 Hz, 1H), 3.84 (s, 2H), 3.64 (dd, J = 14.0, 6.9 Hz, 1H), 2.46 (dd, J = 13.5, 4.1 Hz, 1H), 2.29 (dd, J = 14.0, 2.9 Hz, 1H), 2.14 – 1.98 (m, 1H).

^{13}C NMR (101 MHz, $CDCl_3$) δ 152.5, 147.4, 143.8, 142.9, 137.4, 134.1, 130.7, 130.5, 130.2, 130.0, 129.5, 128.9, 128.6, 128.1, 128.0, 127.4, 126.6, 126.1, 125.5, 125.3, 123.3, 122.4, 120.0, 119.4, 118.9, 114.4, 46.8, 43.2, 42.4, 41.4, 40.1.

HRMS (ES-) Calcd for $C_{37}H_{28}NO^-$ (M - H^+): 502.2176, Found: 502.2163.

VI. Synthesis of SPHENAM Derivatives

1. Synthesis of the Diphosphine L1

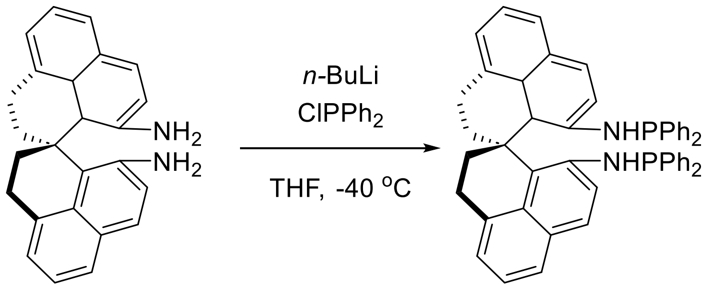

(*S*)-**1a** **L1**

(1*S*)-N^9,$N^{9'}$-Bis(diphenylphosphaneyl)-2,2',3,3a1,3',9a-hexahydro-1,1'-spirobi[phenalene]-9,9'-diamine (L1). At -40 °C under N_2, to a solution of (*S*)-**1a** (>99% ee, 105 mg, 0.3 mmol) in freshly distilled THF (10 mL) was added ^{n}BuLi (2.4 M in hexanes, 0.3 mL, 0.72 mmol) dropwise. After stirring for 2 h, $ClPPh_2$ (177 mg, 0.8 mmol) was added dropwise. The reaction mixture was slowly warmed to room temperature and stirred for 2 h before it was quenched with a saturated aqueous solution of NH_4Cl (20 mL). The resulting mixture was extracted with Et_2O (15 mL × 3), and the combined organic layers were dried over Na_2SO_4, and concentrated. The residue was purified by silica gel flash chromatography (eluent: hexanes/DCM = 10:1) to afford the desired product **L1** as a white foam in 72% yield (155 mg).

$[\alpha]_D^{20}$: -183.4 (c = 0.7, CH_2Cl_2).

^{1}H NMR (400 MHz, $CDCl_3$) δ 7.67 – 7.49 (m, 6H), 7.32 – 7.07 (m, 12H), 7.04 – 6.87 (m, 8H), 6.78 – 6.61 (m, 4H), 4.62 (d, J = 8.5 Hz, 2H), 3.27 – 3.09 (m, 2H), 2.90 – 2.65 (m, 2H), 2.33 – 2.21 (m, 2H), 2.19 – 2.03 (m, 2H).

^{13}C NMR (101 MHz, $CDCl_3$) δ 141.4, 141.2, 139.9, 139.8, 139.8, 139.7, 132.7, 131.1, 130.9, 130.3, 130.1, 129.8, 129.1, 128.8, 128.48, 128.46, 128.34, 128.32, 128.26, 128.16, 128.1, 126.6, 125.2, 122.3, 120.22, 120.19, 118.2, 118.0, 40.1, 28.7, 26.2.

^{31}P NMR (162 MHz, $CDCl_3$) δ 26.1.

HRMS (ES-) Calcd for $C_{49}H_{41}N_2P_2$ (M - H^+): 719.2750, Found: 719.2745.

2. Synthesis of the Diphosphine Ph_2-L1

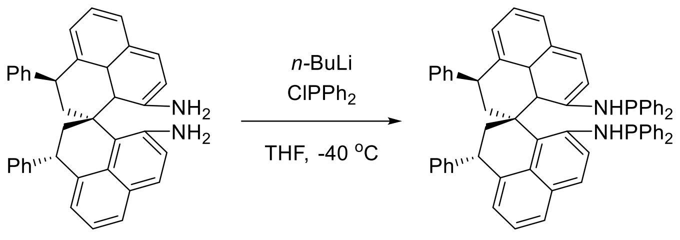

(1*S*)-*N*⁹,*N*⁹'-Bis(diphenylphosphaneyl)-3,3'-diphenyl-2,2',3,3a1,3',9a-hexahydro-1,1'-spirobi[phenalene]-9,9'-diamine (Ph_2-L1). At -40 °C under N_2, to a solution of **9a** (>99% ee, 251 mg, 0.5 mmol) in freshly distilled THF (15 mL) was added ^{n}BuLi (1.6 M in hexanes, 0.75 mL, 1.2 mmol) dropwise. After stirring for 2 h, $ClPPh_2$ (287 mg, 1.3 mmol) was added dropwise. The reaction mixture was slowly warmed to room temperature and stirred for 2 h before it was quenched with a saturated aqueous solution of NH_4Cl (20 mL). The resulting mixture was extracted with Et_2O (15 mL × 3), and the combined organic layers were dried over Na_2SO_4, and concentrated. The residue was purified by silica gel flash chromatography (eluent: hexanes/DCM = 10:1) to afford the desired product **Ph_2-L1** as a white foam in 65% yield (284 mg).

$[\alpha]_D^{20}$: -19.3 (c = 2.3, $CHCl_3$).

^{1}H NMR (400 MHz, $CDCl_3$) δ 7.81 – 7.74 (m, 2H), 7.67 – 7.62 (m, 2H), 7.55 (d, J = 8.0 Hz, 2H), 7.20 – 6.99 (m, 20H), 6.88 – 6.78 (m, 8H), 6.71 (d, J = 7.0 Hz, 2H), 6.52 (d, J = 6.5 Hz, 4H), 5.13 (d, J = 8.5 Hz, 2H), 4.62 (d, J = 8.5 Hz, 2H), 4.16 (t, J = 5.8 Hz, 2H), 2.67 (dd, J = 13.8, 5.8 Hz, 2H), 2.25 (dd, J = 13.8, 6.2 Hz, 2H).

^{13}C NMR (101 MHz, $CDCl_3$) δ 146.0, 142.5, 142.3, 139.5, 139.44, 139.39, 139.3, 134.9, 131.5, 131.3, 130.3, 130.2, 130.1, 129.3, 129.0, 128.93, 128.90, 128.60, 128.55, 128.46, 128.4, 128.2, 128.1, 127.9, 127.2, 127.0, 125.4, 122.4, 119.75, 119.72, 118.2, 117.9, 43.2, 43.0, 41.5.

^{31}P NMR (162 MHz, $CDCl_3$) δ 25.1.

HRMS (ES+) Calcd for $C_{61}H_{51}N_2P_2$ (M + H^+): 873.3522, Found: 873.3434.

3. Synthesis of the Urea L2

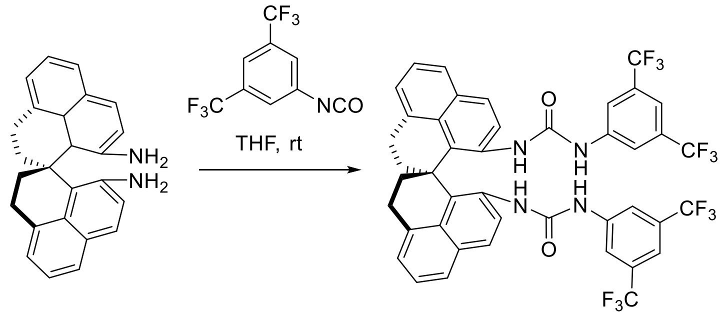

(*S*)-1,1'-(2,2',3,3'-Tetrahydro-1,1'-spirobi[phenalene]-9,9'-diyl)bis(3-(3,5-bis(trifluoromethyl)phenyl)urea) (L2). At room temperature, to a solution of (*S*)-**1a** (>99% ee, 35.0 mg, 0.1 mmol) in anhydrous DCM (2.0 mL) was added 3,5-bis(trifluoromethyl)phenyl isocyanate (76.5 mg, 0.3 mmol). The mixture was stirred at room temperature for 12 h and directly subjected to silica gel flash chromatography (eluent: hexanes/EtOAc = 3:1) to afford the urea **L2** as a white solid in 99% yield (86.1 mg).

$[\alpha]_D^{20}$: -46.3 (c = 1.0, CH_2Cl_2).

^{1}H NMR (400 MHz, $CDCl_3$) δ 7.48 – 7.41 (m, 4H), 7.38 (d, J = 8.7 Hz, 2H), 7.33 – 7.25 (m, 6H), 7.23 – 7.17 (m, 2H), 7.09 (d, J = 8.6 Hz, 2H), 6.94 (s, 2H), 5.82 (s, 2H), 3.21 – 3.02 (m, 2H), 2.99 – 2.81 (m, 2H), 2.43 – 2.30 (m, 2H), 2.05 – 1.93 (m, 2H).

^{13}C NMR (101 MHz, $CDCl_3$) δ 152.9, 139.6, 137.2, 134.1, 132.3, 131.7 (q, J = 33.1 Hz), 129.6, 129.5, 127.3, 127.1, 126.5, 125.8, 125.0, 123.1 (q, J = 271.0 Hz), 118.9, 116.0, 41.4, 32.1, 26.2.

^{19}F NMR (376 MHz, $CDCl_3$) δ -63.2.

HRMS (ES+) Calcd for $C_{43}H_{28}F_{12}N_4NaO_2$ (M + Na^+): 883.1913, Found: 883.1917.

4. Synthesis of the Thiourea L3

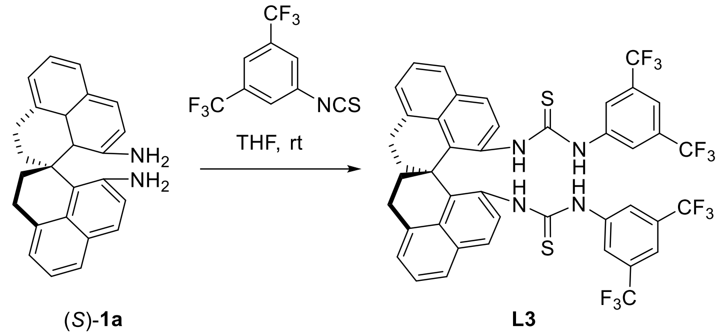

(*S*)-1,1'-(2,2',3,3'-Tetrahydro-1,1'-spirobi[phenalene]-9,9'-diyl)bis(3-(3,5-bis(trifluoromethyl)phenyl)thiourea) (L3). At room temperature, to a solution of (*S*)-**1a** (>99% ee, 35.0 mg, 0.1 mmol) in anhydrous DCM (2.0 mL) was added 3,5-bis(trifluoromethyl)phenyl isothiocyanate (81.0 mg, 0.3 mmol). The mixture was stirred at room temperature for 12 h and directly subjected to silica gel flash chromatography (eluent: hexanes/EtOAc = 3:1) to afford the thiourea **L3** as a pale-yellow solid in 95% yield (85.2 mg).

$[\alpha]_D^{20}$: -327.9 (c = 1.0, CH_2Cl_2).

^{1}H NMR (400 MHz, DMSO-d_6) δ 9.47 (s, 2H), 7.94 – 7.30 (m, 16H), 7.30 – 7.09 (m, 2H), 3.38 – 3.22 (m, 2H), 3.17 – 2.98 (m, 2H), 2.55 – 2.39 (m, 4H).

^{13}C NMR (101 MHz, DMSO-d_6) δ 179.2, 140.4, 138.5, 135.0, 132.7, 130.8, 130.2 (q, J = 32.0 Hz), 129.2, 125.8, 125.7, 125.6, 124.6, 123.2 (q, J = 272.0 Hz), 123.1, 117.2, 41.5, 30.7, 26.0.

^{19}F NMR (377 MHz, DMSO-d_6) δ -61.5.

HRMS (ES+) Calcd for $C_{43}H_{28}F_{12}N_4NaS_2$ (M + Na^+): 915.1456, Found: 915.1459.

5. Synthesis of the Diamide L4

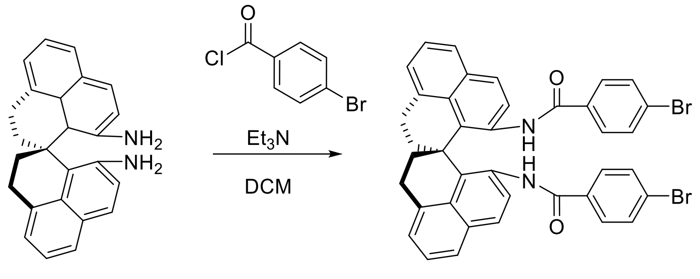

(*S*)-**1a** L4

(*S*)-*N*,*N'*-(2,2',3,3'-Tetrahydro-1,1'-spirobi[phenalene]-9,9'-diyl)bis(4-bromobenzamide) (L4). At room temperature, to a solution of (*S*)-**1a** (>99% ee, 35.0 mg, 0.1 mmol) and Et_3N (101 mg, 1.0 mmol) in anhydrous DCM (2 mL) was added 4-bromobenzoyl chloride (66.0 mg, 0.3 mmol). After stirring for 24 h, the reaction mixture was directly subjected to silica gel flash chromatography (eluent: hexanes/EtOAc = 3:1) to afford the desired product **L4** as a white solid 98% yield (70.2 mg).

$[\alpha]_D^{20}$: -176.4 (c = 1.0, CH_2Cl_2).

^{1}H NMR (400 MHz, $CDCl_3$) δ 8.03 – 7.85 (m, 2H), 7.85 – 7.69 (m, 4H), 7.52 – 7.38 (m, 2H), 7.34 – 7.28 (m, 2H), 7.12 (d, J = 8.1 Hz, 4H), 6.54 (d, J = 8.1 Hz, 4H), 3.43 – 3.20 (m, 2H), 3.20 – 2.95 (m, 2H), 2.67 – 2.46 (m, 2H), 2.36 – 2.08 (m, 2H).

^{13}C NMR (101 MHz, $CDCl_3$) δ 164.4, 134.1, 132.7, 132.0, 131.4, 131.2, 131.2, 129.0, 128.0, 127.8, 126.8, 126.1, 125.8, 125.7, 125.2, 41.7, 31.2, 26.2.

HRMS (ES+) Calcd for $C_{39}H_{28}Br_2N_2NaO_2$ (M + Na^+): 737.0410, Found: 737.0414.

VII. Synthesis of NOSPHEN and NOBIN Derivatives

1. Synthesis of the Phosphite L5

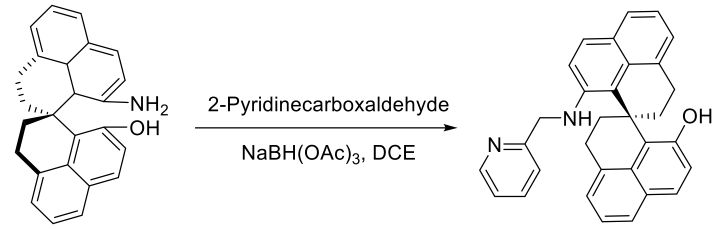

(*S*)-**2a** **S2a-1**

(*S*)-9'-((Pyridin-2-ylmethyl)amino)-2,2',3,3'-tetrahydro-1,1'-spirobi[phenalen]-9-ol (S2a-1). At room temperature, to a solution of (*S*)-**2a** (>99% ee, 70.2 mg, 0.2 mmol) in anhydrous DCE (2.0 mL) were added sodium triacetoxyborohydride (126 mg, 0.6 mmol) and 2-pyridinecarboxaldehyde (32.0 mg, 0.3 mmol). The mixture was stirred for 12 h before it was directly subjected to silica gel flash chromatography (eluent: hexanes/EtOAc = 2:1) to afford the product **S2a-1** as a yellow foam in 96% yield (84.7 mg).

$[\alpha]_D^{20}$: -150.0 (c = 0.5, CH_2Cl_2).

^{1}H NMR (400 MHz, $CDCl_3$) δ 8.10 – 7.98 (m, 1H), 7.76 – 7.59 (m, 4H), 7.49 – 7.40 (m, 1H), 7.38 – 7.31 (m, 2H), 7.31 – 7.19 (m, 2H), 7.06 – 6.92 (m, 3H), 6.86 (d, J = 7.9 Hz, 1H), 5.64 (s, 1H), 4.75 (s, 1H), 4.38 – 4.13 (m, 2H), 3.50 – 3.25 (m, 2H), 3.18 – 2.97 (m, 2H), 2.69 – 2.49 (m, 2H), 2.49 – 2.21 (m, 2H).

^{13}C NMR (101 MHz, $CDCl_3$) δ 157.7, 151.4, 148.6, 142.7, 136.3, 133.2, 132.7, 130.6, 130.0, 129.94, 129.90, 128.7, 128.2, 126.7, 126.5, 125.6, 125.2, 122.9, 121.7, 121.6, 120.6, 120.4, 118.6, 114.4, 114.1, 49.3, 39.6, 31.2, 27.6, 26.4, 26.2.

HRMS (ES+) Calcd for $C_{31}H_{27}N_2O^+$ (M + H^+): 443.2118, Found: 443.2124.

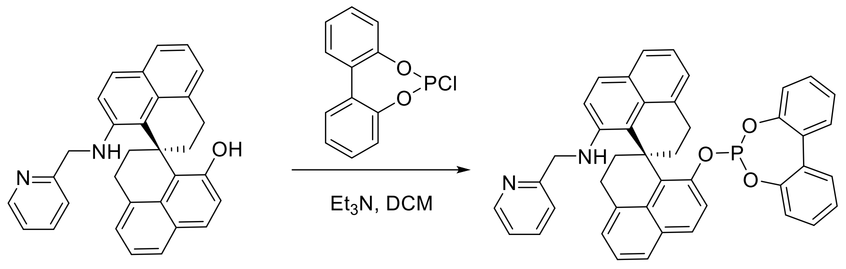

S2a-1 **L5**

(*S*)-9'-(Dibenzo[d,f][1,3,2]dioxaphosphepin-6-yloxy)-N-(pyridin-2-ylmethyl)-2,2',3,3'-tetrahydro-1,1'-spirobi[phenalen]-9-amine (L5). To a solution of S**2a-1** (44.0 mg, 0.1 mmol) in anhydrous DCM (2.0 mL) were added triethylamine (101 mg, 1.0 mmol) and a solution of phosphorochloridite in DCM (1.0 M, 0.5 mL). The mixture was stirred at room temperature for 2 h before it was directly subjected to silica gel flash chromatography (eluent: hexanes/EtOAc = 2:1) to afford the product **L5** as a yellow foam in 67% yield (43.7 mg).

$[\alpha]_D^{20}$: -160.2 (c = 0.6, CH_2Cl_2).

^{1}H NMR (400 MHz, $CDCl_3$) δ 7.98 (d, J = 4.9 Hz, 1H), 7.76 – 7.59 (m, 4H), 7.45 – 7.27 (m, 8H), 7.25 – 6.88 (m, 8H), 6.81 – 6.70 (m, 1H), 5.48 (d, J = 8.1 Hz, 1H), 4.32 – 4.07 (m, 2H), 3.50 – 3.30 (m, 2H), 3.18 – 3.04 (m, 2H), 2.69 – 2.39 (m, 4H).

^{13}C NMR (101 MHz, $CDCl_3$) δ 158.0, 148.5, 140.2, 136.0, 135.2, 133.2, 131.9, 130.8, 130.5, 130.2, 129.8, 129.7, 129.3, 128.9, 128.74, 128.67, 128.2, 127.9, 126.8, 126.5, 125.2, 124.7, 124.54, 124.48, 122.1, 122.0, 121.4, 121.2, 120.8, 115.2, 49.5, 40.7, 30.5, 28.7, 26.8, 26.5.

^{31}P NMR (162 MHz, $CDCl_3$) δ 142.4.

HRMS (ES+) Calcd for $C_{43}H_{34}N_2O_3P^+$ (M + H^+): 657.2302, Found: 657.2304.

2. Synthesis of the Phosphite L5′

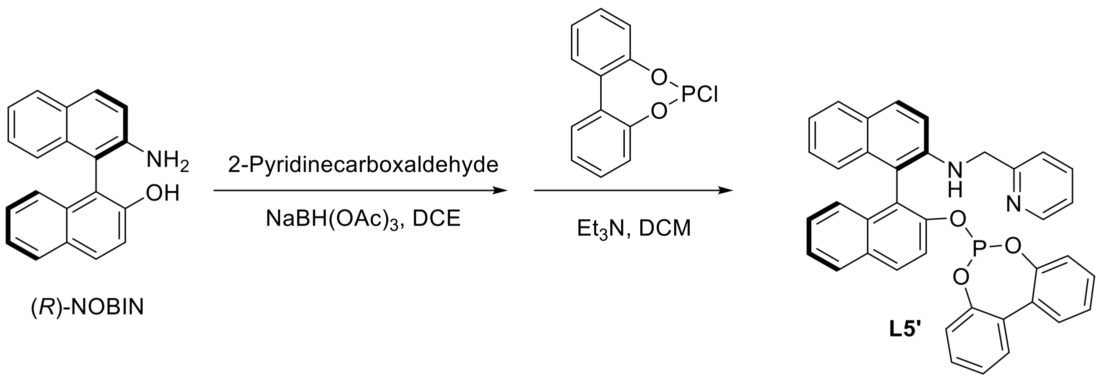

2'-(Dibenzo[d,f][1,3,2]dioxaphosphepin-6-yloxy)-N-(pyridin-2-ylmethyl)-[1,1'-binaphthalen]-2-amine (L5′). At room temperature, to a solution of (*R*)-NOBIN (>99% ee, 228 mg, 0.8 mmol) in anhydrous DCE (8.0 mL) were added sodium triacetoxyborohydride (509 mg, 2.4 mmol) and 2-pyridinecarboxaldehyde (129 mg, 1.2 mmol). The mixture was stirred for 12 h before it was directly subjected to silica gel flash chromatography (eluent: hexanes/EtOAc = 2:1) to afford the intermediate as a white solid in 92% yield (277 mg).

To a solution of the above intermediate (113 mg, 0.3 mmol) in anhydrous DCM (6.0 mL) were added triethylamine (304 mg, 3.0 mmol) and a solution of phosphorochloridite in DCM (1.0 M, 1.5 mL). The mixture was stirred at room temperature for 2 h before it was directly subjected to silica gel flash chromatography (eluent: hexanes/EtOAc = 2:1) to afford the product **L5′** as a white solid in 52% yield (92.1 mg).

$[\alpha]_D^{20}$: +42.2 (c = 2.3, $CHCl_3$).

^{1}H NMR (400 MHz, $CDCl_3$) δ 8.42 – 8.37 (d, J = 4.7 Hz, 1H), 8.06 – 8.01 (d, J = 8.8 Hz, 1H), 7.99 – 7.95 (d, J = 8.2 Hz, 1H), 7.91 – 7.86 (d, J = 9.0 Hz, 1H), 7.86 – 7.81 (d, J = 8.4 Hz, 1H), 7.62 – 7.56 (d, J = 8.8 Hz, 1H), 7.52 – 7.45 (m, 1H), 7.42 – 7.32 (m, 4H), 7.26 – 7.11 (m, 8H), 7.09 – 7.04 (d, J = 9.0 Hz, 1H), 7.04 – 6.94 (m, 3H), 6.53 – 6.47 (d, J = 7.5 Hz, 1H), 6.43 – 6.37 (d, J = 7.4 Hz, 1H), 4.60 – 4.40 (m, 2H).

^{13}**C NMR** (101 MHz, $CDCl_3$) δ 159.4, 148.54, 148.50, 143.9, 137.0, 134.4, 133.7, 131.6, 130.3, 129.9, 129.5,129.4, 129.0, 128.9, 128.3, 128.0, 127.5, 127.2, 126.7, 125.9, 125.5, 125.1, 124.3, 123.4, 122.1, 122.0, 121.9, 121.8, 121.1, 117.0, 113.7, 112.0, 48.9.

^{31}**P NMR** (162 MHz, $CDCl_3$) δ 144.3.

HRMS (ES+) Calcd for $C_{38}H_{28}N_2O_3P^+$ (M + H^+): 591.1832, Found: 591.1937.

3. Synthesis of the Thiourea-Diphenylphosphine L6

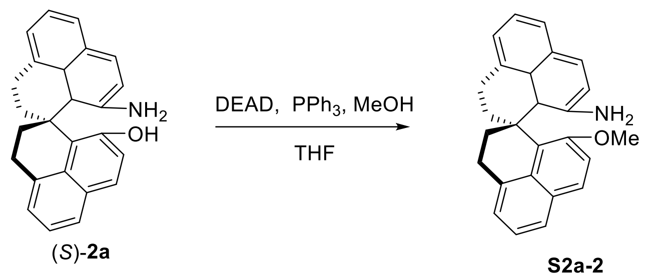

(1*S*)-9'-Methoxy-2,2',3,3a1,3',9a-hexahydro-1,1'-spirobi[phenalen]-9-amine (S2a-2). To a solution of (*S*)-**2a** (>99% ee, 351 mg, 1.0 mmol) in anhydrous THF (5.0 mL) were added PPh_3 (524 mg, 2.0 mmol), MeOH (64.0 mg, 2.0 mmol), and diethyl diazodicarboxylate (348 mg, 2.0 mmol). The mixture was stirred at room temperature for 24 h. Next, the solvent was evaporated, and the residue was purified by silica gel flash chromatography (eluent: hexanes/DCM = 2:1) to afford **S2a-2** as a white solid in 97% yield (354 mg).

$[\alpha]_D^{20}$: -155.0 (c = 0.5, CH_2Cl_2).

^{1}**H NMR** (400 MHz, $CDCl_3$) δ 7.74 (d, J = 8.9 Hz, 1H), 7.66 (d, J = 8.1 Hz, 1H), 7.60 – 7.53 (m, 1H), 7.49 (d, J = 8.6 Hz, 1H), 7.33 – 7.24 (m, 2H), 7.21 – 7.11 (m, 3H), 6.68 (d, J = 8.5 Hz, 1H), 3.37 – 3.23 (m, 2H), 3.21 (s, 3H), 3.07 – 2.92 (m, 4H), 2.54 – 2.29 (m, 4H).

^{13}**C NMR** (101 MHz, $CDCl_3$) δ 154.1, 138.3, 134.5, 133.5, 130.7, 130.0, 129.8, 128.7, 128.3, 126.8, 126.5, 126.3, 126.2, 124.9, 124.2, 123.4, 122.9, 121.3, 119.7, 115.0, 56.4, 40.3, 30.6, 26.8, 26.5.

HRMS (ES-) Calcd for $C_{26}H_{22}NO^-$ (M - H^+): 365.1774, Found: 365.1774.

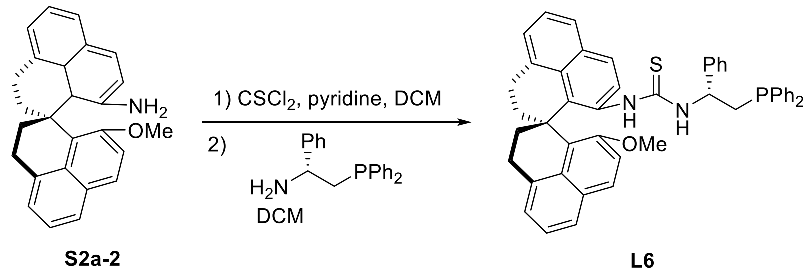

1-((*R*)-2-(Diphenylphosphaneyl)-1-phenylethyl)-3-((*S*)-9'-methoxy-2,2',3,3'-tetrahydro-1,1'-spirobi[phenalen]-9-yl)thiourea (L6). To a solution of **S2a-2** (76.0 mg, 0.2 mmol) in anhydrous DCM (2.0 mL) were added pyridine (63.2 mg, 0.8 mmol) and $CSCl_2$ (27.6 mg, 0.24 mmol). The mixture was stirred at room temperature for 2 h. Next, the solvent was evaporated, and the residue was purified by silica gel flash chromatography (eluent: hexanes/DCM = 2:1) to afford the isothiocyanate intermediate as a white solid.

To a solution of the above isothiocyanate in anhydrous DCM (2.0 mL) were added pyridine (63.2 mg, 0.8 mmol) and (*R*)-2-(diphenylphosphino)-1-phenylethanamine (91.6 mg, 0.3 mmol). The mixture was stirred at room temperature for 12 h before it was directly subjected to silica gel flash chromatography (eluent: hexanes/EtOAc = 2:1) to afford the thiourea **L6** as a yellow solid in 70% yield over 2 steps (100 mg).

$[\alpha]_D^{20}$: -370.9 (c = 0.5, CH_2Cl_2).

^{1}H NMR (400 MHz, $CDCl_3$) δ 7.88 – 7.80 (m, 1H), 7.78 – 7.66 (m, 3H), 7.50 – 7.27 (m, 14H), 7.24 – 7.14 (m, 5H), 7.04 – 6.90 (m, 1H), 6.71 (s, 1H), 5.72 (s, 1H), 3.38 – 3.21 (m, 2H), 3.18 (s, 3H), 3.13 – 3.04 (m, 1H), 2.99 – 2.87 (m, 2H), 2.78 – 2.46 (m, 2H), 2.45 – 2.27 (m, 2H), 2.07 – 1.93 (m, 2H).

^{13}C NMR (101 MHz, $CDCl_3$) δ 179.2, 152.1, 141.1, 140.0, 138.3 (d, J = 13.1 Hz), 137.6 (d, J = 12.3 Hz), 135.5, 133.8, 130.5, 130.0, 129.6, 128.9, 128.60, 128.55, 128.43, 128.38, 128.3, 127.5, 126.7, 126.6, 126.5, 126.4, 126.3, 125.9, 125.4, 125.2, 124.7, 124.0, 114.1, 56.4, 56.2, 55.9, 41.2, 36.2 (d, J = 15.6 Hz), 30.4 (d, J = 13.8 Hz), 26.5 (d, J = 23.6 Hz).

^{31}P NMR (162 MHz, $CDCl_3$) δ -24.3.

HRMS (ES-) Calcd for $C_{47}H_{42}OPS^-$ (M - H^+): 711.2604, Found: 711.2599.

VIII. Application of SPHENAM and NOSPHEN Derivatives

1. Enantioselective Hydrogenation of the Enamide 13

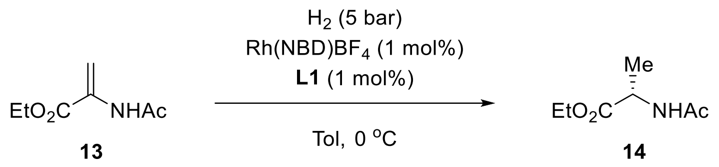

Ethyl acetyl-*L*-alaninate (14). Under nitrogen, a mixture of $Rh(NBD)_2BF_4$ (7.4 mg, 0.02 mmol), **L1** (14.4 mg, 0.02 mmol), and anhydrous DCM (2.0 mL) was stirred 30 min. Then, an aliquot of this solution of chiral rhodium complex (0.2 mL) was added to a solution of the dehydroamino ester **13** (29.0 mg, 0.2 mmol) in anhydrous toluene (2.0 mL). The reaction mixture was transferred into the autoclave. The autoclave was evacuated and refilled with H_2 gas (< 5 bar) for three times and finally pressurized at 5 bar. The mixture was stirred at room temperature for 12 h before it was concentrated *in vacuo*. The residue was purified by silica gel column chromatography to afford the hydrogenated product **31** as a colorless oil in 98% yield (28.3 mg, 92% ee).

$[\alpha]_D^{20}$: -4.0 (c = 1.0, CH_2Cl_2). HPLC analysis of the product: Daicel CHIRALCEL OD-H column; 5% *i*-PrOH in hexanes; 1.0 mL/min; retention times: 12.5 min (major), 15.8 min (minor).

^{1}H NMR (400 MHz, $CDCl_3$) δ 6.16 (s, 1H), 4.72 – 4.47 (m, 1H), 4.19 (q, J = 7.1 Hz, 2H), 2.00 (s, 3H), 1.38 (d, J = 7.2 Hz, 3H), 1.27 (t, J = 7.2 Hz, 3H).

^{13}C NMR (101 MHz, $CDCl_3$) δ 173.2, 169.5, 61.5, 48.1, 23.1, 18.6, 14.1.

HRMS (ES+) Calcd for $C_7H_{13}NNaO_3$ (M + Na^+): 182.0788, Found: 182.0788.

This is a known compound. The characterization data are consistent with the literature.[1]

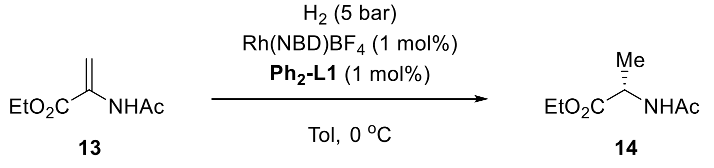

Under nitrogen, a mixture of Rh(NBD)$_2$BF$_4$ (7.4 mg, 0.02 mmol), **Ph$_2$-L1** (17.4 mg, 0.02 mmol), and anhydrous DCM (2.0 mL) was stirred for 30 min. Then, an aliquot of this solution of the chiral rhodium complex (0.2 mL) was added to a solution of the dehydroamino ester **13** (29.0 mg, 0.2 mmol) in anhydrous toluene (2.0 mL). The reaction mixture was transferred into an autoclave. The autoclave was evacuated and refilled with H$_2$ gas (< 5 bar) three times and finally pressurized at 5 bar. The mixture was stirred at room temperature for 12 h before it was concentrated *in vacuo*. The residue was purified by silica gel column chromatography to afford the hydrogenated product **31** as a colorless oil in 61% yield (17.8 mg, 83% ee).

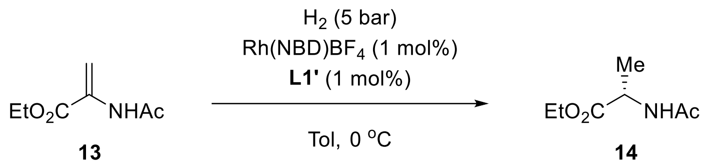

Under nitrogen, a mixture of Rh(NBD)$_2$BF$_4$ (7.4 mg, 0.02 mmol), **L1′** (13.0 mg, 0.02 mmol), and anhydrous DCM (2.0 mL) was stirred for 30 min. Then, an aliquot of this solution of the chiral rhodium complex (0.2 mL) was added to a solution of the dehydroamino ester **13** (29.0 mg, 0.2 mmol) in anhydrous toluene (2.0 mL). The reaction mixture was transferred into an autoclave. The autoclave was evacuated and refilled with H$_2$ gas (< 5 bar) three times and finally pressurized at 5 bar. The mixture was stirred at room temperature for 12 h before it was concentrated *in vacuo*. The residue was purified by silica gel column chromatography to afford the hydrogenated product **31** as a colorless oil in 89% yield (25.8 mg, 87% ee).

2. Enantioselective Hydrogenation of the Enamide 15

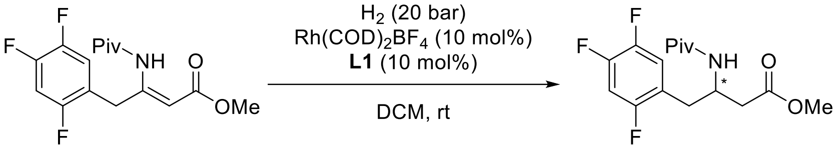

15 **16**

(+)-Methyl 3-pivalamido-4-(2,4,5-trifluorophenyl)butanoate (16). Under nitrogen, a mixture of $Rh(NBD)_2BF_4$ (4.1 mg, 0.01 mmol), **L1** (8.6 mg, 0.012 mmol), and anhydrous DCM (1.0 mL) was stirred for 30 min. Then, to this solution of the chiral rhodium complex was added dehydroamino ester **15** (33.0 mg, 0.1 mmol). The reaction mixture was transferred into an autoclave. The autoclave was evacuated and refilled with H_2 gas (< 5 bar) three times and finally pressurized at 20 bar. The mixture was stirred at room temperature for 12 h and concentrated *in vacuo*. The residue was purified by silica gel column chromatography to afford the hydrogenated product **16** as a colorless oil in 95% yield (31.3 mg, 80% ee).

$[\alpha]_D^{20}$: +15.1 (c = 1.0, CH_2Cl_2). HPLC analysis of the product: Daicel CHIRALCEL OD-H column; 2% *i*-PrOH in hexanes; 1.0 mL/min; retention times: 15.5 min (minor), 16.7 min (major).

^{1}H NMR (400 MHz, $CDCl_3$) δ 7.08 – 6.93 (m, 1H), 6.93 – 6.78 (m, 1H), 4.46 – 4.25 (m, 1H), 3.66 (s, 3H), 2.89 – 2.73 (m, 2H), 2.63 – 2.43 (m, 2H), 1.08 (s, 9H).

^{13}C NMR (101 MHz, $CDCl_3$) δ 177.9, 172.1, 121.3, 121.1, 119.0 (d, J = 6.1 Hz), 118.8 (d, J = 5.6 Hz), 105.3 (d, J = 20.8 Hz), 105.0 (d, J = 20.7 Hz), 51.7, 46.2, 38.5, 37.1, 32.4, 27.2.

^{19}F NMR (376 MHz, $CDCl_3$) δ -119.35 (d, J = 15.5 Hz), -135.54 (d, J = 21.5 Hz), -142.89 (dd, J = 21.5, 15.5 Hz).

HRMS (ES+) Calcd for $C_{16}H_{20}F_3NNaO_3$ (M + Na$^+$): 354.1287, Found: 354.1291.

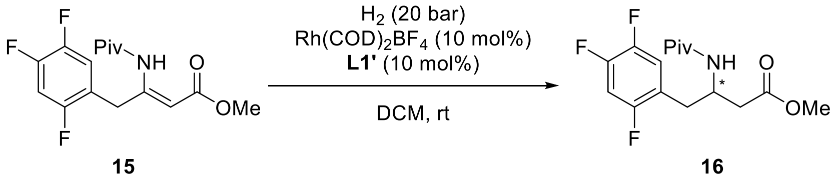

Under nitrogen, a mixture of $Rh(NBD)_2BF_4$ (4.1 mg, 0.01 mmol), **L1′** (7.8 mg, 0.012 mmol), and anhydrous DCM (1.0 mL) was stirred for 30 min. Then, to this solution of chiral rhodium complex was added dehydroamino ester **15** (33.0 mg, 0.1 mmol). The reaction mixture was transferred into an autoclave. The autoclave was evacuated and refilled with H_2 gas (< 5 bar) three times and finally pressurized at 20 bar. The mixture was stirred at room temperature for 12 h and concentrated *in vacuo*. The residue was purified by silica gel column chromatography to afford the hydrogenated product **16** as a colorless oil in 95% yield (31.5 mg, <5% ee).

3. Asymmetric Allylic Substitution of 17

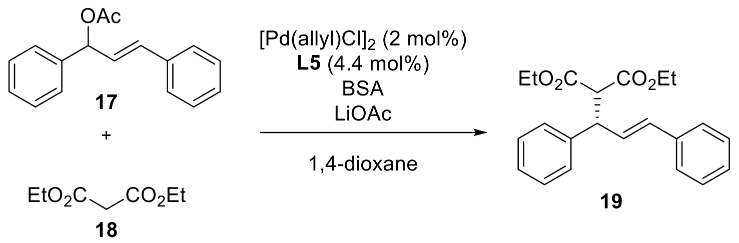

Diethyl (*S*,*E*)-2-(1,3-diphenylallyl)malonate (19). Under N_2, to a vial were added $[Pd(allyl)Cl]_2$ (1.6 mg, 0.005 mmol), **L5** (7.9 mg, 0.012 mmol), and anhydrous DCM (0.5 mL). The mixture was stirred for 1 h to form a solution of the chiral palladium complex. Then, an aliquot of this solution (0.2 mL) was added to a mixture of (*E*)-1,3-diphenylallyl acetate **17** (25.2 mg, 0.1 mmol), diethyl malonate **18** (32.0 mg, 0.2 mmol), LiOAc (19.2 mg, 0.3 mmol), *N,O*-bis(trimethylsilyl)acetamide (60.9 mg, 0.3 mmol), and anhydrous 1,4-dioxane (1.0 mL). The mixture was stirred at room temperature for 12 h before it was directly subjected to silica gel flash chromatography (eluent: hexanes/DCM = 2:1) to afford the product **19** as a colorless oil in 96% yield (33.7 mg, 93% ee). $[\alpha]_D^{20}$: -12.6 (c = 1.0, CH_2Cl_2). HPLC analysis of the product: Daicel CHIRALPAK AD-H column; 20% *i*-PrOH in hexanes; 1.0 mL/min; retention times: 15.9 min (minor), 22.0 min (major).

^{1}H NMR (400 MHz, $CDCl_3$) δ 7.36 – 7.15 (m, 10H), 6.49 (d, J = 15.7 Hz, 1H), 6.35 (dd, J = 15.7, 8.5 Hz, 1H), 4.31 – 4.22 (m, 1H), 4.18 (q, J = 7.2 Hz, 2H), 4.02 – 3.95 (m, 2H), 3.93 (d, J = 11.0 Hz, 1H), 1.21 (t, J = 7.1 Hz, 3H), 1.01 (t, J = 7.1 Hz, 3H).

^{13}C NMR (101 MHz, $CDCl_3$) δ 167.8, 167.4, 140.3, 136.8, 131.6, 129.3, 128.6, 128.4, 127.9, 127.5, 127.1, 126.3, 61.5, 61.3, 57.7, 49.2, 14.1, 13.7.

This is a known compound. The characterization data are consistent with the literature.[2]

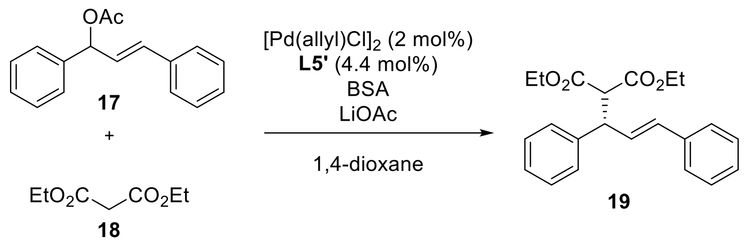

Under N_2, to a vial were added $[Pd(allyl)Cl]_2$ (1.6 mg, 0.005 mmol), **L5′** (7.1 mg, 0.012 mmol), and anhydrous DCM (0.5 mL). The mixture was stirred for 1 h to form a solution of the chiral palladium complex. Then, an aliquot of this solution (0.2 mL) was added to a mixture of (*E*)-1,3-diphenylallyl acetate **17** (25.2 mg, 0.1 mmol), diethyl malonate **18** (32.0 mg, 0.2 mmol), LiOAc (19.2 mg, 0.3 mmol), *N,O*-bis(trimethylsilyl)acetamide (60.9 mg, 0.3 mmol), and anhydrous 1,4-dioxane (1.0 mL). The mixture was stirred at room temperature for 12 h before it was directly subjected to silica gel flash chromatography (eluent: hexanes/DCM = 2:1) to afford the product **19** as a colorless oil in 87% yield (30.6 mg, 5% ee).

4. Asymmetric Morita–Baylis–Hillman Reaction of N-Methyl Isatin 20 with Methyl Acrylate 21

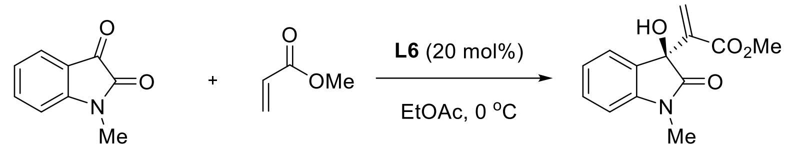

20 **21** **22**

Methyl (*S*)-2-(3-hydroxy-1-methyl-2-oxoindolin-3-yl)acrylate (22). At 0 °C, to a solution of methyl acrylate **21** (42 μL, 0.45 mmol) and chiral organocatalyst **L6** (21.3 mg, 0.03 mmol) in EtOAc (0.5 mL) was added *N*-methyl isatin **20** (24.1 mg, 0.15 mmol). The mixture was stirred at 0 °C for 14 days before it was warmed to room temperature, evaporated and subjected to silica gel flash chromatography (eluent: hexanes/EtOAc = 4:1) to afford the product **22** as a white solid in 86% yield (31.8 mg, 81% ee).

$[\alpha]_D^{20}$: +32.0 (c = 1.0, CH_2Cl_2). HPLC analysis of the product: Daicel CHIRALCEL OD-H column; 10% *i*-PrOH in hexanes; 1.0 mL/min; retention times: 11.6 min (minor), 13.2 min (major).

^{1}H NMR (400 MHz, $CDCl_3$) δ 7.36 – 7.29 (m, 1H), 7.17 (d, *J* = 7.2 Hz, 1H), 7.07 – 6.99 (m, 1H), 6.86 (d, *J* = 7.8 Hz, 1H), 6.55 (s, 1H), 6.43 (s, 1H), 4.01 (s, 1H), 3.62 (s, 3H), 3.24 (s, 3H).

^{13}C NMR (101 MHz, $CDCl_3$) δ 176.3, 165.1, 144.5, 139.1, 130.2, 129.3, 127.8, 123.8, 123.0, 108.6, 76.1, 52.0, 26.4.

This is a known compound. The characterization data are consistent with the literature.[3]

IX. Product Structure Determination

The structure and absolute stereochemistry of SPHENAM (*S*)-**1a** was determined by single crystal X-ray crystallography. The X-ray data have been deposited at the Cambridge Crystallographic Data Center (CCDC 2413493).

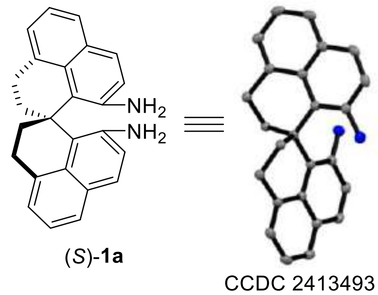

(*S*)-**1a** CCDC 2413493

Table S1. Crystal Data and Structure Refinement for (*S*)-1a

Identification code	(*S*)-**1a**
Empirical formula	$C_{25}H_{22}N_2$
Formula weight	350.44
Temperature/K	100.00(10)
Crystal system	orthorhombic
Space group	$P2_12_12_1$
a/Å	8.19360(10)
b/Å	9.35410(10)
c/Å	22.7707(3)
α/°	90
β/°	90
γ/°	90
Volume/Å^3	1745.23(4)
Z	4

ρ_{calc}g/cm^3	1.334
μ/mm^{-1}	0.598
F(000)	744.0
Crystal size/mm^3	0.2 × 0.18 × 0.15
Radiation	Cu Kα (λ = 1.54184)
2Θ range for data collection/°	7.766 to 153.768
Index ranges	-10 ≤ h ≤ 10, -6 ≤ k ≤ 11, -28 ≤ l ≤ 28
Reflections collected	11185
Independent reflections	3639 [R_{int} = 0.0354, R_{sigma} = 0.0330]
Data/restraints/parameters	3639/0/260
Goodness-of-fit on F^2	1.031
Final R indexes [I>=2σ (I)]	R_1 = 0.0341, wR_2 = 0.0895
Final R indexes [all data]	R_1 = 0.0350, wR_2 = 0.0908
Largest diff. peak/hole / e Å^{-3}	0.25/-0.20
Flack parameter	-0.3(3)

The structure and absolute stereochemistry of NOSPHEN (*S*)-**2** was determined by single crystal X-ray crystallography. The X-ray data have been deposited at the Cambridge Crystallographic Data Center (CCDC 2413494).

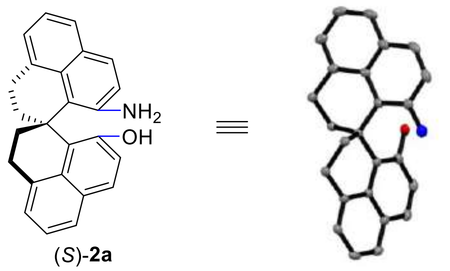

(*S*)-**2a**

CCDC 2413494

Table S2. Crystal Data and Structure Refinement for (*S*)-2a

Identification code	(*S*)-**2a**
Empirical formula	$C_{25}H_{21}NO$
Formula weight	351.43
Temperature/K	100.01(10)
Crystal system	orthorhombic
Space group	$P2_12_12_1$
a/Å	8.20951(12)
b/Å	9.33216(18)
c/Å	22.7053(4)
α/°	90
β/°	90
γ/°	90
Volume/Å^3	1739.51(5)
Z	4
ρ_{calc}g/cm^3	1.342
μ/mm^{-1}	0.630
F(000)	744.0

Crystal size/mm^3	0.2 × 0.13 × 0.12
Radiation	Cu Kα (λ = 1.54184)
2Θ range for data collection/°	10.248 to 154.136
Index ranges	-6 ≤ h ≤ 10, -11 ≤ k ≤ 11, -28 ≤ l ≤ 28
Reflections collected	11437
Independent reflections	3595 [R_{int} = 0.0267, R_{sigma} = 0.0275]
Data/restraints/parameters	3595/38/250
Goodness-of-fit on F^2	1.039
Final R indexes [I>=2σ (I)]	R_1 = 0.0316, wR_2 = 0.0755
Final R indexes [all data]	R_1 = 0.0340, wR_2 = 0.0770
Largest diff. peak/hole / e $Å^{-3}$	0.18/-0.16
Flack parameter	-1.08(19)

The structure and absolute stereochemistry of Diphenyl SPHENAM **1a'** was determined by single crystal X-ray crystallography. The X-ray data have been deposited at the Cambridge Crystallographic Data Center (CCDC 2413495).

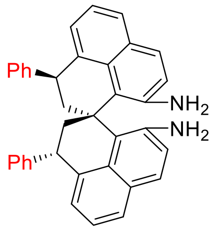

9a

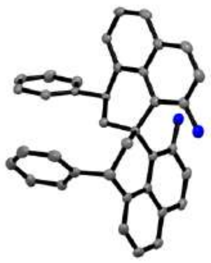

CCDC 2413495

Table S3. Crystal Data and Structure Refinement for 9a

Identification code	**9a**
Empirical formula	$C_{37}H_{30}N_2$
Formula weight	502.63
Temperature/K	100.01(10)
Crystal system	orthorhombic
Space group	$P2_12_12_1$
a/Å	9.01928(9)
b/Å	13.29087(14)
c/Å	21.9107(2)
α/°	90
β/°	90
γ/°	90
Volume/Å^3	2626.52(5)
Z	4
ρ_{calc}g/cm^3	1.271
μ/mm^{-1}	0.562
F(000)	1064.0
Crystal size/mm^3	0.4 × 0.15 × 0.15

Radiation	Cu Kα (λ = 1.54184)
2Θ range for data collection/°	7.78 to 148.456
Index ranges	-10 ≤ h ≤ 10, -15 ≤ k ≤ 16, -27 ≤ l ≤ 21
Reflections collected	15790
Independent reflections	5216 [R_{int} = 0.0252, R_{sigma} = 0.0247]
Data/restraints/parameters	5216/0/355
Goodness-of-fit on F^2	1.036
Final R indexes [I>=2σ (I)]	R_1 = 0.0310, wR_2 = 0.0751
Final R indexes [all data]	R_1 = 0.0335, wR_2 = 0.0769
Largest diff. peak/hole / e Å^{-3}	0.18/-0.15
Flack parameter	-0.5(3)

X. References

(1) Fu, Y., Xie, J.-H., Hu, A.-G., Zhou, H., Wang, L.-X.; Zhou, Q.-L. Novel Monodentate Spiro Phosphorus Ligands for Rhodium-Catalyzed Hydrogenation Reactions. *Chem. Commun*. **2002**, *38*, 480–481.

(2) Matsushima, Y., Onitsuka, K., Kondo, T., Mitsudo, T.; Takahashi, S. Asymmetric Catalysis of Planar-Chiral Cyclopentadienylruthenium Complexes in Allylic Amination and Alkylation. *J. Am. Chem. Soc.* **2001**, *123*, 10405–10406.

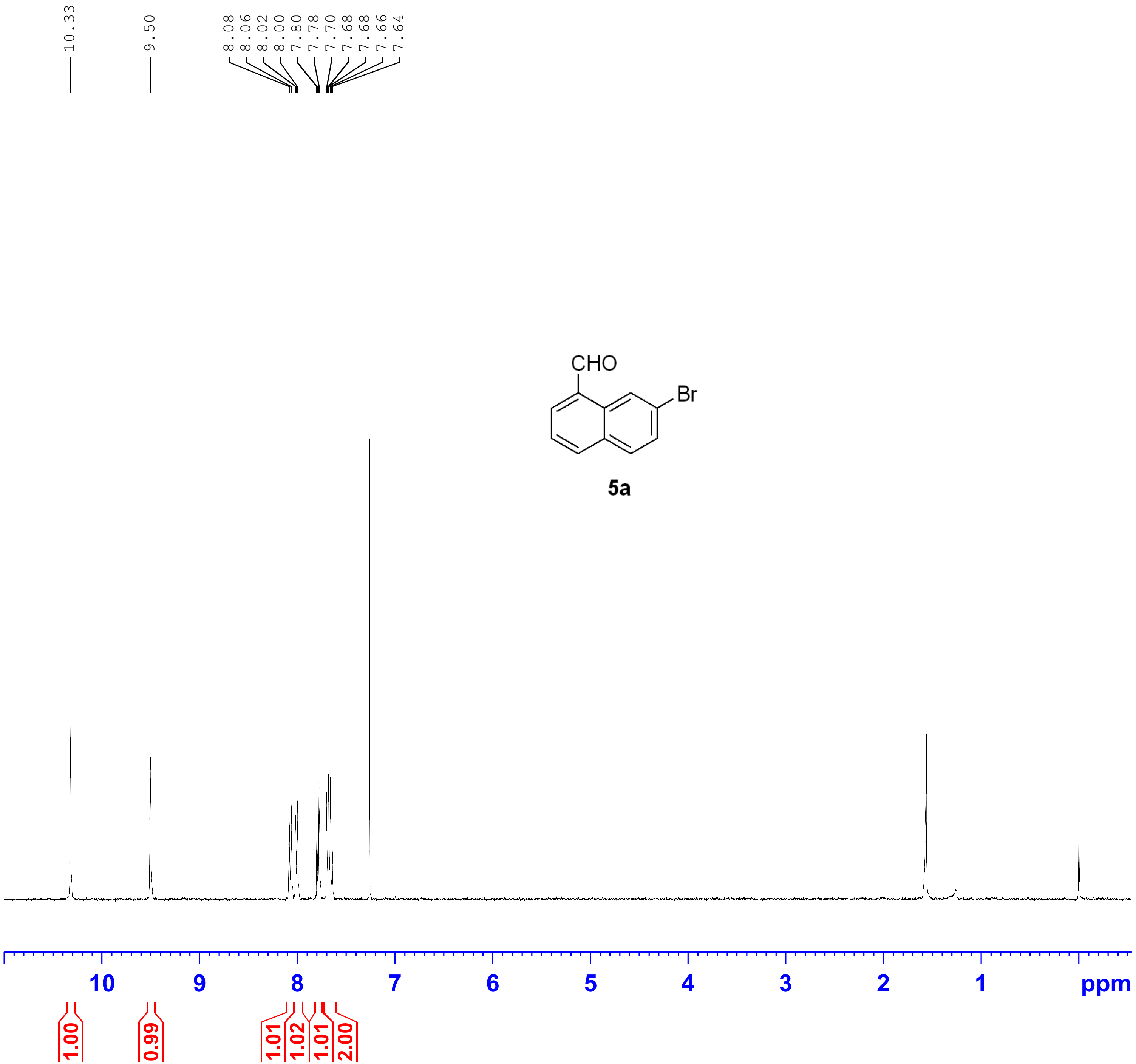


```
Current Data Parameters
NAME      zrh-6-25-1st-h
EXPNO                  1
PROCNO                 1

F2 - Acquisition Parameters
Date_           20220414
Time               14.42
INSTRUM            spect
PROBHD   5 mm DUL 13C-1
PULPROG             zg30
TD                 65536
SOLVENT            CDCl3
NS                     1
DS                     0
SWH             8223.685 Hz
FIDRES          0.125483 Hz
AQ             3.9845889 sec
RG                   724
DW                60.800 usec
DE                  6.00 usec
TE                 293.4 K
D1            1.00000000 sec
TD0                    1

======== CHANNEL f1 ========
NUC1                  1H
P1                 15.80 usec
PL1                -1.00 dB
PL1W         12.17476940 W
SFO1         400.1324710 MHz

F2 - Processing parameters
SI                 32768
SF           400.1300100 MHz
WDW                   EM
SSB                    0
LB                  0.30 Hz
GB                     0
PC                  1.00
```

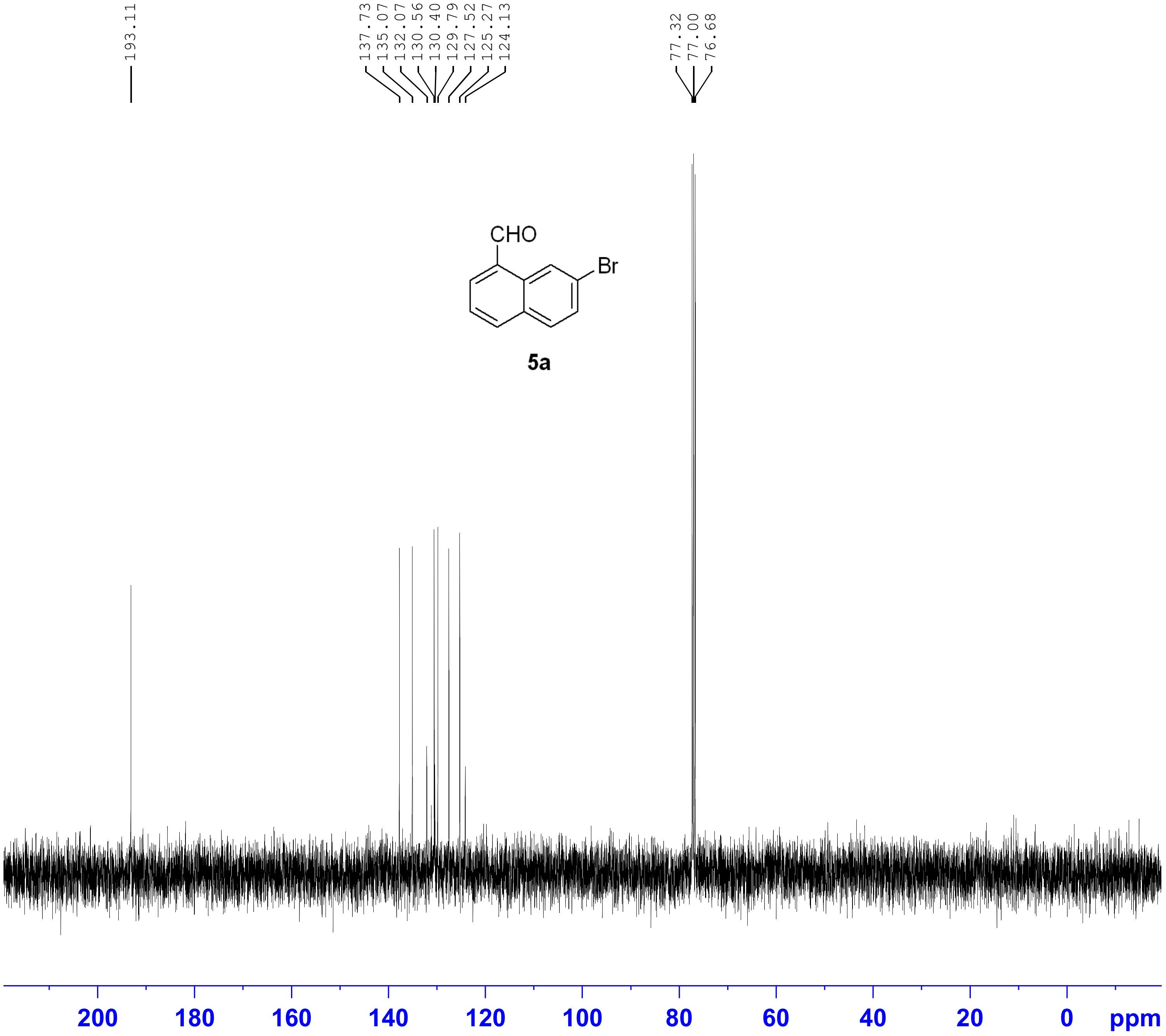

Current Data Parameters
NAME zrh-6-25-c
EXPNO 1
PROCNO 1

F2 - Acquisition Parameters
Date_ 20220429
Time 15.43
INSTRUM spect
PROBHD 5 mm DUL 13C-1
PULPROG zgpg30
TD 65536
SOLVENT CDCl3
NS 100
DS 0
SWH 24038.461 Hz
FIDRES 0.366798 Hz
AQ 1.3631488 sec
RG 2050
DW 20.800 usec
DE 6.00 usec
TE 292.8 K
D1 2.00000000 sec
D11 0.03000000 sec
TD0 1

======== CHANNEL f1 ========
NUC1 13C
P1 40.00 usec
PL1 -3.00 dB
PL1W 60.64365387 W
SFO1 100.6228298 MHz

======== CHANNEL f2 ========
CPDPRG[2 waltz16
NUC2 1H
PCPD2 80.00 usec
PL2 -1.00 dB
PL12 14.39 dB
PL13 18.00 dB
PL2W 12.17476940 W
PL12W 0.35193357 W
PL13W 0.15327126 W
SFO2 400.1316005 MHz

F2 - Processing parameters
SI 32768
SF 100.6127747 MHz
WDW EM
SSB 0
LB 1.00 Hz
GB 0
PC 1.40

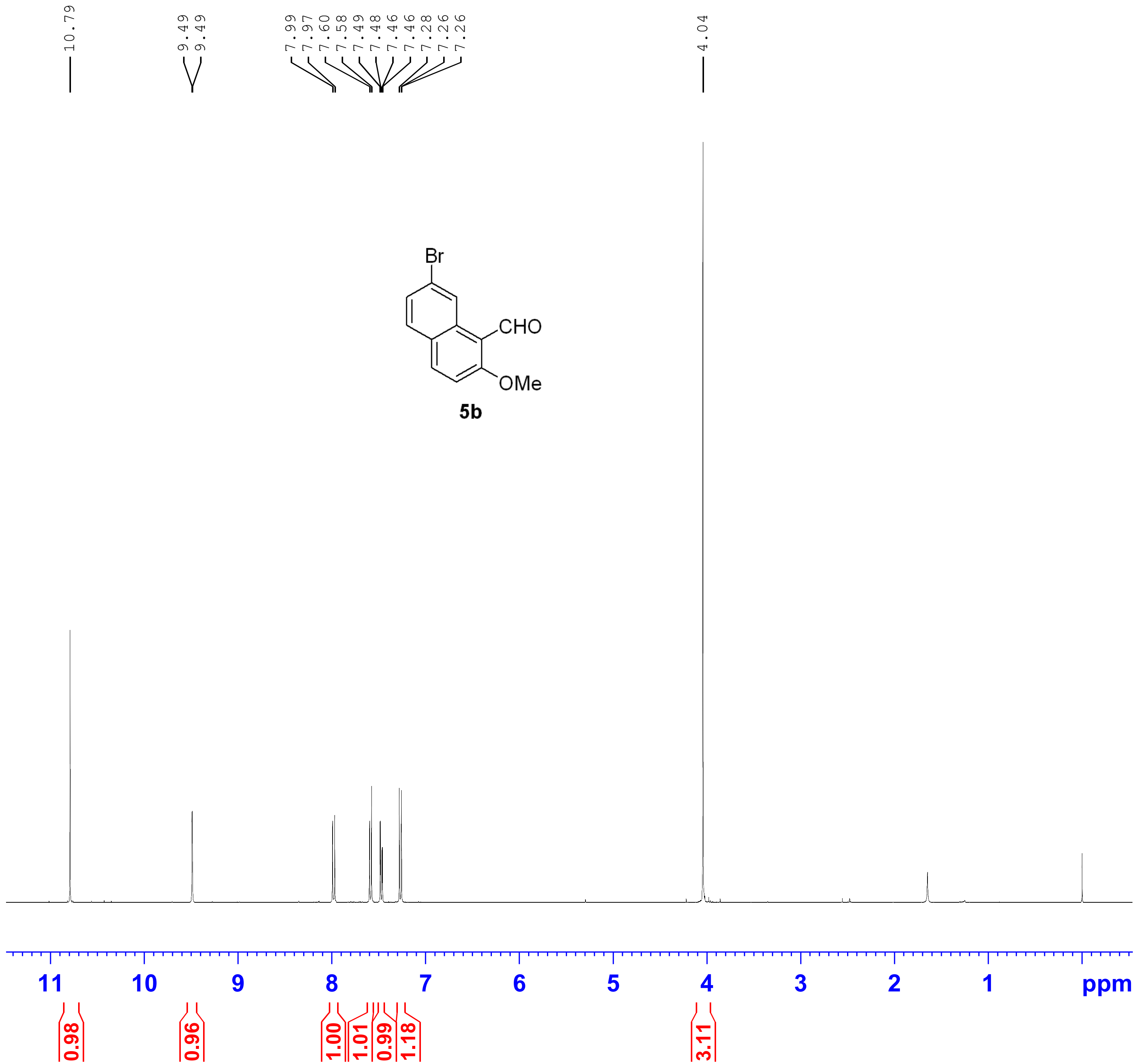


```
Current Data Parameters
NAME          zrh-6-101-h
EXPNO                   1
PROCNO                  1

F2 - Acquisition Parameters
Date_            20220610
Time                11.16
INSTRUM             spect
PROBHD   5 mm DUL 13C-1
PULPROG              zg30
TD                  65536
SOLVENT             CDCl3
NS                      4
DS                      0
SWH              8223.685 Hz
FIDRES           0.125483 Hz
AQ              3.9845889 sec
RG                    362
DW                 60.800 usec
DE                   6.00 usec
TE                  293.4 K
D1             1.00000000 sec
TD0                     1

======== CHANNEL f1 ========
NUC1                   1H
P1                  15.80 usec
PL1                 -1.00 dB
PL1W          12.17476940 W
SFO1          400.1324710 MHz

F2 - Processing parameters
SI                  32768
SF            400.1300098 MHz
WDW                    EM
SSB                     0
LB                   0.30 Hz
GB                      0
PC                   1.00
```

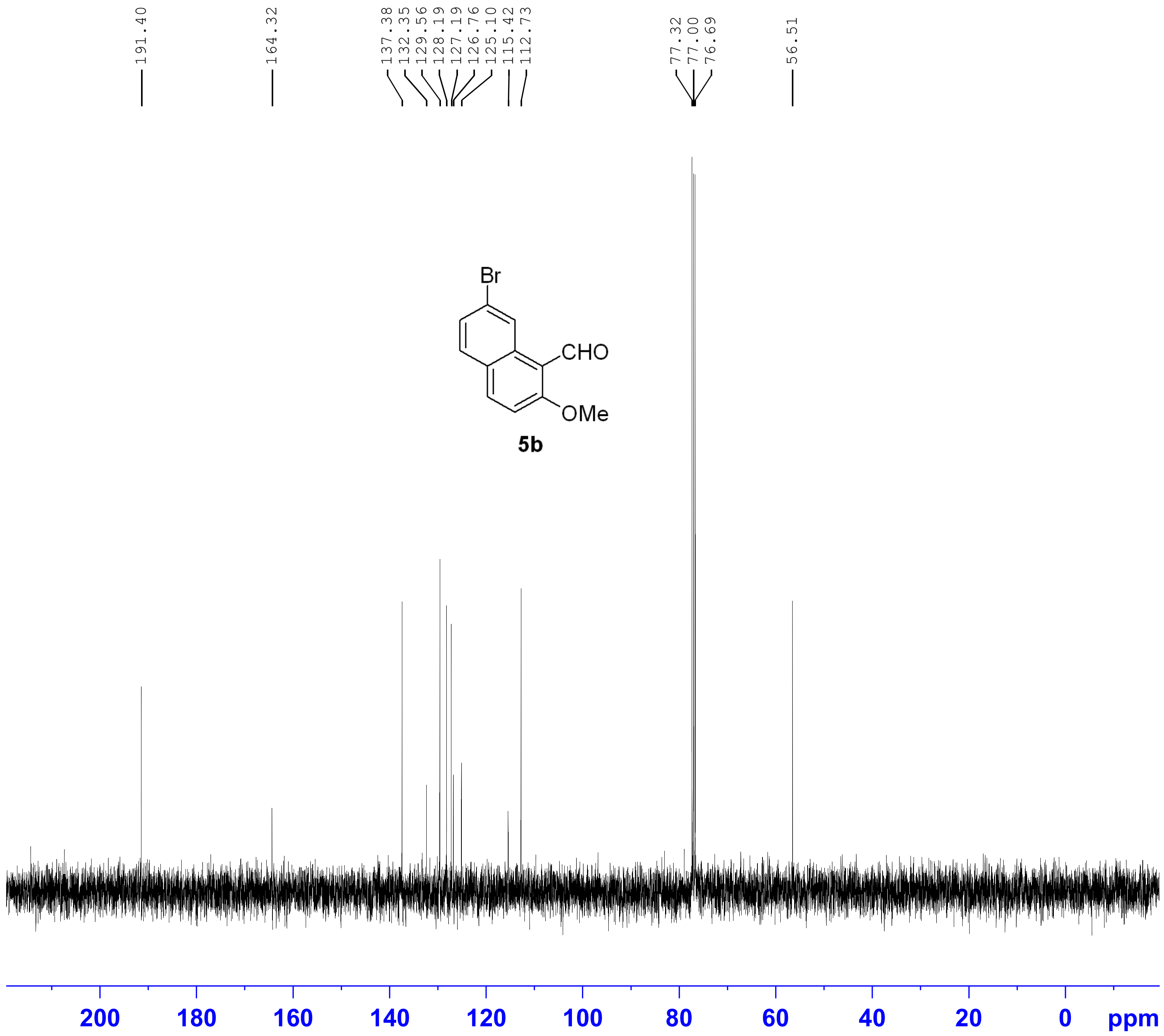


```
Current Data Parameters
NAME          zrh-6-101-c
EXPNO                   1
PROCNO                  1

F2 - Acquisition Parameters
Date_            20220610
Time                11.18
INSTRUM             spect
PROBHD    5 mm DUL 13C-1
PULPROG           zgpg30
TD                  65536
SOLVENT             CDCl3
NS                     48
DS                      0
SWH             24038.461 Hz
FIDRES           0.366798 Hz
AQ              1.3631488 sec
RG                   2050
DW                 20.800 usec
DE                   6.00 usec
TE                  293.7 K
D1             2.00000000 sec
D11            0.03000000 sec
TD0                     1

======== CHANNEL f1 ========
NUC1                  13C
P1                  40.00 usec
PL1                 -3.00 dB
PL1W          60.64365387 W
SFO1          100.6228298 MHz

======== CHANNEL f2 ========
CPDPRG[2          waltz16
NUC2                   1H
PCPD2               80.00 usec
PL2                 -1.00 dB
PL12                14.39 dB
PL13                18.00 dB
PL2W          12.17476940 W
PL12W          0.35193357 W
PL13W          0.15327126 W
SFO2          400.1316005 MHz

F2 - Processing parameters
SI                  32768
SF            100.6127744 MHz
WDW                    EM
SSB                     0
LB                   1.00 Hz
GB                      0
PC                   1.40
```

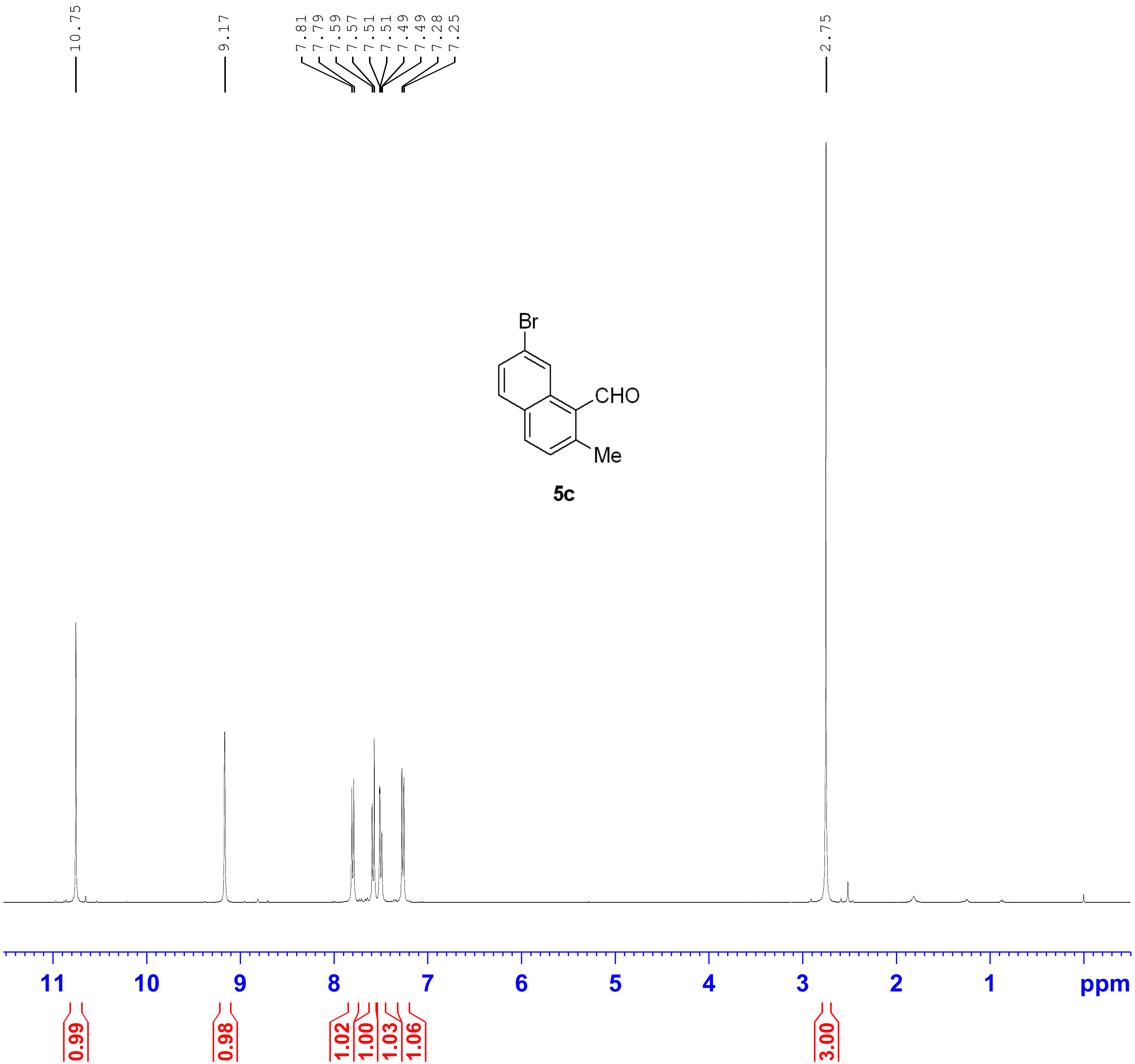


```
Current Data Parameters
NAME     zrh-6-184-pure-h
EXPNO                   1
PROCNO                  1

F2 - Acquisition Parameters
Date_            20220627
Time                21.21
INSTRUM             spect
PROBHD   5 mm DUL 13C-1
PULPROG              zg30
TD                  65536
SOLVENT             CDCl3
NS                      2
DS                      0
SWH              8223.685 Hz
FIDRES           0.125483 Hz
AQ              3.9845889 sec
RG                    144
DW                 60.800 usec
DE                   6.00 usec
TE                  293.2 K
D1             1.00000000 sec
TD0                     1

======== CHANNEL f1 ========
NUC1                   1H
P1                  15.80 usec
PL1                 -1.00 dB
PL1W          12.17476940 W
SFO1          400.1324710 MHz

F2 - Processing parameters
SI                  32768
SF            400.1300095 MHz
WDW                    EM
SSB                     0
LB                   0.30 Hz
GB                      0
PC                   1.00
```

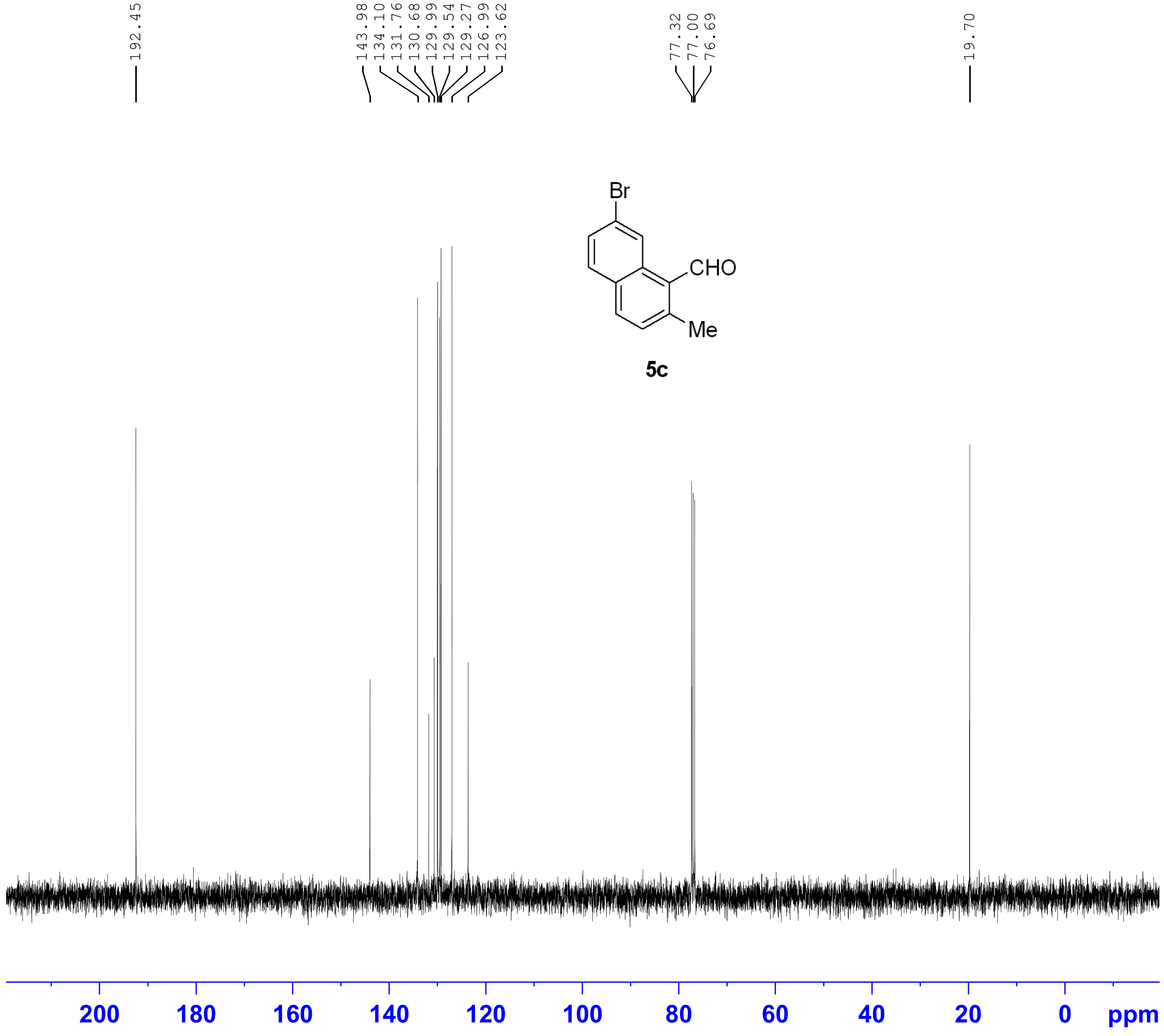


```
Current Data Parameters
NAME      zrh-6-184-pure-c
EXPNO                    1
PROCNO                   1

F2 - Acquisition Parameters
Date_             20220627
Time                 21.23
INSTRUM              spect
PROBHD    5 mm DUL 13C-1
PULPROG            zgpg30
TD                   65536
SOLVENT              CDCl3
NS                      41
DS                       0
SWH              24038.461 Hz
FIDRES            0.366798 Hz
AQ               1.3631488 sec
RG                    2050
DW                  20.800 usec
DE                    6.00 usec
TE                   293.4 K
D1              2.00000000 sec
D11             0.03000000 sec
TD0                      1

======== CHANNEL f1 ========
NUC1                   13C
P1                   40.00 usec
PL1                  -3.00 dB
PL1W           60.64365387 W
SFO1          100.6228298 MHz

======== CHANNEL f2 ========
CPDPRG[2           waltz16
NUC2                    1H
PCPD2                80.00 usec
PL2                  -1.00 dB
PL12                 14.39 dB
PL13                 18.00 dB
PL2W           12.17476940 W
PL12W           0.35193357 W
PL13W           0.15327126 W
SFO2          400.1316005 MHz

F2 - Processing parameters
SI                   32768
SF            100.6127825 MHz
WDW                     EM
SSB                      0
LB                    1.00 Hz
GB                       0
PC                    1.40
```

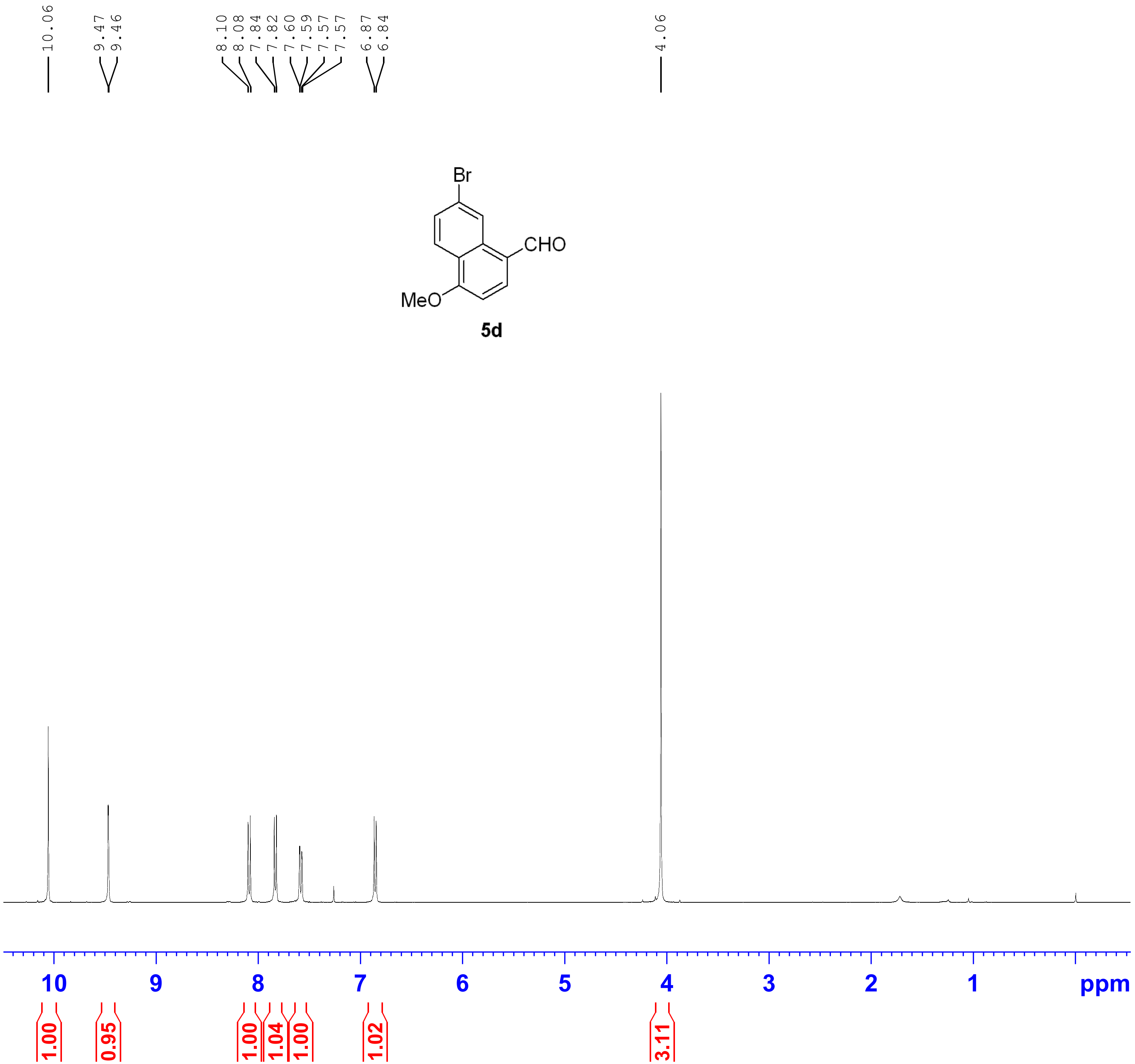


```
Current Data Parameters
NAME         zrh-7-45-re-h
EXPNO                    1
PROCNO                   1

F2 - Acquisition Parameters
Date_             20230207
Time                 10.12
INSTRUM              spect
PROBHD      5 mm PABBO BB/
PULPROG               zg30
TD                   65536
SOLVENT              CDCl3
NS                       3
DS                       2
SWH               8012.820 Hz
FIDRES            0.122266 Hz
AQ               4.0894465 sec
RG                   62.93
DW                  62.400 usec
DE                    6.50 usec
TE                   295.6 K
D1              1.00000000 sec
TD0                      1

======== CHANNEL f1 ========
SFO1           400.1324710 MHz
NUC1                    1H
P1                   14.50 usec
PLW1           11.99499989 W

F2 - Processing parameters
SI                   65536
SF             400.1300100 MHz
WDW                     EM
SSB                      0
LB                    0.30 Hz
GB                       0
PC                    1.00
```

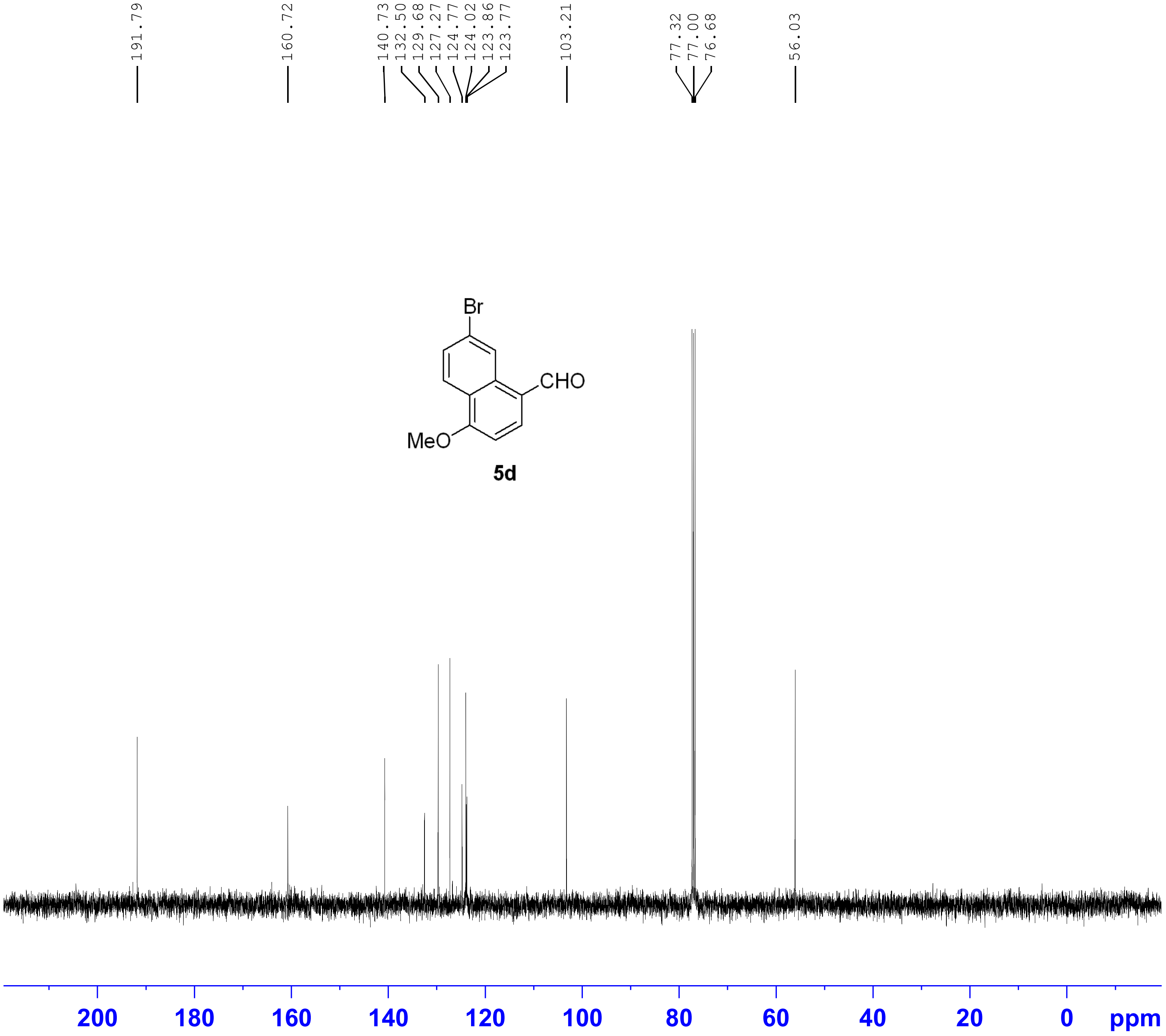


```
Current Data Parameters
NAME          zrh-7-45-re-c
EXPNO                     1
PROCNO                    1

F2 - Acquisition Parameters
Date_              20230207
Time                  10.13
INSTRUM               spect
PROBHD    5 mm PABBO BB/
PULPROG             zgpg30
TD                    65536
SOLVENT               CDCl3
NS                       20
DS                        2
SWH           24038.461 Hz
FIDRES         0.366798 Hz
AQ            1.3631488 sec
RG                   196.92
DW               20.800 usec
DE                 6.50 usec
TE                296.0 K
D1           2.00000000 sec
D11          0.03000000 sec
TD0                       1

======== CHANNEL f1 ========
SFO1        100.6228298 MHz
NUC1                    13C
P1                 9.70 usec
PLW1        46.98899841 W

======== CHANNEL f2 ========
SFO2        400.1316005 MHz
NUC2                     1H
CPDPRG[2            waltz16
PCPD2             90.00 usec
PLW2        11.99499989 W
PLW12        0.34213999 W
PLW13        0.27713001 W

F2 - Processing parameters
SI                    32768
SF          100.6127772 MHz
WDW                      EM
SSB                       0
LB                  1.00 Hz
GB                        0
PC                     1.40
```

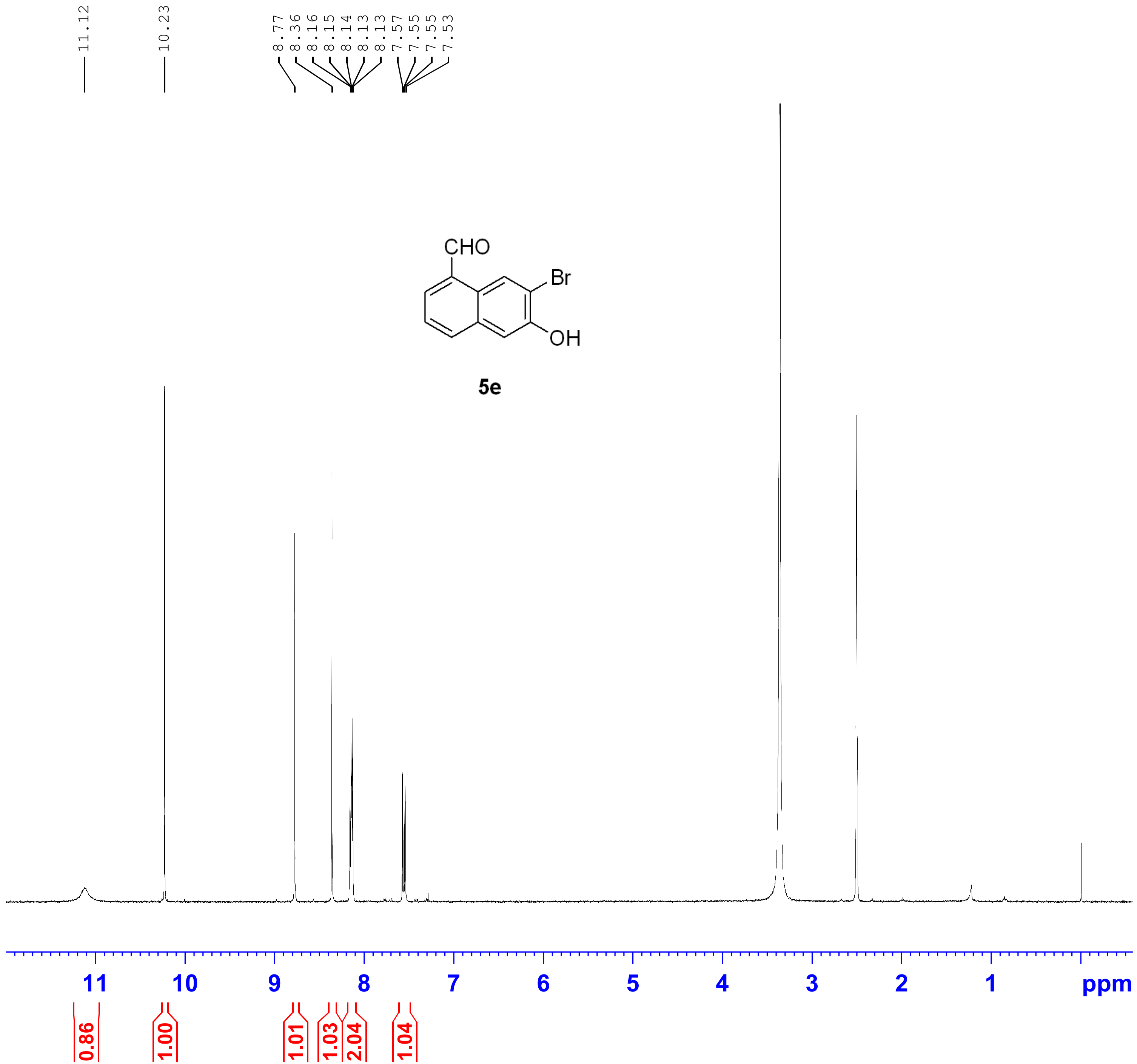

Current Data Parameters
NAME zrh-5-188-bypdt-h
EXPNO 1
PROCNO 1

F2 - Acquisition Parameters
Date_ 20220401
Time 10.57
INSTRUM spect
PROBHD 5 mm PABBO BB/
PULPROG zg30
TD 65536
SOLVENT DMSO
NS 2
DS 0
SWH 8012.820 Hz
FIDRES 0.122266 Hz
AQ 4.0894465 sec
RG 112.31
DW 62.400 usec
DE 6.50 usec
TE 295.4 K
D1 1.00000000 sec
TD0 1

======== CHANNEL f1 ========
SFO1 400.1324710 MHz
NUC1 1H
P1 14.50 usec
PLW1 11.99499989 W

F2 - Processing parameters
SI 65536
SF 400.1300036 MHz
WDW EM
SSB 0
LB 0.30 Hz
GB 0
PC 1.00

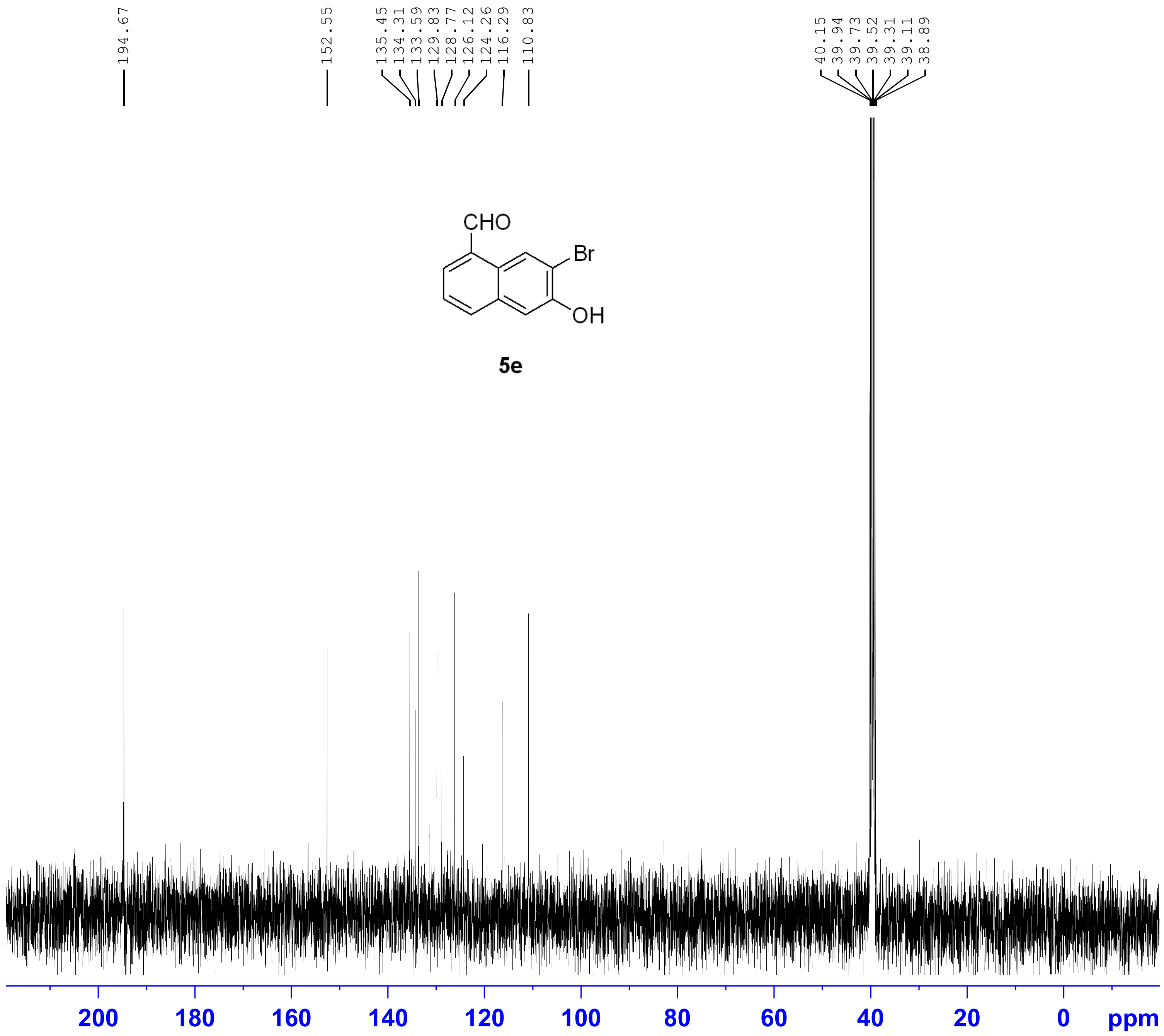


```
Current Data Parameters
NAME     zrh-5-188-bypdt-re-c
EXPNO                   1
PROCNO                  1

F2 - Acquisition Parameters
Date_            20220607
Time                15.55
INSTRUM             spect
PROBHD    5 mm DUL 13C-1
PULPROG            zgpg30
TD                  65536
SOLVENT              DMSO
NS                    175
DS                      0
SWH             24038.461 Hz
FIDRES           0.366798 Hz
AQ              1.3631488 sec
RG                   2050
DW                 20.800 usec
DE                   6.00 usec
TE                  292.8 K
D1             2.00000000 sec
D11            0.03000000 sec
TD0                     1

======== CHANNEL f1 ========
NUC1                  13C
P1                  40.00 usec
PL1                 -3.00 dB
PL1W          60.64365387 W
SFO1          100.6228298 MHz

======== CHANNEL f2 ========
CPDPRG[2          waltz16
NUC2                   1H
PCPD2               80.00 usec
PL2                 -1.00 dB
PL12                14.39 dB
PL13                18.00 dB
PL2W          12.17476940 W
PL12W          0.35193357 W
PL13W          0.15327126 W
SFO2          400.1316005 MHz

F2 - Processing parameters
SI                  32768
SF            100.6128091 MHz
WDW                    EM
SSB                     0
LB                   1.00 Hz
GB                      0
PC                   1.40
```

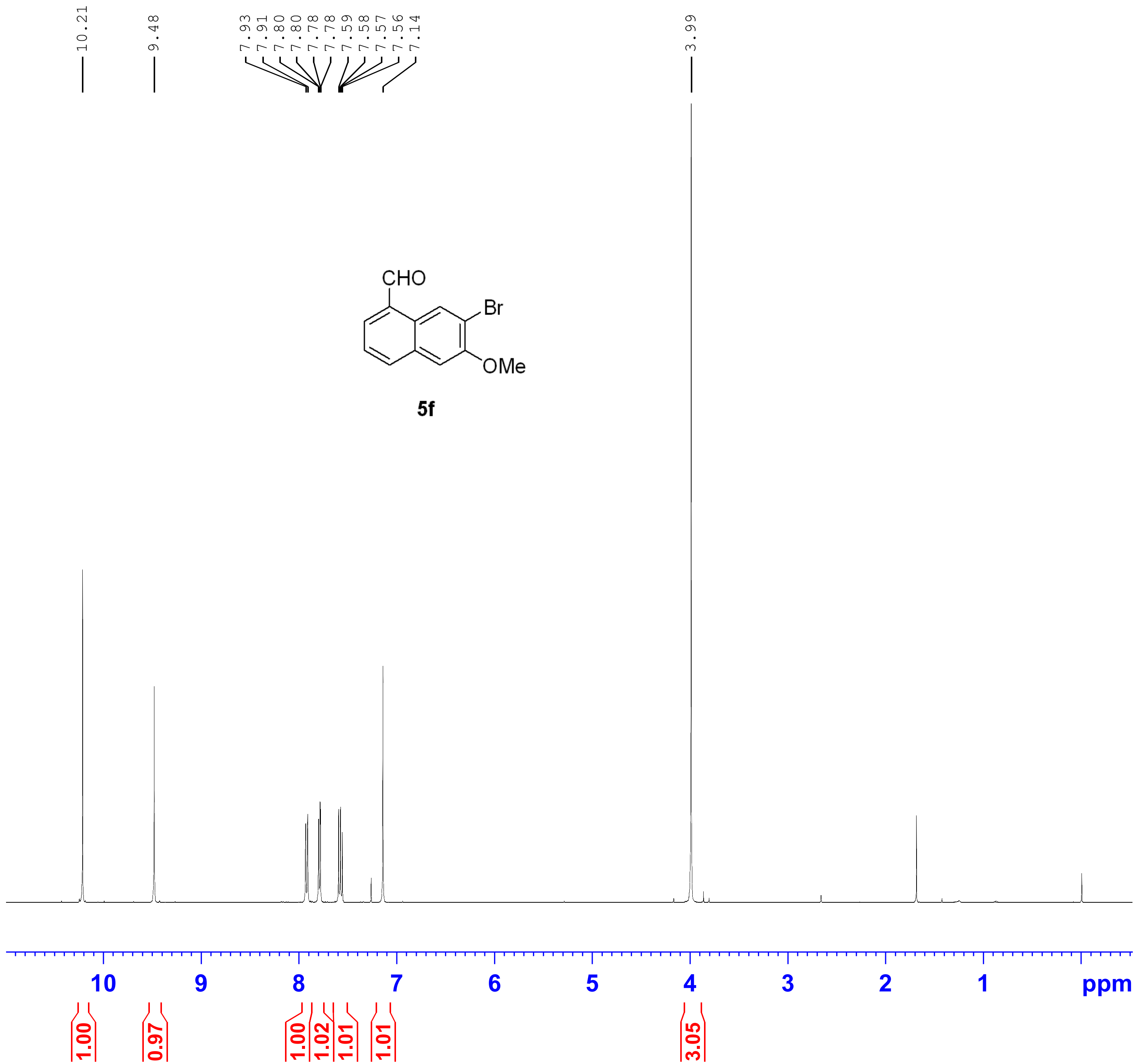


```
Current Data Parameters
NAME              zrh-6-76-h
EXPNO                      1
PROCNO                     1

F2 - Acquisition Parameters
Date_               20220511
Time                   14.58
INSTRUM                spect
PROBHD     5 mm PABBO BB/
PULPROG                 zg30
TD                     65536
SOLVENT                CDCl3
NS                         2
DS                         0
SWH                 8012.820 Hz
FIDRES              0.122266 Hz
AQ                 4.0894465 sec
RG                     49.32
DW                    62.400 usec
DE                      6.50 usec
TE                     296.3 K
D1                1.00000000 sec
TD0                        1

======== CHANNEL f1 ========
SFO1             400.1324710 MHz
NUC1                      1H
P1                     14.50 usec
PLW1             11.99499989 W

F2 - Processing parameters
SI                     65536
SF               400.1300099 MHz
WDW                       EM
SSB                        0
LB                      0.30 Hz
GB                         0
PC                      1.00
```

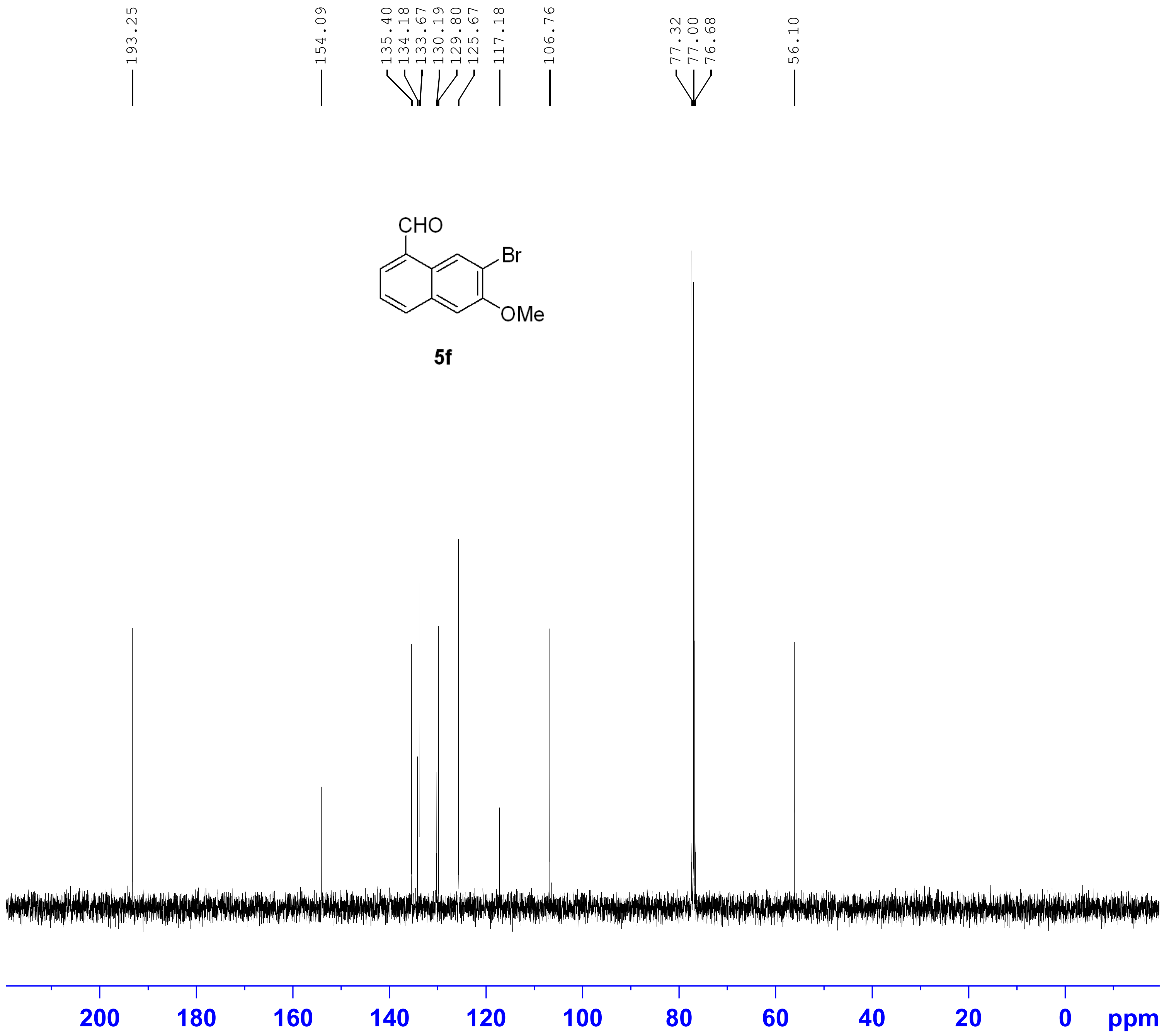


```
Current Data Parameters
NAME          zrh-6-76-c
EXPNO                  1
PROCNO                 1

F2 - Acquisition Parameters
Date_           20220511
Time               15.00
INSTRUM            spect
PROBHD   5 mm PABBO BB/
PULPROG          zgpg30
TD                65536
SOLVENT            CDCl3
NS                    10
DS                     0
SWH            24038.461 Hz
FIDRES          0.366798 Hz
AQ             1.3631488 sec
RG                196.92
DW                20.800 usec
DE                  6.50 usec
TE                 296.6 K
D1            2.00000000 sec
D11           0.03000000 sec
TD0                    1

======== CHANNEL f1 ========
SFO1         100.6228298 MHz
NUC1                 13C
P1                  9.70 usec
PLW1         46.98899841 W

======== CHANNEL f2 ========
SFO2         400.1316005 MHz
NUC2                  1H
CPDPRG[2         waltz16
PCPD2              90.00 usec
PLW2         11.99499989 W
PLW12         0.34213999 W
PLW13         0.27713001 W

F2 - Processing parameters
SI                 32768
SF           100.6127774 MHz
WDW                   EM
SSB                    0
LB                  1.00 Hz
GB                     0
PC                  1.40
```

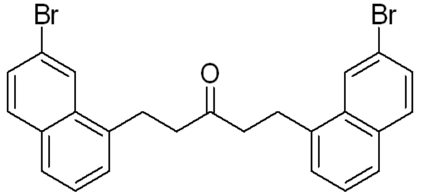

S3a

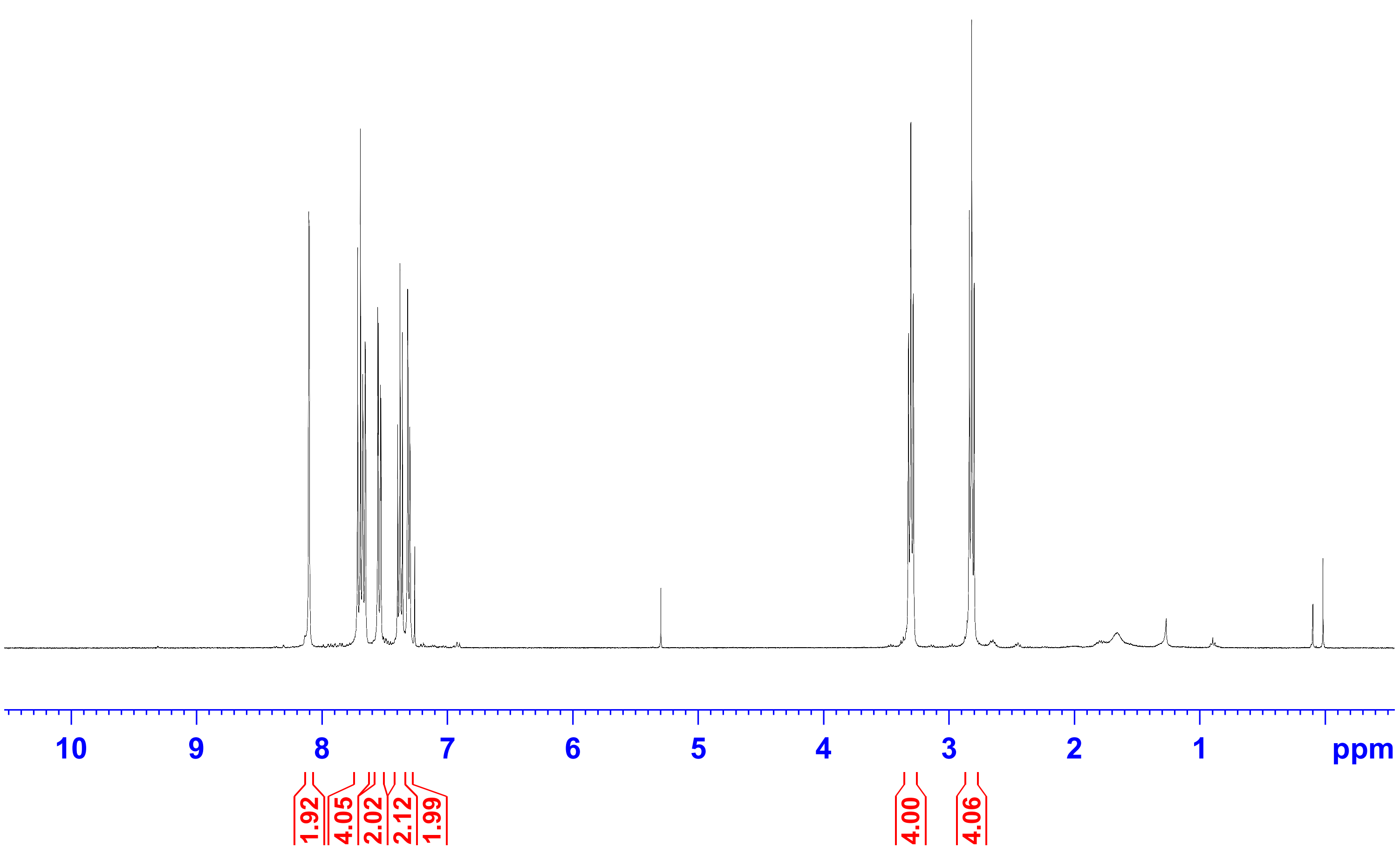

Current Data Parameters
NAME zrh-6-28-3-h
EXPNO 1
PROCNO 1

F2 - Acquisition Parameters
Date_ 20220415
Time 15.00
INSTRUM spect
PROBHD 5 mm PABBO BB/
PULPROG zg30
TD 65536
SOLVENT CDCl3
NS 2
DS 0
SWH 8012.820 Hz
FIDRES 0.122266 Hz
AQ 4.0894465 sec
RG 82.92
DW 62.400 usec
DE 6.50 usec
TE 294.7 K
D1 1.00000000 sec
TD0 1

======== CHANNEL f1 ========
SFO1 400.1324710 MHz
NUC1 1H
P1 14.50 usec
PLW1 11.99499989 W

F2 - Processing parameters
SI 65536
SF 400.1300099 MHz
WDW EM
SSB 0
LB 0.30 Hz
GB 0
PC 1.00

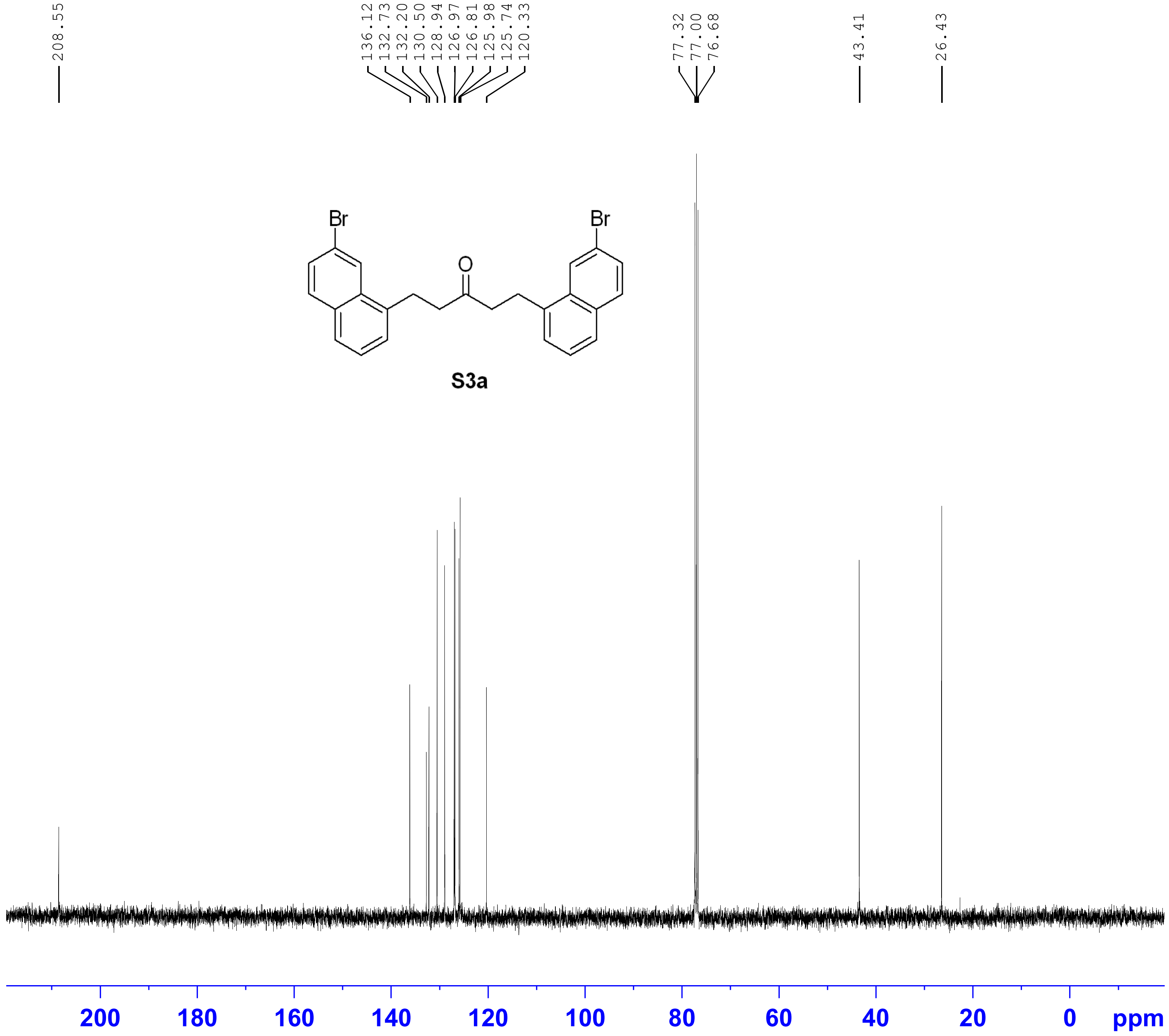

Current Data Parameters
NAME zrh-6-28-3-c
EXPNO 1
PROCNO 1

F2 - Acquisition Parameters
Date_ 20220415
Time 15.01
INSTRUM spect
PROBHD 5 mm PABBO BB/
PULPROG zgpg30
TD 65536
SOLVENT CDCl3
NS 71
DS 0
SWH 24038.461 Hz
FIDRES 0.366798 Hz
AQ 1.3631488 sec
RG 196.92
DW 20.800 usec
DE 6.50 usec
TE 295.2 K
D1 2.00000000 sec
D11 0.03000000 sec
TD0 1

======== CHANNEL f1 ========
SFO1 100.6228298 MHz
NUC1 13C
P1 9.70 usec
PLW1 46.98899841 W

======== CHANNEL f2 ========
SFO2 400.1316005 MHz
NUC2 1H
CPDPRG[2 waltz16
PCPD2 90.00 usec
PLW2 11.99499989 W
PLW12 0.34213999 W
PLW13 0.27713001 W

F2 - Processing parameters
SI 32768
SF 100.6127766 MHz
WDW EM
SSB 0
LB 1.00 Hz
GB 0
PC 1.40

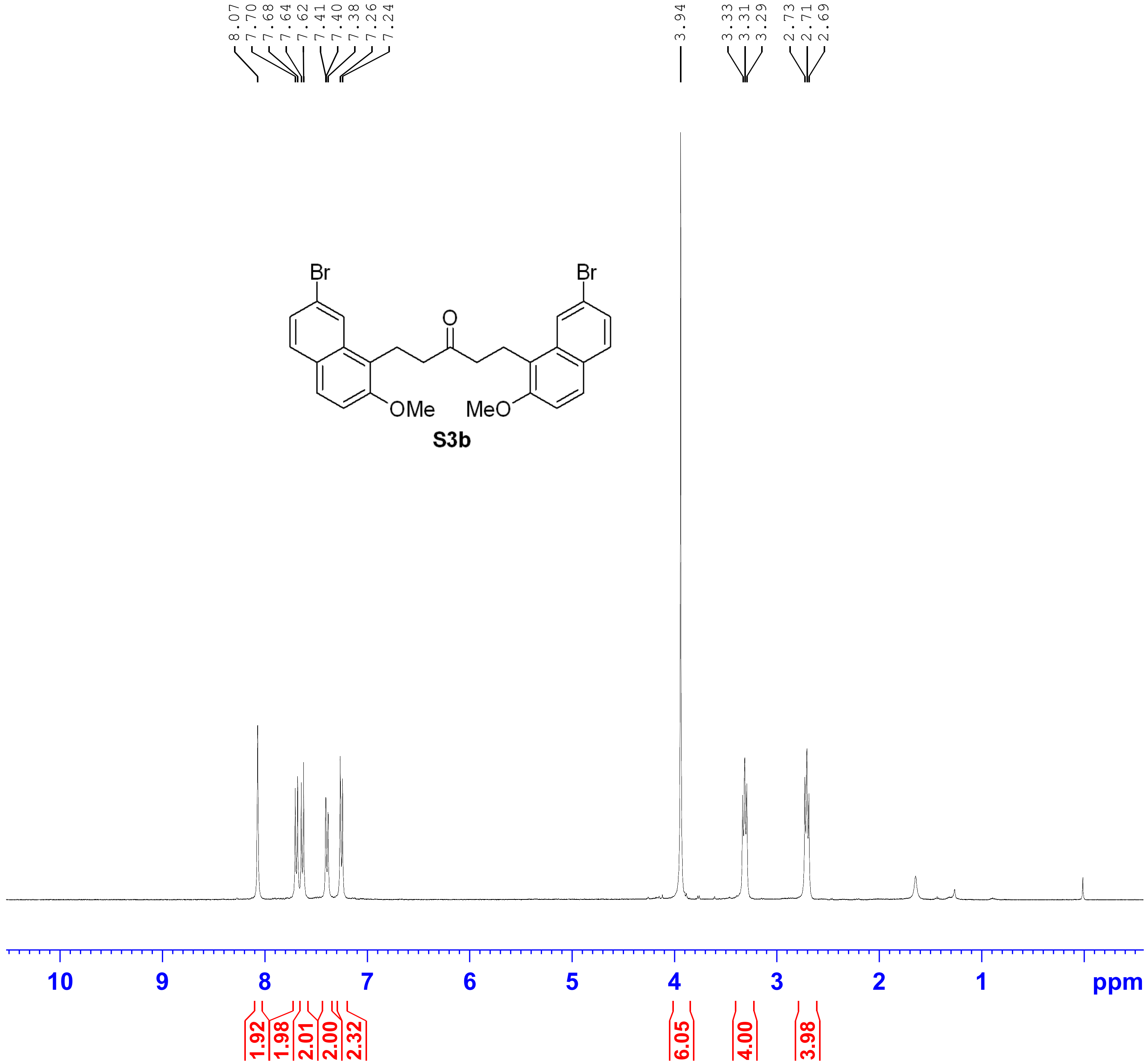


```
Current Data Parameters
NAME       zrh-6-126-re-h
EXPNO                   1
PROCNO                  1

F2 - Acquisition Parameters
Date_            20220602
Time                19.34
INSTRUM             spect
PROBHD     5 mm DUL 13C-1
PULPROG              zg30
TD                  65536
SOLVENT             CDCl3
NS                      2
DS                      0
SWH              8223.685 Hz
FIDRES           0.125483 Hz
AQ              3.9845889 sec
RG                    256
DW                 60.800 usec
DE                   6.00 usec
TE                  292.7 K
D1             1.00000000 sec
TD0                     1

======== CHANNEL f1 ========
NUC1                   1H
P1                  15.80 usec
PL1                 -1.00 dB
PL1W          12.17476940 W
SFO1          400.1324710 MHz

F2 - Processing parameters
SI                  32768
SF            400.1300100 MHz
WDW                    EM
SSB                     0
LB                   0.30 Hz
GB                      0
PC                   1.00
```

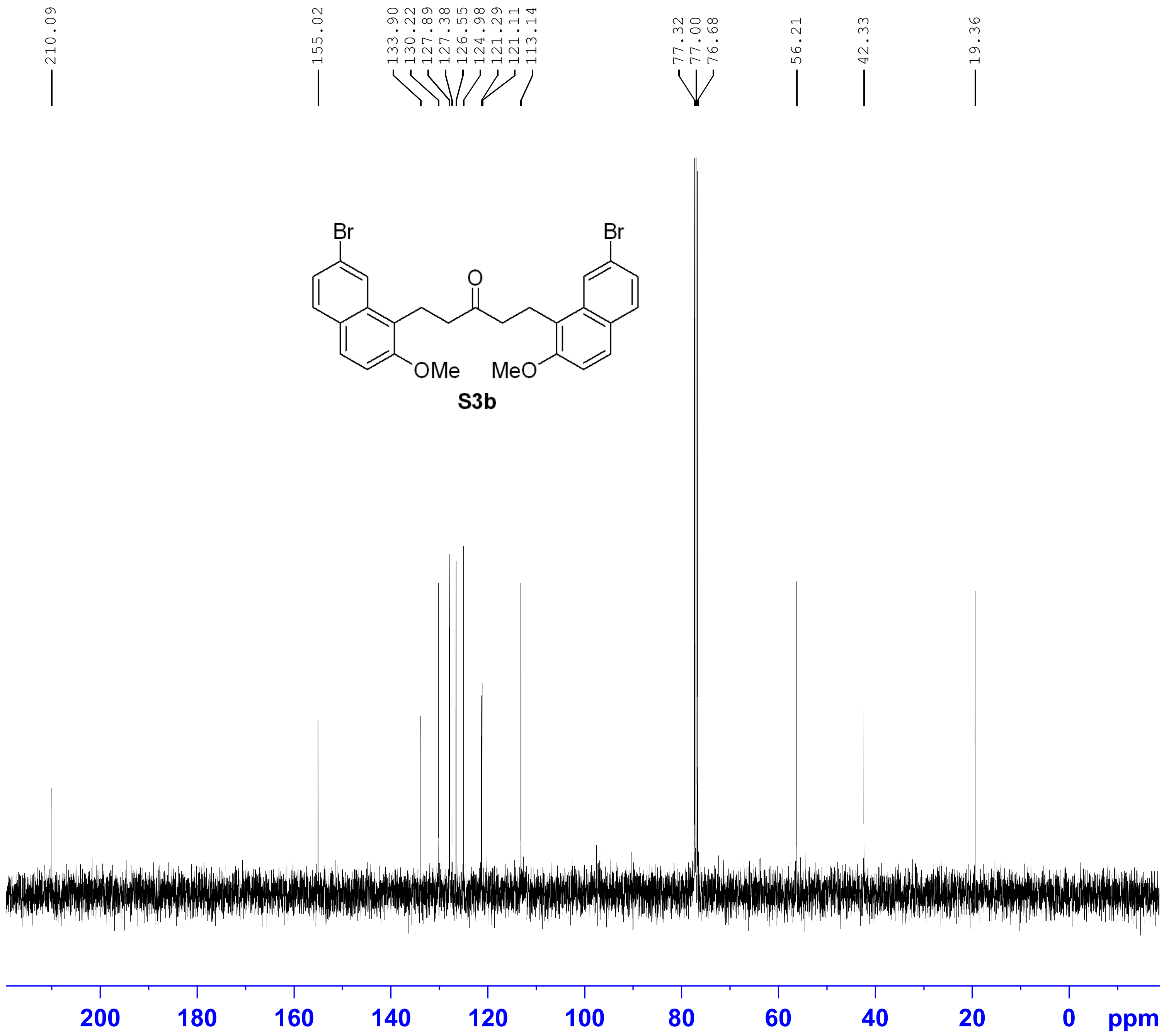


```
Current Data Parameters
NAME        zrh-6-126-re-c
EXPNO                    1
PROCNO                   1

F2 - Acquisition Parameters
Date_             20220602
Time                 19.36
INSTRUM              spect
PROBHD      5 mm DUL 13C-1
PULPROG            zgpg30
TD                   65536
SOLVENT              CDCl3
NS                      63
DS                       0
SWH              24038.461 Hz
FIDRES            0.366798 Hz
AQ               1.3631488 sec
RG                    2050
DW                  20.800 usec
DE                    6.00 usec
TE                   292.9 K
D1              2.00000000 sec
D11             0.03000000 sec
TD0                      1

======== CHANNEL f1 ========
NUC1                   13C
P1                   40.00 usec
PL1                  -3.00 dB
PL1W           60.64365387 W
SFO1           100.6228298 MHz

======== CHANNEL f2 ========
CPDPRG[2           waltz16
NUC2                    1H
PCPD2                80.00 usec
PL2                  -1.00 dB
PL12                 14.39 dB
PL13                 18.00 dB
PL2W           12.17476940 W
PL12W           0.35193357 W
PL13W           0.15327126 W
SFO2           400.1316005 MHz

F2 - Processing parameters
SI                   32768
SF             100.6127758 MHz
WDW                     EM
SSB                      0
LB                    1.00 Hz
GB                       0
PC                    1.40
```

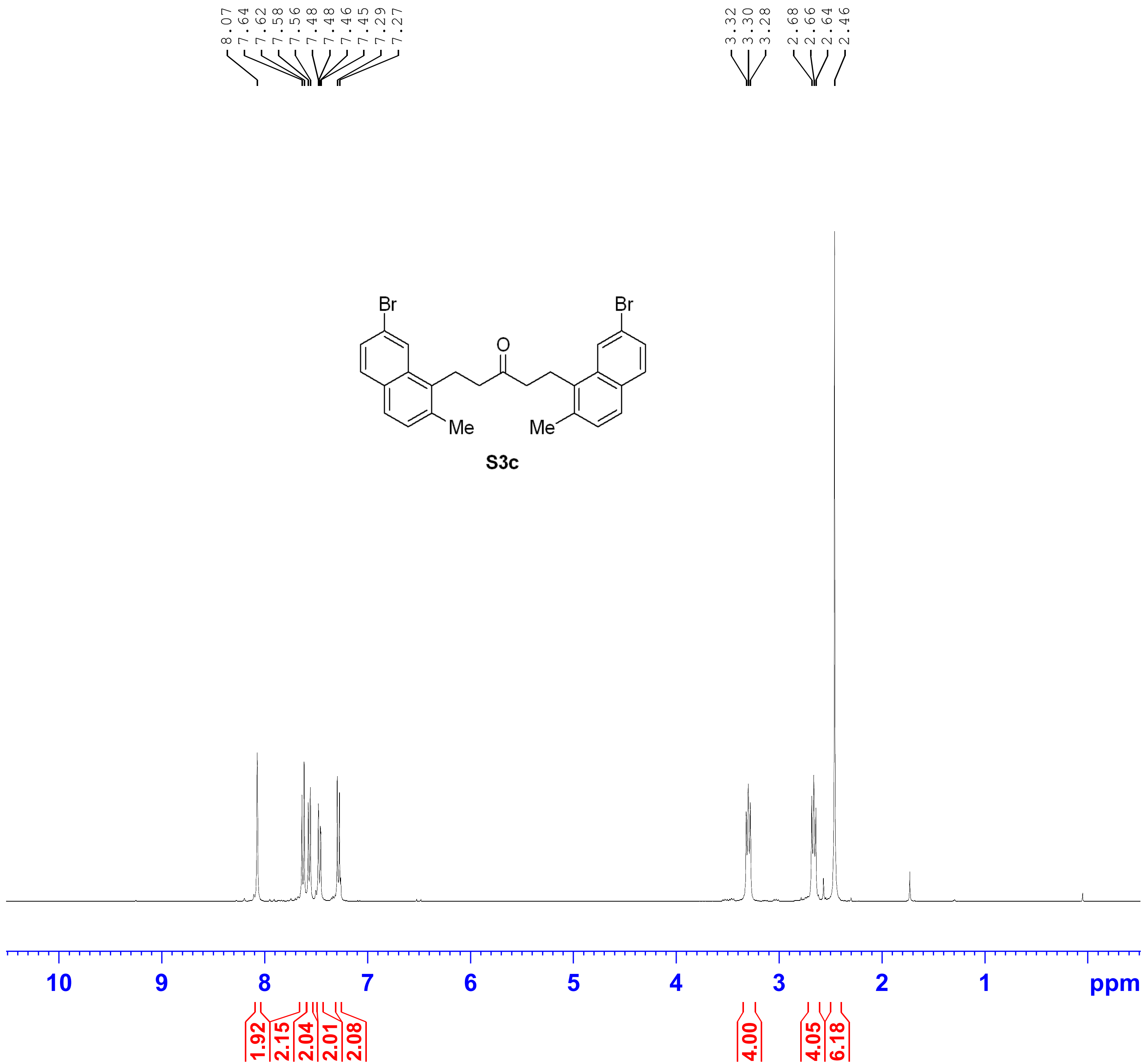

S3c

Current Data Parameters
NAME zrh-6-189-2-h
EXPNO 1
PROCNO 1

F2 - Acquisition Parameters
Date_ 20220629
Time 19.16
INSTRUM spect
PROBHD 5 mm DUL 13C-1
PULPROG zg30
TD 65536
SOLVENT CDCl3
NS 2
DS 0
SWH 8223.685 Hz
FIDRES 0.125483 Hz
AQ 3.9845889 sec
RG 64
DW 60.800 usec
DE 6.00 usec
TE 292.6 K
D1 1.00000000 sec
TD0 1

======== CHANNEL f1 ========
NUC1 1H
P1 15.80 usec
PL1 -1.00 dB
PL1W 12.17476940 W
SFO1 400.1324710 MHz

F2 - Processing parameters
SI 32768
SF 400.1300095 MHz
WDW EM
SSB 0
LB 0.30 Hz
GB 0
PC 1.00

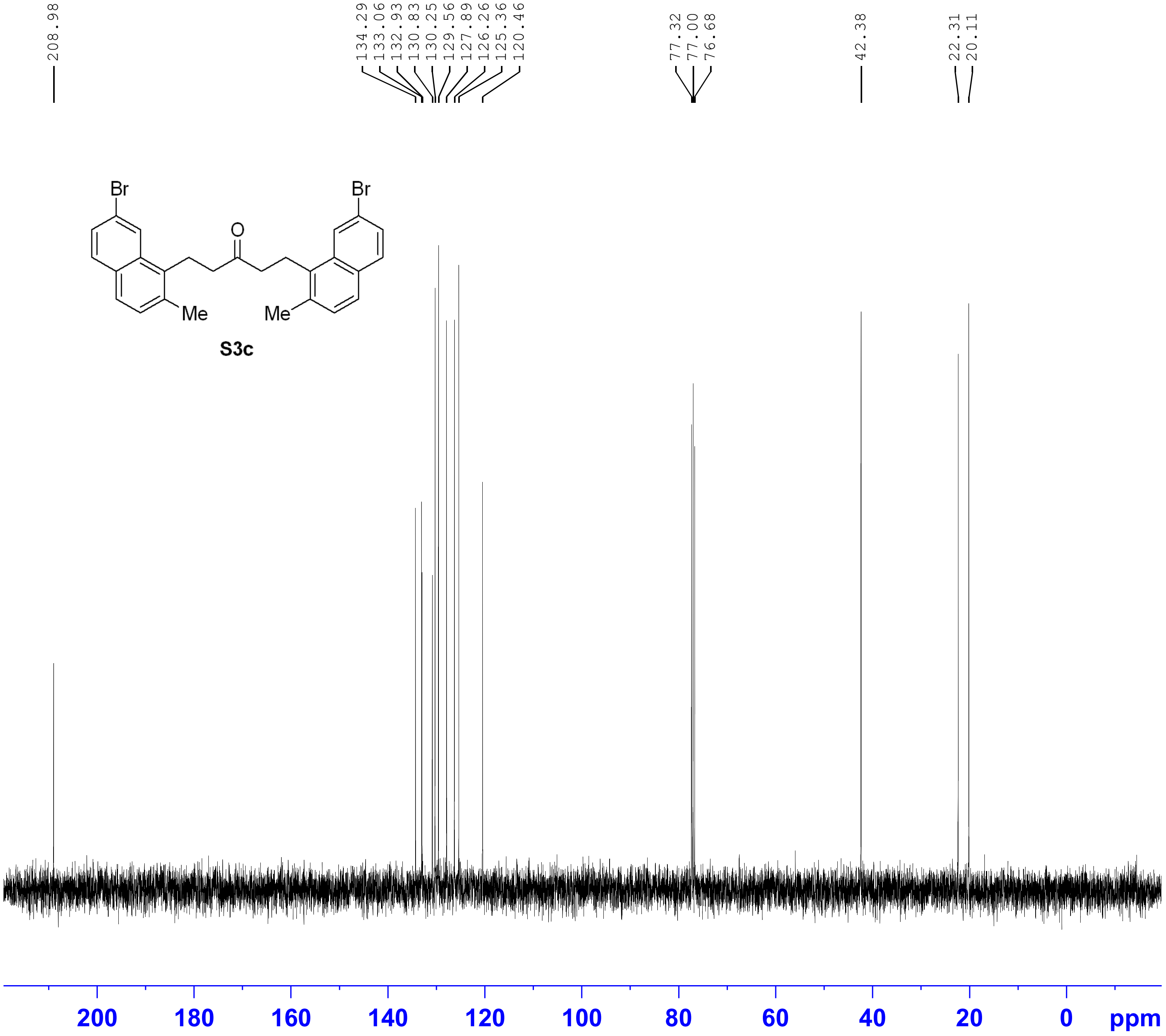

S3c

```
Current Data Parameters
NAME        zrh-6-189-2-c
EXPNO                   1
PROCNO                  1

F2 - Acquisition Parameters
Date_            20220629
Time                19.19
INSTRUM             spect
PROBHD    5 mm DUL 13C-1
PULPROG           zgpg30
TD                 65536
SOLVENT            CDCl3
NS                    22
DS                     0
SWH            24038.461 Hz
FIDRES          0.366798 Hz
AQ             1.3631488 sec
RG                  2050
DW                20.800 usec
DE                  6.00 usec
TE                 292.8 K
D1            2.00000000 sec
D11           0.03000000 sec
TD0                    1

======== CHANNEL f1 ========
NUC1                 13C
P1                 40.00 usec
PL1                -3.00 dB
PL1W         60.64365387 W
SFO1         100.6228298 MHz

======== CHANNEL f2 ========
CPDPRG[2         waltz16
NUC2                  1H
PCPD2              80.00 usec
PL2                -1.00 dB
PL12               14.39 dB
PL13               18.00 dB
PL2W         12.17476940 W
PL12W         0.35193357 W
PL13W         0.15327126 W
SFO2         400.1316005 MHz

F2 - Processing parameters
SI                 32768
SF           100.6127854 MHz
WDW                   EM
SSB                    0
LB                  1.00 Hz
GB                     0
PC                  1.40
```

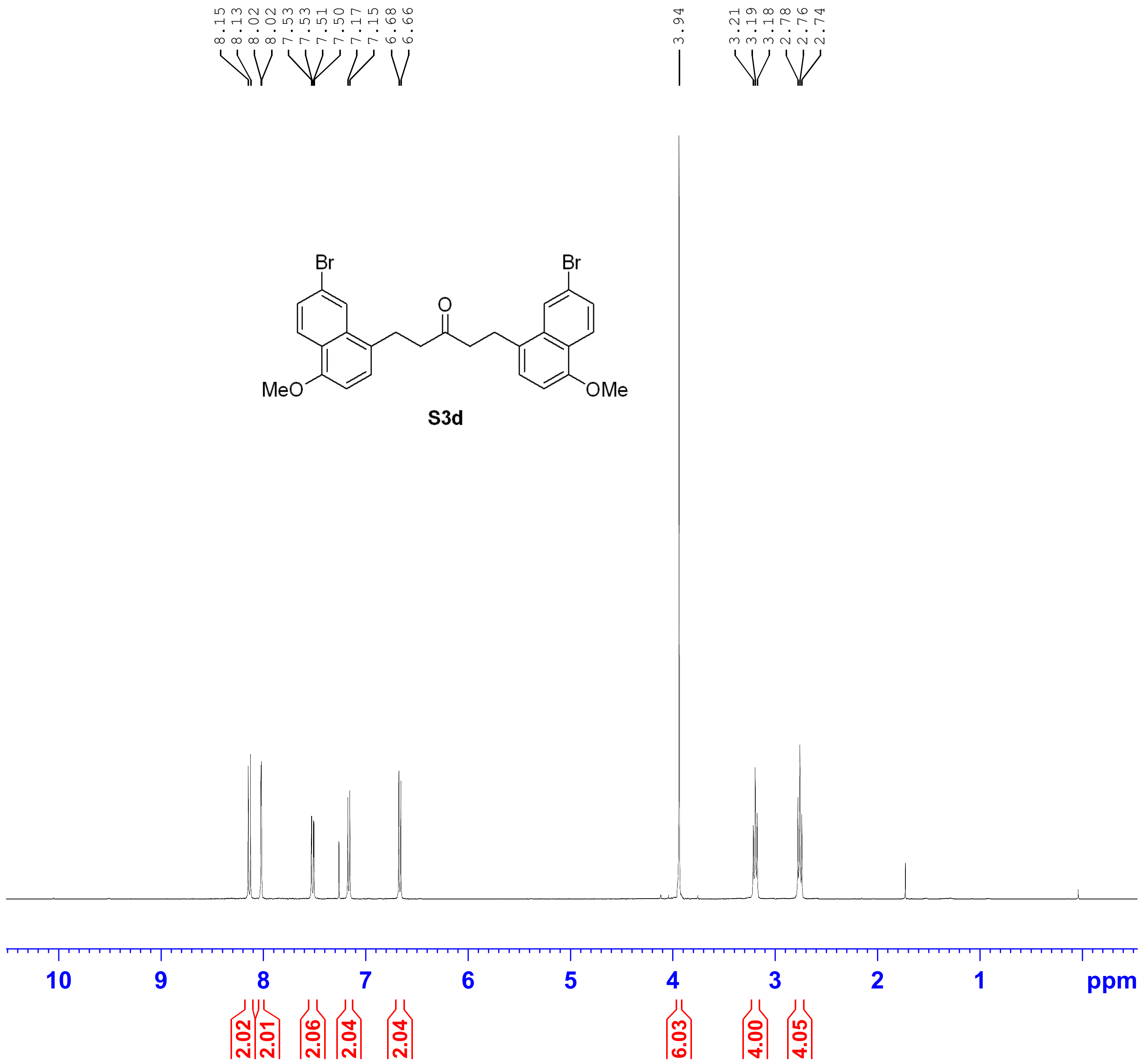


```
Current Data Parameters
NAME           zrh-7-50-h
EXPNO                   1
PROCNO                  1

F2 - Acquisition Parameters
Date_            20220724
Time                16.13
INSTRUM             spect
PROBHD    5 mm DUL 13C-1
PULPROG              zg30
TD                  65536
SOLVENT             CDCl3
NS                      1
DS                      0
SWH              8223.685 Hz
FIDRES           0.125483 Hz
AQ              3.9845889 sec
RG                    101
DW                 60.800 usec
DE                   6.00 usec
TE                  292.9 K
D1             1.00000000 sec
TD0                     1

======== CHANNEL f1 ========
NUC1                   1H
P1                  15.80 usec
PL1                 -1.00 dB
PL1W          12.17476940 W
SFO1          400.1324710 MHz

F2 - Processing parameters
SI                  32768
SF            400.1300096 MHz
WDW                    EM
SSB                     0
LB                   0.30 Hz
GB                      0
PC                   1.00
```

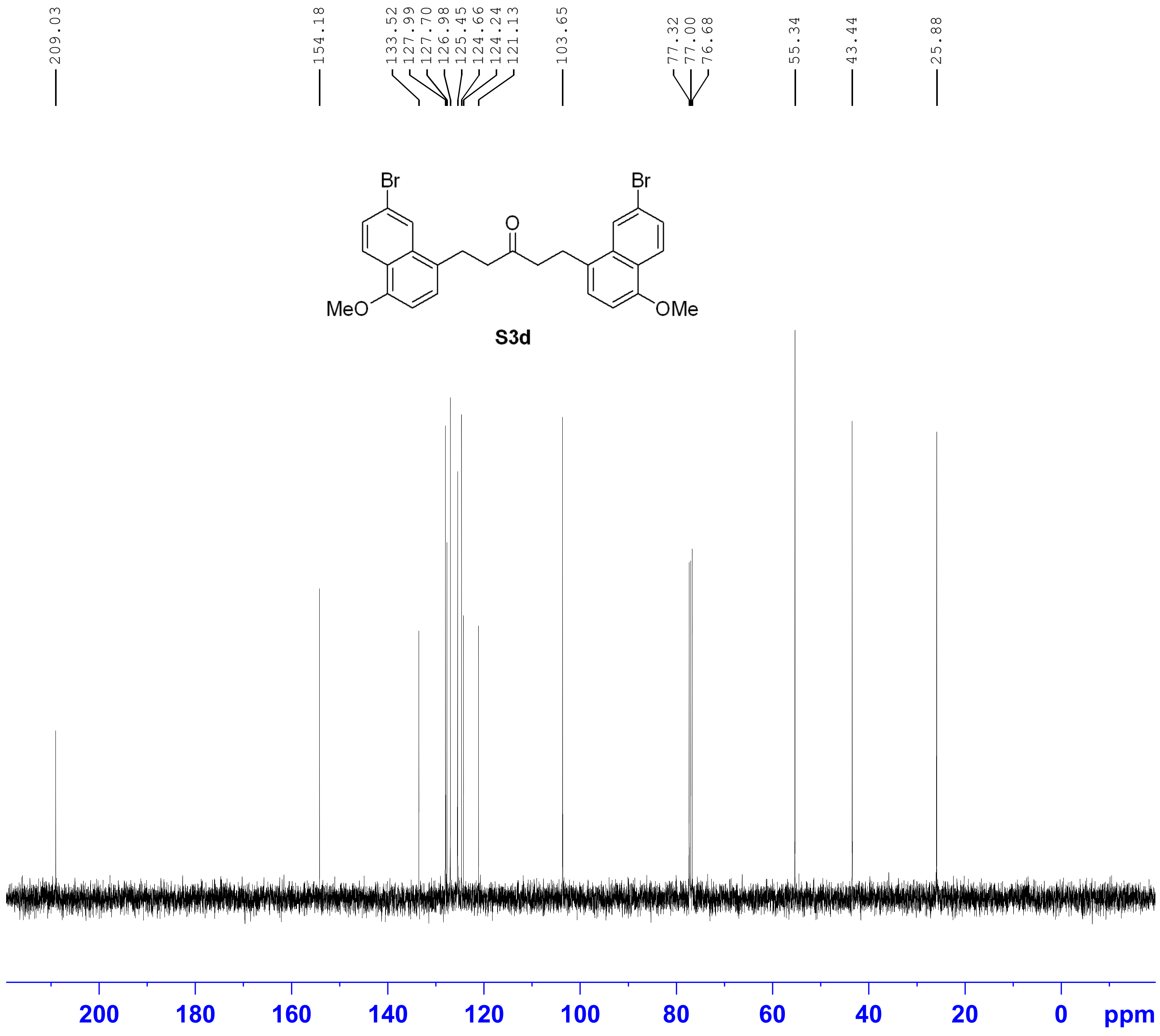


```
Current Data Parameters
NAME            zrh-7-50-c
EXPNO                    1
PROCNO                   1

F2 - Acquisition Parameters
Date_             20220724
Time                 16.14
INSTRUM              spect
PROBHD   5 mm DUL 13C-1
PULPROG            zgpg30
TD                   65536
SOLVENT              CDCl3
NS                      20
DS                       0
SWH              24038.461 Hz
FIDRES            0.366798 Hz
AQ               1.3631488 sec
RG                    2050
DW                  20.800 usec
DE                    6.00 usec
TE                   293.1 K
D1              2.00000000 sec
D11             0.03000000 sec
TD0                      1

======== CHANNEL f1 ========
NUC1                   13C
P1                   40.00 usec
PL1                  -3.00 dB
PL1W           60.64365387 W
SFO1          100.6228298 MHz

======== CHANNEL f2 ========
CPDPRG[2           waltz16
NUC2                    1H
PCPD2                80.00 usec
PL2                  -1.00 dB
PL12                 14.39 dB
PL13                 18.00 dB
PL2W           12.17476940 W
PL12W           0.35193357 W
PL13W           0.15327126 W
SFO2          400.1316005 MHz

F2 - Processing parameters
SI                   32768
SF            100.6127846 MHz
WDW                     EM
SSB                      0
LB                    1.00 Hz
GB                       0
PC                    1.40
```

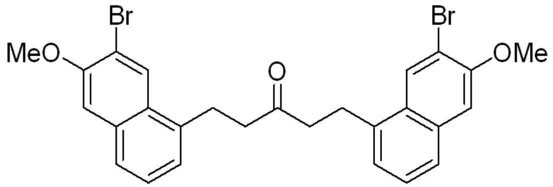

S3e

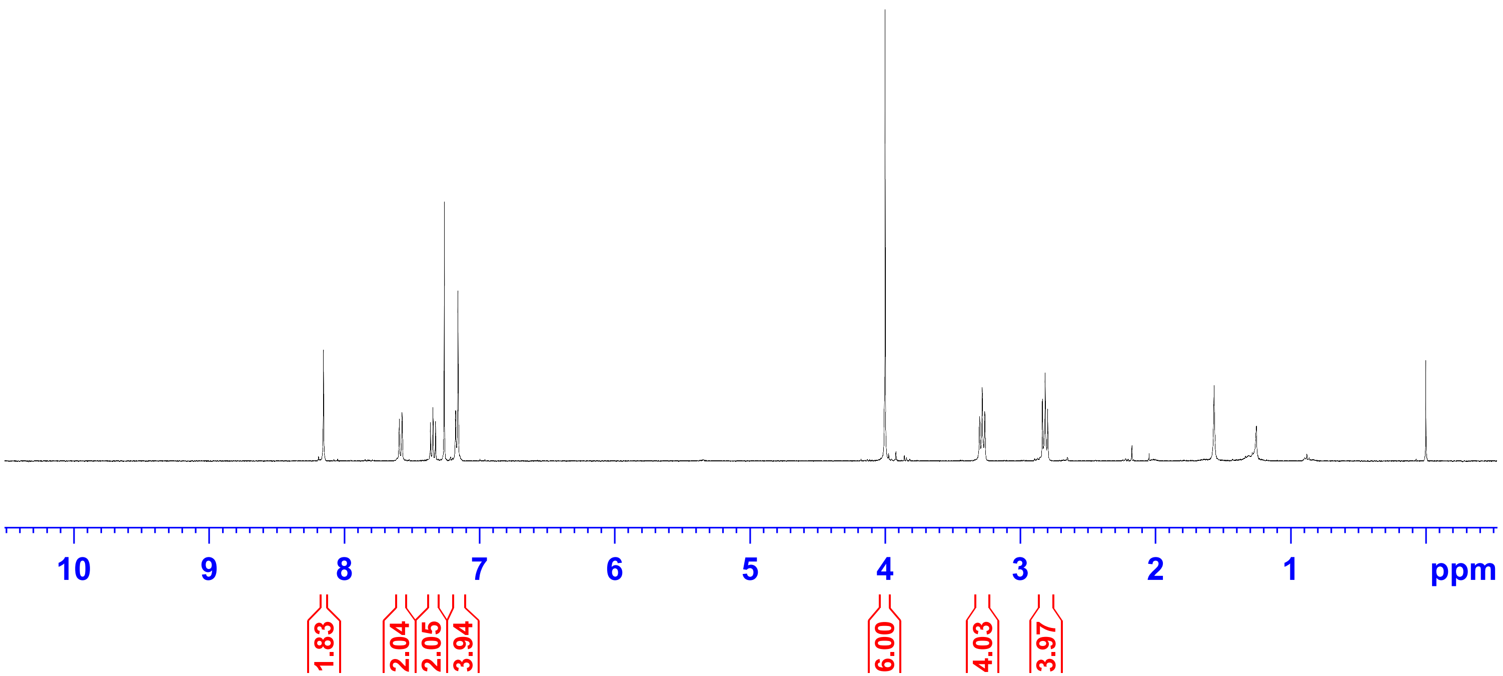

Current Data Parameters
NAME zrh-6-143-re-h
EXPNO 1
PROCNO 1

F2 - Acquisition Parameters
Date_ 20230131
Time 21.12
INSTRUM spect
PROBHD 5 mm DUL 13C-1
PULPROG zg30
TD 65536
SOLVENT CDCl3
NS 3
DS 0
SWH 8223.685 Hz
FIDRES 0.125483 Hz
AQ 3.9845889 sec
RG 645
DW 60.800 usec
DE 6.00 usec
TE 292.8 K
D1 1.00000000 sec
TD0 1

======== CHANNEL f1 ========
NUC1 1H
P1 15.80 usec
PL1 -1.00 dB
PL1W 12.17476940 W
SFO1 400.1324710 MHz

F2 - Processing parameters
SI 32768
SF 400.1300096 MHz
WDW EM
SSB 0
LB 0.30 Hz
GB 0
PC 1.00

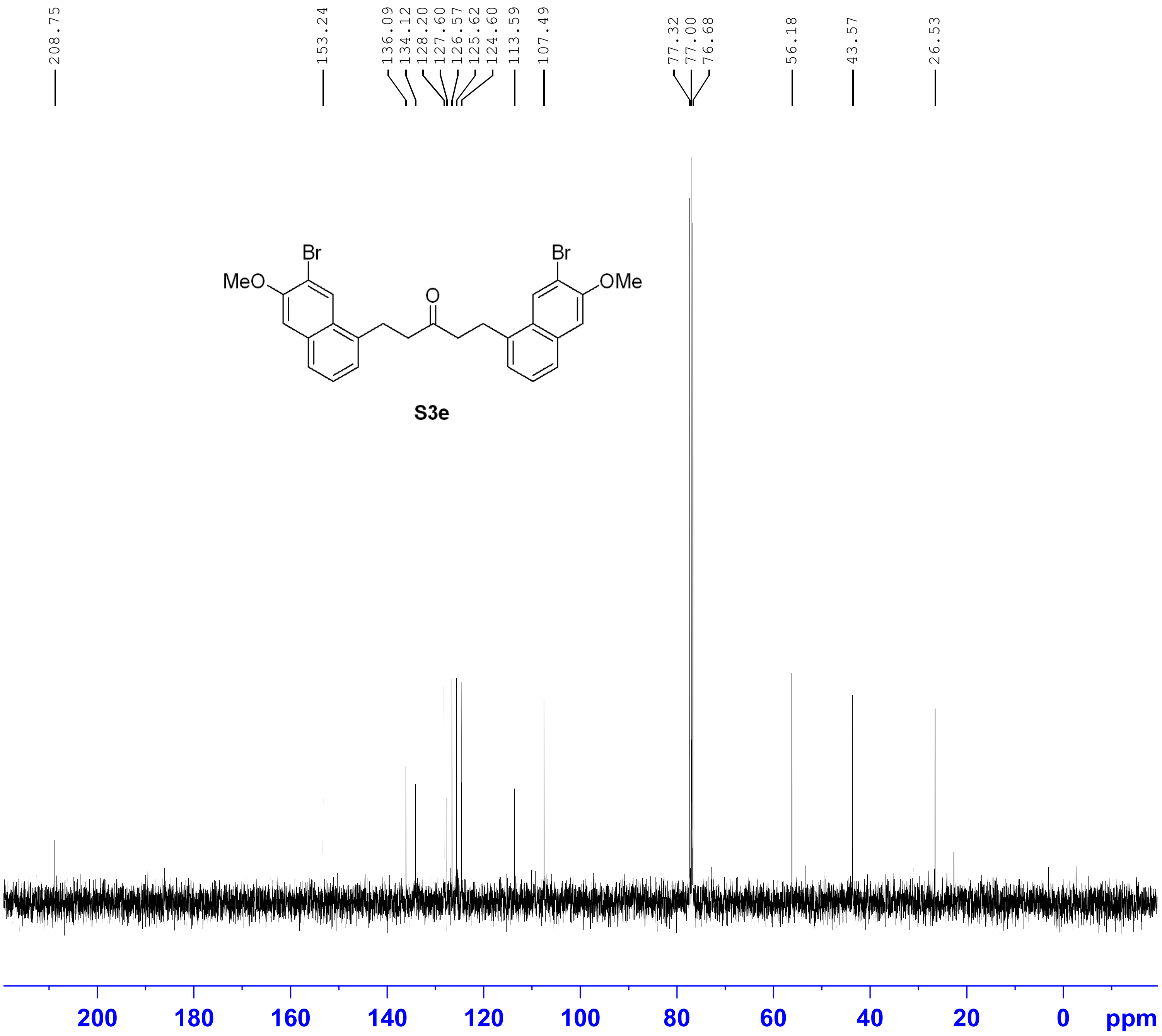


```
Current Data Parameters
NAME         zrh-6-143-c
EXPNO                  1
PROCNO                 1

F2 - Acquisition Parameters
Date_           20220611
Time               13.23
INSTRUM            spect
PROBHD    5 mm DUL 13C-1
PULPROG          zgpg30
TD                 65536
SOLVENT            CDCl3
NS                   104
DS                     0
SWH            24038.461 Hz
FIDRES          0.366798 Hz
AQ             1.3631488 sec
RG                  2050
DW                20.800 usec
DE                  6.00 usec
TE                 293.2 K
D1            2.00000000 sec
D11           0.03000000 sec
TD0                    1

======== CHANNEL f1 ========
NUC1                 13C
P1                 40.00 usec
PL1                -3.00 dB
PL1W         60.64365387 W
SFO1         100.6228298 MHz

======== CHANNEL f2 ========
CPDPRG[2         waltz16
NUC2                  1H
PCPD2              80.00 usec
PL2                -1.00 dB
PL12               14.39 dB
PL13               18.00 dB
PL2W         12.17476940 W
PL12W         0.35193357 W
PL13W         0.15327126 W
SFO2         400.1316005 MHz

F2 - Processing parameters
SI                 32768
SF           100.6127744 MHz
WDW                   EM
SSB                    0
LB                  1.00 Hz
GB                     0
PC                  1.40
```

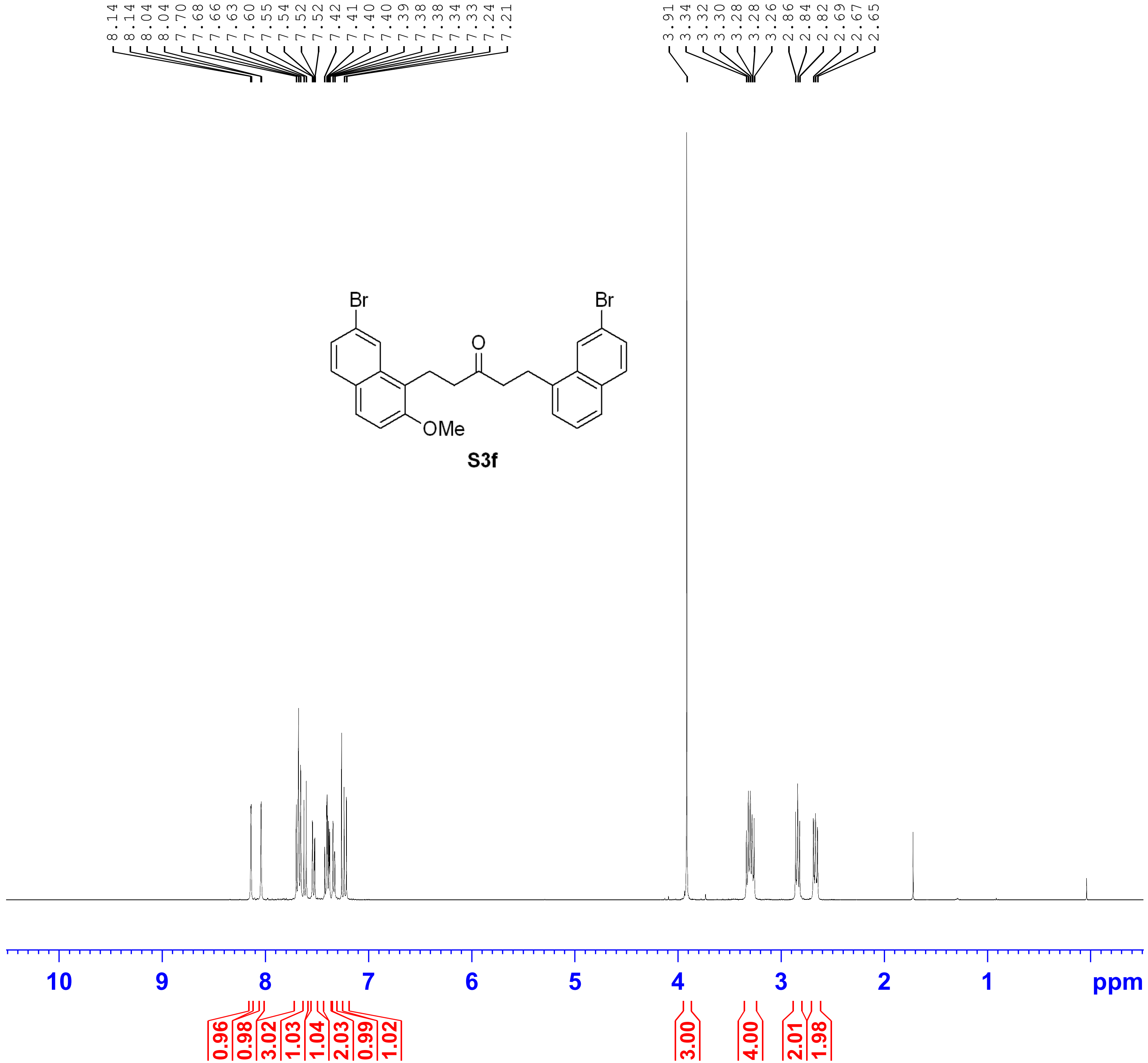

Current Data Parameters
NAME zrh-6-171-h
EXPNO 1
PROCNO 1

F2 - Acquisition Parameters
Date_ 20220622
Time 22.26
INSTRUM spect
PROBHD 5 mm DUL 13C-1
PULPROG zg30
TD 65536
SOLVENT CDCl3
NS 2
DS 0
SWH 8223.685 Hz
FIDRES 0.125483 Hz
AQ 3.9845889 sec
RG 144
DW 60.800 usec
DE 6.00 usec
TE 294.1 K
D1 1.00000000 sec
TD0 1

======== CHANNEL f1 ========
NUC1 1H
P1 15.80 usec
PL1 -1.00 dB
PL1W 12.17476940 W
SFO1 400.1324710 MHz

F2 - Processing parameters
SI 32768
SF 400.1300096 MHz
WDW EM
SSB 0
LB 0.30 Hz
GB 0
PC 1.00

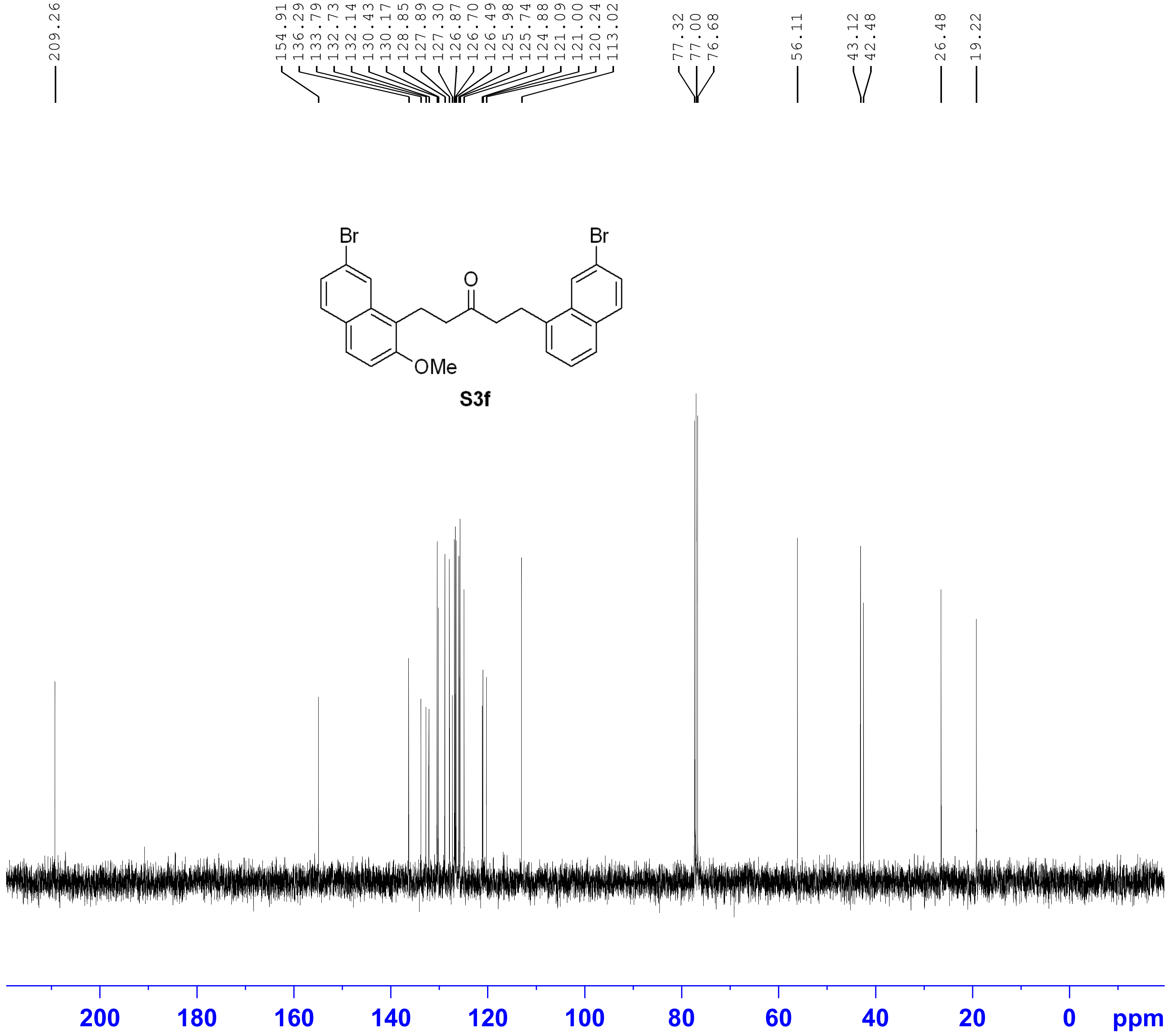

S3f

Current Data Parameters	
NAME	zrh-6-171-c
EXPNO	1
PROCNO	1
F2 - Acquisition Parameters	
Date_	20220622
Time	22.29
INSTRUM	spect
PROBHD	5 mm DUL 13C-1
PULPROG	zgpg30
TD	65536
SOLVENT	CDCl3
NS	34
DS	0
SWH	24038.461 Hz
FIDRES	0.366798 Hz
AQ	1.3631488 sec
RG	2050
DW	20.800 usec
DE	6.00 usec
TE	294.4 K
D1	2.00000000 sec
D11	0.03000000 sec
TD0	1
======== CHANNEL f1 ========	
NUC1	13C
P1	40.00 usec
PL1	-3.00 dB
PL1W	60.64365387 W
SFO1	100.6228298 MHz
======== CHANNEL f2 ========	
CPDPRG[2	waltz16
NUC2	1H
PCPD2	80.00 usec
PL2	-1.00 dB
PL12	14.39 dB
PL13	18.00 dB
PL2W	12.17476940 W
PL12W	0.35193357 W
PL13W	0.15327126 W
SFO2	400.1316005 MHz
F2 - Processing parameters	
SI	32768
SF	100.6127832 MHz
WDW	EM
SSB	0
LB	1.00 Hz
GB	0
PC	1.40

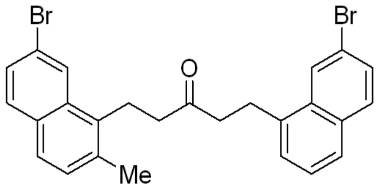

S3g

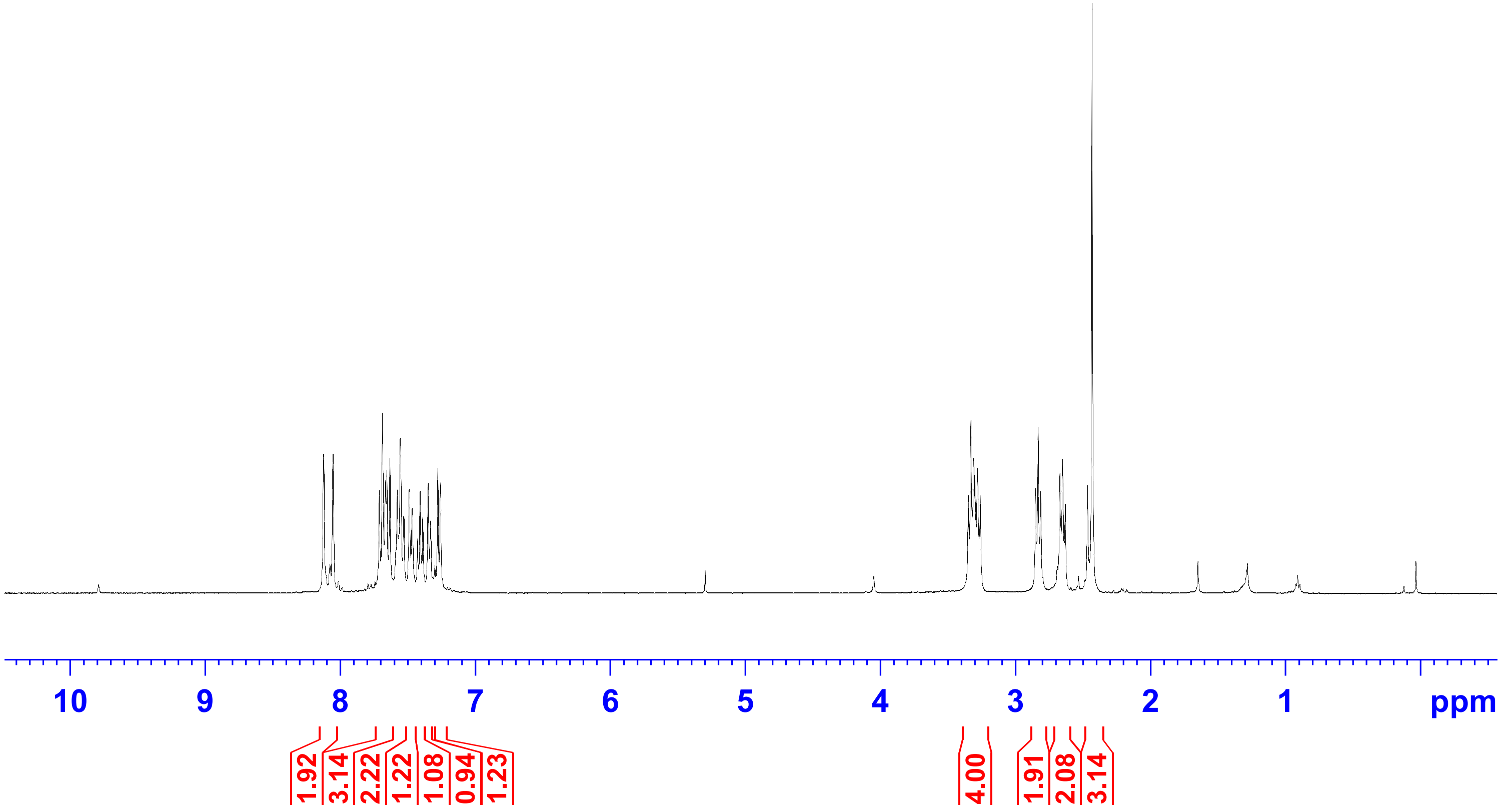

Current Data Parameters
NAME zrh-8-92-2-h
EXPNO 1
PROCNO 1

F2 - Acquisition Parameters
Date_ 20221221
Time 19.37
INSTRUM spect
PROBHD 5 mm DUL 13C-1
PULPROG zg30
TD 65536
SOLVENT CDCl3
NS 2
DS 0
SWH 8223.685 Hz
FIDRES 0.125483 Hz
AQ 3.9845889 sec
RG 144
DW 60.800 usec
DE 6.00 usec
TE 292.6 K
D1 1.00000000 sec
TD0 1

======== CHANNEL f1 ========
NUC1 1H
P1 15.80 usec
PL1 -1.00 dB
PL1W 12.17476940 W
SFO1 400.1324710 MHz

F2 - Processing parameters
SI 32768
SF 400.1300095 MHz
WDW EM
SSB 0
LB 0.30 Hz
GB 0
PC 1.00

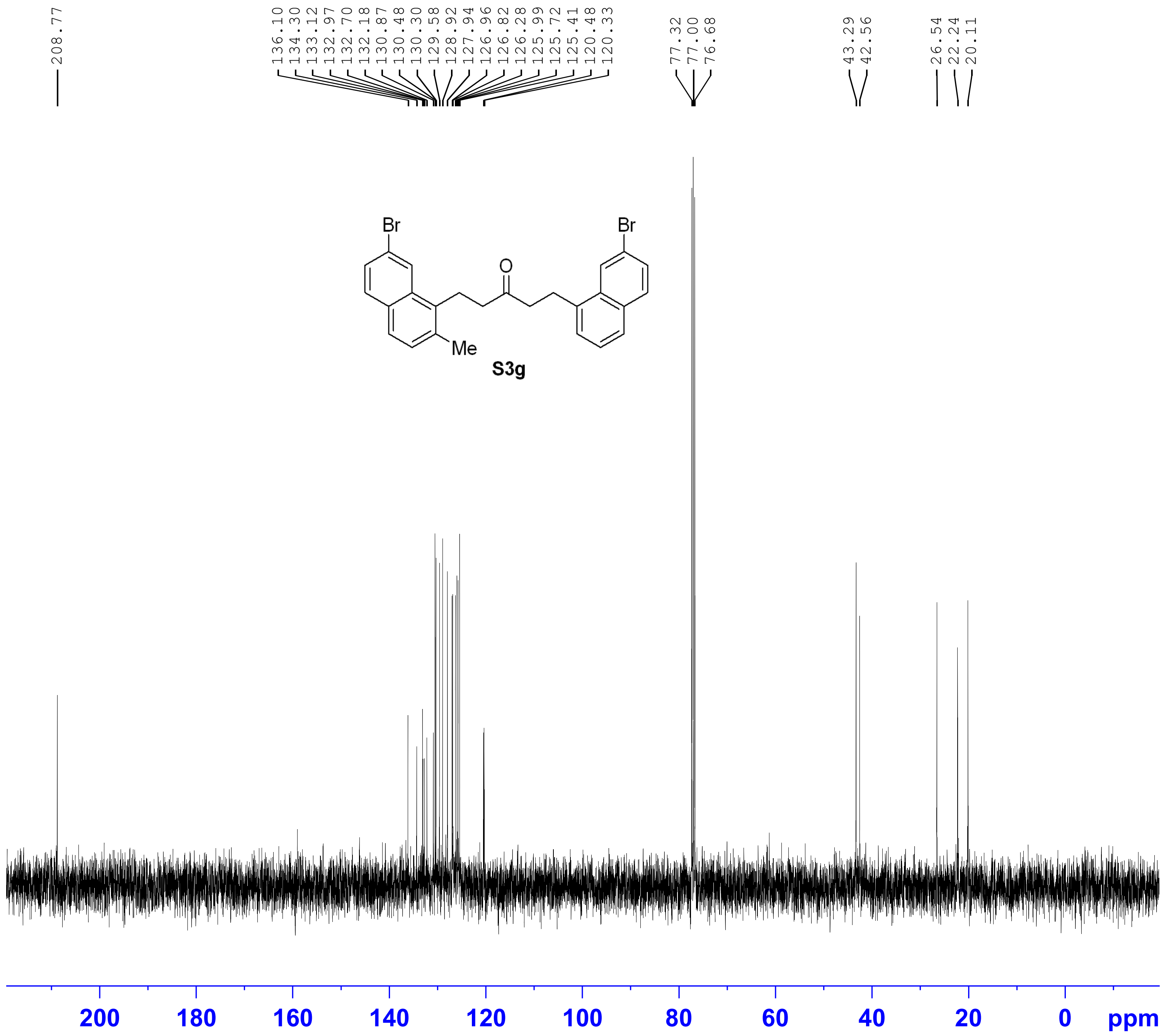

Current Data Parameters
NAME zrh-8-92-2-c
EXPNO 1
PROCNO 1

F2 - Acquisition Parameters
Date_ 20221221
Time 19.39
INSTRUM spect
PROBHD 5 mm DUL 13C-1
PULPROG zgpg30
TD 65536
SOLVENT CDCl3
NS 40
DS 0
SWH 24038.461 Hz
FIDRES 0.366798 Hz
AQ 1.3631488 sec
RG 2050
DW 20.800 usec
DE 6.00 usec
TE 292.9 K
D1 2.00000000 sec
D11 0.03000000 sec
TD0 1

======== CHANNEL f1 ========
NUC1 13C
P1 40.00 usec
PL1 -3.00 dB
PL1W 60.64365387 W
SFO1 100.6228298 MHz

======== CHANNEL f2 ========
CPDPRG[2 waltz16
NUC2 1H
PCPD2 80.00 usec
PL2 -1.00 dB
PL12 14.39 dB
PL13 18.00 dB
PL2W 12.17476940 W
PL12W 0.35193357 W
PL13W 0.15327126 W
SFO2 400.1316005 MHz

F2 - Processing parameters
SI 32768
SF 100.6127802 MHz
WDW EM
SSB 0
LB 1.00 Hz
GB 0
PC 1.40

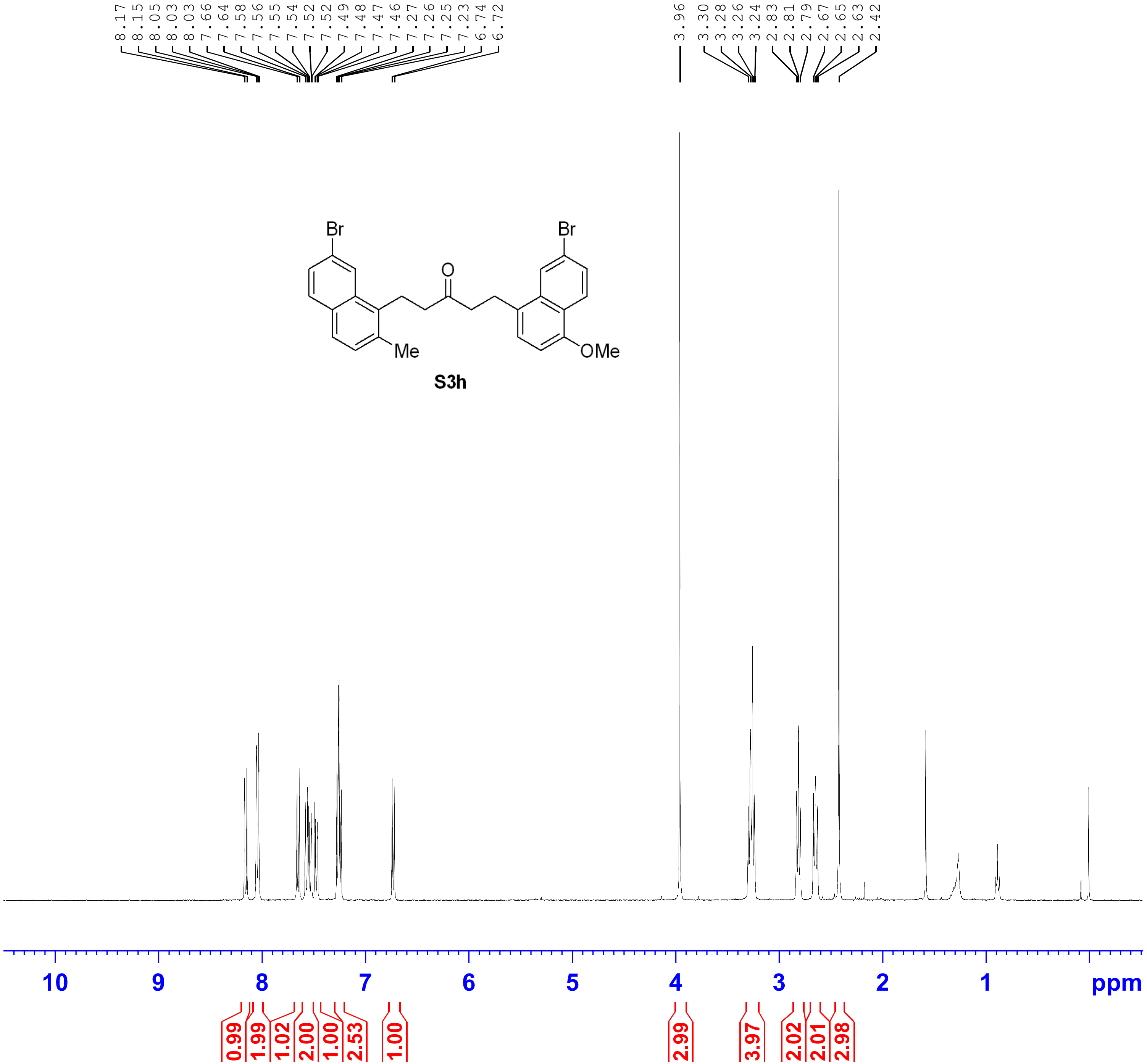


```
Current Data Parameters
NAME      zrh-8-92-1-1-h
EXPNO                  1
PROCNO                 1

F2 - Acquisition Parameters
Date_           20221219
Time               21.07
INSTRUM            spect
PROBHD    5 mm DUL 13C-1
PULPROG             zg30
TD                 65536
SOLVENT            CDCl3
NS                     6
DS                     0
SWH             8223.685 Hz
FIDRES          0.125483 Hz
AQ             3.9845889 sec
RG                   362
DW                60.800 usec
DE                  6.00 usec
TE                 292.7 K
D1            1.00000000 sec
TD0                    1

======== CHANNEL f1 ========
NUC1                  1H
P1                 15.80 usec
PL1                -1.00 dB
PL1W         12.17476940 W
SFO1         400.1324710 MHz

F2 - Processing parameters
SI                 32768
SF           400.1300094 MHz
WDW                   EM
SSB                    0
LB                  0.30 Hz
GB                     0
PC                  1.00
```

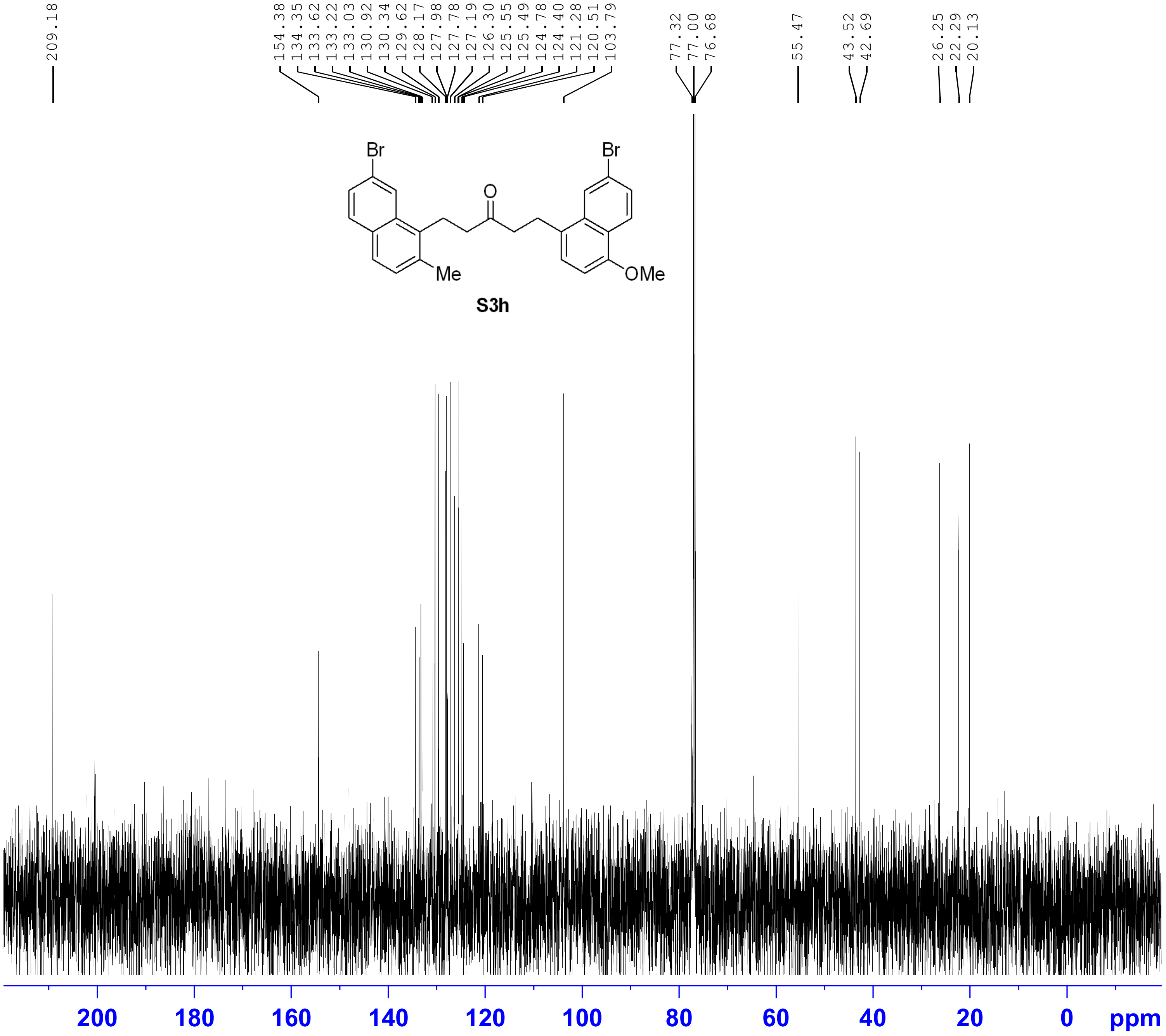

Current Data Parameters
NAME zrh-8-92-1-1-c
EXPNO 1
PROCNO 1

F2 - Acquisition Parameters
Date_ 20221219
Time 21.11
INSTRUM spect
PROBHD 5 mm DUL 13C-1
PULPROG zgpg30
TD 65536
SOLVENT CDCl3
NS 91
DS 0
SWH 24038.461 Hz
FIDRES 0.366798 Hz
AQ 1.3631488 sec
RG 2050
DW 20.800 usec
DE 6.00 usec
TE 293.1 K
D1 2.00000000 sec
D11 0.03000000 sec
TD0 1

======== CHANNEL f1 ========
NUC1 13C
P1 40.00 usec
PL1 -3.00 dB
PL1W 60.64365387 W
SFO1 100.6228298 MHz

======== CHANNEL f2 ========
CPDPRG[2 waltz16
NUC2 1H
PCPD2 80.00 usec
PL2 -1.00 dB
PL12 14.39 dB
PL13 18.00 dB
PL2W 12.17476940 W
PL12W 0.35193357 W
PL13W 0.15327126 W
SFO2 400.1316005 MHz

F2 - Processing parameters
SI 32768
SF 100.6127736 MHz
WDW EM
SSB 0
LB 1.00 Hz
GB 0
PC 1.40

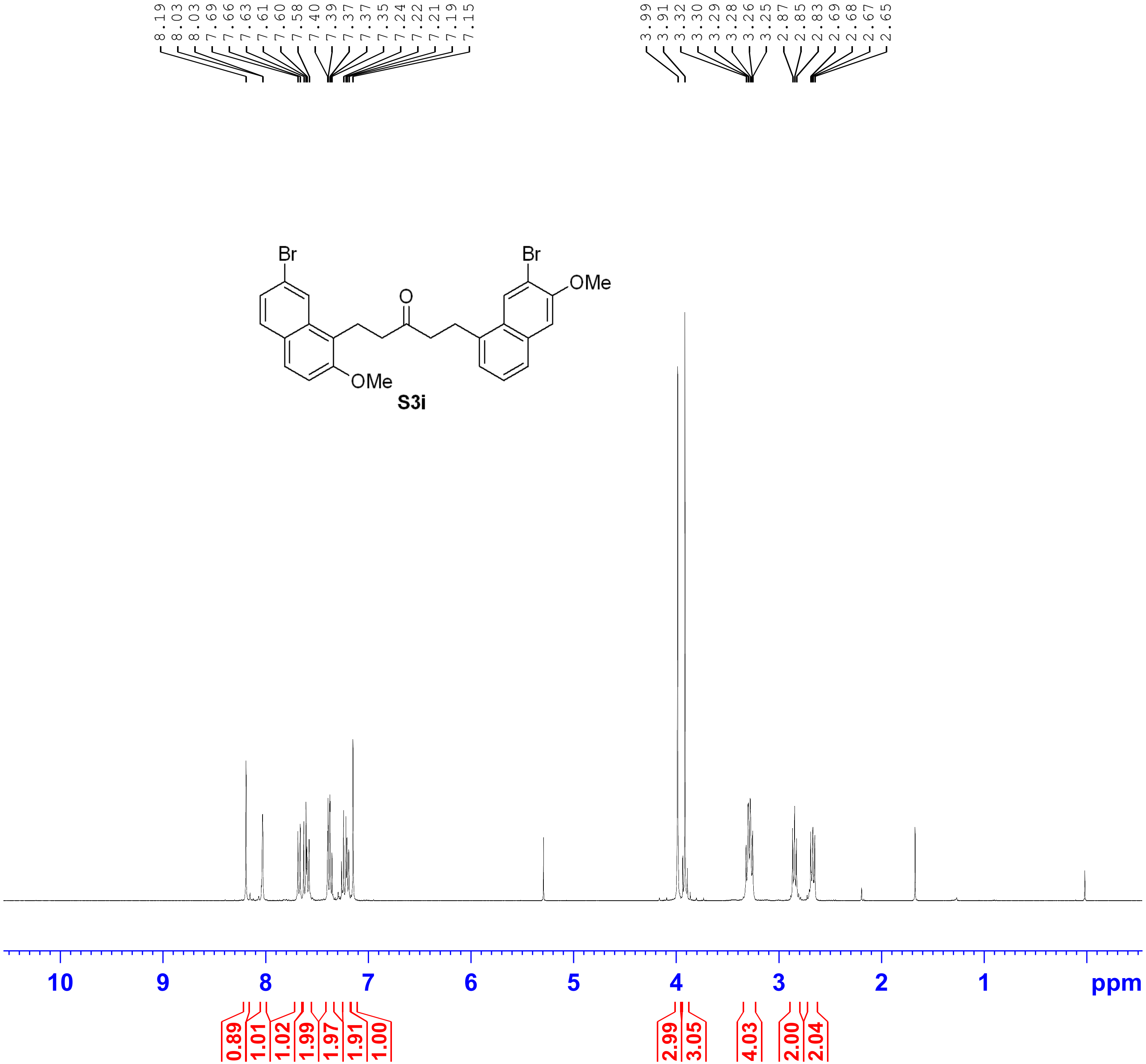


```
Current Data Parameters
NAME        zrh-6-169-1-h
EXPNO                   1
PROCNO                  1

F2 - Acquisition Parameters
Date_            20220621
Time                14.26
INSTRUM             spect
PROBHD    5 mm DUL 13C-1
PULPROG              zg30
TD                  65536
SOLVENT             CDCl3
NS                      2
DS                      0
SWH              8223.685 Hz
FIDRES           0.125483 Hz
AQ              3.9845889 sec
RG                    181
DW                 60.800 usec
DE                   6.00 usec
TE                  293.0 K
D1             1.00000000 sec
TD0                     1

======== CHANNEL f1 ========
NUC1                   1H
P1                  15.80 usec
PL1                 -1.00 dB
PL1W          12.17476940 W
SFO1          400.1324710 MHz

F2 - Processing parameters
SI                  32768
SF            400.1300096 MHz
WDW                    EM
SSB                     0
LB                   0.30 Hz
GB                      0
PC                   1.00
```

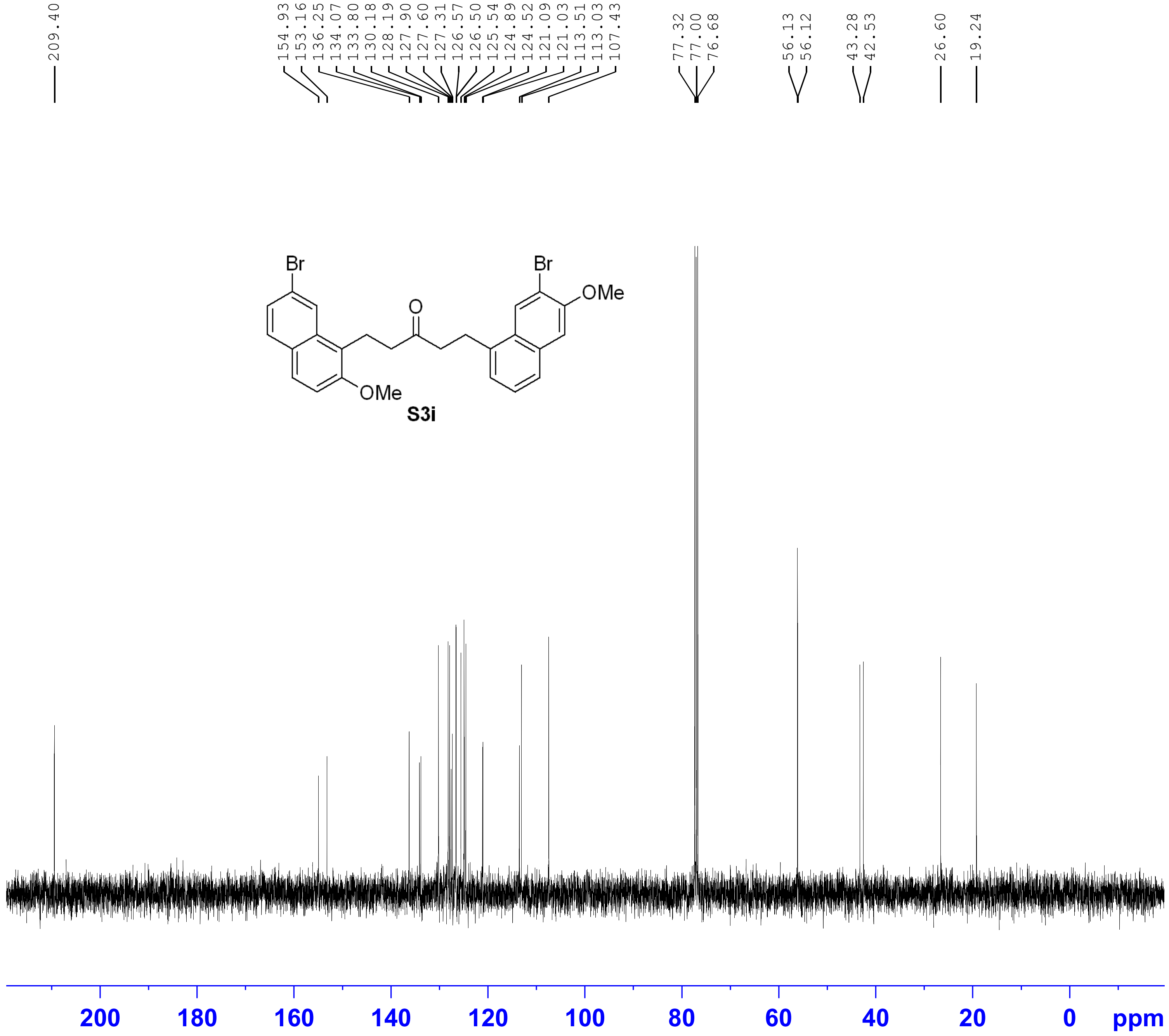


```
Current Data Parameters
NAME      zrh-6-169-1-re-c
EXPNO                    1
PROCNO                   1

F2 - Acquisition Parameters
Date_             20220621
Time                 14.28
INSTRUM              spect
PROBHD    5 mm DUL 13C-1
PULPROG            zgpg30
TD                   65536
SOLVENT              CDCl3
NS                      50
DS                       0
SWH           24038.461 Hz
FIDRES         0.366798 Hz
AQ            1.3631488 sec
RG                    2050
DW              20.800 usec
DE                6.00 usec
TE               293.1 K
D1          2.00000000 sec
D11         0.03000000 sec
TD0                      1

======== CHANNEL f1 ========
NUC1                   13C
P1               40.00 usec
PL1              -3.00 dB
PL1W       60.64365387 W
SFO1       100.6228298 MHz

======== CHANNEL f2 ========
CPDPRG[2           waltz16
NUC2                    1H
PCPD2            80.00 usec
PL2              -1.00 dB
PL12             14.39 dB
PL13             18.00 dB
PL2W       12.17476940 W
PL12W       0.35193357 W
PL13W       0.15327126 W
SFO2       400.1316005 MHz

F2 - Processing parameters
SI                   32768
SF         100.6127802 MHz
WDW                     EM
SSB                      0
LB                1.00 Hz
GB                       0
PC                    1.40
```

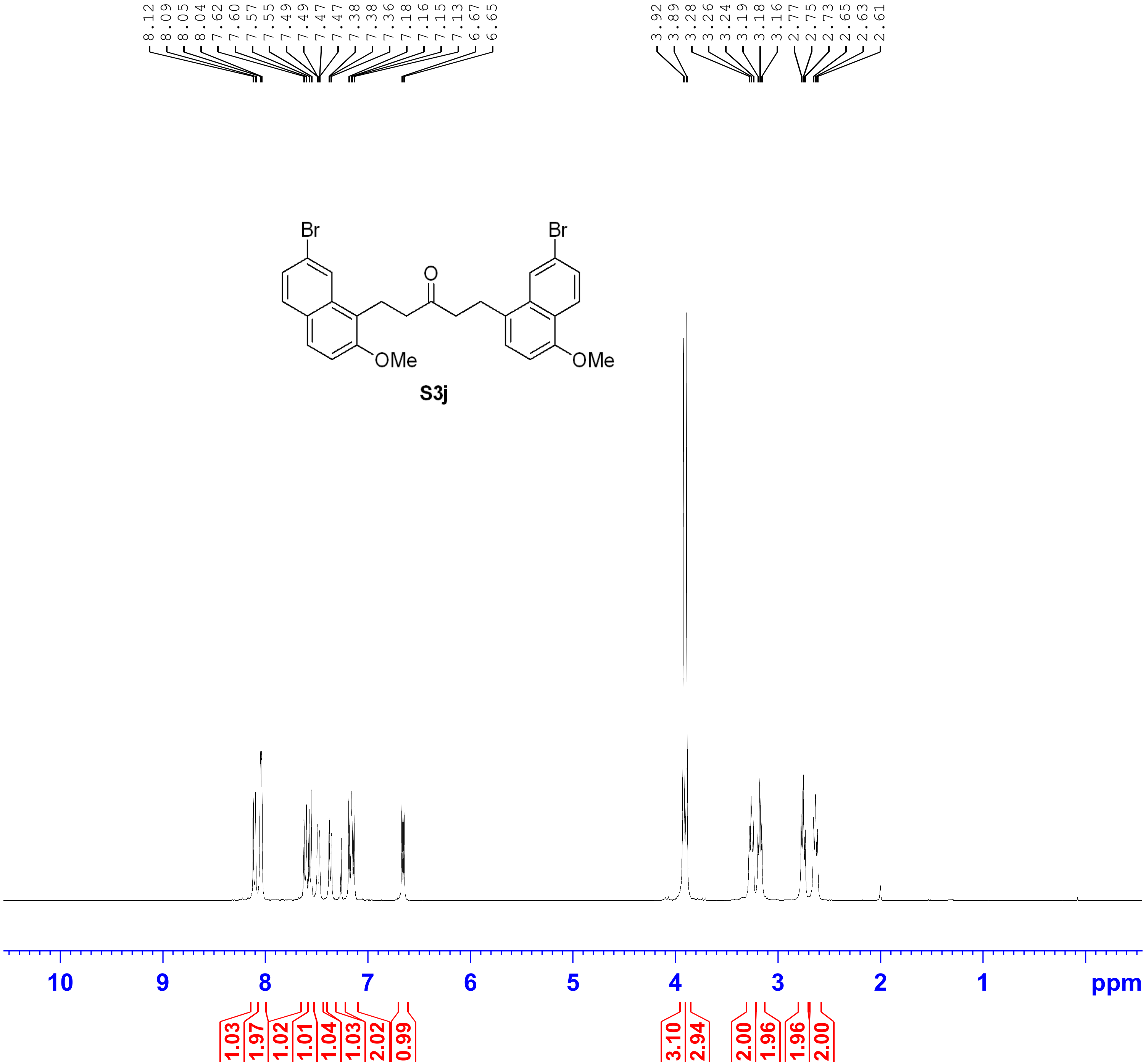

Current Data Parameters
NAME zrh-7-54-h
EXPNO 1
PROCNO 1

F2 - Acquisition Parameters
Date_ 20220727
Time 20.23
INSTRUM spect
PROBHD 5 mm DUL 13C-1
PULPROG zg30
TD 65536
SOLVENT CDCl3
NS 2
DS 0
SWH 8223.685 Hz
FIDRES 0.125483 Hz
AQ 3.9845889 sec
RG 40.3
DW 60.800 usec
DE 6.00 usec
TE 292.8 K
D1 1.00000000 sec
TD0 1

======== CHANNEL f1 ========
NUC1 1H
P1 15.80 usec
PL1 -1.00 dB
PL1W 12.17476940 W
SFO1 400.1324710 MHz

F2 - Processing parameters
SI 32768
SF 400.1300091 MHz
WDW EM
SSB 0
LB 0.30 Hz
GB 0
PC 1.00

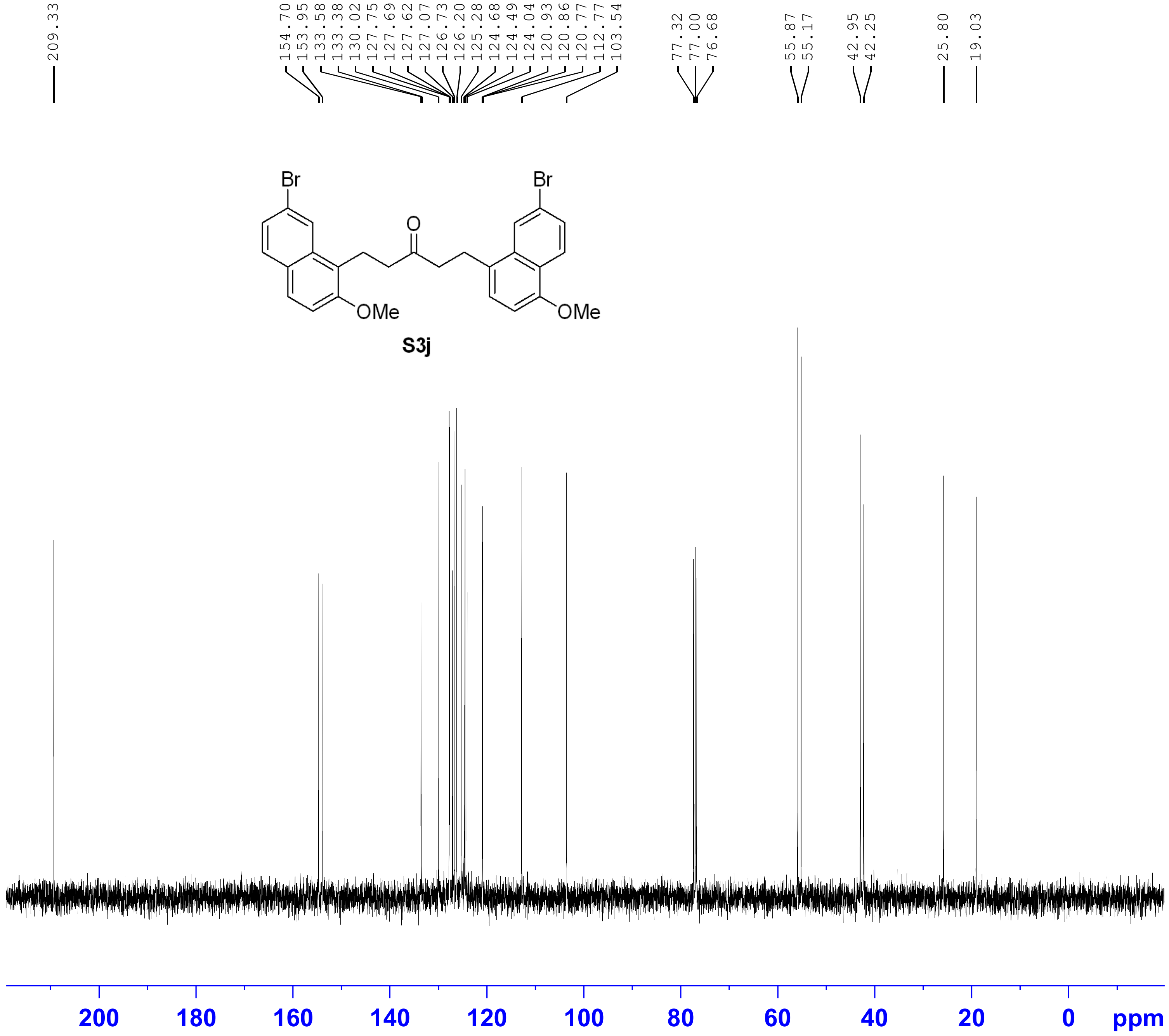


```
Current Data Parameters
NAME            zrh-7-54-c
EXPNO                    1
PROCNO                   1

F2 - Acquisition Parameters
Date_             20220727
Time                 20.25
INSTRUM              spect
PROBHD   5 mm DUL 13C-1
PULPROG            zgpg30
TD                   65536
SOLVENT              CDCl3
NS                      27
DS                       0
SWH              24038.461 Hz
FIDRES            0.366798 Hz
AQ               1.3631488 sec
RG                    2050
DW                  20.800 usec
DE                    6.00 usec
TE                   293.0 K
D1              2.00000000 sec
D11             0.03000000 sec
TD0                      1

======== CHANNEL f1 ========
NUC1                   13C
P1                   40.00 usec
PL1                  -3.00 dB
PL1W           60.64365387 W
SFO1           100.6228298 MHz

======== CHANNEL f2 ========
CPDPRG[2           waltz16
NUC2                    1H
PCPD2                80.00 usec
PL2                  -1.00 dB
PL12                 14.39 dB
PL13                 18.00 dB
PL2W           12.17476940 W
PL12W           0.35193357 W
PL13W           0.15327126 W
SFO2           400.1316005 MHz

F2 - Processing parameters
SI                   32768
SF             100.6128023 MHz
WDW                     EM
SSB                      0
LB                    1.00 Hz
GB                       0
PC                    1.40
```

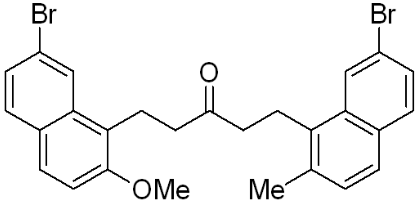

S3k

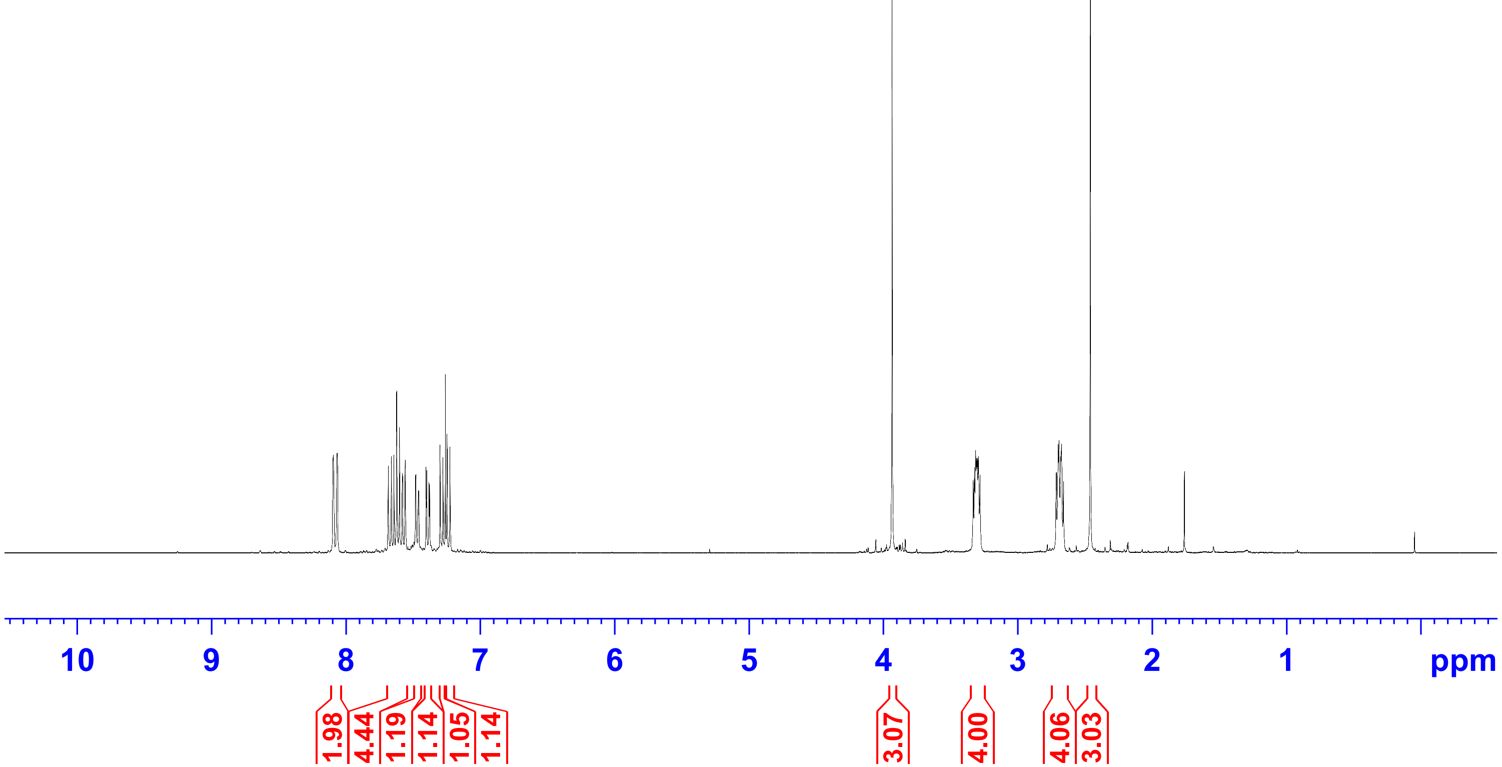

Current Data Parameters
NAME zrh-6-189-1-h
EXPNO 1
PROCNO 1

F2 - Acquisition Parameters
Date_ 20220629
Time 15.44
INSTRUM spect
PROBHD 5 mm DUL 13C-1
PULPROG zg30
TD 65536
SOLVENT CDCl3
NS 1
DS 0
SWH 8223.685 Hz
FIDRES 0.125483 Hz
AQ 3.9845889 sec
RG 64
DW 60.800 usec
DE 6.00 usec
TE 292.8 K
D1 1.00000000 sec
TD0 1

======== CHANNEL f1 ========
NUC1 1H
P1 15.80 usec
PL1 -1.00 dB
PL1W 12.17476940 W
SFO1 400.1324710 MHz

F2 - Processing parameters
SI 32768
SF 400.1300096 MHz
WDW EM
SSB 0
LB 0.30 Hz
GB 0
PC 1.00

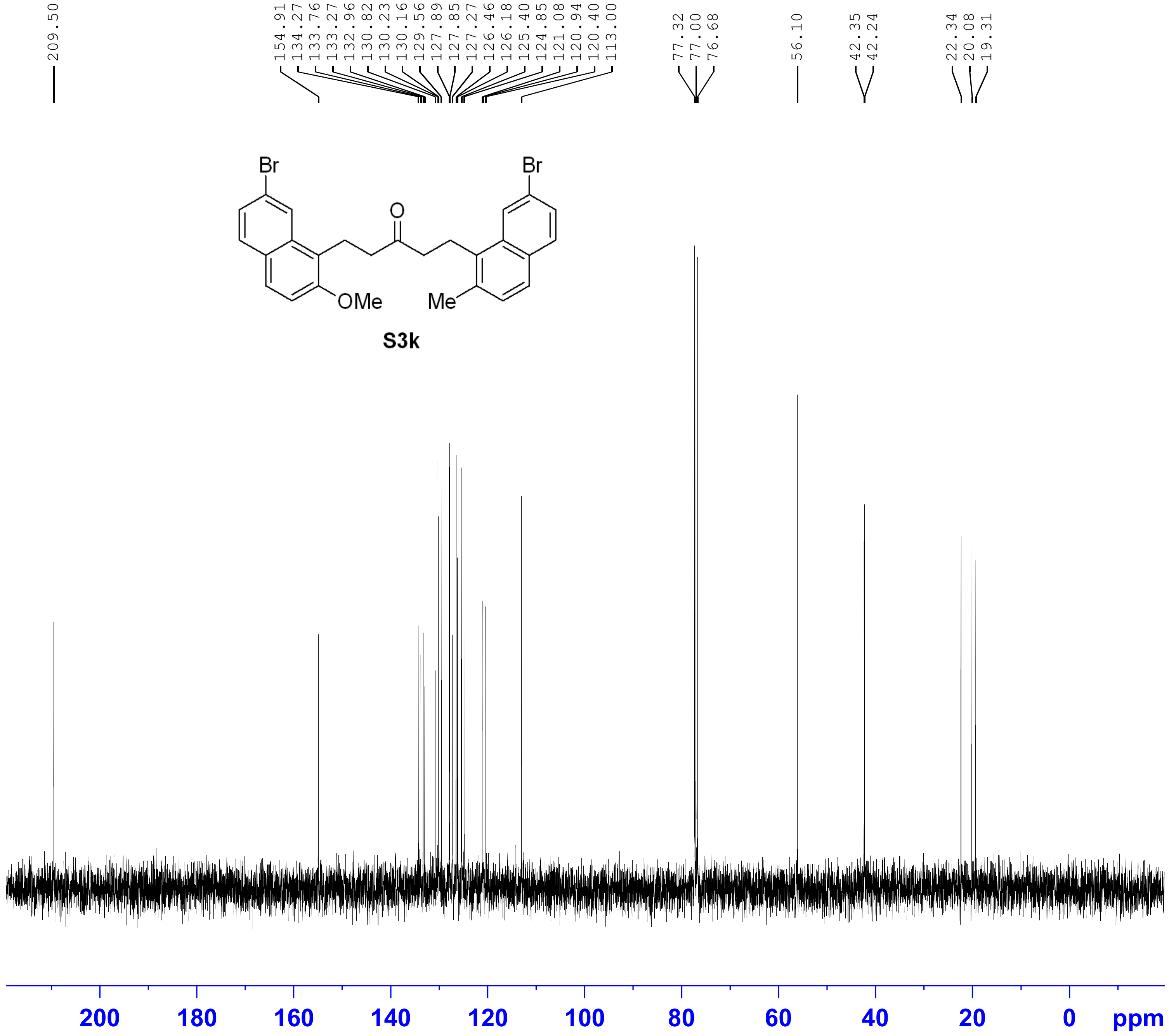


```
Current Data Parameters
NAME          zrh-6-189-1-c
EXPNO                     1
PROCNO                    1

F2 - Acquisition Parameters
Date_              20220629
Time                  15.46
INSTRUM               spect
PROBHD     5 mm DUL 13C-1
PULPROG             zgpg30
TD                    65536
SOLVENT               CDCl3
NS                       28
DS                        0
SWH               24038.461 Hz
FIDRES             0.366798 Hz
AQ                1.3631488 sec
RG                     2050
DW                   20.800 usec
DE                     6.00 usec
TE                    293.0 K
D1               2.00000000 sec
D11              0.03000000 sec
TD0                       1

======== CHANNEL f1 ========
NUC1                    13C
P1                    40.00 usec
PL1                   -3.00 dB
PL1W            60.64365387 W
SFO1            100.6228298 MHz

======== CHANNEL f2 ========
CPDPRG[2            waltz16
NUC2                     1H
PCPD2                 80.00 usec
PL2                   -1.00 dB
PL12                  14.39 dB
PL13                  18.00 dB
PL2W            12.17476940 W
PL12W            0.35193357 W
PL13W            0.15327126 W
SFO2            400.1316005 MHz

F2 - Processing parameters
SI                    32768
SF              100.6127860 MHz
WDW                      EM
SSB                       0
LB                     1.00 Hz
GB                        0
PC                     1.40
```

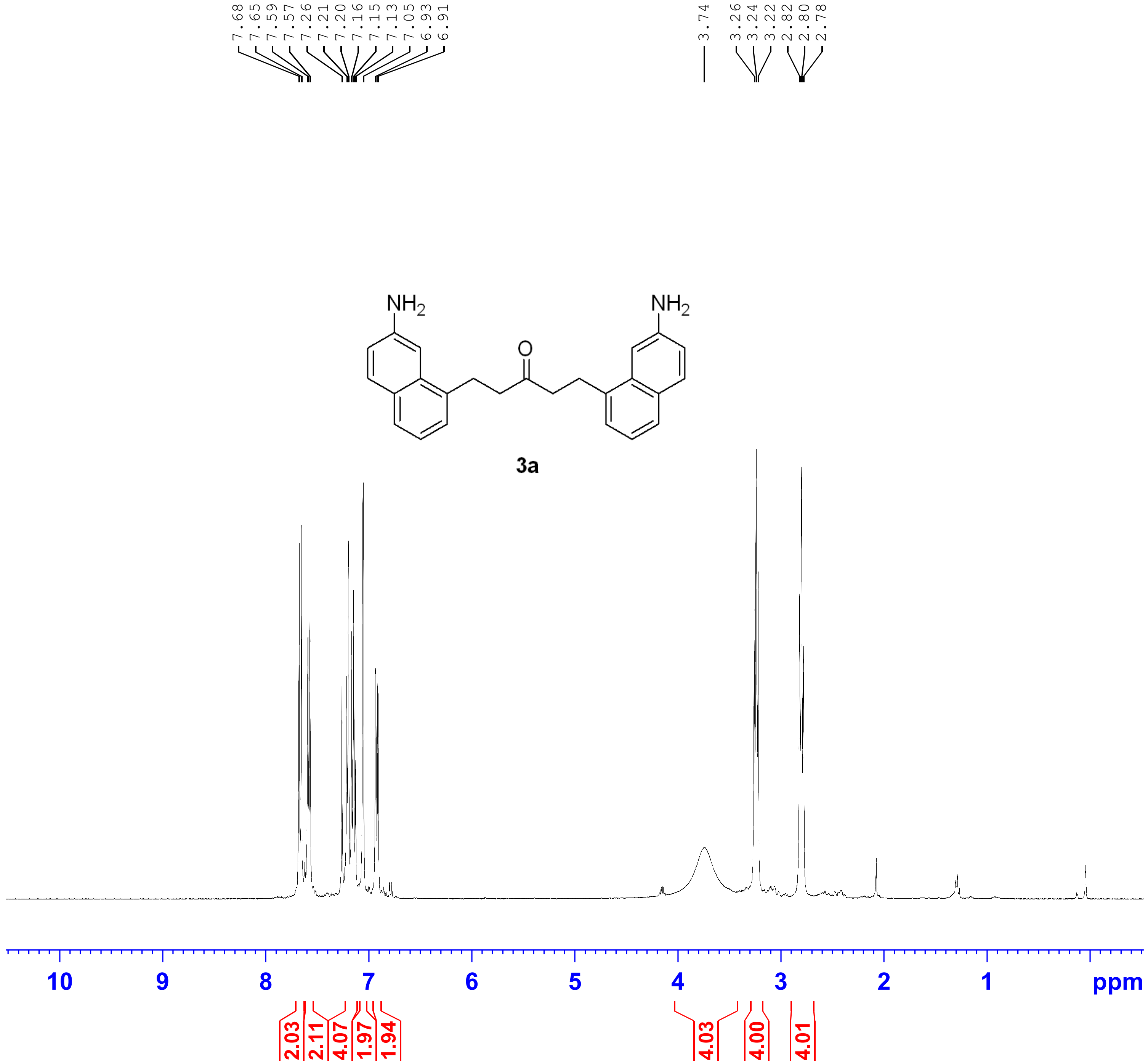


```
Current Data Parameters
NAME       zrh-2-182-2-amine-h
EXPNO                    1
PROCNO                   1

F2 - Acquisition Parameters
Date_             20210408
Time                 19.18
INSTRUM              spect
PROBHD    5 mm PABBO BB/
PULPROG               zg30
TD                   65536
SOLVENT              CDCl3
NS                       2
DS                       0
SWH               8012.820 Hz
FIDRES            0.122266 Hz
AQ               4.0894465 sec
RG                   39.46
DW                  62.400 usec
DE                    6.50 usec
TE                   296.7 K
D1              1.00000000 sec
TD0                      1

======== CHANNEL f1 ========
SFO1           400.1324710 MHz
NUC1                    1H
P1                   14.50 usec
PLW1           11.99499989 W

F2 - Processing parameters
SI                   65536
SF             400.1300100 MHz
WDW                     EM
SSB                      0
LB                    0.30 Hz
GB                       0
PC                    1.00
```

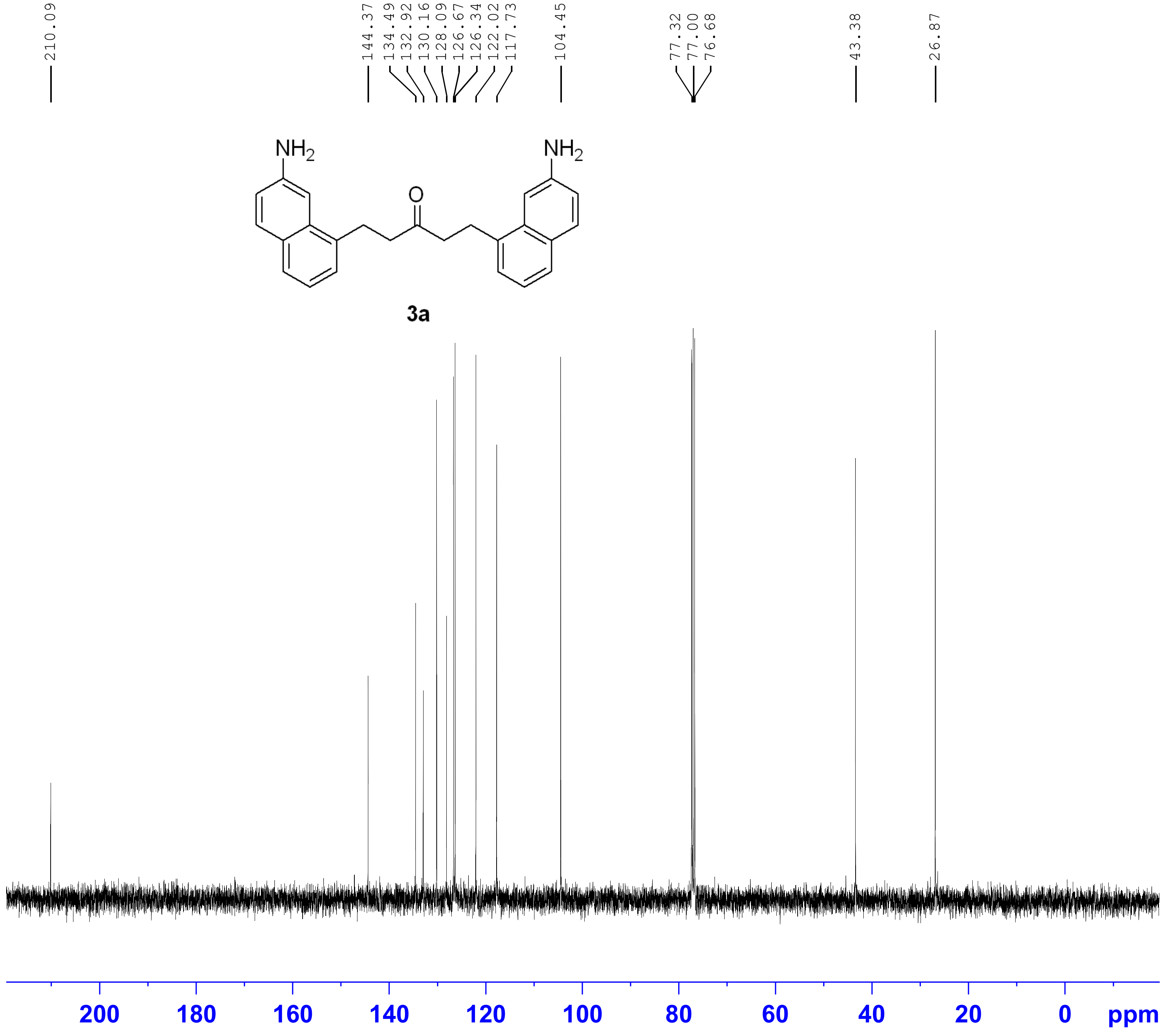


```
Current Data Parameters
NAME      zrh-2-182-2-amine-c
EXPNO                  1
PROCNO                 1

F2 - Acquisition Parameters
Date_            20210408
Time                19.19
INSTRUM             spect
PROBHD   5 mm PABBO BB/
PULPROG           zgpg30
TD                 65536
SOLVENT            CDCl3
NS                    40
DS                     0
SWH            24038.461 Hz
FIDRES          0.366798 Hz
AQ             1.3631488 sec
RG                196.92
DW                20.800 usec
DE                  6.50 usec
TE                 297.3 K
D1            2.00000000 sec
D11           0.03000000 sec
TD0                    1

======== CHANNEL f1 ========
SFO1         100.6228298 MHz
NUC1                 13C
P1                  9.70 usec
PLW1         46.98899841 W

======== CHANNEL f2 ========
SFO2         400.1316005 MHz
NUC2                  1H
CPDPRG[2         waltz16
PCPD2              90.00 usec
PLW2         11.99499989 W
PLW12         0.34213999 W
PLW13         0.27713001 W

F2 - Processing parameters
SI                 32768
SF           100.6127817 MHz
WDW                   EM
SSB                    0
LB                  1.00 Hz
GB                     0
PC                  1.40
```

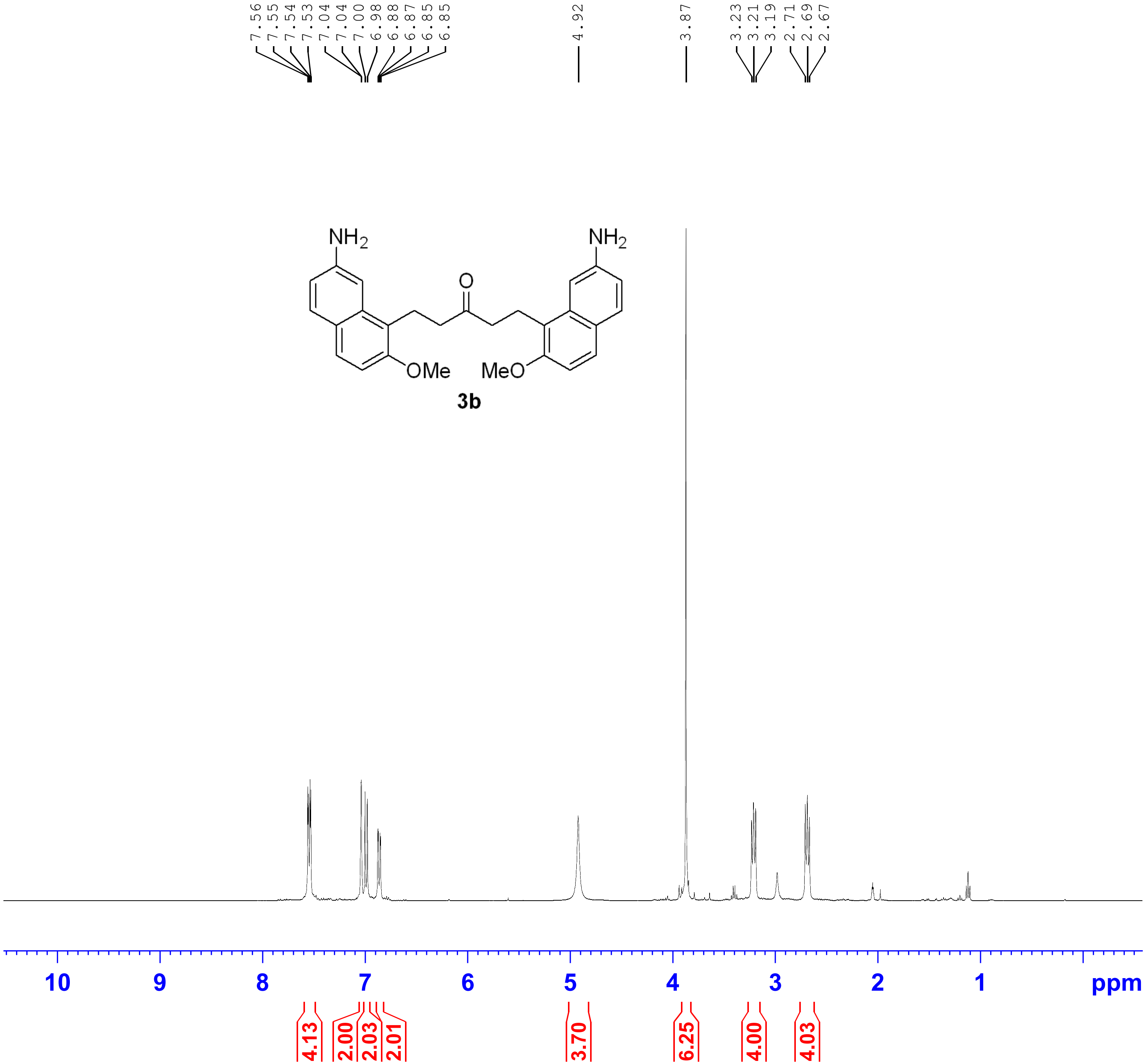


```
Current Data Parameters
NAME      zrh-6-130-nh2-acetone-h
EXPNO                   1
PROCNO                  1

F2 - Acquisition Parameters
Date_            20220608
Time                19.03
INSTRUM             spect
PROBHD    5 mm DUL 13C-1
PULPROG              zg30
TD                  65536
SOLVENT           Acetone
NS                      2
DS                      0
SWH              8223.685 Hz
FIDRES           0.125483 Hz
AQ              3.9845889 sec
RG                     64
DW                 60.800 usec
DE                   6.00 usec
TE                  292.6 K
D1             1.00000000 sec
TD0                     1

======== CHANNEL f1 ========
NUC1                   1H
P1                  15.80 usec
PL1                 -1.00 dB
PL1W          12.17476940 W
SFO1          400.1324710 MHz

F2 - Processing parameters
SI                  32768
SF            400.1300070 MHz
WDW                    EM
SSB                     0
LB                   0.30 Hz
GB                      0
PC                   1.00
```

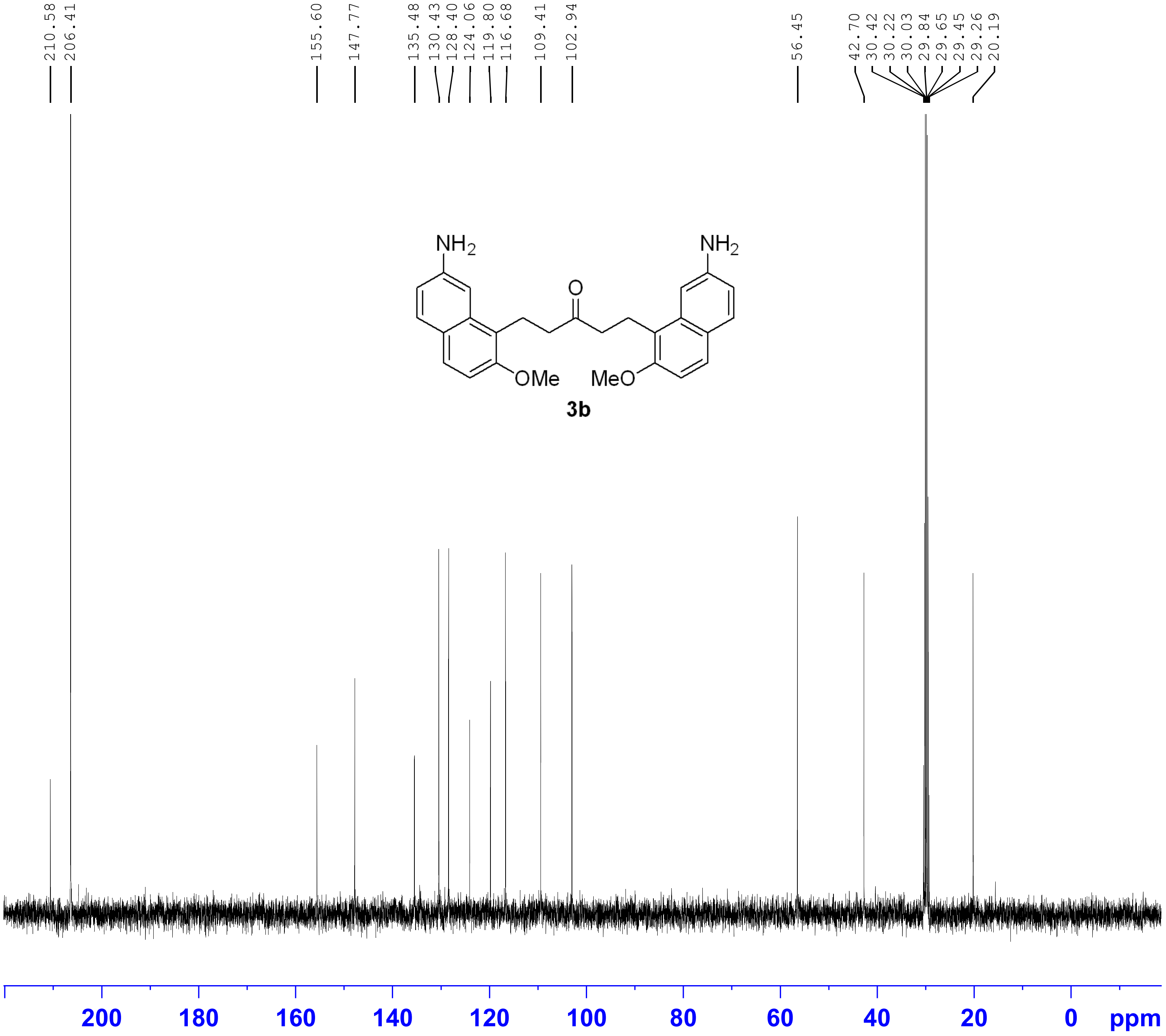

Current Data Parameters
NAME zrh-6-130-nh2-acetone-c
EXPNO 1
PROCNO 1

F2 - Acquisition Parameters
Date_ 20220608
Time 19.05
INSTRUM spect
PROBHD 5 mm DUL 13C-1
PULPROG zgpg30
TD 65536
SOLVENT Acetone
NS 51
DS 0
SWH 24038.461 Hz
FIDRES 0.366798 Hz
AQ 1.3631488 sec
RG 2050
DW 20.800 usec
DE 6.00 usec
TE 292.8 K
D1 2.00000000 sec
D11 0.03000000 sec
TD0 1

======== CHANNEL f1 ========
NUC1 13C
P1 40.00 usec
PL1 -3.00 dB
PL1W 60.64365387 W
SFO1 100.6228298 MHz

======== CHANNEL f2 ========
CPDPRG[2 waltz16
NUC2 1H
PCPD2 80.00 usec
PL2 -1.00 dB
PL12 14.39 dB
PL13 18.00 dB
PL2W 12.17476940 W
PL12W 0.35193357 W
PL13W 0.15327126 W
SFO2 400.1316005 MHz

F2 - Processing parameters
SI 32768
SF 100.6126893 MHz
WDW EM
SSB 0
LB 1.00 Hz
GB 0
PC 1.40

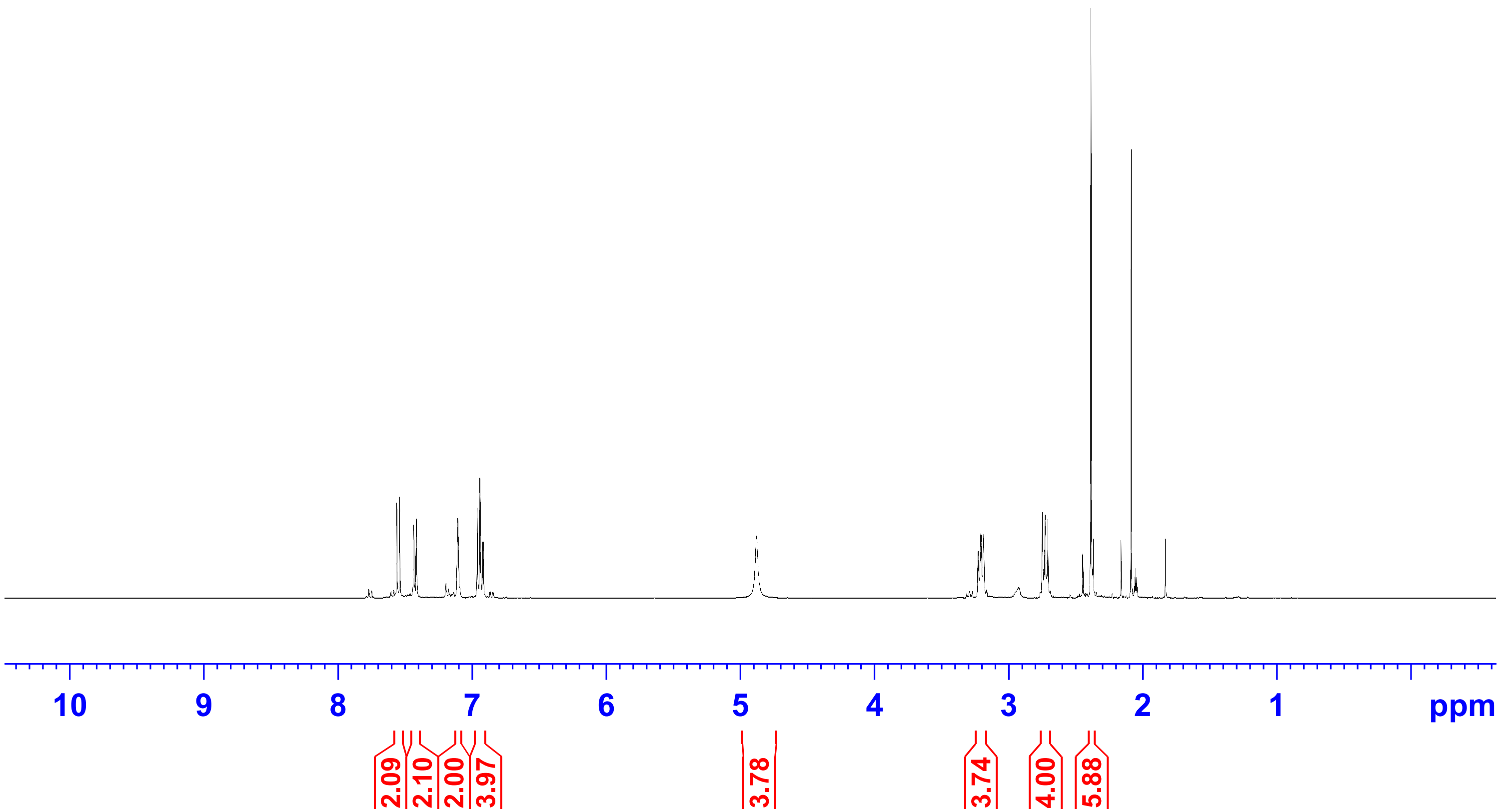

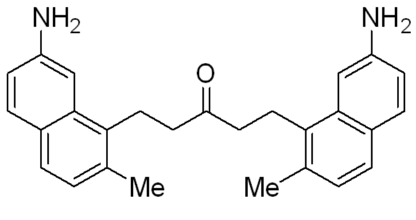

3c

```
Current Data Parameters
NAME      zrh-6-194-NH2-h
EXPNO                   1
PROCNO                  1

F2 - Acquisition Parameters
Date_            20220630
Time                21.54
INSTRUM             spect
PROBHD   5 mm DUL 13C-1
PULPROG              zg30
TD                  65536
SOLVENT           Acetone
NS                      2
DS                      0
SWH              8223.685 Hz
FIDRES           0.125483 Hz
AQ              3.9845889 sec
RG                    128
DW                 60.800 usec
DE                   6.00 usec
TE                  293.6 K
D1             1.00000000 sec
TD0                     1

======== CHANNEL f1 ========
NUC1                   1H
P1                  15.80 usec
PL1                 -1.00 dB
PL1W          12.17476940 W
SFO1          400.1324710 MHz

F2 - Processing parameters
SI                  32768
SF            400.1300068 MHz
WDW                    EM
SSB                     0
LB                   0.30 Hz
GB                      0
PC                   1.00
```

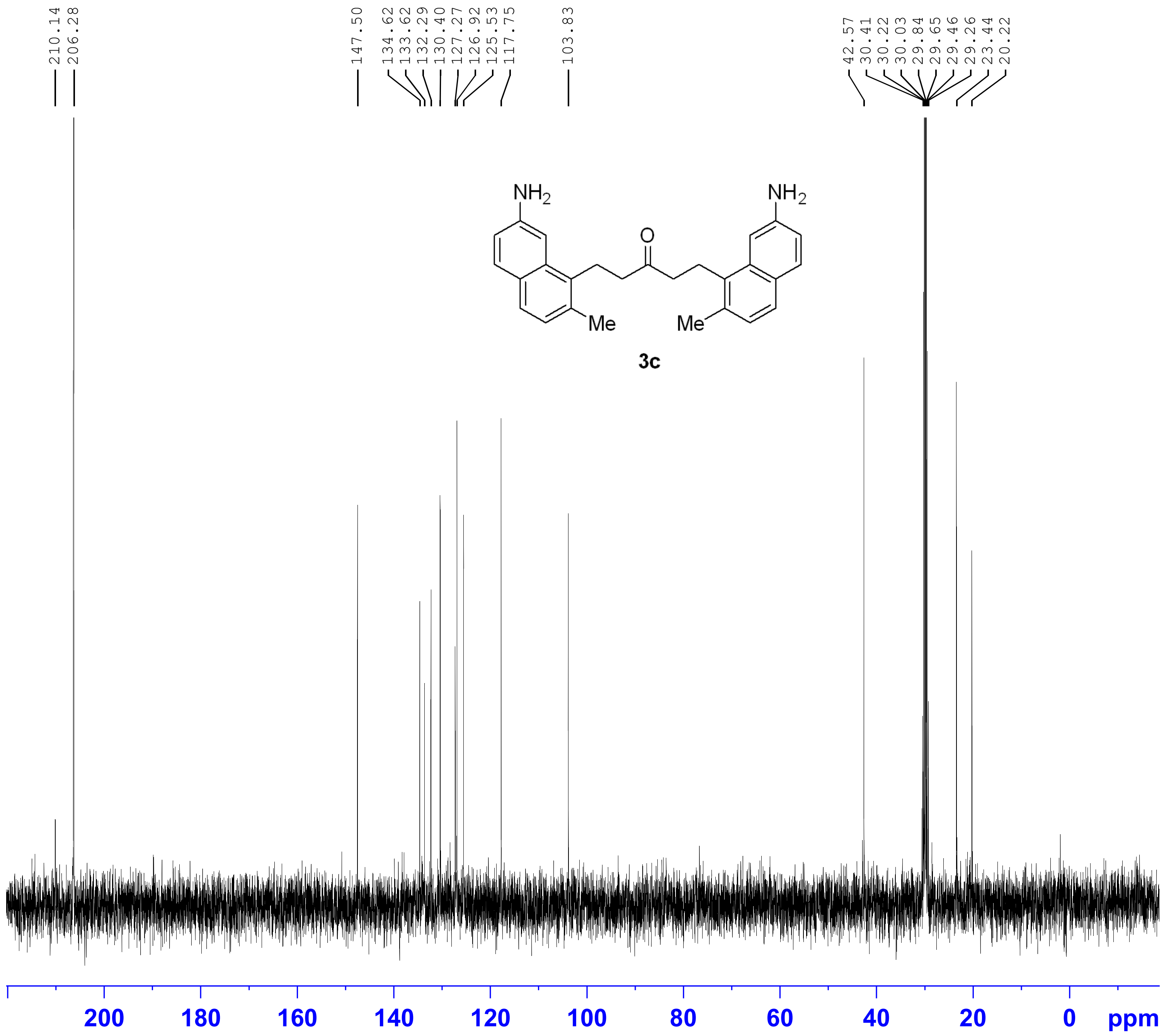

Current Data Parameters
NAME zrh-6-194-NH2-c
EXPNO 1
PROCNO 1

F2 - Acquisition Parameters
Date_ 20220630
Time 21.56
INSTRUM spect
PROBHD 5 mm DUL 13C-1
PULPROG zgpg30
TD 65536
SOLVENT Acetone
NS 37
DS 0
SWH 24038.461 Hz
FIDRES 0.366798 Hz
AQ 1.3631488 sec
RG 2050
DW 20.800 usec
DE 6.00 usec
TE 293.9 K
D1 2.00000000 sec
D11 0.03000000 sec
TD0 1

======== CHANNEL f1 ========
NUC1 13C
P1 40.00 usec
PL1 -3.00 dB
PL1W 60.64365387 W
SFO1 100.6228298 MHz

======== CHANNEL f2 ========
CPDPRG[2 waltz16
NUC2 1H
PCPD2 80.00 usec
PL2 -1.00 dB
PL12 14.39 dB
PL13 18.00 dB
PL2W 12.17476940 W
PL12W 0.35193357 W
PL13W 0.15327126 W
SFO2 400.1316005 MHz

F2 - Processing parameters
SI 32768
SF 100.6126860 MHz
WDW EM
SSB 0
LB 1.00 Hz
GB 0
PC 1.40

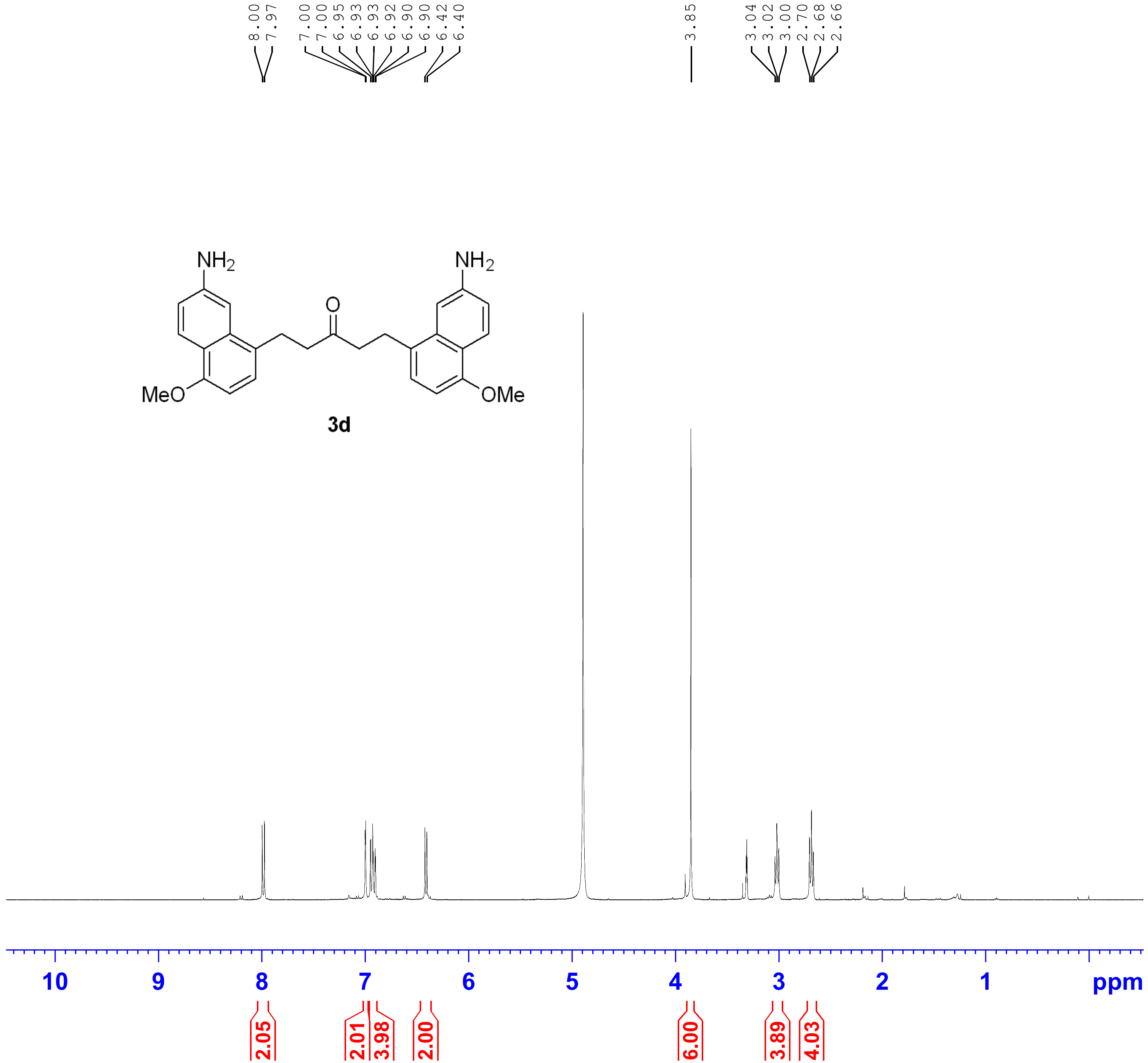


```
Current Data Parameters
NAME         zrh-7-53-re-h
EXPNO                    1
PROCNO                   1

F2 - Acquisition Parameters
Date_             20220726
Time                 19.46
INSTRUM              spect
PROBHD     5 mm DUL 13C-1
PULPROG               zg30
TD                   65536
SOLVENT               MeOD
NS                       2
DS                       0
SWH               8223.685 Hz
FIDRES            0.125483 Hz
AQ               3.9845889 sec
RG                     256
DW                  60.800 usec
DE                    6.00 usec
TE                   292.9 K
D1              1.00000000 sec
TD0                      1

======== CHANNEL f1 ========
NUC1                    1H
P1                   15.80 usec
PL1                  -1.00 dB
PL1W           12.17476940 W
SFO1           400.1324710 MHz

F2 - Processing parameters
SI                   32768
SF             400.1300077 MHz
WDW                     EM
SSB                      0
LB                    0.30 Hz
GB                       0
PC                    1.00
```

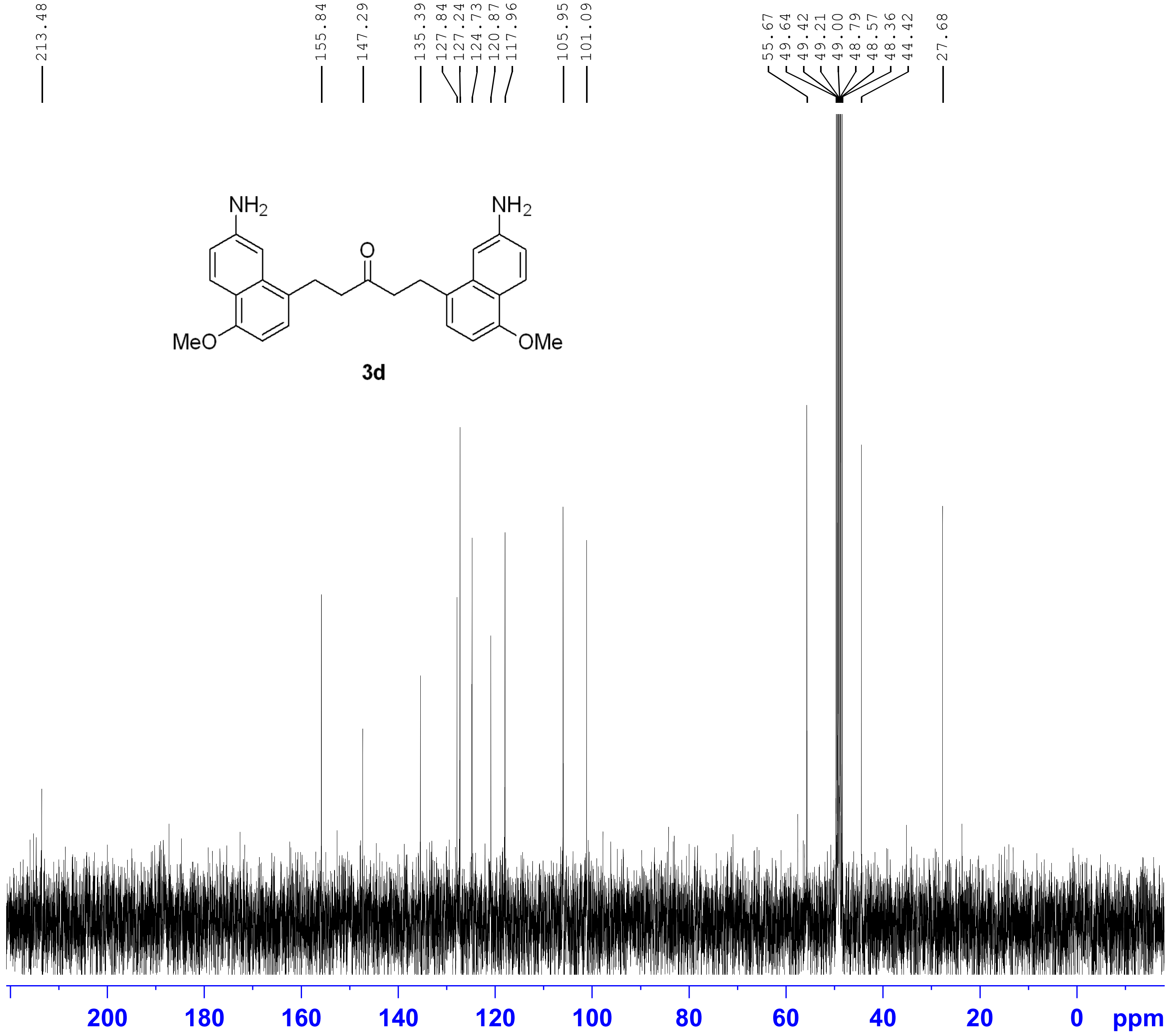


```
Current Data Parameters
NAME      zrh-7-53-re-cc
EXPNO                  1
PROCNO                 1

F2 - Acquisition Parameters
Date_           20220726
Time               19.49
INSTRUM            spect
PROBHD    5 mm DUL 13C-1
PULPROG          zgpg30
TD                 65536
SOLVENT             MeOD
NS                    60
DS                     0
SWH            24038.461 Hz
FIDRES          0.366798 Hz
AQ             1.3631488 sec
RG                  2050
DW                20.800 usec
DE                  6.00 usec
TE                 293.2 K
D1            2.00000000 sec
D11           0.03000000 sec
TD0                    1

======== CHANNEL f1 ========
NUC1                 13C
P1                 40.00 usec
PL1                -3.00 dB
PL1W         60.64365387 W
SFO1        100.6228298 MHz

======== CHANNEL f2 ========
CPDPRG[2          waltz16
NUC2                  1H
PCPD2              80.00 usec
PL2                -1.00 dB
PL12               14.39 dB
PL13               18.00 dB
PL2W         12.17476940 W
PL12W         0.35193357 W
PL13W         0.15327126 W
SFO2        400.1316005 MHz

F2 - Processing parameters
SI                 32768
SF           100.6126300 MHz
WDW                   EM
SSB                    0
LB                  1.00 Hz
GB                     0
PC                  1.40
```

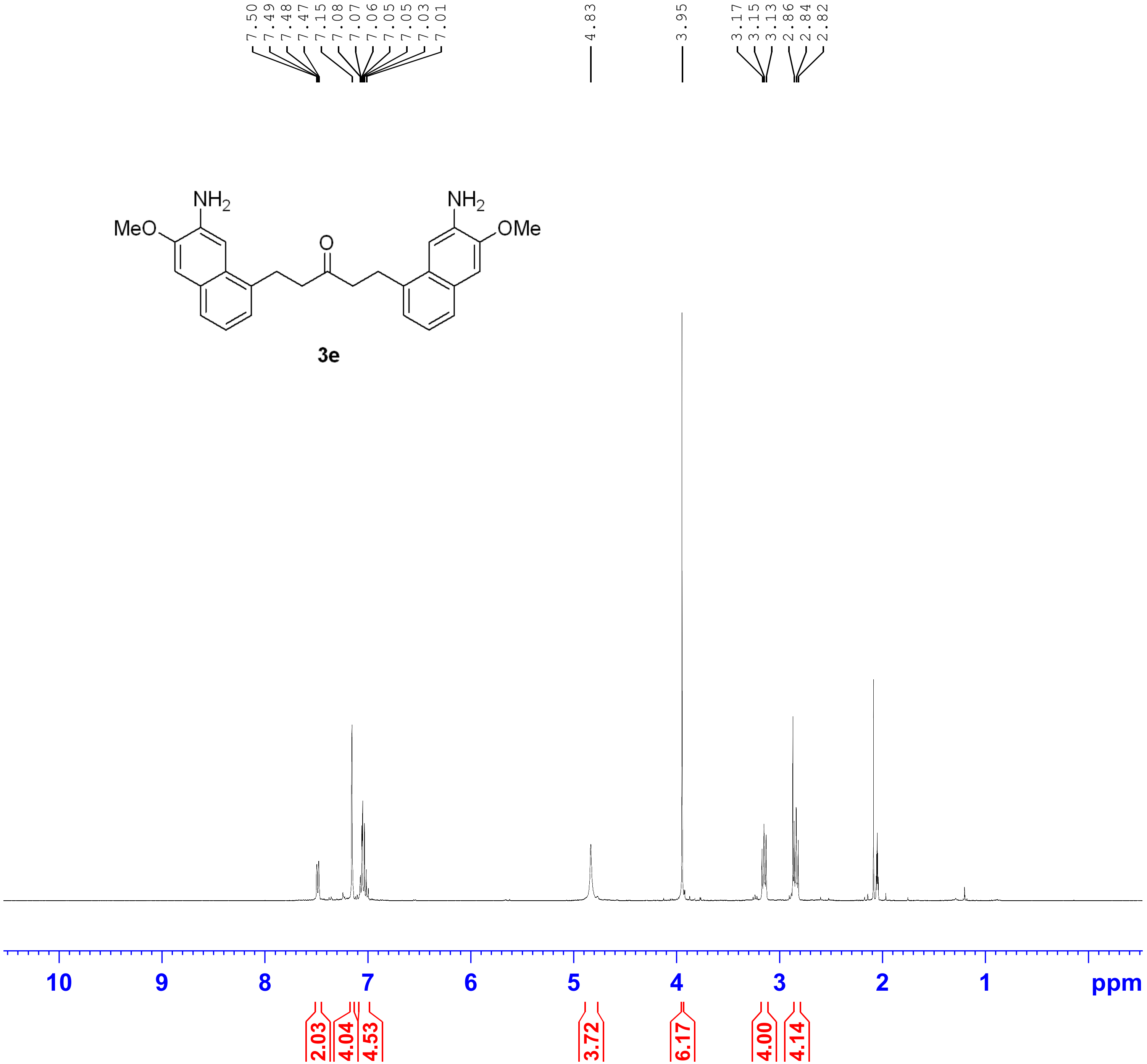


```
Current Data Parameters
NAME      zrh-6-154-re-h
EXPNO                  1
PROCNO                 1

F2 - Acquisition Parameters
Date_           20220615
Time               20.25
INSTRUM            spect
PROBHD   5 mm DUL 13C-1
PULPROG             zg30
TD                 65536
SOLVENT          Acetone
NS                     2
DS                     0
SWH             8223.685 Hz
FIDRES          0.125483 Hz
AQ             3.9845889 sec
RG                   287
DW                60.800 usec
DE                  6.00 usec
TE                 292.6 K
D1            1.00000000 sec
TD0                    1

======== CHANNEL f1 ========
NUC1                  1H
P1                 15.80 usec
PL1                -1.00 dB
PL1W         12.17476940 W
SFO1         400.1324710 MHz

F2 - Processing parameters
SI                 32768
SF           400.1300070 MHz
WDW                   EM
SSB                    0
LB                  0.30 Hz
GB                     0
PC                  1.00
```

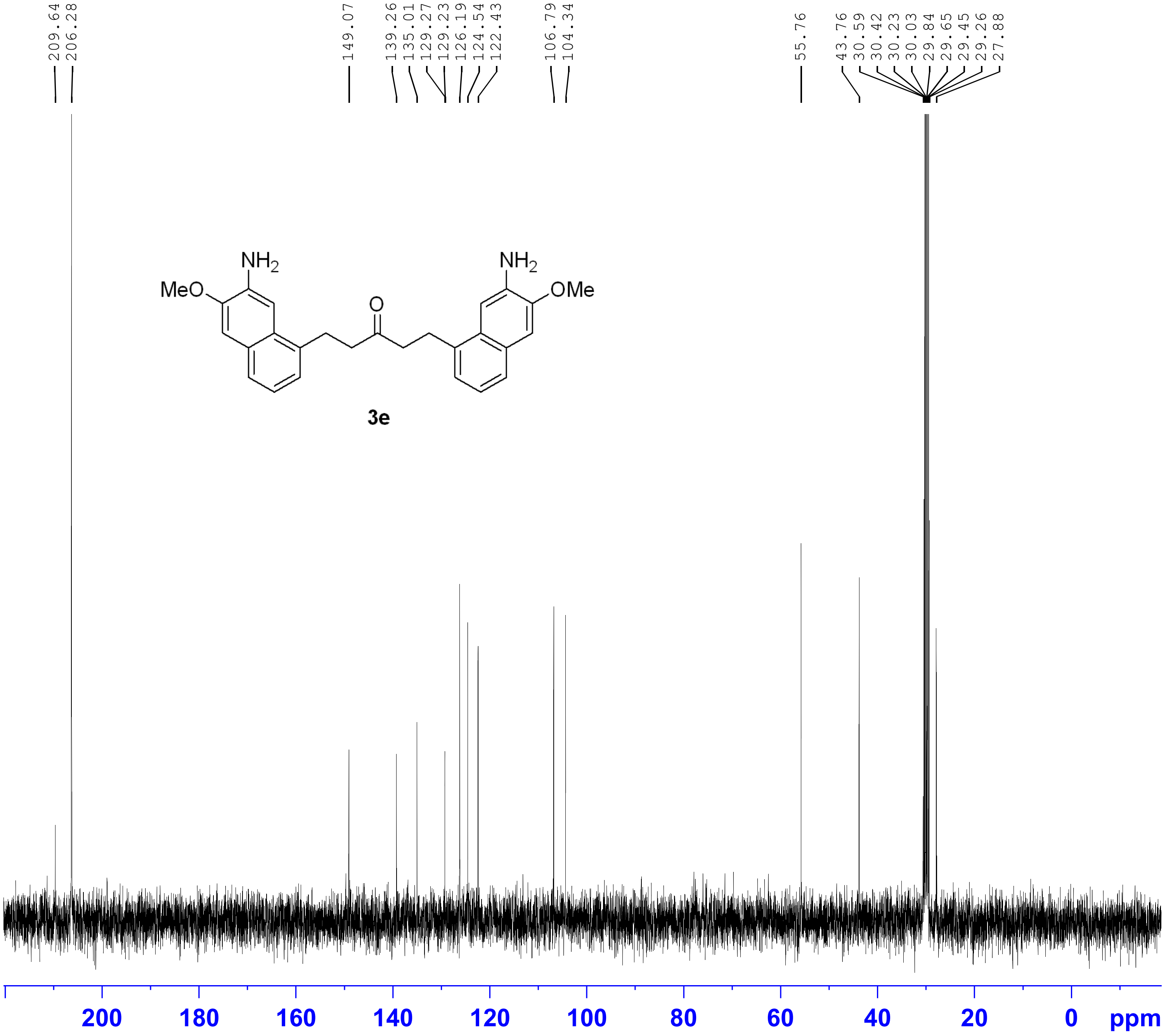

Current Data Parameters
NAME zrh-6-154-pdt-c
EXPNO 1
PROCNO 1

F2 - Acquisition Parameters
Date_ 20220615
Time 13.34
INSTRUM spect
PROBHD 5 mm DUL 13C-1
PULPROG zgpg30
TD 65536
SOLVENT Acetone
NS 89
DS 0
SWH 24038.461 Hz
FIDRES 0.366798 Hz
AQ 1.3631488 sec
RG 2050
DW 20.800 usec
DE 6.00 usec
TE 293.2 K
D1 2.00000000 sec
D11 0.03000000 sec
TD0 1

======== CHANNEL f1 ========
NUC1 13C
P1 40.00 usec
PL1 -3.00 dB
PL1W 60.64365387 W
SFO1 100.6228298 MHz

======== CHANNEL f2 ========
CPDPRG[2 waltz16
NUC2 1H
PCPD2 80.00 usec
PL2 -1.00 dB
PL12 14.39 dB
PL13 18.00 dB
PL2W 12.17476940 W
PL12W 0.35193357 W
PL13W 0.15327126 W
SFO2 400.1316005 MHz

F2 - Processing parameters
SI 32768
SF 100.6126827 MHz
WDW EM
SSB 0
LB 1.00 Hz
GB 0
PC 1.40

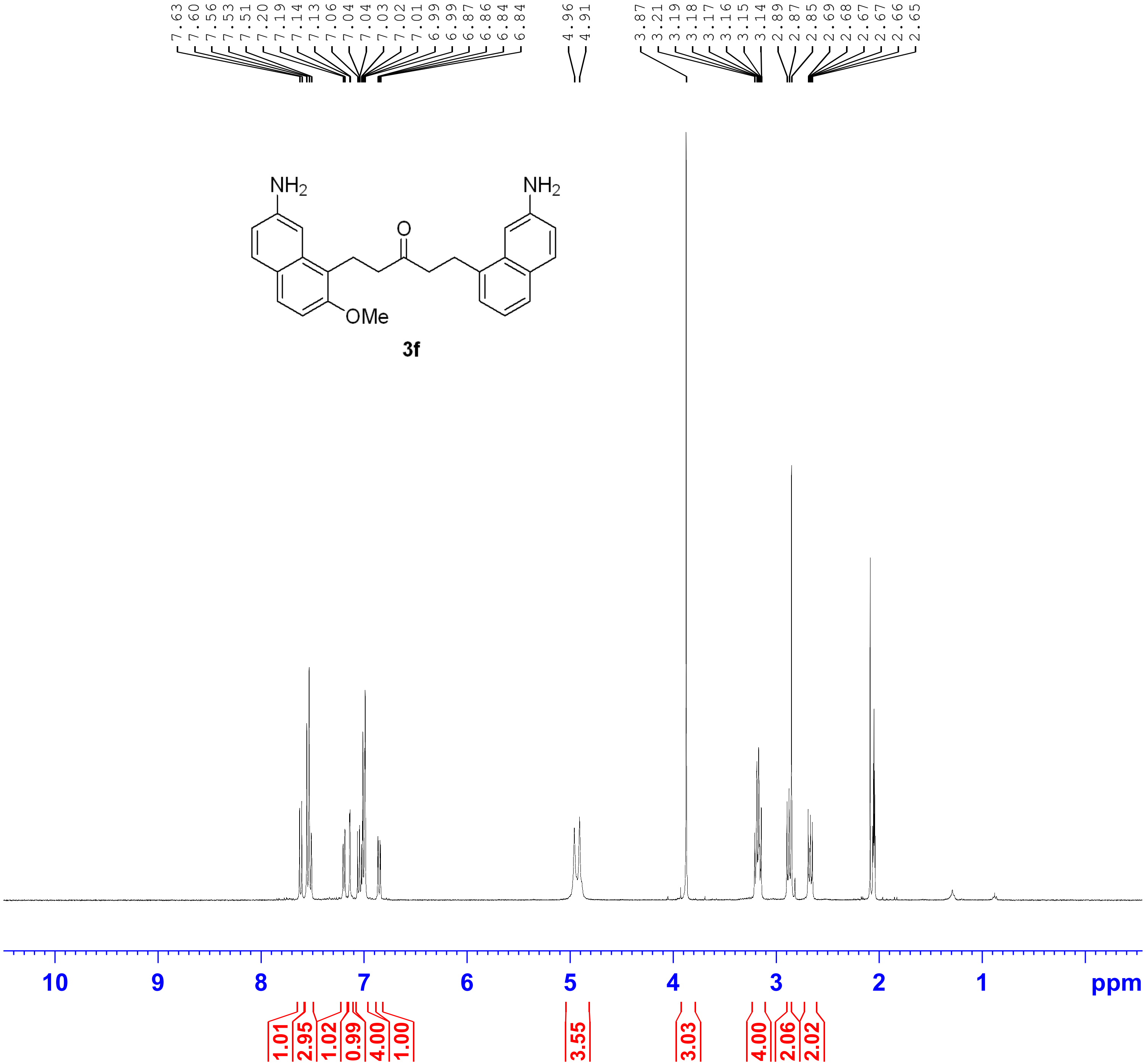


```
Current Data Parameters
NAME      zrh-6-176-re-h
EXPNO                  1
PROCNO                 1

F2 - Acquisition Parameters
Date_           20220623
Time               22.16
INSTRUM            spect
PROBHD    5 mm PABBO BB/
PULPROG             zg30
TD                 65536
SOLVENT          Acetone
NS                     2
DS                     0
SWH             8012.820 Hz
FIDRES          0.122266 Hz
AQ             4.0894465 sec
RG                142.88
DW                62.400 usec
DE                  6.50 usec
TE                 295.9 K
D1            1.00000000 sec
TD0                    1

======== CHANNEL f1 ========
SFO1         400.1324710 MHz
NUC1                  1H
P1                 14.50 usec
PLW1         11.99499989 W

F2 - Processing parameters
SI                 65536
SF           400.1300070 MHz
WDW                   EM
SSB                    0
LB                  0.30 Hz
GB                     0
PC                  1.00
```

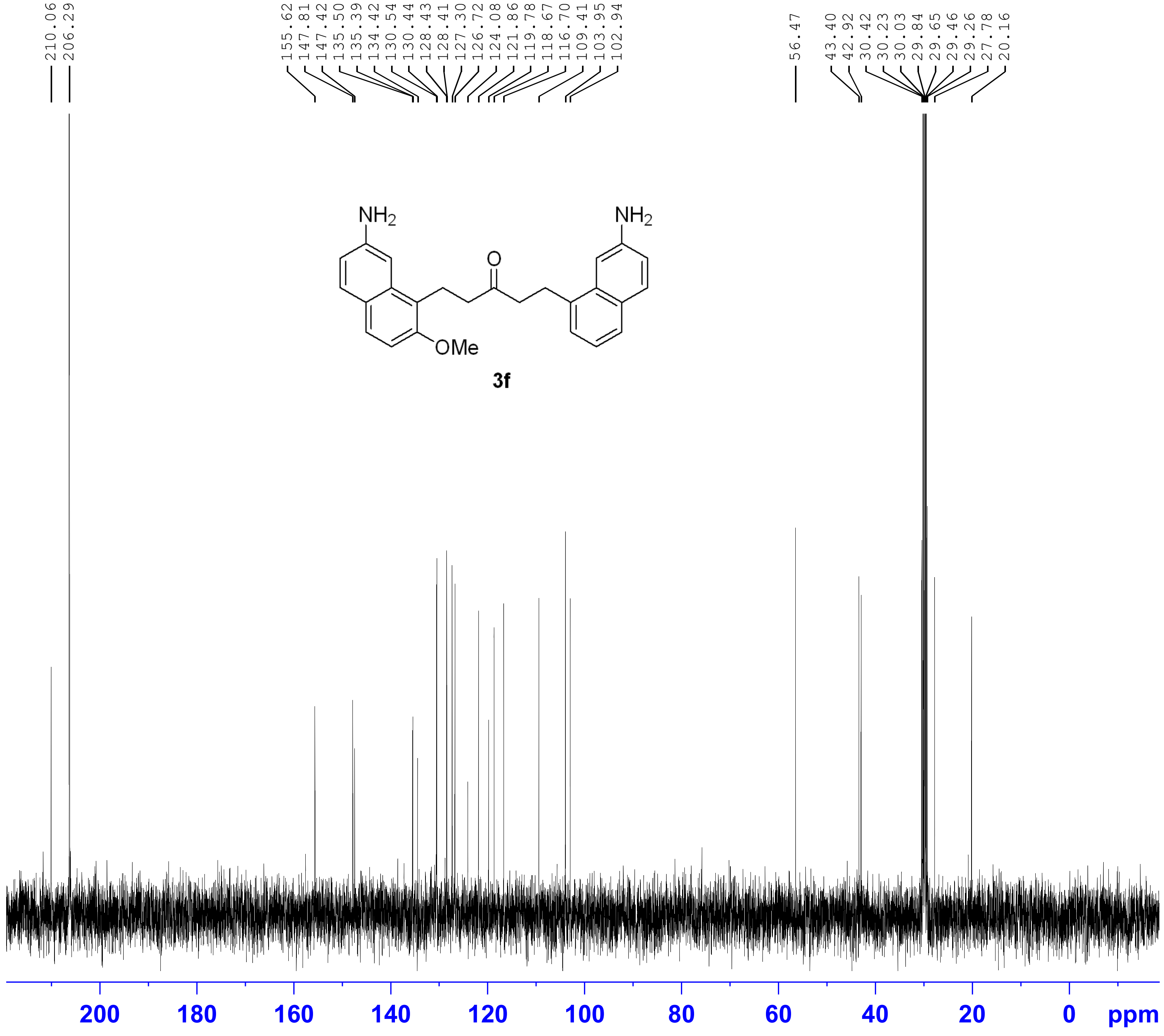

Current Data Parameters
NAME zrh-6-176-NH2-c
EXPNO 1
PROCNO 1

F2 - Acquisition Parameters
Date_ 20220623
Time 19.38
INSTRUM spect
PROBHD 5 mm DUL 13C-1
PULPROG zgpg30
TD 65536
SOLVENT Acetone
NS 40
DS 0
SWH 24038.461 Hz
FIDRES 0.366798 Hz
AQ 1.3631488 sec
RG 2050
DW 20.800 usec
DE 6.00 usec
TE 292.9 K
D1 2.00000000 sec
D11 0.03000000 sec
TD0 1

======== CHANNEL f1 ========
NUC1 13C
P1 40.00 usec
PL1 -3.00 dB
PL1W 60.64365387 W
SFO1 100.6228298 MHz

======== CHANNEL f2 ========
CPDPRG[2 waltz16
NUC2 1H
PCPD2 80.00 usec
PL2 -1.00 dB
PL12 14.39 dB
PL13 18.00 dB
PL2W 12.17476940 W
PL12W 0.35193357 W
PL13W 0.15327126 W
SFO2 400.1316005 MHz

F2 - Processing parameters
SI 32768
SF 100.6126849 MHz
WDW EM
SSB 0
LB 1.00 Hz
GB 0
PC 1.40

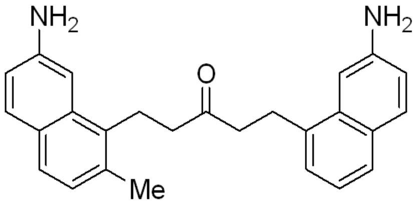

3g

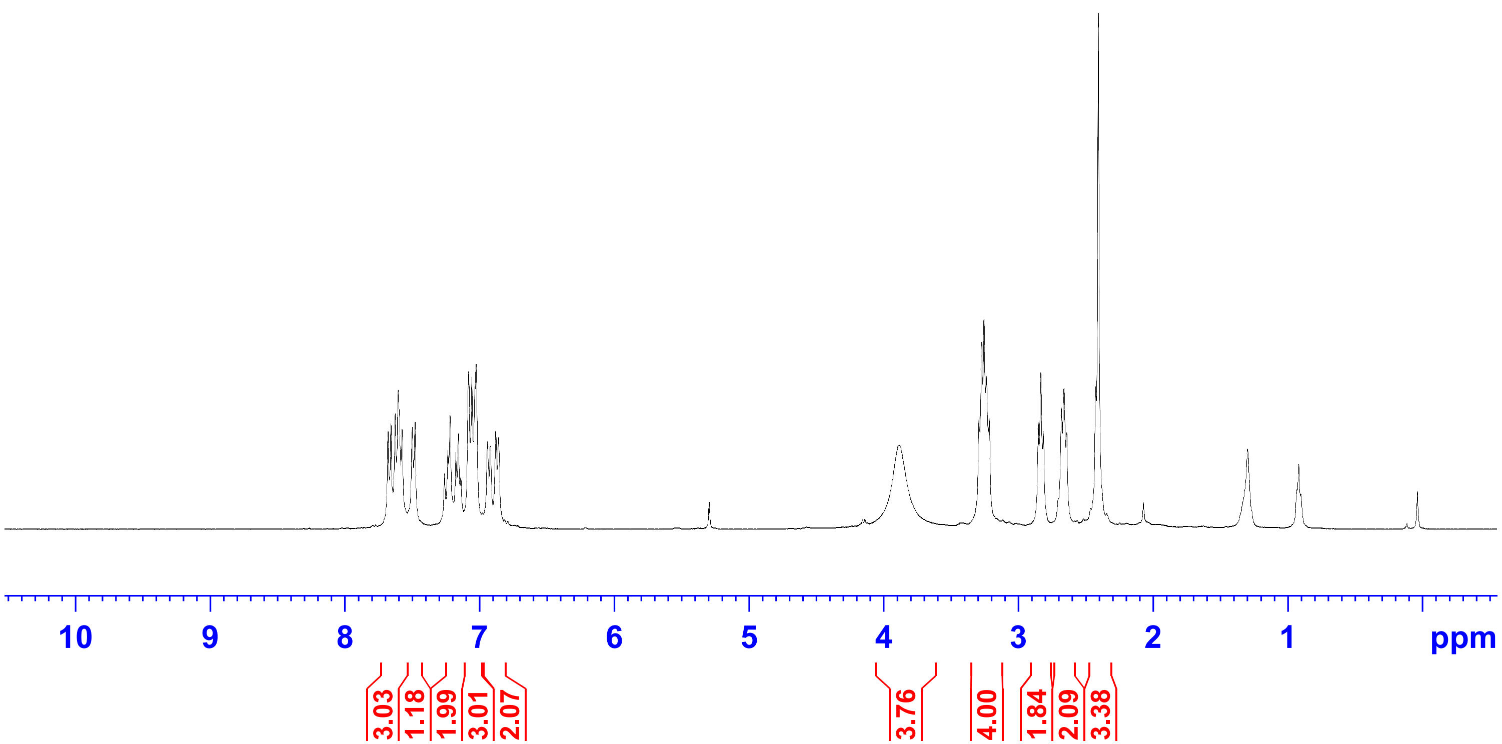


```
Current Data Parameters
NAME      zrh-8-95-NH2-h
EXPNO                  1
PROCNO                 1

F2 - Acquisition Parameters
Date_           20221224
Time               19.15
INSTRUM            spect
PROBHD   5 mm DUL 13C-1
PULPROG             zg30
TD                 65536
SOLVENT            CDCl3
NS                     7
DS                     0
SWH             8223.685 Hz
FIDRES          0.125483 Hz
AQ             3.9845889 sec
RG                   181
DW                60.800 usec
DE                  6.00 usec
TE                 294.8 K
D1            1.00000000 sec
TD0                    1

======== CHANNEL f1 ========
NUC1                  1H
P1                 15.80 usec
PL1                -1.00 dB
PL1W         12.17476940 W
SFO1         400.1324710 MHz

F2 - Processing parameters
SI                 32768
SF           400.1300095 MHz
WDW                   EM
SSB                    0
LB                  0.30 Hz
GB                     0
PC                  1.00
```

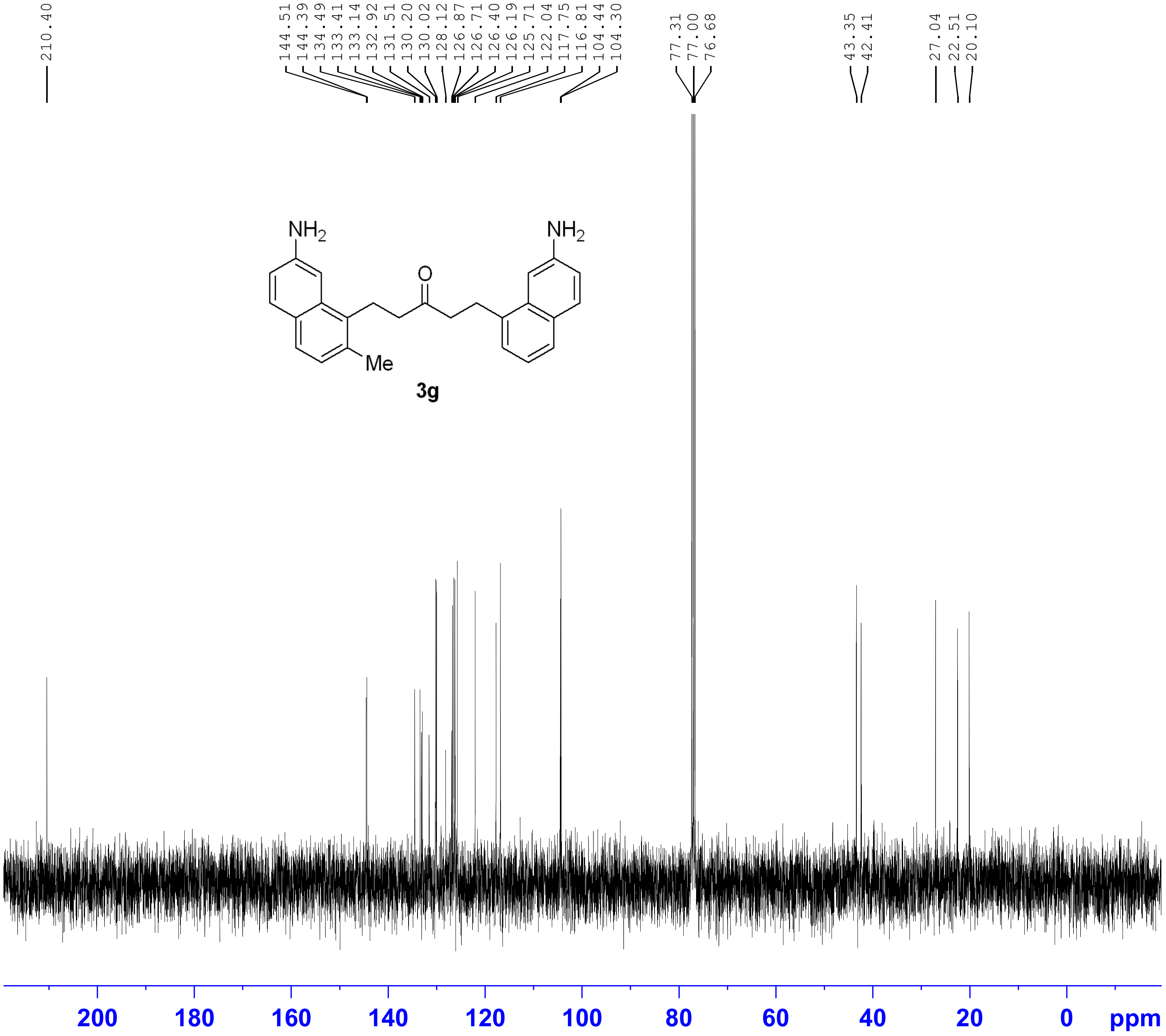

Current Data Parameters
NAME zrh-8-95-NH2-c
EXPNO 1
PROCNO 1

F2 - Acquisition Parameters
Date_ 20221224
Time 19.18
INSTRUM spect
PROBHD 5 mm DUL 13C-1
PULPROG zgpg30
TD 65536
SOLVENT CDCl3
NS 66
DS 0
SWH 24038.461 Hz
FIDRES 0.366798 Hz
AQ 1.3631488 sec
RG 2050
DW 20.800 usec
DE 6.00 usec
TE 295.1 K
D1 2.00000000 sec
D11 0.03000000 sec
TD0 1

======== CHANNEL f1 ========
NUC1 13C
P1 40.00 usec
PL1 -3.00 dB
PL1W 60.64365387 W
SFO1 100.6228298 MHz

======== CHANNEL f2 ========
CPDPRG[2 waltz16
NUC2 1H
PCPD2 80.00 usec
PL2 -1.00 dB
PL12 14.39 dB
PL13 18.00 dB
PL2W 12.17476940 W
PL12W 0.35193357 W
PL13W 0.15327126 W
SFO2 400.1316005 MHz

F2 - Processing parameters
SI 32768
SF 100.6127802 MHz
WDW EM
SSB 0
LB 1.00 Hz
GB 0
PC 1.40

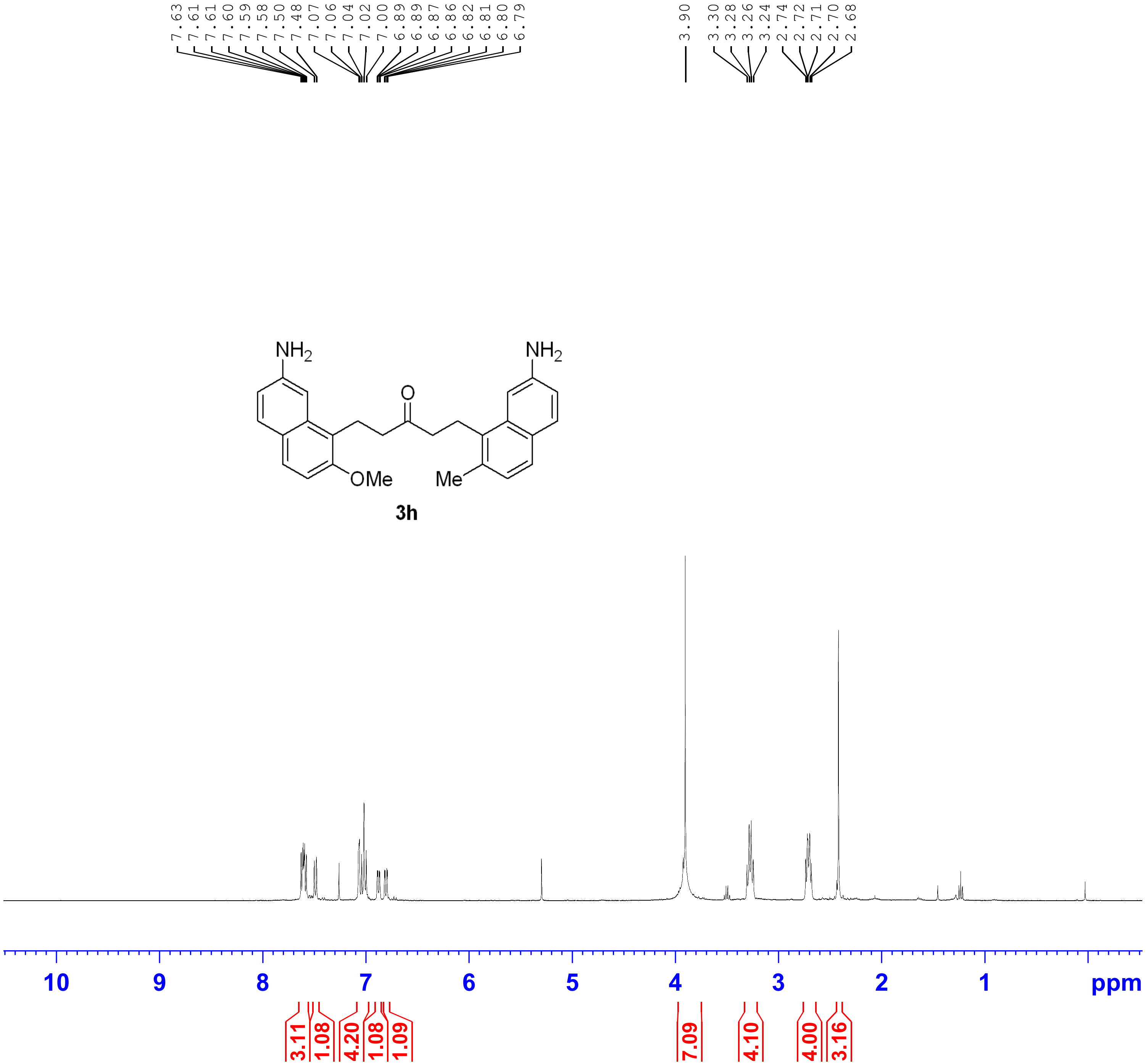

3h

Current Data Parameters
NAME zrh-7-1-re-h
EXPNO 1
PROCNO 1

F2 - Acquisition Parameters
Date_ 20230131
Time 21.05
INSTRUM spect
PROBHD 5 mm DUL 13C-1
PULPROG zg30
TD 65536
SOLVENT CDCl3
NS 2
DS 0
SWH 8223.685 Hz
FIDRES 0.125483 Hz
AQ 3.9845889 sec
RG 203
DW 60.800 usec
DE 6.00 usec
TE 292.6 K
D1 1.00000000 sec
TD0 1

======== CHANNEL f1 ========
NUC1 1H
P1 15.80 usec
PL1 -1.00 dB
PL1W 12.17476940 W
SFO1 400.1324710 MHz

F2 - Processing parameters
SI 32768
SF 400.1300096 MHz
WDW EM
SSB 0
LB 0.30 Hz
GB 0
PC 1.00

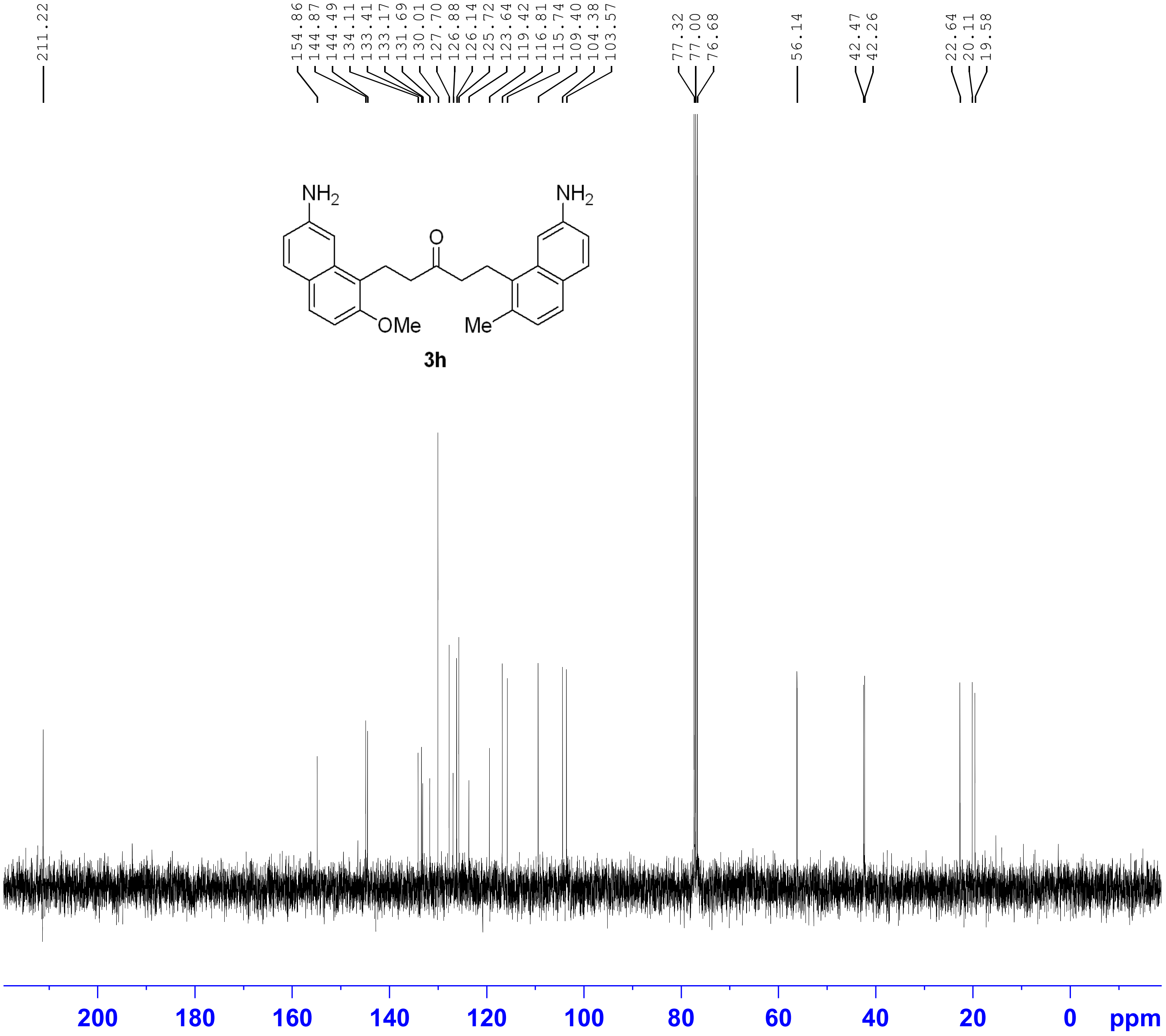


```
Current Data Parameters
NAME          zrh-7-1-re-c
EXPNO                    1
PROCNO                   1

F2 - Acquisition Parameters
Date_             20230131
Time                 21.06
INSTRUM              spect
PROBHD   5 mm DUL 13C-1
PULPROG            zgpg30
TD                   65536
SOLVENT              CDCl3
NS                      68
DS                       0
SWH              24038.461 Hz
FIDRES            0.366798 Hz
AQ               1.3631488 sec
RG                    2050
DW                  20.800 usec
DE                    6.00 usec
TE                   292.9 K
D1              2.00000000 sec
D11             0.03000000 sec
TD0                      1

======== CHANNEL f1 ========
NUC1                   13C
P1                   40.00 usec
PL1                  -3.00 dB
PL1W           60.64365387 W
SFO1           100.6228298 MHz

======== CHANNEL f2 ========
CPDPRG[2           waltz16
NUC2                    1H
PCPD2                80.00 usec
PL2                  -1.00 dB
PL12                 14.39 dB
PL13                 18.00 dB
PL2W           12.17476940 W
PL12W           0.35193357 W
PL13W           0.15327126 W
SFO2           400.1316005 MHz

F2 - Processing parameters
SI                   32768
SF             100.6127788 MHz
WDW                     EM
SSB                      0
LB                    1.00 Hz
GB                       0
PC                    1.40
```

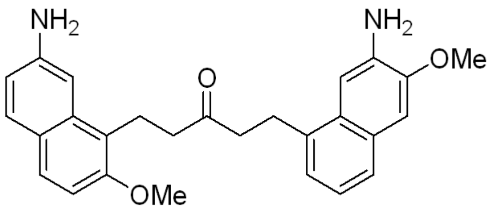

3i

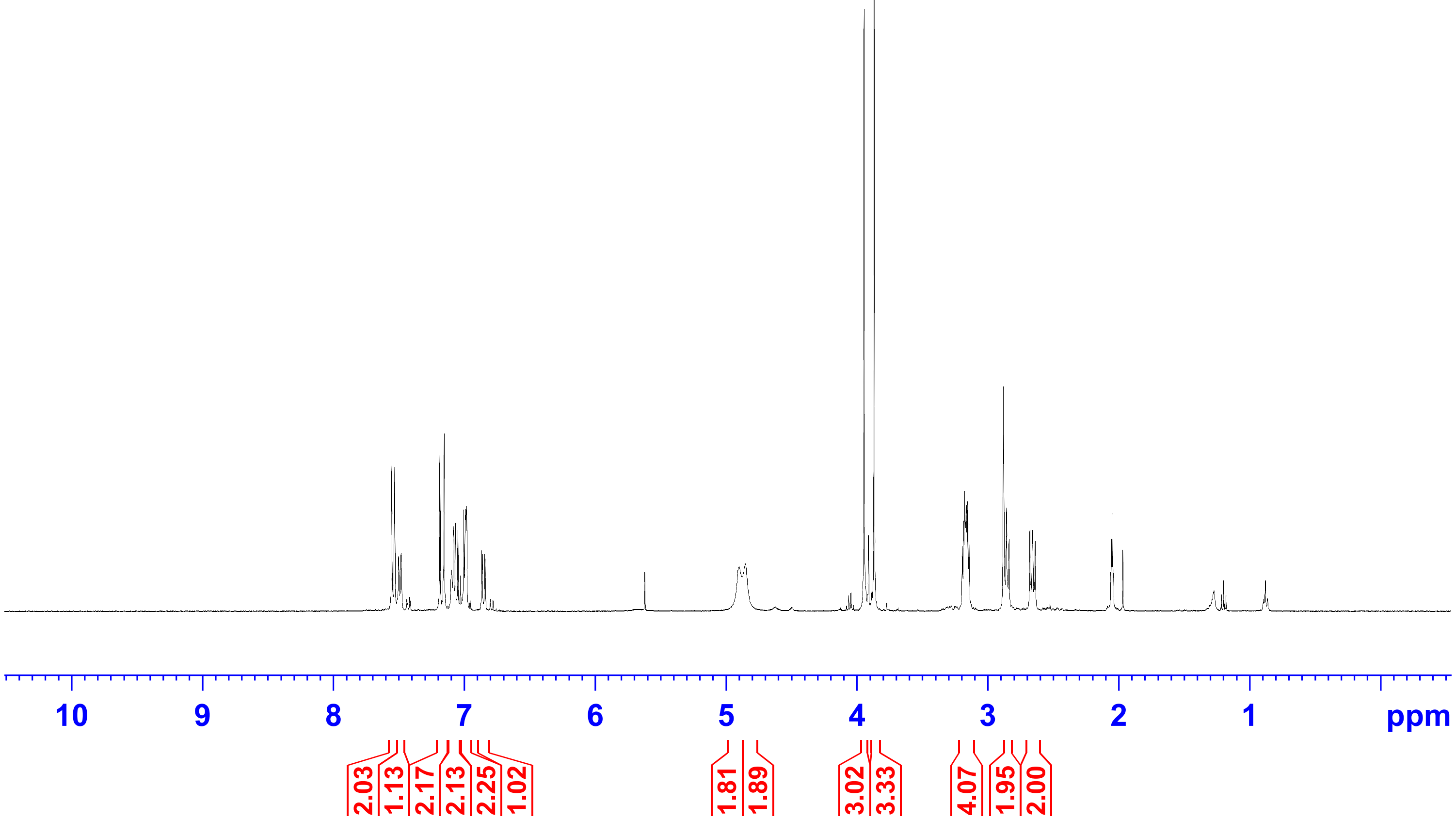


```
Current Data Parameters
NAME       zrh-6-172-nh2-h
EXPNO                    1
PROCNO                   1

F2 - Acquisition Parameters
Date_             20220622
Time                 19.17
INSTRUM              spect
PROBHD    5 mm DUL 13C-1
PULPROG               zg30
TD                   65536
SOLVENT            Acetone
NS                       2
DS                       0
SWH               8223.685 Hz
FIDRES            0.125483 Hz
AQ               3.9845889 sec
RG                     256
DW                  60.800 usec
DE                    6.00 usec
TE                   292.8 K
D1              1.00000000 sec
TD0                      1

======== CHANNEL f1 ========
NUC1                    1H
P1                   15.80 usec
PL1                  -1.00 dB
PL1W           12.17476940 W
SFO1           400.1324710 MHz

F2 - Processing parameters
SI                   32768
SF             400.1300069 MHz
WDW                     EM
SSB                      0
LB                    0.30 Hz
GB                       0
PC                    1.00
```

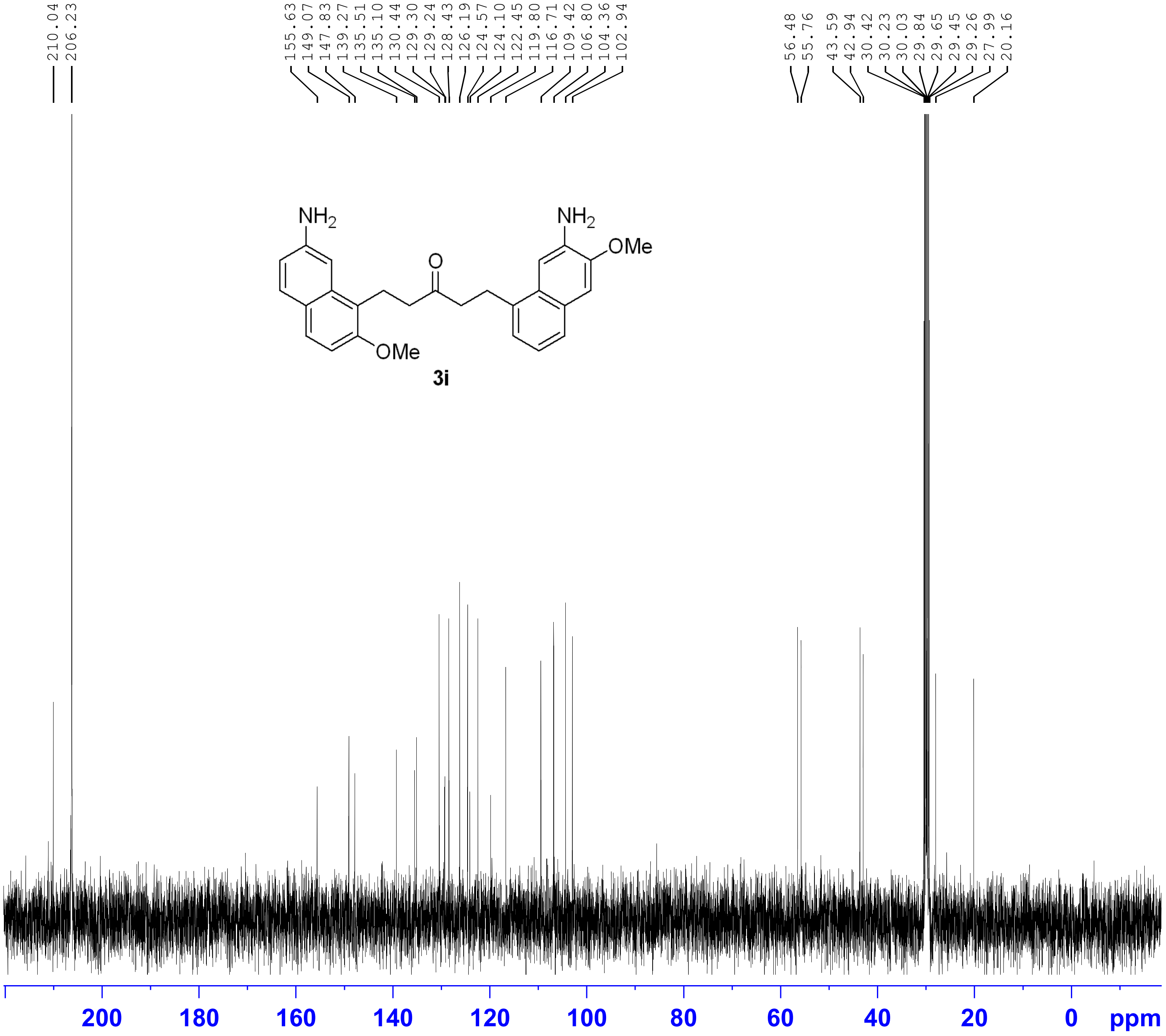


```
Current Data Parameters
NAME      zrh-6-172-nh2-c
EXPNO                  1
PROCNO                 1

F2 - Acquisition Parameters
Date_           20220622
Time               19.20
INSTRUM            spect
PROBHD    5 mm DUL 13C-1
PULPROG           zgpg30
TD                 65536
SOLVENT          Acetone
NS                    79
DS                     0
SWH            24038.461 Hz
FIDRES          0.366798 Hz
AQ             1.3631488 sec
RG                  2050
DW                20.800 usec
DE                  6.00 usec
TE                 293.0 K
D1            2.00000000 sec
D11           0.03000000 sec
TD0                    1

======== CHANNEL f1 ========
NUC1                 13C
P1                 40.00 usec
PL1                -3.00 dB
PL1W         60.64365387 W
SFO1        100.6228298 MHz

======== CHANNEL f2 ========
CPDPRG[2         waltz16
NUC2                  1H
PCPD2              80.00 usec
PL2                -1.00 dB
PL12               14.39 dB
PL13               18.00 dB
PL2W         12.17476940 W
PL12W         0.35193357 W
PL13W         0.15327126 W
SFO2        400.1316005 MHz

F2 - Processing parameters
SI                 32768
SF           100.6126827 MHz
WDW                   EM
SSB                    0
LB                  1.00 Hz
GB                     0
PC                  1.40
```

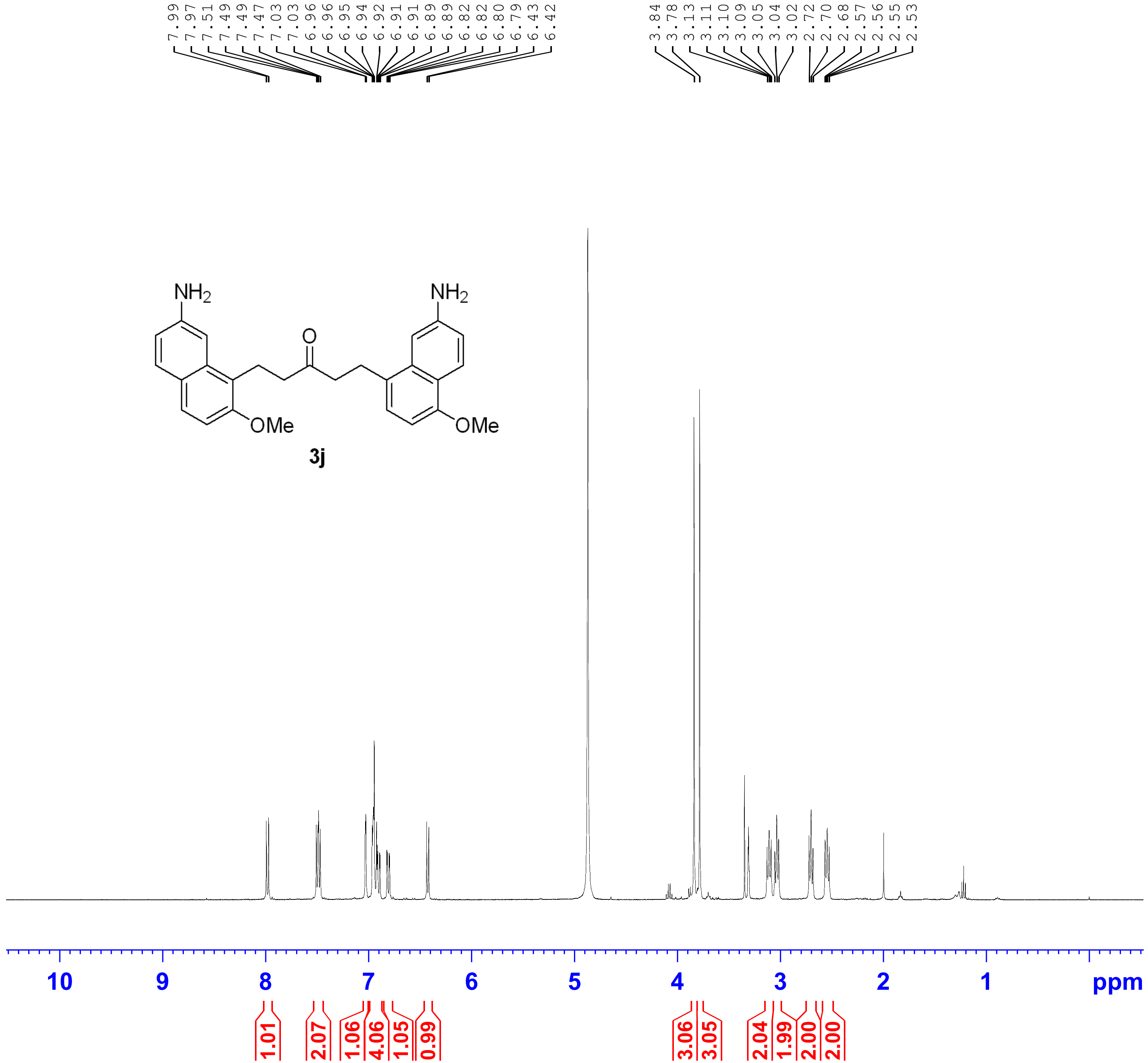

Current Data Parameters
NAME zrh-7-59-nh2-h
EXPNO 1
PROCNO 1

F2 - Acquisition Parameters
Date_ 20220728
Time 19.24
INSTRUM spect
PROBHD 5 mm PABBO BB/
PULPROG zg30
TD 65536
SOLVENT MeOD
NS 2
DS 0
SWH 8012.820 Hz
FIDRES 0.122266 Hz
AQ 4.0894465 sec
RG 49.32
DW 62.400 usec
DE 6.50 usec
TE 296.4 K
D1 1.00000000 sec
TD0 1

======== CHANNEL f1 ========
SFO1 400.1324710 MHz
NUC1 1H
P1 14.50 usec
PLW1 11.99499989 W

F2 - Processing parameters
SI 65536
SF 400.1300079 MHz
WDW EM
SSB 0
LB 0.30 Hz
GB 0
PC 1.00

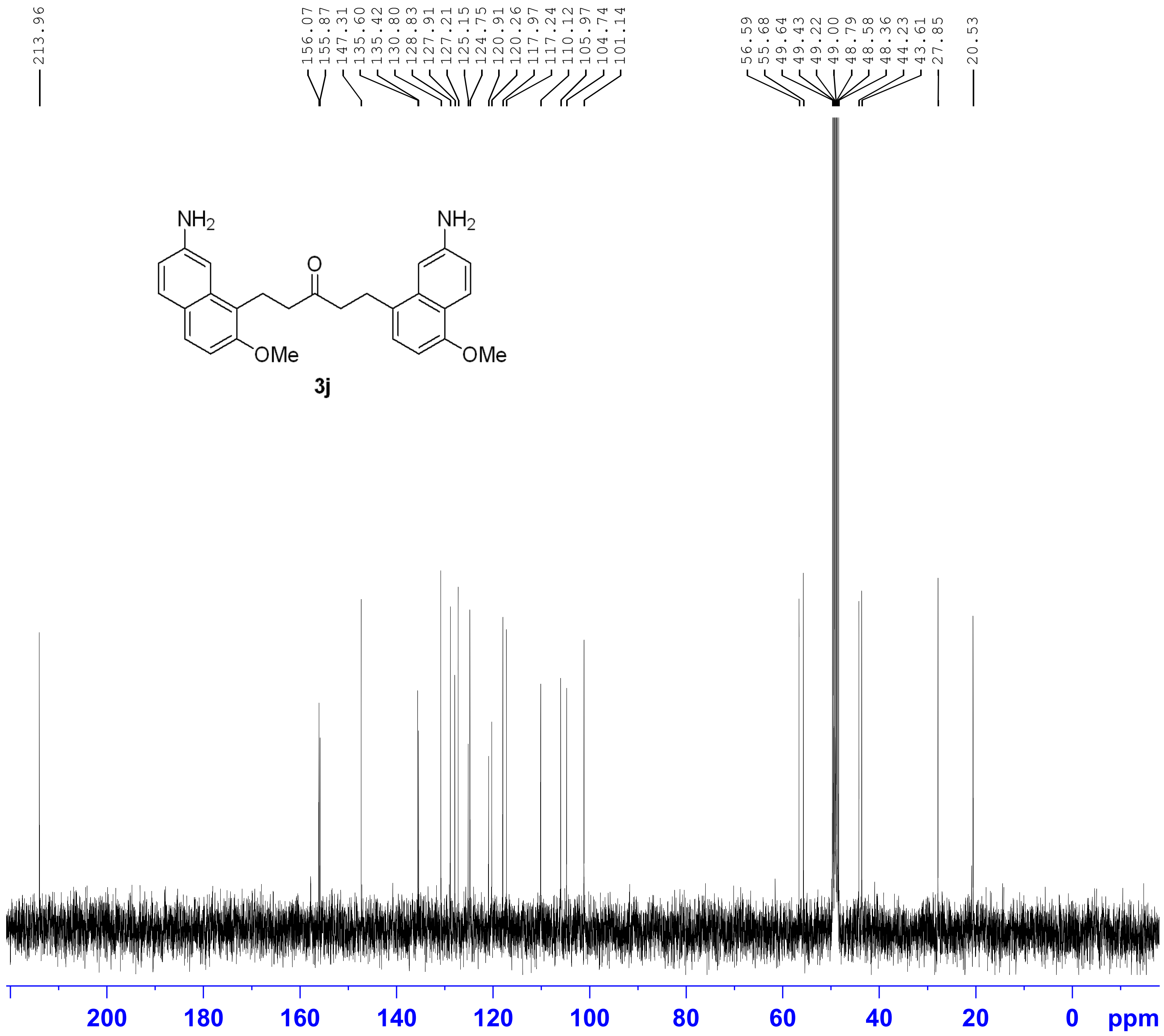

3j

```
Current Data Parameters
NAME      zrh-7-59-nh2-c
EXPNO                  1
PROCNO                 1

F2 - Acquisition Parameters
Date_           20220728
Time               19.26
INSTRUM            spect
PROBHD    5 mm PABBO BB/
PULPROG          zgpg30
TD                 65536
SOLVENT             MeOD
NS                    51
DS                     0
SWH            24038.461 Hz
FIDRES          0.366798 Hz
AQ             1.3631488 sec
RG                196.92
DW                20.800 usec
DE                  6.50 usec
TE                 297.0 K
D1            2.00000000 sec
D11           0.03000000 sec
TD0                    1

======== CHANNEL f1 ========
SFO1         100.6228298 MHz
NUC1                 13C
P1                  9.70 usec
PLW1         46.98899841 W

======== CHANNEL f2 ========
SFO2         400.1316005 MHz
NUC2                  1H
CPDPRG[2         waltz16
PCPD2              90.00 usec
PLW2         11.99499989 W
PLW12         0.34213999 W
PLW13         0.27713001 W

F2 - Processing parameters
SI                 32768
SF           100.6126295 MHz
WDW                   EM
SSB                    0
LB                  1.00 Hz
GB                     0
PC                  1.40
```

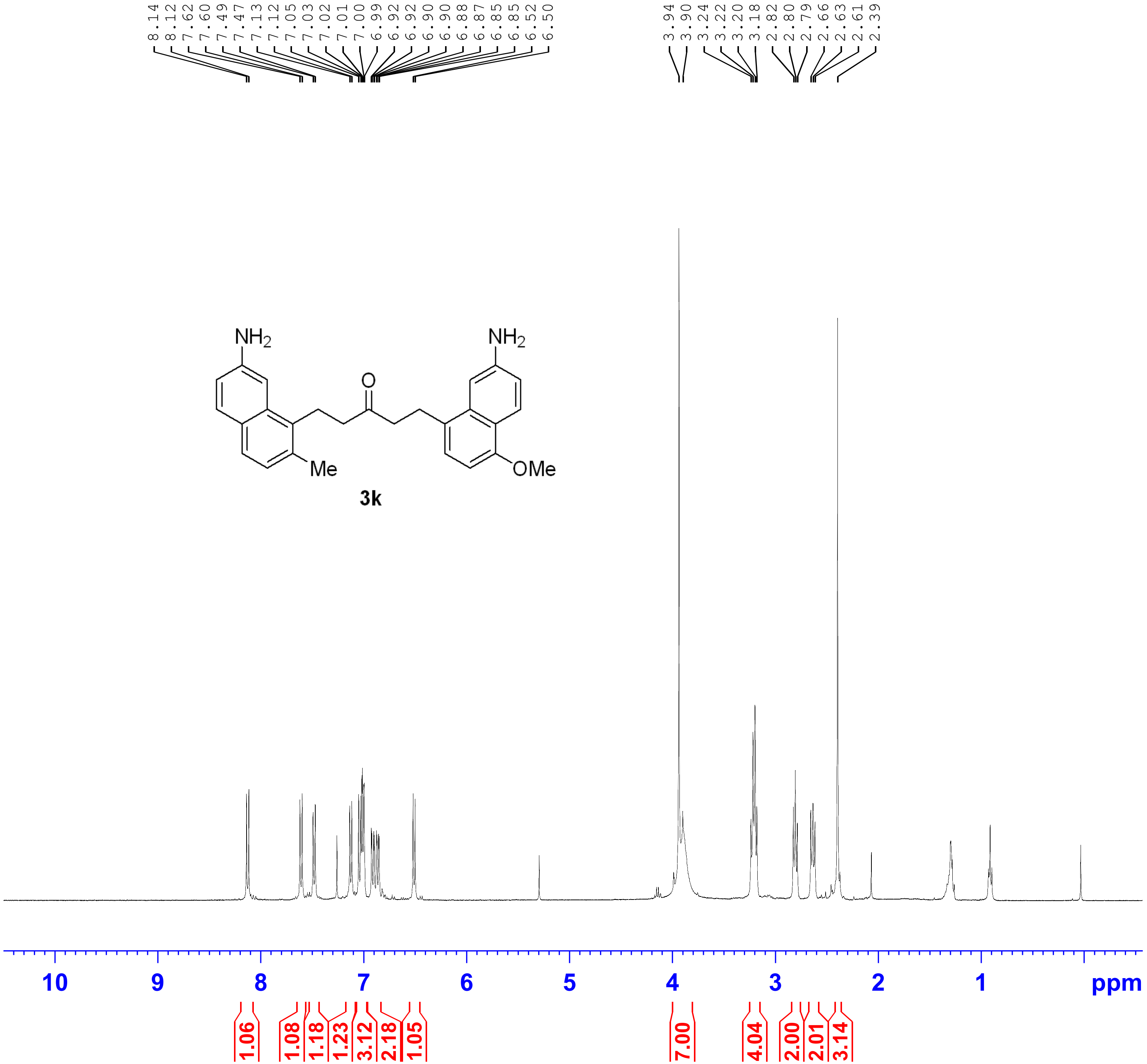

3k

```
Current Data Parameters
NAME       zrh-8-94-NH2-h
EXPNO                   1
PROCNO                  1

F2 - Acquisition Parameters
Date_            20221224
Time                19.09
INSTRUM             spect
PROBHD    5 mm DUL 13C-1
PULPROG              zg30
TD                  65536
SOLVENT             CDCl3
NS                      2
DS                      0
SWH              8223.685 Hz
FIDRES           0.125483 Hz
AQ              3.9845889 sec
RG                    181
DW                 60.800 usec
DE                   6.00 usec
TE                  295.0 K
D1             1.00000000 sec
TD0                     1

======== CHANNEL f1 ========
NUC1                   1H
P1                  15.80 usec
PL1                 -1.00 dB
PL1W          12.17476940 W
SFO1          400.1324710 MHz

F2 - Processing parameters
SI                  32768
SF            400.1300096 MHz
WDW                    EM
SSB                     0
LB                   0.30 Hz
GB                      0
PC                   1.00
```

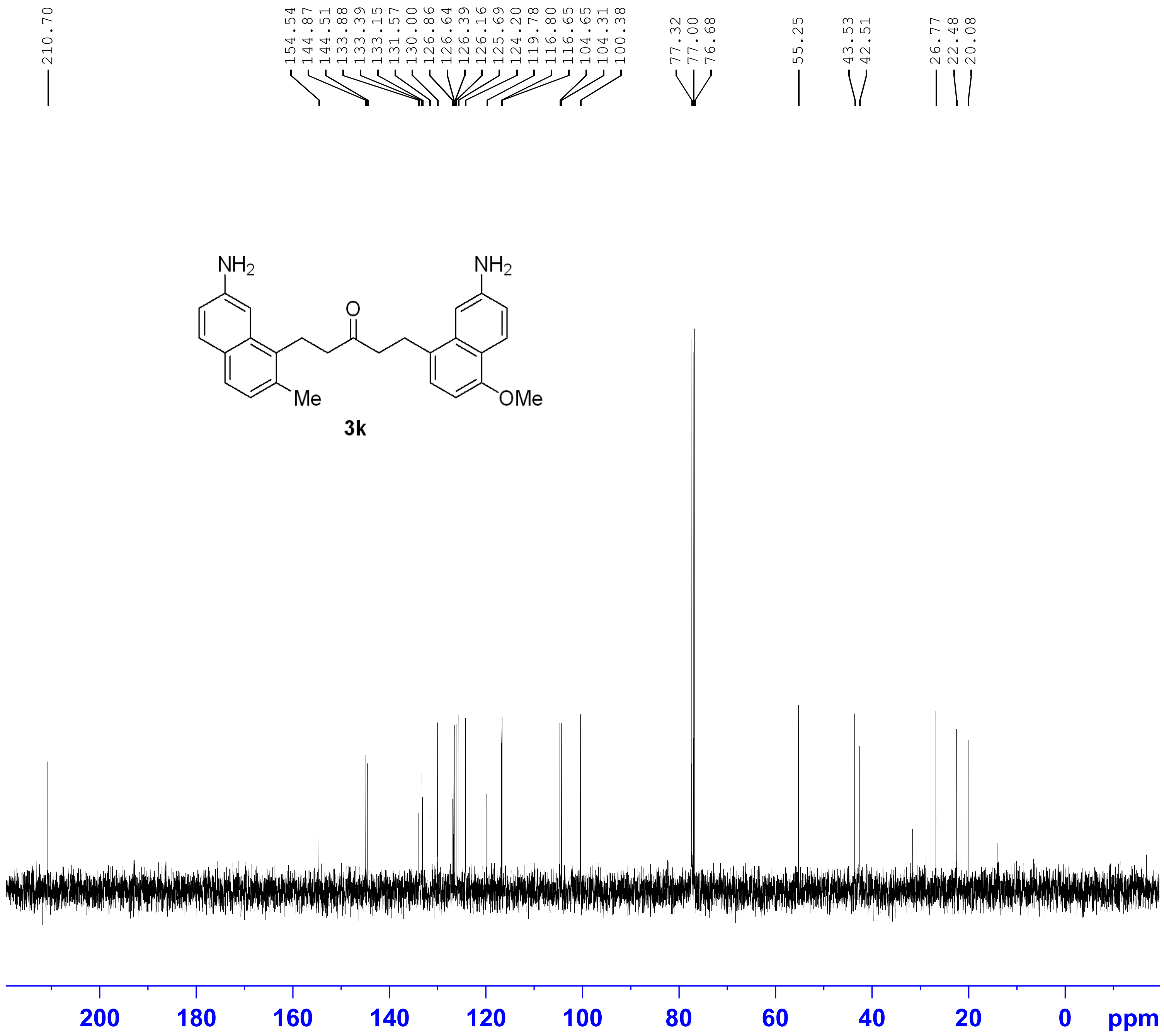

3k

Current Data Parameters
NAME zrh-8-94-NH2-c
EXPNO 1
PROCNO 1

F2 - Acquisition Parameters
Date_ 20221224
Time 19.10
INSTRUM spect
PROBHD 5 mm DUL 13C-1
PULPROG zgpg30
TD 65536
SOLVENT CDCl3
NS 52
DS 0
SWH 24038.461 Hz
FIDRES 0.366798 Hz
AQ 1.3631488 sec
RG 2050
DW 20.800 usec
DE 6.00 usec
TE 295.1 K
D1 2.00000000 sec
D11 0.03000000 sec
TD0 1

======== CHANNEL f1 ========
NUC1 13C
P1 40.00 usec
PL1 -3.00 dB
PL1W 60.64365387 W
SFO1 100.6228298 MHz

======== CHANNEL f2 ========
CPDPRG[2 waltz16
NUC2 1H
PCPD2 80.00 usec
PL2 -1.00 dB
PL12 14.39 dB
PL13 18.00 dB
PL2W 12.17476940 W
PL12W 0.35193357 W
PL13W 0.15327126 W
SFO2 400.1316005 MHz

F2 - Processing parameters
SI 32768
SF 100.6127794 MHz
WDW EM
SSB 0
LB 1.00 Hz
GB 0
PC 1.40

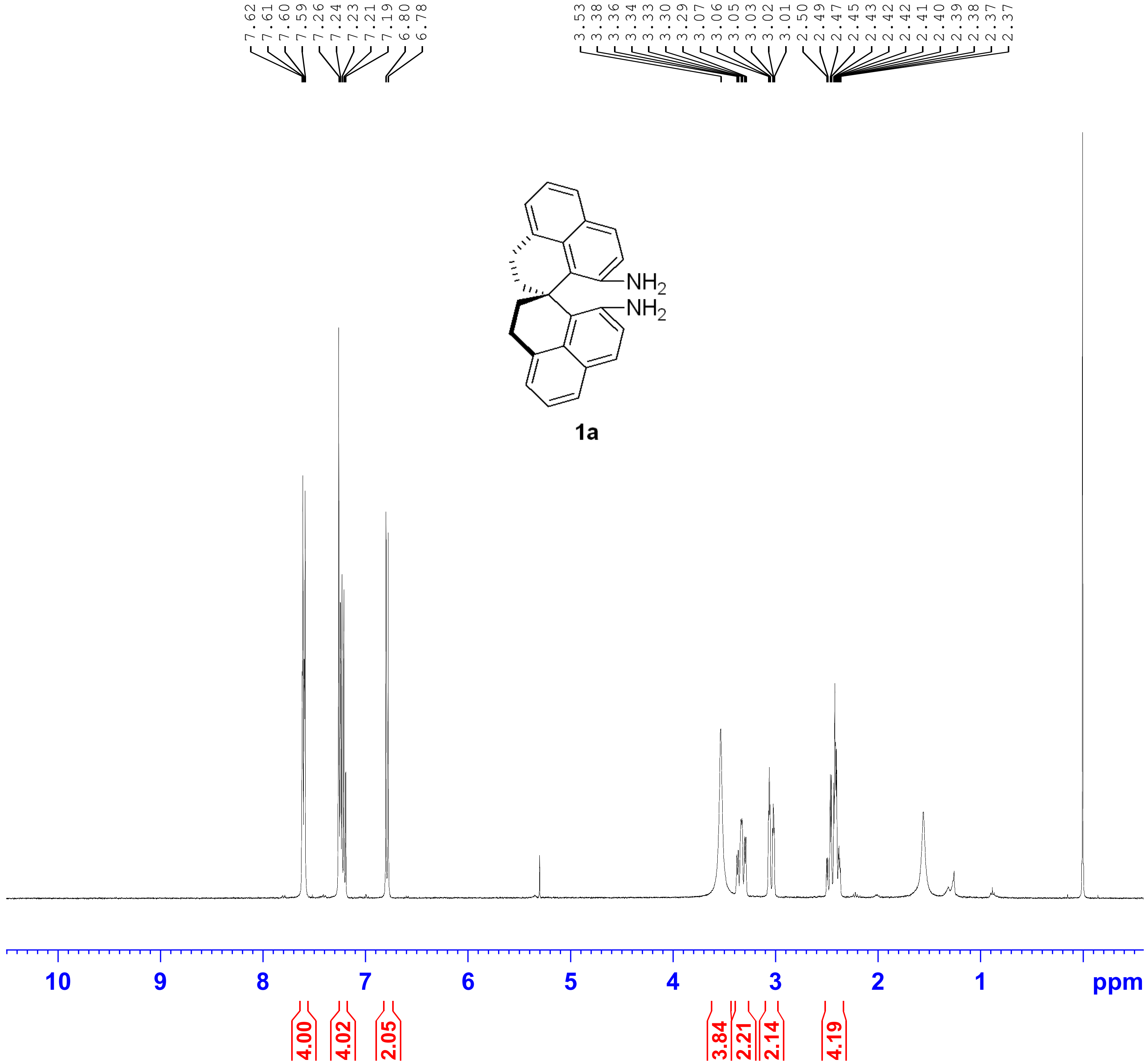


```
Current Data Parameters
NAME      zrh-5-148-1-sphenam-h
EXPNO                       1
PROCNO                      1

F2 - Acquisition Parameters
Date_              20220304
Time                  15.40
INSTRUM               spect
PROBHD   5 mm PABBO BB/
PULPROG                zg30
TD                    65536
SOLVENT               CDCl3
NS                       10
DS                        0
SWH                8012.820 Hz
FIDRES             0.122266 Hz
AQ                4.0894465 sec
RG                   196.92
DW                   62.400 usec
DE                     6.50 usec
TE                    295.1 K
D1               1.00000000 sec
TD0                       1

======== CHANNEL f1 ========
SFO1            400.1324710 MHz
NUC1                     1H
P1                    14.50 usec
PLW1            11.99499989 W

F2 - Processing parameters
SI                    65536
SF              400.1300099 MHz
WDW                      EM
SSB                       0
LB                     0.30 Hz
GB                        0
PC                     1.00
```

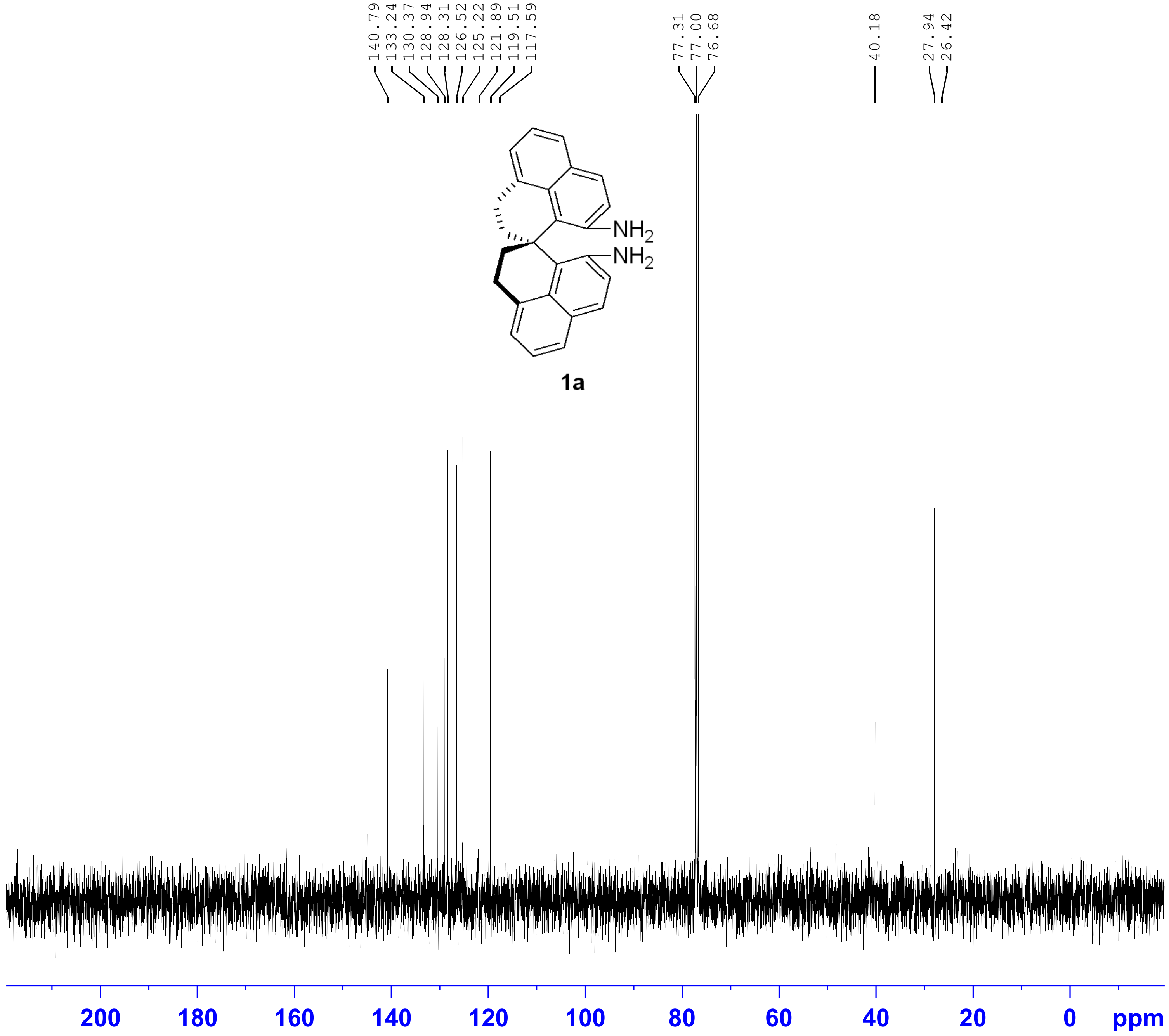

Current Data Parameters
NAME zrh-2-171-c
EXPNO 1
PROCNO 1

F2 - Acquisition Parameters
Date_ 20210405
Time 13.52
INSTRUM spect
PROBHD 5 mm DUL 13C-1
PULPROG zgpg30
TD 65536
SOLVENT CDCl3
NS 129
DS 0
SWH 24038.461 Hz
FIDRES 0.366798 Hz
AQ 1.3631488 sec
RG 2050
DW 20.800 usec
DE 6.00 usec
TE 293.9 K
D1 2.00000000 sec
D11 0.03000000 sec
TD0 1

======== CHANNEL f1 ========
NUC1 13C
P1 40.00 usec
PL1 -3.00 dB
PL1W 60.64365387 W
SFO1 100.6228298 MHz

======== CHANNEL f2 ========
CPDPRG[2 waltz16
NUC2 1H
PCPD2 80.00 usec
PL2 -1.00 dB
PL12 14.39 dB
PL13 18.00 dB
PL2W 12.17476940 W
PL12W 0.35193357 W
PL13W 0.15327126 W
SFO2 400.1316005 MHz

F2 - Processing parameters
SI 32768
SF 100.6127743 MHz
WDW EM
SSB 0
LB 1.00 Hz
GB 0
PC 1.40

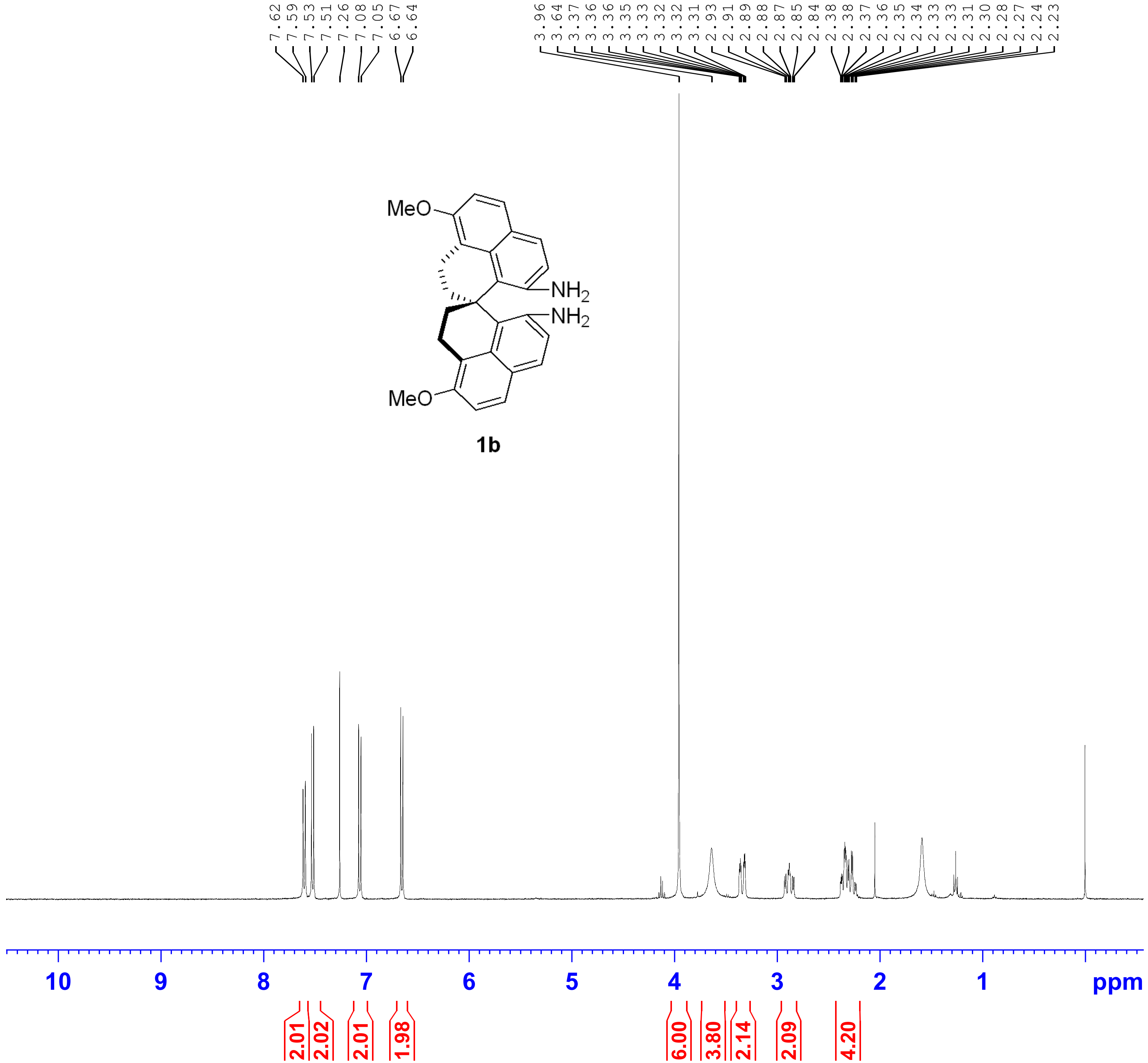


```
Current Data Parameters
NAME      zrh-6-120-sphenam-h
EXPNO                       1
PROCNO                      1

F2 - Acquisition Parameters
Date_                20220605
Time                    15.21
INSTRUM                 spect
PROBHD     5 mm PABBO BB/
PULPROG                  zg30
TD                      65536
SOLVENT                 CDCl3
NS                          2
DS                          0
SWH                  8012.820 Hz
FIDRES               0.122266 Hz
AQ                  4.0894465 sec
RG                     164.33
DW                     62.400 usec
DE                       6.50 usec
TE                      295.5 K
D1                 1.00000000 sec
TD0                         1

======== CHANNEL f1 ========
SFO1              400.1324710 MHz
NUC1                       1H
P1                      14.50 usec
PLW1              11.99499989 W

F2 - Processing parameters
SI                      65536
SF                400.1300101 MHz
WDW                        EM
SSB                         0
LB                       0.30 Hz
GB                          0
PC                       1.00
```

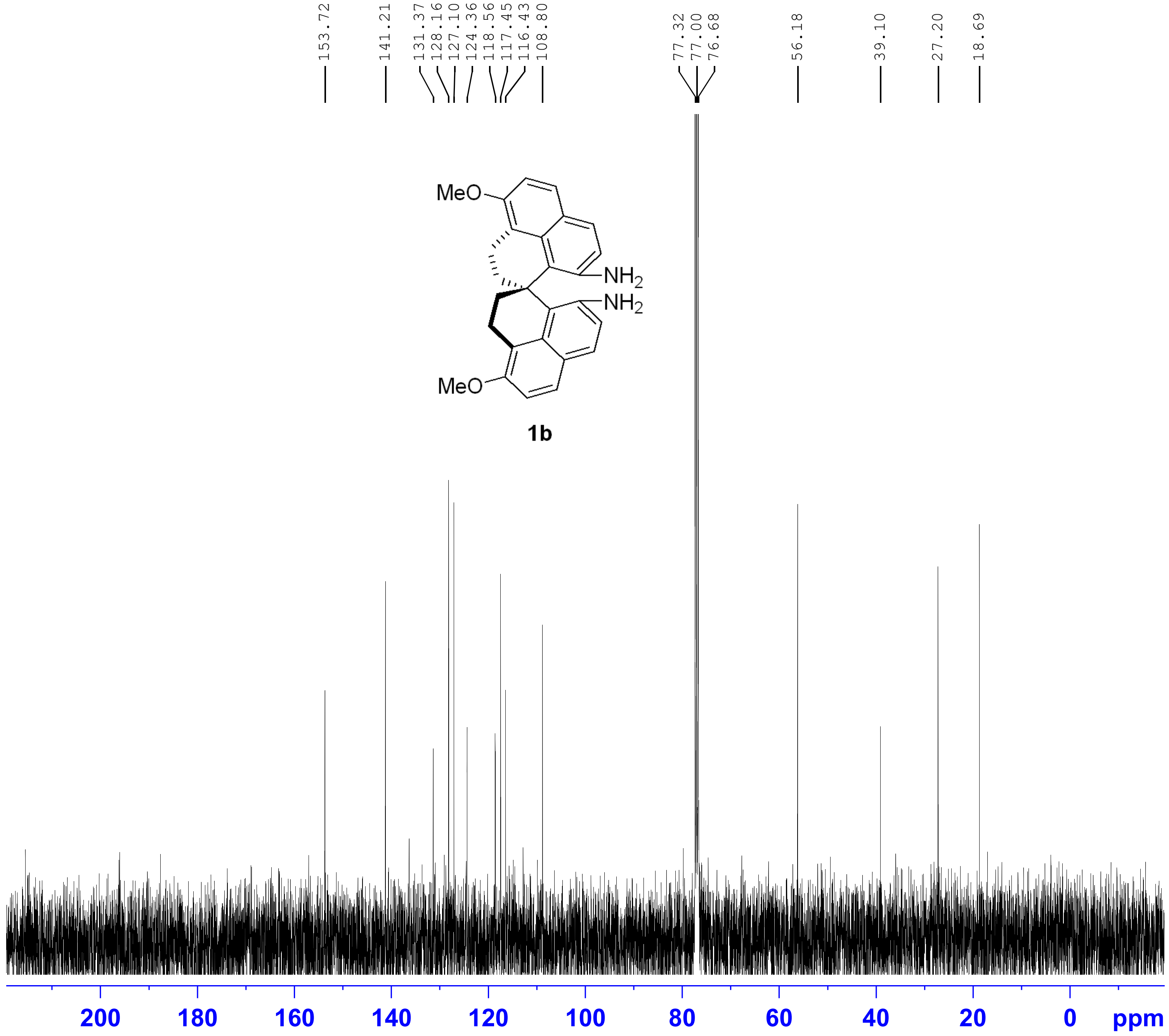

Current Data Parameters
NAME zrh-6-120-sphenam-c
EXPNO 1
PROCNO 1

F2 - Acquisition Parameters
Date_ 20220605
Time 15.22
INSTRUM spect
PROBHD 5 mm PABBO BB/
PULPROG zgpg30
TD 65536
SOLVENT CDCl3
NS 102
DS 0
SWH 24038.461 Hz
FIDRES 0.366798 Hz
AQ 1.3631488 sec
RG 196.92
DW 20.800 usec
DE 6.50 usec
TE 295.6 K
D1 2.00000000 sec
D11 0.03000000 sec
TD0 1

======== CHANNEL f1 ========
SFO1 100.6228298 MHz
NUC1 13C
P1 9.70 usec
PLW1 46.98899841 W

======== CHANNEL f2 ========
SFO2 400.1316005 MHz
NUC2 1H
CPDPRG[2 waltz16
PCPD2 90.00 usec
PLW2 11.99499989 W
PLW12 0.34213999 W
PLW13 0.27713001 W

F2 - Processing parameters
SI 32768
SF 100.6127722 MHz
WDW EM
SSB 0
LB 1.00 Hz
GB 0
PC 1.40

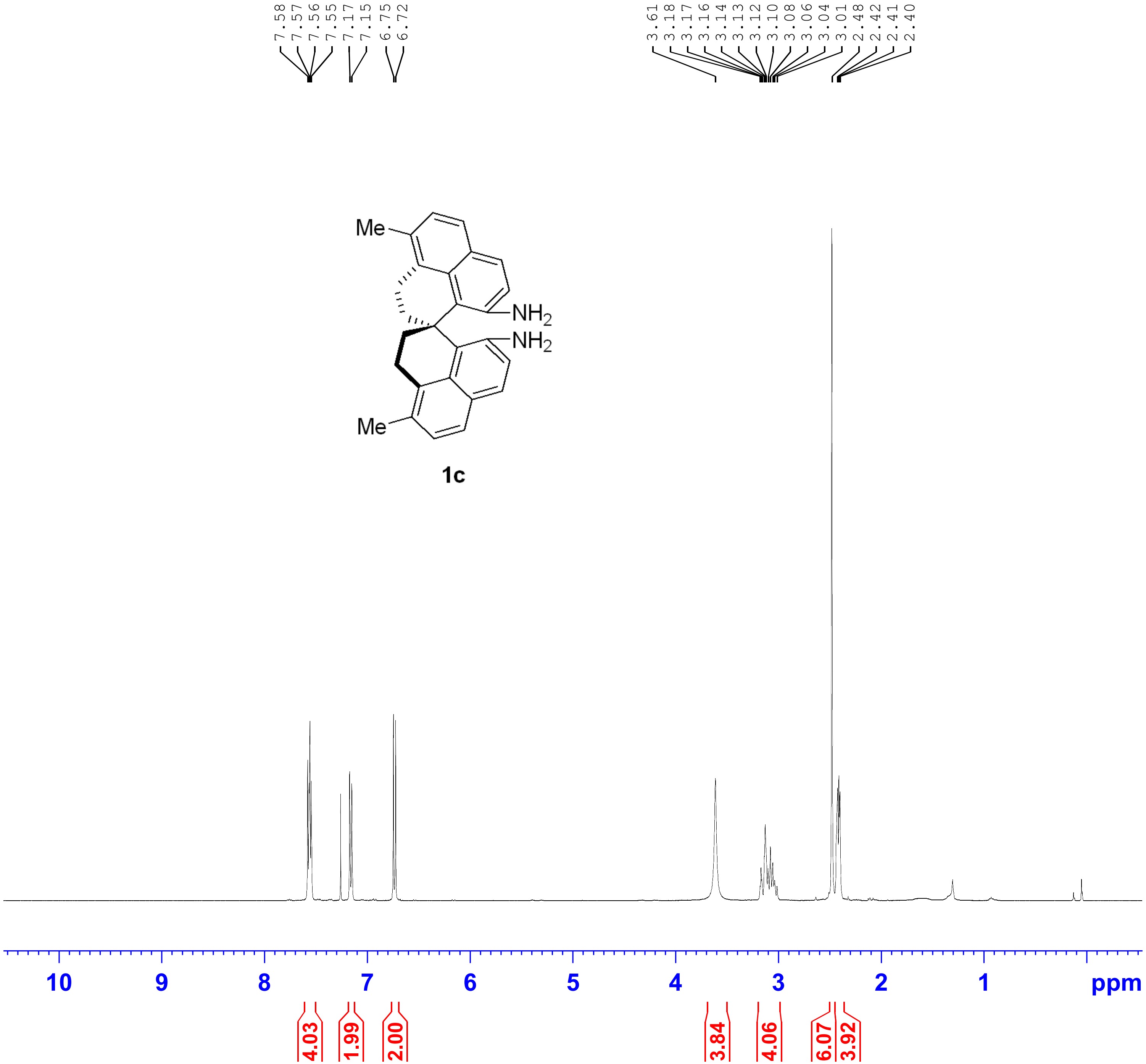


```
Current Data Parameters
NAME             zrh-7-4-h
EXPNO                    1
PROCNO                   1

F2 - Acquisition Parameters
Date_             20220702
Time                 20.10
INSTRUM              spect
PROBHD   5 mm DUL 13C-1
PULPROG               zg30
TD                   65536
SOLVENT              CDCl3
NS                       2
DS                       0
SWH               8223.685 Hz
FIDRES            0.125483 Hz
AQ               3.9845889 sec
RG                     228
DW                  60.800 usec
DE                    6.00 usec
TE                   295.1 K
D1              1.00000000 sec
TD0                      1

======== CHANNEL f1 ========
NUC1                    1H
P1                   15.80 usec
PL1                  -1.00 dB
PL1W           12.17476940 W
SFO1           400.1324710 MHz

F2 - Processing parameters
SI                   32768
SF             400.1300100 MHz
WDW                     EM
SSB                      0
LB                    0.30 Hz
GB                       0
PC                    1.00
```

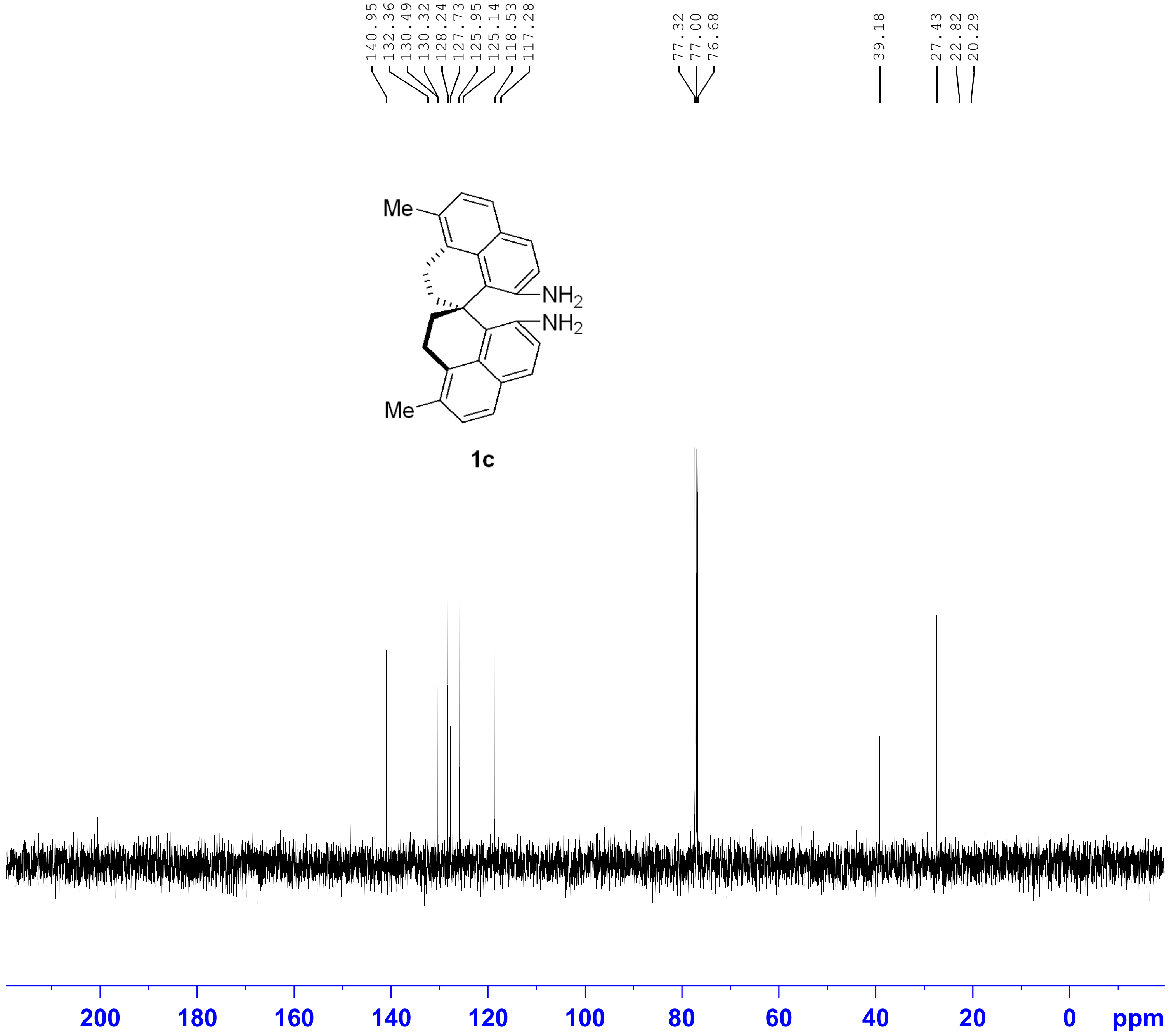

Current Data Parameters
NAME zrh-7-4-c
EXPNO 1
PROCNO 1

F2 - Acquisition Parameters
Date_ 20220702
Time 20.11
INSTRUM spect
PROBHD 5 mm DUL 13C-1
PULPROG zgpg30
TD 65536
SOLVENT CDCl3
NS 20
DS 0
SWH 24038.461 Hz
FIDRES 0.366798 Hz
AQ 1.3631488 sec
RG 2050
DW 20.800 usec
DE 6.00 usec
TE 295.2 K
D1 2.00000000 sec
D11 0.03000000 sec
TD0 1

======== CHANNEL f1 ========
NUC1 13C
P1 40.00 usec
PL1 -3.00 dB
PL1W 60.64365387 W
SFO1 100.6228298 MHz

======== CHANNEL f2 ========
CPDPRG[2 waltz16
NUC2 1H
PCPD2 80.00 usec
PL2 -1.00 dB
PL12 14.39 dB
PL13 18.00 dB
PL2W 12.17476940 W
PL12W 0.35193357 W
PL13W 0.15327126 W
SFO2 400.1316005 MHz

F2 - Processing parameters
SI 32768
SF 100.6127788 MHz
WDW EM
SSB 0
LB 1.00 Hz
GB 0
PC 1.40

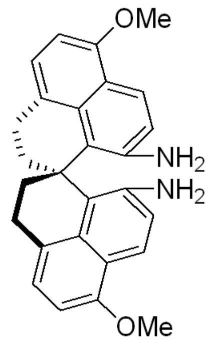

1d

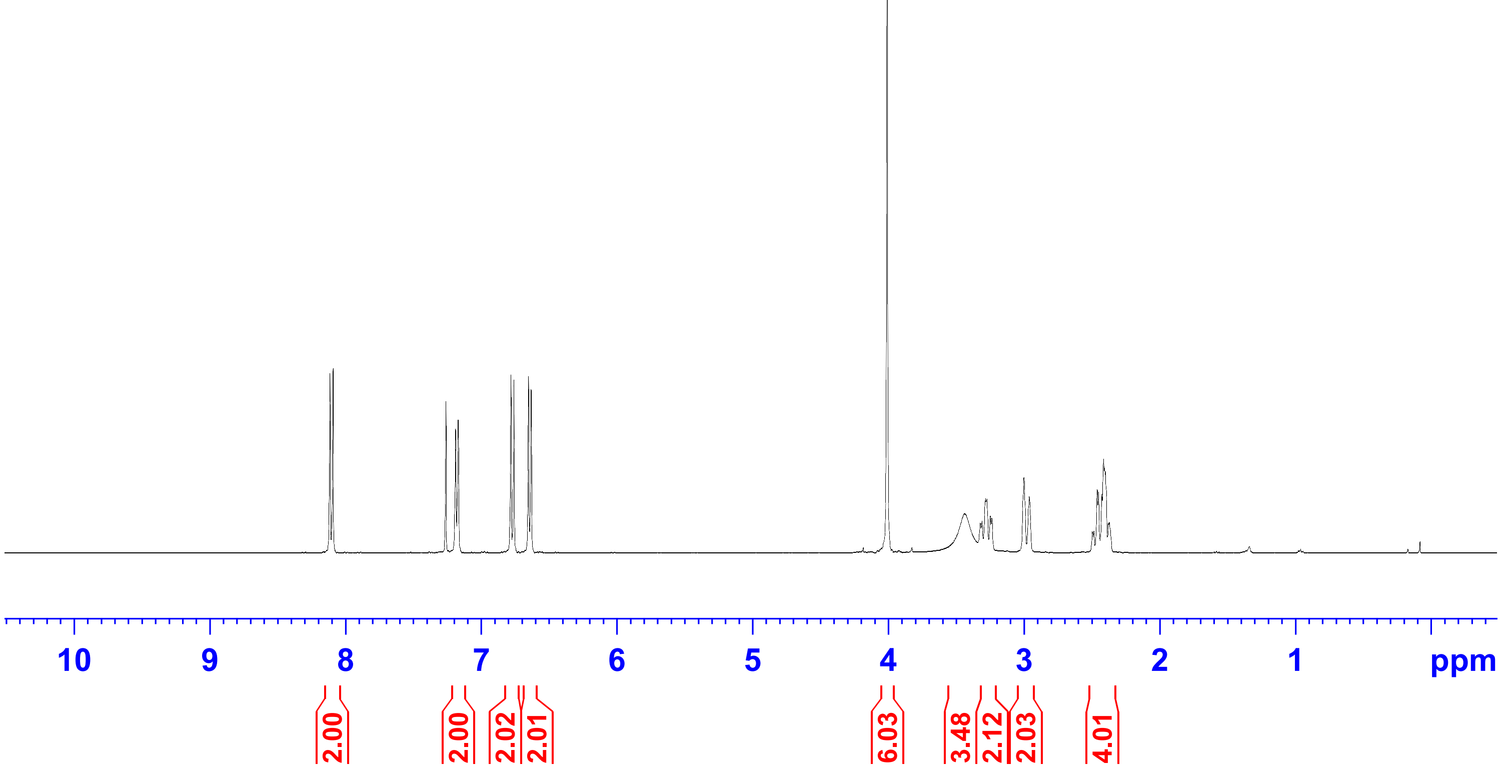

Current Data Parameters
NAME zrh-7-57-h
EXPNO 1
PROCNO 1

F2 - Acquisition Parameters
Date_ 20220730
Time 19.57
INSTRUM spect
PROBHD 5 mm DUL 13C-1
PULPROG zg30
TD 65536
SOLVENT CDCl3
NS 1
DS 0
SWH 8223.685 Hz
FIDRES 0.125483 Hz
AQ 3.9845889 sec
RG 114
DW 60.800 usec
DE 6.00 usec
TE 295.0 K
D1 1.00000000 sec
TD0 1

======== CHANNEL f1 ========
NUC1 1H
P1 15.80 usec
PL1 -1.00 dB
PL1W 12.17476940 W
SFO1 400.1324710 MHz

F2 - Processing parameters
SI 32768
SF 400.1300093 MHz
WDW EM
SSB 0
LB 0.30 Hz
GB 0
PC 1.00

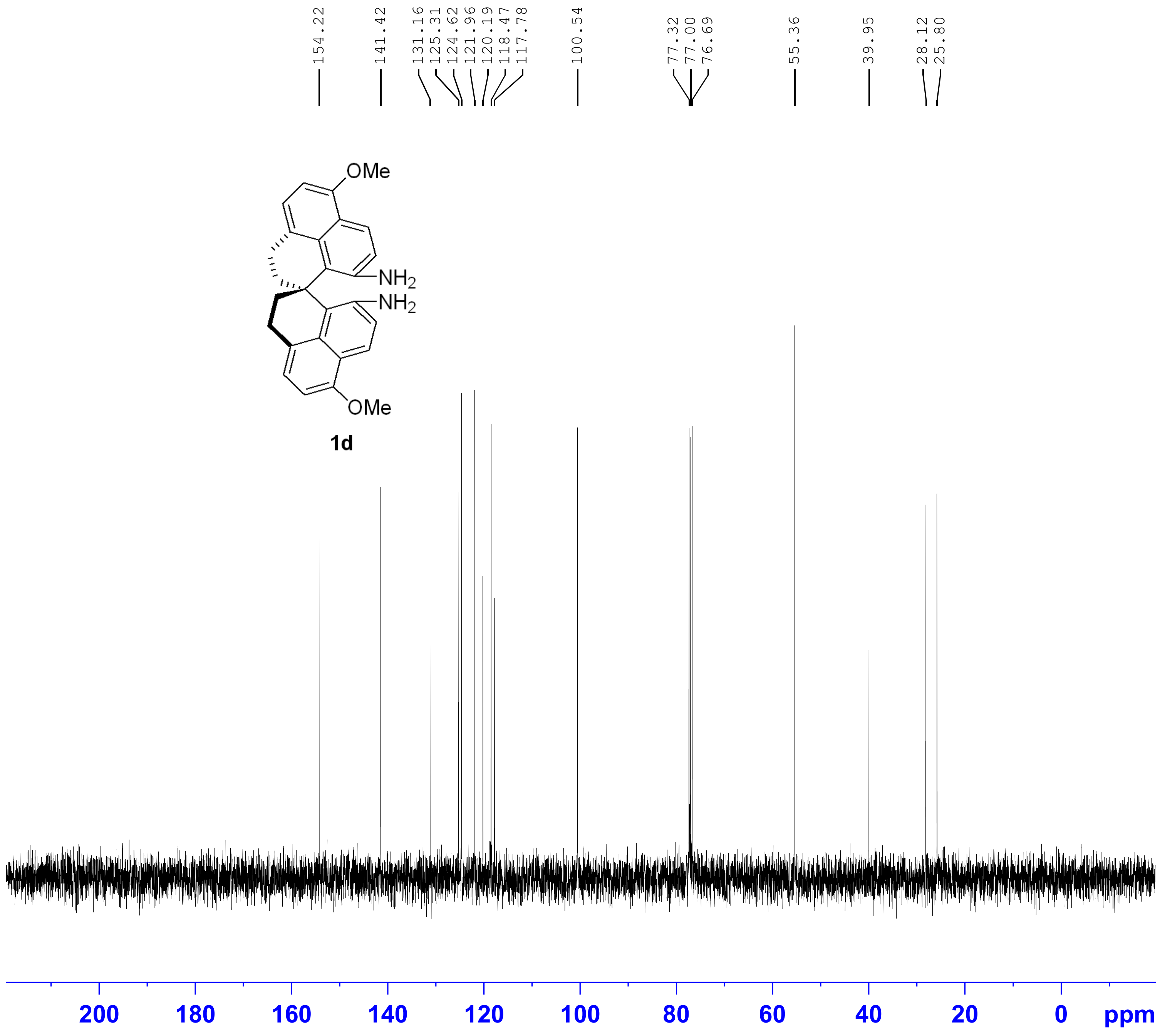

1d

```
Current Data Parameters
NAME          zrh-7-57-c
EXPNO                  1
PROCNO                 1

F2 - Acquisition Parameters
Date_           20220730
Time               19.58
INSTRUM            spect
PROBHD   5 mm DUL 13C-1
PULPROG          zgpg30
TD                 65536
SOLVENT            CDCl3
NS                    13
DS                     0
SWH            24038.461 Hz
FIDRES          0.366798 Hz
AQ             1.3631488 sec
RG                  2050
DW                20.800 usec
DE                  6.00 usec
TE                 295.2 K
D1            2.00000000 sec
D11           0.03000000 sec
TD0                    1

======== CHANNEL f1 ========
NUC1                 13C
P1                 40.00 usec
PL1                -3.00 dB
PL1W         60.64365387 W
SFO1         100.6228298 MHz

======== CHANNEL f2 ========
CPDPRG[2         waltz16
NUC2                  1H
PCPD2              80.00 usec
PL2                -1.00 dB
PL12               14.39 dB
PL13               18.00 dB
PL2W         12.17476940 W
PL12W         0.35193357 W
PL13W         0.15327126 W
SFO2         400.1316005 MHz

F2 - Processing parameters
SI                 32768
SF           100.6127854 MHz
WDW                   EM
SSB                    0
LB                  1.00 Hz
GB                     0
PC                  1.40
```

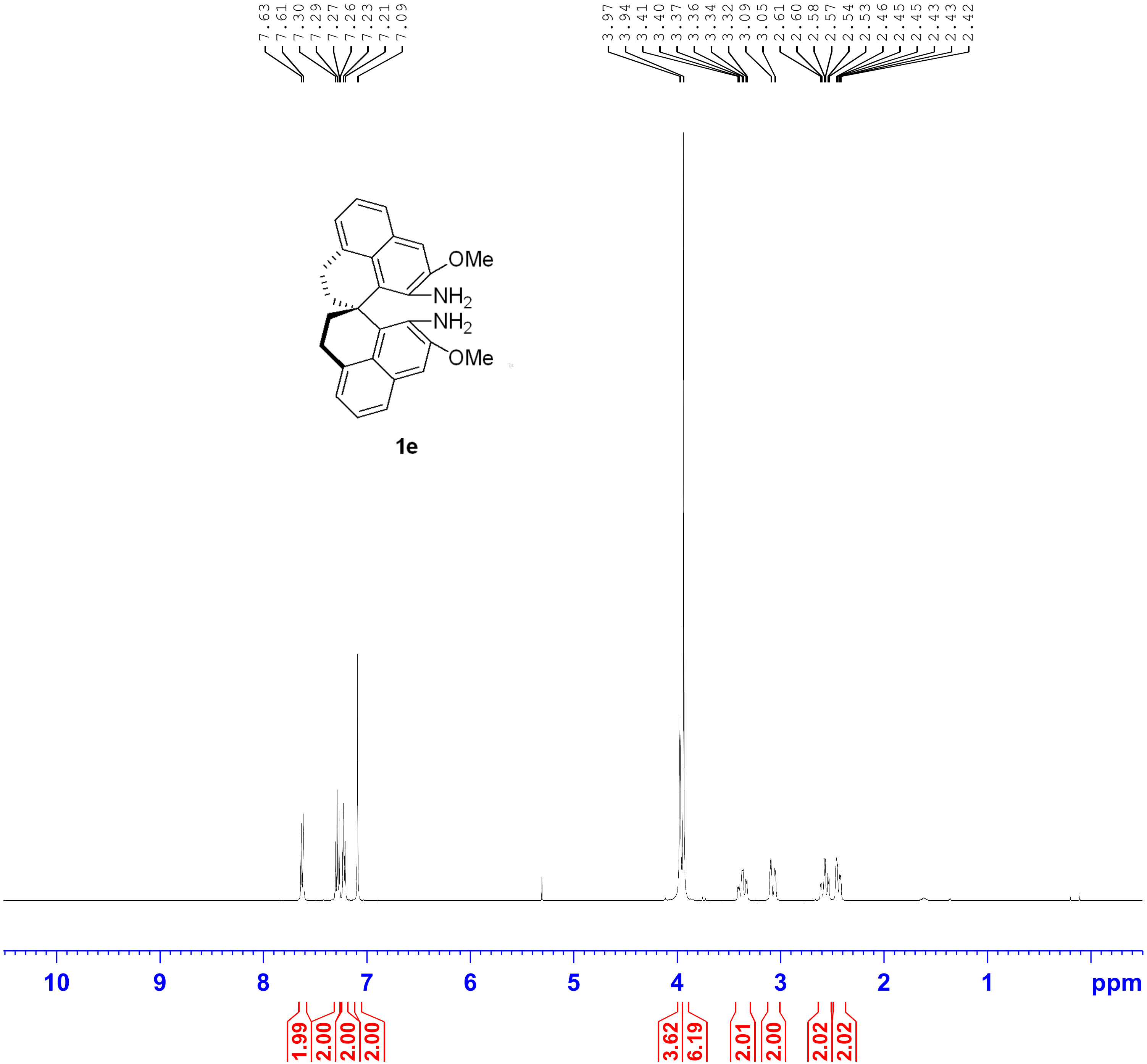


```
Current Data Parameters
NAME       zrh-6-151-SPHENAM-large-h
EXPNO                       1
PROCNO                      1

F2 - Acquisition Parameters
Date_             20220615
Time                 20.30
INSTRUM              spect
PROBHD    5 mm DUL 13C-1
PULPROG               zg30
TD                   65536
SOLVENT              CDCl3
NS                       2
DS                       0
SWH               8223.685 Hz
FIDRES            0.125483 Hz
AQ               3.9845889 sec
RG                      64
DW                  60.800 usec
DE                    6.00 usec
TE                   292.6 K
D1              1.00000000 sec
TD0                      1

======== CHANNEL f1 ========
NUC1                    1H
P1                   15.80 usec
PL1                  -1.00 dB
PL1W           12.17476940 W
SFO1           400.1324710 MHz

F2 - Processing parameters
SI                   32768
SF             400.1300098 MHz
WDW                     EM
SSB                      0
LB                    0.30 Hz
GB                       0
PC                    1.00
```

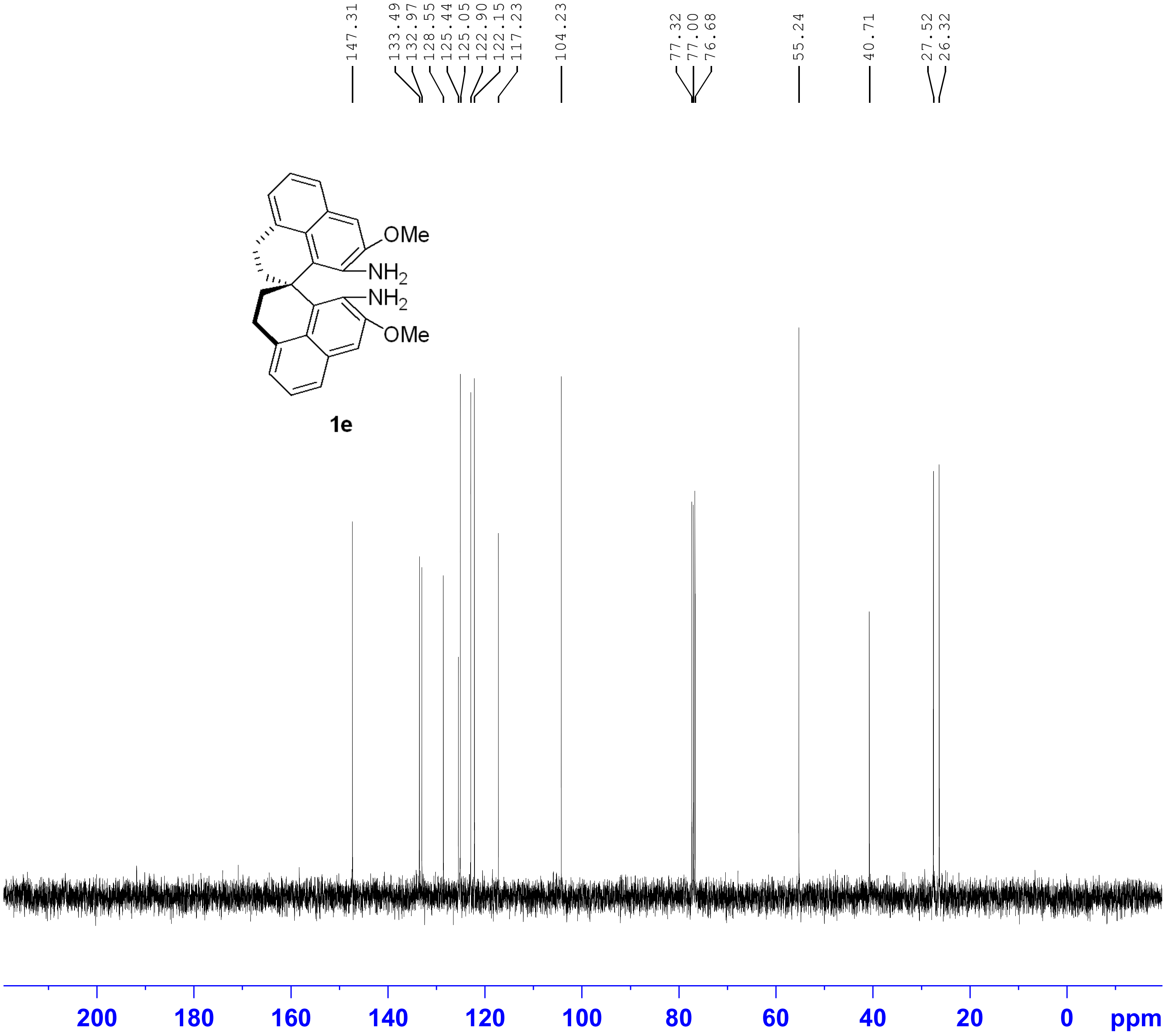


```
Current Data Parameters
NAME      zrh-6-151-SPHENAM-large-c
EXPNO                     1
PROCNO                    1

F2 - Acquisition Parameters
Date_              20220615
Time                  20.32
INSTRUM               spect
PROBHD   5 mm DUL 13C-1
PULPROG             zgpg30
TD                   65536
SOLVENT              CDCl3
NS                      21
DS                       0
SWH              24038.461 Hz
FIDRES            0.366798 Hz
AQ               1.3631488 sec
RG                    2050
DW                  20.800 usec
DE                    6.00 usec
TE                   292.7 K
D1              2.00000000 sec
D11             0.03000000 sec
TD0                      1

======== CHANNEL f1 ========
NUC1                   13C
P1                   40.00 usec
PL1                  -3.00 dB
PL1W           60.64365387 W
SFO1           100.6228298 MHz

======== CHANNEL f2 ========
CPDPRG[2           waltz16
NUC2                    1H
PCPD2                80.00 usec
PL2                  -1.00 dB
PL12                 14.39 dB
PL13                 18.00 dB
PL2W           12.17476940 W
PL12W           0.35193357 W
PL13W           0.15327126 W
SFO2           400.1316005 MHz

F2 - Processing parameters
SI                   32768
SF             100.6127897 MHz
WDW                     EM
SSB                      0
LB                    1.00 Hz
GB                       0
PC                    1.40
```

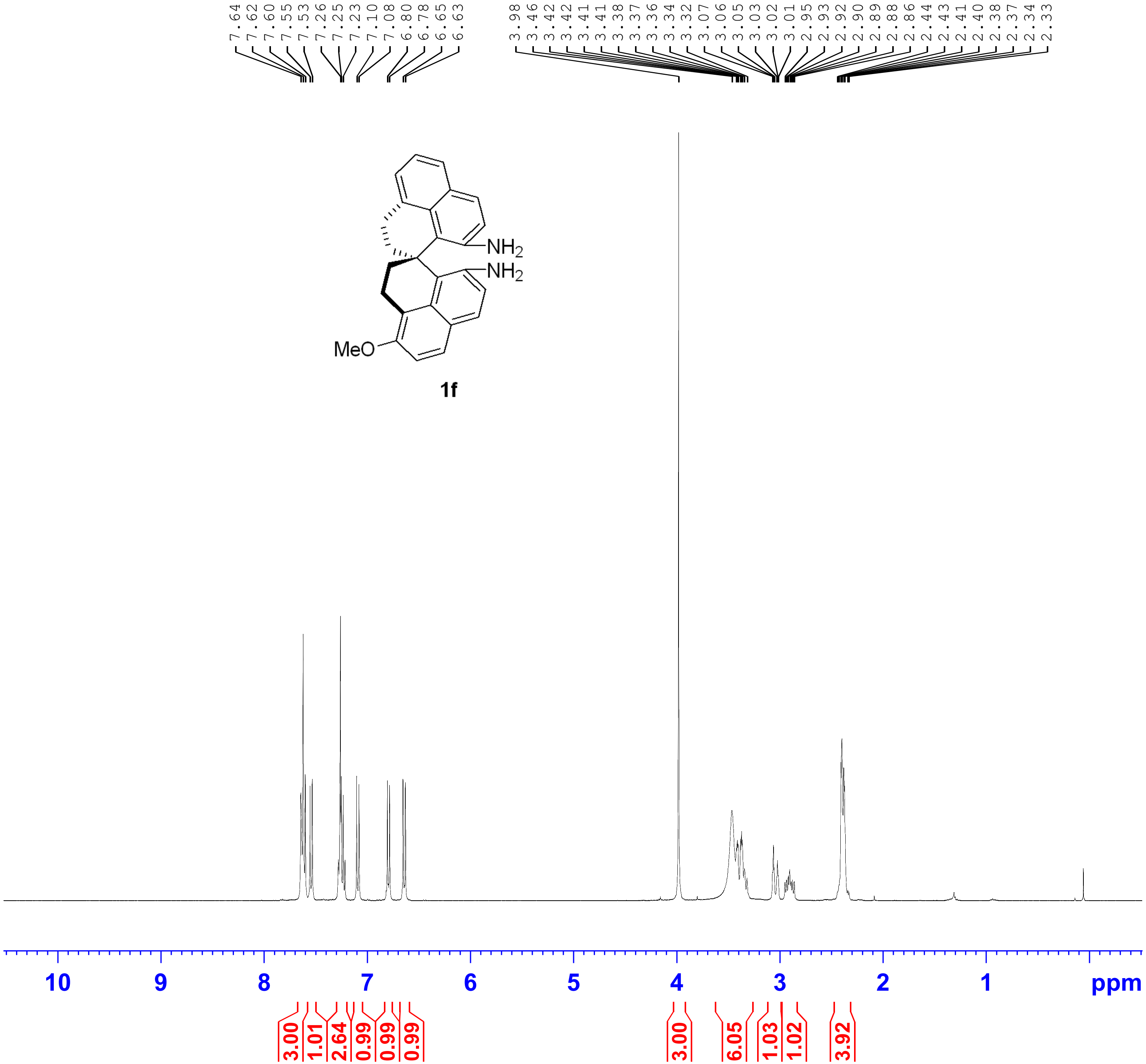

1f

Current Data Parameters
NAME zrh-6-177-large-h
EXPNO 1
PROCNO 1

F2 - Acquisition Parameters
Date_ 20220624
Time 19.00
INSTRUM spect
PROBHD 5 mm DUL 13C-1
PULPROG zg30
TD 65536
SOLVENT CDCl3
NS 2
DS 0
SWH 8223.685 Hz
FIDRES 0.125483 Hz
AQ 3.9845889 sec
RG 144
DW 60.800 usec
DE 6.00 usec
TE 292.8 K
D1 1.00000000 sec
TD0 1

======== CHANNEL f1 ========
NUC1 1H
P1 15.80 usec
PL1 -1.00 dB
PL1W 12.17476940 W
SFO1 400.1324710 MHz

F2 - Processing parameters
SI 32768
SF 400.1300099 MHz
WDW EM
SSB 0
LB 0.30 Hz
GB 0
PC 1.00

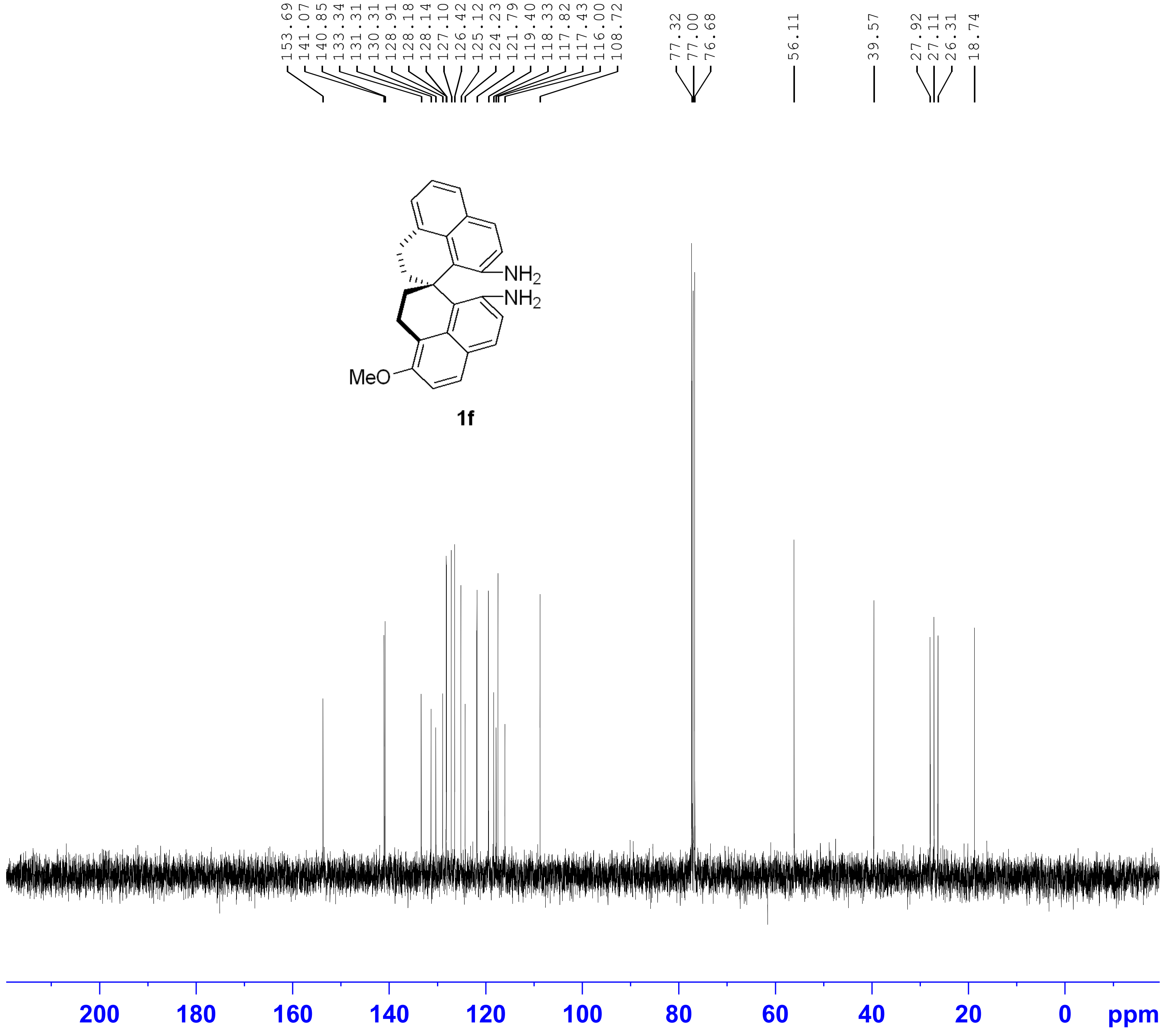


```
Current Data Parameters
NAME        zrh-6-177-large-c
EXPNO                       1
PROCNO                      1

F2 - Acquisition Parameters
Date_              20220624
Time                  19.03
INSTRUM               spect
PROBHD     5 mm DUL 13C-1
PULPROG             zgpg30
TD                   65536
SOLVENT              CDCl3
NS                      50
DS                       0
SWH              24038.461 Hz
FIDRES            0.366798 Hz
AQ               1.3631488 sec
RG                    2050
DW                  20.800 usec
DE                    6.00 usec
TE                   292.9 K
D1              2.00000000 sec
D11             0.03000000 sec
TD0                      1

======== CHANNEL f1 ========
NUC1                   13C
P1                   40.00 usec
PL1                  -3.00 dB
PL1W           60.64365387 W
SFO1           100.6228298 MHz

======== CHANNEL f2 ========
CPDPRG[2           waltz16
NUC2                    1H
PCPD2                80.00 usec
PL2                  -1.00 dB
PL12                 14.39 dB
PL13                 18.00 dB
PL2W           12.17476940 W
PL12W           0.35193357 W
PL13W           0.15327126 W
SFO2           400.1316005 MHz

F2 - Processing parameters
SI                   32768
SF             100.6127823 MHz
WDW                     EM
SSB                      0
LB                    1.00 Hz
GB                       0
PC                    1.40
```

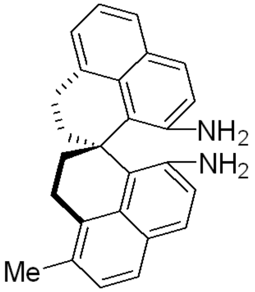

1g

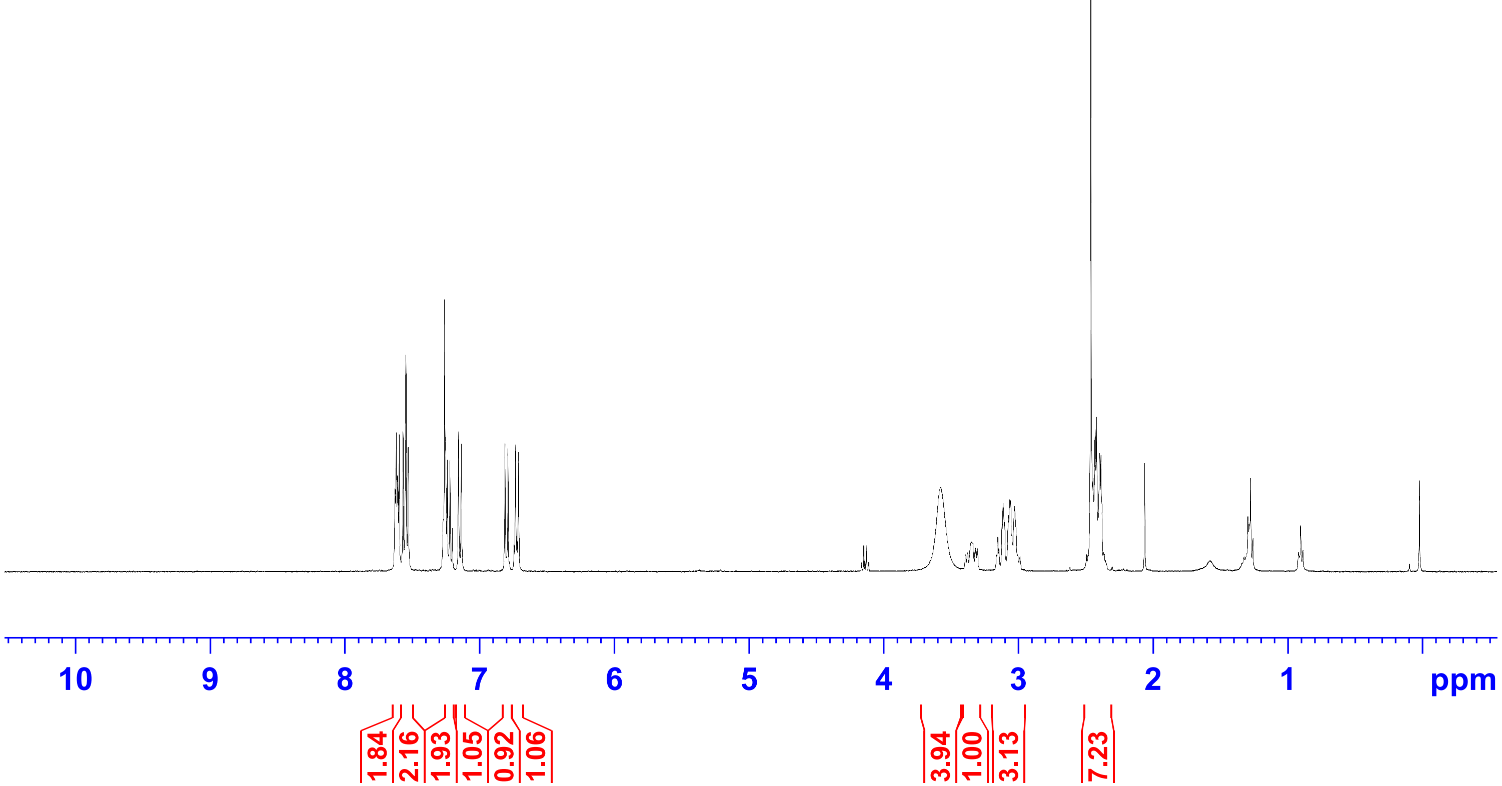

Current Data Parameters
NAME zrh-8-107-h
EXPNO 1
PROCNO 1

F2 - Acquisition Parameters
Date_ 20221230
Time 19.28
INSTRUM spect
PROBHD 5 mm DUL 13C-1
PULPROG zg30
TD 65536
SOLVENT CDCl3
NS 4
DS 0
SWH 8223.685 Hz
FIDRES 0.125483 Hz
AQ 3.9845889 sec
RG 287
DW 60.800 usec
DE 6.00 usec
TE 293.0 K
D1 1.00000000 sec
TD0 1

======== CHANNEL f1 ========
NUC1 1H
P1 15.80 usec
PL1 -1.00 dB
PL1W 12.17476940 W
SFO1 400.1324710 MHz

F2 - Processing parameters
SI 32768
SF 400.1300099 MHz
WDW EM
SSB 0
LB 0.30 Hz
GB 0
PC 1.00

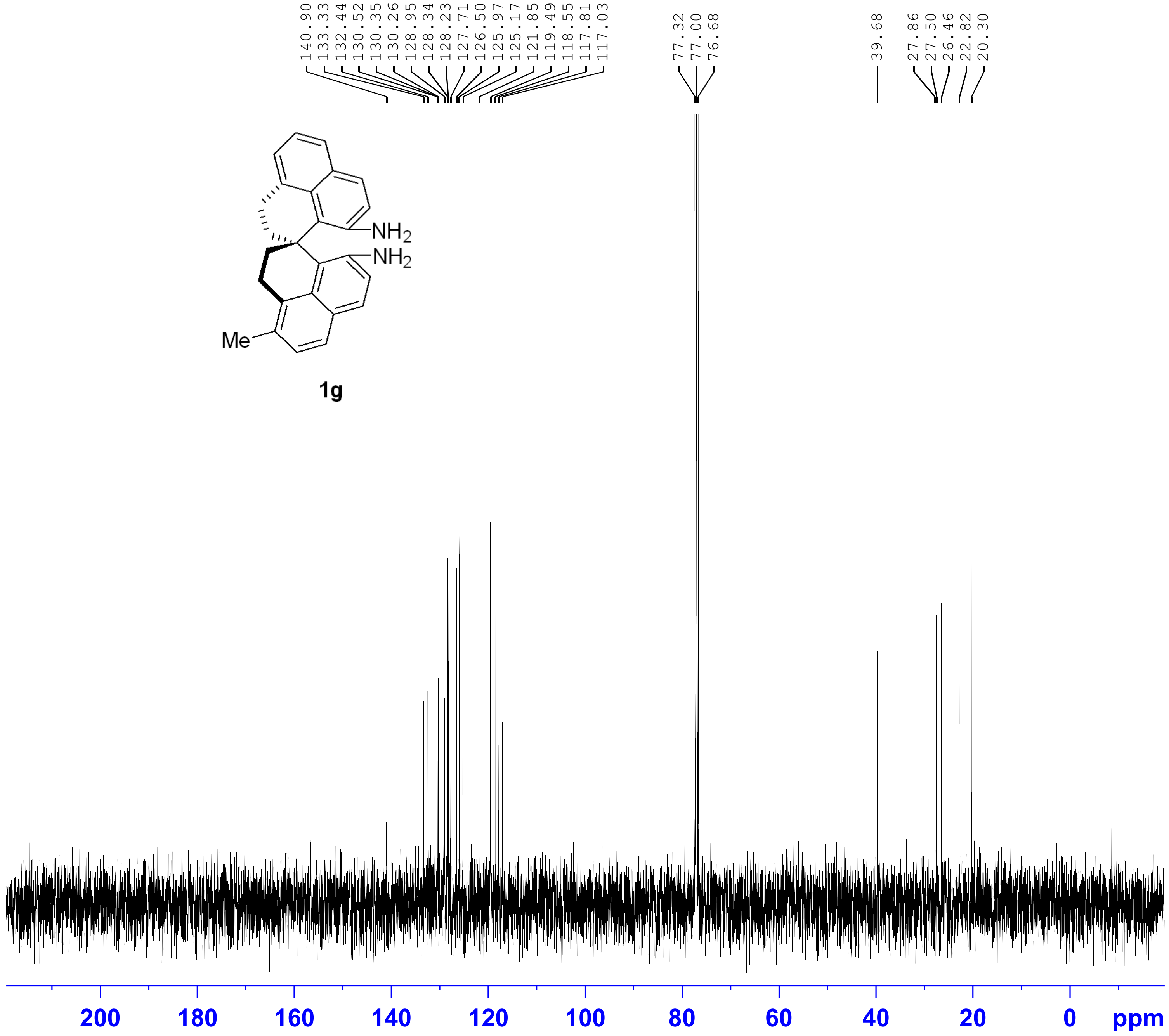


```
Current Data Parameters
NAME          zrh-8-107-c
EXPNO                   1
PROCNO                  1

F2 - Acquisition Parameters
Date_            20221230
Time                19.30
INSTRUM             spect
PROBHD    5 mm DUL 13C-1
PULPROG           zgpg30
TD                  65536
SOLVENT             CDCl3
NS                    120
DS                      0
SWH             24038.461 Hz
FIDRES           0.366798 Hz
AQ              1.3631488 sec
RG                   2050
DW                 20.800 usec
DE                   6.00 usec
TE                  293.2 K
D1             2.00000000 sec
D11            0.03000000 sec
TD0                     1

======== CHANNEL f1 ========
NUC1                  13C
P1                  40.00 usec
PL1                 -3.00 dB
PL1W          60.64365387 W
SFO1          100.6228298 MHz

======== CHANNEL f2 ========
CPDPRG[2          waltz16
NUC2                   1H
PCPD2               80.00 usec
PL2                 -1.00 dB
PL12                14.39 dB
PL13                18.00 dB
PL2W          12.17476940 W
PL12W          0.35193357 W
PL13W          0.15327126 W
SFO2          400.1316005 MHz

F2 - Processing parameters
SI                  32768
SF            100.6127751 MHz
WDW                    EM
SSB                     0
LB                   1.00 Hz
GB                      0
PC                   1.40
```

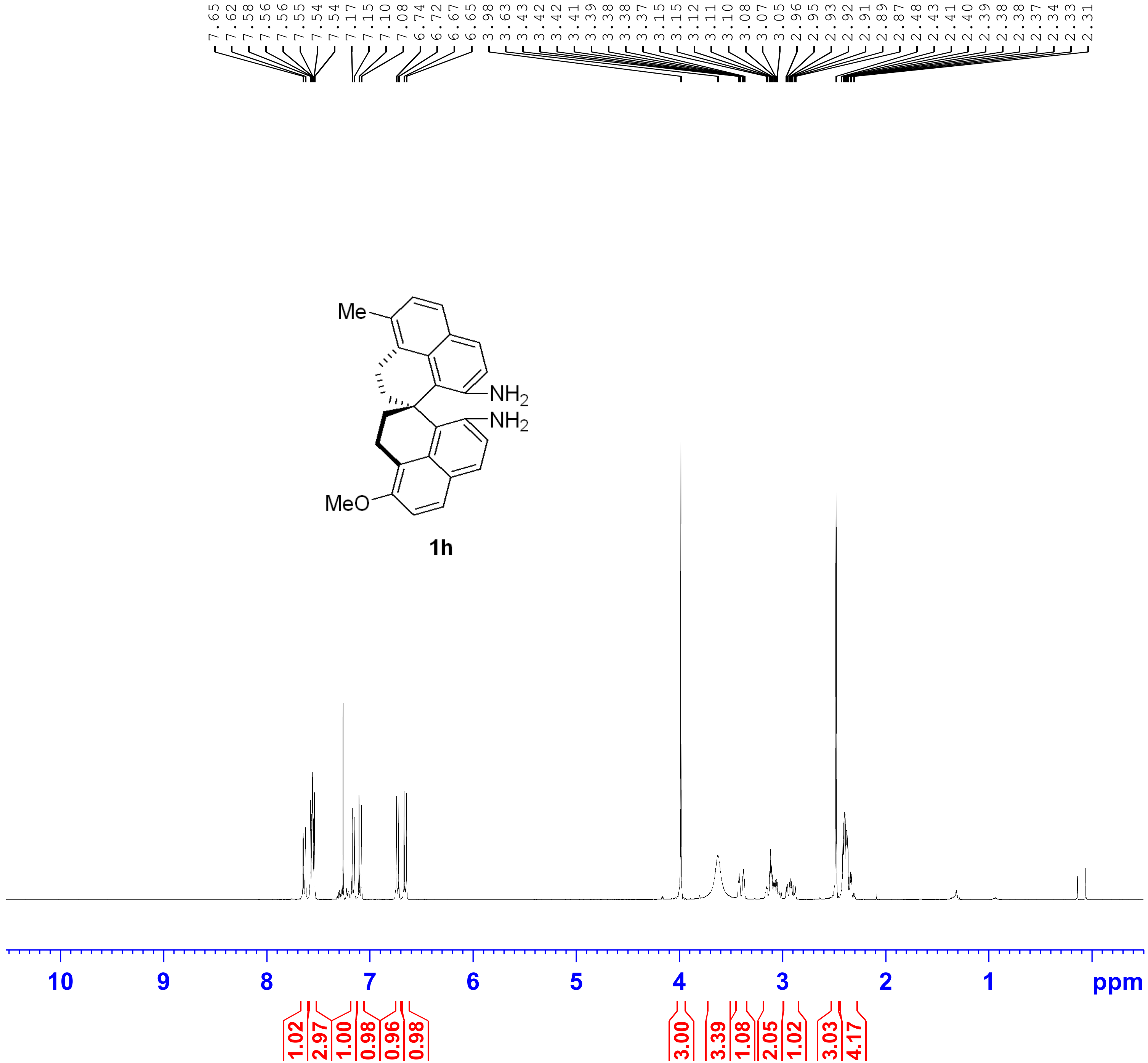

Current Data Parameters
NAME zrh-7-5-h
EXPNO 1
PROCNO 1

F2 - Acquisition Parameters
Date_ 20220702
Time 19.51
INSTRUM spect
PROBHD 5 mm DUL 13C-1
PULPROG zg30
TD 65536
SOLVENT CDCl3
NS 1
DS 0
SWH 8223.685 Hz
FIDRES 0.125483 Hz
AQ 3.9845889 sec
RG 181
DW 60.800 usec
DE 6.00 usec
TE 295.0 K
D1 1.00000000 sec
TD0 1

======== CHANNEL f1 ========
NUC1 1H
P1 15.80 usec
PL1 -1.00 dB
PL1W 12.17476940 W
SFO1 400.1324710 MHz

F2 - Processing parameters
SI 32768
SF 400.1300097 MHz
WDW EM
SSB 0
LB 0.30 Hz
GB 0
PC 1.00

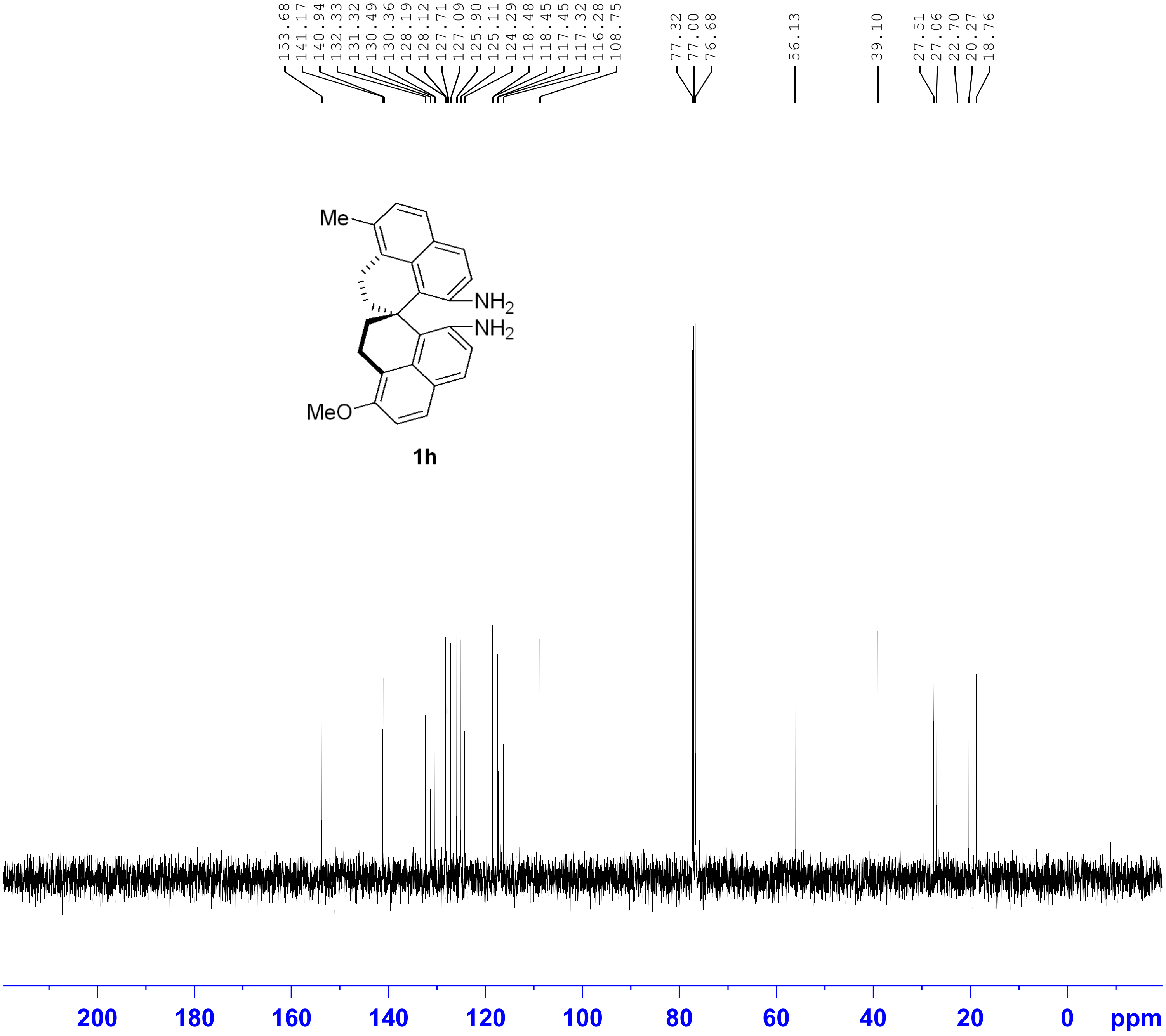

1h

Current Data Parameters
NAME zrh-7-5-c
EXPNO 1
PROCNO 1

F2 - Acquisition Parameters
Date_ 20220702
Time 19.53
INSTRUM spect
PROBHD 5 mm DUL 13C-1
PULPROG zgpg30
TD 65536
SOLVENT CDCl3
NS 32
DS 0
SWH 24038.461 Hz
FIDRES 0.366798 Hz
AQ 1.3631488 sec
RG 2050
DW 20.800 usec
DE 6.00 usec
TE 295.2 K
D1 2.00000000 sec
D11 0.03000000 sec
TD0 1

======== CHANNEL f1 ========
NUC1 13C
P1 40.00 usec
PL1 -3.00 dB
PL1W 60.64365387 W
SFO1 100.6228298 MHz

======== CHANNEL f2 ========
CPDPRG[2 waltz16
NUC2 1H
PCPD2 80.00 usec
PL2 -1.00 dB
PL12 14.39 dB
PL13 18.00 dB
PL2W 12.17476940 W
PL12W 0.35193357 W
PL13W 0.15327126 W
SFO2 400.1316005 MHz

F2 - Processing parameters
SI 32768
SF 100.6127810 MHz
WDW EM
SSB 0
LB 1.00 Hz
GB 0
PC 1.40

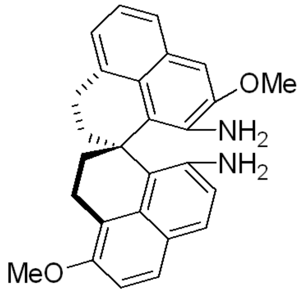

1i

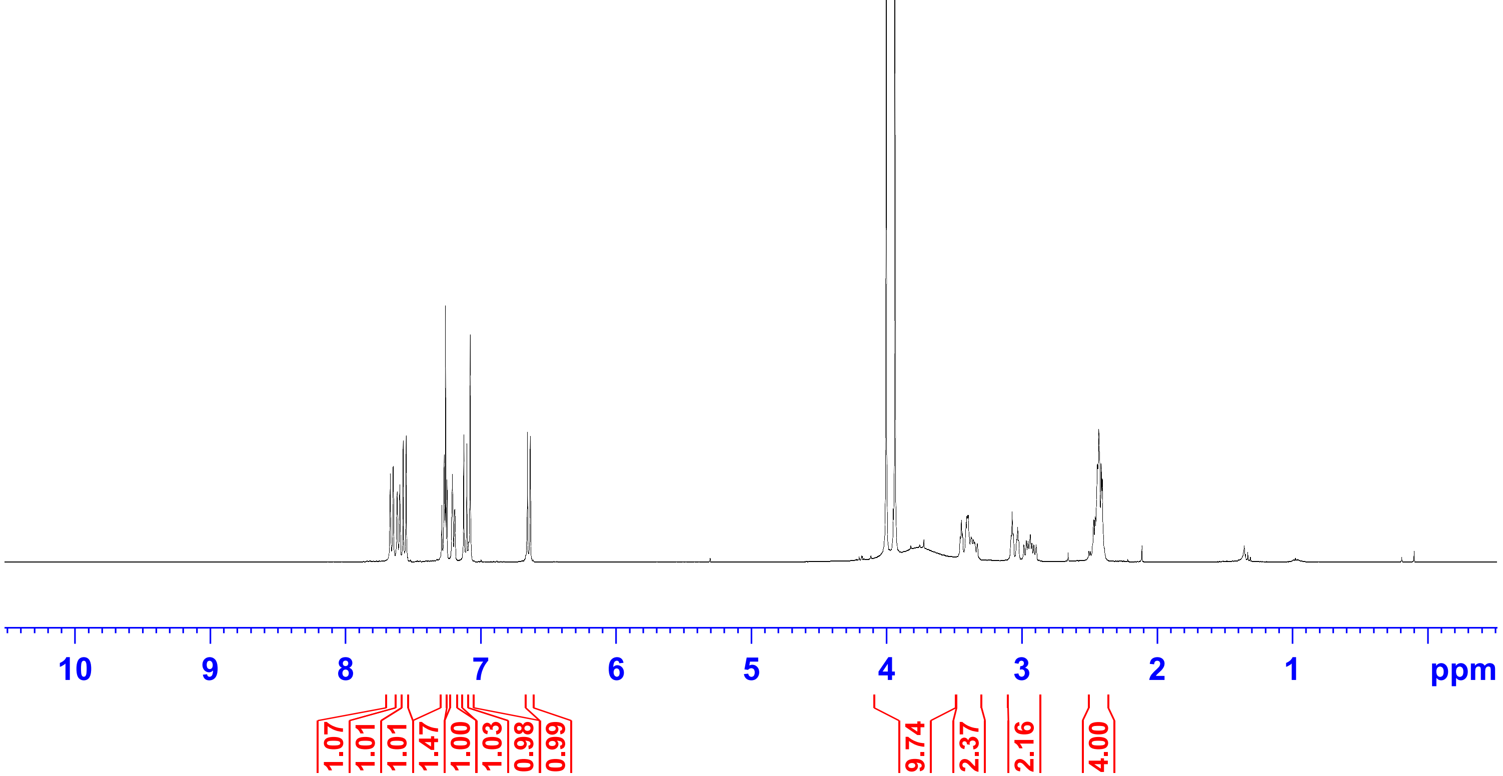


```
Current Data Parameters
NAME     zrh-6-175-large-h
EXPNO                    1
PROCNO                   1

F2 - Acquisition Parameters
Date_             20220623
Time                 22.07
INSTRUM              spect
PROBHD    5 mm PABBO BB/
PULPROG               zg30
TD                   65536
SOLVENT              CDCl3
NS                       3
DS                       0
SWH               8012.820 Hz
FIDRES            0.122266 Hz
AQ               4.0894465 sec
RG                   25.32
DW                  62.400 usec
DE                    6.50 usec
TE                   295.9 K
D1              1.00000000 sec
TD0                      1

======== CHANNEL f1 ========
SFO1           400.1324710 MHz
NUC1                    1H
P1                   14.50 usec
PLW1           11.99499989 W

F2 - Processing parameters
SI                   65536
SF             400.1300099 MHz
WDW                     EM
SSB                      0
LB                    0.30 Hz
GB                       0
PC                    1.00
```

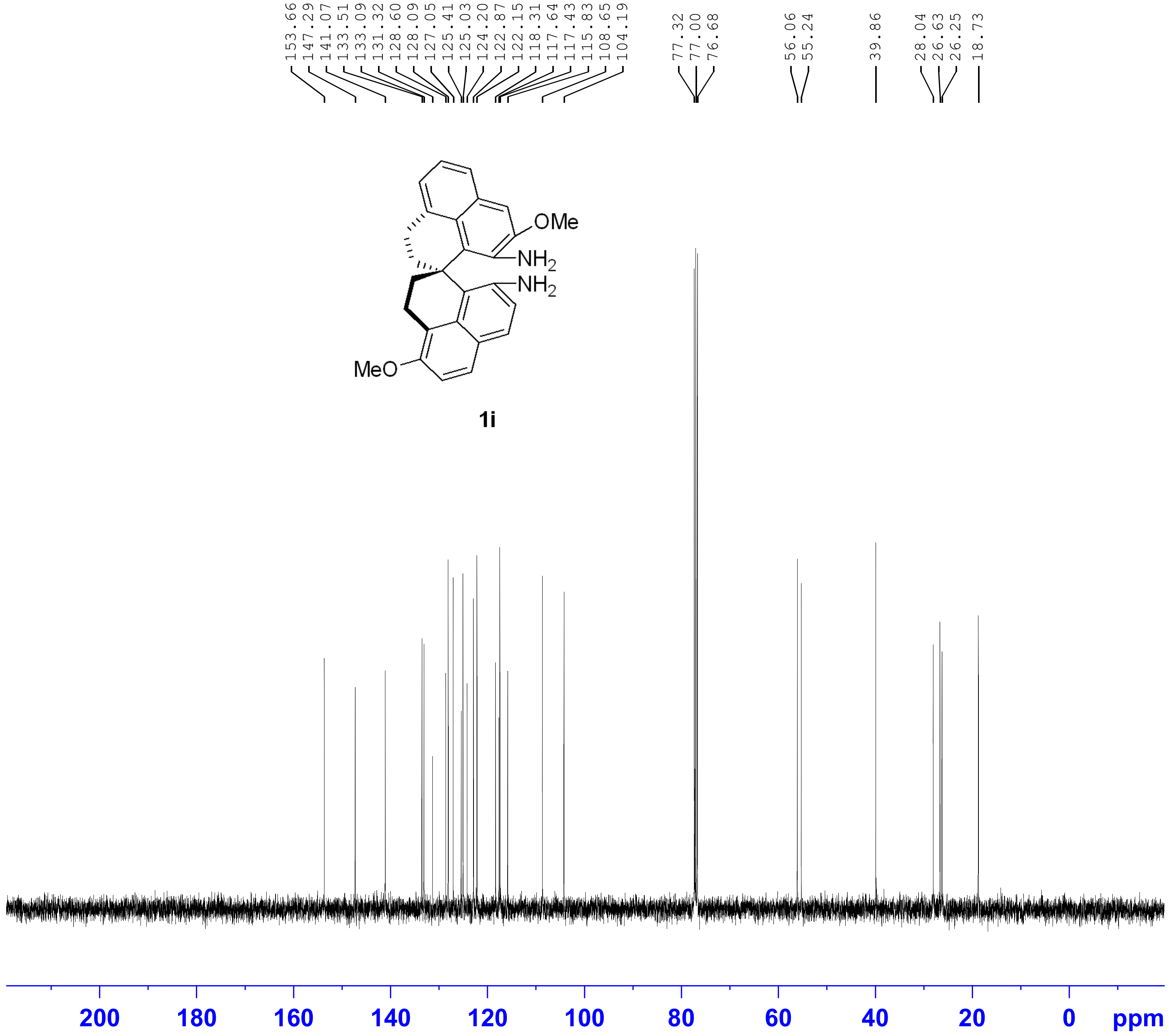

Current Data Parameters
NAME zrh-6-175-large-c
EXPNO 1
PROCNO 1

F2 - Acquisition Parameters
Date_ 20220623
Time 22.10
INSTRUM spect
PROBHD 5 mm PABBO BB/
PULPROG zgpg30
TD 65536
SOLVENT CDCl3
NS 22
DS 0
SWH 24038.461 Hz
FIDRES 0.366798 Hz
AQ 1.3631488 sec
RG 196.92
DW 20.800 usec
DE 6.50 usec
TE 296.3 K
D1 2.00000000 sec
D11 0.03000000 sec
TD0 1

======== CHANNEL f1 ========
SFO1 100.6228298 MHz
NUC1 13C
P1 9.70 usec
PLW1 46.98899841 W

======== CHANNEL f2 ========
SFO2 400.1316005 MHz
NUC2 1H
CPDPRG[2 waltz16
PCPD2 90.00 usec
PLW2 11.99499989 W
PLW12 0.34213999 W
PLW13 0.27713001 W

F2 - Processing parameters
SI 32768
SF 100.6127890 MHz
WDW EM
SSB 0
LB 1.00 Hz
GB 0
PC 1.40

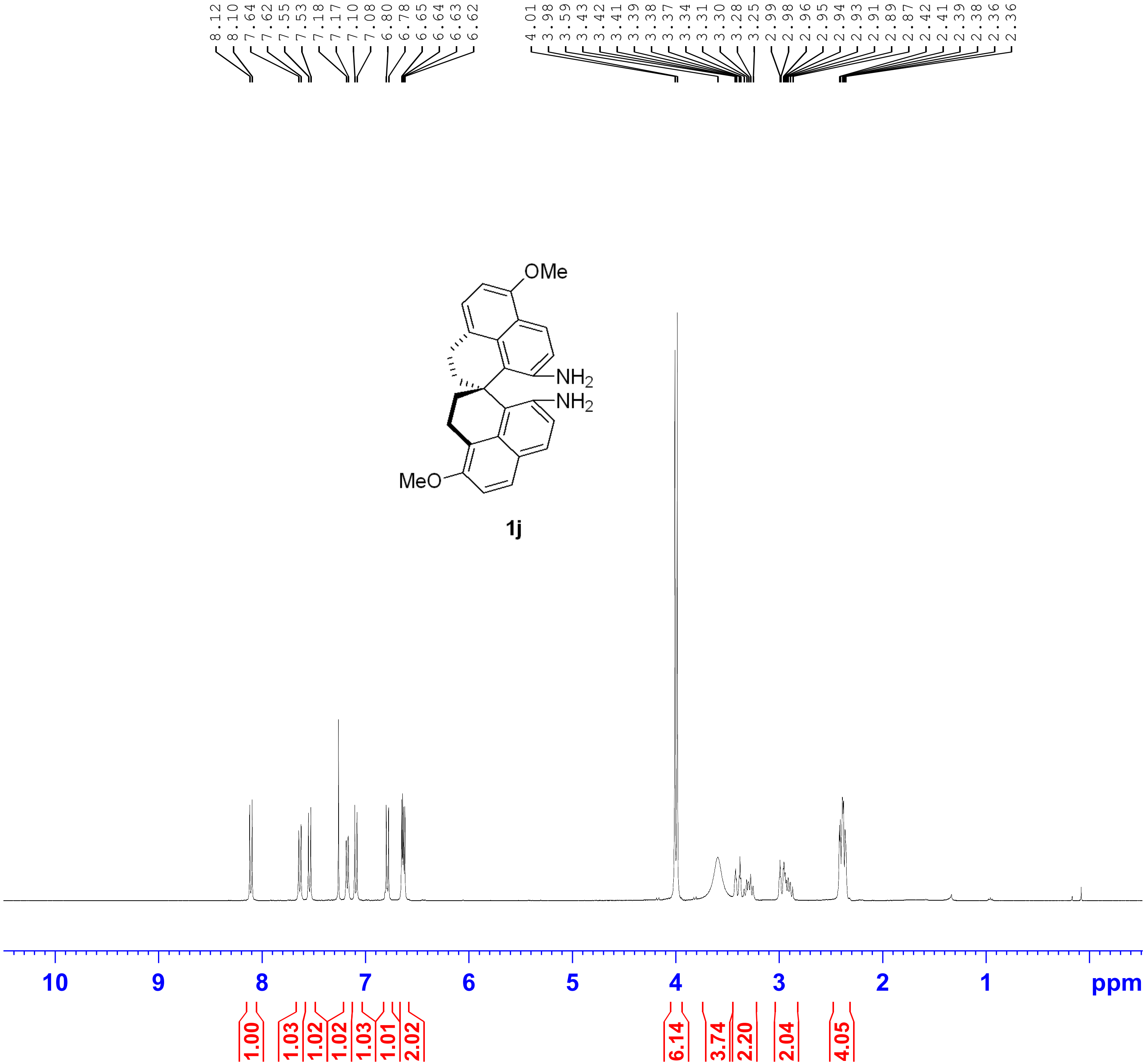

Current Data Parameters
NAME zrh-7-60-h
EXPNO 1
PROCNO 1

F2 - Acquisition Parameters
Date_ 20220730
Time 20.00
INSTRUM spect
PROBHD 5 mm DUL 13C-1
PULPROG zg30
TD 65536
SOLVENT CDCl3
NS 1
DS 0
SWH 8223.685 Hz
FIDRES 0.125483 Hz
AQ 3.9845889 sec
RG 128
DW 60.800 usec
DE 6.00 usec
TE 295.0 K
D1 1.00000000 sec
TD0 1

======== CHANNEL f1 ========
NUC1 1H
P1 15.80 usec
PL1 -1.00 dB
PL1W 12.17476940 W
SFO1 400.1324710 MHz

F2 - Processing parameters
SI 32768
SF 400.1300096 MHz
WDW EM
SSB 0
LB 0.30 Hz
GB 0
PC 1.00

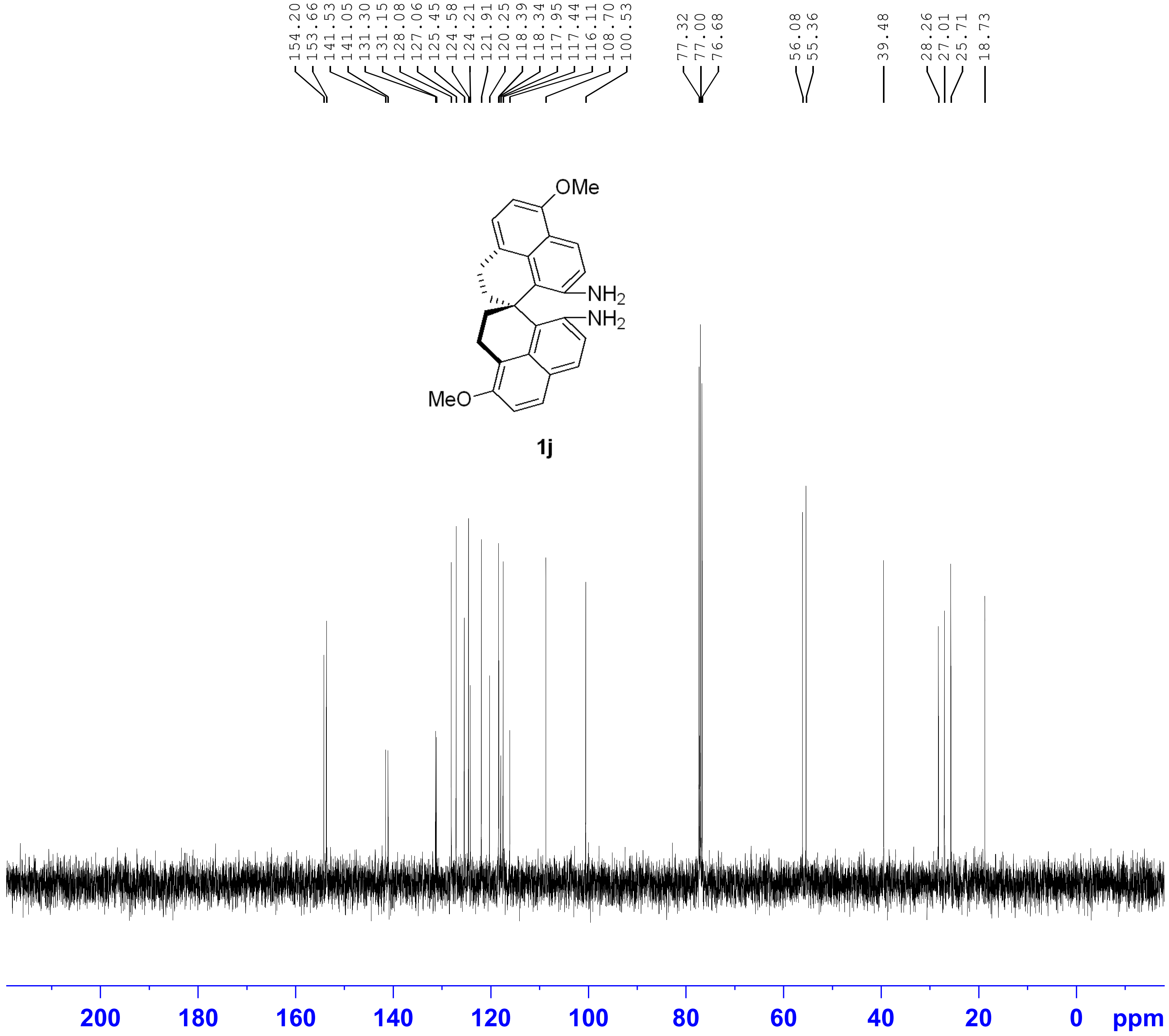

1j

Current Data Parameters

NAME	zrh-7-60-c
EXPNO	1
PROCNO	1

F2 - Acquisition Parameters

Date_	20220730
Time	20.01
INSTRUM	spect
PROBHD	5 mm DUL 13C-1
PULPROG	zgpg30
TD	65536
SOLVENT	CDCl3
NS	27
DS	0
SWH	24038.461 Hz
FIDRES	0.366798 Hz
AQ	1.3631488 sec
RG	2050
DW	20.800 usec
DE	6.00 usec
TE	295.2 K
D1	2.00000000 sec
D11	0.03000000 sec
TD0	1

======== CHANNEL f1 ========

NUC1	13C
P1	40.00 usec
PL1	-3.00 dB
PL1W	60.64365387 W
SFO1	100.6228298 MHz

======== CHANNEL f2 ========

CPDPRG[2	waltz16
NUC2	1H
PCPD2	80.00 usec
PL2	-1.00 dB
PL12	14.39 dB
PL13	18.00 dB
PL2W	12.17476940 W
PL12W	0.35193357 W
PL13W	0.15327126 W
SFO2	400.1316005 MHz

F2 - Processing parameters

SI	32768
SF	100.6127854 MHz
WDW	EM
SSB	0
LB	1.00 Hz
GB	0
PC	1.40

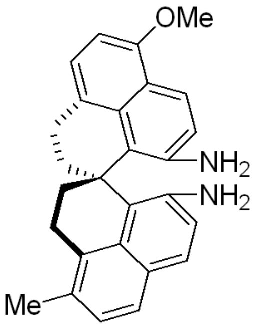

1k

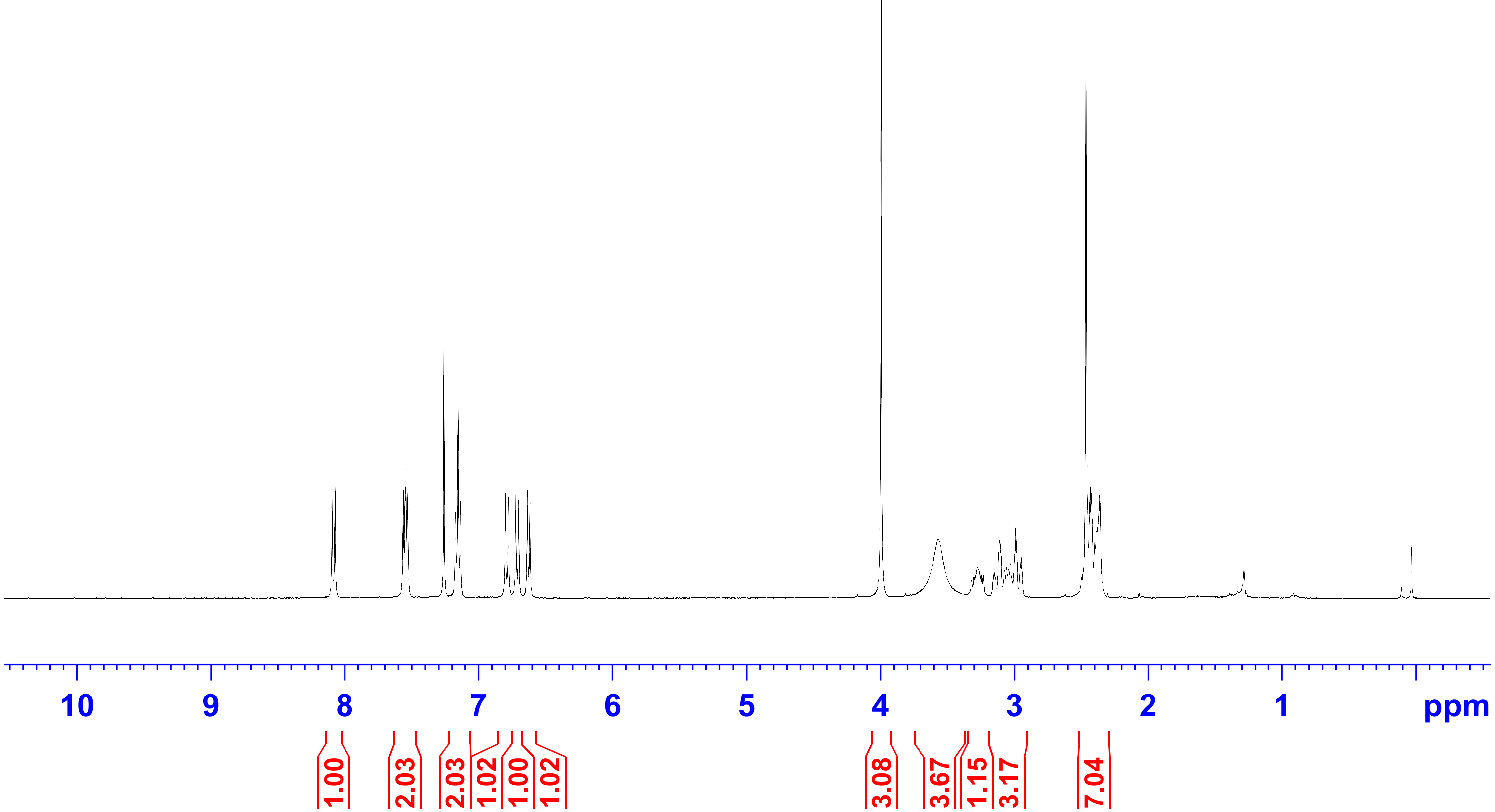


```
Current Data Parameters
NAME          zrh-8-106-h
EXPNO                   1
PROCNO                  1

F2 - Acquisition Parameters
Date_            20221230
Time                19.09
INSTRUM             spect
PROBHD   5 mm DUL 13C-1
PULPROG              zg30
TD                  65536
SOLVENT             CDCl3
NS                      3
DS                      0
SWH              8223.685 Hz
FIDRES           0.125483 Hz
AQ              3.9845889 sec
RG                    228
DW                 60.800 usec
DE                   6.00 usec
TE                  293.1 K
D1             1.00000000 sec
TD0                     1

======== CHANNEL f1 ========
NUC1                   1H
P1                  15.80 usec
PL1                 -1.00 dB
PL1W          12.17476940 W
SFO1          400.1324710 MHz

F2 - Processing parameters
SI                  32768
SF            400.1300097 MHz
WDW                    EM
SSB                     0
LB                   0.30 Hz
GB                      0
PC                   1.00
```

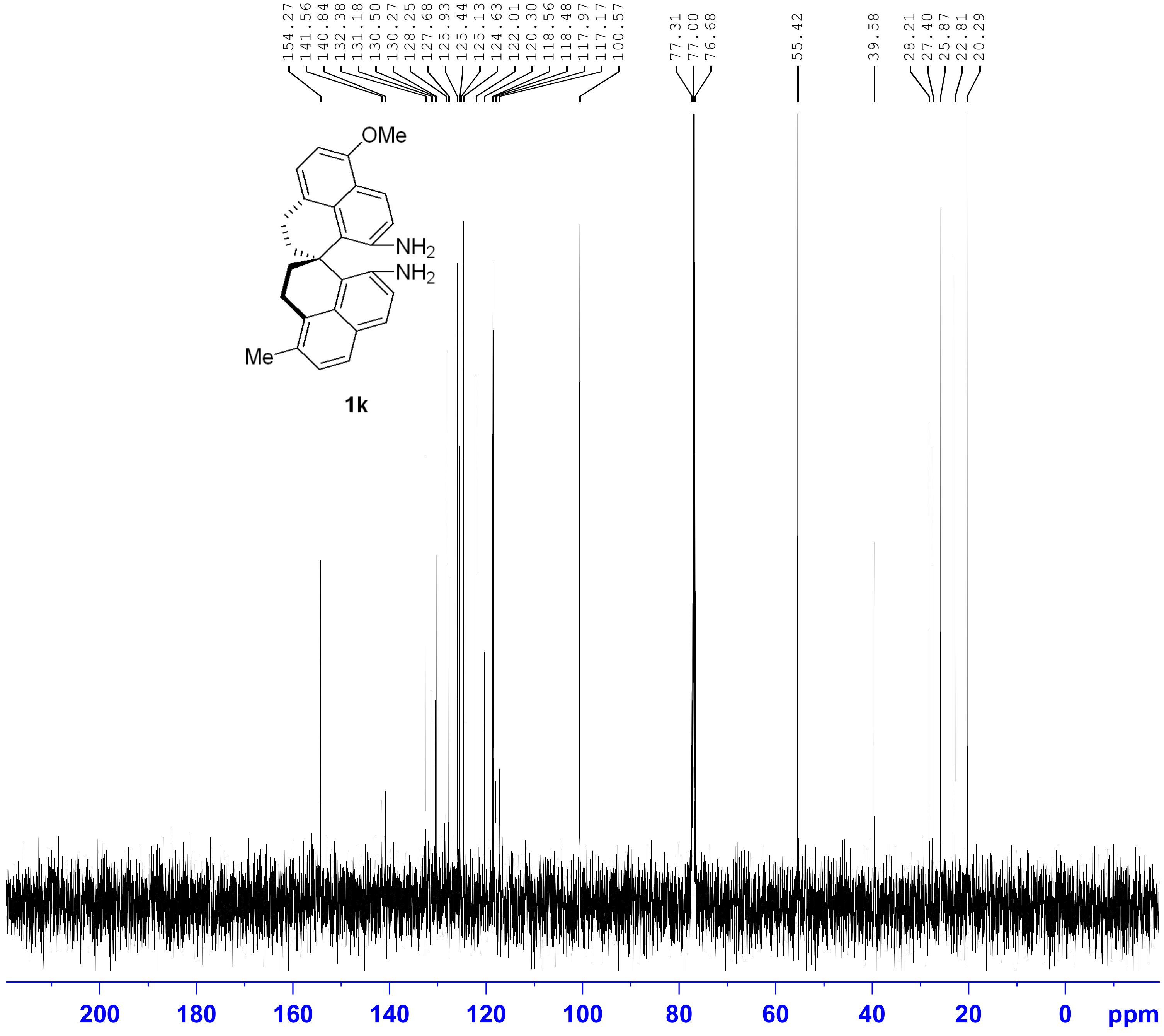


```
Current Data Parameters
NAME          zrh-8-106-c
EXPNO                   1
PROCNO                  1

F2 - Acquisition Parameters
Date_            20221230
Time                19.11
INSTRUM             spect
PROBHD   5 mm DUL 13C-1
PULPROG           zgpg30
TD                 65536
SOLVENT            CDCl3
NS                   257
DS                     0
SWH            24038.461 Hz
FIDRES          0.366798 Hz
AQ             1.3631488 sec
RG                  2050
DW                20.800 usec
DE                  6.00 usec
TE                 293.3 K
D1            2.00000000 sec
D11           0.03000000 sec
TD0                    1

======== CHANNEL f1 ========
NUC1                 13C
P1                 40.00 usec
PL1                -3.00 dB
PL1W         60.64365387 W
SFO1         100.6228298 MHz

======== CHANNEL f2 ========
CPDPRG[2         waltz16
NUC2                  1H
PCPD2              80.00 usec
PL2                -1.00 dB
PL12               14.39 dB
PL13               18.00 dB
PL2W         12.17476940 W
PL12W         0.35193357 W
PL13W         0.15327126 W
SFO2         400.1316005 MHz

F2 - Processing parameters
SI                 32768
SF           100.6127773 MHz
WDW                   EM
SSB                    0
LB                  1.00 Hz
GB                     0
PC                  1.40
```

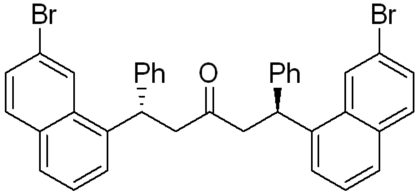

7a

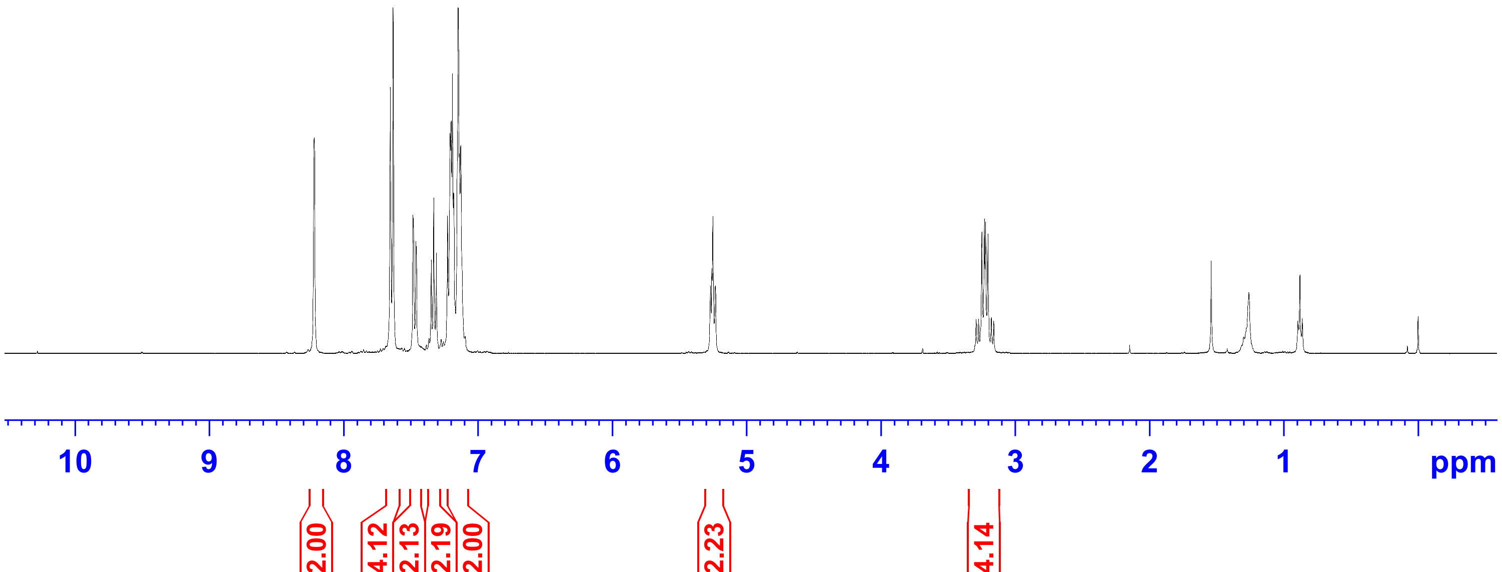


```
Current Data Parameters
NAME           zrh-9-35-h
EXPNO                   1
PROCNO                  1

F2 - Acquisition Parameters
Date_            20230327
Time                20.36
INSTRUM             spect
PROBHD     5 mm DUL 13C-1
PULPROG              zg30
TD                  65536
SOLVENT             CDCl3
NS                      3
DS                      0
SWH              8223.685 Hz
FIDRES           0.125483 Hz
AQ              3.9845889 sec
RG                    203
DW                 60.800 usec
DE                   6.00 usec
TE                  292.7 K
D1             1.00000000 sec
TD0                     1

======== CHANNEL f1 ========
NUC1                   1H
P1                  15.80 usec
PL1                 -1.00 dB
PL1W          12.17476940 W
SFO1          400.1324710 MHz

F2 - Processing parameters
SI                  32768
SF            400.1300231 MHz
WDW                    EM
SSB                     0
LB                   0.30 Hz
GB                      0
PC                   1.00
```

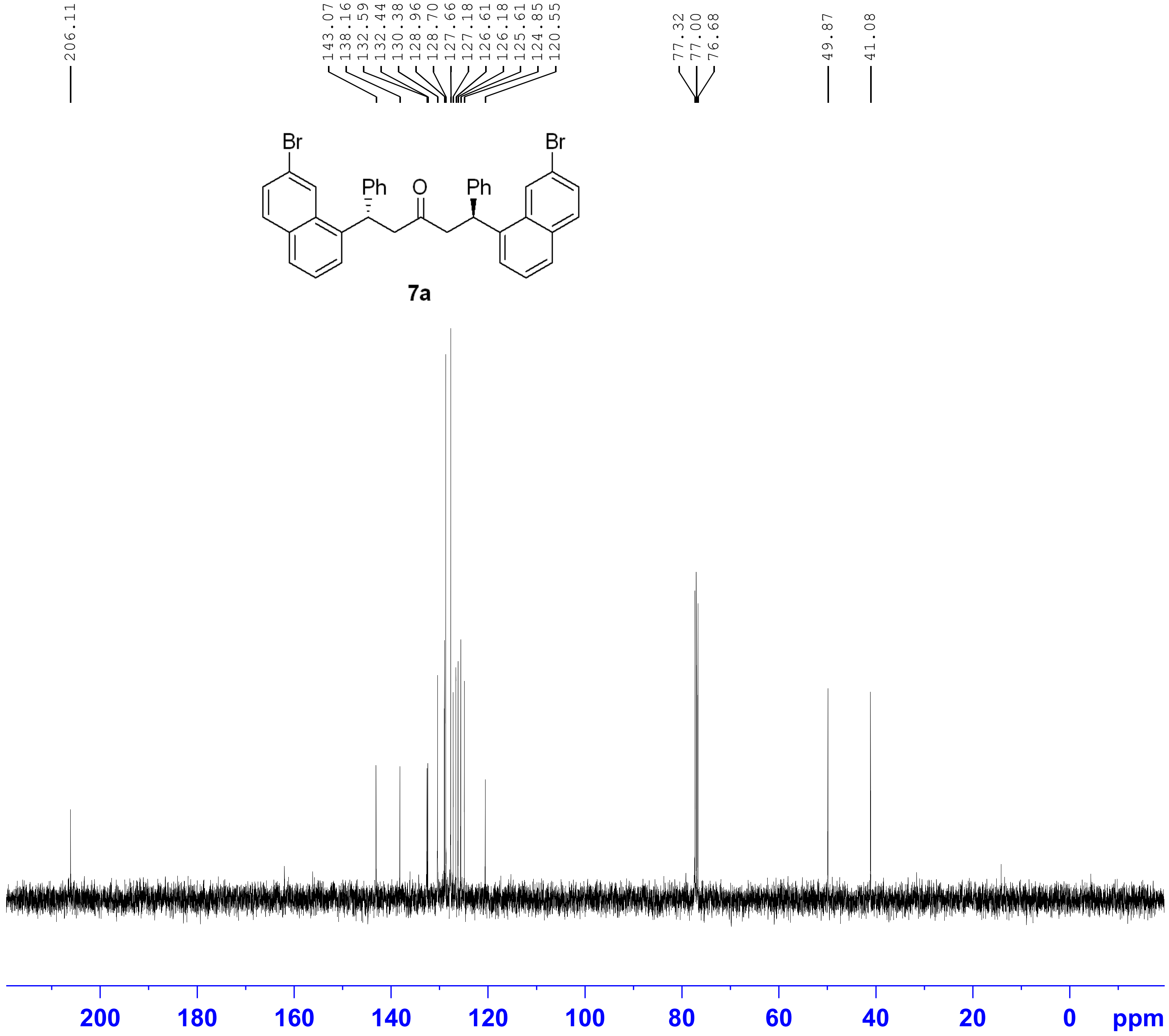
206.11
143.07
138.16
132.59
132.44
130.38
128.96
128.70
127.66
127.18
126.61
126.18
125.61
124.85
120.55
77.32
77.00
76.68
49.87
41.08
Br
Br
Ph
O
Ph
7a
200
180
160
140
120
100
80
60
40
20
0
ppm

Current Data Parameters
NAME zrh-9-35-c
EXPNO 1
PROCNO 1

F2 - Acquisition Parameters
Date_ 20230327
Time 20.37
INSTRUM spect
PROBHD 5 mm DUL 13C-1
PULPROG zgpg30
TD 65536
SOLVENT CDCl3
NS 15
DS 0
SWH 24038.461 Hz
FIDRES 0.366798 Hz
AQ 1.3631488 sec
RG 2050
DW 20.800 usec
DE 6.00 usec
TE 292.7 K
D1 2.00000000 sec
D11 0.03000000 sec
TD0 1

======== CHANNEL f1 ========
NUC1 13C
P1 40.00 usec
PL1 -3.00 dB
PL1W 60.64365387 W
SFO1 100.6228298 MHz

======== CHANNEL f2 ========
CPDPRG[2 waltz16
NUC2 1H
PCPD2 80.00 usec
PL2 -1.00 dB
PL12 14.39 dB
PL13 18.00 dB
PL2W 12.17476940 W
PL12W 0.35193357 W
PL13W 0.15327126 W
SFO2 400.1316005 MHz

F2 - Processing parameters
SI 32768
SF 100.6127780 MHz
WDW EM
SSB 0
LB 1.00 Hz
GB 0
PC 1.40

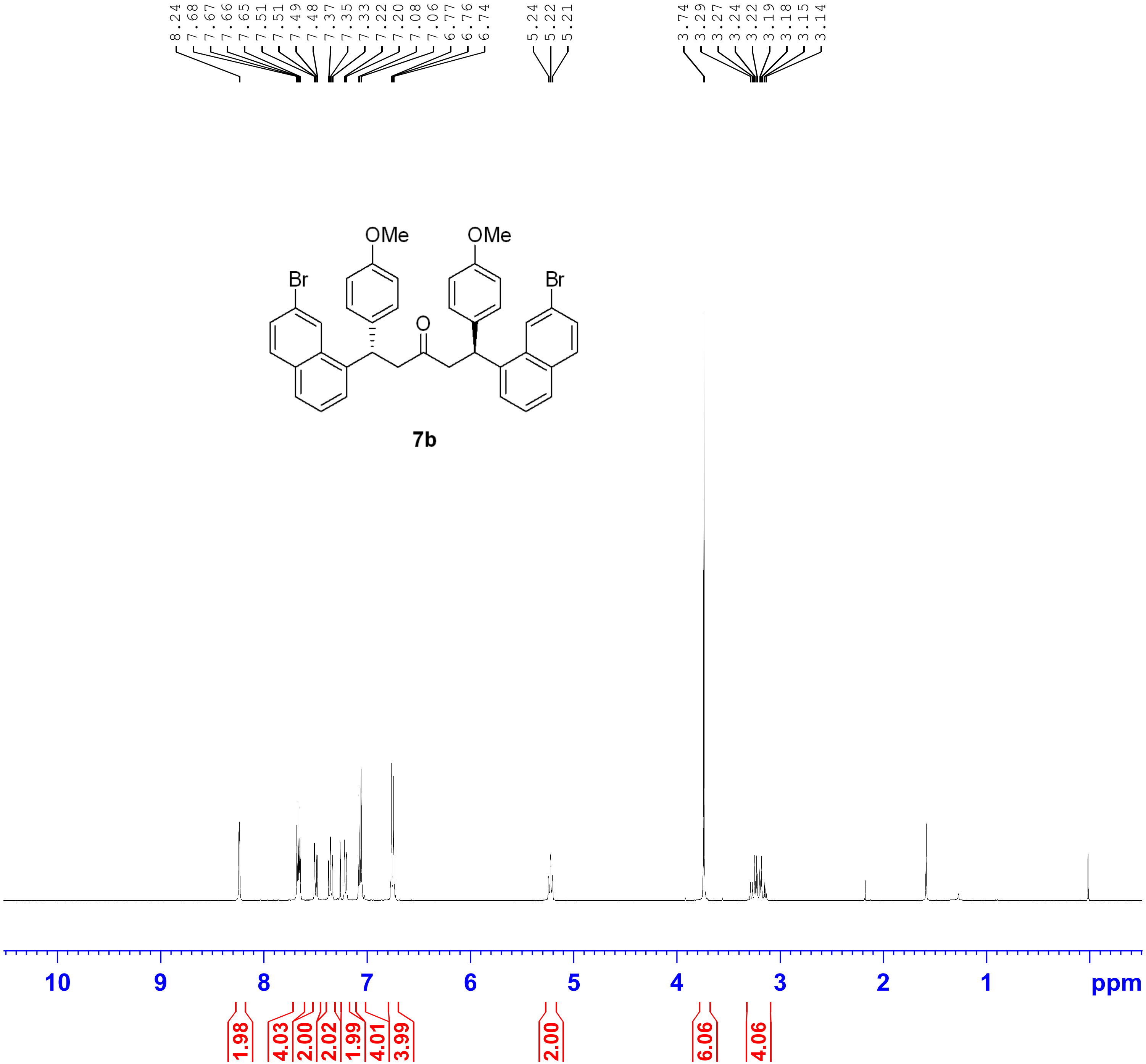

7b

```
Current Data Parameters
NAME      zrh-9-37-1-re-h
EXPNO                   1
PROCNO                  1

F2 - Acquisition Parameters
Date_            20230429
Time                14.49
INSTRUM             spect
PROBHD    5 mm PABBO BB/
PULPROG              zg30
TD                  65536
SOLVENT             CDCl3
NS                      2
DS                      2
SWH              8012.820 Hz
FIDRES           0.122266 Hz
AQ              4.0894465 sec
RG                  82.92
DW                 62.400 usec
DE                   6.50 usec
TE                  296.1 K
D1             1.00000000 sec
TD0                     1

======== CHANNEL f1 ========
SFO1          400.1324710 MHz
NUC1                   1H
P1                  14.50 usec
PLW1          11.99499989 W

F2 - Processing parameters
SI                  65536
SF            400.1300100 MHz
WDW                    EM
SSB                     0
LB                   0.30 Hz
GB                      0
PC                   1.00
```

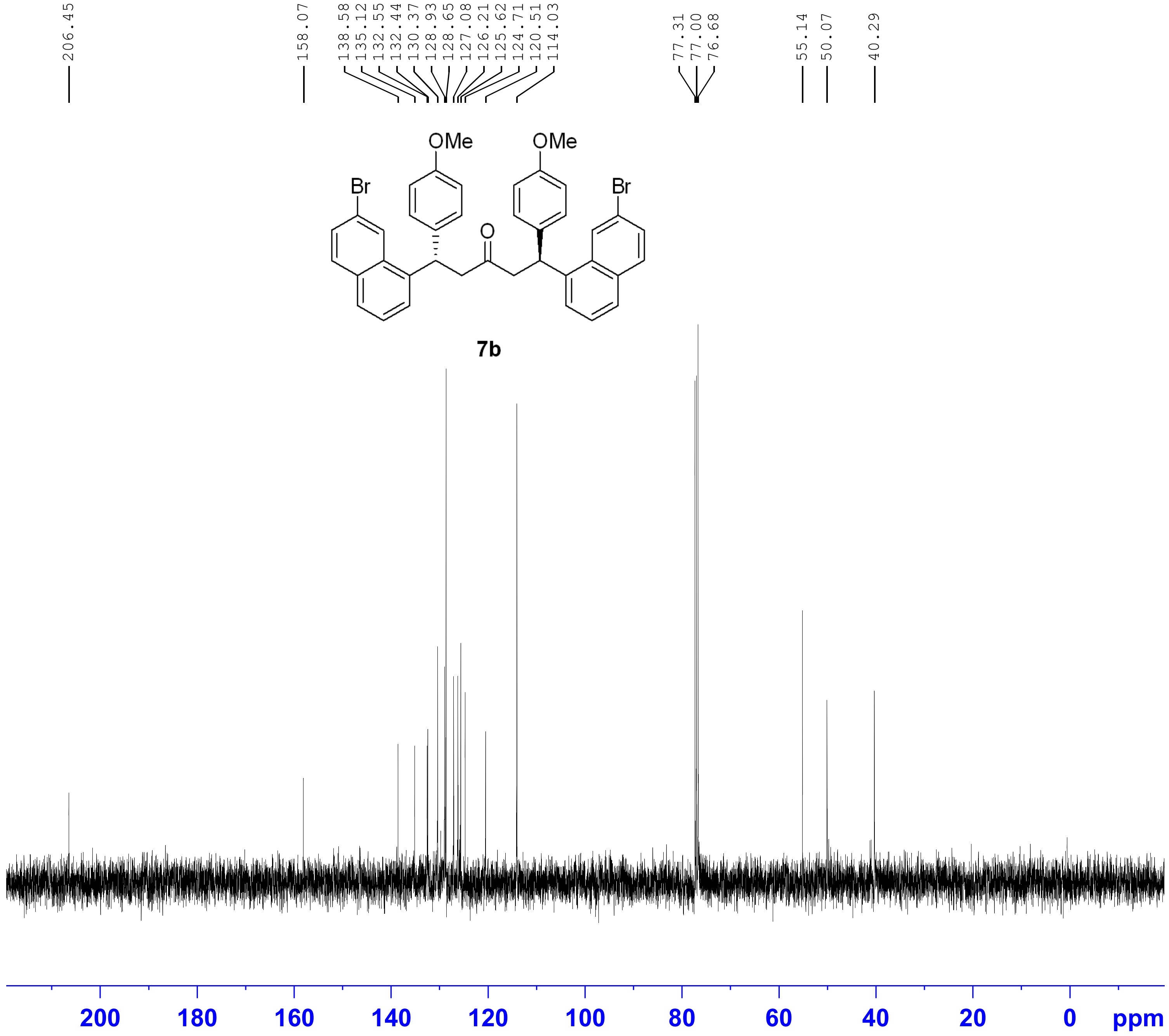

7b

```
Current Data Parameters
NAME          zrh-9-37-1-c
EXPNO                    1
PROCNO                   1

F2 - Acquisition Parameters
Date_             20230327
Time                 20.42
INSTRUM              spect
PROBHD    5 mm DUL 13C-1
PULPROG            zgpg30
TD                  65536
SOLVENT             CDCl3
NS                     21
DS                      0
SWH             24038.461 Hz
FIDRES           0.366798 Hz
AQ              1.3631488 sec
RG                   2050
DW                 20.800 usec
DE                   6.00 usec
TE                  292.8 K
D1             2.00000000 sec
D11            0.03000000 sec
TD0                     1

======== CHANNEL f1 ========
NUC1                  13C
P1                  40.00 usec
PL1                 -3.00 dB
PL1W          60.64365387 W
SFO1          100.6228298 MHz

======== CHANNEL f2 ========
CPDPRG[2          waltz16
NUC2                   1H
PCPD2               80.00 usec
PL2                 -1.00 dB
PL12                14.39 dB
PL13                18.00 dB
PL2W          12.17476940 W
PL12W          0.35193357 W
PL13W          0.15327126 W
SFO2          400.1316005 MHz

F2 - Processing parameters
SI                  32768
SF            100.6127758 MHz
WDW                    EM
SSB                     0
LB                   1.00 Hz
GB                      0
PC                   1.40
```

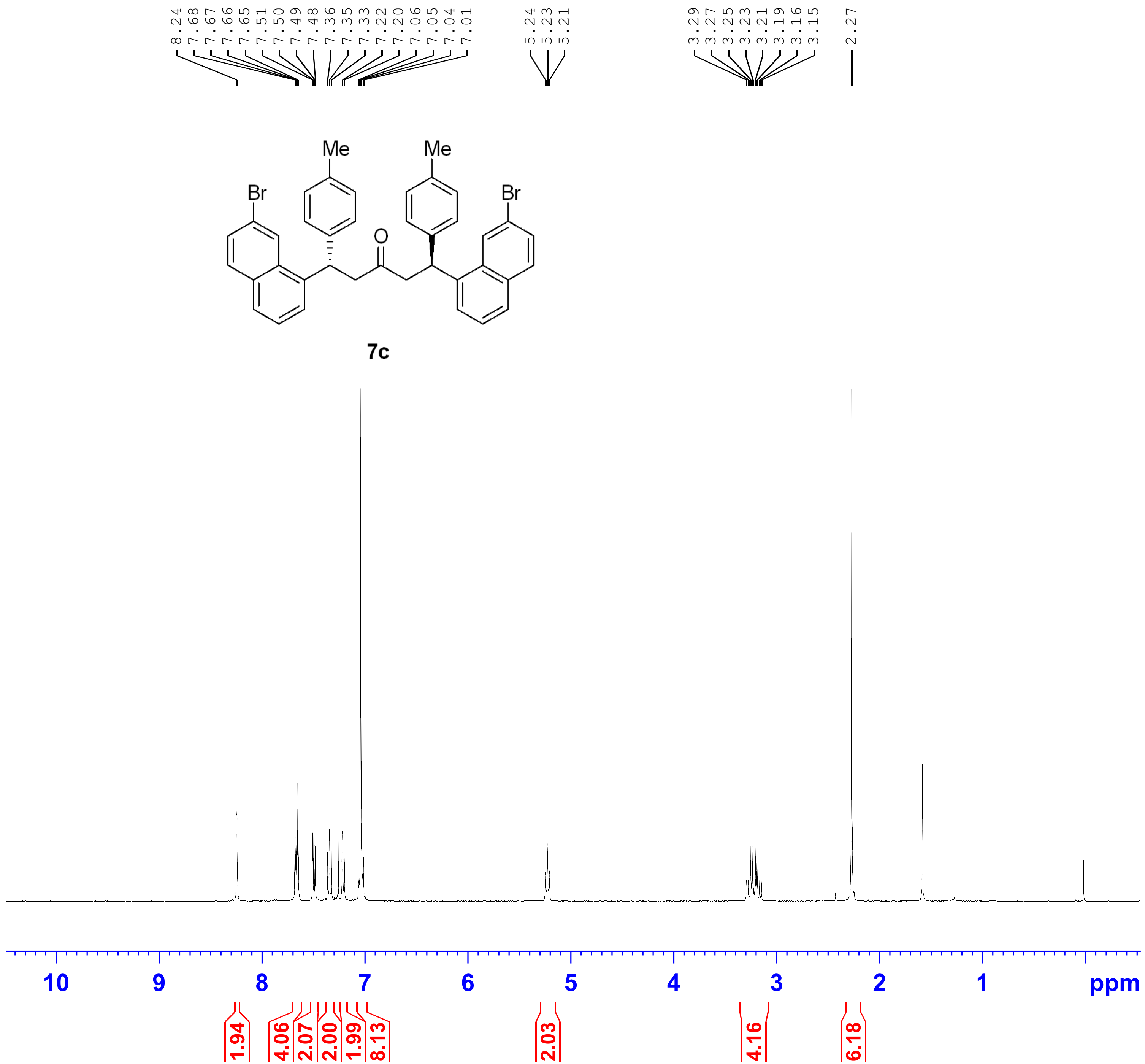


```
Current Data Parameters
NAME          zrh-9-48-1-h
EXPNO                    1
PROCNO                   1

F2 - Acquisition Parameters
Date_             20230403
Time                 19.11
INSTRUM              spect
PROBHD     5 mm DUL 13C-1
PULPROG               zg30
TD                   65536
SOLVENT              CDCl3
NS                       2
DS                       0
SWH               8223.685 Hz
FIDRES            0.125483 Hz
AQ               3.9845889 sec
RG                     322
DW                  60.800 usec
DE                    6.00 usec
TE                   292.8 K
D1              1.00000000 sec
TD0                      1

======== CHANNEL f1 ========
NUC1                    1H
P1                   15.80 usec
PL1                  -1.00 dB
PL1W           12.17476940 W
SFO1           400.1324710 MHz

F2 - Processing parameters
SI                   32768
SF             400.1300098 MHz
WDW                     EM
SSB                      0
LB                    0.30 Hz
GB                       0
PC                    1.00
```

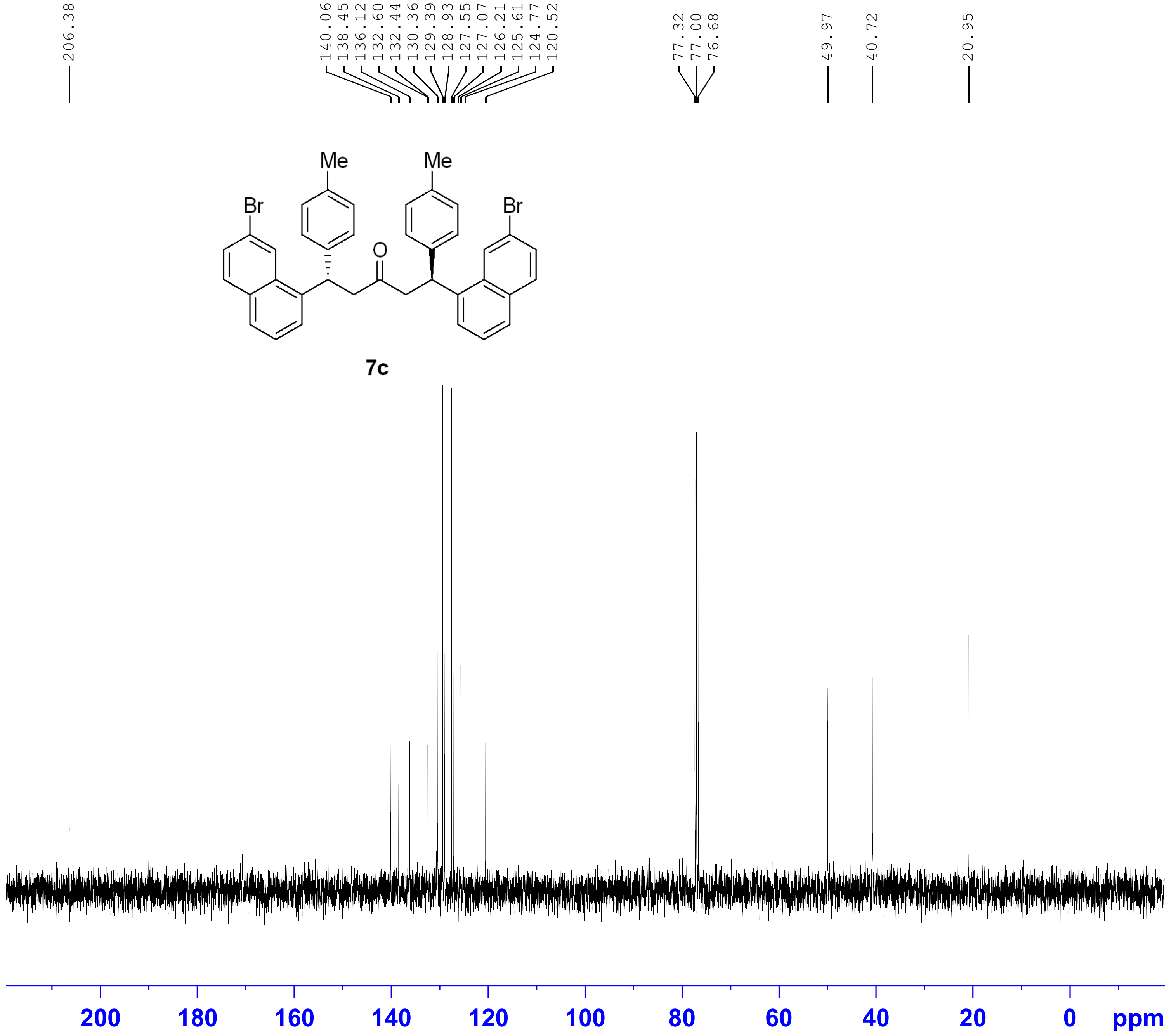


```
Current Data Parameters
NAME          zrh-9-48-1-c
EXPNO                    1
PROCNO                   1

F2 - Acquisition Parameters
Date_             20230403
Time                 19.09
INSTRUM              spect
PROBHD   5 mm DUL 13C-1
PULPROG            zgpg30
TD                   65536
SOLVENT              CDCl3
NS                      27
DS                       0
SWH              24038.461 Hz
FIDRES            0.366798 Hz
AQ               1.3631488 sec
RG                    2050
DW                  20.800 usec
DE                    6.00 usec
TE                   292.8 K
D1              2.00000000 sec
D11             0.03000000 sec
TD0                      1

======== CHANNEL f1 ========
NUC1                   13C
P1                   40.00 usec
PL1                  -3.00 dB
PL1W           60.64365387 W
SFO1          100.6228298 MHz

======== CHANNEL f2 ========
CPDPRG[2           waltz16
NUC2                    1H
PCPD2                80.00 usec
PL2                  -1.00 dB
PL12                 14.39 dB
PL13                 18.00 dB
PL2W           12.17476940 W
PL12W           0.35193357 W
PL13W           0.15327126 W
SFO2          400.1316005 MHz

F2 - Processing parameters
SI                   32768
SF            100.6127751 MHz
WDW                     EM
SSB                      0
LB                    1.00 Hz
GB                       0
PC                    1.40
```

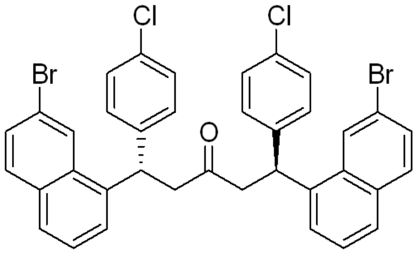

7d

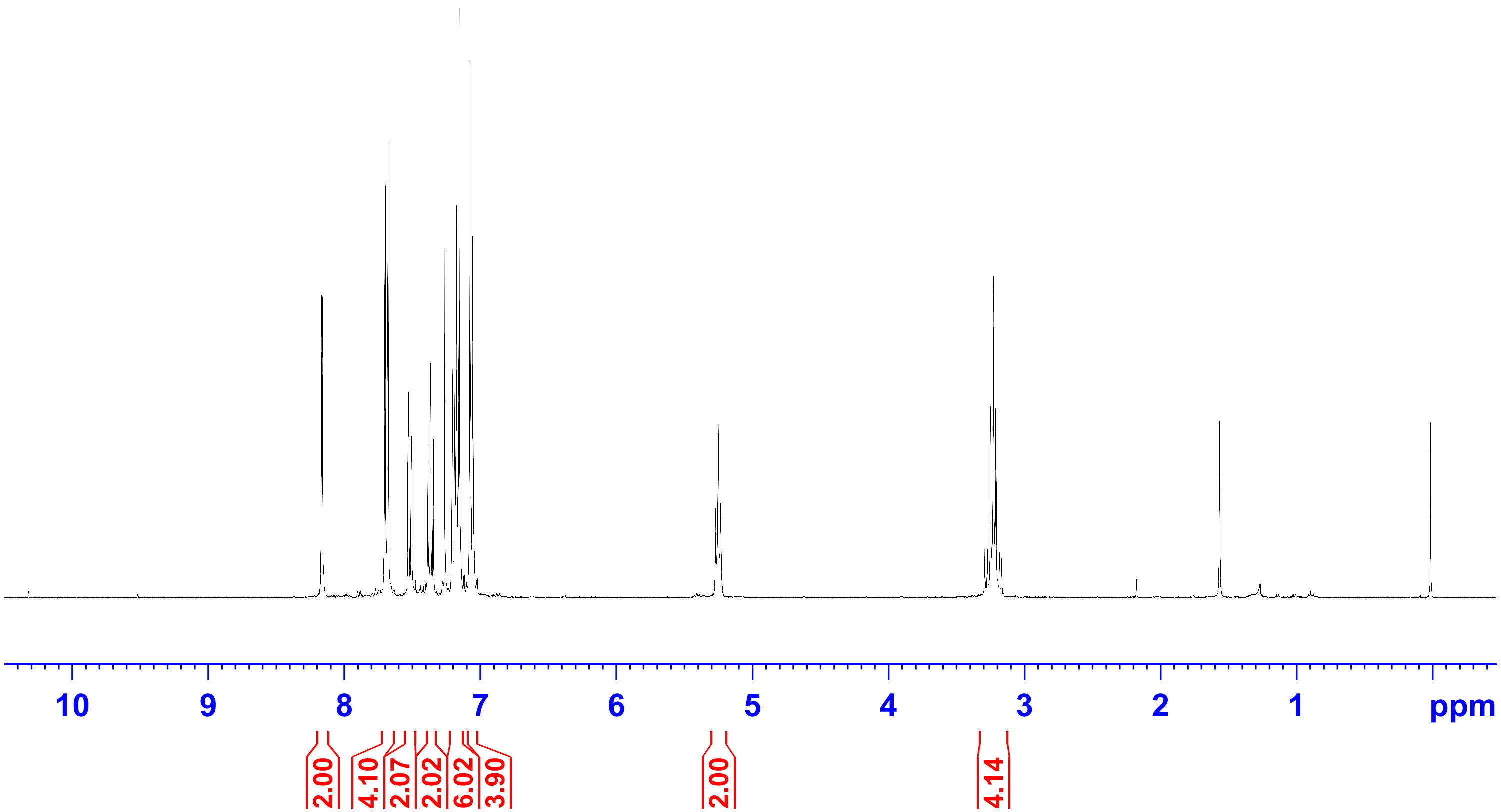

Current Data Parameters
NAME zrh-9-75-2-h
EXPNO 1
PROCNO 1

F2 - Acquisition Parameters
Date_ 20230425
Time 21.07
INSTRUM spect
PROBHD 5 mm PABBO BB/
PULPROG zg30
TD 65536
SOLVENT CDCl3
NS 3
DS 2
SWH 8012.820 Hz
FIDRES 0.122266 Hz
AQ 4.0894465 sec
RG 82.92
DW 62.400 usec
DE 6.50 usec
TE 296.5 K
D1 1.00000000 sec
TD0 1

======== CHANNEL f1 ========
SFO1 400.1324710 MHz
NUC1 1H
P1 14.50 usec
PLW1 11.99499989 W

F2 - Processing parameters
SI 65536
SF 400.1300100 MHz
WDW EM
SSB 0
LB 0.30 Hz
GB 0
PC 1.00

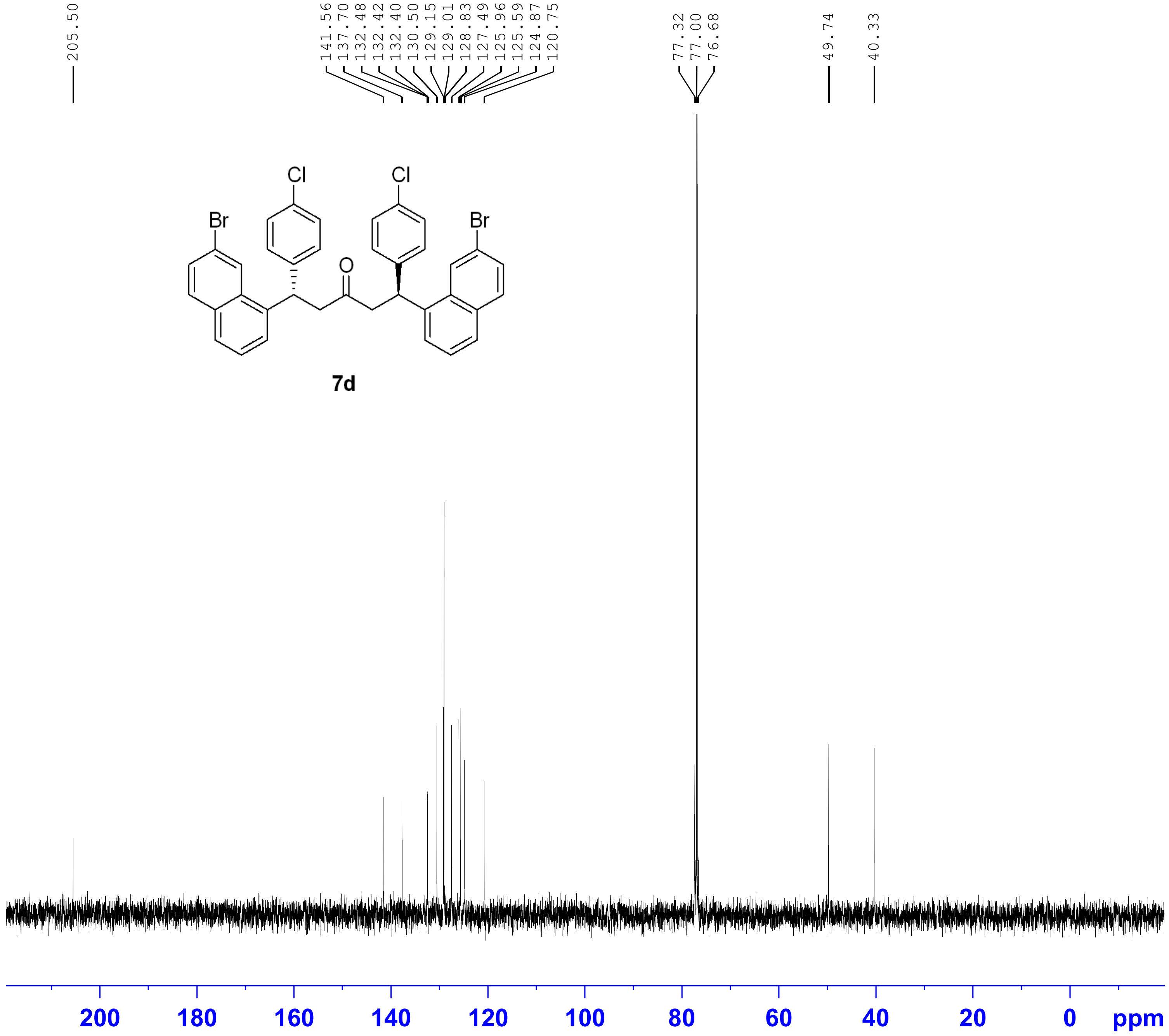

Current Data Parameters
NAME zrh-9-75-2-c
EXPNO 1
PROCNO 1

F2 - Acquisition Parameters
Date_ 20230425
Time 21.10
INSTRUM spect
PROBHD 5 mm PABBO BB/
PULPROG zgpg30
TD 65536
SOLVENT CDCl3
NS 36
DS 2
SWH 24038.461 Hz
FIDRES 0.366798 Hz
AQ 1.3631488 sec
RG 196.92
DW 20.800 usec
DE 6.50 usec
TE 297.2 K
D1 2.00000000 sec
D11 0.03000000 sec
TD0 1

======== CHANNEL f1 ========
SFO1 100.6228298 MHz
NUC1 13C
P1 9.70 usec
PLW1 46.98899841 W

======== CHANNEL f2 ========
SFO2 400.1316005 MHz
NUC2 1H
CPDPRG[2 waltz16
PCPD2 90.00 usec
PLW2 11.99499989 W
PLW12 0.34213999 W
PLW13 0.27713001 W

F2 - Processing parameters
SI 32768
SF 100.6127740 MHz
WDW EM
SSB 0
LB 1.00 Hz
GB 0
PC 1.40

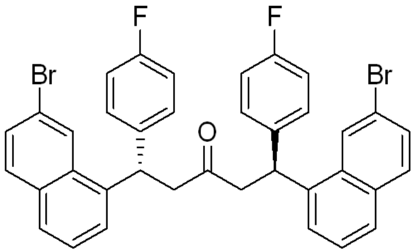

7e

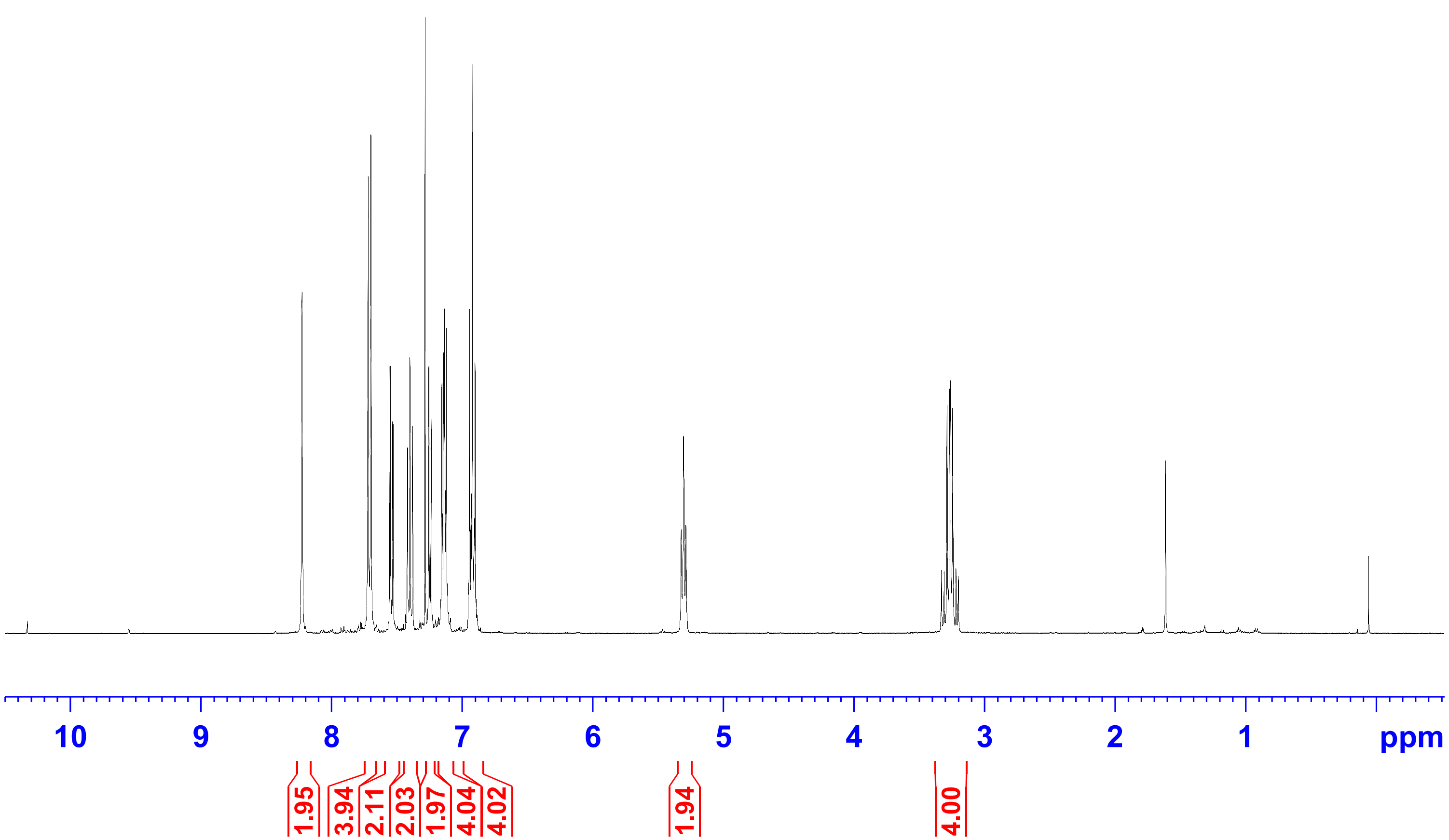

Current Data Parameters
NAME zrh-9-75-1-h
EXPNO 4
PROCNO 1

F2 - Acquisition Parameters
Date_ 20230425
Time 12.46 h
INSTRUM AvanceNeo 400MHz
PROBHD Z163739_0629 (
PULPROG zg30
TD 65536
SOLVENT CDCl3
NS 2
DS 2
SWH 8196.722 Hz
FIDRES 0.250144 Hz
AQ 3.9976959 sec
RG 101
DW 61.000 usec
DE 13.89 usec
TE 297.3 K
D1 1.00000000 sec
TD0 1
SFO1 400.1824711 MHz
NUC1 1H
P0 2.67 usec
P1 8.00 usec
PLW1 21.26700020 W

F2 - Processing parameters
SI 65536
SF 400.1800000 MHz
WDW EM
SSB 0
LB 0.30 Hz
GB 0
PC 1.00

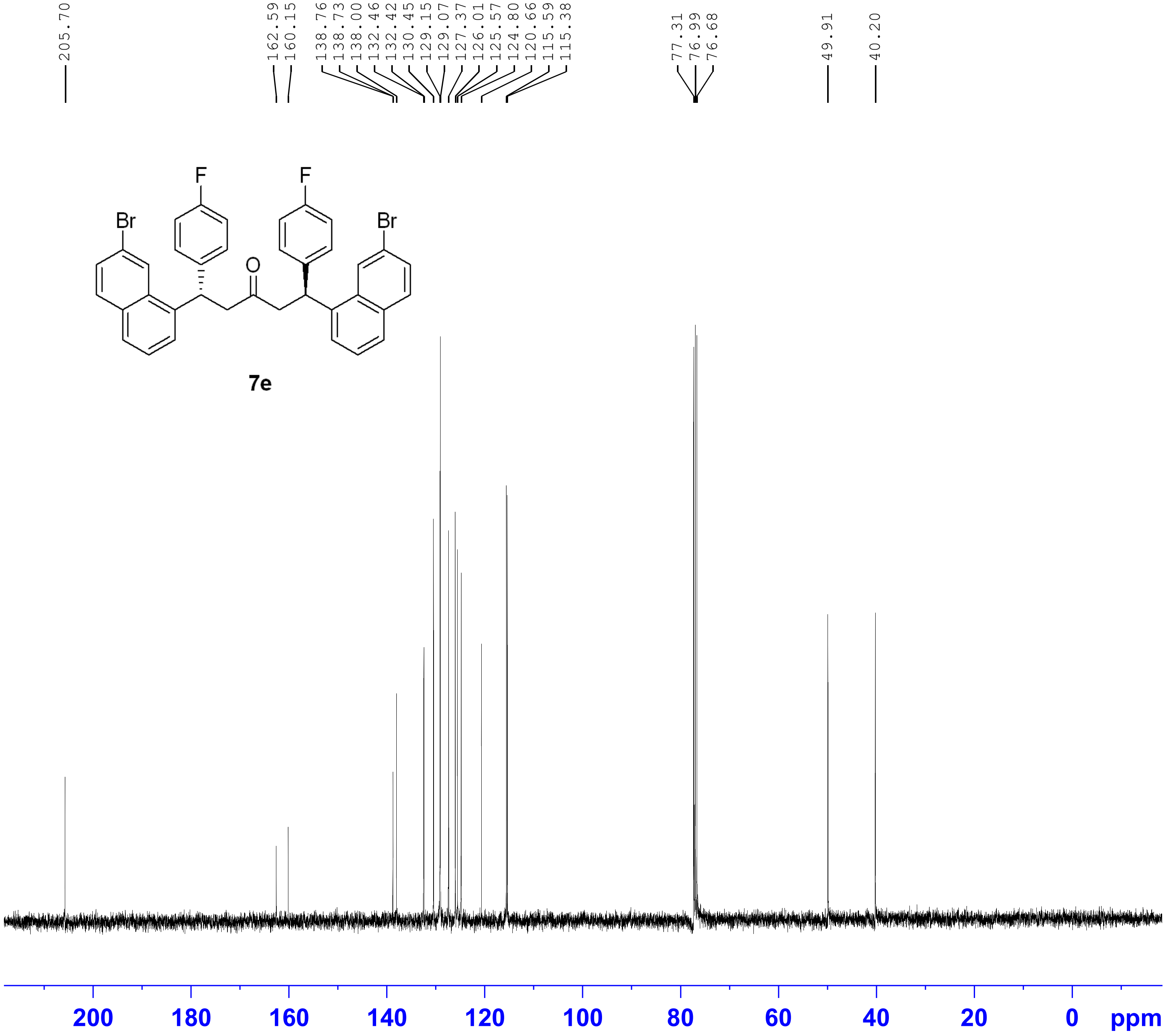


```
Current Data Parameters
NAME          zrh-9-75-1-c
EXPNO                    5
PROCNO                   1

F2 - Acquisition Parameters
Date_             20230425
Time                 12.50 h
INSTRUM   AvanceNeo 400MHz
PROBHD    Z163739_0629 (
PULPROG            zgpg30
TD                  65536
SOLVENT             CDCl3
NS                     54
DS                      4
SWH             23809.523 Hz
FIDRES           0.726609 Hz
AQ              1.3762560 sec
RG                     10
DW                 21.000 usec
DE                   6.50 usec
TE                  297.8 K
D1             2.00000000 sec
D11            0.03000000 sec
TD0                     1
SFO1          100.6354036 MHz
NUC1                  13C
P0                   2.67 usec
P1                   8.00 usec
PLW1          85.25399780 W
SFO2          400.1816007 MHz
NUC2                   1H
CPDPRG[2          waltz65
PCPD2               90.00 usec
PLW2          21.26700020 W
PLW12          0.16802999 W
PLW13          0.08452000 W

F2 - Processing parameters
SI                  32768
SF            100.6253513 MHz
WDW                    EM
SSB                     0
LB                   1.00 Hz
GB                      0
PC                   1.40
```

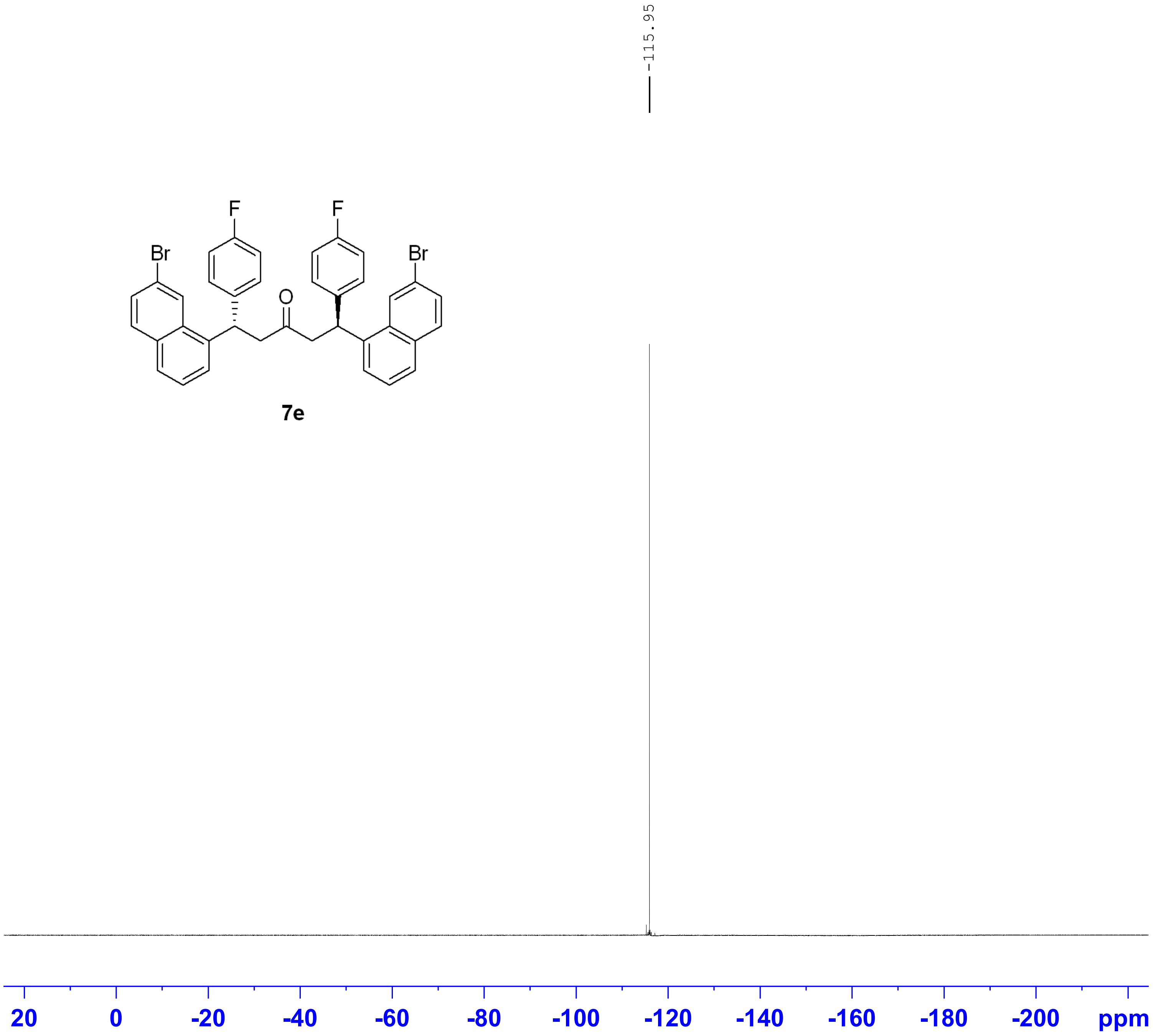

7e

```
Current Data Parameters
NAME          zrh-9-75-1-f
EXPNO                    1
PROCNO                   1

F2 - Acquisition Parameters
Date_             20230425
Time                 13.07
INSTRUM              spect
PROBHD    5 mm PABBO BB/
PULPROG            zgpg30
TD                  65536
SOLVENT             CDCl3
NS                      4
DS                      2
SWH             93750.000 Hz
FIDRES           1.430511 Hz
AQ              0.3495253 sec
RG                 196.92
DW                  5.333 usec
DE                   6.50 usec
TE                  296.2 K
D1             2.00000000 sec
D11            0.03000000 sec
TD0                     1

======== CHANNEL f1 ========
SFO1          376.4607162 MHz
NUC1                  19F
P1                  14.70 usec
PLW1          15.99600029 W

======== CHANNEL f2 ========
SFO2          400.1316005 MHz
NUC2                   1H
CPDPRG[2          waltz16
PCPD2               90.00 usec
PLW2          11.99499989 W
PLW12          0.34213999 W
PLW13          0.27713001 W

F2 - Processing parameters
SI                  32768
SF            376.4983660 MHz
WDW                    EM
SSB                     0
LB                   1.00 Hz
GB                      0
PC                   1.40
```

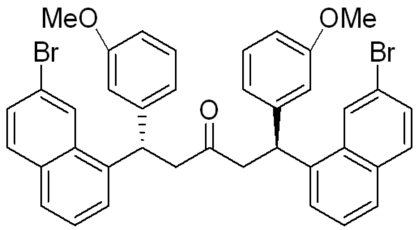

7f

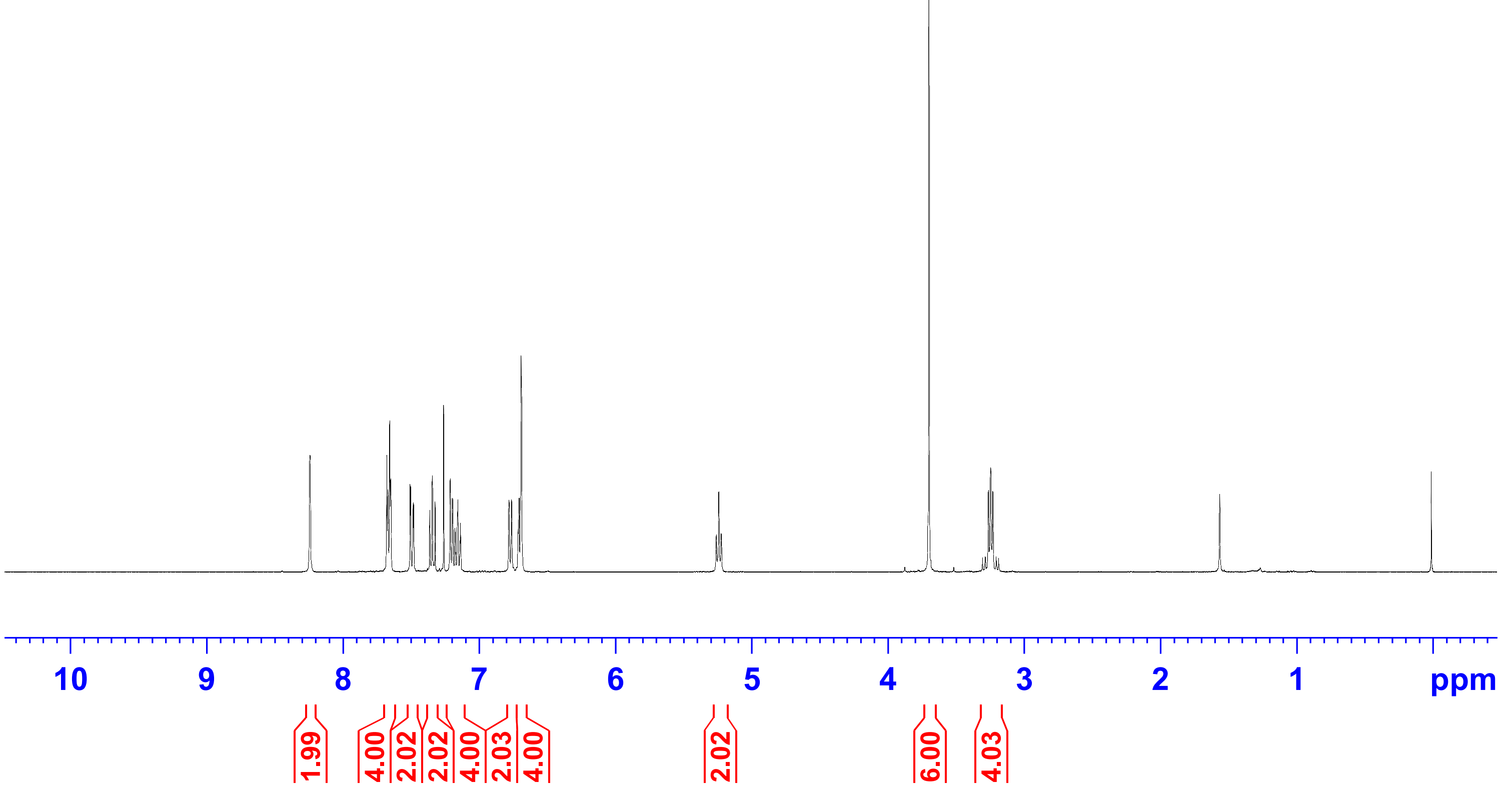


```
Current Data Parameters
NAME      zrh-9-77-3-re-h
EXPNO                   1
PROCNO                  1

F2 - Acquisition Parameters
Date_            20230425
Time                21.04
INSTRUM             spect
PROBHD    5 mm PABBO BB/
PULPROG              zg30
TD                  65536
SOLVENT             CDCl3
NS                      2
DS                      2
SWH              8012.820 Hz
FIDRES           0.122266 Hz
AQ              4.0894465 sec
RG                  88.84
DW                 62.400 usec
DE                   6.50 usec
TE                  296.5 K
D1             1.00000000 sec
TD0                     1

======== CHANNEL f1 ========
SFO1          400.1324710 MHz
NUC1                   1H
P1                  14.50 usec
PLW1          11.99499989 W

F2 - Processing parameters
SI                  65536
SF            400.1300100 MHz
WDW                    EM
SSB                     0
LB                   0.30 Hz
GB                      0
PC                   1.00
```

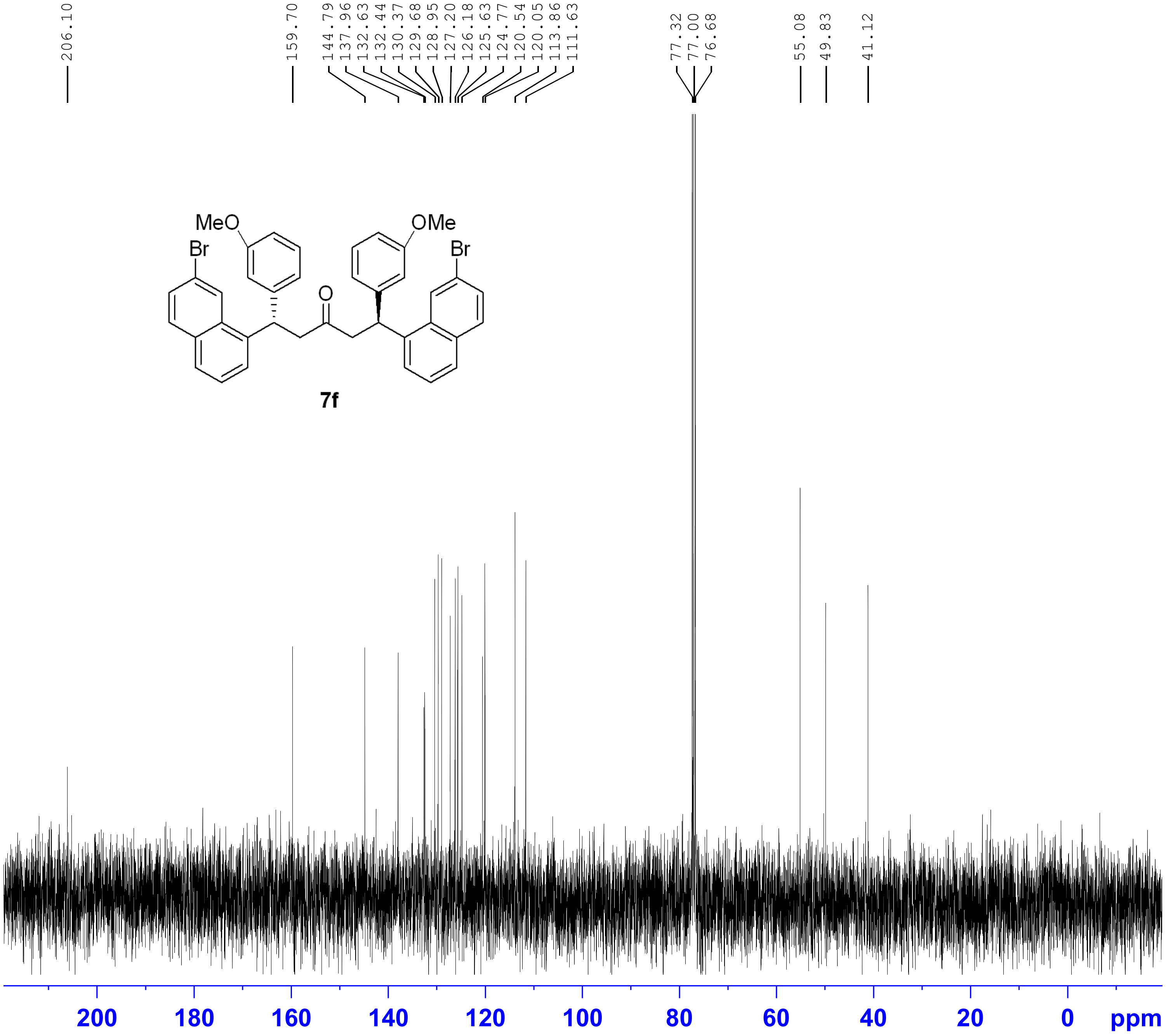

7f

```
Current Data Parameters
NAME          zrh-9-77-3-c
EXPNO                    1
PROCNO                   1

F2 - Acquisition Parameters
Date_             20230424
Time                 20.19
INSTRUM              spect
PROBHD    5 mm DUL 13C-1
PULPROG            zgpg30
TD                   65536
SOLVENT              CDCl3
NS                     143
DS                       0
SWH              24038.461 Hz
FIDRES            0.366798 Hz
AQ               1.3631488 sec
RG                    71.8
DW                  20.800 usec
DE                    6.00 usec
TE                   292.8 K
D1              2.00000000 sec
D11             0.03000000 sec
TD0                      1

======== CHANNEL f1 ========
NUC1                   13C
P1                   40.00 usec
PL1                  -3.00 dB
PL1W           60.64365387 W
SFO1           100.6228298 MHz

======== CHANNEL f2 ========
CPDPRG[2           waltz16
NUC2                    1H
PCPD2                80.00 usec
PL2                  -1.00 dB
PL12                 14.39 dB
PL13                 18.00 dB
PL2W           12.17476940 W
PL12W           0.35193357 W
PL13W           0.15327126 W
SFO2           400.1316005 MHz

F2 - Processing parameters
SI                   32768
SF             100.6127751 MHz
WDW                     EM
SSB                      0
LB                    1.00 Hz
GB                       0
PC                    1.40
```

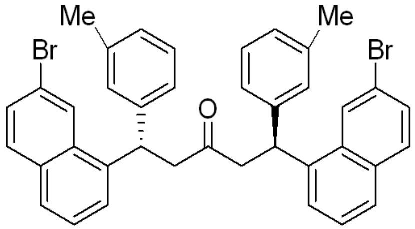

7g

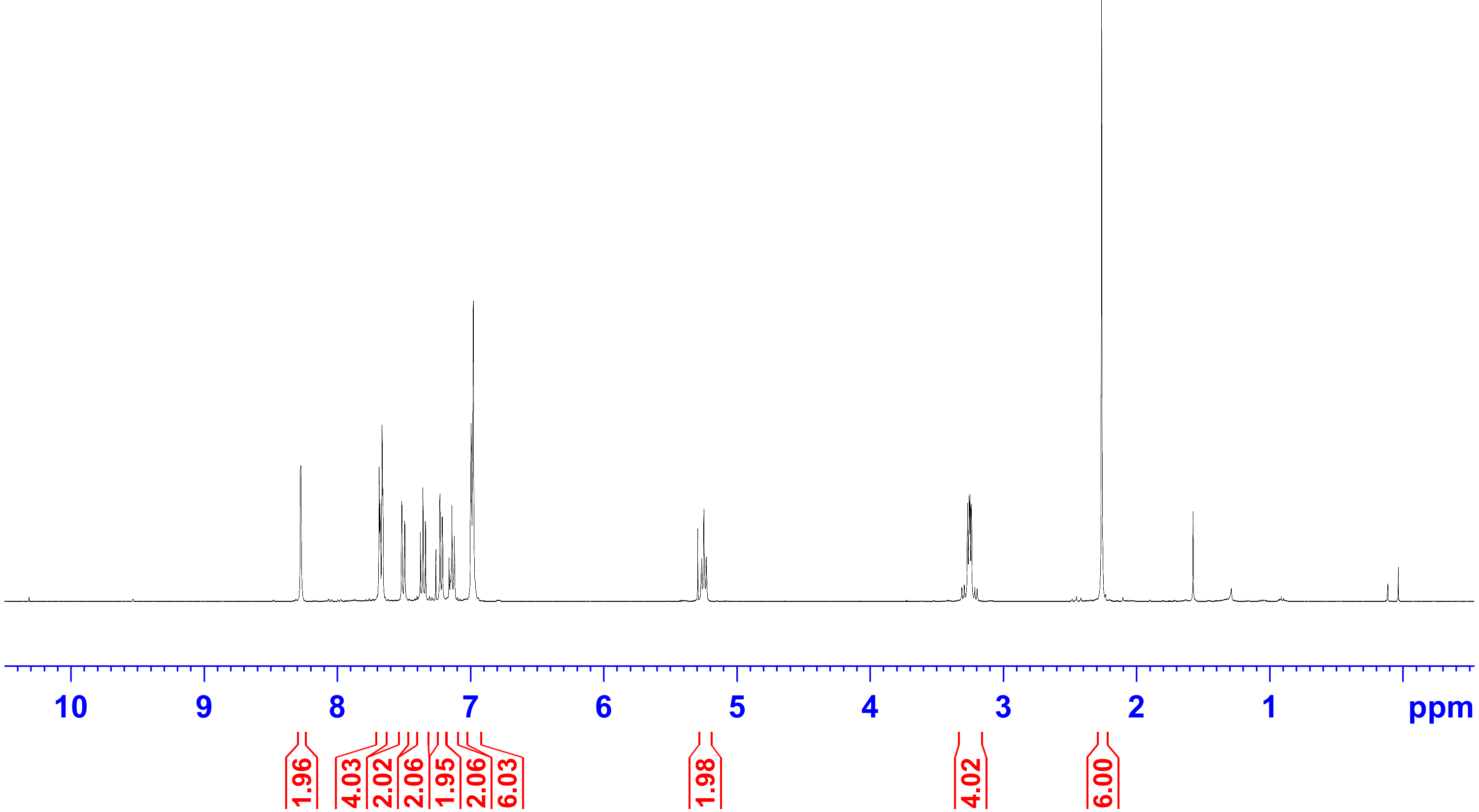

Current Data Parameters
NAME zrh-9-38-2-h
EXPNO 1
PROCNO 1

F2 - Acquisition Parameters
Date_ 20230327
Time 20.45
INSTRUM spect
PROBHD 5 mm DUL 13C-1
PULPROG zg30
TD 65536
SOLVENT CDCl3
NS 2
DS 0
SWH 8223.685 Hz
FIDRES 0.125483 Hz
AQ 3.9845889 sec
RG 203
DW 60.800 usec
DE 6.00 usec
TE 292.8 K
D1 1.00000000 sec
TD0 1

======== CHANNEL f1 ========
NUC1 1H
P1 15.80 usec
PL1 -1.00 dB
PL1W 12.17476940 W
SFO1 400.1324710 MHz

F2 - Processing parameters
SI 32768
SF 400.1300097 MHz
WDW EM
SSB 0
LB 0.30 Hz
GB 0
PC 1.00

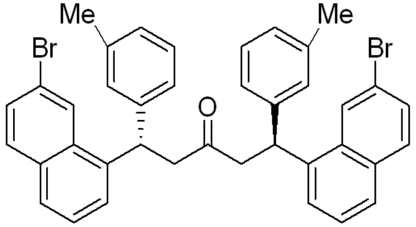

7g

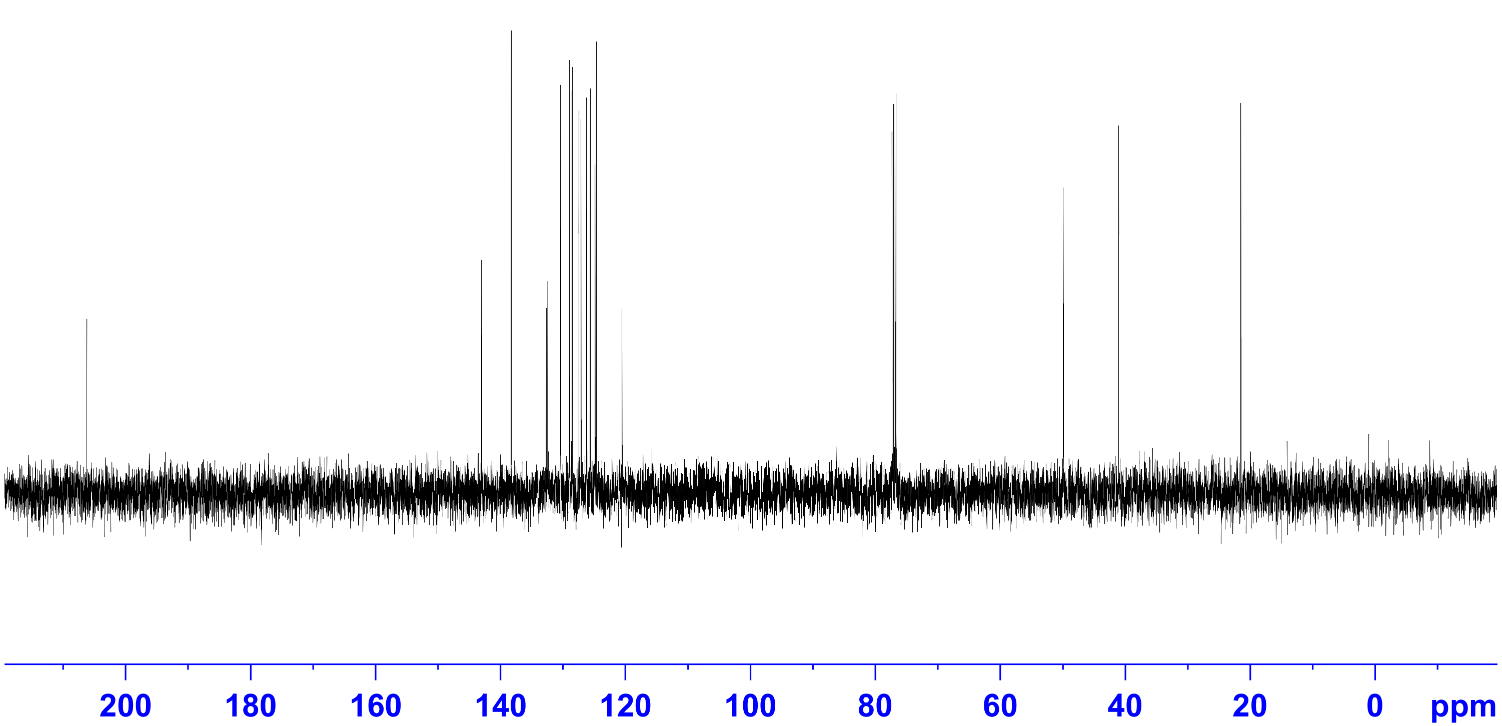

Current Data Parameters
NAME zrh-9-38-2-c
EXPNO 1
PROCNO 1

F2 - Acquisition Parameters
Date_ 20230327
Time 20.47
INSTRUM spect
PROBHD 5 mm DUL 13C-1
PULPROG zgpg30
TD 65536
SOLVENT CDCl3
NS 10
DS 0
SWH 24038.461 Hz
FIDRES 0.366798 Hz
AQ 1.3631488 sec
RG 2050
DW 20.800 usec
DE 6.00 usec
TE 292.9 K
D1 2.00000000 sec
D11 0.03000000 sec
TD0 1

======== CHANNEL f1 ========
NUC1 13C
P1 40.00 usec
PL1 -3.00 dB
PL1W 60.64365387 W
SFO1 100.6228298 MHz

======== CHANNEL f2 ========
CPDPRG[2 waltz16
NUC2 1H
PCPD2 80.00 usec
PL2 -1.00 dB
PL12 14.39 dB
PL13 18.00 dB
PL2W 12.17476940 W
PL12W 0.35193357 W
PL13W 0.15327126 W
SFO2 400.1316005 MHz

F2 - Processing parameters
SI 32768
SF 100.6127780 MHz
WDW EM
SSB 0
LB 1.00 Hz
GB 0
PC 1.40

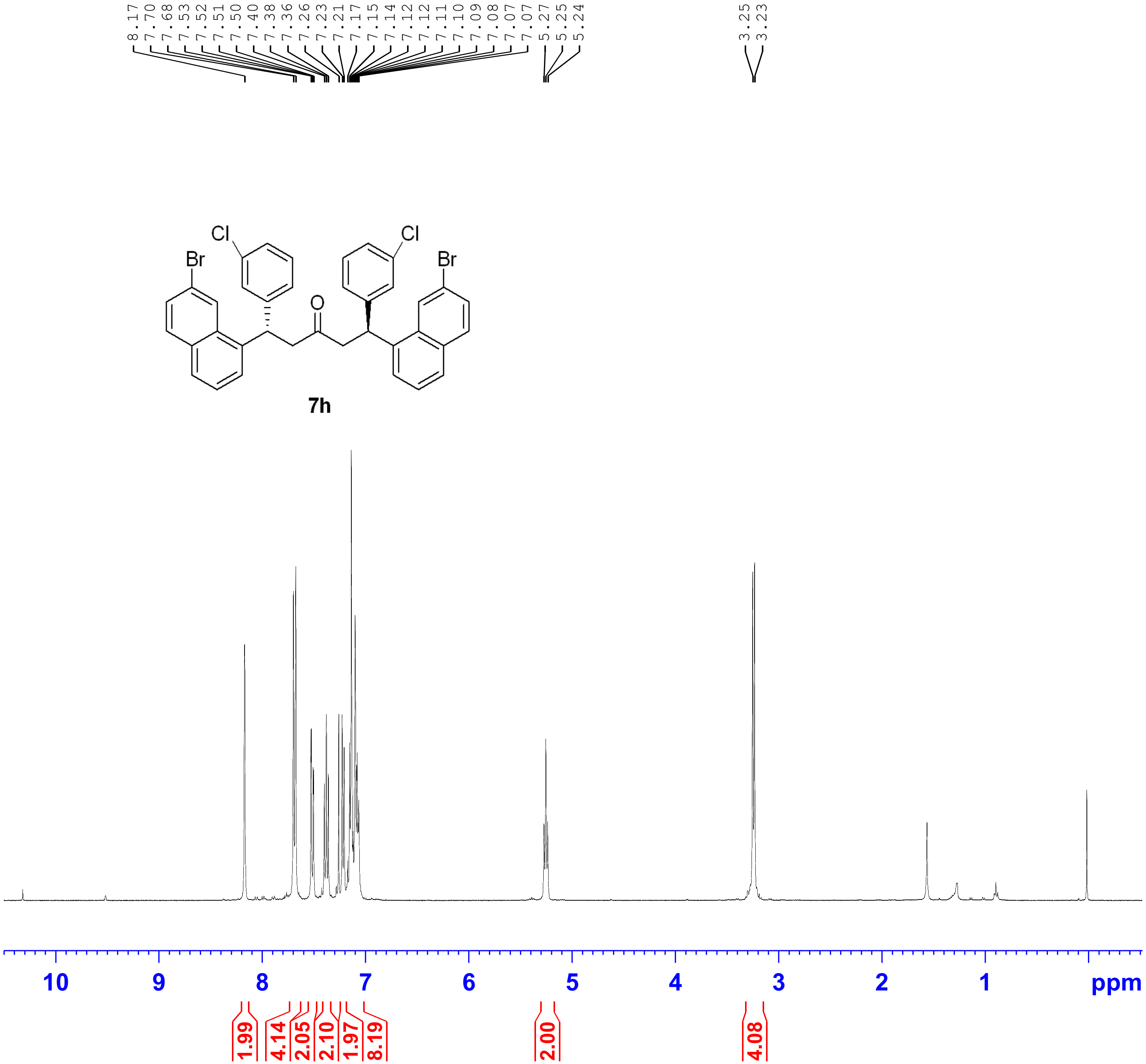


```
Current Data Parameters
NAME        zrh-9-77-1-h
EXPNO                  1
PROCNO                 1

F2 - Acquisition Parameters
Date_           20230425
Time               12.29 h
INSTRUM   AvanceNeo 400MHz
PROBHD    Z163739_0629 (
PULPROG             zg30
TD                 65536
SOLVENT            CDCl3
NS                     4
DS                     2
SWH             8196.722 Hz
FIDRES          0.250144 Hz
AQ             3.9976959 sec
RG                   101
DW                61.000 usec
DE                 13.89 usec
TE                 297.8 K
D1            1.00000000 sec
TD0                    1
SFO1         400.1824711 MHz
NUC1                  1H
P0                  2.67 usec
P1                  8.00 usec
PLW1         21.26700020 W

F2 - Processing parameters
SI                 65536
SF           400.1800094 MHz
WDW                   EM
SSB                    0
LB                  0.30 Hz
GB                     0
PC                  1.00
```

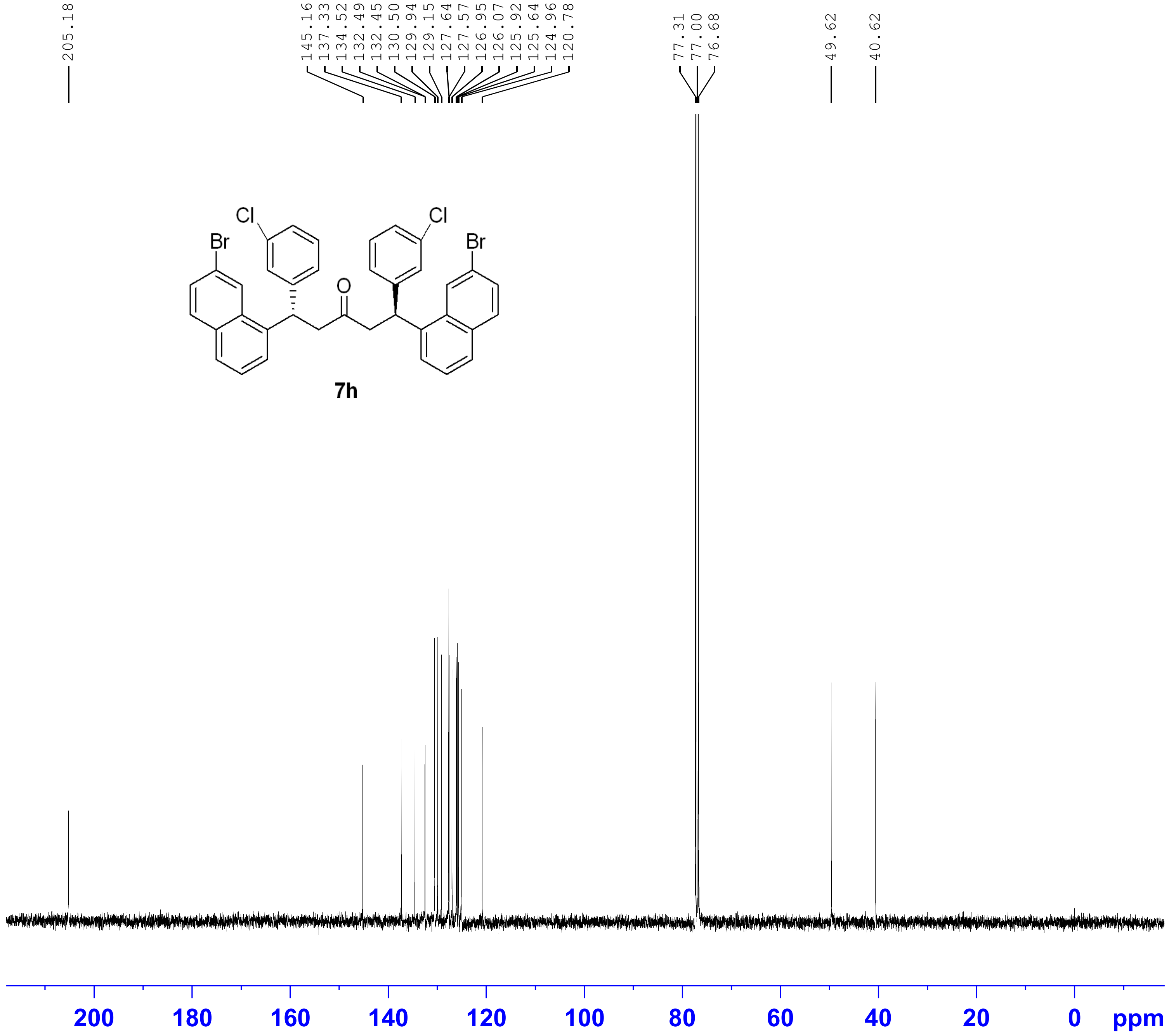

Current Data Parameters
NAME zrh-9-77-1-c
EXPNO 3
PROCNO 1

F2 - Acquisition Parameters
Date_ 20230425
Time 12.42 h
INSTRUM AvanceNeo 400MHz
PROBHD Z163739_0629 (
PULPROG zgpg30
TD 65536
SOLVENT CDCl3
NS 177
DS 4
SWH 23809.523 Hz
FIDRES 0.726609 Hz
AQ 1.3762560 sec
RG 10
DW 21.000 usec
DE 6.50 usec
TE 298.1 K
D1 2.00000000 sec
D11 0.03000000 sec
TD0 1
SFO1 100.6354036 MHz
NUC1 13C
P0 2.67 usec
P1 8.00 usec
PLW1 85.25399780 W
SFO2 400.1816007 MHz
NUC2 1H
CPDPRG[2 waltz65
PCPD2 90.00 usec
PLW2 21.26700020 W
PLW12 0.16802999 W
PLW13 0.08452000 W

F2 - Processing parameters
SI 32768
SF 100.6253470 MHz
WDW EM
SSB 0
LB 1.00 Hz
GB 0
PC 1.40

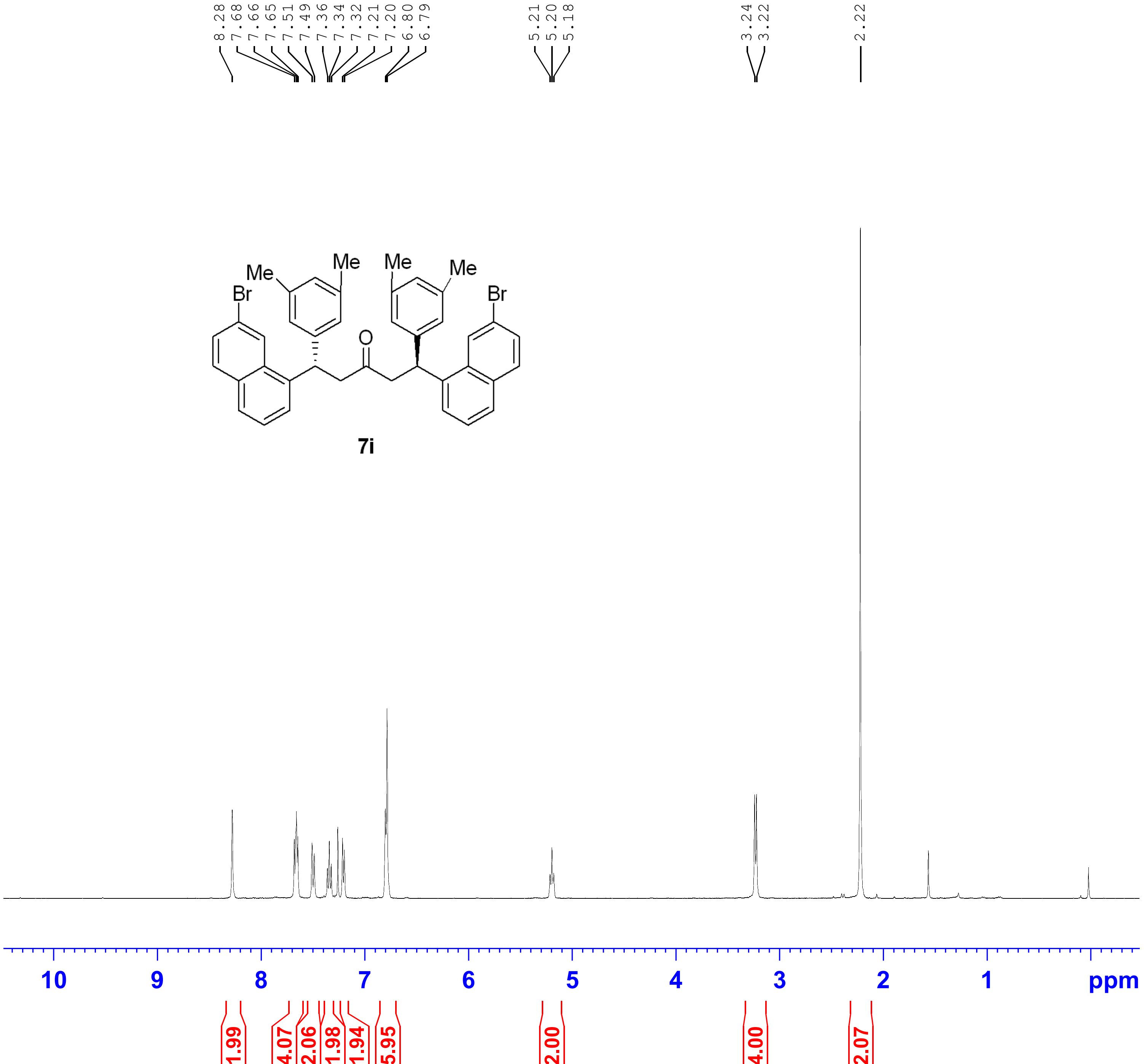

7i

```
Current Data Parameters
NAME          zrh-9-77-2-h
EXPNO                    1
PROCNO                   1

F2 - Acquisition Parameters
Date_             20230424
Time                 20.04
INSTRUM              spect
PROBHD     5 mm DUL 13C-1
PULPROG               zg30
TD                   65536
SOLVENT              CDCl3
NS                       2
DS                       0
SWH               8223.685 Hz
FIDRES            0.125483 Hz
AQ               3.9845889 sec
RG                     256
DW                  60.800 usec
DE                    6.00 usec
TE                   292.5 K
D1              1.00000000 sec
TD0                      1

======== CHANNEL f1 ========
NUC1                    1H
P1                   15.80 usec
PL1                  -1.00 dB
PL1W           12.17476940 W
SFO1           400.1324710 MHz

F2 - Processing parameters
SI                   32768
SF             400.1300099 MHz
WDW                     EM
SSB                      0
LB                    0.30 Hz
GB                       0
PC                    1.00
```

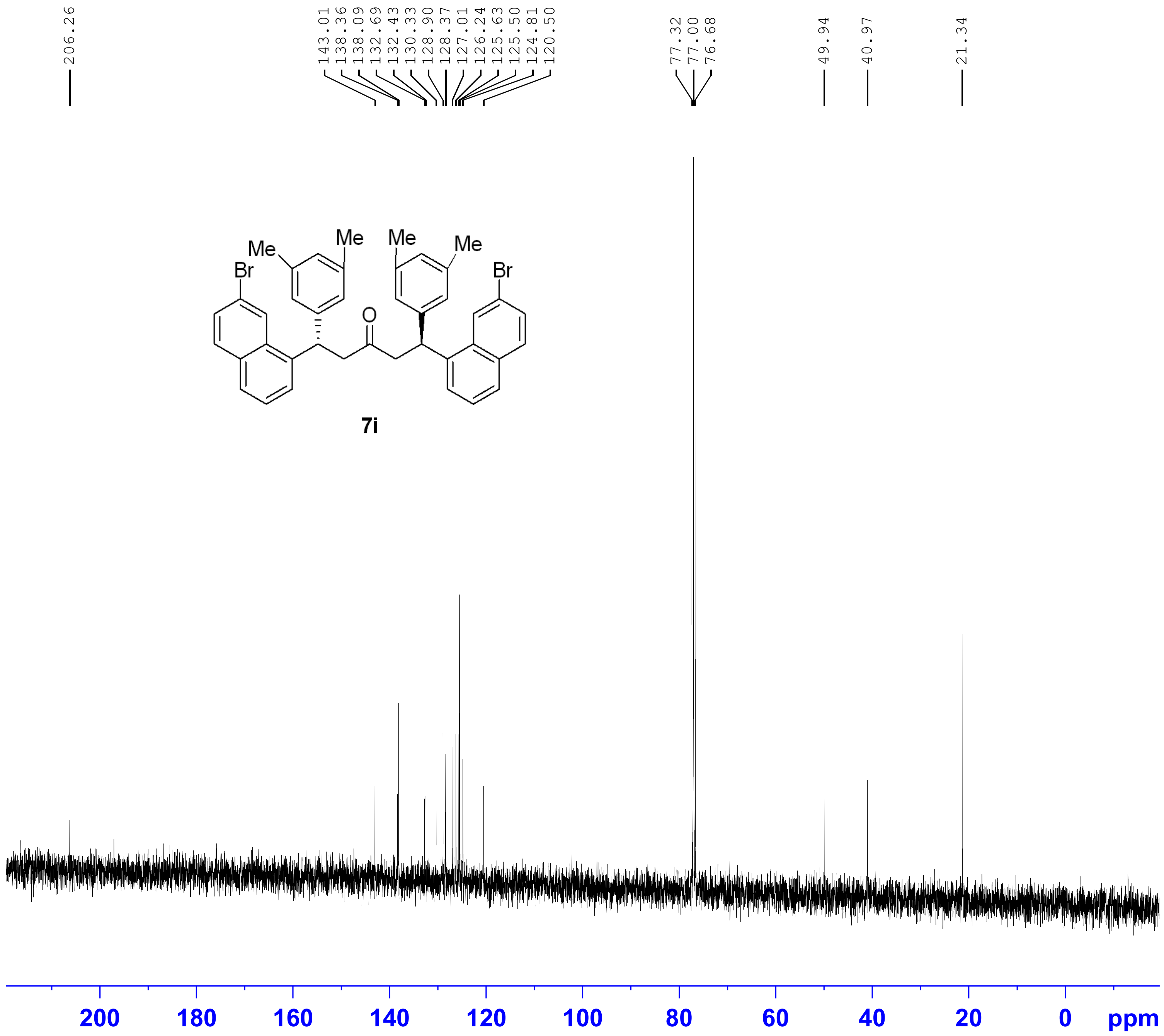


```
Current Data Parameters
NAME         zrh-9-77-2-c
EXPNO                   1
PROCNO                  1

F2 - Acquisition Parameters
Date_            20230424
Time                20.06
INSTRUM             spect
PROBHD   5 mm DUL 13C-1
PULPROG           zgpg30
TD                  65536
SOLVENT             CDCl3
NS                    161
DS                      0
SWH             24038.461 Hz
FIDRES           0.366798 Hz
AQ              1.3631488 sec
RG                   71.8
DW                 20.800 usec
DE                   6.00 usec
TE                  292.7 K
D1             2.00000000 sec
D11            0.03000000 sec
TD0                     1

======== CHANNEL f1 ========
NUC1                  13C
P1                  40.00 usec
PL1                 -3.00 dB
PL1W          60.64365387 W
SFO1          100.6228298 MHz

======== CHANNEL f2 ========
CPDPRG[2          waltz16
NUC2                   1H
PCPD2               80.00 usec
PL2                 -1.00 dB
PL12                14.39 dB
PL13                18.00 dB
PL2W          12.17476940 W
PL12W          0.35193357 W
PL13W          0.15327126 W
SFO2          400.1316005 MHz

F2 - Processing parameters
SI                  32768
SF            100.6127754 MHz
WDW                    EM
SSB                     0
LB                   1.00 Hz
GB                      0
PC                   1.40
```

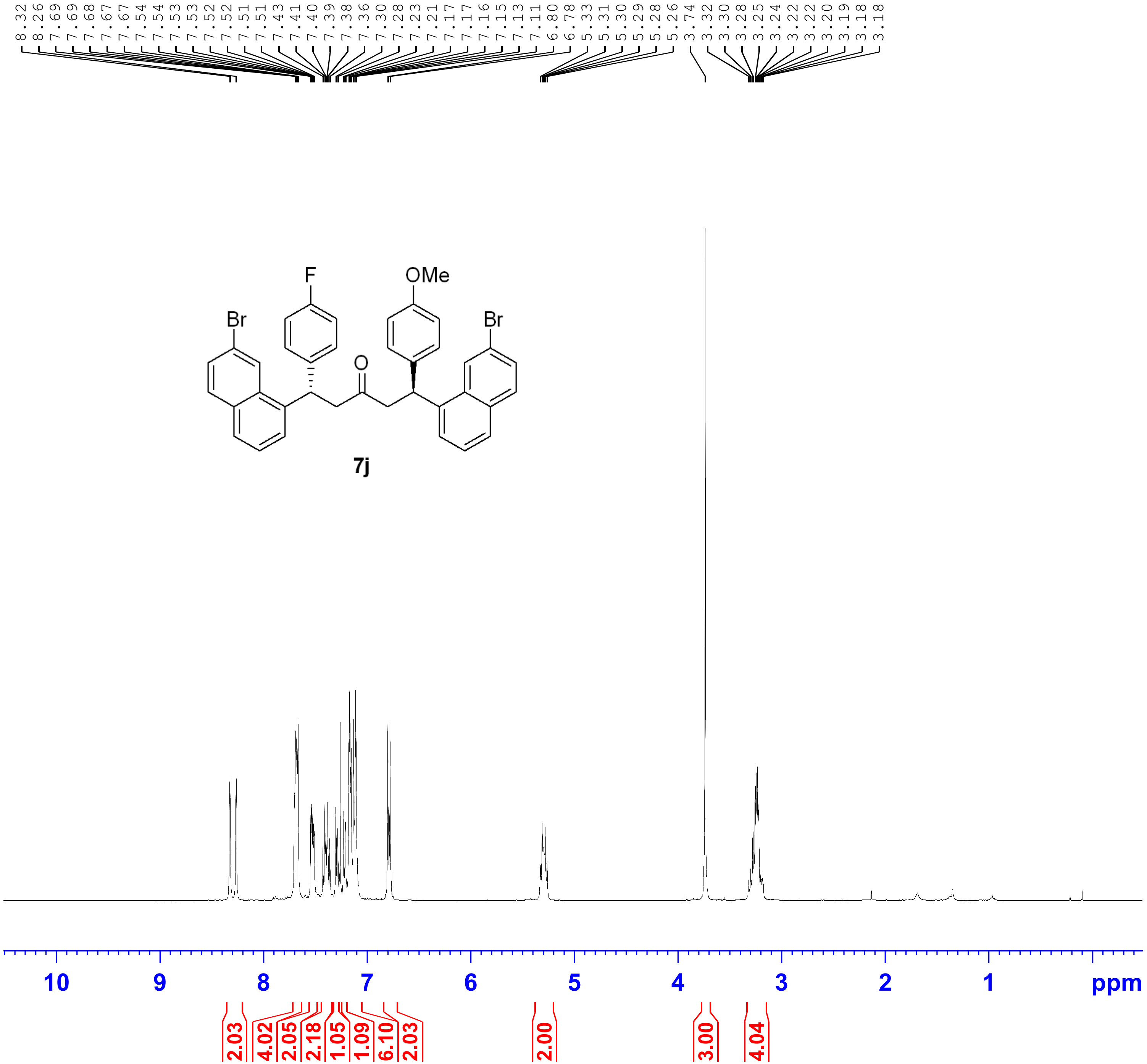

Current Data Parameters
NAME zrh-9-86-h
EXPNO 1
PROCNO 1

F2 - Acquisition Parameters
Date_ 20230501
Time 18.57
INSTRUM spect
PROBHD 5 mm PABBO BB/
PULPROG zg30
TD 65536
SOLVENT CDCl3
NS 4
DS 2
SWH 8012.820 Hz
FIDRES 0.122266 Hz
AQ 4.0894465 sec
RG 15.71
DW 62.400 usec
DE 6.50 usec
TE 296.0 K
D1 1.00000000 sec
TD0 1

======== CHANNEL f1 ========
SFO1 400.1324710 MHz
NUC1 1H
P1 14.50 usec
PLW1 11.99499989 W

F2 - Processing parameters
SI 65536
SF 400.1300099 MHz
WDW EM
SSB 0
LB 0.30 Hz
GB 0
PC 1.00

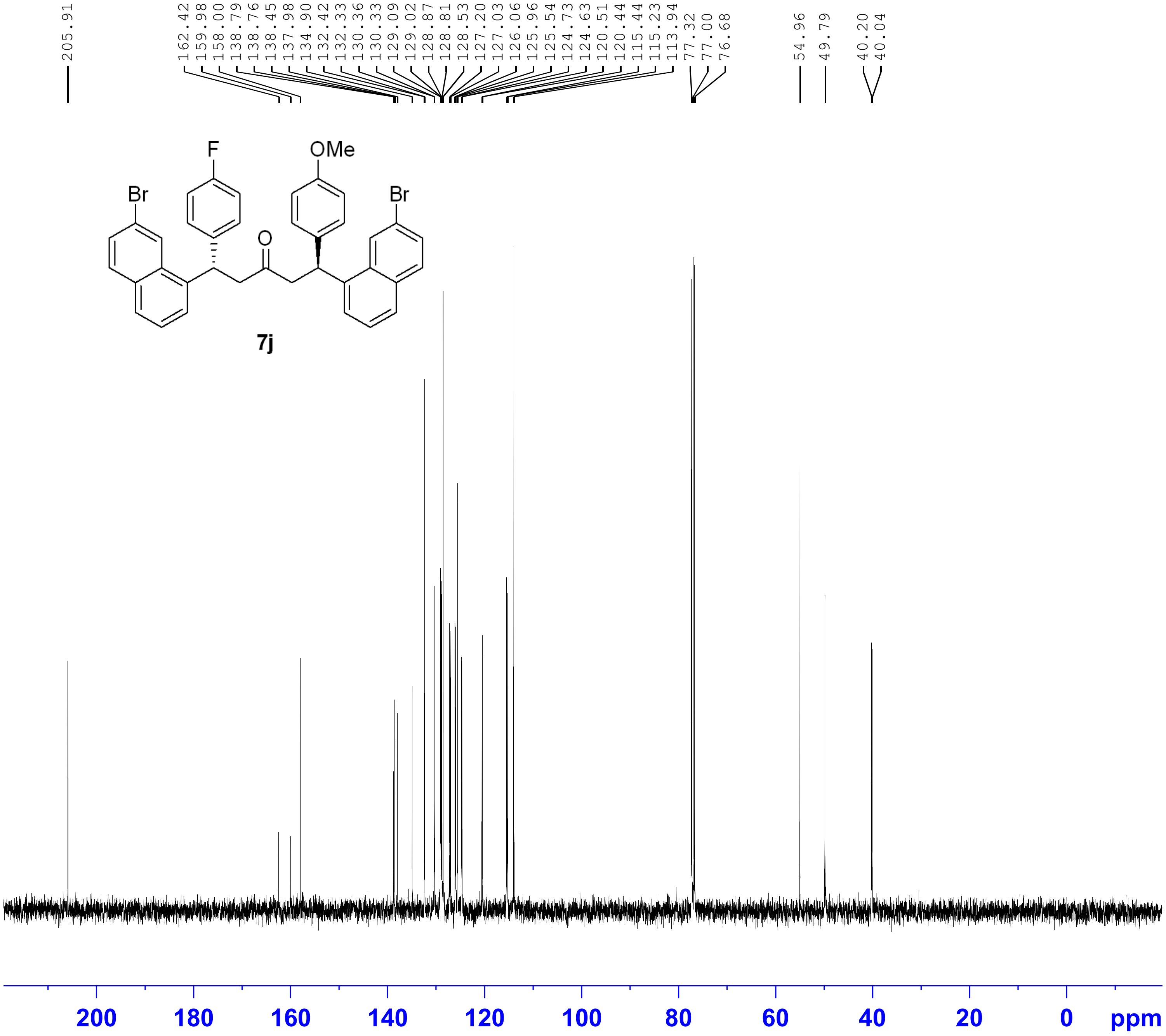

7j

Current Data Parameters
NAME zrh-9-86-c
EXPNO 3
PROCNO 1

F2 - Acquisition Parameters
Date_ 20230501
Time 19.04
INSTRUM spect
PROBHD 5 mm PABBO BB/
PULPROG zgpg30
TD 65536
SOLVENT CDCl3
NS 50
DS 2
SWH 24038.461 Hz
FIDRES 0.366798 Hz
AQ 1.3631488 sec
RG 196.92
DW 20.800 usec
DE 6.50 usec
TE 296.5 K
D1 2.00000000 sec
D11 0.03000000 sec
TD0 1

======== CHANNEL f1 ========
SFO1 100.6228298 MHz
NUC1 13C
P1 9.70 usec
PLW1 46.98899841 W

======== CHANNEL f2 ========
SFO2 400.1316005 MHz
NUC2 1H
CPDPRG[2 waltz16
PCPD2 90.00 usec
PLW2 11.99499989 W
PLW12 0.34213999 W
PLW13 0.27713001 W

F2 - Processing parameters
SI 32768
SF 100.6127978 MHz
WDW EM
SSB 0
LB 1.00 Hz
GB 0
PC 1.40

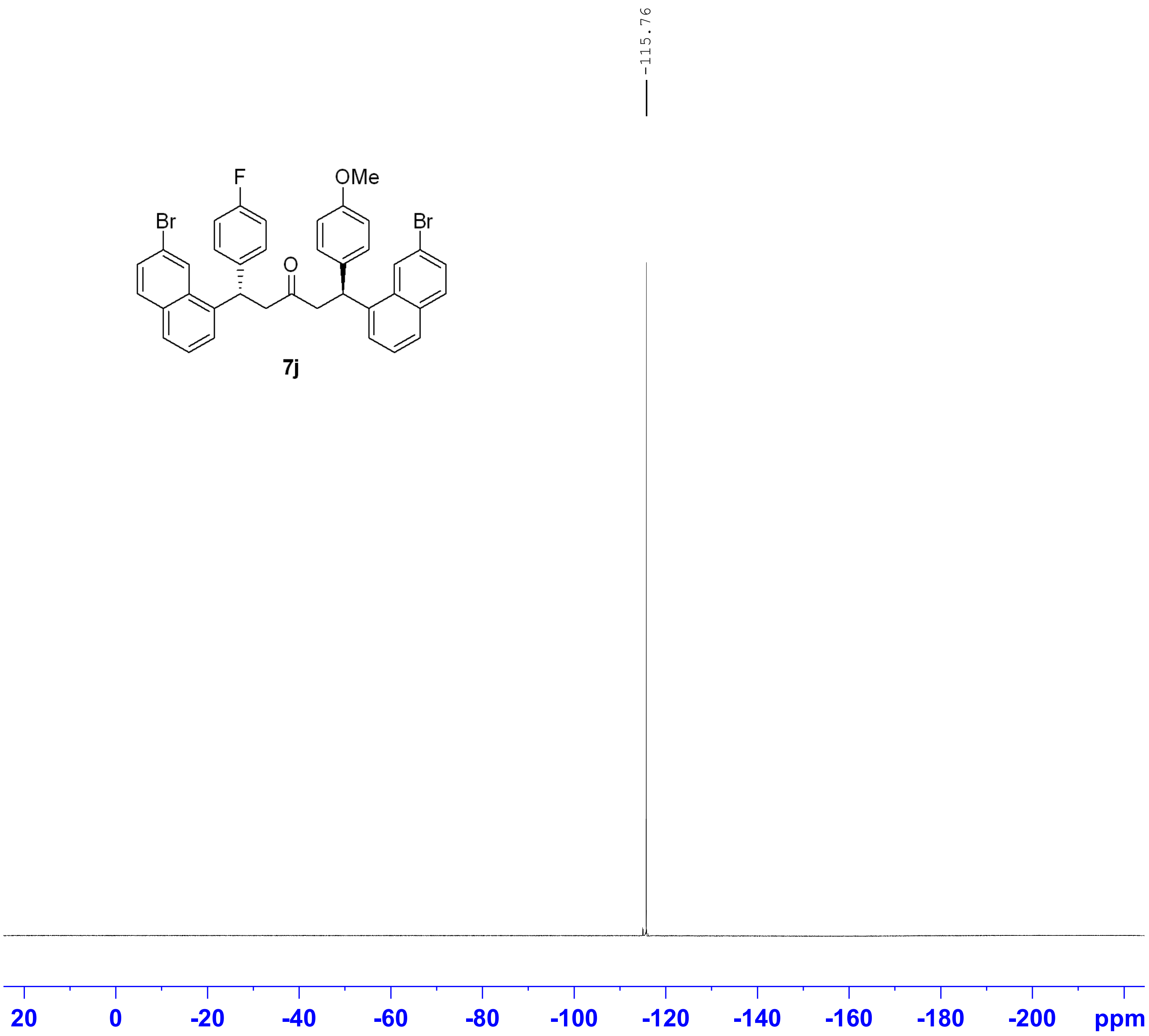

Current Data Parameters
NAME zrh-9-86-f
EXPNO 3
PROCNO 1

F2 - Acquisition Parameters
Date_ 20230501
Time 19.08
INSTRUM spect
PROBHD 5 mm PABBO BB/
PULPROG zgpg30
TD 65536
SOLVENT CDCl3
NS 4
DS 2
SWH 93750.000 Hz
FIDRES 1.430511 Hz
AQ 0.3495253 sec
RG 196.92
DW 5.333 usec
DE 6.50 usec
TE 296.3 K
D1 2.00000000 sec
D11 0.03000000 sec
TD0 1

======== CHANNEL f1 ========
SFO1 376.4607162 MHz
NUC1 19F
P1 14.70 usec
PLW1 15.99600029 W

======== CHANNEL f2 ========
SFO2 400.1316005 MHz
NUC2 1H
CPDPRG[2 waltz16
PCPD2 90.00 usec
PLW2 11.99499989 W
PLW12 0.34213999 W
PLW13 0.27713001 W

F2 - Processing parameters
SI 32768
SF 376.4983660 MHz
WDW EM
SSB 0
LB 1.00 Hz
GB 0
PC 1.40

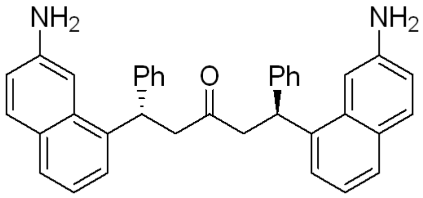

8a

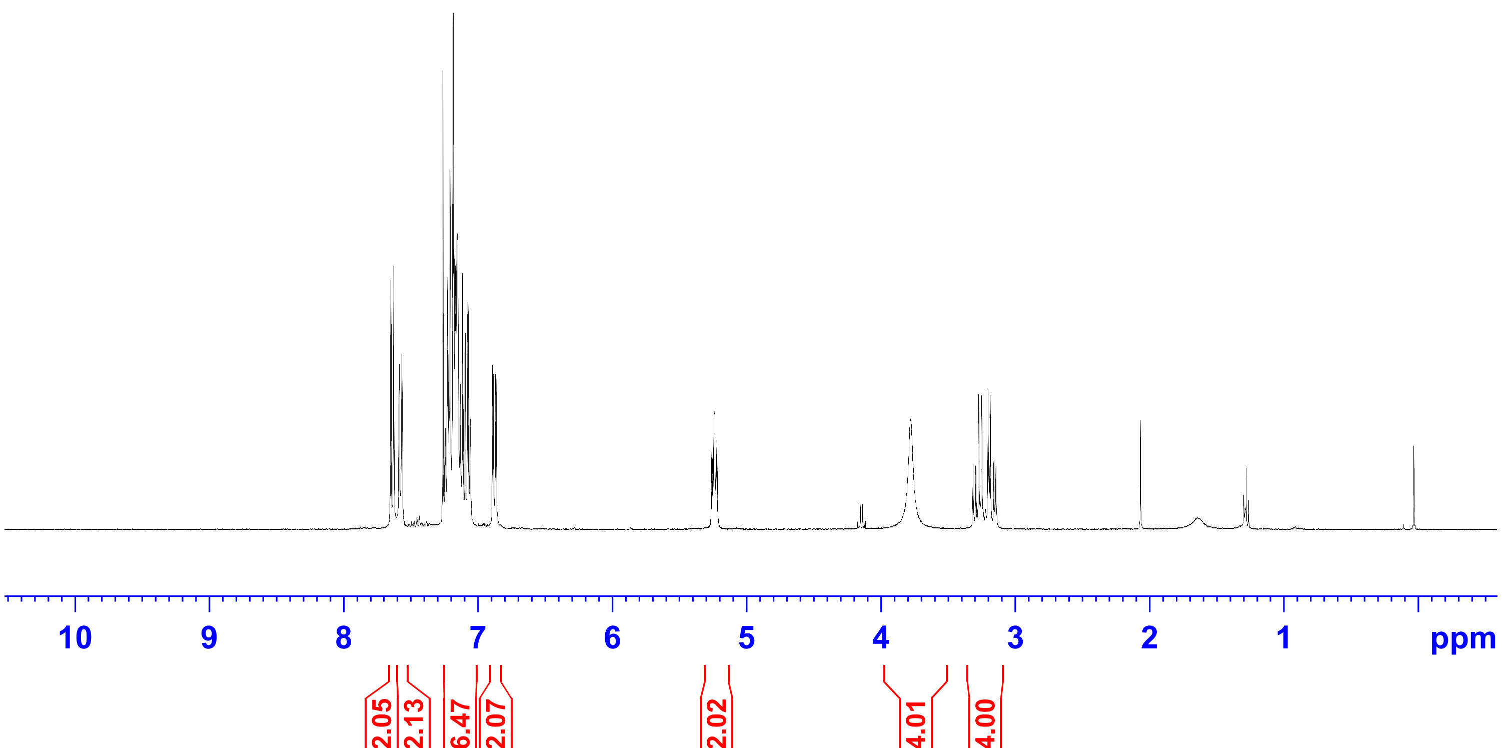

Current Data Parameters
NAME zrh-9-41-nh2-h
EXPNO 1
PROCNO 1

F2 - Acquisition Parameters
Date_ 20230328
Time 19.05
INSTRUM spect
PROBHD 5 mm DUL 13C-1
PULPROG zg30
TD 65536
SOLVENT CDCl3
NS 2
DS 0
SWH 8223.685 Hz
FIDRES 0.125483 Hz
AQ 3.9845889 sec
RG 228
DW 60.800 usec
DE 6.00 usec
TE 292.6 K
D1 1.00000000 sec
TD0 1

======== CHANNEL f1 ========
NUC1 1H
P1 15.80 usec
PL1 -1.00 dB
PL1W 12.17476940 W
SFO1 400.1324710 MHz

F2 - Processing parameters
SI 32768
SF 400.1300096 MHz
WDW EM
SSB 0
LB 0.30 Hz
GB 0
PC 1.00

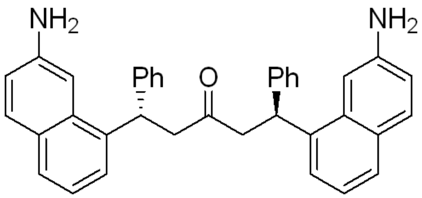

8a

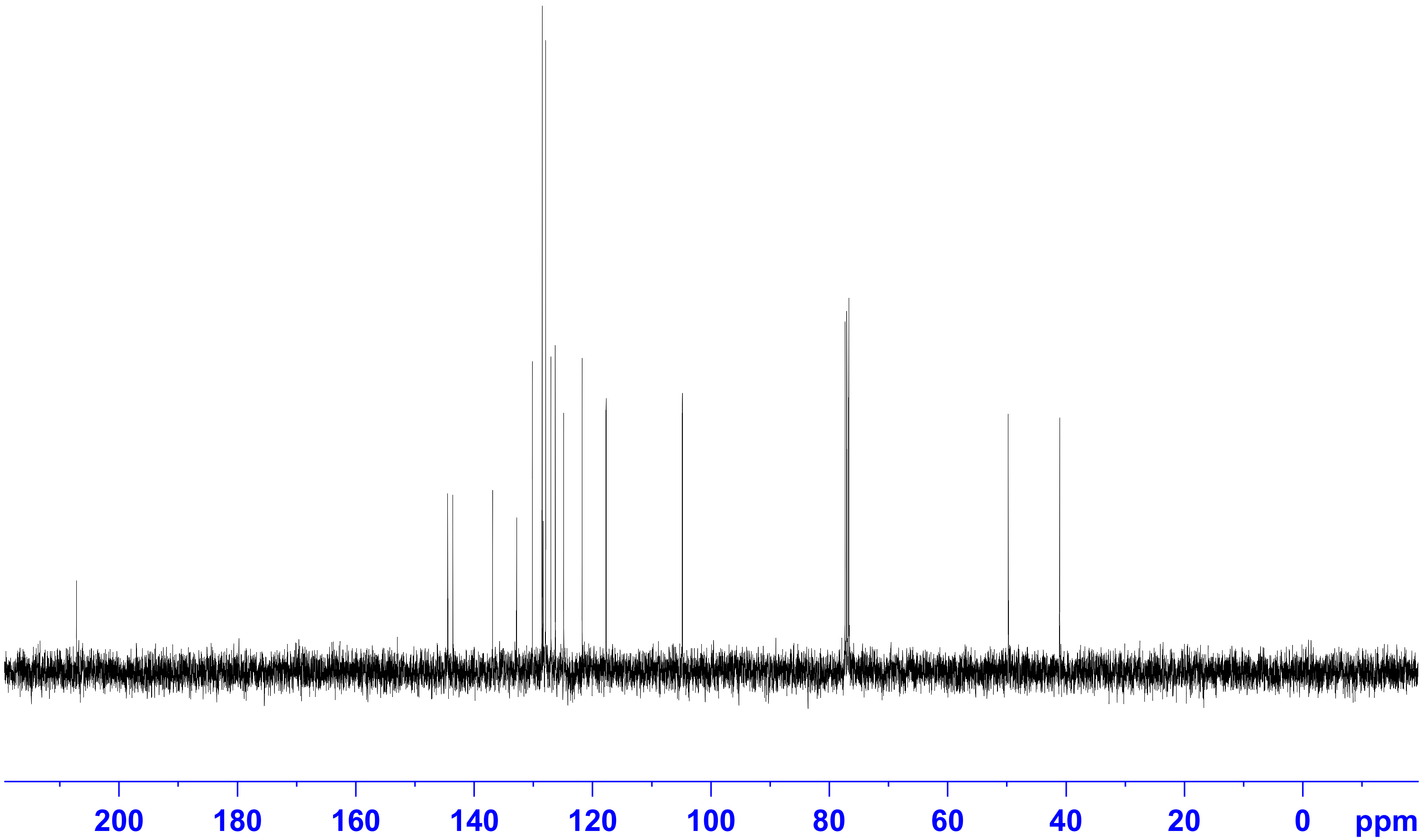


```
Current Data Parameters
NAME     zrh-9-41-nh2-c
EXPNO                 1
PROCNO                1

F2 - Acquisition Parameters
Date_          20230328
Time              19.07
INSTRUM           spect
PROBHD   5 mm DUL 13C-1
PULPROG          zgpg30
TD                65536
SOLVENT           CDCl3
NS                   20
DS                    0
SWH           24038.461 Hz
FIDRES         0.366798 Hz
AQ            1.3631488 sec
RG                 2050
DW               20.800 usec
DE                 6.00 usec
TE                292.7 K
D1           2.00000000 sec
D11          0.03000000 sec
TD0                   1

======== CHANNEL f1 ========
NUC1                13C
P1                40.00 usec
PL1               -3.00 dB
PL1W        60.64365387 W
SFO1       100.6228298 MHz

======== CHANNEL f2 ========
CPDPRG[2        waltz16
NUC2                 1H
PCPD2             80.00 usec
PL2               -1.00 dB
PL12              14.39 dB
PL13              18.00 dB
PL2W        12.17476940 W
PL12W        0.35193357 W
PL13W        0.15327126 W
SFO2       400.1316005 MHz

F2 - Processing parameters
SI                32768
SF          100.6127787 MHz
WDW                  EM
SSB                   0
LB                 1.00 Hz
GB                    0
PC                 1.40
```

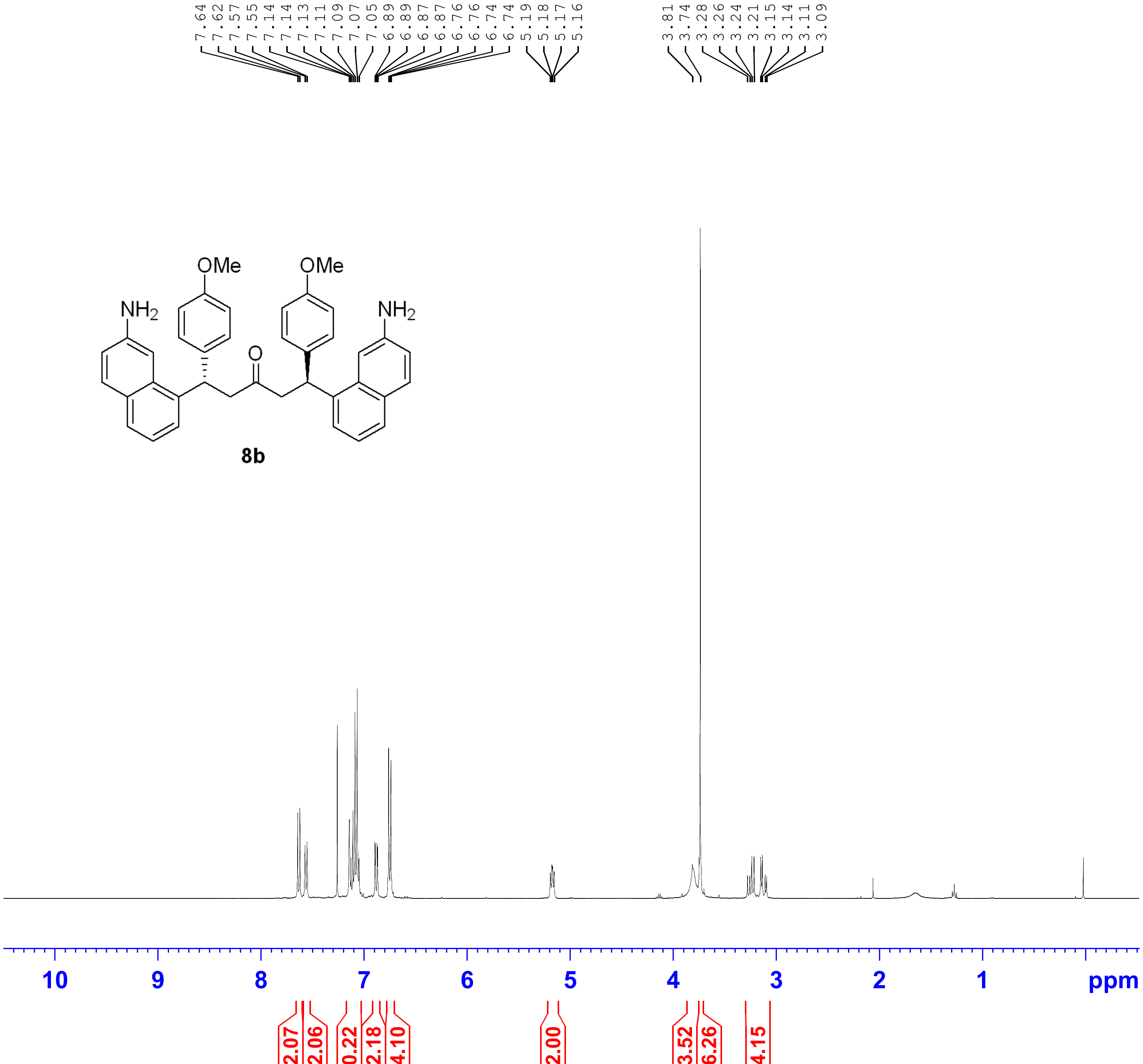

Current Data Parameters
NAME zrh-9-43-nh2-h
EXPNO 1
PROCNO 1

F2 - Acquisition Parameters
Date_ 20230330
Time 13.34
INSTRUM spect
PROBHD 5 mm DUL 13C-1
PULPROG zg30
TD 65536
SOLVENT CDCl3
NS 7
DS 0
SWH 8223.685 Hz
FIDRES 0.125483 Hz
AQ 3.9845889 sec
RG 256
DW 60.800 usec
DE 6.00 usec
TE 292.8 K
D1 1.00000000 sec
TD0 1

======== CHANNEL f1 ========
NUC1 1H
P1 15.80 usec
PL1 -1.00 dB
PL1W 12.17476940 W
SFO1 400.1324710 MHz

F2 - Processing parameters
SI 32768
SF 400.1300096 MHz
WDW EM
SSB 0
LB 0.30 Hz
GB 0
PC 1.00

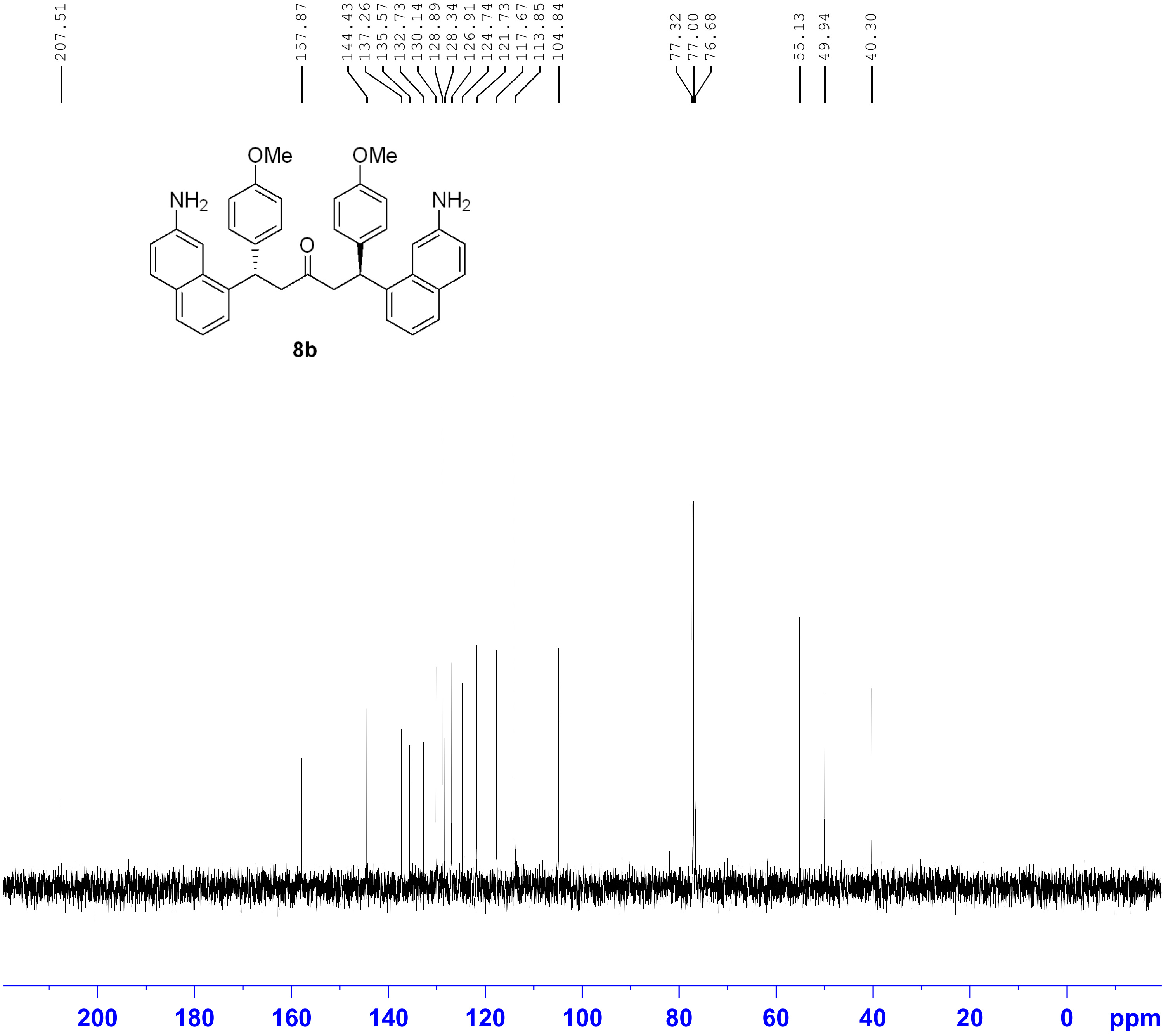

Current Data Parameters
NAME zrh-9-43-nh2-c
EXPNO 1
PROCNO 1

F2 - Acquisition Parameters
Date_ 20230330
Time 13.36
INSTRUM spect
PROBHD 5 mm DUL 13C-1
PULPROG zgpg30
TD 65536
SOLVENT CDCl3
NS 33
DS 0
SWH 24038.461 Hz
FIDRES 0.366798 Hz
AQ 1.3631488 sec
RG 2050
DW 20.800 usec
DE 6.00 usec
TE 293.1 K
D1 2.00000000 sec
D11 0.03000000 sec
TD0 1

======== CHANNEL f1 ========
NUC1 13C
P1 40.00 usec
PL1 -3.00 dB
PL1W 60.64365387 W
SFO1 100.6228298 MHz

======== CHANNEL f2 ========
CPDPRG[2 waltz16
NUC2 1H
PCPD2 80.00 usec
PL2 -1.00 dB
PL12 14.39 dB
PL13 18.00 dB
PL2W 12.17476940 W
PL12W 0.35193357 W
PL13W 0.15327126 W
SFO2 400.1316005 MHz

F2 - Processing parameters
SI 32768
SF 100.6127766 MHz
WDW EM
SSB 0
LB 1.00 Hz
GB 0
PC 1.40

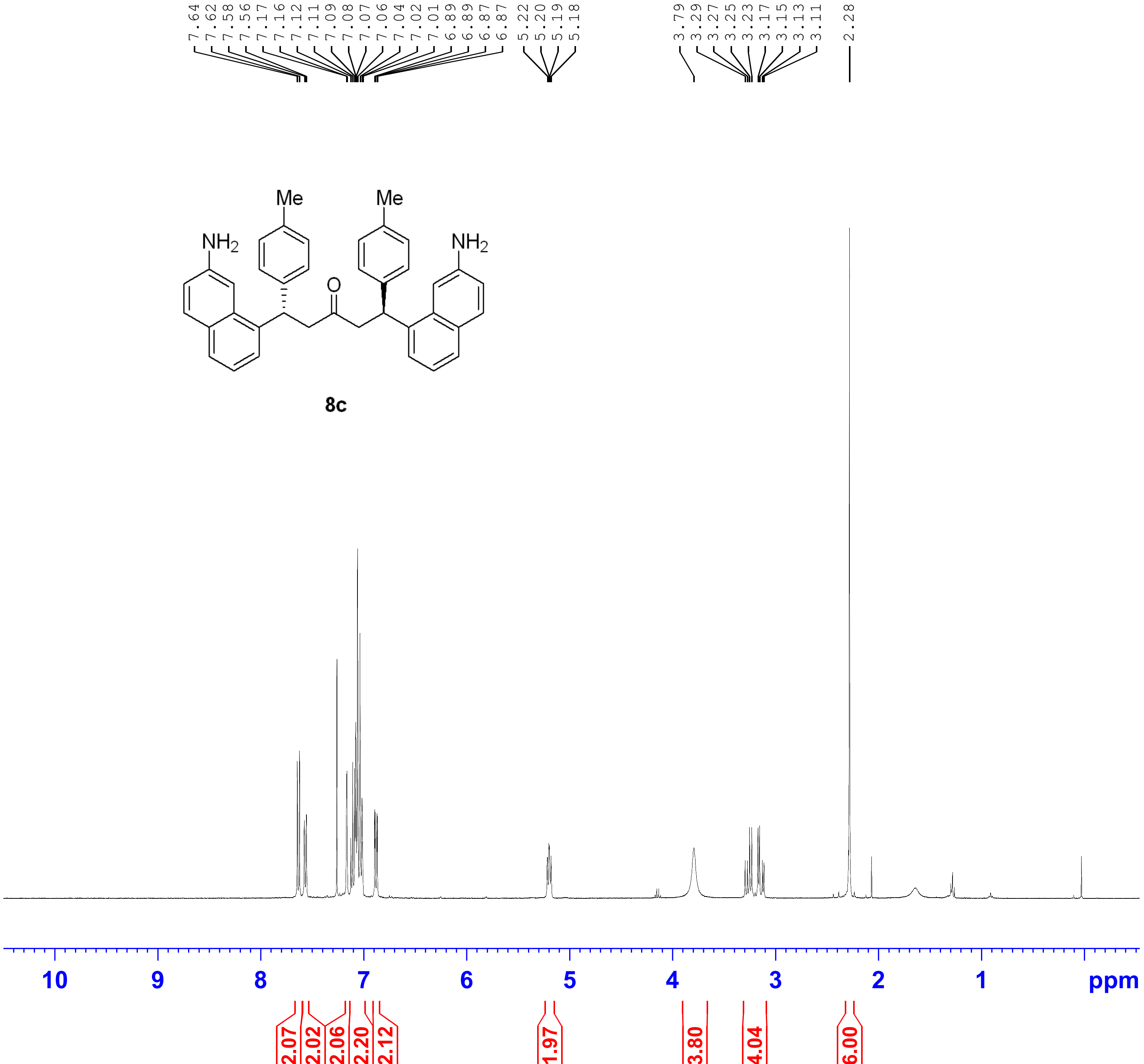


```
Current Data Parameters
NAME          zrh-9-51-1-h
EXPNO                    1
PROCNO                   1

F2 - Acquisition Parameters
Date_             20230404
Time                 19.20
INSTRUM              spect
PROBHD    5 mm DUL 13C-1
PULPROG               zg30
TD                   65536
SOLVENT              CDCl3
NS                       2
DS                       0
SWH               8223.685 Hz
FIDRES            0.125483 Hz
AQ               3.9845889 sec
RG                     256
DW                  60.800 usec
DE                    6.00 usec
TE                   292.7 K
D1              1.00000000 sec
TD0                      1

======== CHANNEL f1 ========
NUC1                    1H
P1                   15.80 usec
PL1                  -1.00 dB
PL1W           12.17476940 W
SFO1           400.1324710 MHz

F2 - Processing parameters
SI                   32768
SF             400.1300096 MHz
WDW                     EM
SSB                      0
LB                    0.30 Hz
GB                       0
PC                    1.00
```

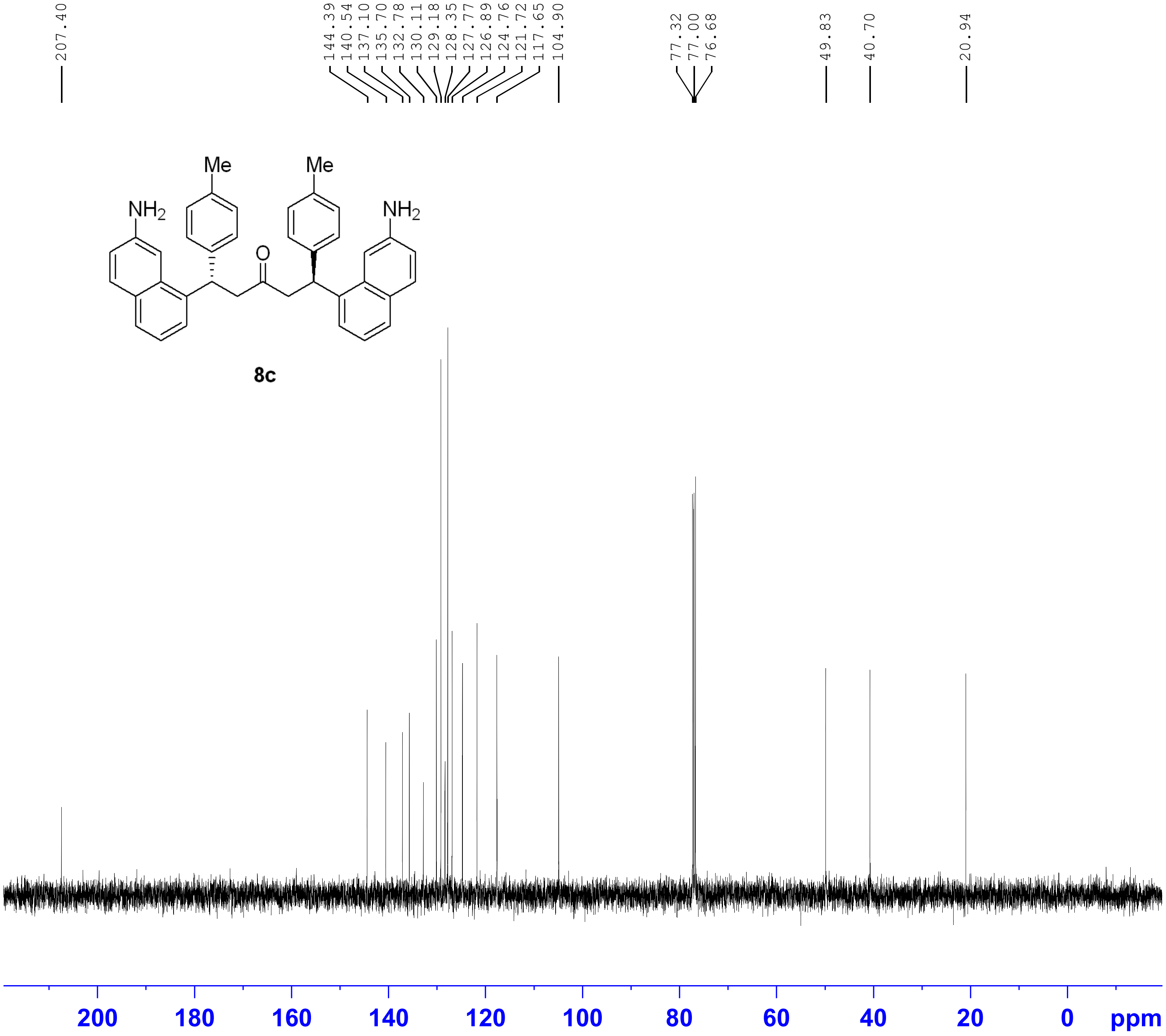

8c

Current Data Parameters
NAME zrh-9-51-1-c
EXPNO 1
PROCNO 1

F2 - Acquisition Parameters
Date_ 20230404
Time 19.22
INSTRUM spect
PROBHD 5 mm DUL 13C-1
PULPROG zgpg30
TD 65536
SOLVENT CDCl3
NS 32
DS 0
SWH 24038.461 Hz
FIDRES 0.366798 Hz
AQ 1.3631488 sec
RG 2050
DW 20.800 usec
DE 6.00 usec
TE 292.9 K
D1 2.00000000 sec
D11 0.03000000 sec
TD0 1

======== CHANNEL f1 ========
NUC1 13C
P1 40.00 usec
PL1 -3.00 dB
PL1W 60.64365387 W
SFO1 100.6228298 MHz

======== CHANNEL f2 ========
CPDPRG[2 waltz16
NUC2 1H
PCPD2 80.00 usec
PL2 -1.00 dB
PL12 14.39 dB
PL13 18.00 dB
PL2W 12.17476940 W
PL12W 0.35193357 W
PL13W 0.15327126 W
SFO2 400.1316005 MHz

F2 - Processing parameters
SI 32768
SF 100.6127779 MHz
WDW EM
SSB 0
LB 1.00 Hz
GB 0
PC 1.40

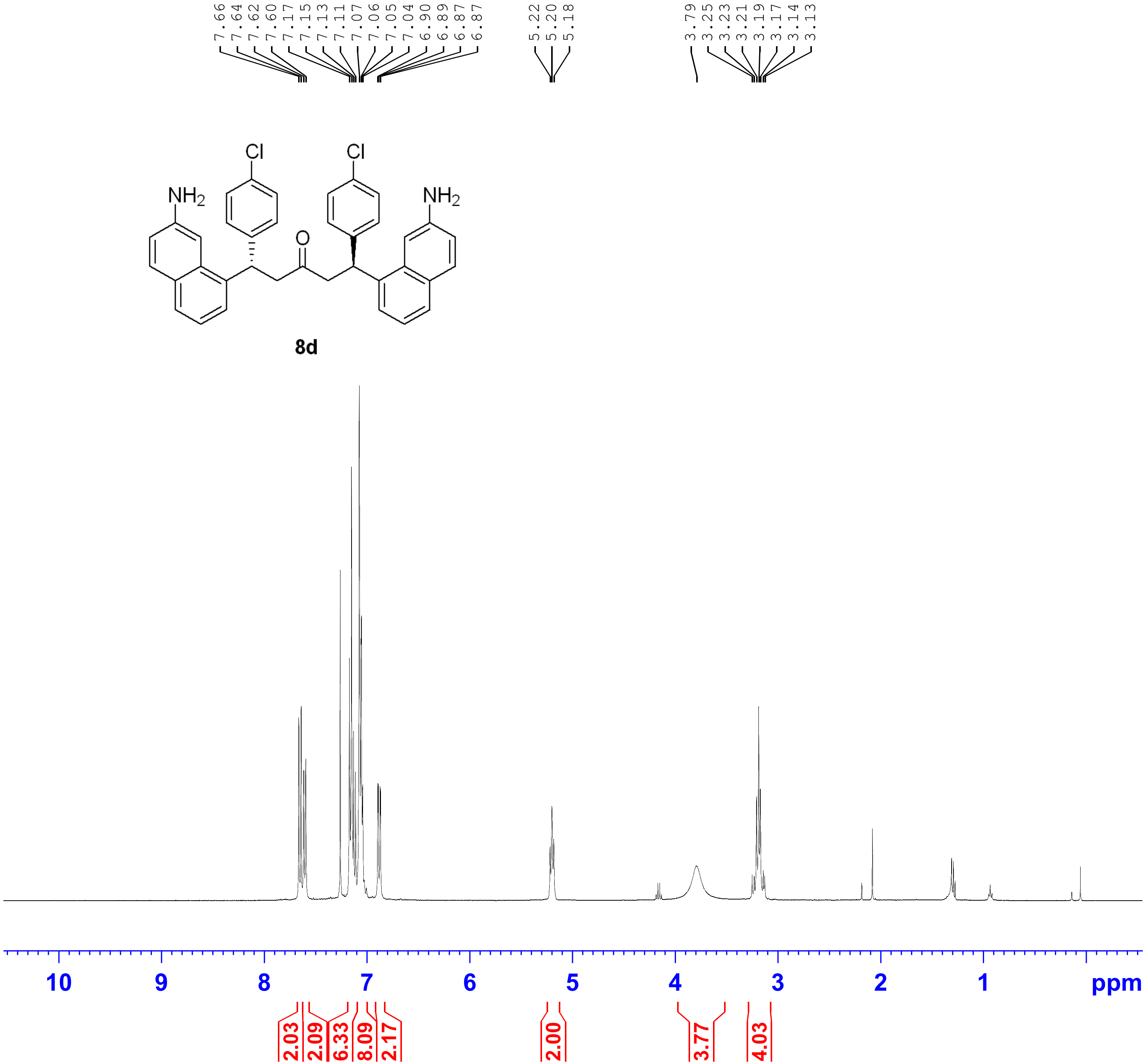

8d

```
Current Data Parameters
NAME       zrh-9-78-4Cl-h
EXPNO                   1
PROCNO                  1

F2 - Acquisition Parameters
Date_            20230427
Time                10.20
INSTRUM             spect
PROBHD     5 mm DUL 13C-1
PULPROG              zg30
TD                  65536
SOLVENT             CDCl3
NS                      2
DS                      0
SWH              8223.685 Hz
FIDRES           0.125483 Hz
AQ              3.9845889 sec
RG                    128
DW                 60.800 usec
DE                   6.00 usec
TE                  293.4 K
D1             1.00000000 sec
TD0                     1

======== CHANNEL f1 ========
NUC1                   1H
P1                  15.80 usec
PL1                 -1.00 dB
PL1W          12.17476940 W
SFO1          400.1324710 MHz

F2 - Processing parameters
SI                  32768
SF            400.1300096 MHz
WDW                    EM
SSB                     0
LB                   0.30 Hz
GB                      0
PC                   1.00
```

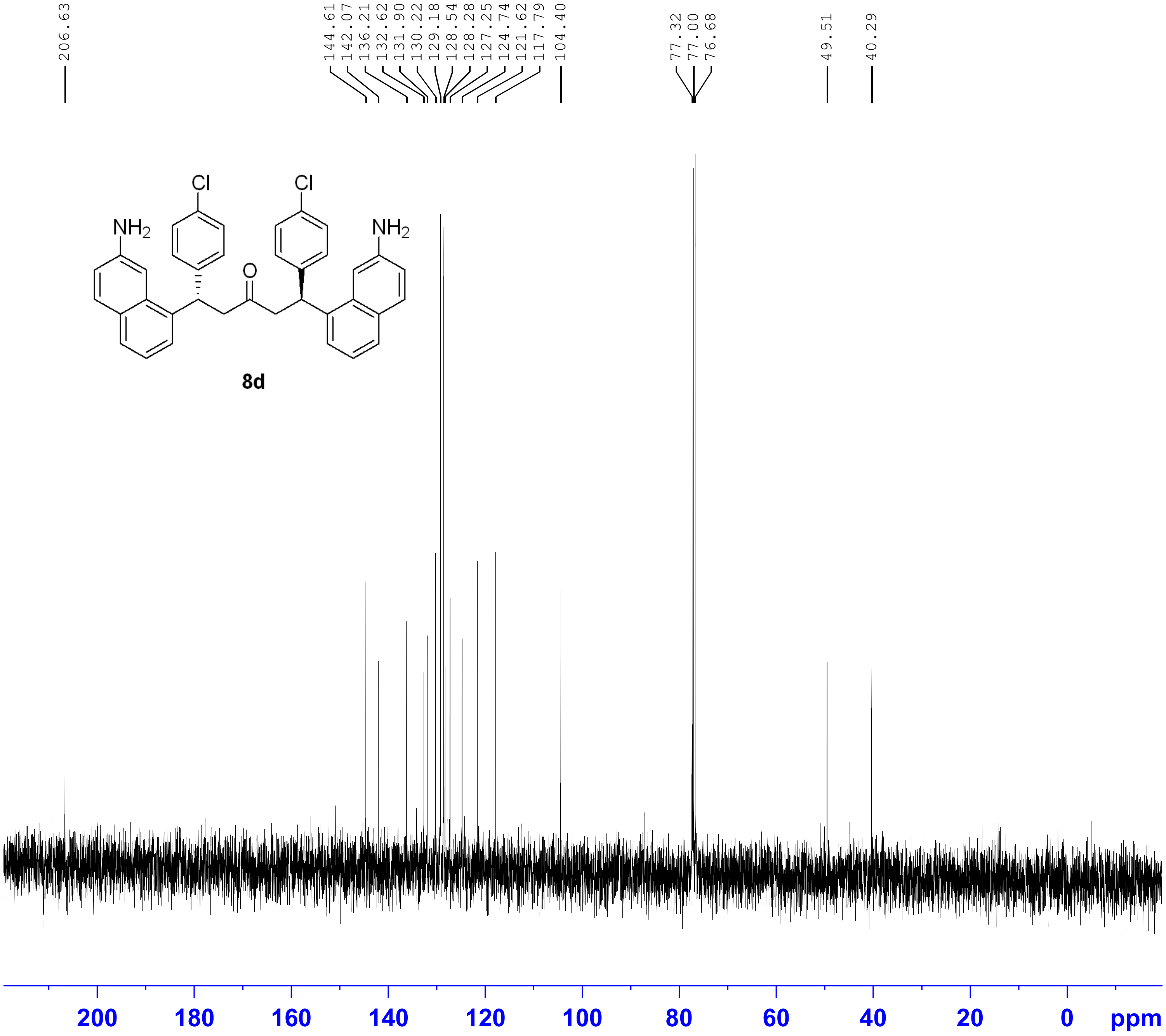

8d

Current Data Parameters
NAME zrh-9-78-4Cl-c
EXPNO 1
PROCNO 1

F2 - Acquisition Parameters
Date_ 20230427
Time 10.22
INSTRUM spect
PROBHD 5 mm DUL 13C-1
PULPROG zgpg30
TD 65536
SOLVENT CDCl3
NS 36
DS 0
SWH 24038.461 Hz
FIDRES 0.366798 Hz
AQ 1.3631488 sec
RG 101
DW 20.800 usec
DE 6.00 usec
TE 293.6 K
D1 2.00000000 sec
D11 0.03000000 sec
TD0 1

======== CHANNEL f1 ========
NUC1 13C
P1 40.00 usec
PL1 -3.00 dB
PL1W 60.64365387 W
SFO1 100.6228298 MHz

======== CHANNEL f2 ========
CPDPRG[2 waltz16
NUC2 1H
PCPD2 80.00 usec
PL2 -1.00 dB
PL12 14.39 dB
PL13 18.00 dB
PL2W 12.17476940 W
PL12W 0.35193357 W
PL13W 0.15327126 W
SFO2 400.1316005 MHz

F2 - Processing parameters
SI 32768
SF 100.6127843 MHz
WDW EM
SSB 0
LB 1.00 Hz
GB 0
PC 1.40

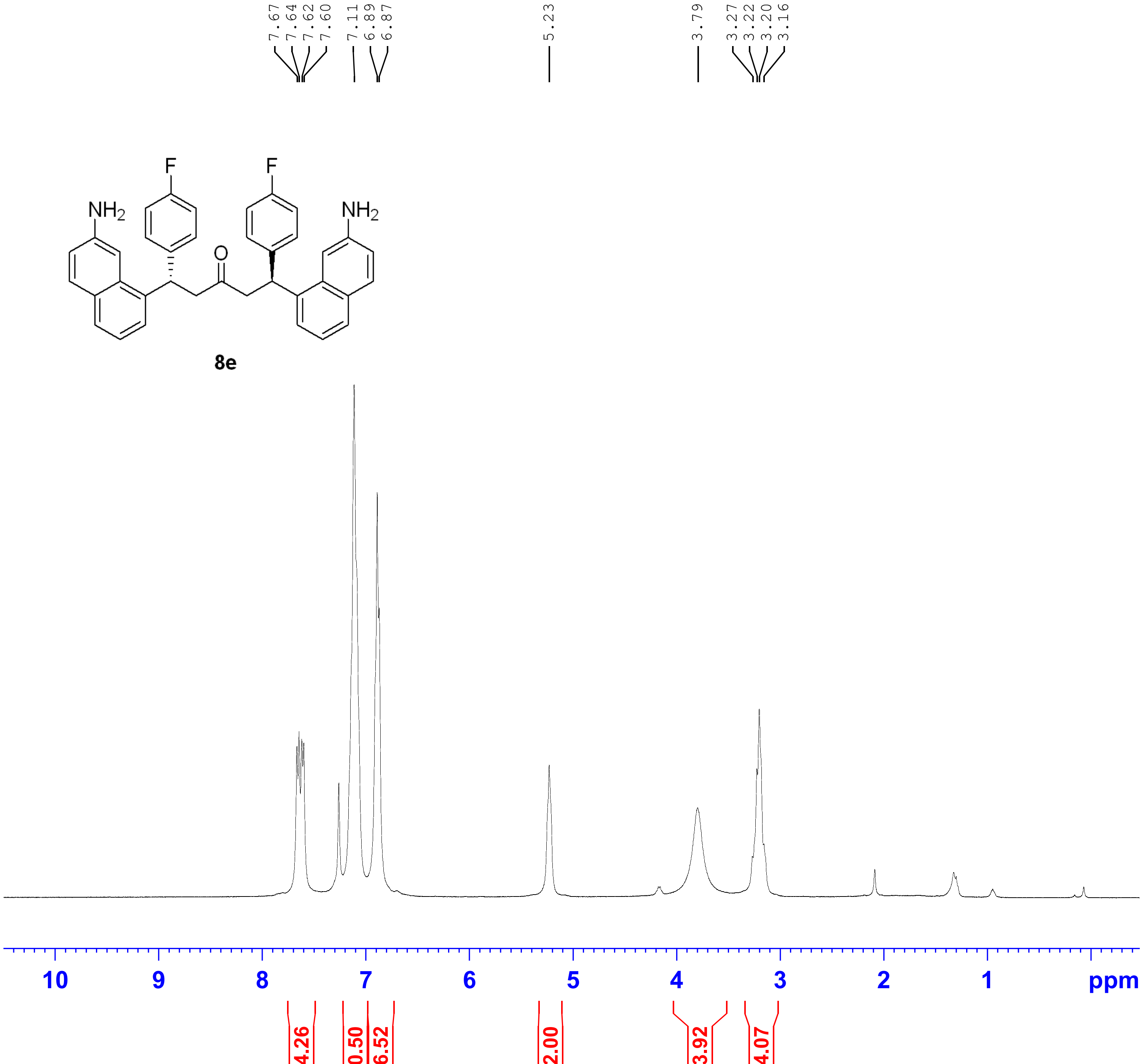

Current Data Parameters
NAME zrh-9-84-h
EXPNO 1
PROCNO 1

F2 - Acquisition Parameters
Date_ 20230428
Time 22.07
INSTRUM spect
PROBHD 5 mm PABBO BB/
PULPROG zg30
TD 65536
SOLVENT CDCl3
NS 4
DS 2
SWH 8012.820 Hz
FIDRES 0.122266 Hz
AQ 4.0894465 sec
RG 25.32
DW 62.400 usec
DE 6.50 usec
TE 296.3 K
D1 1.00000000 sec
TD0 1

======== CHANNEL f1 ========
SFO1 400.1324710 MHz
NUC1 1H
P1 14.50 usec
PLW1 11.99499989 W

F2 - Processing parameters
SI 65536
SF 400.1300108 MHz
WDW EM
SSB 0
LB 0.30 Hz
GB 0
PC 1.00

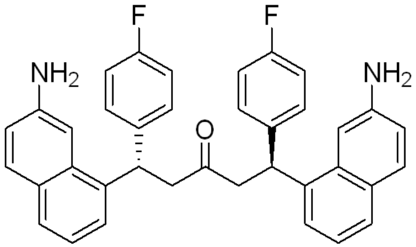

8e

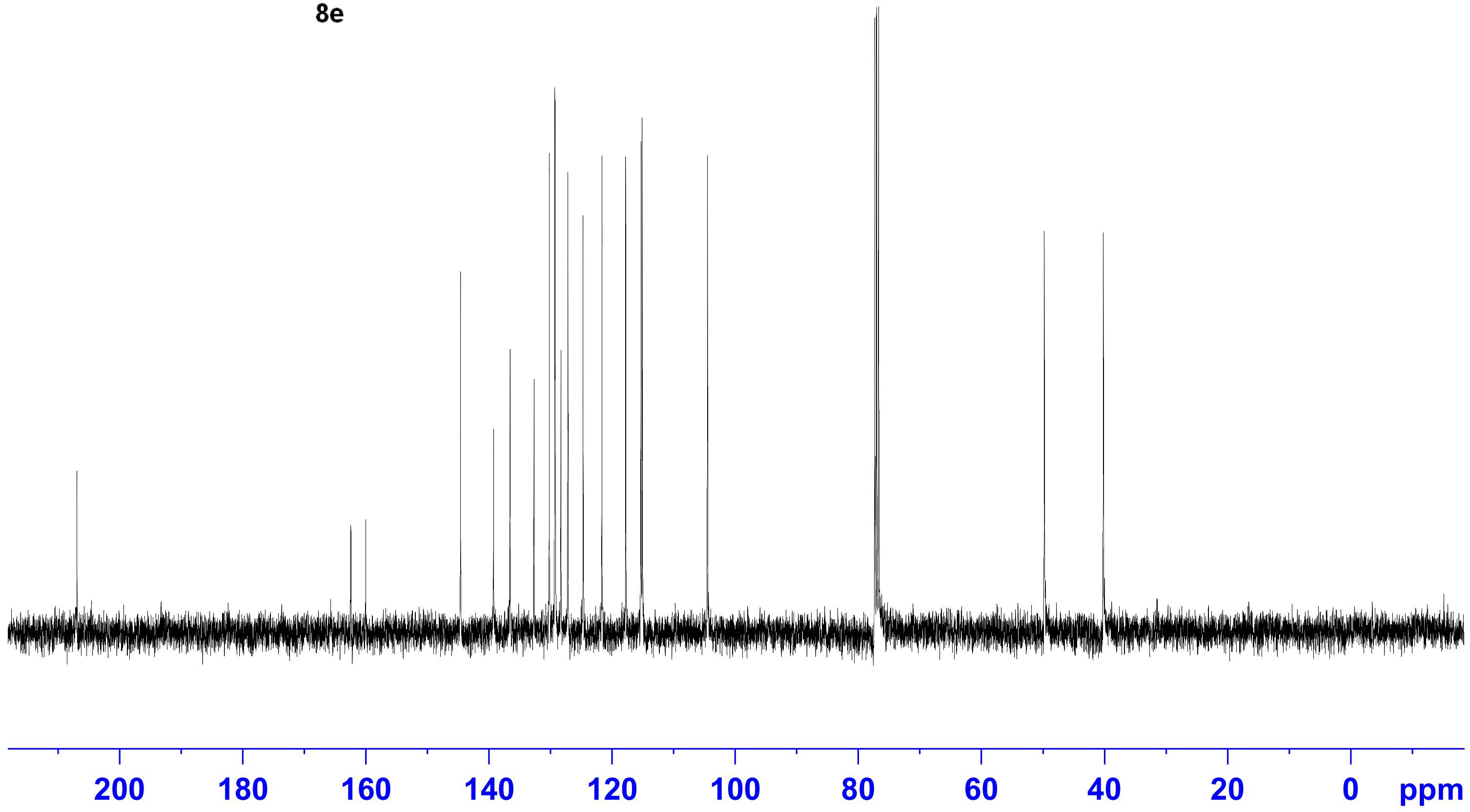

Current Data Parameters
NAME zrh-9-84-c
EXPNO 3
PROCNO 1

F2 - Acquisition Parameters
Date_ 20230428
Time 21.55 h
INSTRUM AvanceNeo 400MHz
PROBHD Z163739_0629 (
PULPROG zgpg30
TD 65536
SOLVENT CDCl3
NS 52
DS 4
SWH 23809.523 Hz
FIDRES 0.726609 Hz
AQ 1.3762560 sec
RG 10
DW 21.000 usec
DE 6.50 usec
TE 297.1 K
D1 2.00000000 sec
D11 0.03000000 sec
TD0 1
SFO1 100.6354036 MHz
NUC1 13C
P0 2.67 usec
P1 8.00 usec
PLW1 85.25399780 W
SFO2 400.1816007 MHz
NUC2 1H
CPDPRG[2 waltz65
PCPD2 90.00 usec
PLW2 21.26700020 W
PLW12 0.16802999 W
PLW13 0.08452000 W

F2 - Processing parameters
SI 32768
SF 100.6253579 MHz
WDW EM
SSB 0
LB 1.00 Hz
GB 0
PC 1.40

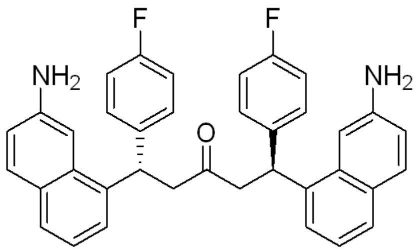

8e

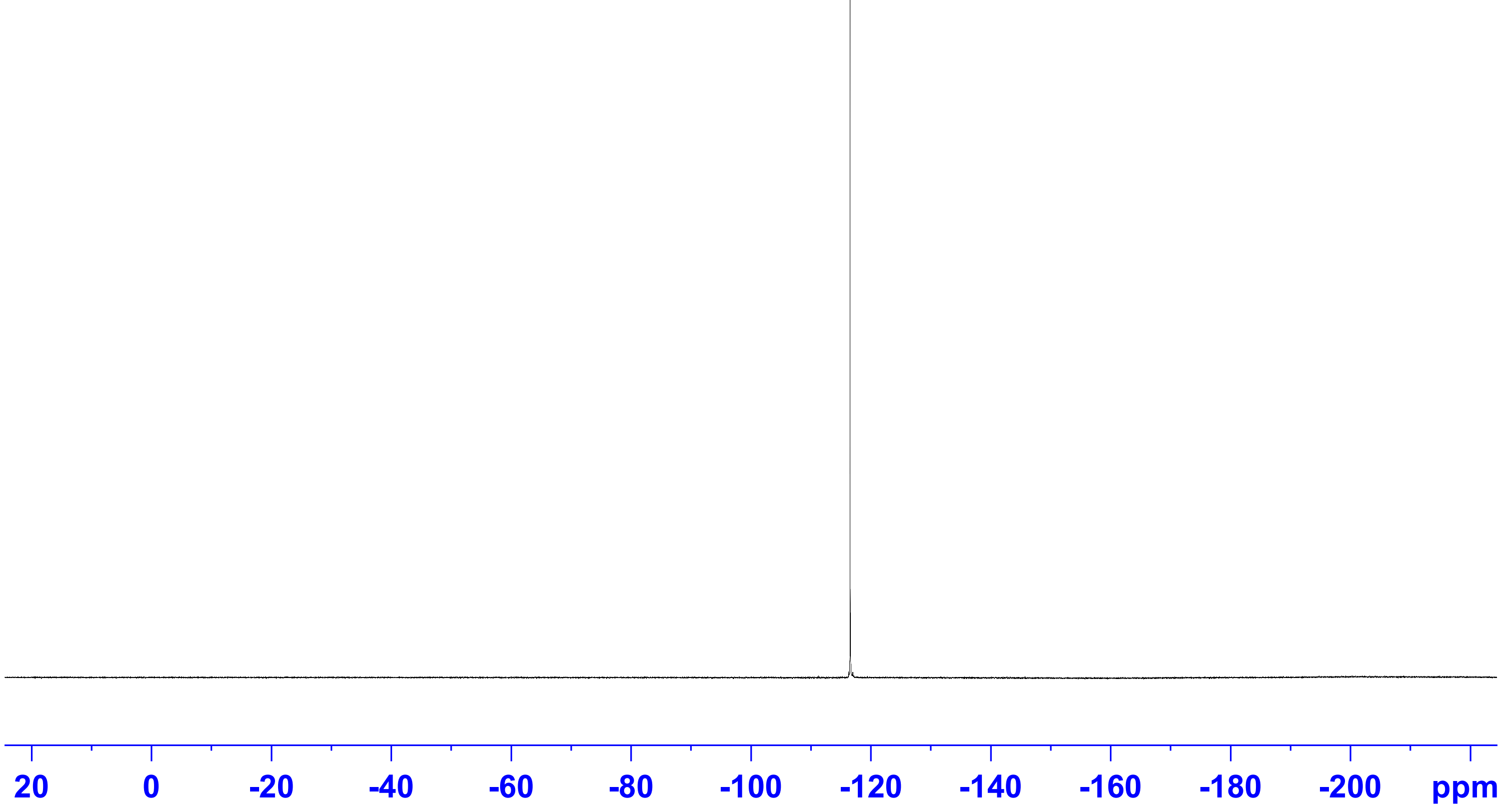

Current Data Parameters
NAME zrh-9-84-f
EXPNO 1
PROCNO 1

F2 - Acquisition Parameters
Date_ 20230428
Time 22.09
INSTRUM spect
PROBHD 5 mm PABBO BB/
PULPROG zgpg30
TD 65536
SOLVENT CDCl3
NS 2
DS 2
SWH 93750.000 Hz
FIDRES 1.430511 Hz
AQ 0.3495253 sec
RG 196.92
DW 5.333 usec
DE 6.50 usec
TE 296.4 K
D1 2.00000000 sec
D11 0.03000000 sec
TD0 1

======== CHANNEL f1 ========
SFO1 376.4607162 MHz
NUC1 19F
P1 14.70 usec
PLW1 15.99600029 W

======== CHANNEL f2 ========
SFO2 400.1316005 MHz
NUC2 1H
CPDPRG[2 waltz16
PCPD2 90.00 usec
PLW2 11.99499989 W
PLW12 0.34213999 W
PLW13 0.27713001 W

F2 - Processing parameters
SI 32768
SF 376.4983660 MHz
WDW EM
SSB 0
LB 1.00 Hz
GB 0
PC 1.40

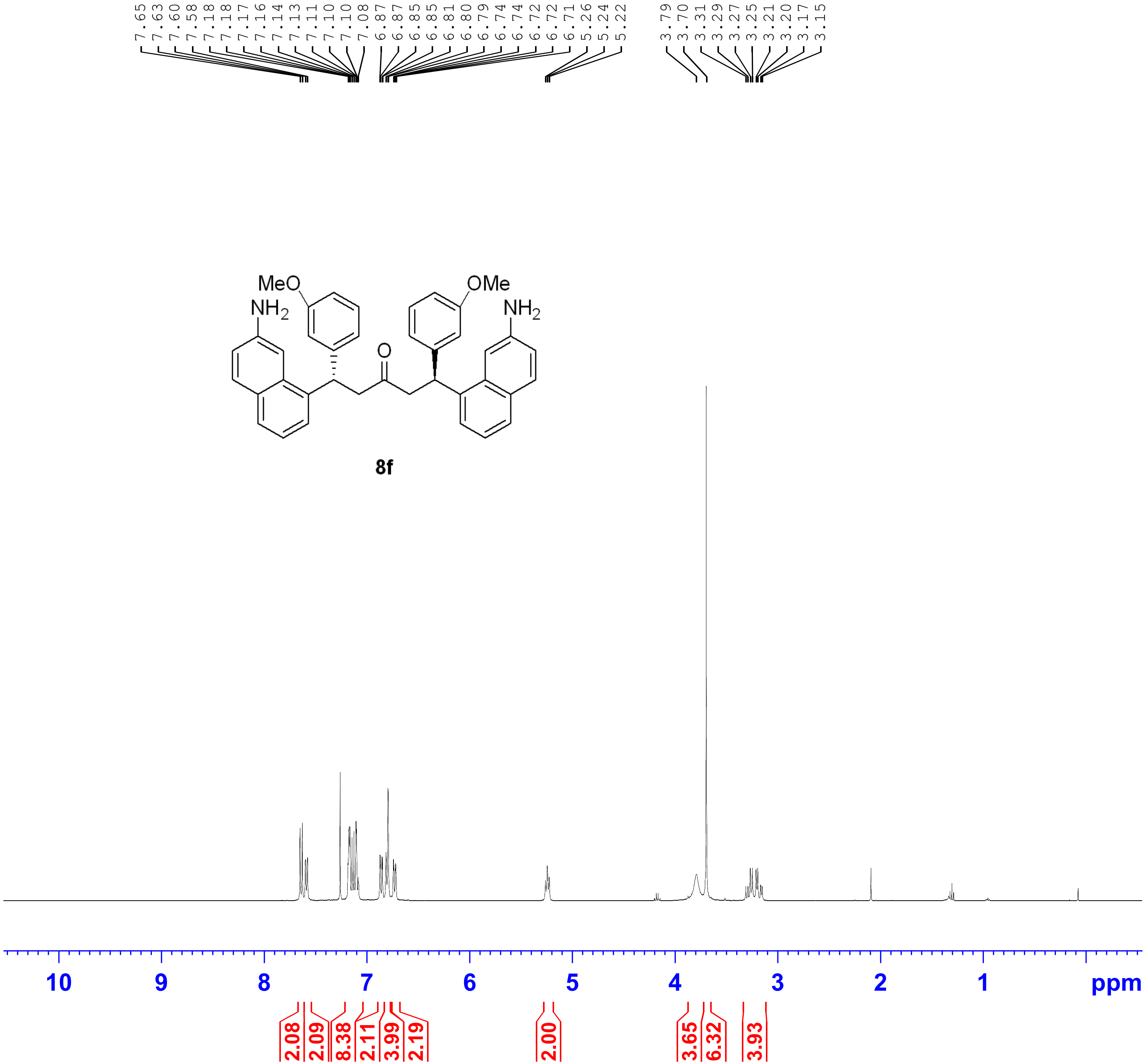

8f


```
Current Data Parameters
NAME      zrh-9-80-3OMe-h
EXPNO                   1
PROCNO                  1

F2 - Acquisition Parameters
Date_            20230427
Time                10.33
INSTRUM             spect
PROBHD   5 mm DUL 13C-1
PULPROG              zg30
TD                  65536
SOLVENT             CDCl3
NS                      6
DS                      0
SWH              8223.685 Hz
FIDRES           0.125483 Hz
AQ              3.9845889 sec
RG                     64
DW                 60.800 usec
DE                   6.00 usec
TE                  293.4 K
D1             1.00000000 sec
TD0                     1

======== CHANNEL f1 ========
NUC1                   1H
P1                  15.80 usec
PL1                 -1.00 dB
PL1W          12.17476940 W
SFO1          400.1324710 MHz

F2 - Processing parameters
SI                  32768
SF            400.1300094 MHz
WDW                    EM
SSB                     0
LB                   0.30 Hz
GB                      0
PC                   1.00
```

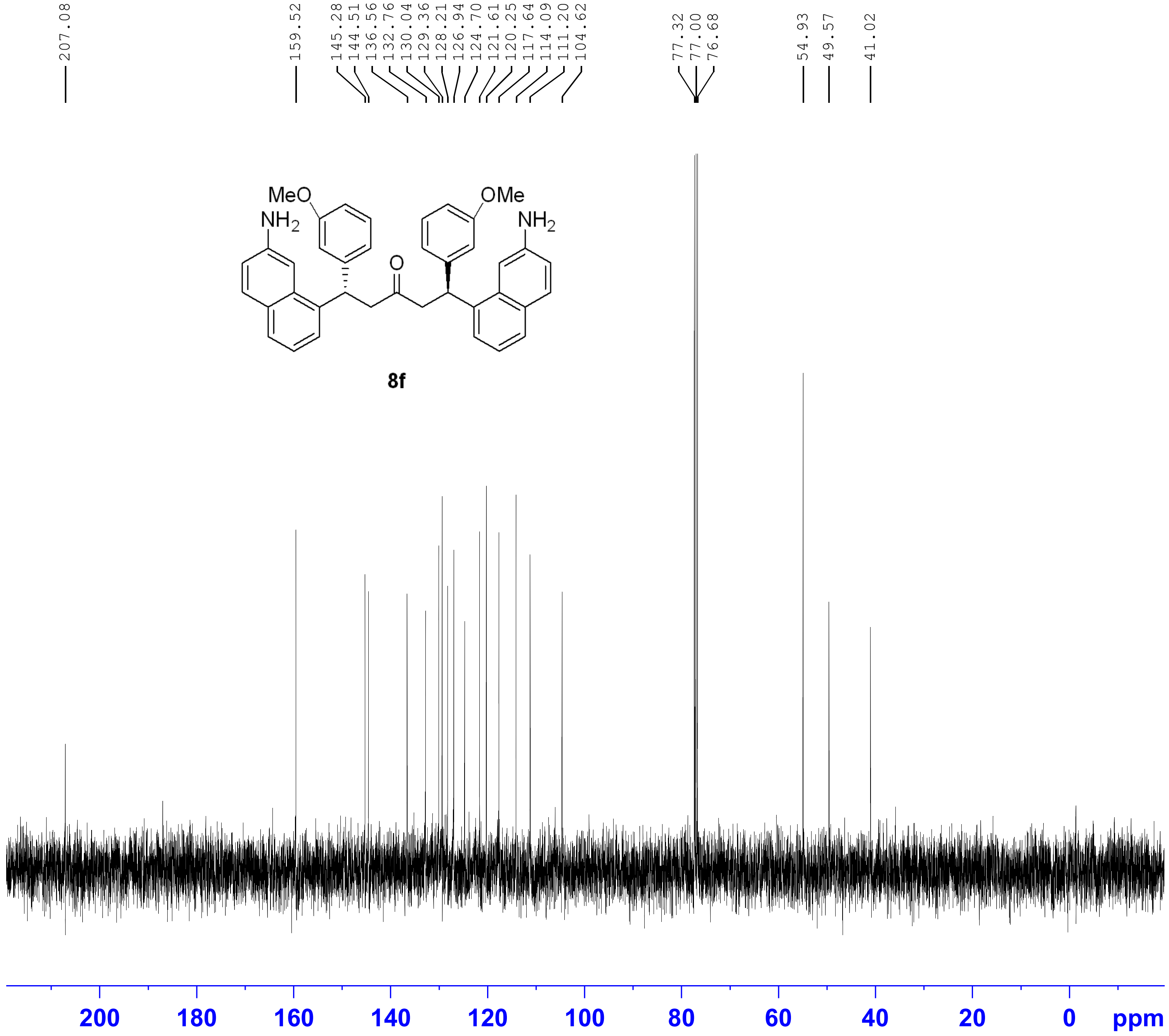


```
Current Data Parameters
NAME      zrh-9-80-3OMe-c
EXPNO                   1
PROCNO                  1

F2 - Acquisition Parameters
Date_            20230427
Time                10.36
INSTRUM             spect
PROBHD    5 mm DUL 13C-1
PULPROG           zgpg30
TD                  65536
SOLVENT             CDCl3
NS                     35
DS                      0
SWH             24038.461 Hz
FIDRES           0.366798 Hz
AQ              1.3631488 sec
RG                   71.8
DW                 20.800 usec
DE                   6.00 usec
TE                  293.6 K
D1             2.00000000 sec
D11            0.03000000 sec
TD0                     1

======== CHANNEL f1 ========
NUC1                  13C
P1                  40.00 usec
PL1                 -3.00 dB
PL1W          60.64365387 W
SFO1         100.6228298 MHz

======== CHANNEL f2 ========
CPDPRG[2          waltz16
NUC2                   1H
PCPD2               80.00 usec
PL2                 -1.00 dB
PL12                14.39 dB
PL13                18.00 dB
PL2W          12.17476940 W
PL12W          0.35193357 W
PL13W          0.15327126 W
SFO2         400.1316005 MHz

F2 - Processing parameters
SI                  32768
SF           100.6127885 MHz
WDW                    EM
SSB                     0
LB                   1.00 Hz
GB                      0
PC                   1.40
```

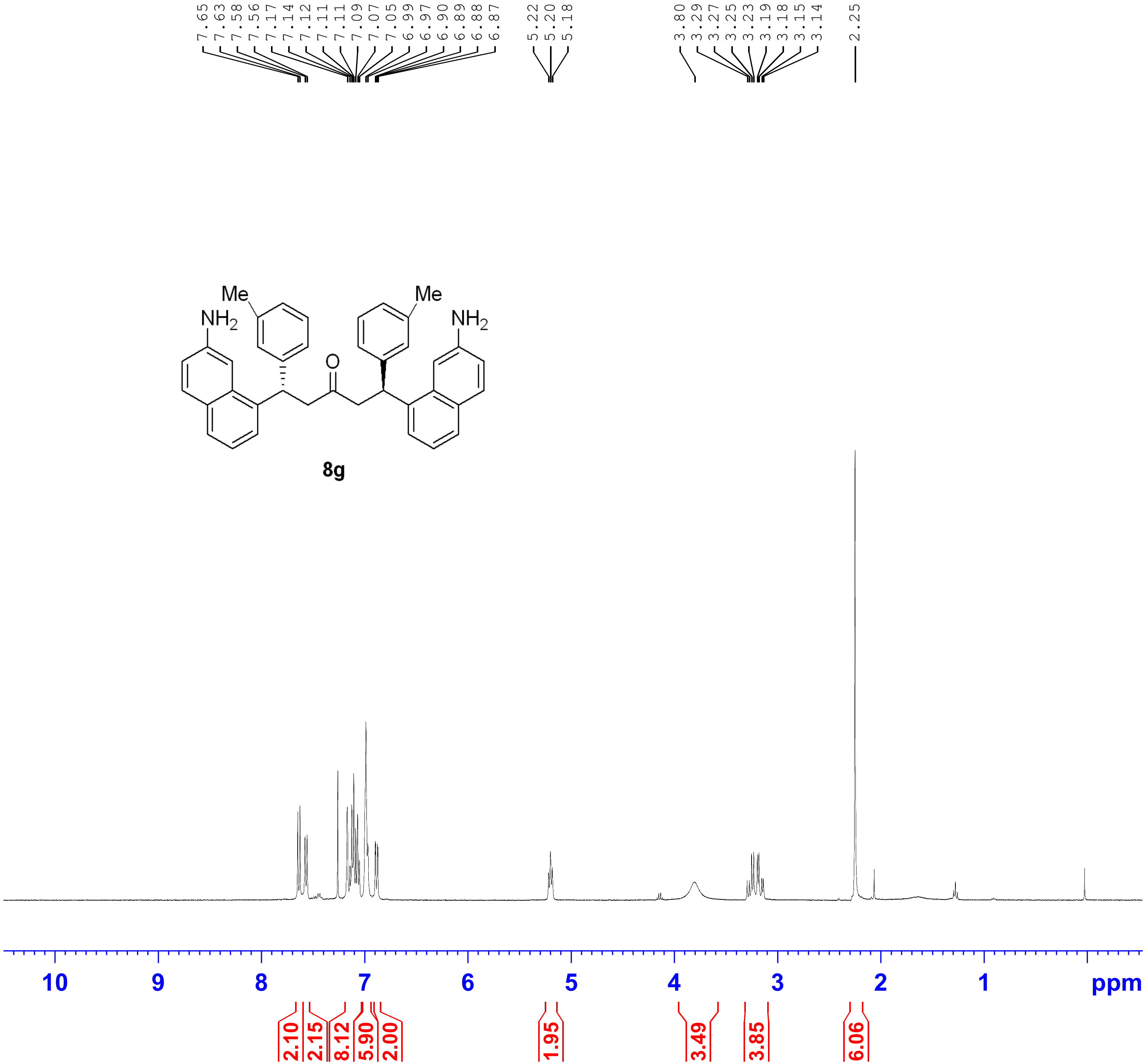

8g

```
Current Data Parameters
NAME          zrh-9-42-re-h
EXPNO                     1
PROCNO                    1

F2 - Acquisition Parameters
Date_              20230330
Time                  19.02
INSTRUM               spect
PROBHD    5 mm DUL 13C-1
PULPROG                zg30
TD                    65536
SOLVENT               CDCl3
NS                        1
DS                        0
SWH                8223.685 Hz
FIDRES             0.125483 Hz
AQ                3.9845889 sec
RG                      256
DW                   60.800 usec
DE                     6.00 usec
TE                    292.7 K
D1               1.00000000 sec
TD0                       1

======== CHANNEL f1 ========
NUC1                     1H
P1                    15.80 usec
PL1                   -1.00 dB
PL1W            12.17476940 W
SFO1            400.1324710 MHz

F2 - Processing parameters
SI                    32768
SF              400.1300098 MHz
WDW                      EM
SSB                       0
LB                     0.30 Hz
GB                        0
PC                     1.00
```

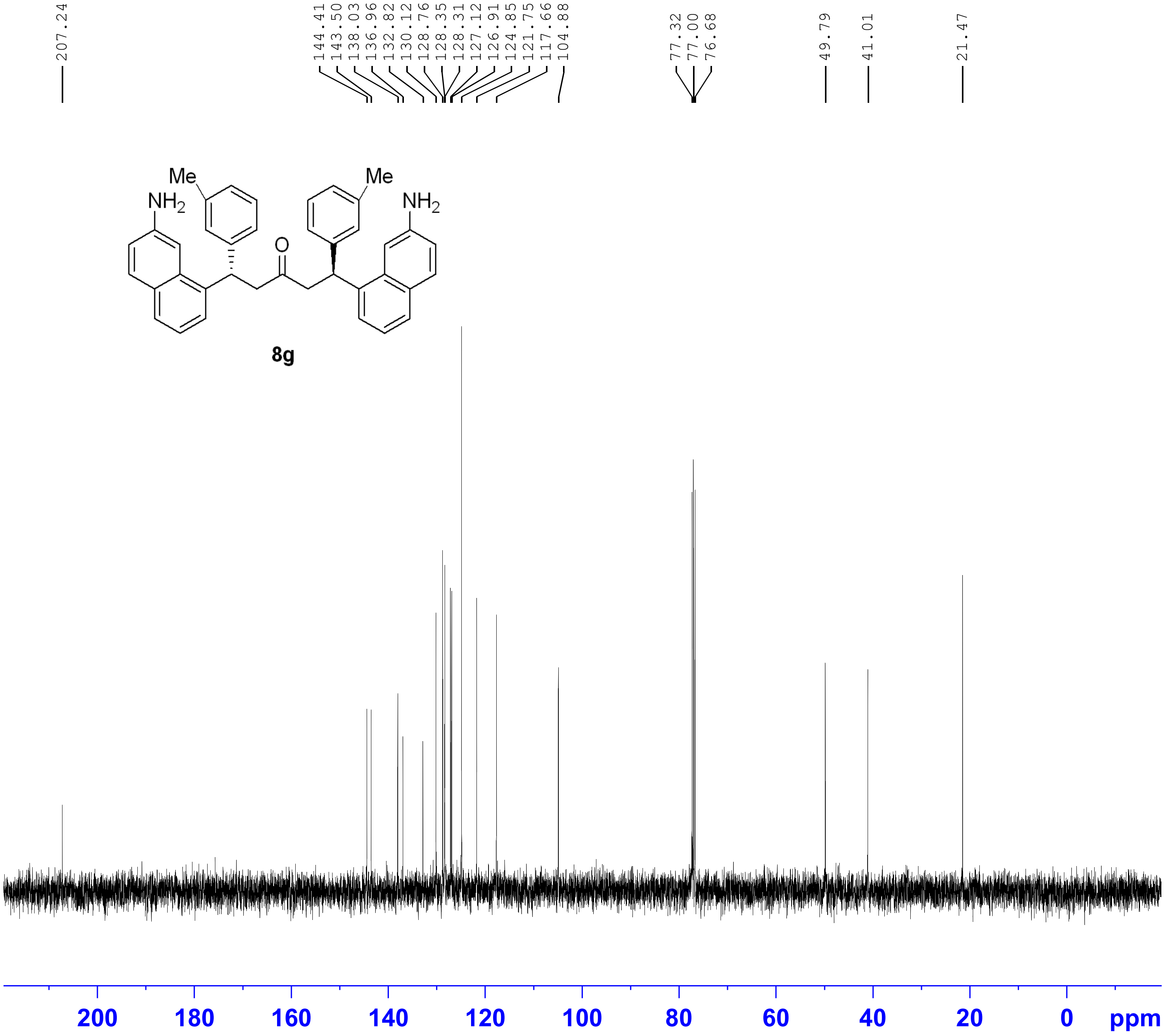

Current Data Parameters
NAME zrh-9-42-re-c
EXPNO 1
PROCNO 1

F2 - Acquisition Parameters
Date_ 20230330
Time 19.03
INSTRUM spect
PROBHD 5 mm DUL 13C-1
PULPROG zgpg30
TD 65536
SOLVENT CDCl3
NS 39
DS 0
SWH 24038.461 Hz
FIDRES 0.366798 Hz
AQ 1.3631488 sec
RG 2050
DW 20.800 usec
DE 6.00 usec
TE 292.7 K
D1 2.00000000 sec
D11 0.03000000 sec
TD0 1

======== CHANNEL f1 ========
NUC1 13C
P1 40.00 usec
PL1 -3.00 dB
PL1W 60.64365387 W
SFO1 100.6228298 MHz

======== CHANNEL f2 ========
CPDPRG[2 waltz16
NUC2 1H
PCPD2 80.00 usec
PL2 -1.00 dB
PL12 14.39 dB
PL13 18.00 dB
PL2W 12.17476940 W
PL12W 0.35193357 W
PL13W 0.15327126 W
SFO2 400.1316005 MHz

F2 - Processing parameters
SI 32768
SF 100.6127773 MHz
WDW EM
SSB 0
LB 1.00 Hz
GB 0
PC 1.40

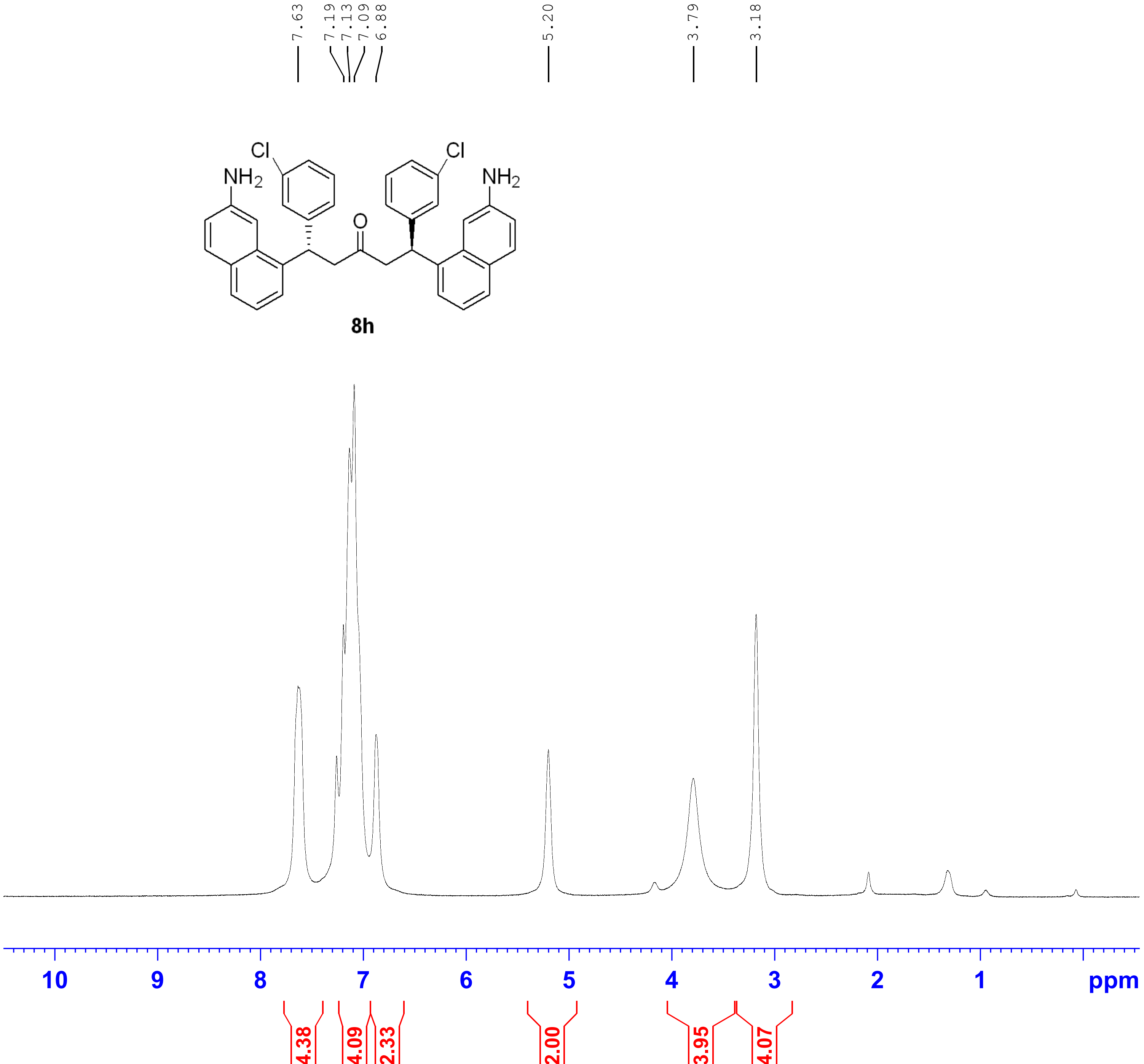

8h

Current Data Parameters
NAME zrh-9-99-h
EXPNO 1
PROCNO 1

F2 - Acquisition Parameters
Date_ 20230508
Time 21.35
INSTRUM spect
PROBHD 5 mm DUL 13C-1
PULPROG zg30
TD 65536
SOLVENT CDCl3
NS 2
DS 0
SWH 8223.685 Hz
FIDRES 0.125483 Hz
AQ 3.9845889 sec
RG 114
DW 60.800 usec
DE 6.00 usec
TE 293.6 K
D1 1.00000000 sec
TD0 1

======== CHANNEL f1 ========
NUC1 1H
P1 15.80 usec
PL1 -1.00 dB
PL1W 12.17476940 W
SFO1 400.1324710 MHz

F2 - Processing parameters
SI 32768
SF 400.1300104 MHz
WDW EM
SSB 0
LB 0.30 Hz
GB 0
PC 1.00

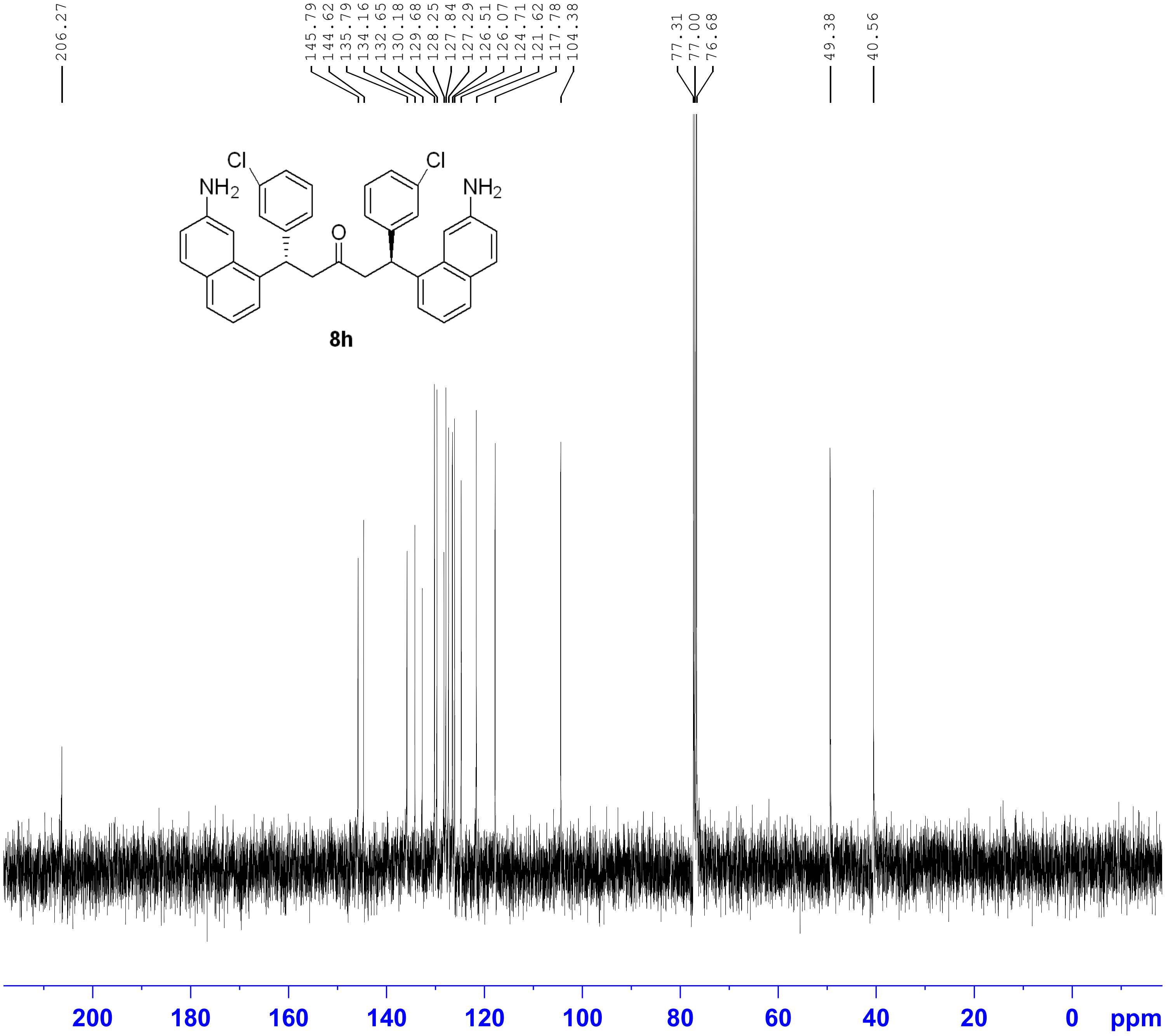

Current Data Parameters
NAME zrh-9-99-c
EXPNO 1
PROCNO 1

F2 - Acquisition Parameters
Date_ 20230508
Time 21.36
INSTRUM spect
PROBHD 5 mm DUL 13C-1
PULPROG zgpg30
TD 65536
SOLVENT CDCl3
NS 196
DS 0
SWH 24038.461 Hz
FIDRES 0.366798 Hz
AQ 1.3631488 sec
RG 114
DW 20.800 usec
DE 6.00 usec
TE 293.7 K
D1 2.00000000 sec
D11 0.03000000 sec
TD0 1

======== CHANNEL f1 ========
NUC1 13C
P1 40.00 usec
PL1 -3.00 dB
PL1W 60.64365387 W
SFO1 100.6228298 MHz

======== CHANNEL f2 ========
CPDPRG[2 waltz16
NUC2 1H
PCPD2 80.00 usec
PL2 -1.00 dB
PL12 14.39 dB
PL13 18.00 dB
PL2W 12.17476940 W
PL12W 0.35193357 W
PL13W 0.15327126 W
SFO2 400.1316005 MHz

F2 - Processing parameters
SI 32768
SF 100.6127875 MHz
WDW EM
SSB 0
LB 1.00 Hz
GB 0
PC 1.40

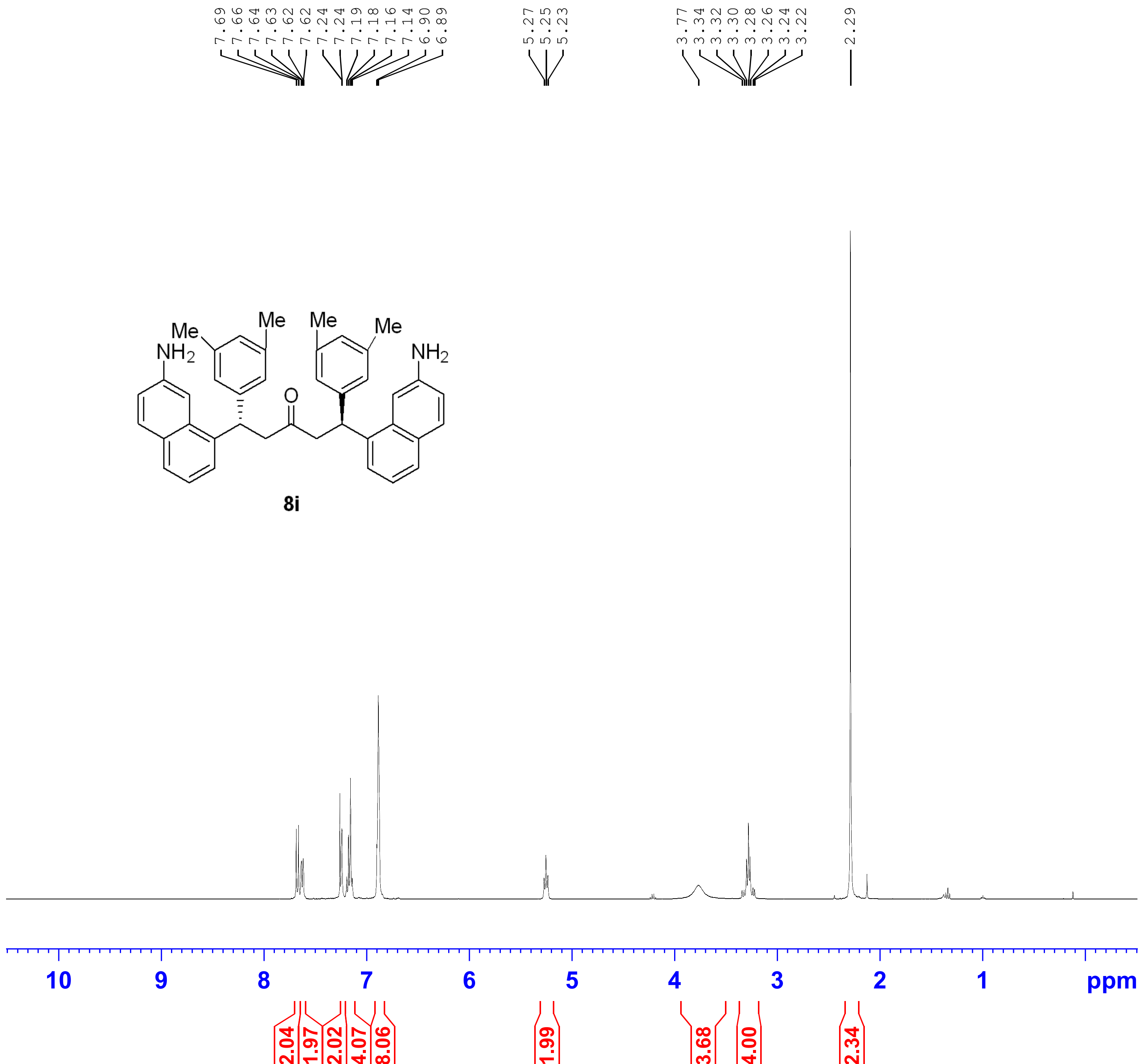

8i

Current Data Parameters
NAME zrh-9-97-3,5Me-h
EXPNO 1
PROCNO 1

F2 - Acquisition Parameters
Date_ 20230427
Time 10.26
INSTRUM spect
PROBHD 5 mm DUL 13C-1
PULPROG zg30
TD 65536
SOLVENT CDCl3
NS 2
DS 0
SWH 8223.685 Hz
FIDRES 0.125483 Hz
AQ 3.9845889 sec
RG 64
DW 60.800 usec
DE 6.00 usec
TE 293.4 K
D1 1.00000000 sec
TD0 1

======== CHANNEL f1 ========
NUC1 1H
P1 15.80 usec
PL1 -1.00 dB
PL1W 12.17476940 W
SFO1 400.1324710 MHz

F2 - Processing parameters
SI 32768
SF 400.1300093 MHz
WDW EM
SSB 0
LB 0.30 Hz
GB 0
PC 1.00

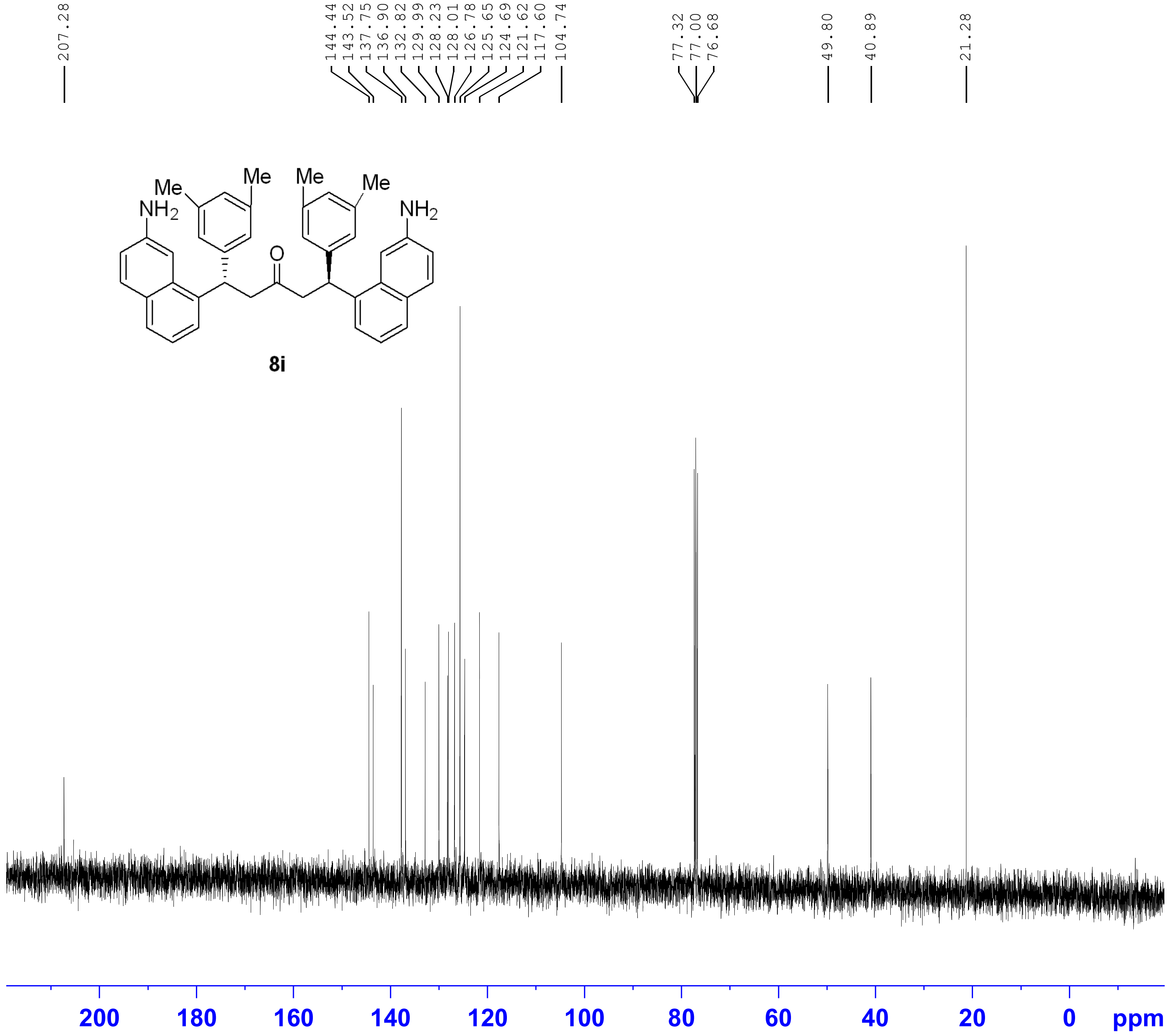

Current Data Parameters	
NAME	zrh-9-97-3,5Me-c
EXPNO	1
PROCNO	1

F2 - Acquisition Parameters	
Date_	20230427
Time	10.29
INSTRUM	spect
PROBHD	5 mm DUL 13C-1
PULPROG	zgpg30
TD	65536
SOLVENT	CDCl3
NS	40
DS	0
SWH	24038.461 Hz
FIDRES	0.366798 Hz
AQ	1.3631488 sec
RG	71.8
DW	20.800 usec
DE	6.00 usec
TE	293.7 K
D1	2.00000000 sec
D11	0.03000000 sec
TD0	1

======== CHANNEL f1 ========	
NUC1	13C
P1	40.00 usec
PL1	-3.00 dB
PL1W	60.64365387 W
SFO1	100.6228298 MHz

======== CHANNEL f2 ========	
CPDPRG[2	waltz16
NUC2	1H
PCPD2	80.00 usec
PL2	-1.00 dB
PL12	14.39 dB
PL13	18.00 dB
PL2W	12.17476940 W
PL12W	0.35193357 W
PL13W	0.15327126 W
SFO2	400.1316005 MHz

F2 - Processing parameters	
SI	32768
SF	100.6127934 MHz
WDW	EM
SSB	0
LB	1.00 Hz
GB	0
PC	1.40

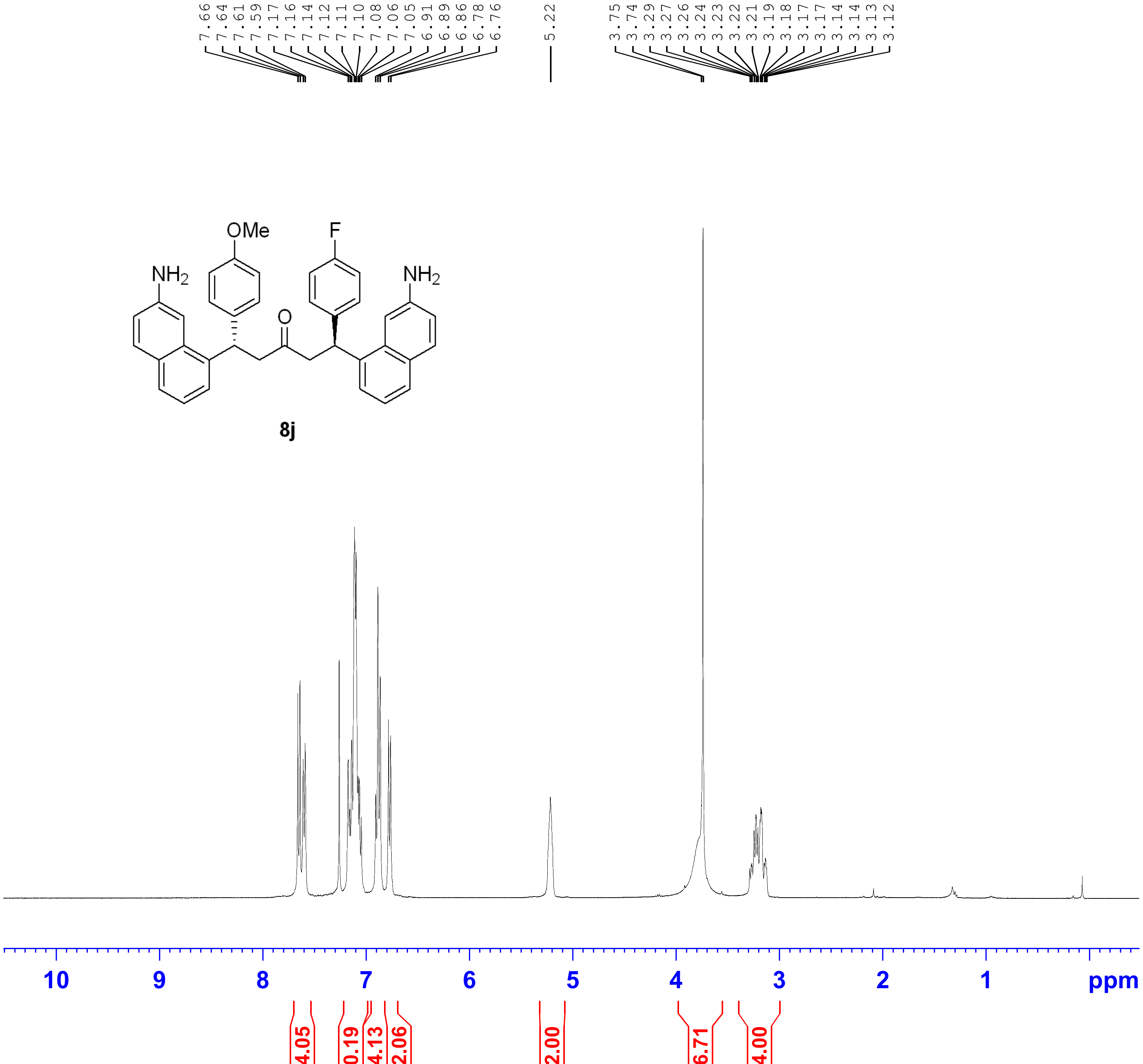

8j

```
Current Data Parameters
NAME            zrh-9-88-h
EXPNO                    1
PROCNO                   1

F2 - Acquisition Parameters
Date_             20230502
Time                 19.41
INSTRUM              spect
PROBHD     5 mm PABBO BB/
PULPROG               zg30
TD                   65536
SOLVENT              CDCl3
NS                       2
DS                       2
SWH               8012.820 Hz
FIDRES            0.122266 Hz
AQ               4.0894465 sec
RG                   25.32
DW                  62.400 usec
DE                    6.50 usec
TE                   296.2 K
D1              1.00000000 sec
TD0                      1

======== CHANNEL f1 ========
SFO1           400.1324710 MHz
NUC1                    1H
P1                   14.50 usec
PLW1           11.99499989 W

F2 - Processing parameters
SI                   65536
SF             400.1300100 MHz
WDW                     EM
SSB                      0
LB                    0.30 Hz
GB                       0
PC                    1.00
```

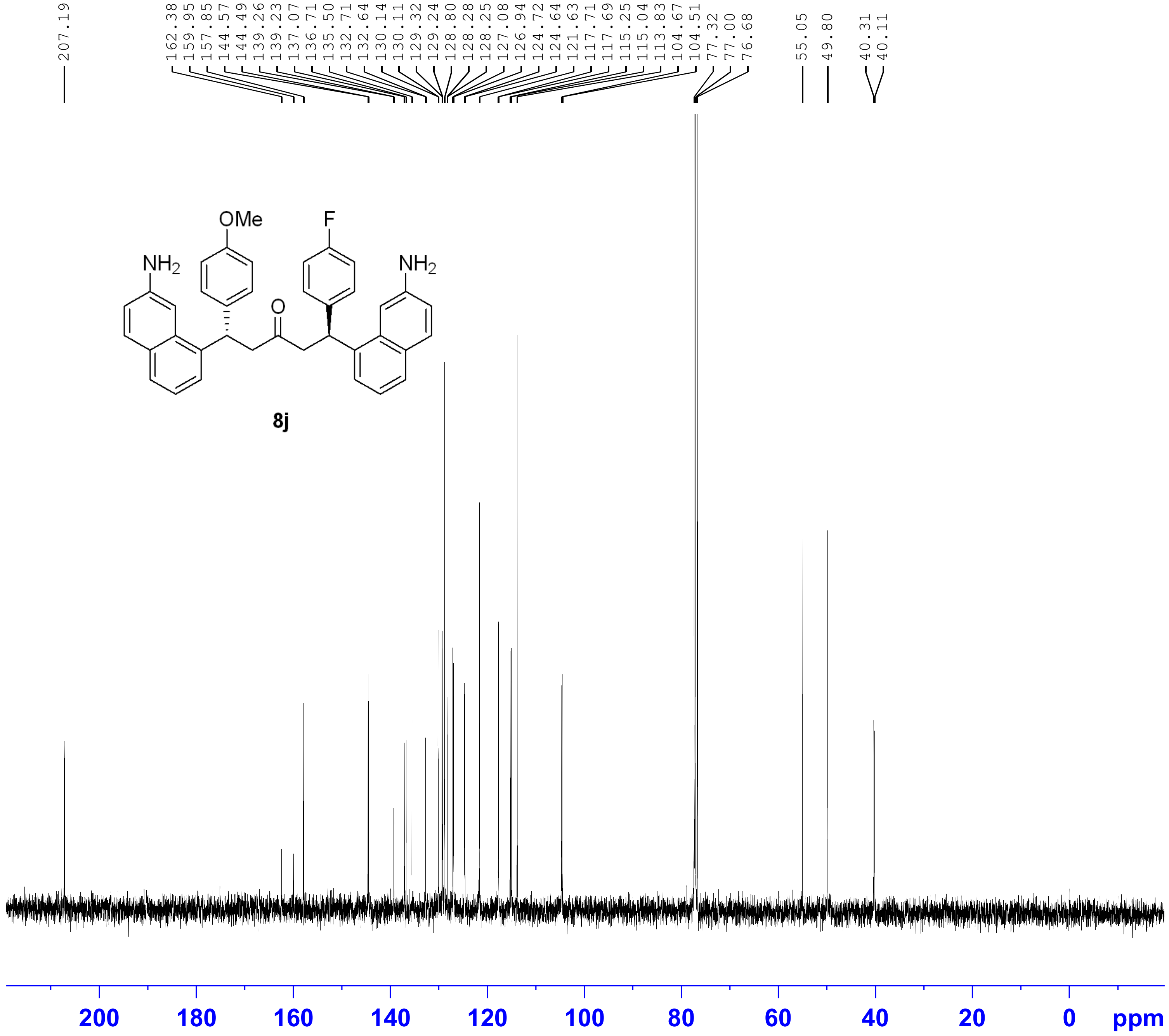

8j

Current Data Parameters
NAME zrh-9-88-c
EXPNO 1
PROCNO 1

F2 - Acquisition Parameters
Date_ 20230502
Time 19.43
INSTRUM spect
PROBHD 5 mm PABBO BB/
PULPROG zgpg30
TD 65536
SOLVENT CDCl3
NS 50
DS 2
SWH 24038.461 Hz
FIDRES 0.366798 Hz
AQ 1.3631488 sec
RG 196.92
DW 20.800 usec
DE 6.50 usec
TE 296.6 K
D1 2.00000000 sec
D11 0.03000000 sec
TD0 1

======== CHANNEL f1 ========
SFO1 100.6228298 MHz
NUC1 13C
P1 9.70 usec
PLW1 46.98899841 W

======== CHANNEL f2 ========
SFO2 400.1316005 MHz
NUC2 1H
CPDPRG[2 waltz16
PCPD2 90.00 usec
PLW2 11.99499989 W
PLW12 0.34213999 W
PLW13 0.27713001 W

F2 - Processing parameters
SI 32768
SF 100.6127861 MHz
WDW EM
SSB 0
LB 1.00 Hz
GB 0
PC 1.40

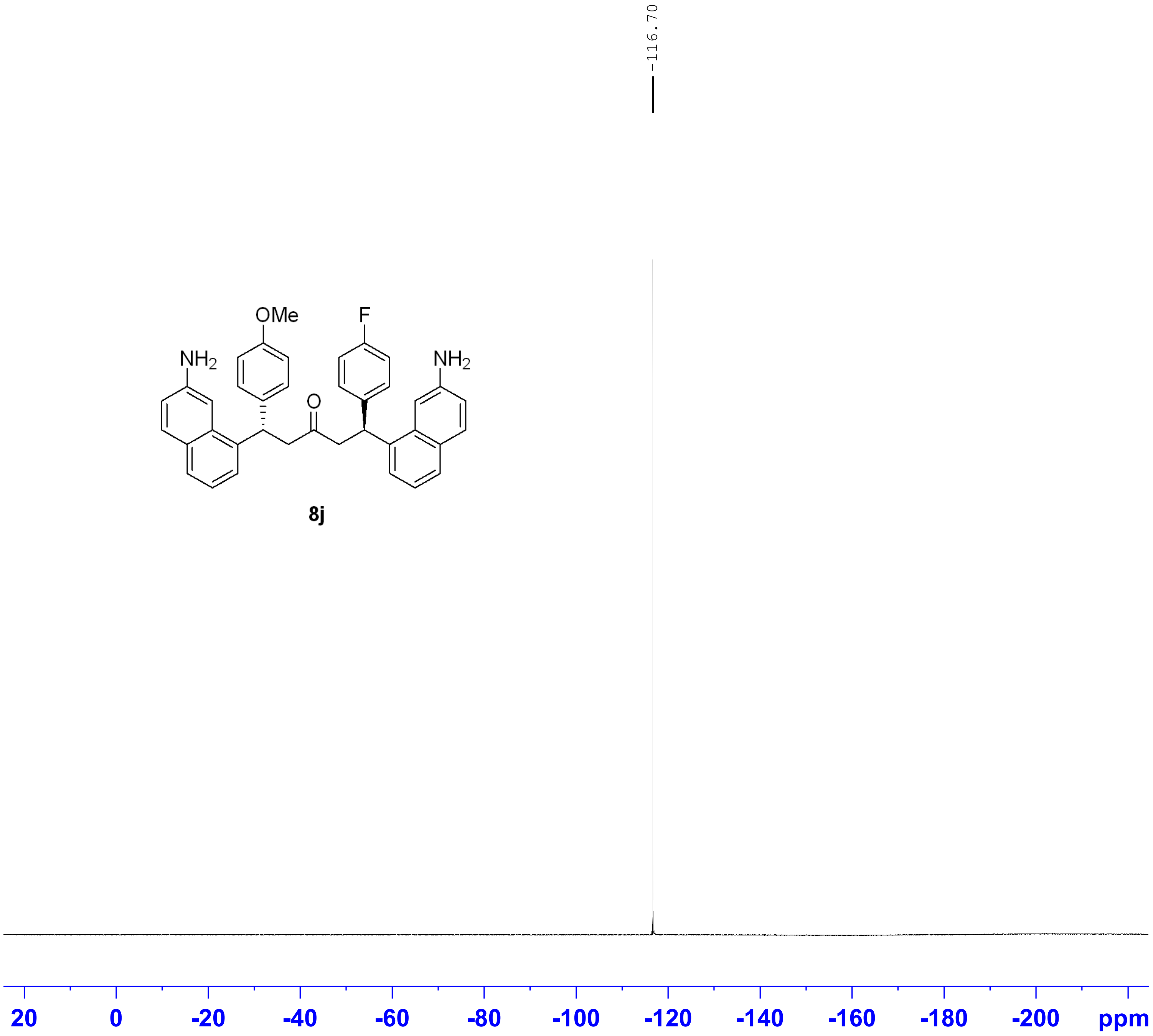

8j

```
Current Data Parameters
NAME             zrh-9-88-f
EXPNO                     1
PROCNO                    1

F2 - Acquisition Parameters
Date_              20230502
Time                  19.46
INSTRUM               spect
PROBHD    5 mm PABBO BB/
PULPROG             zgpg30
TD                    65536
SOLVENT               CDCl3
NS                        4
DS                        2
SWH               93750.000 Hz
FIDRES             1.430511 Hz
AQ                0.3495253 sec
RG                   196.92
DW                    5.333 usec
DE                     6.50 usec
TE                    296.5 K
D1               2.00000000 sec
D11              0.03000000 sec
TD0                       1

======== CHANNEL f1 ========
SFO1            376.4607162 MHz
NUC1                    19F
P1                    14.70 usec
PLW1            15.99600029 W

======== CHANNEL f2 ========
SFO2            400.1316005 MHz
NUC2                     1H
CPDPRG[2            waltz16
PCPD2                 90.00 usec
PLW2            11.99499989 W
PLW12            0.34213999 W
PLW13            0.27713001 W

F2 - Processing parameters
SI                    32768
SF              376.4983660 MHz
WDW                      EM
SSB                       0
LB                     1.00 Hz
GB                        0
PC                     1.40
```

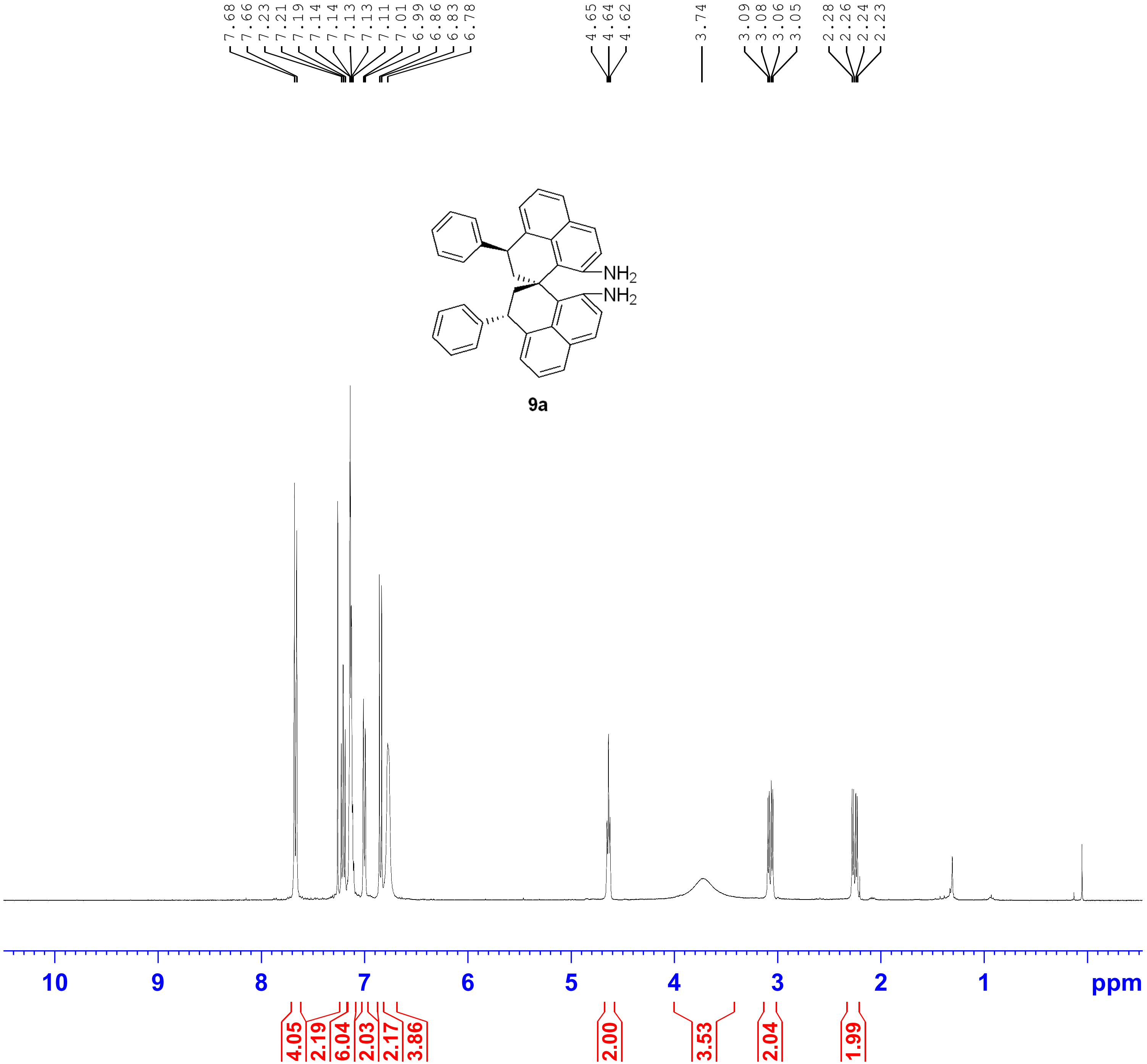

9a

Current Data Parameters		
NAME	zrh-9-11-h	
EXPNO	1	
PROCNO	1	
F2 - Acquisition Parameters		
Date_	20230329	
Time	18.35	
INSTRUM	spect	
PROBHD	5 mm PABBO BB/	
PULPROG	zg30	
TD	65536	
SOLVENT	CDCl3	
NS	4	
DS	2	
SWH	8012.820	Hz
FIDRES	0.122266	Hz
AQ	4.0894465	sec
RG	31.55	
DW	62.400	usec
DE	6.50	usec
TE	295.8	K
D1	1.00000000	sec
TD0	1	
======== CHANNEL f1 ========		
SFO1	400.1324710	MHz
NUC1	1H	
P1	14.50	usec
PLW1	11.99499989	W
F2 - Processing parameters		
SI	65536	
SF	400.1300100	MHz
WDW	EM	
SSB	0	
LB	0.30	Hz
GB	0	
PC	1.00	

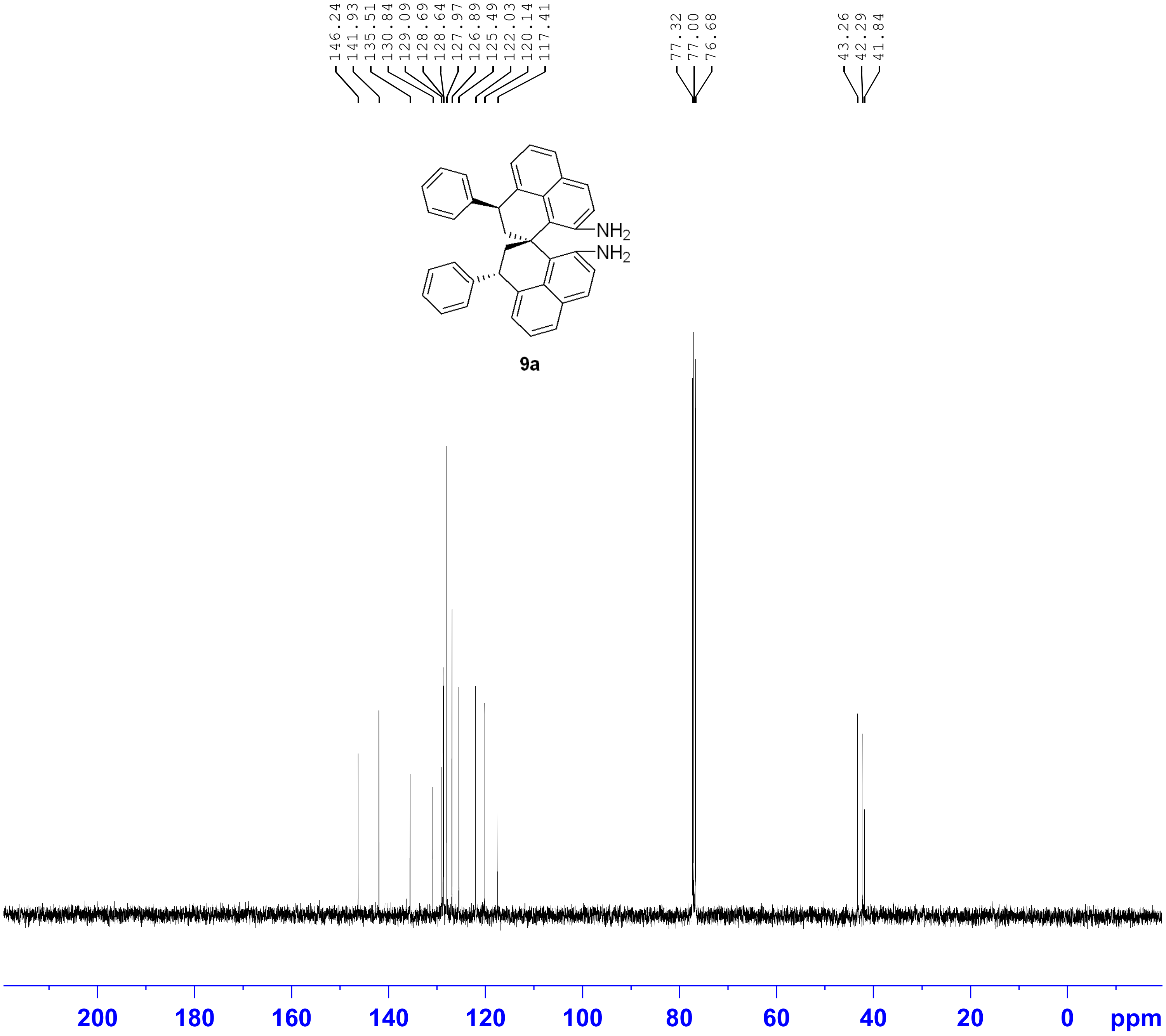


```
Current Data Parameters
NAME            zrh-9-11-c
EXPNO                    1
PROCNO                   1

F2 - Acquisition Parameters
Date_             20230329
Time                 18.37
INSTRUM              spect
PROBHD    5 mm PABBO BB/
PULPROG            zgpg30
TD                   65536
SOLVENT              CDCl3
NS                      35
DS                       2
SWH             24038.461 Hz
FIDRES           0.366798 Hz
AQ              1.3631488 sec
RG                  196.92
DW                  20.800 usec
DE                    6.50 usec
TE                   296.1 K
D1              2.00000000 sec
D11             0.03000000 sec
TD0                      1

======== CHANNEL f1 ========
SFO1          100.6228298 MHz
NUC1                   13C
P1                    9.70 usec
PLW1           46.98899841 W

======== CHANNEL f2 ========
SFO2          400.1316005 MHz
NUC2                    1H
CPDPRG[2           waltz16
PCPD2                90.00 usec
PLW2           11.99499989 W
PLW12           0.34213999 W
PLW13           0.27713001 W

F2 - Processing parameters
SI                   32768
SF            100.6127788 MHz
WDW                     EM
SSB                      0
LB                    1.00 Hz
GB                       0
PC                    1.40
```

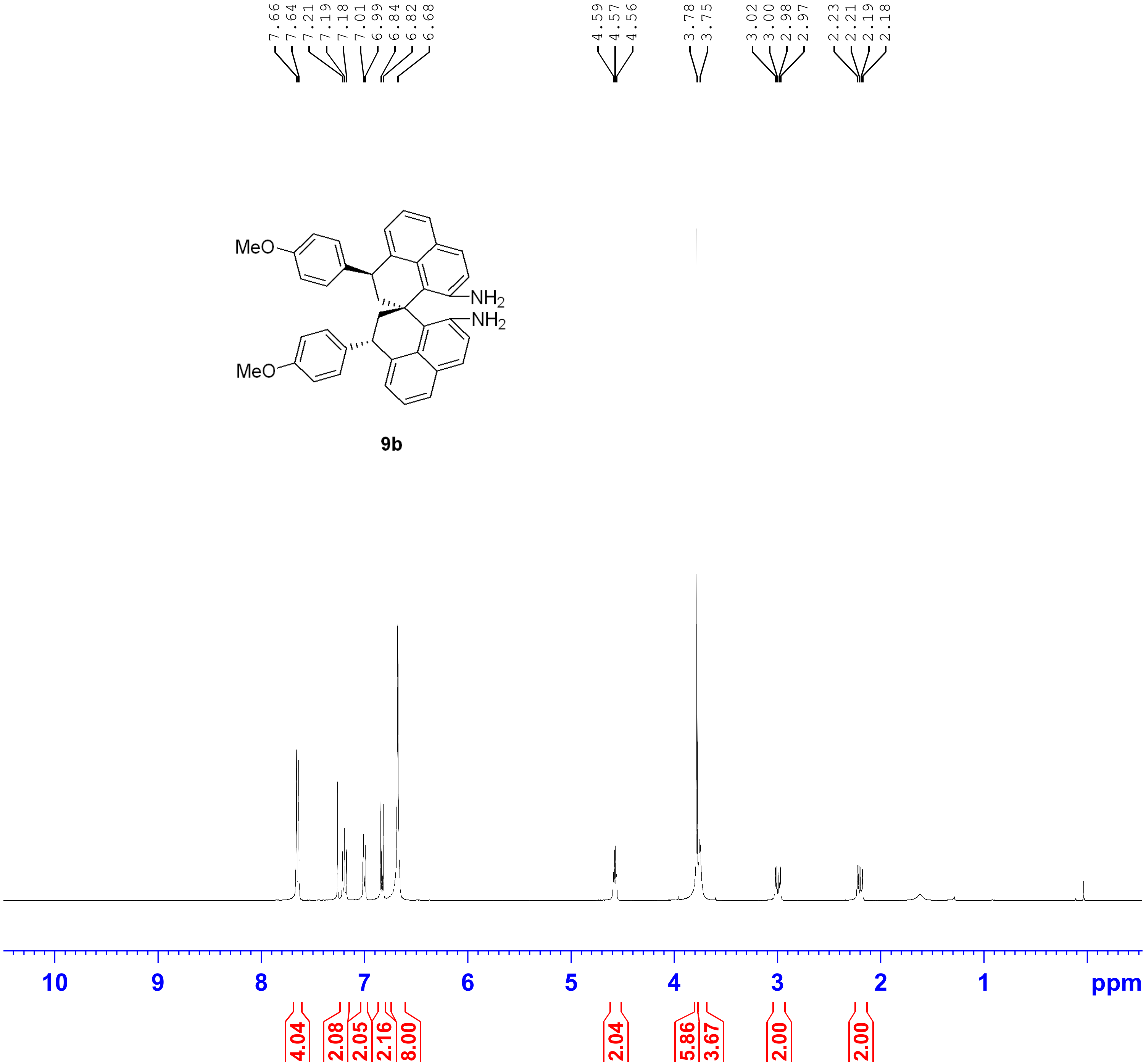

9b

Current Data Parameters
NAME zrh-9-47-2-h
EXPNO 1
PROCNO 1

F2 - Acquisition Parameters
Date_ 20230401
Time 19.27
INSTRUM spect
PROBHD 5 mm PABBO BB/
PULPROG zg30
TD 65536
SOLVENT CDCl3
NS 2
DS 2
SWH 8012.820 Hz
FIDRES 0.122266 Hz
AQ 4.0894465 sec
RG 34.77
DW 62.400 usec
DE 6.50 usec
TE 296.2 K
D1 1.00000000 sec
TD0 1

======== CHANNEL f1 ========
SFO1 400.1324710 MHz
NUC1 1H
P1 14.50 usec
PLW1 11.99499989 W

F2 - Processing parameters
SI 65536
SF 400.1300100 MHz
WDW EM
SSB 0
LB 0.30 Hz
GB 0
PC 1.00

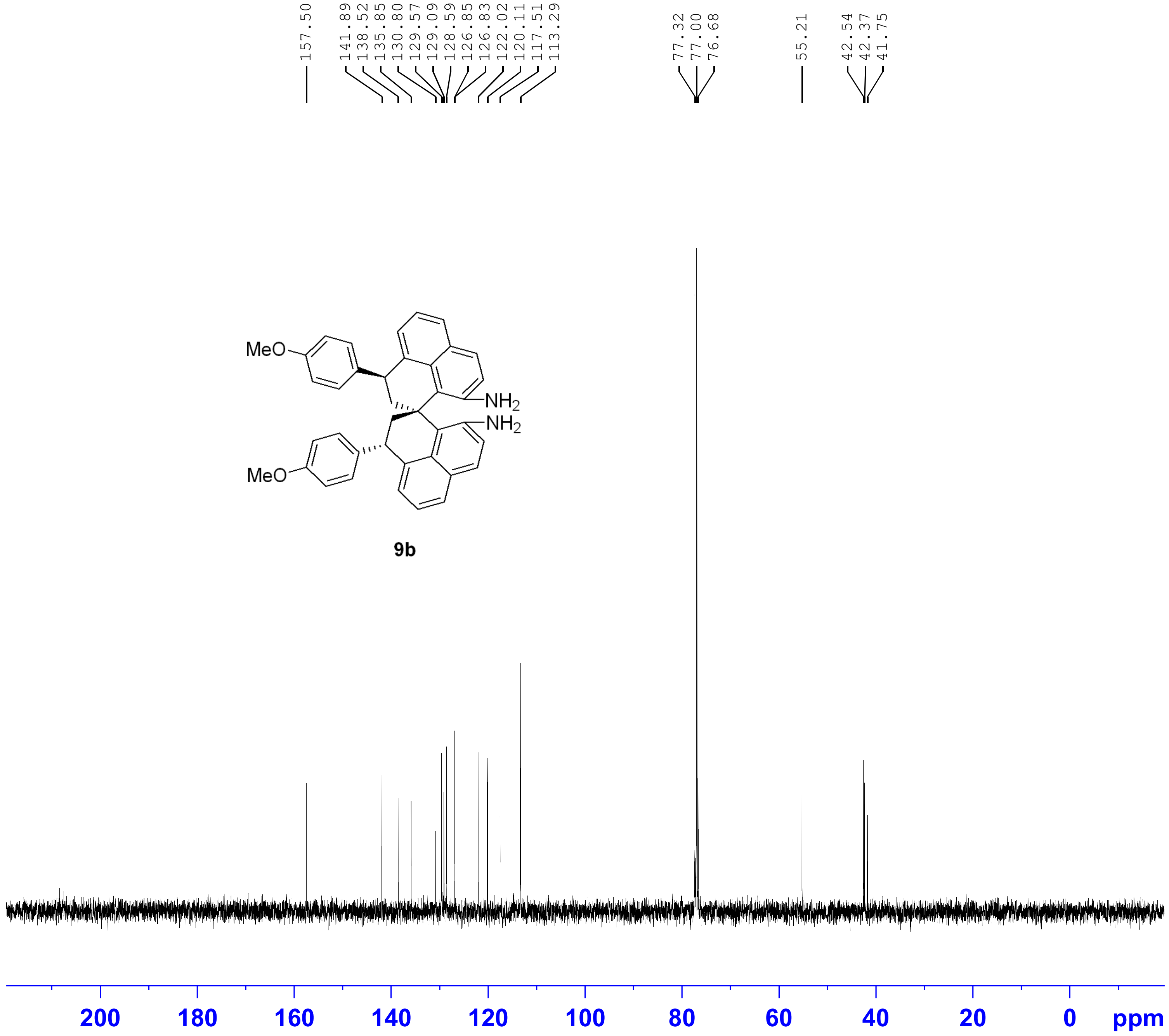

9b

```
Current Data Parameters
NAME          zrh-9-47-2-c
EXPNO                    1
PROCNO                   1

F2 - Acquisition Parameters
Date_             20230401
Time                 19.30
INSTRUM              spect
PROBHD   5 mm PABBO BB/
PULPROG            zgpg30
TD                   65536
SOLVENT              CDCl3
NS                      50
DS                       2
SWH              24038.461 Hz
FIDRES            0.366798 Hz
AQ               1.3631488 sec
RG                  196.92
DW                  20.800 usec
DE                    6.50 usec
TE                   296.8 K
D1              2.00000000 sec
D11             0.03000000 sec
TD0                      1

======== CHANNEL f1 ========
SFO1           100.6228298 MHz
NUC1                   13C
P1                    9.70 usec
PLW1           46.98899841 W

======== CHANNEL f2 ========
SFO2           400.1316005 MHz
NUC2                    1H
CPDPRG[2           waltz16
PCPD2                90.00 usec
PLW2           11.99499989 W
PLW12           0.34213999 W
PLW13           0.27713001 W

F2 - Processing parameters
SI                   32768
SF             100.6127766 MHz
WDW                     EM
SSB                      0
LB                    1.00 Hz
GB                       0
PC                    1.40
```

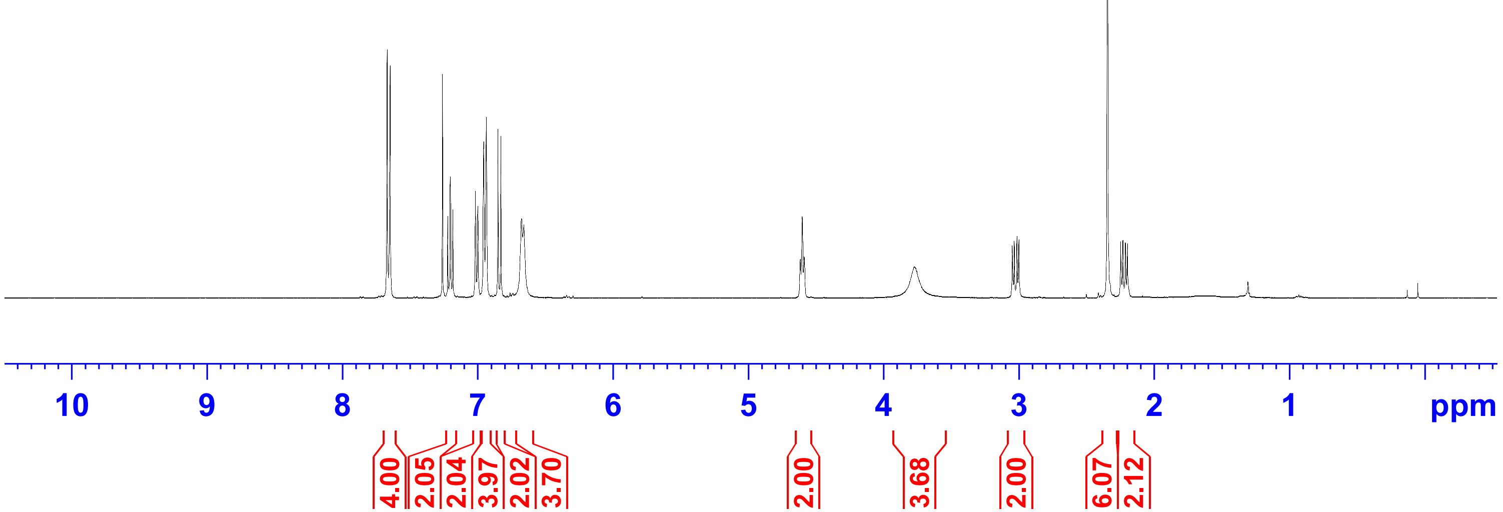

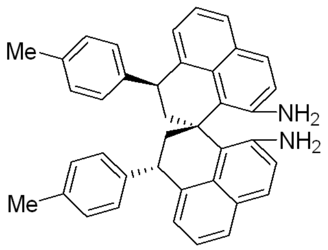

9c

```
Current Data Parameters
NAME          zrh-9-54-h
EXPNO                  1
PROCNO                 1

F2 - Acquisition Parameters
Date_           20230406
Time               15.40
INSTRUM            spect
PROBHD    5 mm DUL 13C-1
PULPROG             zg30
TD                 65536
SOLVENT            CDCl3
NS                     2
DS                     0
SWH             8223.685 Hz
FIDRES          0.125483 Hz
AQ             3.9845889 sec
RG                   161
DW                60.800 usec
DE                  6.00 usec
TE                 292.6 K
D1            1.00000000 sec
TD0                    1

======== CHANNEL f1 ========
NUC1                  1H
P1                 15.80 usec
PL1                -1.00 dB
PL1W         12.17476940 W
SFO1         400.1324710 MHz

F2 - Processing parameters
SI                 32768
SF           400.1300096 MHz
WDW                   EM
SSB                    0
LB                  0.30 Hz
GB                     0
PC                  1.00
```

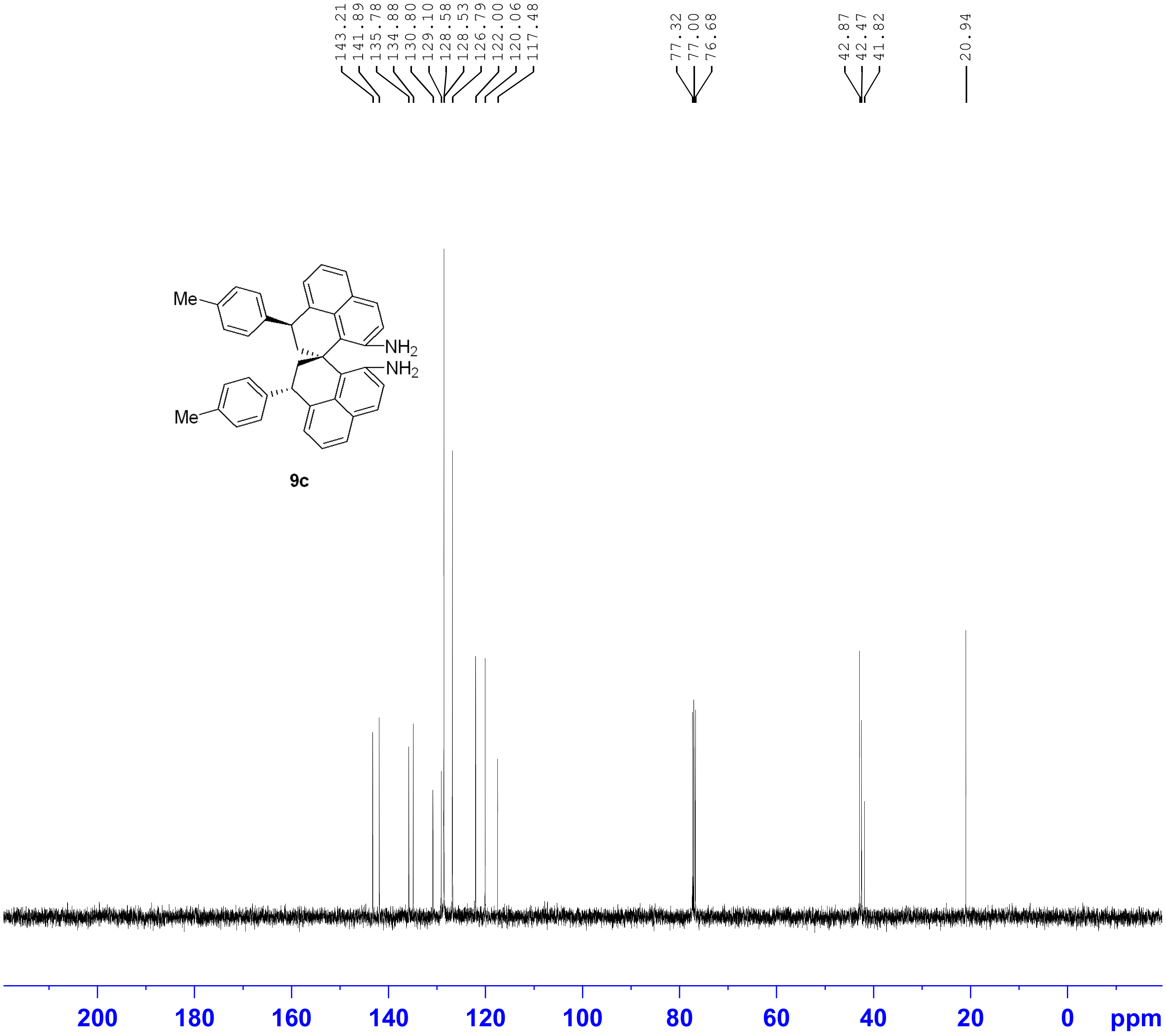

9c

Current Data Parameters
NAME zrh-9-54-c
EXPNO 1
PROCNO 1

F2 - Acquisition Parameters
Date_ 20230406
Time 15.42
INSTRUM spect
PROBHD 5 mm DUL 13C-1
PULPROG zgpg30
TD 65536
SOLVENT CDCl3
NS 34
DS 0
SWH 24038.461 Hz
FIDRES 0.366798 Hz
AQ 1.3631488 sec
RG 2050
DW 20.800 usec
DE 6.00 usec
TE 292.8 K
D1 2.00000000 sec
D11 0.03000000 sec
TD0 1

======== CHANNEL f1 ========
NUC1 13C
P1 40.00 usec
PL1 -3.00 dB
PL1W 60.64365387 W
SFO1 100.6228298 MHz

======== CHANNEL f2 ========
CPDPRG[2 waltz16
NUC2 1H
PCPD2 80.00 usec
PL2 -1.00 dB
PL12 14.39 dB
PL13 18.00 dB
PL2W 12.17476940 W
PL12W 0.35193357 W
PL13W 0.15327126 W
SFO2 400.1316005 MHz

F2 - Processing parameters
SI 32768
SF 100.6127795 MHz
WDW EM
SSB 0
LB 1.00 Hz
GB 0
PC 1.40

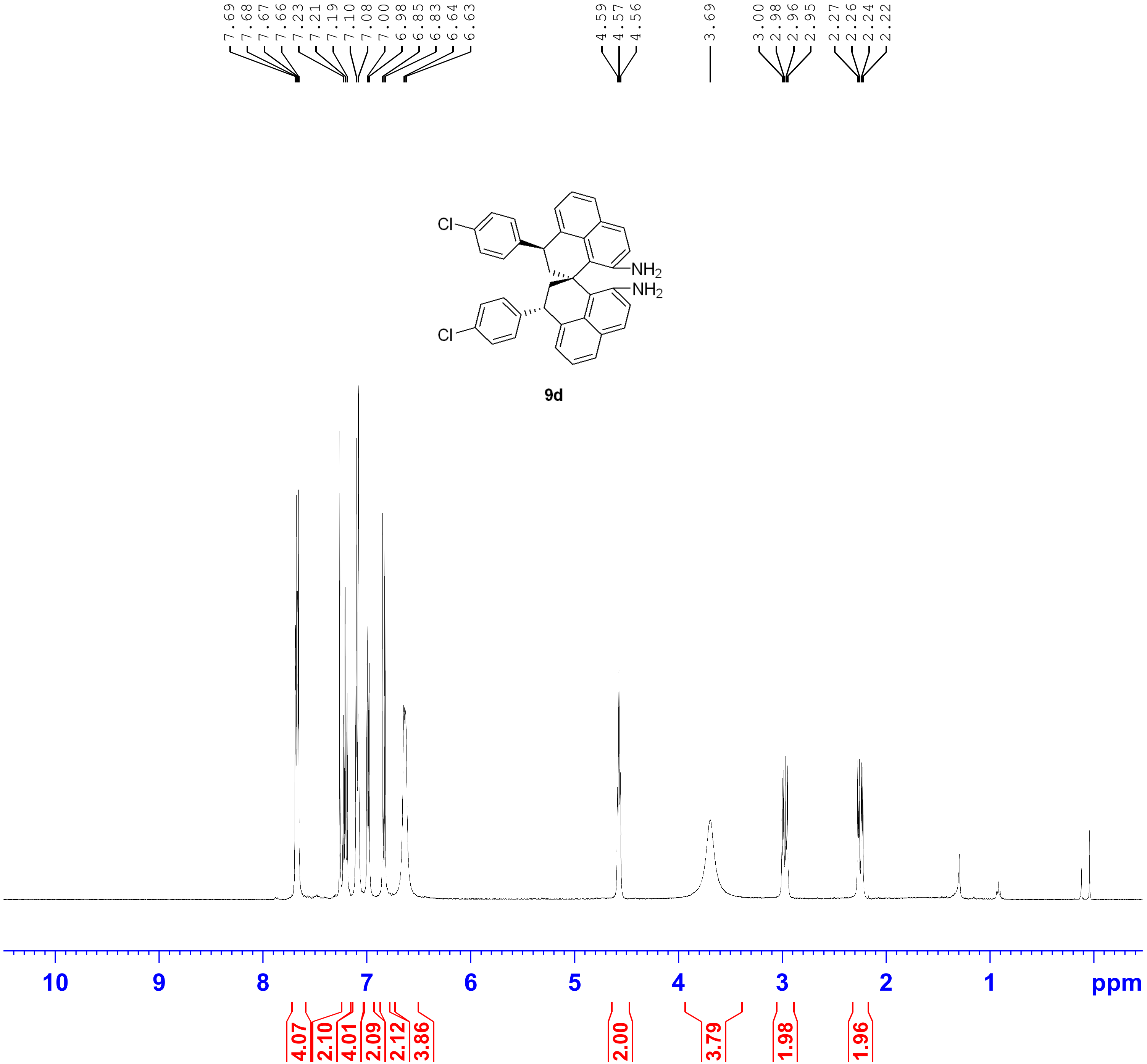


```
Current Data Parameters
NAME      zrh-9-83-1-4Cl-h
EXPNO                    1
PROCNO                   1

F2 - Acquisition Parameters
Date_             20230428
Time                 18.39
INSTRUM              spect
PROBHD    5 mm DUL 13C-1
PULPROG               zg30
TD                   65536
SOLVENT              CDCl3
NS                       1
DS                       0
SWH               8223.685 Hz
FIDRES            0.125483 Hz
AQ               3.9845889 sec
RG                     181
DW                  60.800 usec
DE                    6.00 usec
TE                   292.7 K
D1              1.00000000 sec
TD0                      1

======== CHANNEL f1 ========
NUC1                    1H
P1                   15.80 usec
PL1                  -1.00 dB
PL1W           12.17476940 W
SFO1           400.1324710 MHz

F2 - Processing parameters
SI                   32768
SF             400.1300096 MHz
WDW                     EM
SSB                      0
LB                    0.30 Hz
GB                       0
PC                    1.00
```

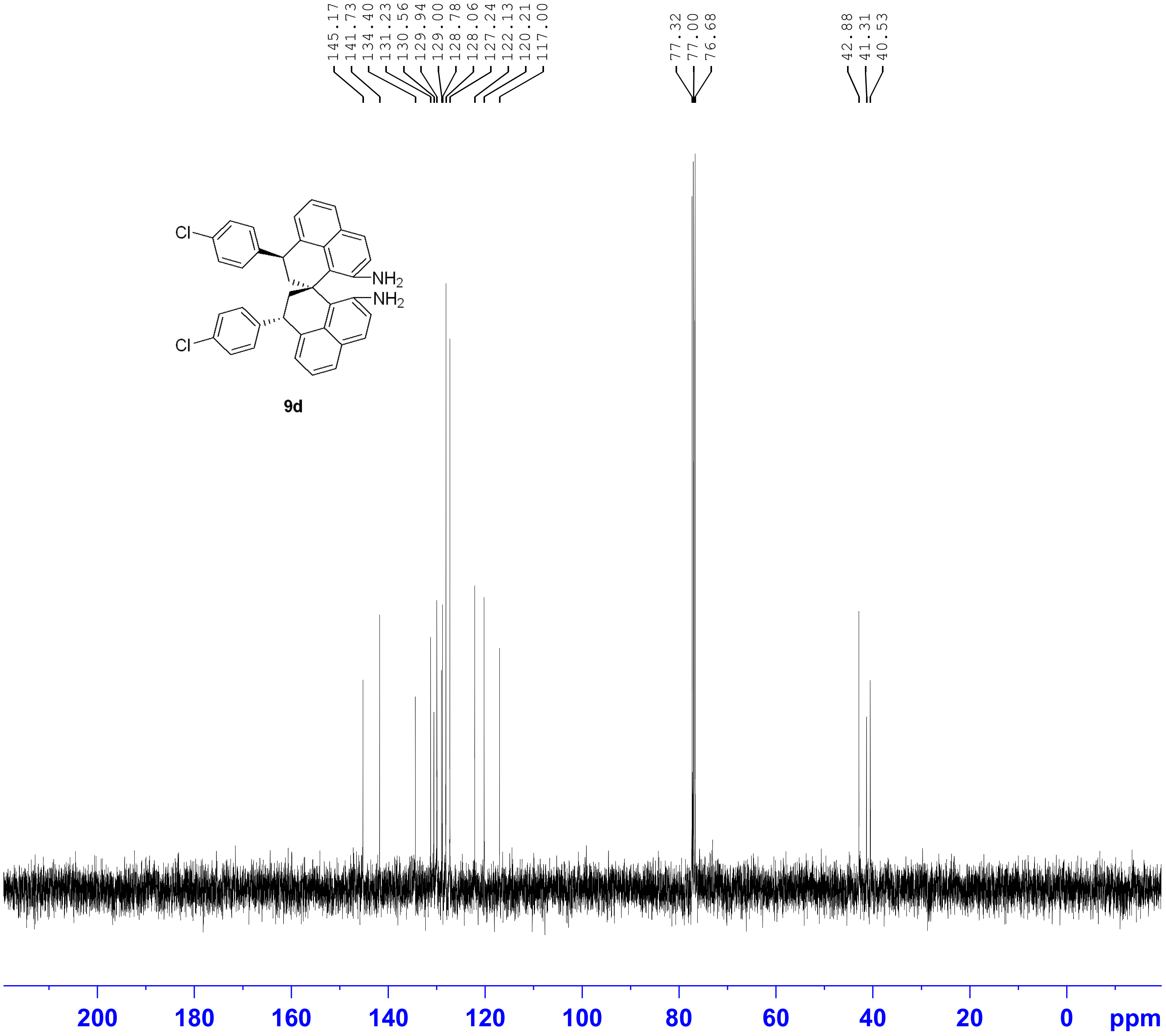

Current Data Parameters
NAME zrh-9-83-1-4Cl-c
EXPNO 1
PROCNO 1

F2 - Acquisition Parameters
Date_ 20230428
Time 18.41
INSTRUM spect
PROBHD 5 mm DUL 13C-1
PULPROG zgpg30
TD 65536
SOLVENT CDCl3
NS 95
DS 0
SWH 24038.461 Hz
FIDRES 0.366798 Hz
AQ 1.3631488 sec
RG 101
DW 20.800 usec
DE 6.00 usec
TE 292.9 K
D1 2.00000000 sec
D11 0.03000000 sec
TD0 1

======== CHANNEL f1 ========
NUC1 13C
P1 40.00 usec
PL1 -3.00 dB
PL1W 60.64365387 W
SFO1 100.6228298 MHz

======== CHANNEL f2 ========
CPDPRG[2 waltz16
NUC2 1H
PCPD2 80.00 usec
PL2 -1.00 dB
PL12 14.39 dB
PL13 18.00 dB
PL2W 12.17476940 W
PL12W 0.35193357 W
PL13W 0.15327126 W
SFO2 400.1316005 MHz

F2 - Processing parameters
SI 32768
SF 100.6127788 MHz
WDW EM
SSB 0
LB 1.00 Hz
GB 0
PC 1.40

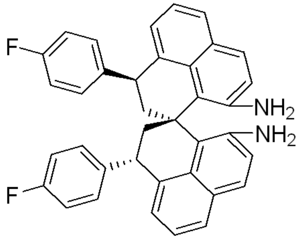

9e

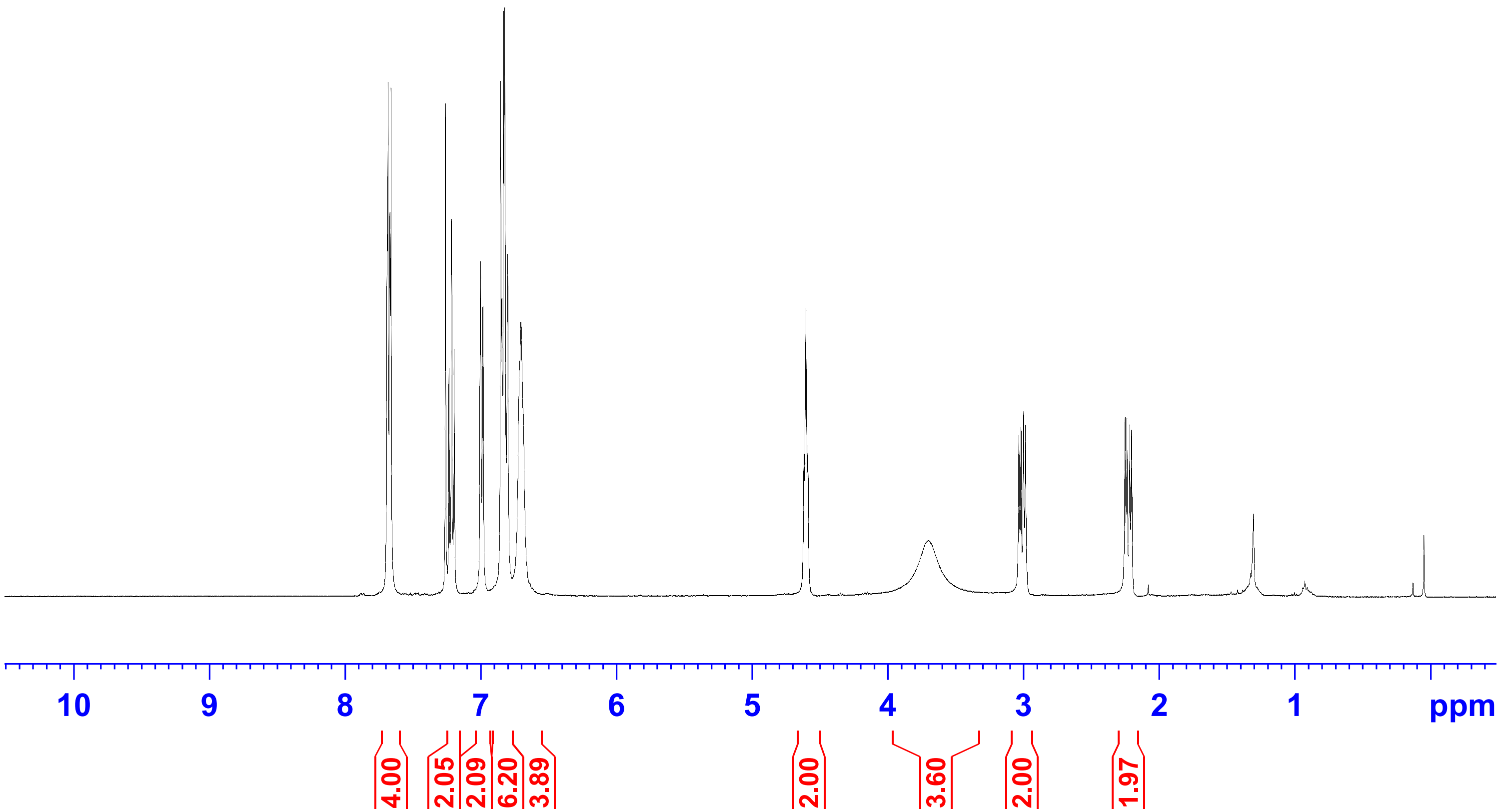

Current Data Parameters
NAME zrh-9-85-h
EXPNO 3
PROCNO 1

F2 - Acquisition Parameters
Date_ 20230429
Time 14.52
INSTRUM spect
PROBHD 5 mm PABBO BB/
PULPROG zg30
TD 65536
SOLVENT CDCl3
NS 3
DS 2
SWH 8012.820 Hz
FIDRES 0.122266 Hz
AQ 4.0894465 sec
RG 31.55
DW 62.400 usec
DE 6.50 usec
TE 296.1 K
D1 1.00000000 sec
TD0 1

======== CHANNEL f1 ========
SFO1 400.1324710 MHz
NUC1 1H
P1 14.50 usec
PLW1 11.99499989 W

F2 - Processing parameters
SI 65536
SF 400.1300100 MHz
WDW EM
SSB 0
LB 0.30 Hz
GB 0
PC 1.00

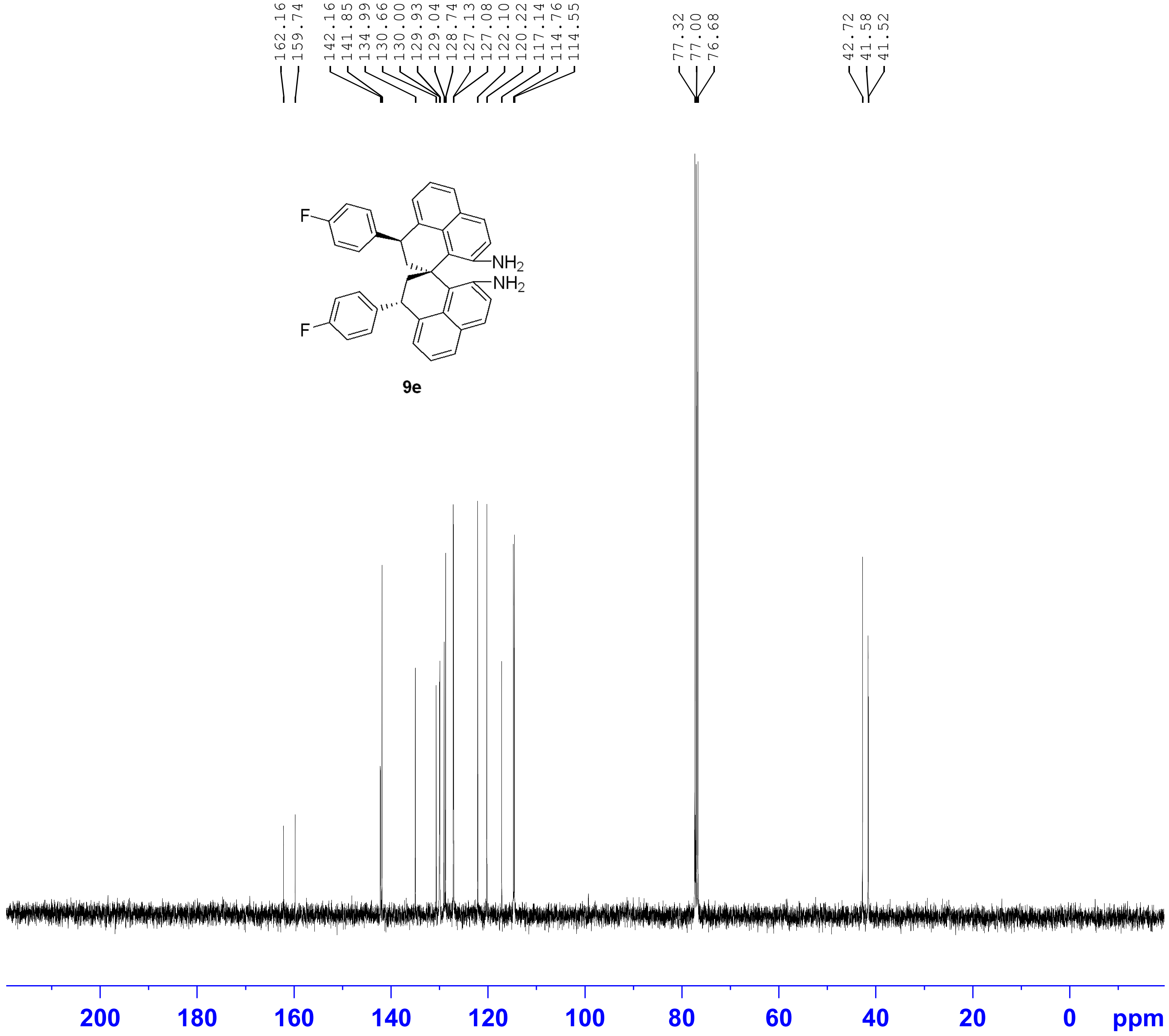

Current Data Parameters
NAME zrh-9-85-c
EXPNO 3
PROCNO 1

F2 - Acquisition Parameters
Date_ 20230429
Time 14.55
INSTRUM spect
PROBHD 5 mm PABBO BB/
PULPROG zgpg30
TD 65536
SOLVENT CDCl3
NS 50
DS 2
SWH 24038.461 Hz
FIDRES 0.366798 Hz
AQ 1.3631488 sec
RG 196.92
DW 20.800 usec
DE 6.50 usec
TE 296.5 K
D1 2.00000000 sec
D11 0.03000000 sec
TD0 1

======== CHANNEL f1 ========
SFO1 100.6228298 MHz
NUC1 13C
P1 9.70 usec
PLW1 46.98899841 W

======== CHANNEL f2 ========
SFO2 400.1316005 MHz
NUC2 1H
CPDPRG[2 waltz16
PCPD2 90.00 usec
PLW2 11.99499989 W
PLW12 0.34213999 W
PLW13 0.27713001 W

F2 - Processing parameters
SI 32768
SF 100.6127787 MHz
WDW EM
SSB 0
LB 1.00 Hz
GB 0
PC 1.40

-117.78

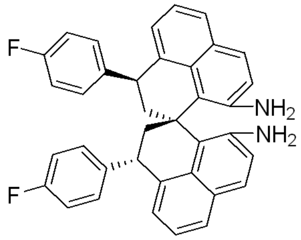

9e

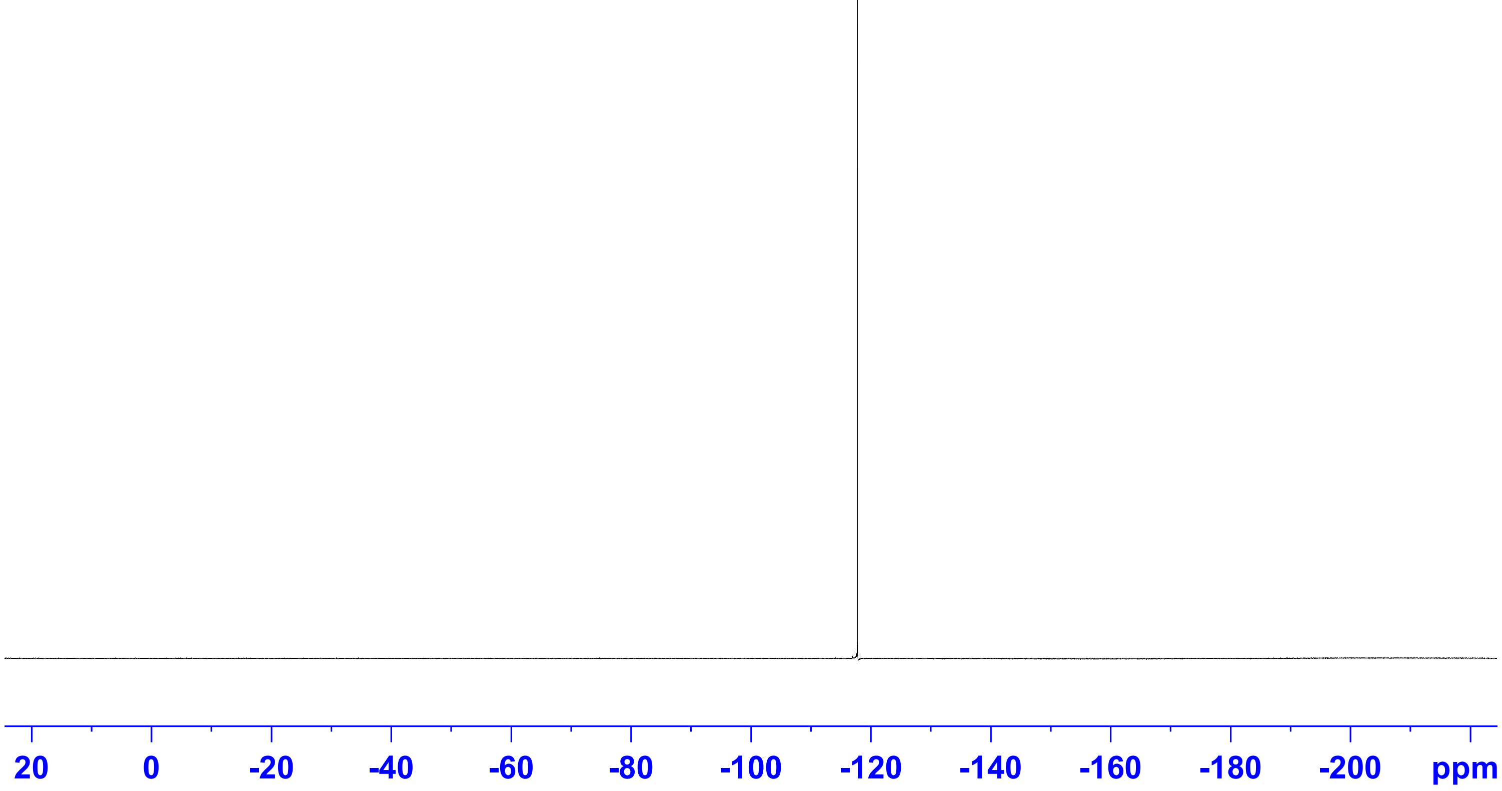

Current Data Parameters
NAME zrh-9-85-f
EXPNO 3
PROCNO 1

F2 - Acquisition Parameters
Date_ 20230429
Time 14.53
INSTRUM spect
PROBHD 5 mm PABBO BB/
PULPROG zgpg30
TD 65536
SOLVENT CDCl3
NS 4
DS 2
SWH 93750.000 Hz
FIDRES 1.430511 Hz
AQ 0.3495253 sec
RG 196.92
DW 5.333 usec
DE 6.50 usec
TE 296.2 K
D1 2.00000000 sec
D11 0.03000000 sec
TD0 1

======== CHANNEL f1 ========
SFO1 376.4607162 MHz
NUC1 19F
P1 14.70 usec
PLW1 15.99600029 W

======== CHANNEL f2 ========
SFO2 400.1316005 MHz
NUC2 1H
CPDPRG[2 waltz16
PCPD2 90.00 usec
PLW2 11.99499989 W
PLW12 0.34213999 W
PLW13 0.27713001 W

F2 - Processing parameters
SI 32768
SF 376.4983660 MHz
WDW EM
SSB 0
LB 1.00 Hz
GB 0
PC 1.40

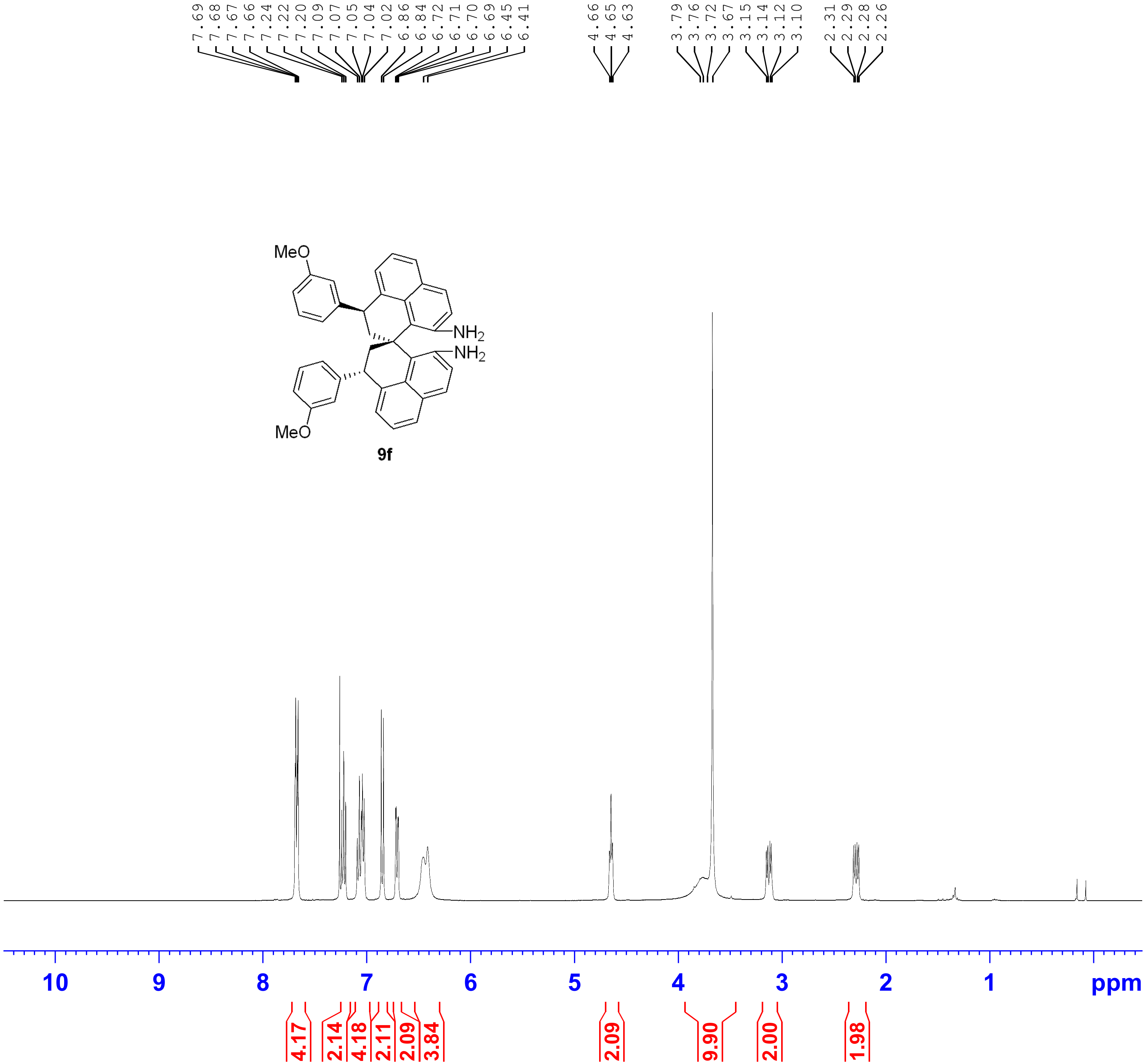


```
Current Data Parameters
NAME        zrh-9-83-3-3OMe-h
EXPNO                       1
PROCNO                      1

F2 - Acquisition Parameters
Date_               20230428
Time                   18.48
INSTRUM                spect
PROBHD     5 mm DUL 13C-1
PULPROG                 zg30
TD                     65536
SOLVENT                CDCl3
NS                         7
DS                         0
SWH                 8223.685 Hz
FIDRES              0.125483 Hz
AQ                 3.9845889 sec
RG                       114
DW                    60.800 usec
DE                      6.00 usec
TE                     292.7 K
D1                1.00000000 sec
TD0                        1

======== CHANNEL f1 ========
NUC1                      1H
P1                     15.80 usec
PL1                    -1.00 dB
PL1W             12.17476940 W
SFO1             400.1324710 MHz

F2 - Processing parameters
SI                     32768
SF               400.1300096 MHz
WDW                       EM
SSB                        0
LB                      0.30 Hz
GB                         0
PC                      1.00
```

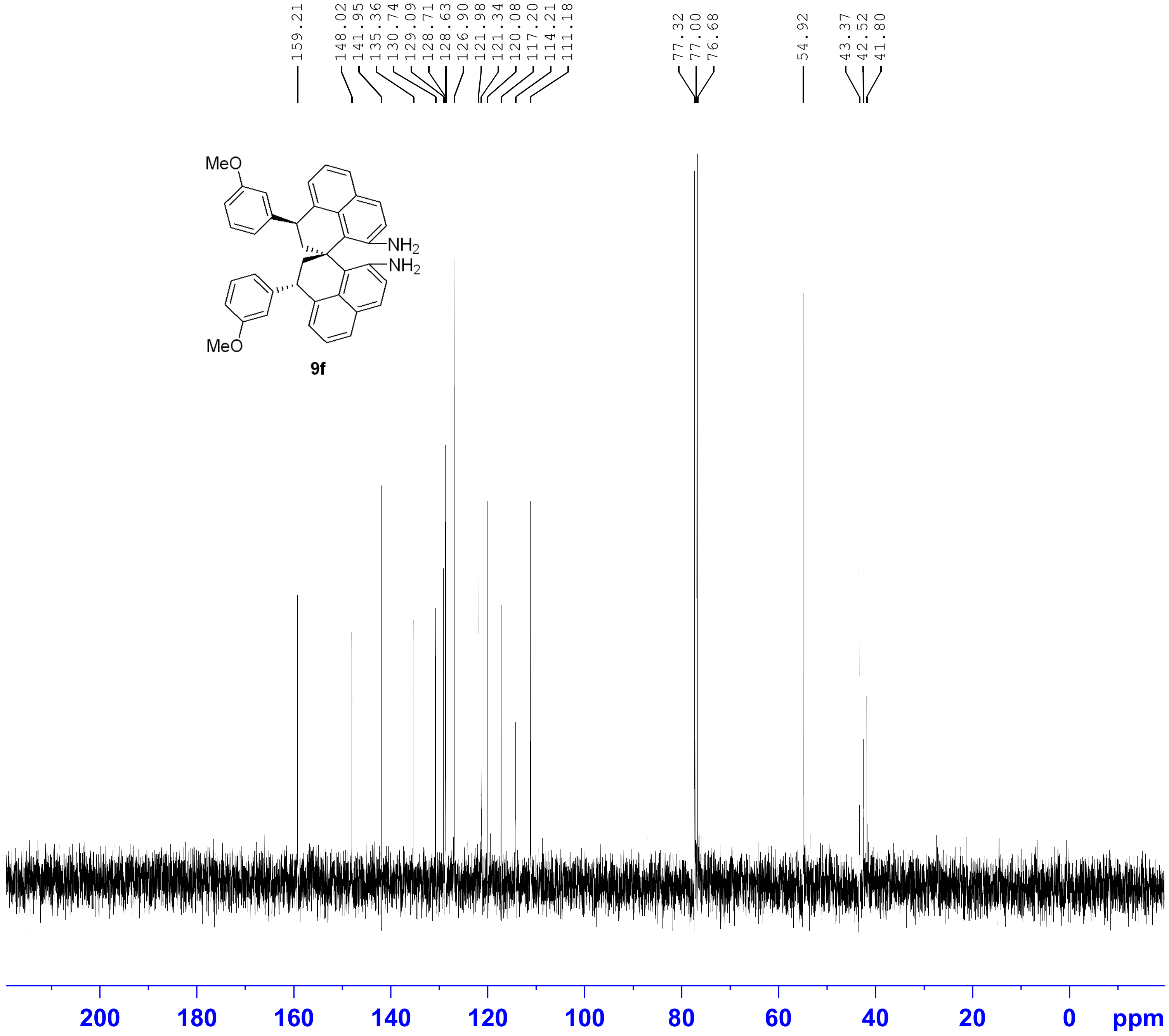


```
Current Data Parameters
NAME      zrh-9-83-3-3OMe-c
EXPNO                    1
PROCNO                   1

F2 - Acquisition Parameters
Date_             20230428
Time                 18.50
INSTRUM              spect
PROBHD    5 mm DUL 13C-1
PULPROG            zgpg30
TD                   65536
SOLVENT              CDCl3
NS                      70
DS                       0
SWH              24038.461 Hz
FIDRES            0.366798 Hz
AQ               1.3631488 sec
RG                    50.8
DW                  20.800 usec
DE                    6.00 usec
TE                   292.9 K
D1              2.00000000 sec
D11             0.03000000 sec
TD0                      1

======== CHANNEL f1 ========
NUC1                   13C
P1                   40.00 usec
PL1                  -3.00 dB
PL1W           60.64365387 W
SFO1          100.6228298 MHz

======== CHANNEL f2 ========
CPDPRG[2           waltz16
NUC2                    1H
PCPD2                80.00 usec
PL2                  -1.00 dB
PL12                 14.39 dB
PL13                 18.00 dB
PL2W           12.17476940 W
PL12W           0.35193357 W
PL13W           0.15327126 W
SFO2          400.1316005 MHz

F2 - Processing parameters
SI                   32768
SF            100.6127846 MHz
WDW                     EM
SSB                      0
LB                    1.00 Hz
GB                       0
PC                    1.40
```

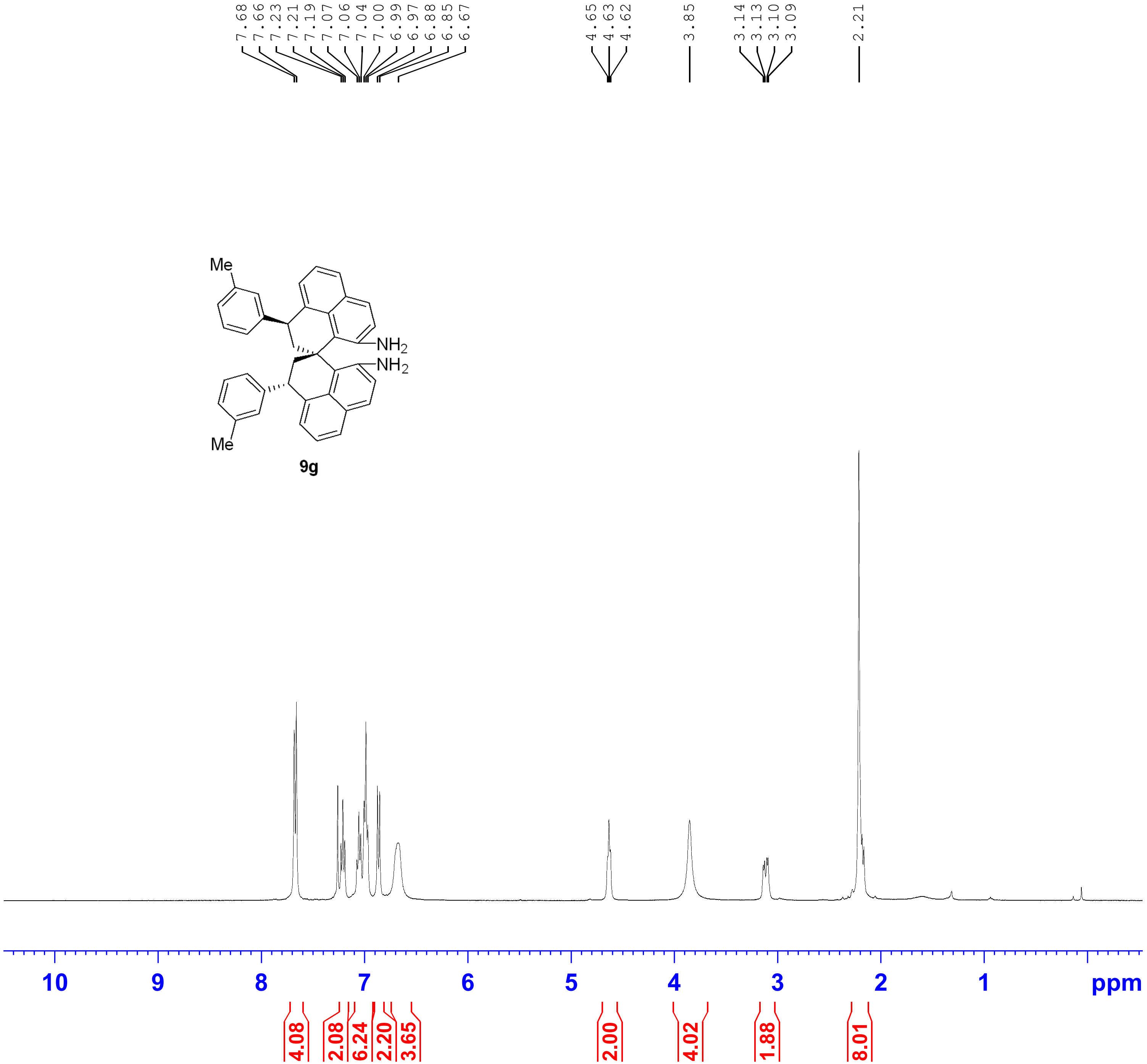

Current Data Parameters
NAME zrh-9-47-1-h
EXPNO 1
PROCNO 1

F2 - Acquisition Parameters
Date_ 20230401
Time 19.19
INSTRUM spect
PROBHD 5 mm PABBO BB/
PULPROG zg30
TD 65536
SOLVENT CDCl3
NS 4
DS 2
SWH 8012.820 Hz
FIDRES 0.122266 Hz
AQ 4.0894465 sec
RG 31.55
DW 62.400 usec
DE 6.50 usec
TE 296.2 K
D1 1.00000000 sec
TD0 1

======== CHANNEL f1 ========
SFO1 400.1324710 MHz
NUC1 1H
P1 14.50 usec
PLW1 11.99499989 W

F2 - Processing parameters
SI 65536
SF 400.1300103 MHz
WDW EM
SSB 0
LB 0.30 Hz
GB 0
PC 1.00

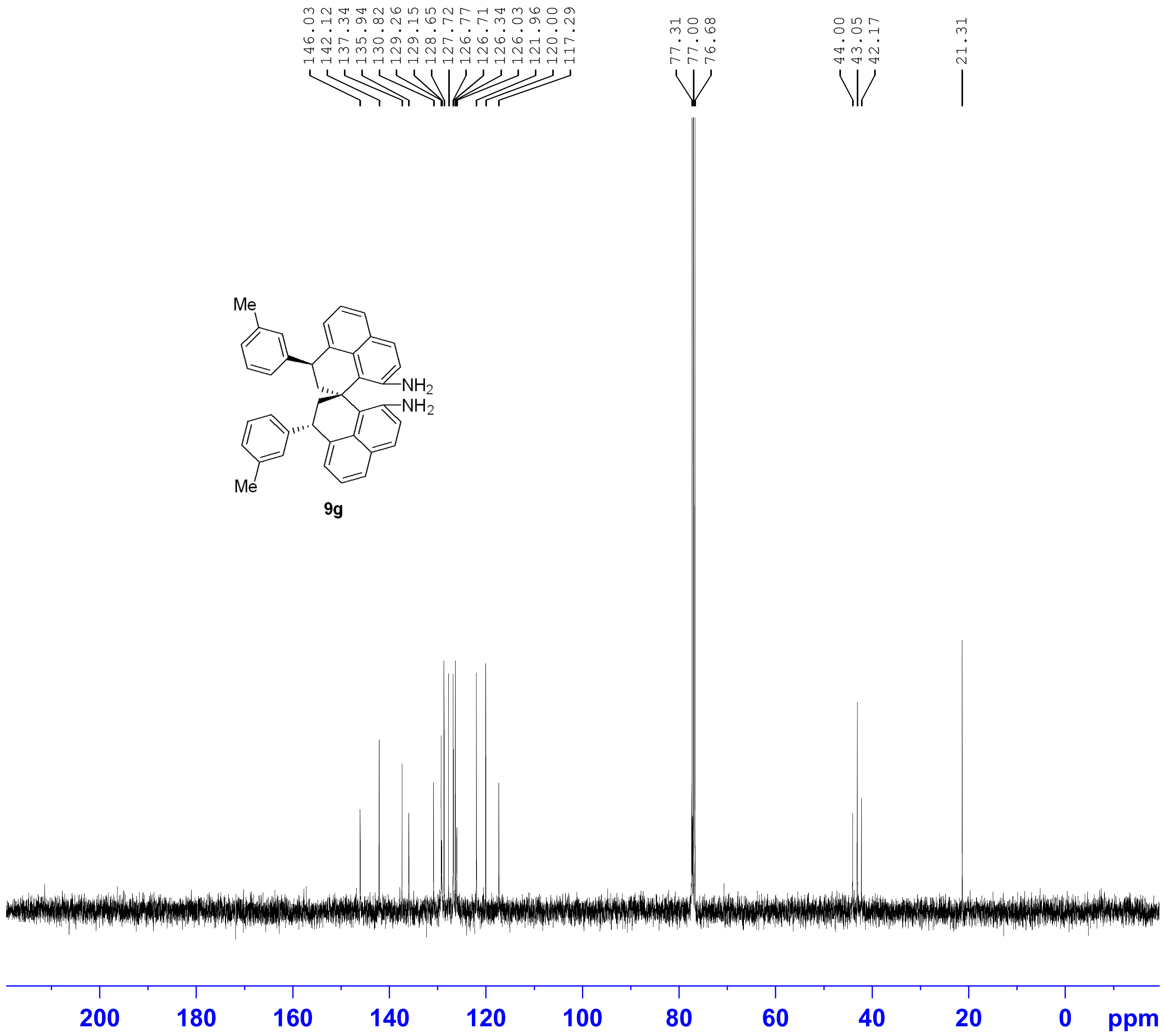


```
Current Data Parameters
NAME          zrh-9-47-1-c
EXPNO                    1
PROCNO                   1

F2 - Acquisition Parameters
Date_             20230401
Time                 19.21
INSTRUM              spect
PROBHD    5 mm PABBO BB/
PULPROG            zgpg30
TD                   65536
SOLVENT              CDCl3
NS                      50
DS                       2
SWH              24038.461 Hz
FIDRES            0.366798 Hz
AQ               1.3631488 sec
RG                  196.92
DW                  20.800 usec
DE                    6.50 usec
TE                   296.7 K
D1              2.00000000 sec
D11             0.03000000 sec
TD0                      1

======== CHANNEL f1 ========
SFO1           100.6228298 MHz
NUC1                   13C
P1                    9.70 usec
PLW1           46.98899841 W

======== CHANNEL f2 ========
SFO2           400.1316005 MHz
NUC2                    1H
CPDPRG[2           waltz16
PCPD2                90.00 usec
PLW2           11.99499989 W
PLW12           0.34213999 W
PLW13           0.27713001 W

F2 - Processing parameters
SI                   32768
SF             100.6127795 MHz
WDW                     EM
SSB                      0
LB                    1.00 Hz
GB                       0
PC                    1.40
```

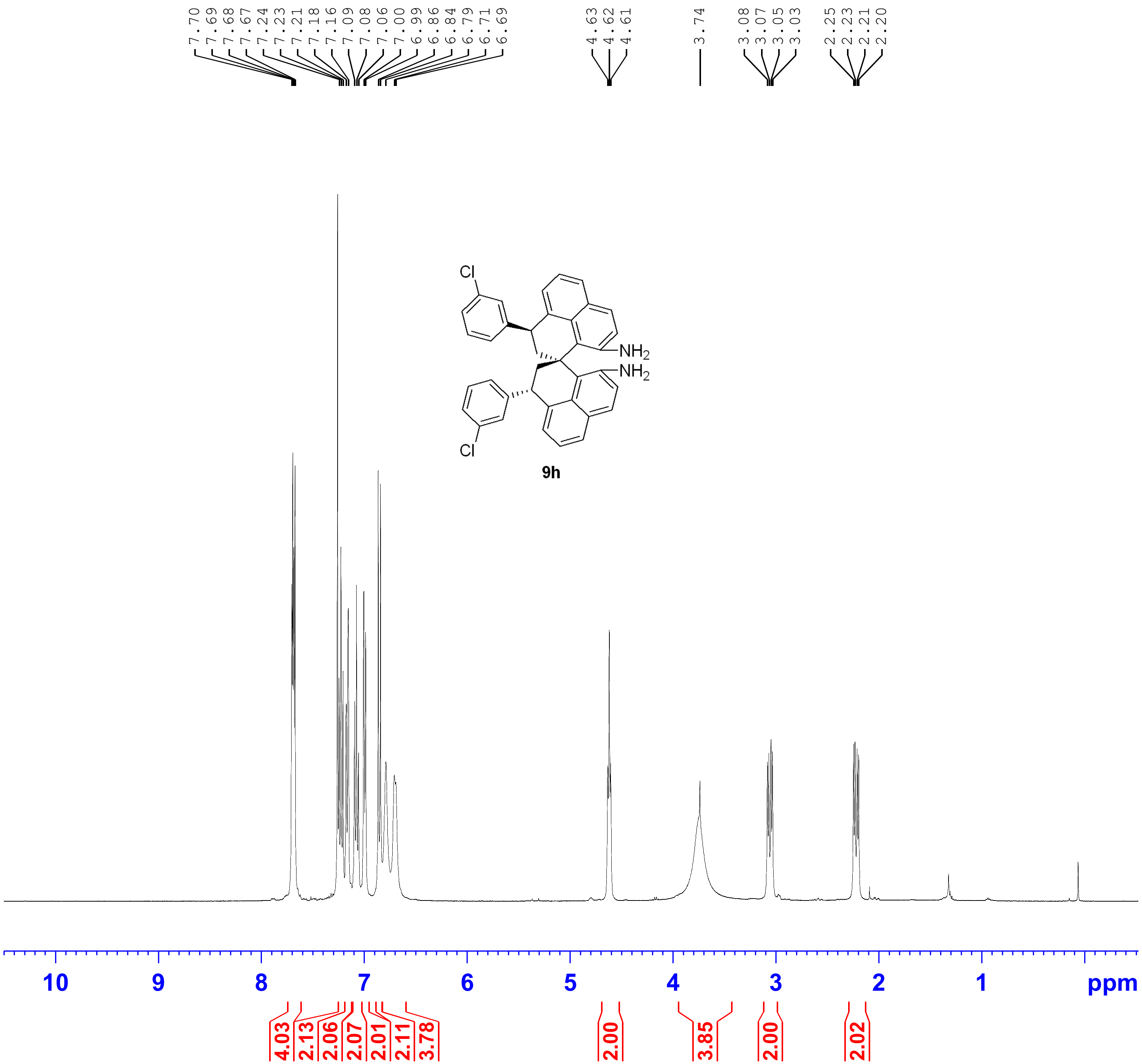

Current Data Parameters
NAME zrh-9-100-h
EXPNO 1
PROCNO 1

F2 - Acquisition Parameters
Date_ 20230509
Time 16.42 h
INSTRUM AvanceNeo 400MHz
PROBHD Z163739_0629 (
PULPROG zg30
TD 65536
SOLVENT CDCl3
NS 3
DS 2
SWH 8196.722 Hz
FIDRES 0.250144 Hz
AQ 3.9976959 sec
RG 64
DW 61.000 usec
DE 13.89 usec
TE 299.0 K
D1 1.00000000 sec
TD0 1
SFO1 400.1824711 MHz
NUC1 1H
P0 2.67 usec
P1 8.00 usec
PLW1 21.26700020 W

F2 - Processing parameters
SI 65536
SF 400.1800092 MHz
WDW EM
SSB 0
LB 0.30 Hz
GB 0
PC 1.00

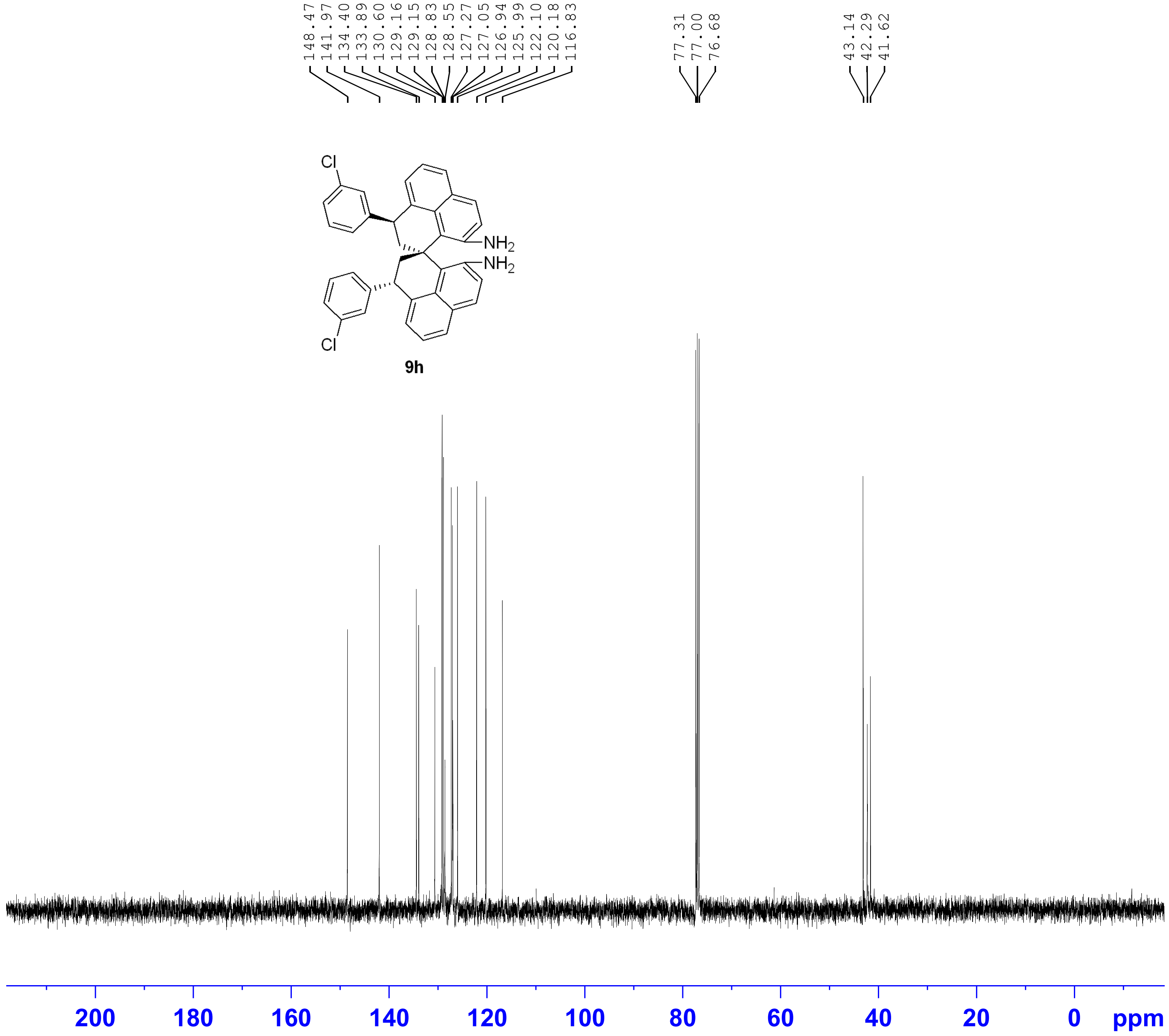

Current Data Parameters
NAME zrh-9-100-c
EXPNO 3
PROCNO 1

F2 - Acquisition Parameters
Date_ 20230509
Time 16.45 h
INSTRUM AvanceNeo 400MHz
PROBHD Z163739_0629 (
PULPROG zgpg30
TD 65536
SOLVENT CDCl3
NS 34
DS 4
SWH 23809.523 Hz
FIDRES 0.726609 Hz
AQ 1.3762560 sec
RG 16
DW 21.000 usec
DE 6.50 usec
TE 299.2 K
D1 2.00000000 sec
D11 0.03000000 sec
TD0 1
SFO1 100.6354036 MHz
NUC1 13C
P0 2.67 usec
P1 8.00 usec
PLW1 85.25399780 W
SFO2 400.1816007 MHz
NUC2 1H
CPDPRG[2 waltz65
PCPD2 90.00 usec
PLW2 21.26700020 W
PLW12 0.16802999 W
PLW13 0.08452000 W

F2 - Processing parameters
SI 32768
SF 100.6253537 MHz
WDW EM
SSB 0
LB 1.00 Hz
GB 0
PC 1.40

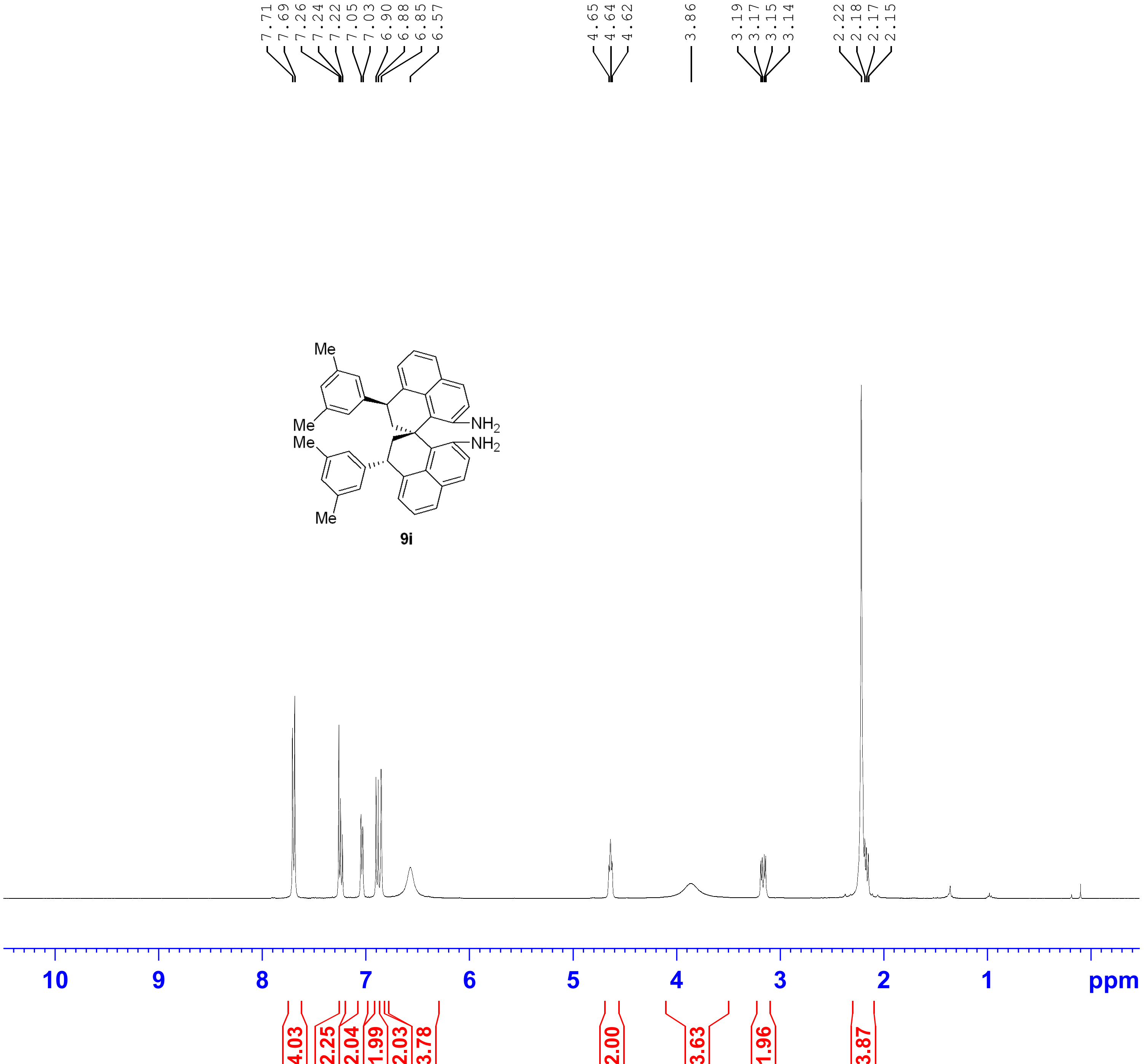


```
Current Data Parameters
NAME      zrh-9-83-2-3.5Me-h
EXPNO                  1
PROCNO                 1

F2 - Acquisition Parameters
Date_           20230428
Time               18.32
INSTRUM            spect
PROBHD    5 mm DUL 13C-1
PULPROG             zg30
TD                 65536
SOLVENT            CDCl3
NS                     2
DS                     0
SWH             8223.685 Hz
FIDRES          0.125483 Hz
AQ             3.9845889 sec
RG                    64
DW                60.800 usec
DE                  6.00 usec
TE                 292.7 K
D1            1.00000000 sec
TD0                    1

======== CHANNEL f1 ========
NUC1                  1H
P1                 15.80 usec
PL1                -1.00 dB
PL1W         12.17476940 W
SFO1         400.1324710 MHz

F2 - Processing parameters
SI                 32768
SF           400.1300096 MHz
WDW                   EM
SSB                    0
LB                  0.30 Hz
GB                     0
PC                  1.00
```

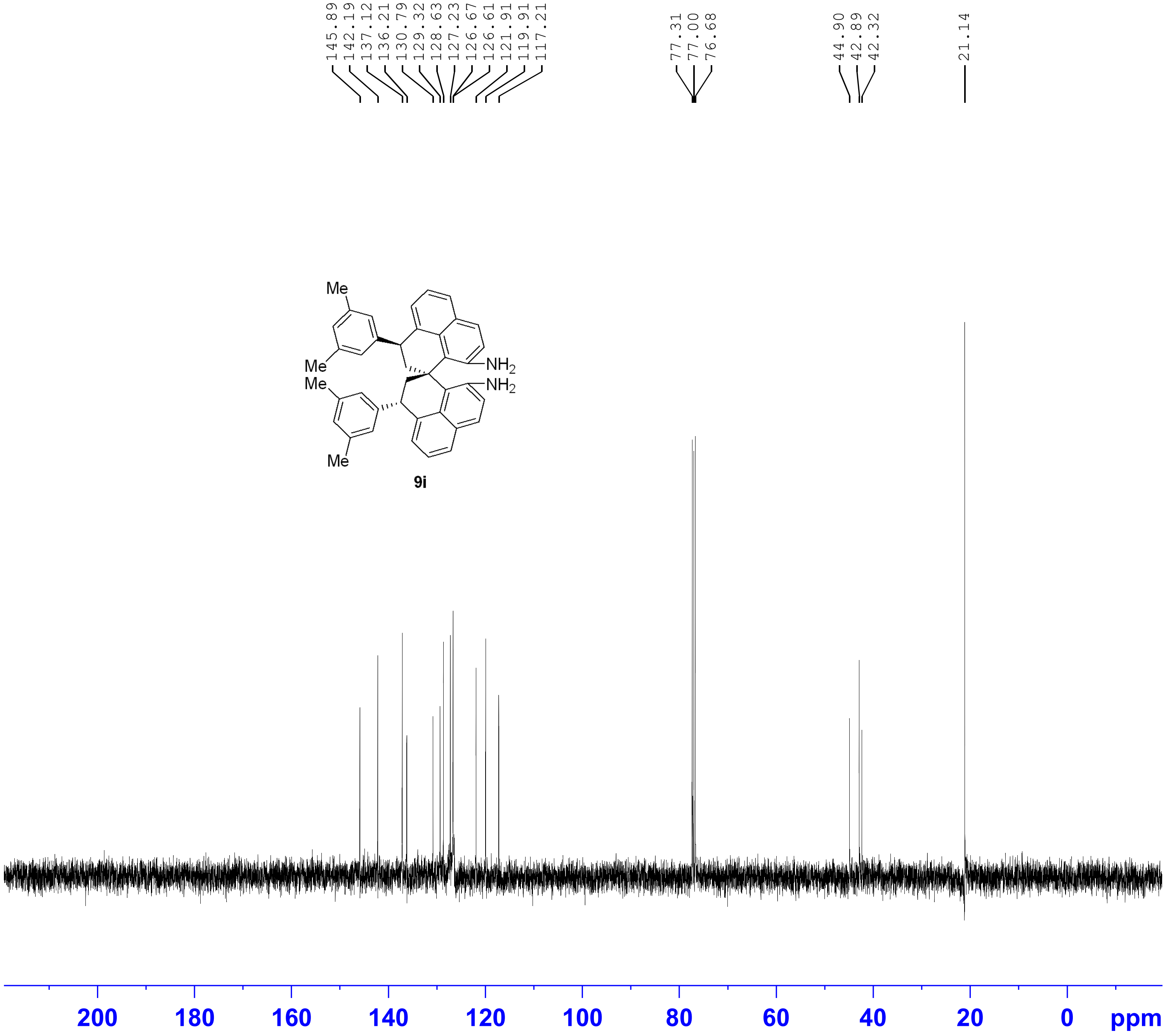

Current Data Parameters
NAME zrh-9-83-2-3.5Me-c
EXPNO 1
PROCNO 1

F2 - Acquisition Parameters
Date_ 20230428
Time 18.33
INSTRUM spect
PROBHD 5 mm DUL 13C-1
PULPROG zgpg30
TD 65536
SOLVENT CDCl3
NS 77
DS 0
SWH 24038.461 Hz
FIDRES 0.366798 Hz
AQ 1.3631488 sec
RG 80.6
DW 20.800 usec
DE 6.00 usec
TE 292.9 K
D1 2.00000000 sec
D11 0.03000000 sec
TD0 1

======== CHANNEL f1 ========
NUC1 13C
P1 40.00 usec
PL1 -3.00 dB
PL1W 60.64365387 W
SFO1 100.6228298 MHz

======== CHANNEL f2 ========
CPDPRG[2 waltz16
NUC2 1H
PCPD2 80.00 usec
PL2 -1.00 dB
PL12 14.39 dB
PL13 18.00 dB
PL2W 12.17476940 W
PL12W 0.35193357 W
PL13W 0.15327126 W
SFO2 400.1316005 MHz

F2 - Processing parameters
SI 32768
SF 100.6127853 MHz
WDW EM
SSB 0
LB 1.00 Hz
GB 0
PC 1.40

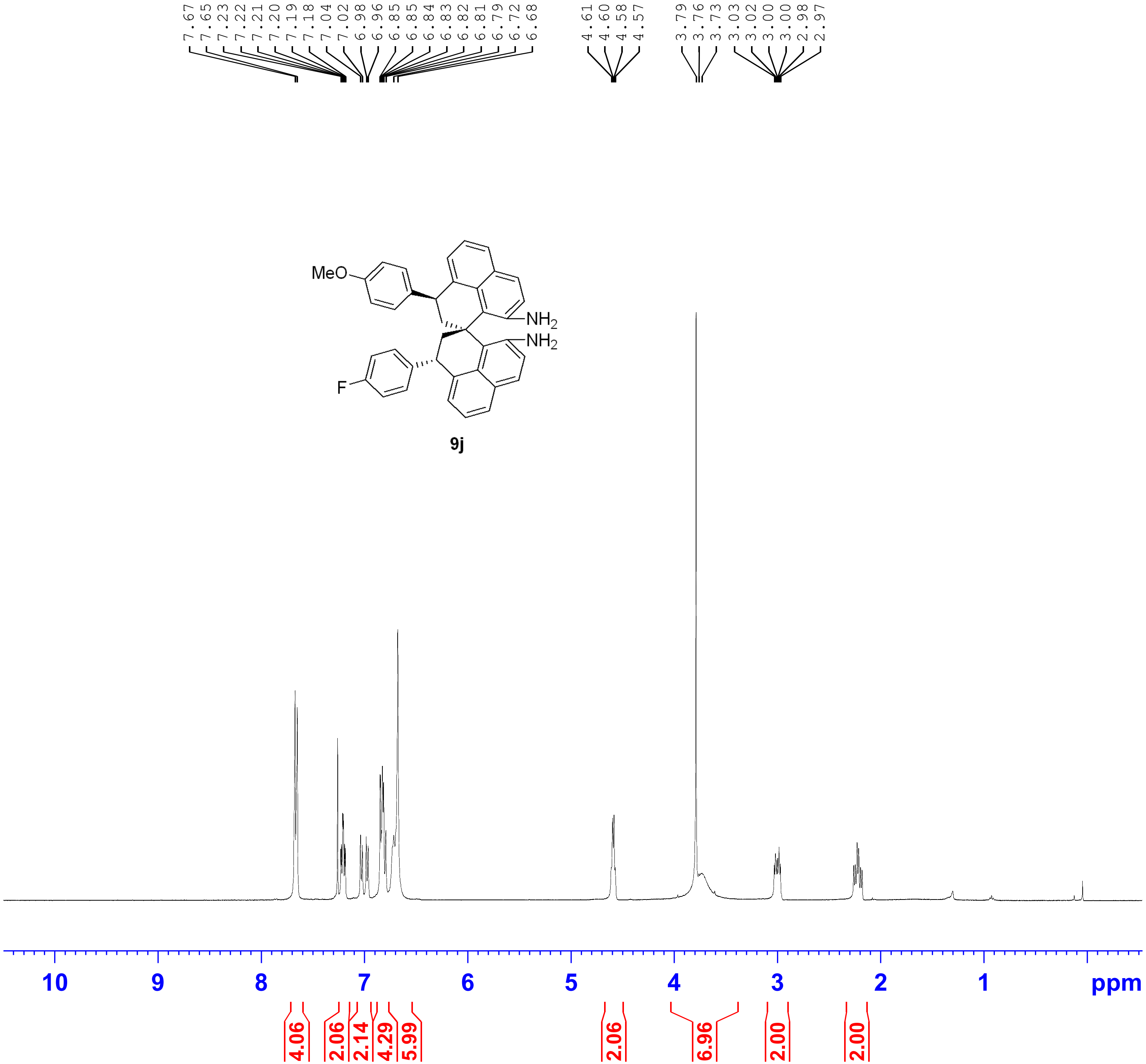

9j

Current Data Parameters
NAME zrh-9-90-h
EXPNO 1
PROCNO 1

F2 - Acquisition Parameters
Date_ 20230503
Time 19.38
INSTRUM spect
PROBHD 5 mm PABBO BB/
PULPROG zg30
TD 65536
SOLVENT CDCl3
NS 2
DS 2
SWH 8012.820 Hz
FIDRES 0.122266 Hz
AQ 4.0894465 sec
RG 31.55
DW 62.400 usec
DE 6.50 usec
TE 295.9 K
D1 1.00000000 sec
TD0 1

======== CHANNEL f1 ========
SFO1 400.1324710 MHz
NUC1 1H
P1 14.50 usec
PLW1 11.99499989 W

F2 - Processing parameters
SI 65536
SF 400.1300097 MHz
WDW EM
SSB 0
LB 0.30 Hz
GB 0
PC 1.00

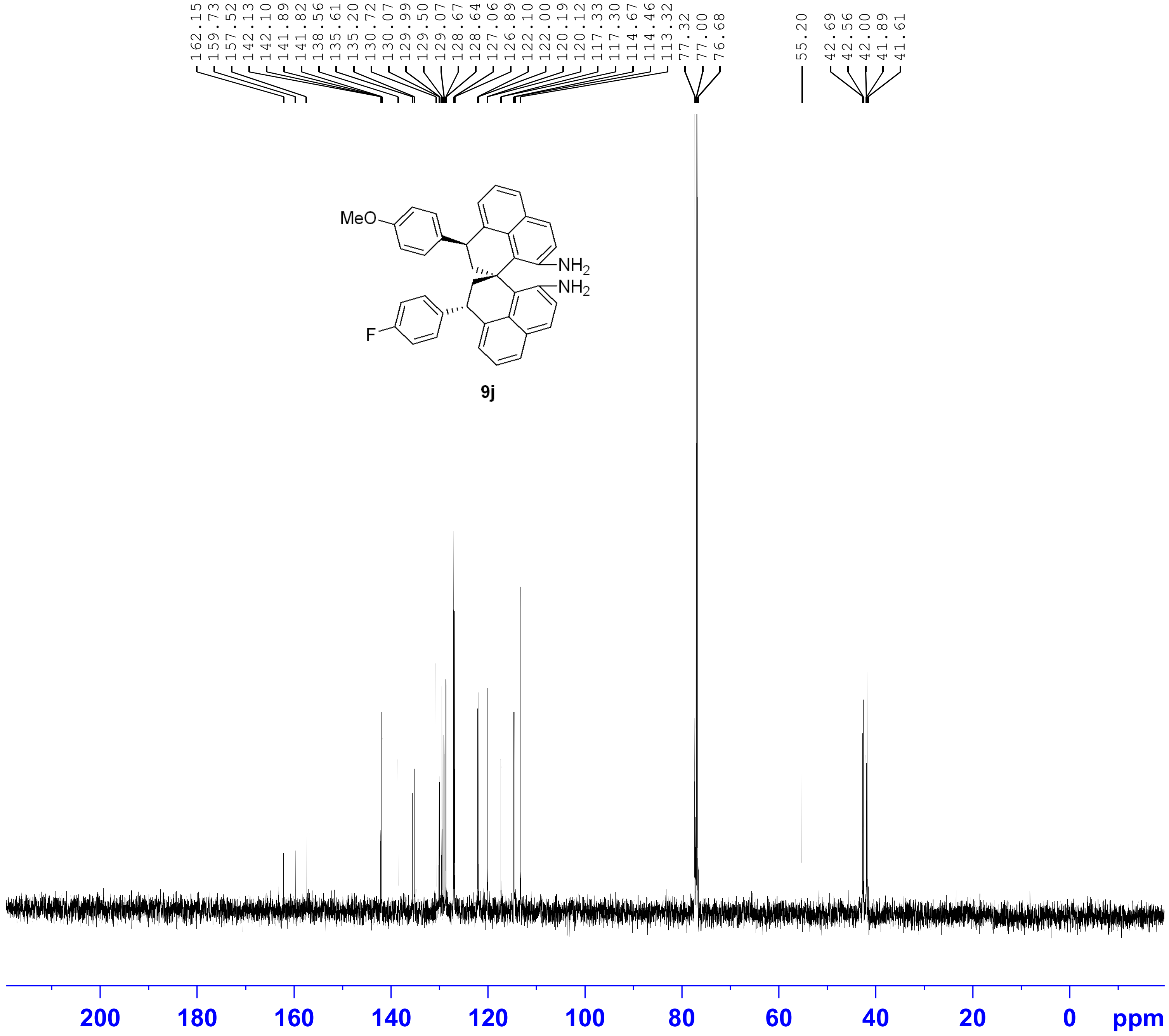

Current Data Parameters
NAME zrh-9-90-c
EXPNO 1
PROCNO 1

F2 - Acquisition Parameters
Date_ 20230503
Time 19.41
INSTRUM spect
PROBHD 5 mm PABBO BB/
PULPROG zgpg30
TD 65536
SOLVENT CDCl3
NS 54
DS 2
SWH 24038.461 Hz
FIDRES 0.366798 Hz
AQ 1.3631488 sec
RG 196.92
DW 20.800 usec
DE 6.50 usec
TE 296.2 K
D1 2.00000000 sec
D11 0.03000000 sec
TD0 1

======== CHANNEL f1 ========
SFO1 100.6228298 MHz
NUC1 13C
P1 9.70 usec
PLW1 46.98899841 W

======== CHANNEL f2 ========
SFO2 400.1316005 MHz
NUC2 1H
CPDPRG[2 waltz16
PCPD2 90.00 usec
PLW2 11.99499989 W
PLW12 0.34213999 W
PLW13 0.27713001 W

F2 - Processing parameters
SI 32768
SF 100.6127788 MHz
WDW EM
SSB 0
LB 1.00 Hz
GB 0
PC 1.40

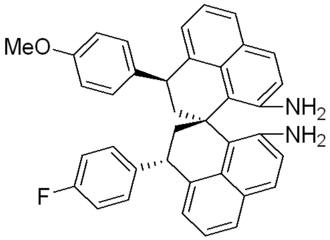

9j

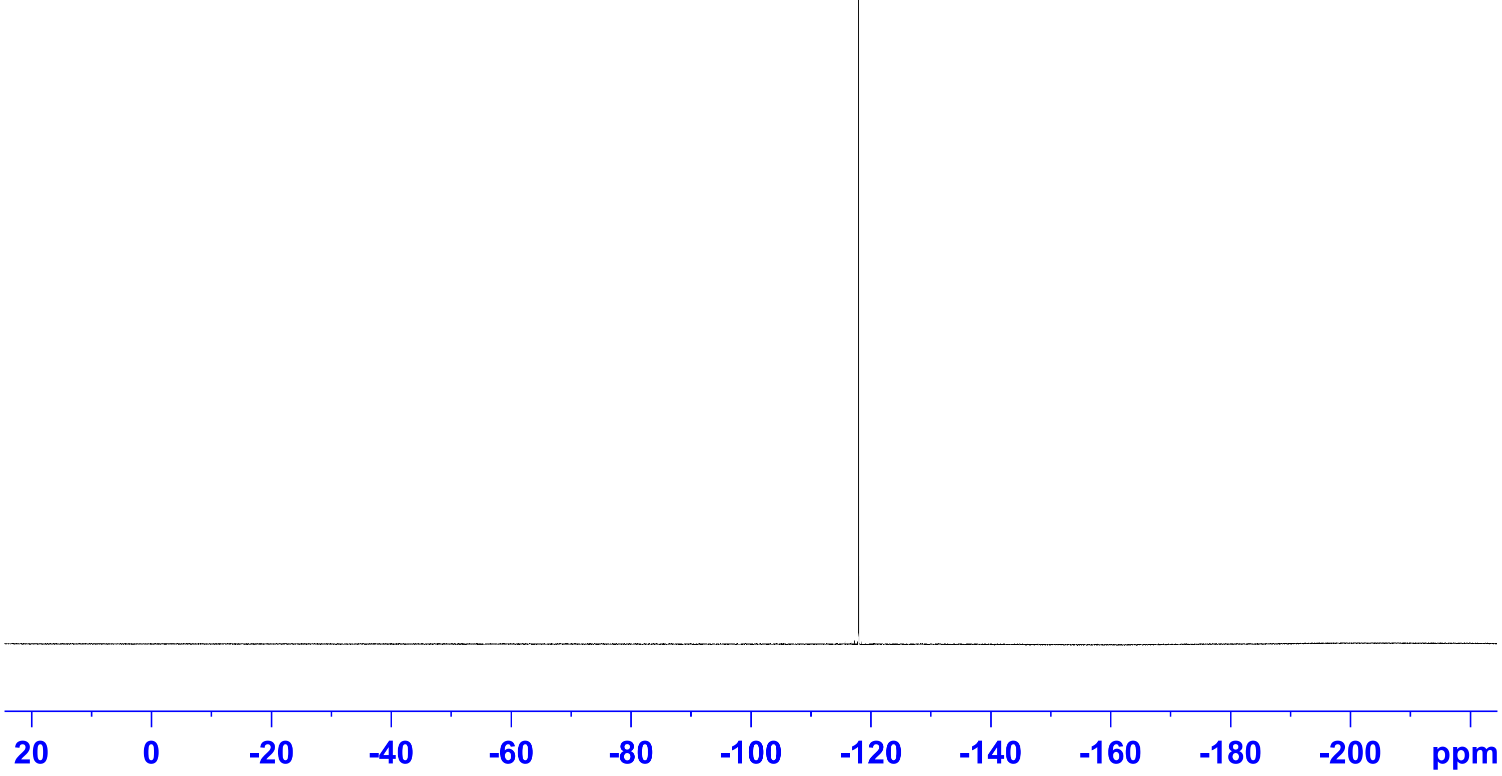


```
Current Data Parameters
NAME          zrh-9-90-f
EXPNO                  1
PROCNO                 1

F2 - Acquisition Parameters
Date_           20230503
Time               19.39
INSTRUM            spect
PROBHD    5 mm PABBO BB/
PULPROG          zgpg30
TD                 65536
SOLVENT            CDCl3
NS                     4
DS                     2
SWH            93750.000 Hz
FIDRES          1.430511 Hz
AQ             0.3495253 sec
RG                196.92
DW                 5.333 usec
DE                  6.50 usec
TE                 296.0 K
D1            2.00000000 sec
D11           0.03000000 sec
TD0                    1

======== CHANNEL f1 ========
SFO1         376.4607162 MHz
NUC1                 19F
P1                 14.70 usec
PLW1         15.99600029 W

======== CHANNEL f2 ========
SFO2         400.1316005 MHz
NUC2                  1H
CPDPRG[2         waltz16
PCPD2              90.00 usec
PLW2         11.99499989 W
PLW12         0.34213999 W
PLW13         0.27713001 W

F2 - Processing parameters
SI                 32768
SF           376.4983660 MHz
WDW                   EM
SSB                    0
LB                  1.00 Hz
GB                     0
PC                  1.40
```

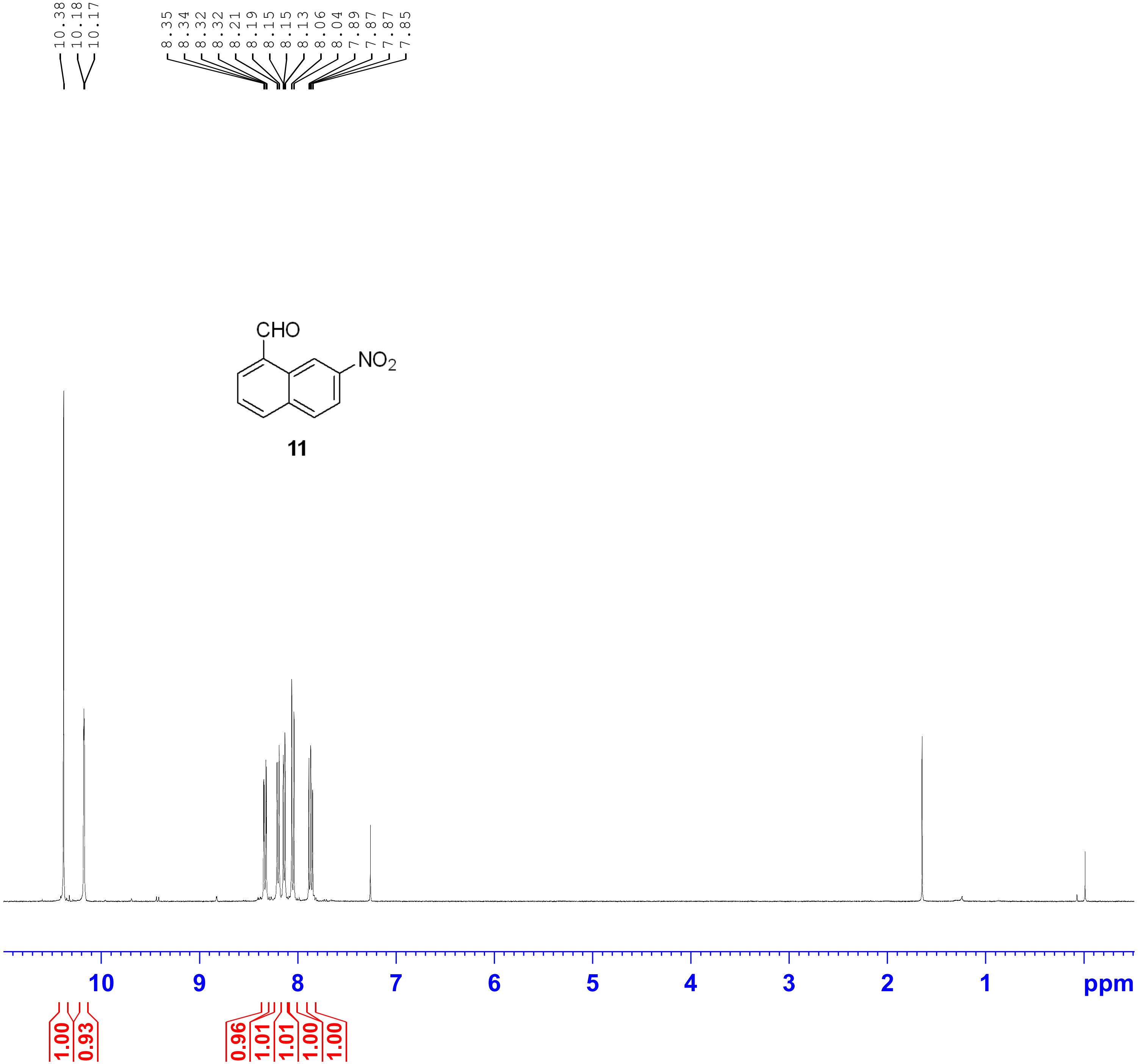


```
Current Data Parameters
NAME          zrh-7-11-h
EXPNO                  1
PROCNO                 1

F2 - Acquisition Parameters
Date_           20220706
Time               11.18
INSTRUM            spect
PROBHD   5 mm PABBO BB/
PULPROG             zg30
TD                 65536
SOLVENT            CDCl3
NS                     1
DS                     0
SWH             8012.820 Hz
FIDRES          0.122266 Hz
AQ             4.0894465 sec
RG                126.97
DW                62.400 usec
DE                  6.50 usec
TE                 295.3 K
D1            1.00000000 sec
TD0                    1

======== CHANNEL f1 ========
SFO1         400.1324710 MHz
NUC1                  1H
P1                 14.50 usec
PLW1         11.99499989 W

F2 - Processing parameters
SI                 65536
SF           400.1300100 MHz
WDW                   EM
SSB                    0
LB                  0.30 Hz
GB                     0
PC                  1.00
```

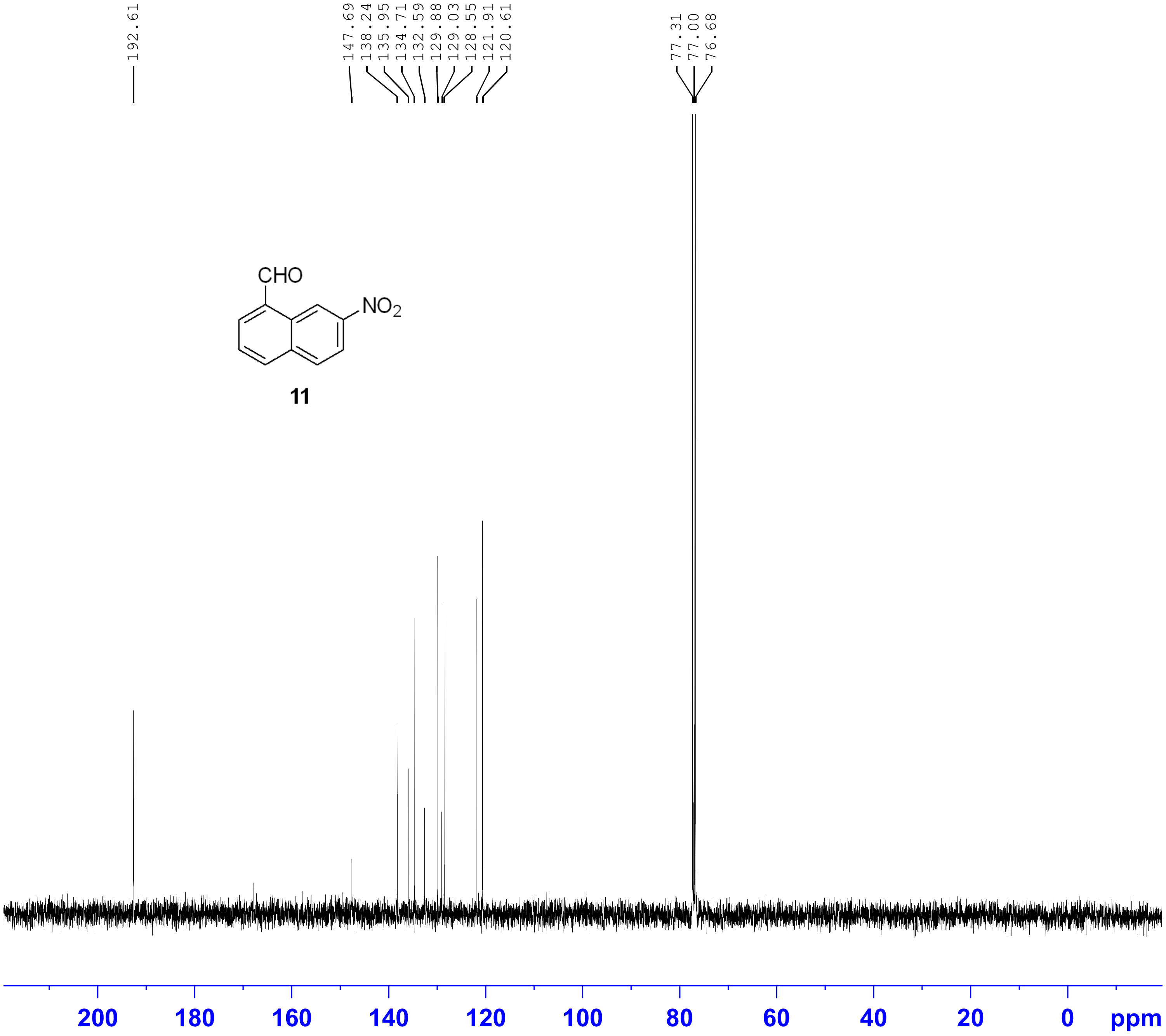


```
Current Data Parameters
NAME            zrh-7-11-c
EXPNO                    1
PROCNO                   1

F2 - Acquisition Parameters
Date_             20220706
Time                 11.19
INSTRUM              spect
PROBHD     5 mm PABBO BB/
PULPROG             zgpg30
TD                   65536
SOLVENT              CDCl3
NS                      50
DS                       0
SWH              24038.461 Hz
FIDRES            0.366798 Hz
AQ               1.3631488 sec
RG                  196.92
DW                  20.800 usec
DE                    6.50 usec
TE                   295.4 K
D1              2.00000000 sec
D11             0.03000000 sec
TD0                      1

======== CHANNEL f1 ========
SFO1           100.6228298 MHz
NUC1                   13C
P1                    9.70 usec
PLW1           46.98899841 W

======== CHANNEL f2 ========
SFO2           400.1316005 MHz
NUC2                    1H
CPDPRG[2           waltz16
PCPD2                90.00 usec
PLW2           11.99499989 W
PLW12           0.34213999 W
PLW13           0.27713001 W

F2 - Processing parameters
SI                   32768
SF             100.6127751 MHz
WDW                     EM
SSB                      0
LB                    1.00 Hz
GB                       0
PC                    1.40
```

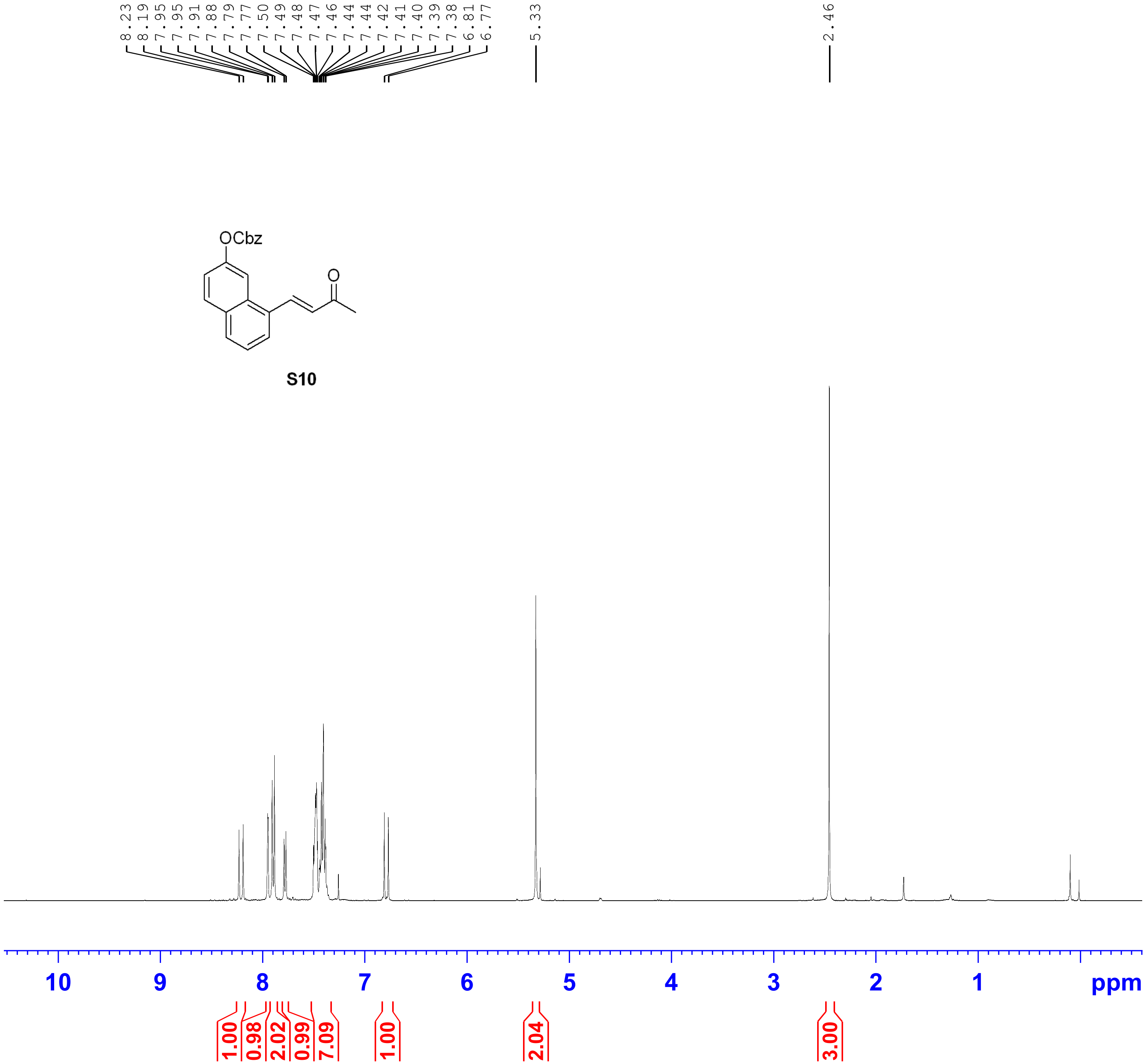

S10

Current Data Parameters	
NAME	zrh-8-113-h
EXPNO	1
PROCNO	1
F2 - Acquisition Parameters	
Date_	20230106
Time	13.58 h
INSTRUM	AvanceNeo 400MHz
PROBHD	Z163739_0629 (
PULPROG	zg30
TD	65536
SOLVENT	CDCl3
NS	3
DS	2
SWH	8196.722 Hz
FIDRES	0.250144 Hz
AQ	3.9976959 sec
RG	101
DW	61.000 usec
DE	13.89 usec
TE	295.9 K
D1	1.00000000 sec
TD0	1
SFO1	400.1824711 MHz
NUC1	1H
P0	2.67 usec
P1	8.00 usec
PLW1	21.26700020 W
F2 - Processing parameters	
SI	65536
SF	400.1800099 MHz
WDW	EM
SSB	0
LB	0.30 Hz
GB	0
PC	1.00

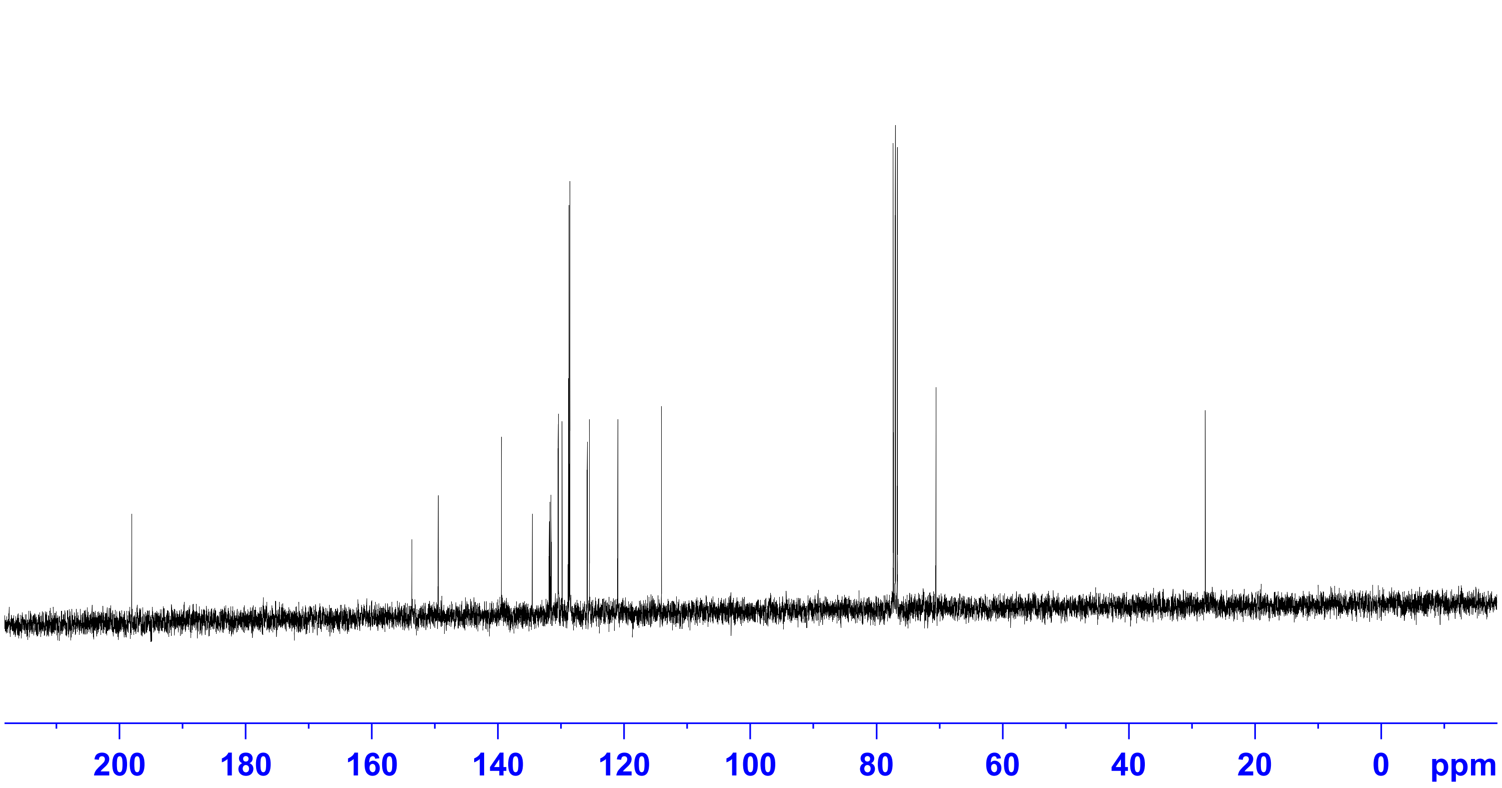

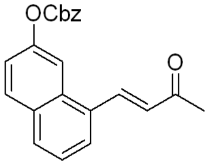

S10

Current Data Parameters
NAME zrh-8-113-c
EXPNO 3
PROCNO 1

F2 - Acquisition Parameters
Date_ 20230106
Time 14.00 h
INSTRUM AvanceNeo 400MHz
PROBHD Z163739_0629 (
PULPROG zgpg30
TD 65536
SOLVENT CDCl3
NS 10
DS 4
SWH 23809.523 Hz
FIDRES 0.726609 Hz
AQ 1.3762560 sec
RG 10
DW 21.000 usec
DE 6.50 usec
TE 296.1 K
D1 2.00000000 sec
D11 0.03000000 sec
TD0 1
SFO1 100.6354036 MHz
NUC1 13C
P0 2.67 usec
P1 8.00 usec
PLW1 85.25399780 W
SFO2 400.1816007 MHz
NUC2 1H
CPDPRG[2 waltz65
PCPD2 90.00 usec
PLW2 21.26700020 W
PLW12 0.16802999 W
PLW13 0.08452000 W

F2 - Processing parameters
SI 32768
SF 100.6253521 MHz
WDW EM
SSB 0
LB 1.00 Hz
GB 0
PC 1.40

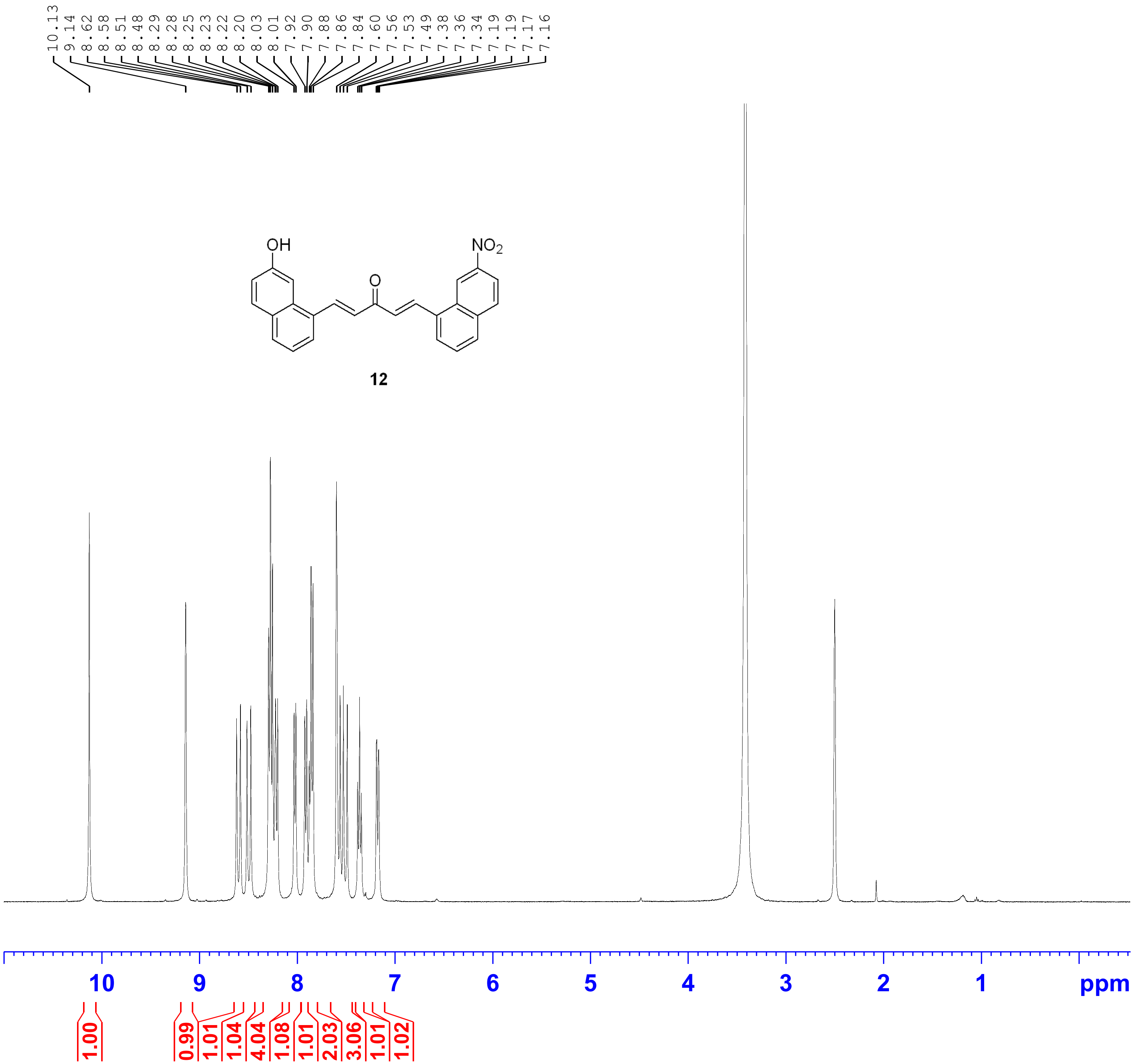


```
Current Data Parameters
NAME          zrh-8-130-h
EXPNO                   1
PROCNO                  1

F2 - Acquisition Parameters
Date_            20230201
Time                20.48 h
INSTRUM    AvanceNeo 400MHz
PROBHD    Z163739_0629 (
PULPROG              zg30
TD                  65536
SOLVENT              DMSO
NS                      6
DS                      2
SWH              8196.722 Hz
FIDRES           0.250144 Hz
AQ              3.9976959 sec
RG                    101
DW                 61.000 usec
DE                  13.89 usec
TE                  295.0 K
D1             1.00000000 sec
TD0                     1
SFO1          400.1824711 MHz
NUC1                   1H
P0                   2.67 usec
P1                   8.00 usec
PLW1          21.26700020 W

F2 - Processing parameters
SI                  65536
SF            400.1800026 MHz
WDW                    EM
SSB                     0
LB                   0.30 Hz
GB                      0
PC                   1.00
```

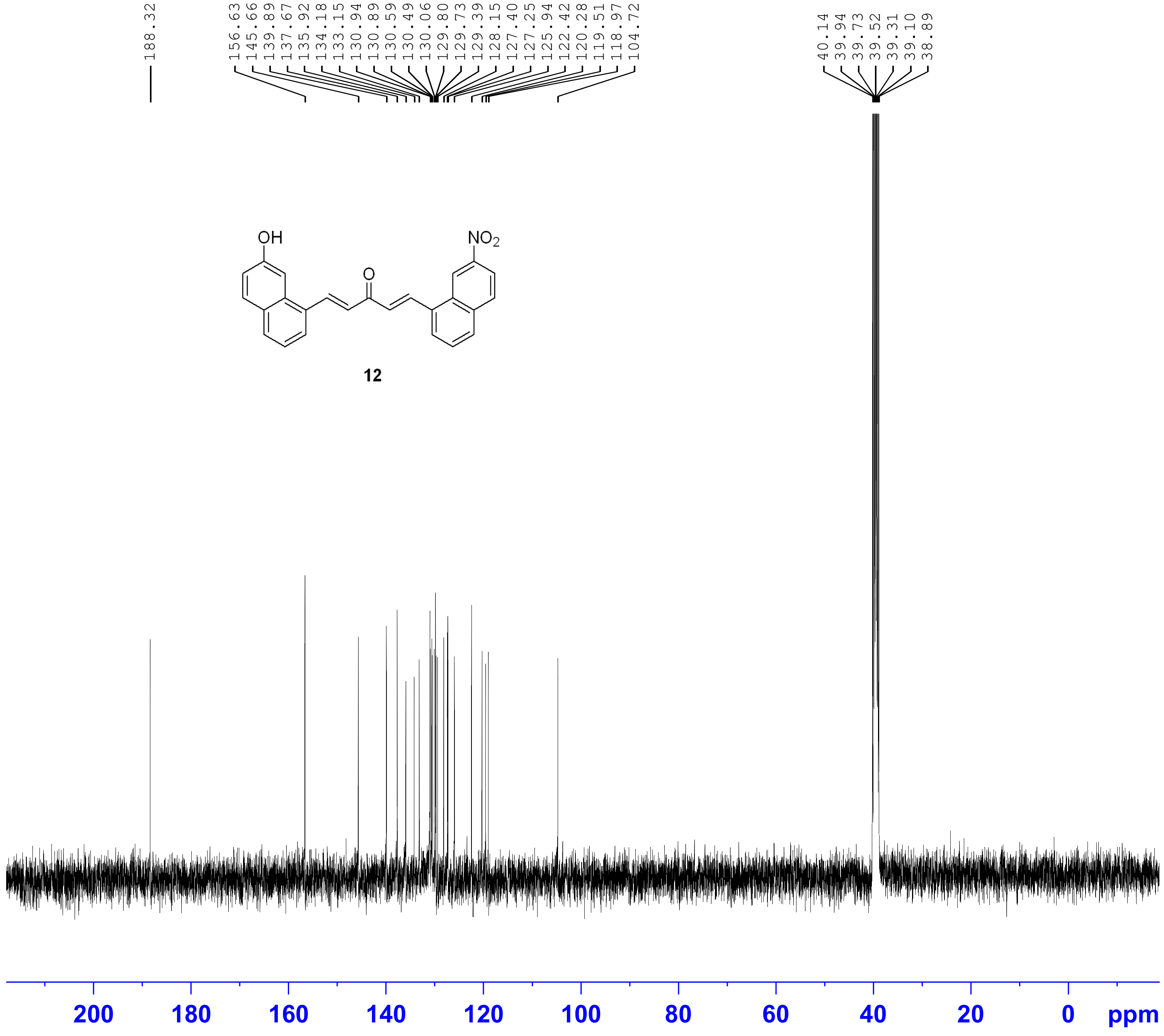

Current Data Parameters
NAME zrh-8-130-c
EXPNO 3
PROCNO 1

F2 - Acquisition Parameters
Date_ 20230201
Time 20.58 h
INSTRUM AvanceNeo 400MHz
PROBHD Z163739_0629 (
PULPROG zgpg30
TD 65536
SOLVENT DMSO
NS 137
DS 4
SWH 23809.523 Hz
FIDRES 0.726609 Hz
AQ 1.3762560 sec
RG 10
DW 21.000 usec
DE 6.50 usec
TE 295.6 K
D1 2.00000000 sec
D11 0.03000000 sec
TD0 1
SFO1 100.6354036 MHz
NUC1 13C
P0 2.67 usec
P1 8.00 usec
PLW1 85.25399780 W
SFO2 400.1816007 MHz
NUC2 1H
CPDPRG[2 waltz65
PCPD2 90.00 usec
PLW2 21.26700020 W
PLW12 0.16802999 W
PLW13 0.08452000 W

F2 - Processing parameters
SI 32768
SF 100.6253822 MHz
WDW EM
SSB 0
LB 1.00 Hz
GB 0
PC 1.40

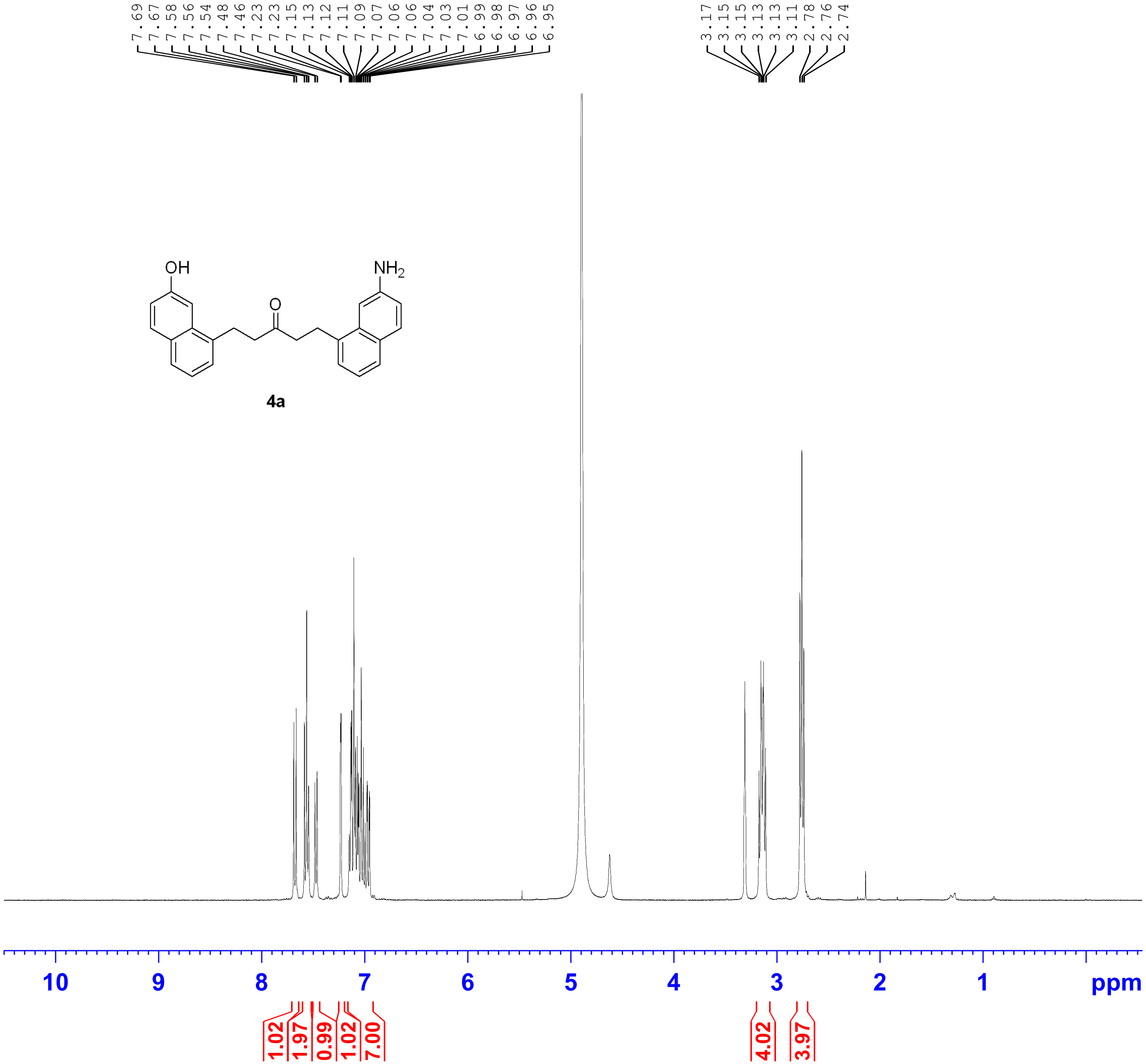


```
Current Data Parameters
NAME      zrh-9-178-white-h
EXPNO                     1
PROCNO                    1

F2 - Acquisition Parameters
Date_              20230705
Time                  16.05 h
INSTRUM   AvanceNeo 400MHz
PROBHD    Z163739_0629 (
PULPROG                zg30
TD                    65536
SOLVENT           CD3OD_SPE
NS                        3
DS                        2
SWH                8196.722 Hz
FIDRES             0.250144 Hz
AQ                3.9976959 sec
RG                      101
DW                   61.000 usec
DE                    13.89 usec
TE                    296.0 K
D1               1.00000000 sec
TD0                       1
SFO1            400.1824711 MHz
NUC1                     1H
P0                     2.67 usec
P1                     8.00 usec
PLW1            21.26700020 W

F2 - Processing parameters
SI                    65536
SF              400.1800075 MHz
WDW                      EM
SSB                       0
LB                     0.30 Hz
GB                        0
PC                     1.00
```

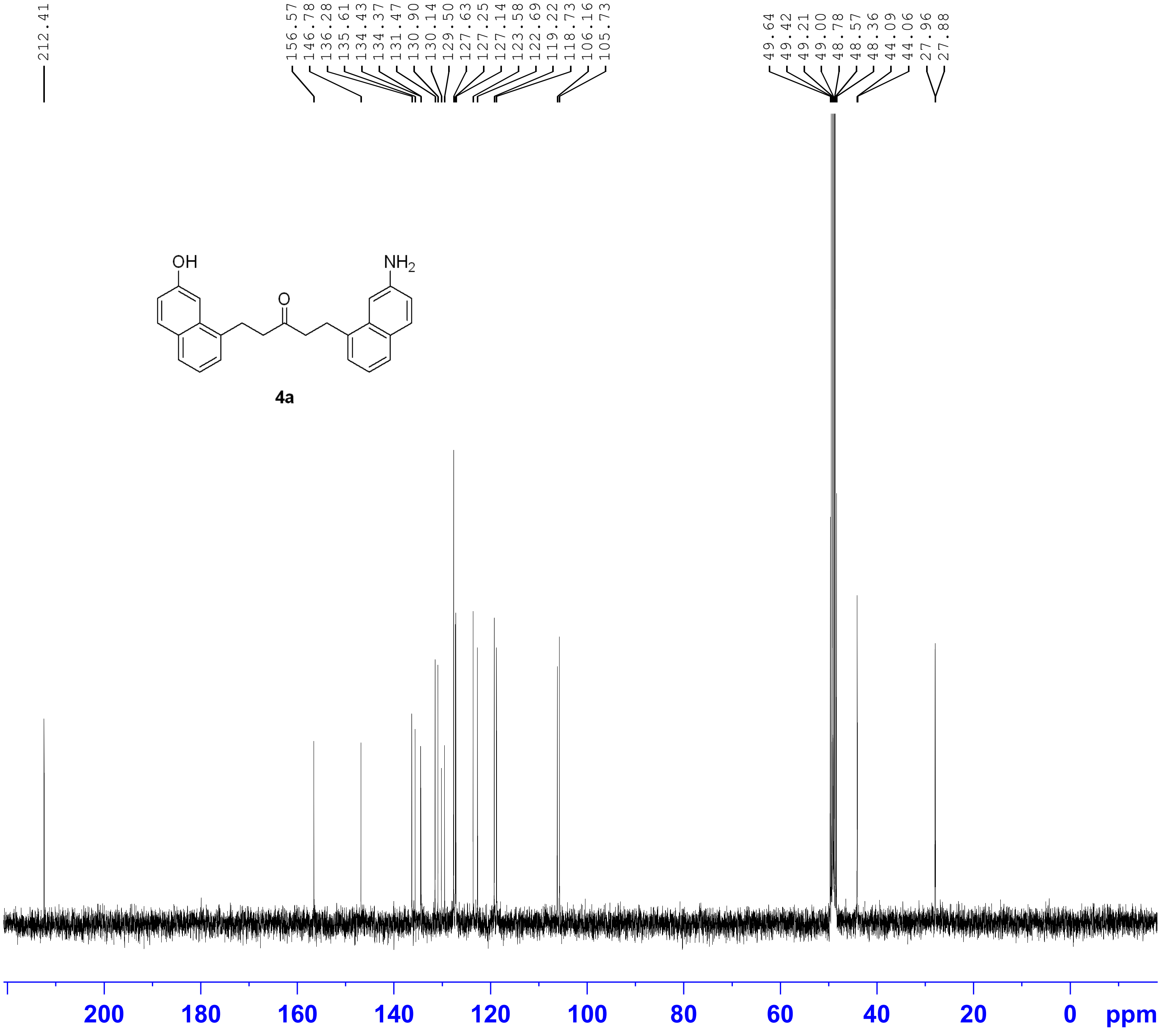

Current Data Parameters
NAME zrh-4-130-big-c
EXPNO 1
PROCNO 1

F2 - Acquisition Parameters
Date_ 20211022
Time 20.18
INSTRUM spect
PROBHD 5 mm DUL 13C-1
PULPROG zgpg30
TD 65536
SOLVENT MeOD
NS 244
DS 0
SWH 24038.461 Hz
FIDRES 0.366798 Hz
AQ 1.3631488 sec
RG 2050
DW 20.800 usec
DE 6.00 usec
TE 293.6 K
D1 2.00000000 sec
D11 0.03000000 sec
TD0 1

======== CHANNEL f1 ========
NUC1 13C
P1 40.00 usec
PL1 -3.00 dB
PL1W 60.64365387 W
SFO1 100.6228298 MHz

======== CHANNEL f2 ========
CPDPRG[2 waltz16
NUC2 1H
PCPD2 80.00 usec
PL2 -1.00 dB
PL12 14.39 dB
PL13 18.00 dB
PL2W 12.17476940 W
PL12W 0.35193357 W
PL13W 0.15327126 W
SFO2 400.1316005 MHz

F2 - Processing parameters
SI 32768
SF 100.6126321 MHz
WDW EM
SSB 0
LB 1.00 Hz
GB 0
PC 1.40

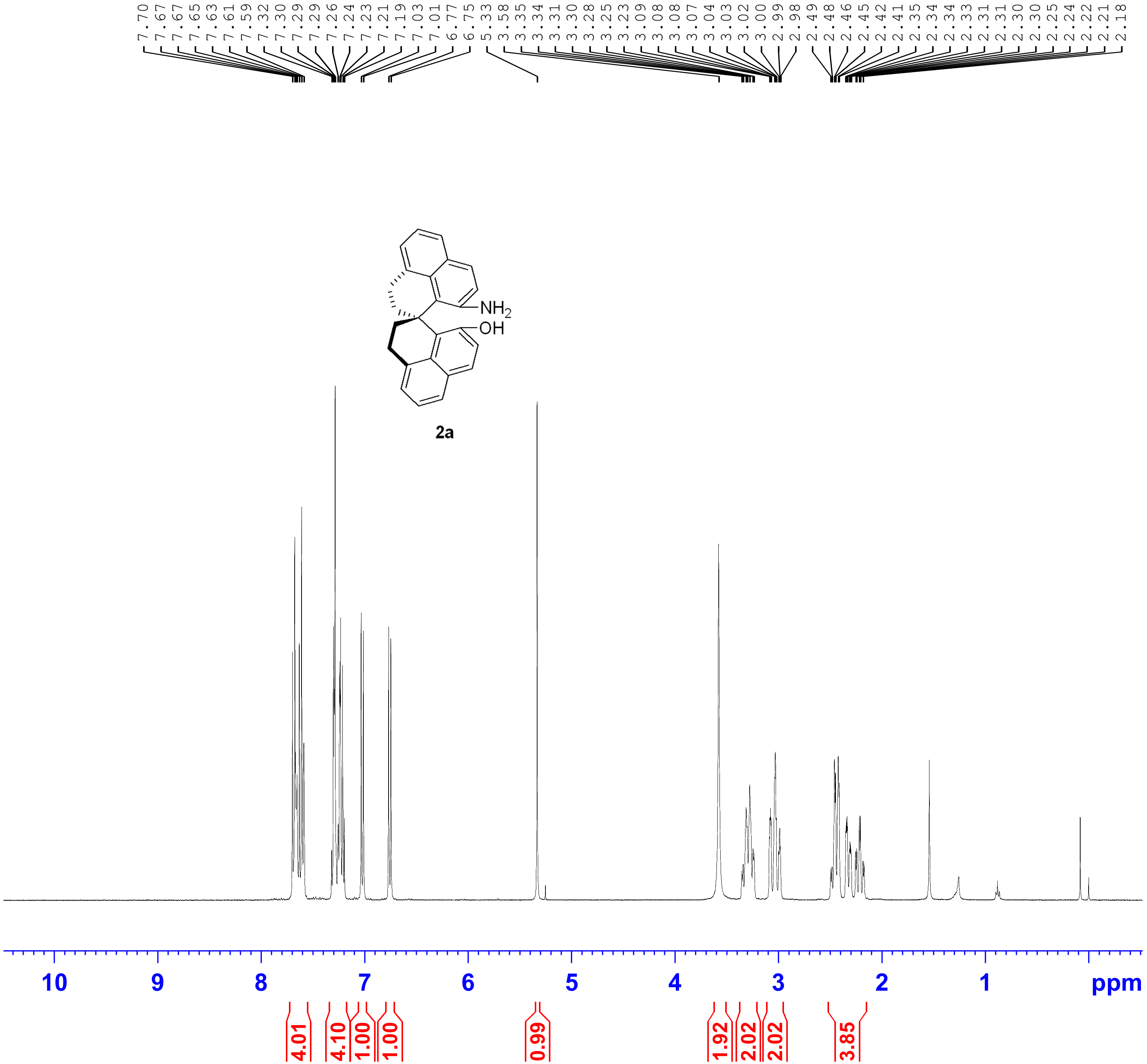

Current Data Parameters
NAME zrh-4-133-NO-h
EXPNO 1
PROCNO 1

F2 - Acquisition Parameters
Date_ 20211023
Time 15.02
INSTRUM spect
PROBHD 5 mm PABBO BB/
PULPROG zg30
TD 65536
SOLVENT CDCl3
NS 3
DS 0
SWH 8012.820 Hz
FIDRES 0.122266 Hz
AQ 4.0894465 sec
RG 39.46
DW 62.400 usec
DE 6.50 usec
TE 296.1 K
D1 1.00000000 sec
TD0 1

======== CHANNEL f1 ========
SFO1 400.1324710 MHz
NUC1 1H
P1 14.50 usec
PLW1 11.99499989 W

F2 - Processing parameters
SI 65536
SF 400.1300311 MHz
WDW EM
SSB 0
LB 0.30 Hz
GB 0
PC 1.00

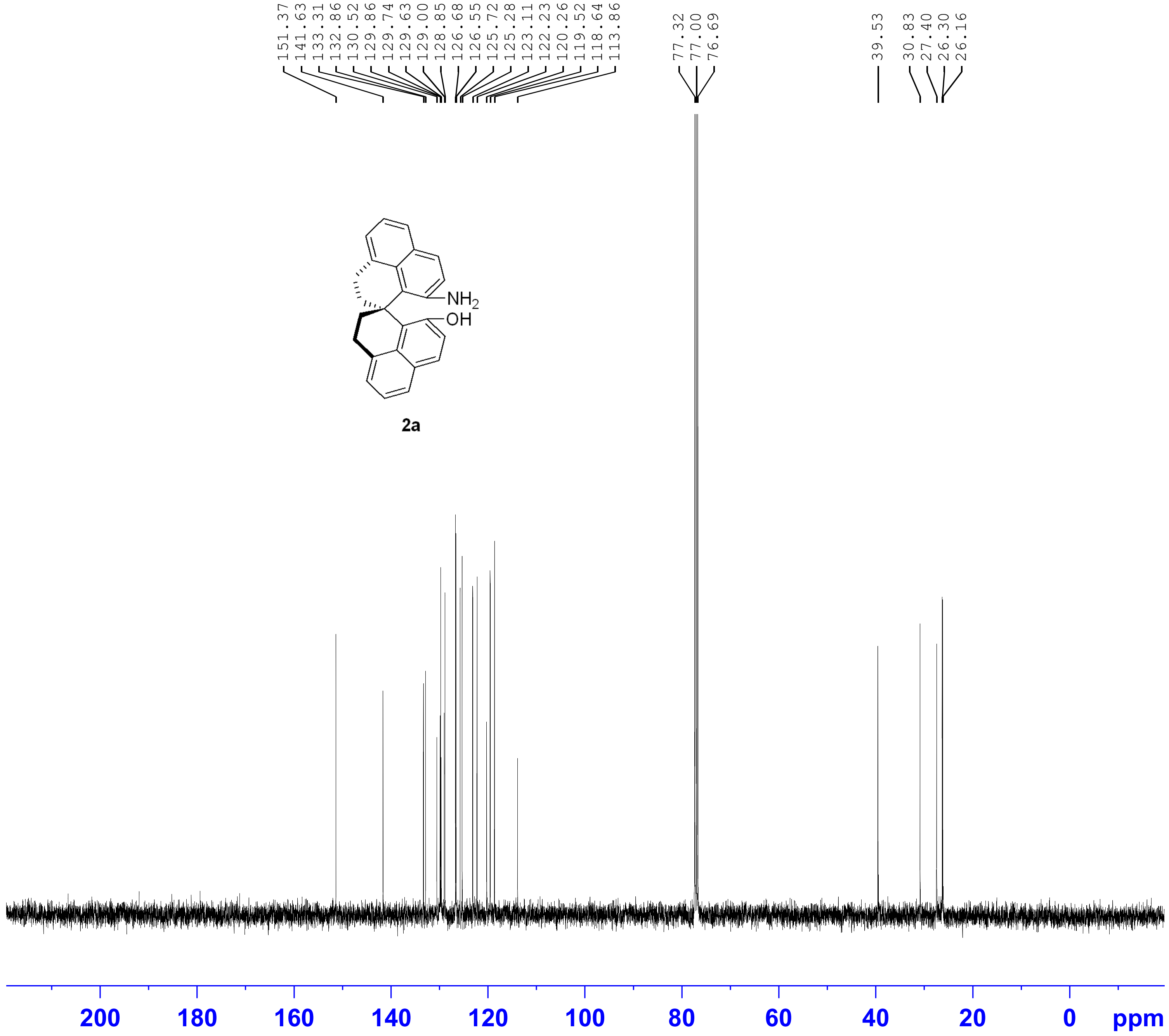


```
Current Data Parameters
NAME       zrh-4-133-NO-c
EXPNO                   1
PROCNO                  1

F2 - Acquisition Parameters
Date_            20211023
Time                15.05
INSTRUM             spect
PROBHD    5 mm PABBO BB/
PULPROG           zgpg30
TD                  65536
SOLVENT             CDCl3
NS                     53
DS                      0
SWH             24038.461 Hz
FIDRES           0.366798 Hz
AQ              1.3631488 sec
RG                 196.92
DW                 20.800 usec
DE                   6.50 usec
TE                  296.8 K
D1             2.00000000 sec
D11            0.03000000 sec
TD0                     1

======== CHANNEL f1 ========
SFO1          100.6228298 MHz
NUC1                  13C
P1                   9.70 usec
PLW1          46.98899841 W

======== CHANNEL f2 ========
SFO2          400.1316005 MHz
NUC2                   1H
CPDPRG[2          waltz16
PCPD2               90.00 usec
PLW2          11.99499989 W
PLW12          0.34213999 W
PLW13          0.27713001 W

F2 - Processing parameters
SI                  32768
SF            100.6127795 MHz
WDW                    EM
SSB                     0
LB                   1.00 Hz
GB                      0
PC                   1.40
```

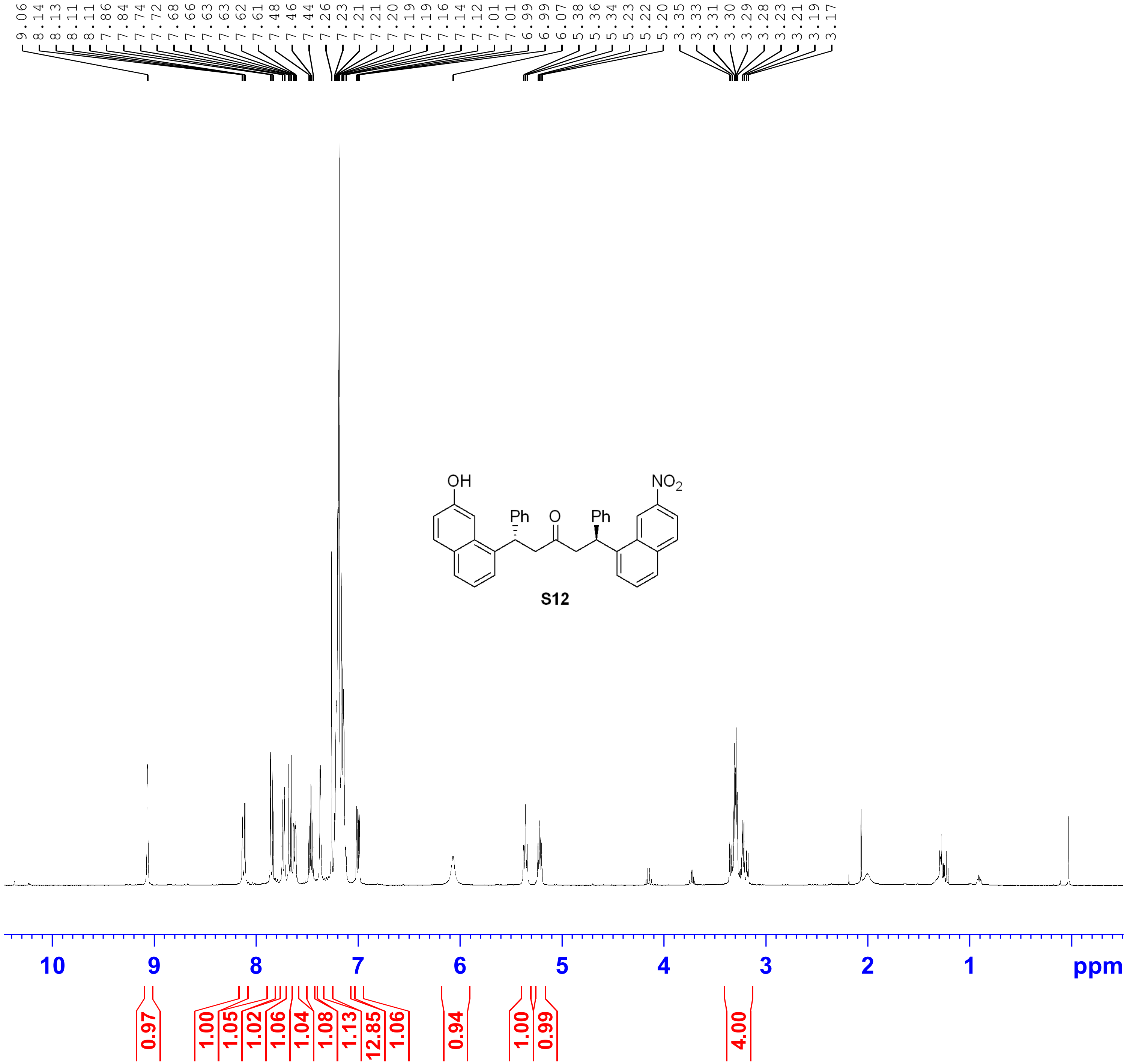

Current Data Parameters
NAME zrh-9-133-re-h
EXPNO 1
PROCNO 1

F2 - Acquisition Parameters
Date_ 20230529
Time 21.56 h
INSTRUM AvanceNeo 400MHz
PROBHD Z163739_0629 (
PULPROG zg30
TD 65536
SOLVENT CDCl3
NS 2
DS 2
SWH 8196.722 Hz
FIDRES 0.250144 Hz
AQ 3.9976959 sec
RG 101
DW 61.000 usec
DE 13.89 usec
TE 296.6 K
D1 1.00000000 sec
TD0 1
SFO1 400.1824711 MHz
NUC1 1H
P0 2.67 usec
P1 8.00 usec
PLW1 21.26700020 W

F2 - Processing parameters
SI 65536
SF 400.1800093 MHz
WDW EM
SSB 0
LB 0.30 Hz
GB 0
PC 1.00

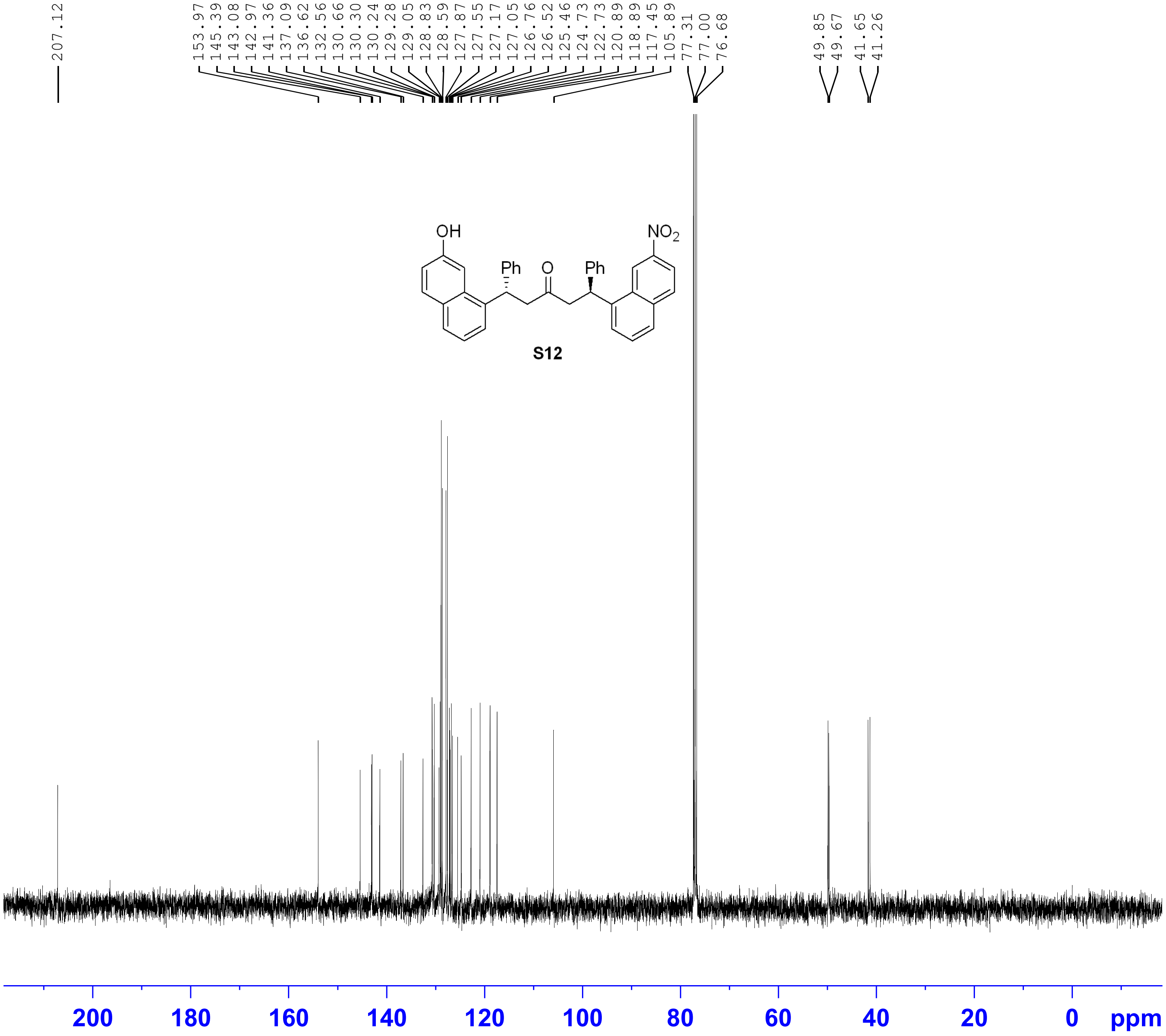

Current Data Parameters
NAME zrh-9-133-re-c
EXPNO 3
PROCNO 1

F2 - Acquisition Parameters
Date_ 20230529
Time 22.01 h
INSTRUM AvanceNeo 400MHz
PROBHD Z163739_0629 (
PULPROG zgpg30
TD 65536
SOLVENT CDCl3
NS 55
DS 4
SWH 23809.523 Hz
FIDRES 0.726609 Hz
AQ 1.3762560 sec
RG 10
DW 21.000 usec
DE 6.50 usec
TE 296.8 K
D1 2.00000000 sec
D11 0.03000000 sec
TD0 1
SFO1 100.6354036 MHz
NUC1 13C
P0 2.67 usec
P1 8.00 usec
PLW1 85.25399780 W
SFO2 400.1816007 MHz
NUC2 1H
CPDPRG[2 waltz65
PCPD2 90.00 usec
PLW2 21.26700020 W
PLW12 0.16802999 W
PLW13 0.08452000 W

F2 - Processing parameters
SI 32768
SF 100.6253506 MHz
WDW EM
SSB 0
LB 1.00 Hz
GB 0
PC 1.40

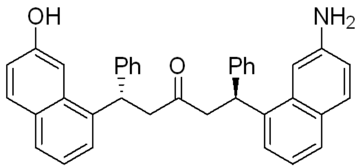

4b

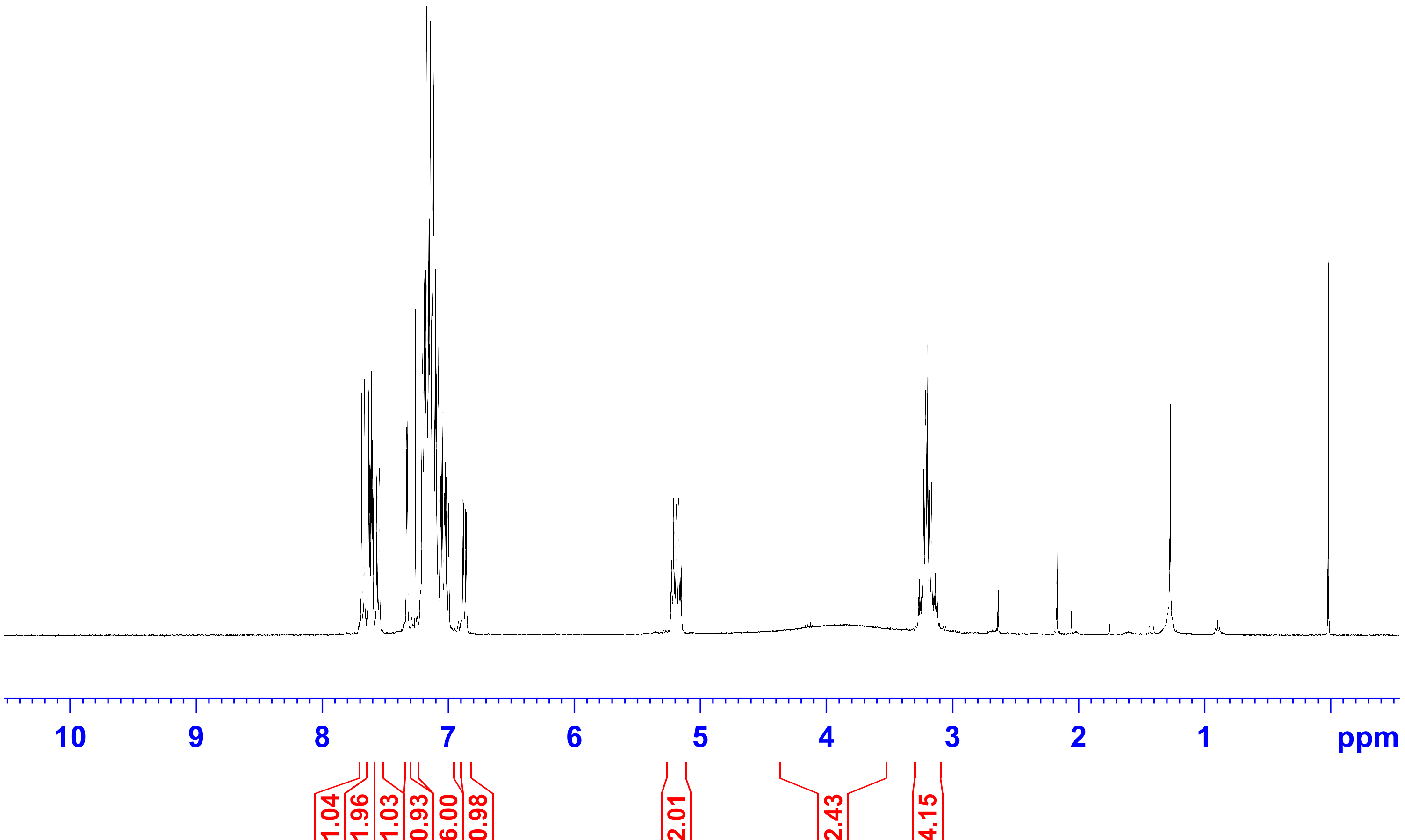


```
Current Data Parameters
NAME          zrh-9-148-h
EXPNO                   1
PROCNO                  1

F2 - Acquisition Parameters
Date_            20230608
Time                22.19
INSTRUM             spect
PROBHD   5 mm PABBO BB/
PULPROG              zg30
TD                  65536
SOLVENT             CDCl3
NS                      3
DS                      2
SWH              8012.820 Hz
FIDRES           0.122266 Hz
AQ              4.0894465 sec
RG                  82.92
DW                 62.400 usec
DE                   6.50 usec
TE                  296.4 K
D1             1.00000000 sec
TD0                     1

======== CHANNEL f1 ========
SFO1          400.1324710 MHz
NUC1                   1H
P1                  14.50 usec
PLW1          11.99499989 W

F2 - Processing parameters
SI                  65536
SF            400.1300103 MHz
WDW                    EM
SSB                     0
LB                   0.30 Hz
GB                      0
PC                   1.00
```

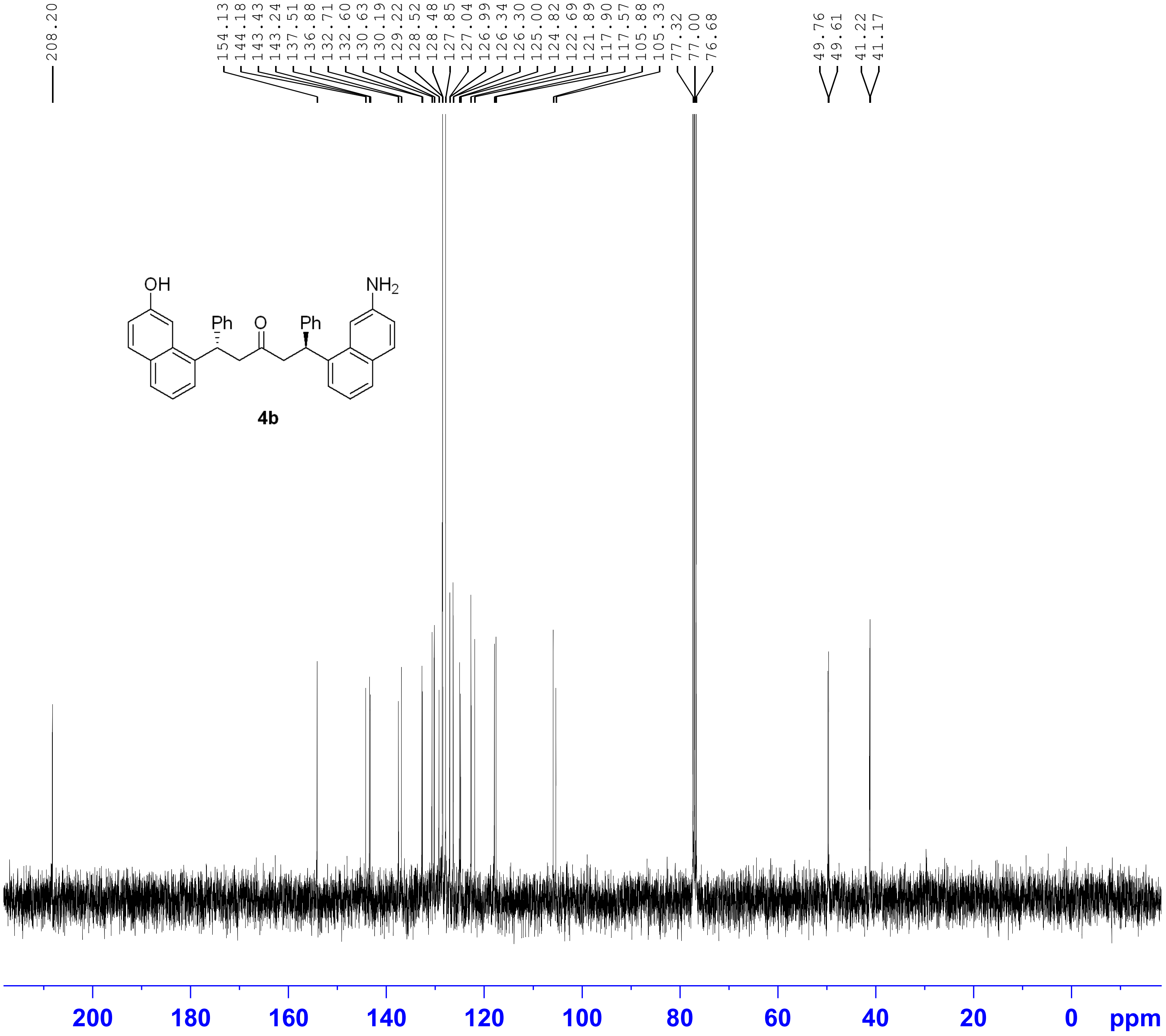

4b

Current Data Parameters
NAME zrh-9-142-re-c
EXPNO 3
PROCNO 1

F2 - Acquisition Parameters
Date_ 20230601
Time 21.27 h
INSTRUM AvanceNeo 400MHz
PROBHD Z163739_0629 (
PULPROG zgpg30
TD 65536
SOLVENT CDCl3
NS 54
DS 4
SWH 23809.523 Hz
FIDRES 0.726609 Hz
AQ 1.3762560 sec
RG 10
DW 21.000 usec
DE 6.50 usec
TE 297.0 K
D1 2.00000000 sec
D11 0.03000000 sec
TD0 1
SFO1 100.6354036 MHz
NUC1 13C
P0 2.67 usec
P1 8.00 usec
PLW1 85.25399780 W
SFO2 400.1816007 MHz
NUC2 1H
CPDPRG[2 waltz65
PCPD2 90.00 usec
PLW2 21.26700020 W
PLW12 0.16802999 W
PLW13 0.08452000 W

F2 - Processing parameters
SI 32768
SF 100.6253514 MHz
WDW EM
SSB 0
LB 1.00 Hz
GB 0
PC 1.40

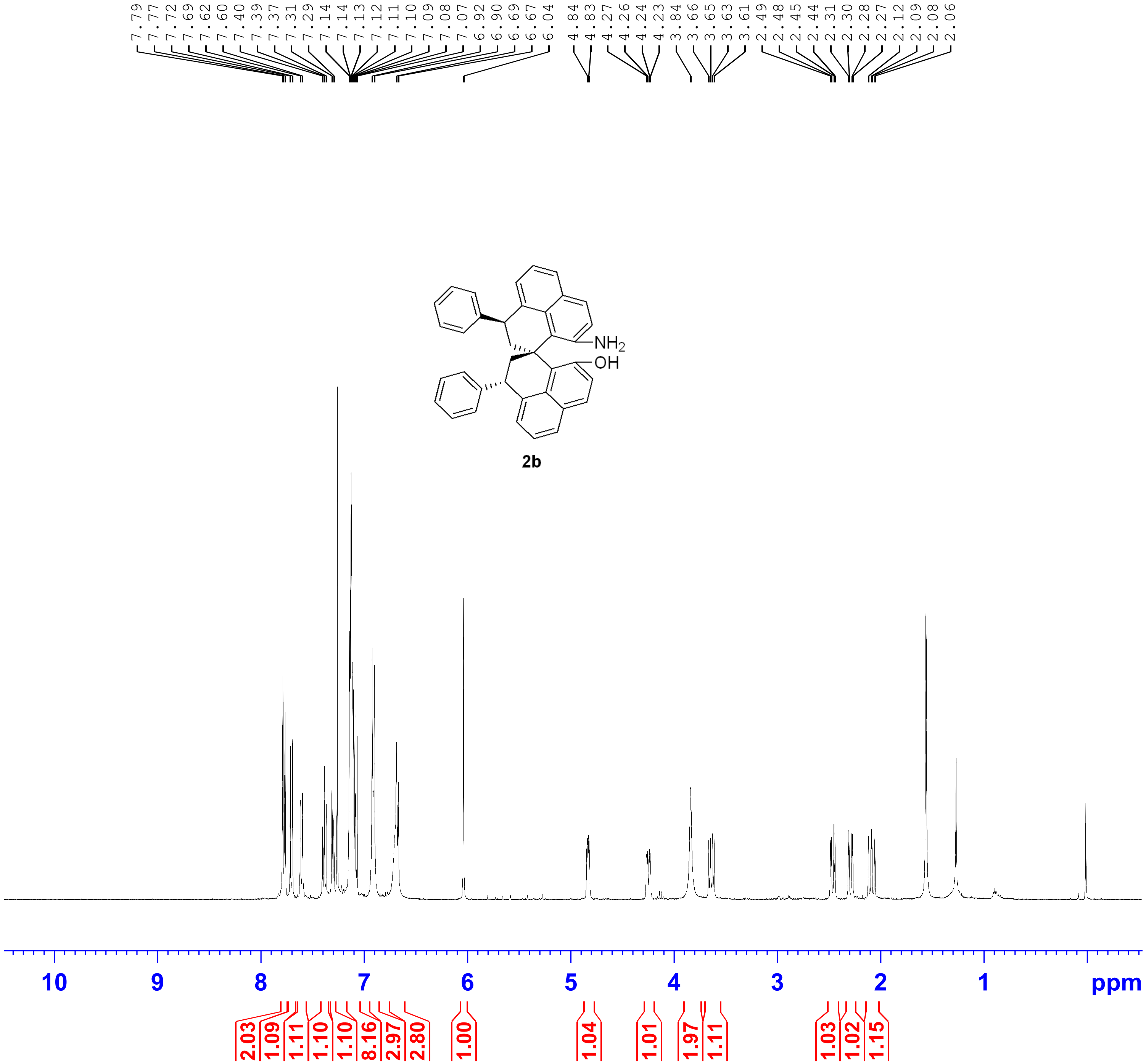

Current Data Parameters
NAME zrh-9-151-h
EXPNO 1
PROCNO 1

F2 - Acquisition Parameters
Date_ 20230609
Time 14.28 h
INSTRUM AvanceNeo 400MHz
PROBHD Z163739_0629 (
PULPROG zg30
TD 65536
SOLVENT CDCl3
NS 8
DS 2
SWH 8196.722 Hz
FIDRES 0.250144 Hz
AQ 3.9976959 sec
RG 101
DW 61.000 usec
DE 13.89 usec
TE 299.8 K
D1 1.00000000 sec
TD0 1
SFO1 400.1824711 MHz
NUC1 1H
P0 2.67 usec
P1 8.00 usec
PLW1 21.26700020 W

F2 - Processing parameters
SI 65536
SF 400.1800093 MHz
WDW EM
SSB 0
LB 0.30 Hz
GB 0
PC 1.00

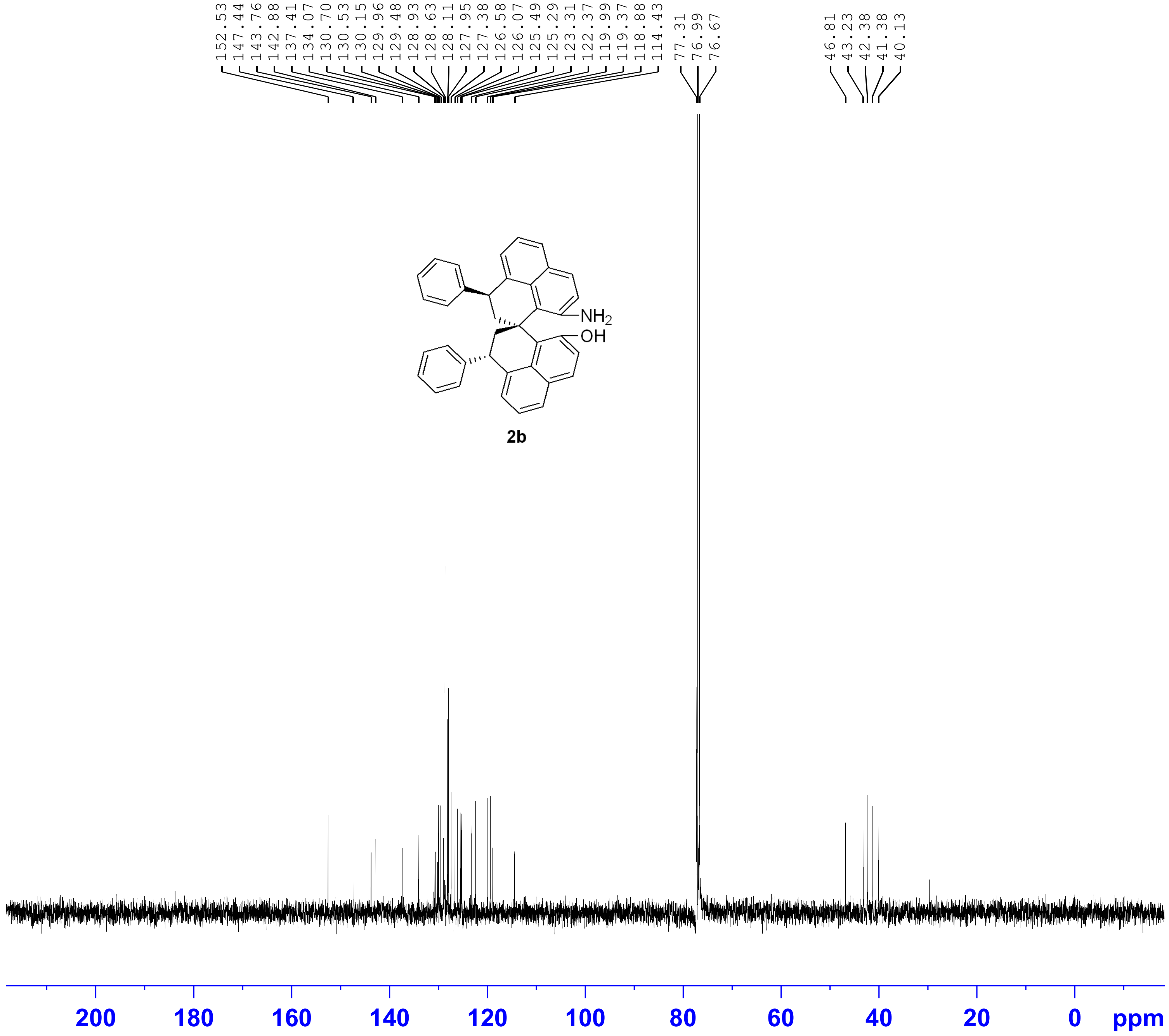


```
Current Data Parameters
NAME          zrh-9-151-c
EXPNO                   3
PROCNO                  1

F2 - Acquisition Parameters
Date_            20230609
Time                14.40 h
INSTRUM   AvanceNeo 400MHz
PROBHD    Z163739_0629 (
PULPROG           zgpg30
TD                 65536
SOLVENT            CDCl3
NS                   180
DS                     4
SWH            23809.523 Hz
FIDRES          0.726609 Hz
AQ             1.3762560 sec
RG                    10
DW                21.000 usec
DE                  6.50 usec
TE                 299.7 K
D1            2.00000000 sec
D11           0.03000000 sec
TD0                    1
SFO1         100.6354036 MHz
NUC1                 13C
P0                  2.67 usec
P1                  8.00 usec
PLW1         85.25399780 W
SFO2         400.1816007 MHz
NUC2                  1H
CPDPRG[2         waltz65
PCPD2              90.00 usec
PLW2         21.26700020 W
PLW12         0.16802999 W
PLW13         0.08452000 W

F2 - Processing parameters
SI                 32768
SF           100.6253448 MHz
WDW                   EM
SSB                    0
LB                  1.00 Hz
GB                     0
PC                  1.40
```

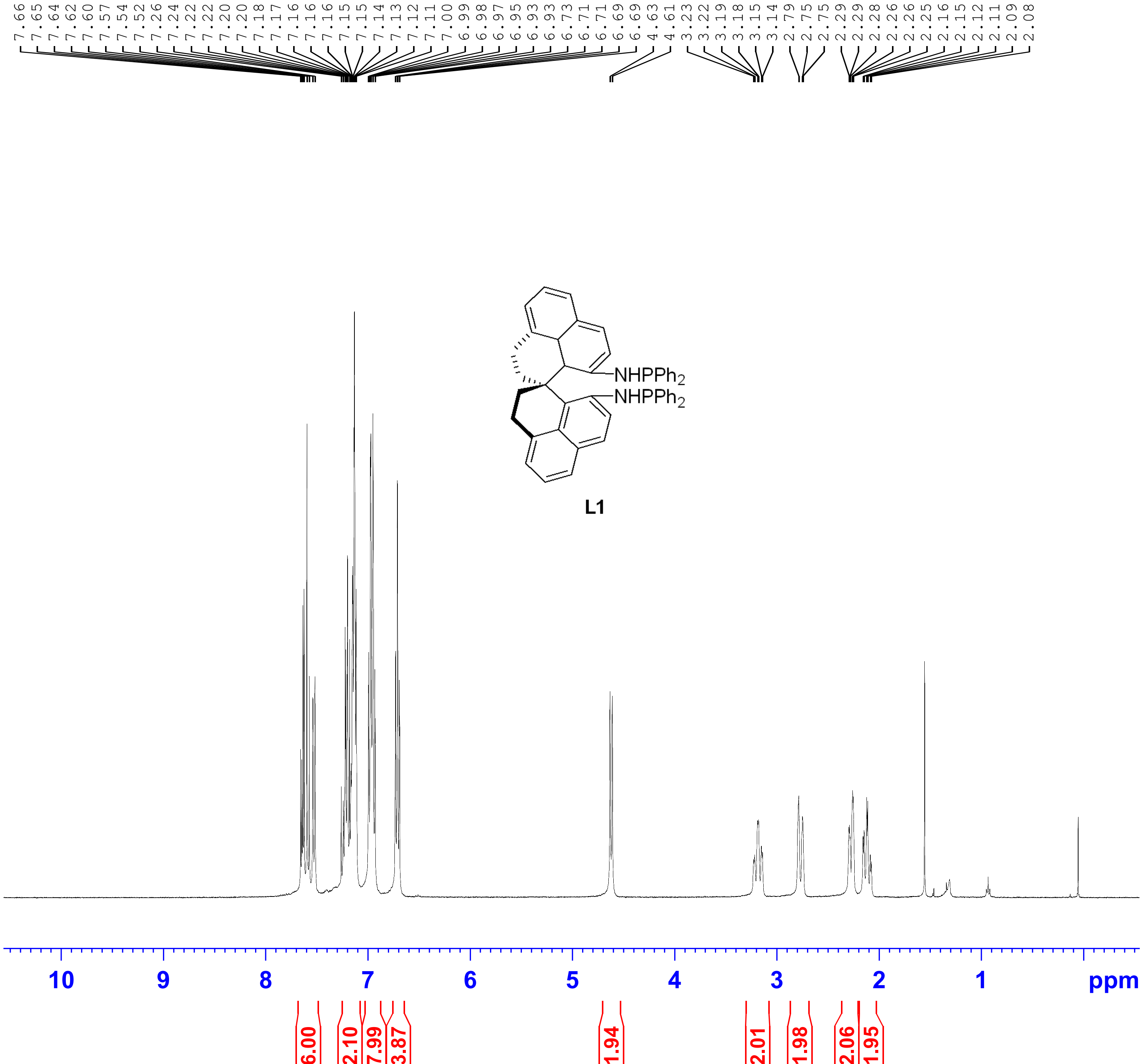

Current Data Parameters

NAME	zrh-4-189-1-pure-h
EXPNO	1
PROCNO	1

F2 - Acquisition Parameters

Date_	20211126
Time	21.46
INSTRUM	spect
PROBHD	5 mm DUL 13C-1
PULPROG	zg30
TD	65536
SOLVENT	CDCl3
NS	1
DS	0
SWH	8223.685 Hz
FIDRES	0.125483 Hz
AQ	3.9845889 sec
RG	161
DW	60.800 usec
DE	6.00 usec
TE	294.1 K
D1	1.00000000 sec
TD0	1

======== CHANNEL f1 ========

NUC1	1H
P1	15.80 usec
PL1	-1.00 dB
PL1W	12.17476940 W
SFO1	400.1324710 MHz

F2 - Processing parameters

SI	32768
SF	400.1300096 MHz
WDW	EM
SSB	0
LB	0.30 Hz
GB	0
PC	1.00

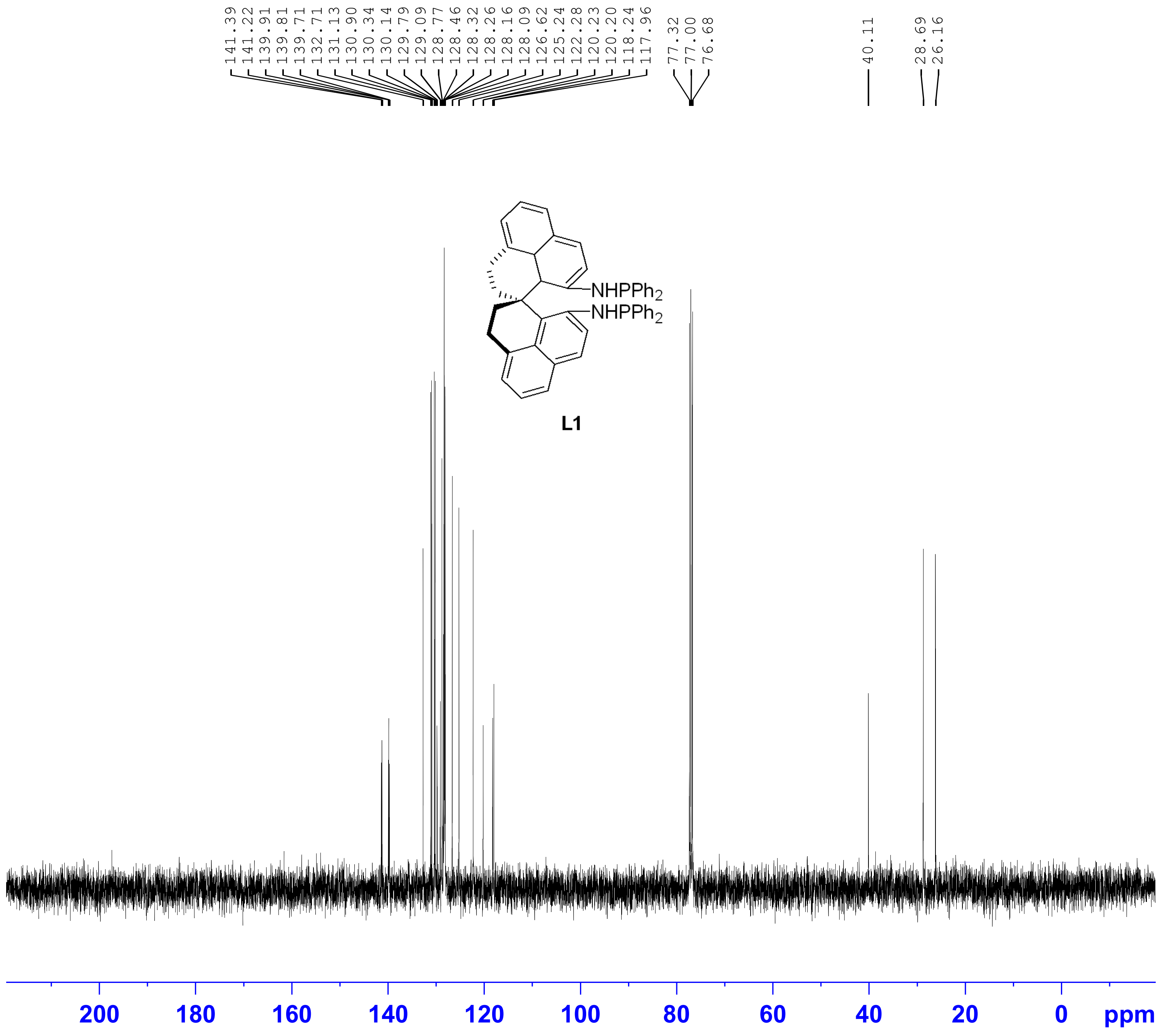

Current Data Parameters
NAME zrh-4-189-1-pure-c
EXPNO 1
PROCNO 1

F2 - Acquisition Parameters
Date_ 20211126
Time 21.47
INSTRUM spect
PROBHD 5 mm DUL 13C-1
PULPROG zgpg30
TD 65536
SOLVENT CDCl3
NS 73
DS 0
SWH 24038.461 Hz
FIDRES 0.366798 Hz
AQ 1.3631488 sec
RG 2050
DW 20.800 usec
DE 6.00 usec
TE 294.3 K
D1 2.00000000 sec
D11 0.03000000 sec
TD0 1

======== CHANNEL f1 ========
NUC1 13C
P1 40.00 usec
PL1 -3.00 dB
PL1W 60.64365387 W
SFO1 100.6228298 MHz

======== CHANNEL f2 ========
CPDPRG[2 waltz16
NUC2 1H
PCPD2 80.00 usec
PL2 -1.00 dB
PL12 14.39 dB
PL13 18.00 dB
PL2W 12.17476940 W
PL12W 0.35193357 W
PL13W 0.15327126 W
SFO2 400.1316005 MHz

F2 - Processing parameters
SI 32768
SF 100.6127788 MHz
WDW EM
SSB 0
LB 1.00 Hz
GB 0
PC 1.40

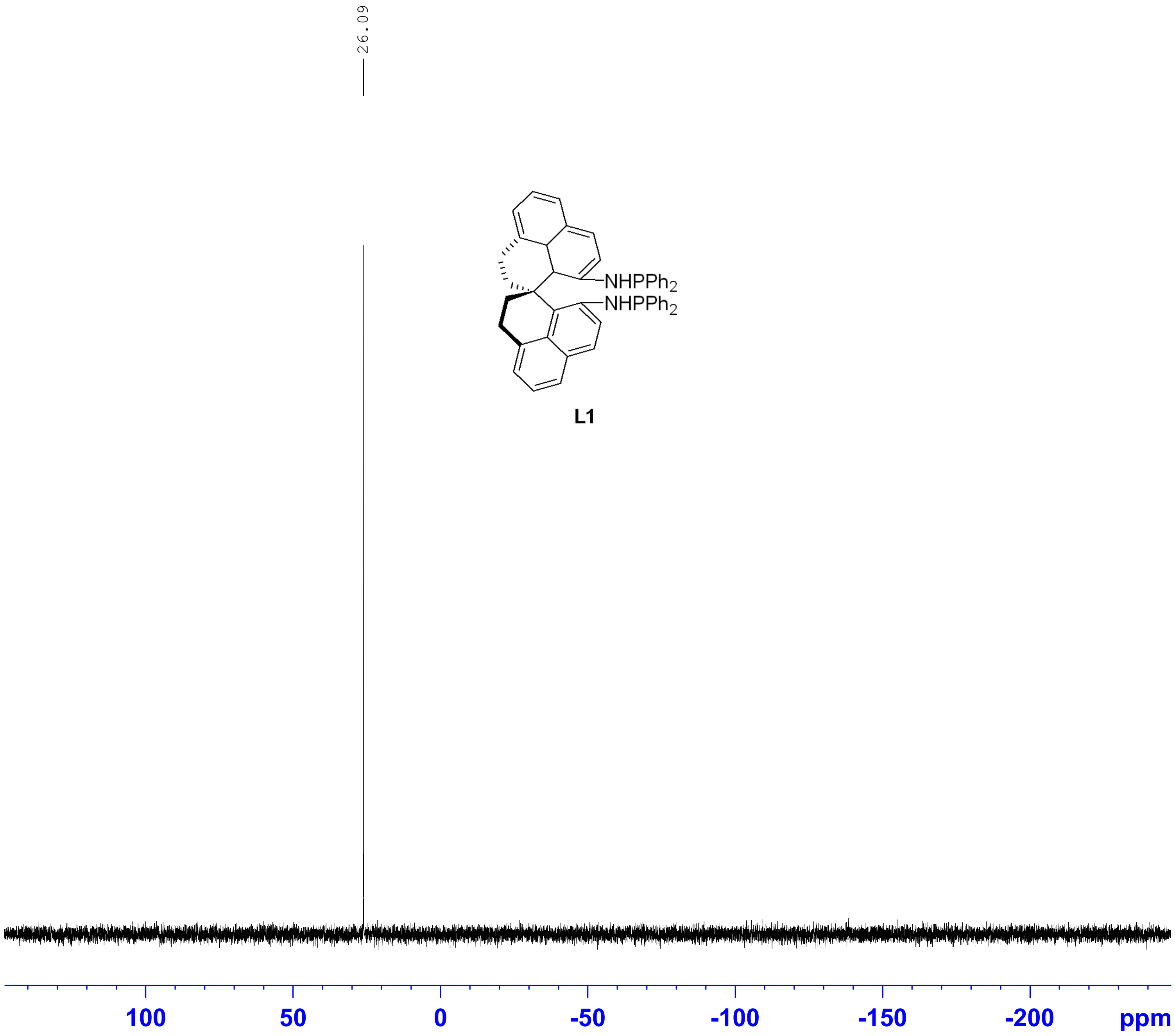


```
Current Data Parameters
NAME      zrh-5-55-bis-p
EXPNO                 12
PROCNO                 1

F2 - Acquisition Parameters
Date_           20220103
Time               10.05
INSTRUM            spect
PROBHD    5 mm PABBO BB/
PULPROG           zgpg30
TD                 65536
SOLVENT            CDCl3
NS                     4
DS                     0
SWH            64102.563 Hz
FIDRES          0.978127 Hz
AQ             0.5111808 sec
RG                196.92
DW                 7.800 usec
DE                  6.50 usec
TE                 295.5 K
D1            2.00000000 sec
D11           0.03000000 sec
TD0                    1

======== CHANNEL f1 ========
SFO1         161.9674942 MHz
NUC1                 31P
P1                 14.70 usec
PLW1         11.99499989 W

======== CHANNEL f2 ========
SFO2         400.1316005 MHz
NUC2                  1H
CPDPRG[2         waltz16
PCPD2              90.00 usec
PLW2         11.99499989 W
PLW12         0.34213999 W
PLW13         0.27713001 W

F2 - Processing parameters
SI                 32768
SF           161.9755930 MHz
WDW                   EM
SSB                    0
LB                  1.00 Hz
GB                     0
PC                  1.40
```

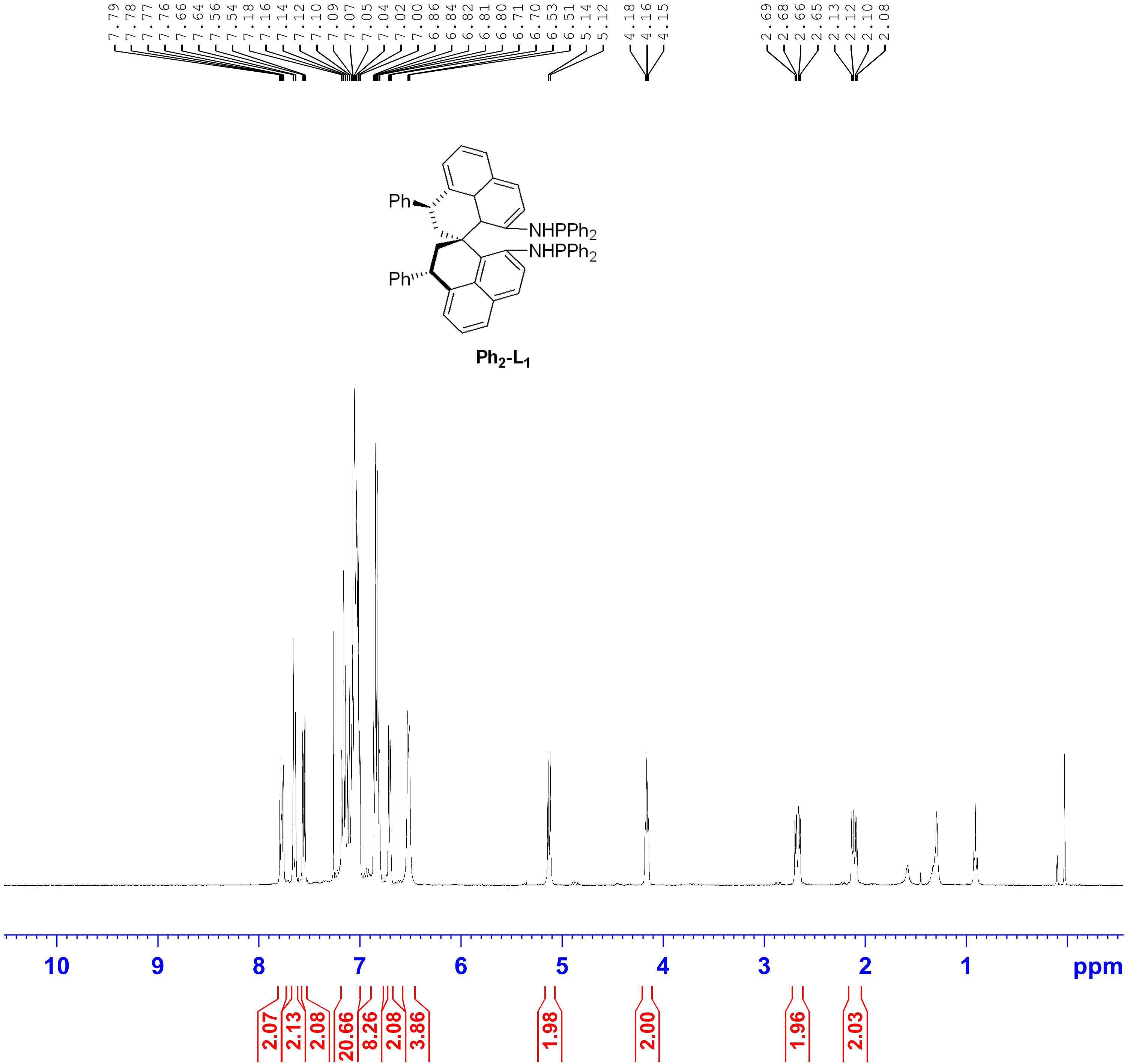

Current Data Parameters	
NAME	zmh-0909-3
EXPNO	1
PROCNO	1

F2 - Acquisition Parameters	
Date_	20250908
Time	22.16 h
INSTRUM	AvanceNeo 400MHz
PROBHD	Z163739_0629 (
PULPROG	zg30
TD	65536
SOLVENT	CDCl3
NS	8
DS	2
SWH	8196.722 Hz
FIDRES	0.250144 Hz
AQ	3.9976959 sec
RG	101
DW	61.000 usec
DE	13.89 usec
TE	297.7 K
D1	1.00000000 sec
TD0	1
SFO1	400.1824711 MHz
NUC1	1H
P0	2.67 usec
P1	8.00 usec
PLW1	21.26700020 W

F2 - Processing parameters	
SI	65536
SF	400.1800097 MHz
WDW	EM
SSB	0
LB	0.30 Hz
GB	0
PC	1.00

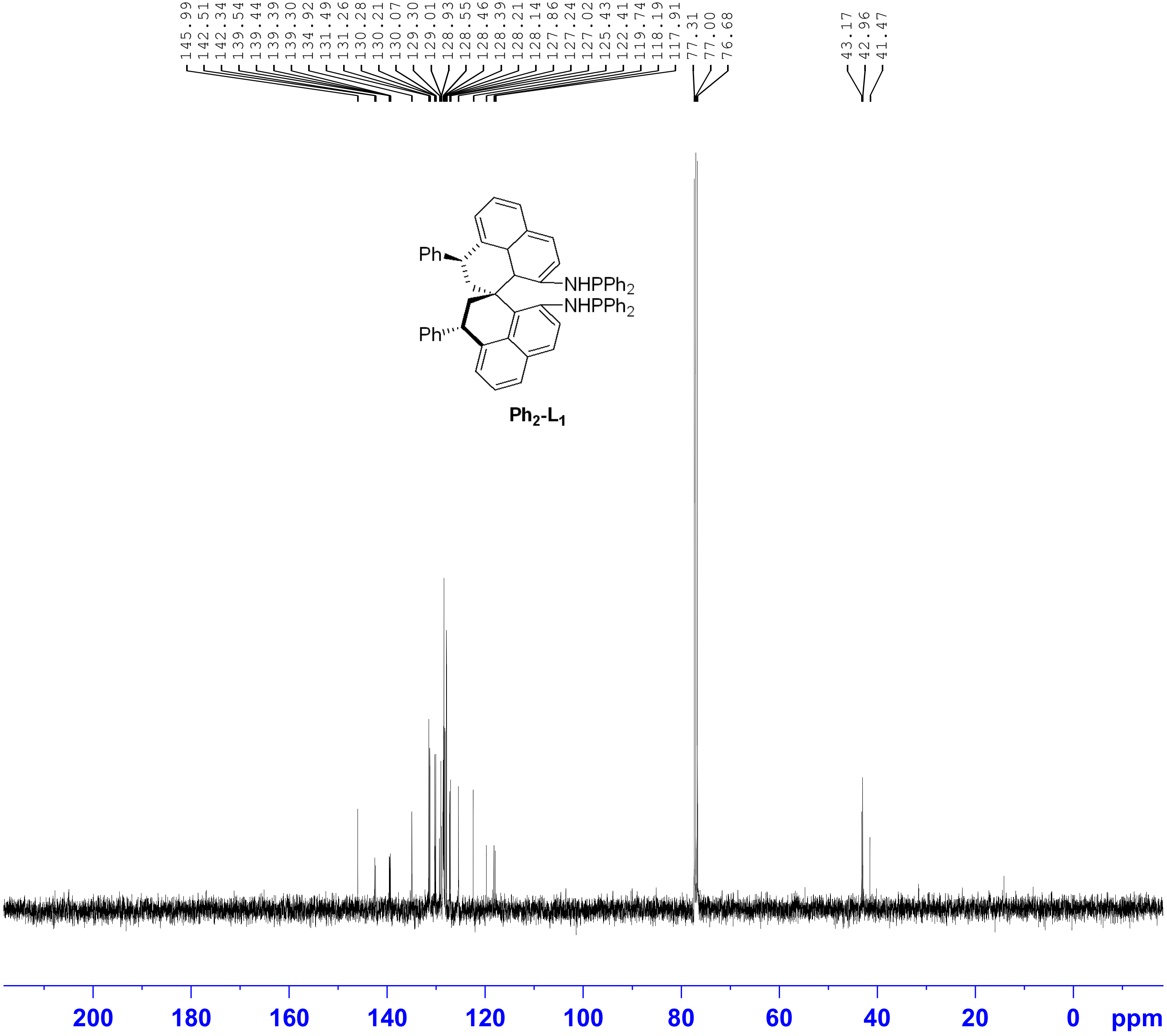

Ph_2-L_1

Current Data Parameters
NAME zmh-0909-3
EXPNO 3
PROCNO 1

F2 - Acquisition Parameters
Date_ 20250908
Time 22.26 h
INSTRUM AvanceNeo 400MHz
PROBHD Z163739_0629 (
PULPROG zgpg30
TD 65536
SOLVENT CDCl3
NS 53
DS 4
SWH 23809.523 Hz
FIDRES 0.726609 Hz
AQ 1.3762560 sec
RG 10
DW 21.000 usec
DE 6.50 usec
TE 298.0 K
D1 2.00000000 sec
D11 0.03000000 sec
TD0 1
SFO1 100.6354036 MHz
NUC1 13C
P0 2.67 usec
P1 8.00 usec
PLW1 85.25399780 W
SFO2 400.1816007 MHz
NUC2 1H
CPDPRG[2 waltz65
PCPD2 90.00 usec
PLW2 21.26700020 W
PLW12 0.16802999 W
PLW13 0.08452000 W

F2 - Processing parameters
SI 32768
SF 100.6253469 MHz
WDW EM
SSB 0
LB 1.00 Hz
GB 0
PC 1.40

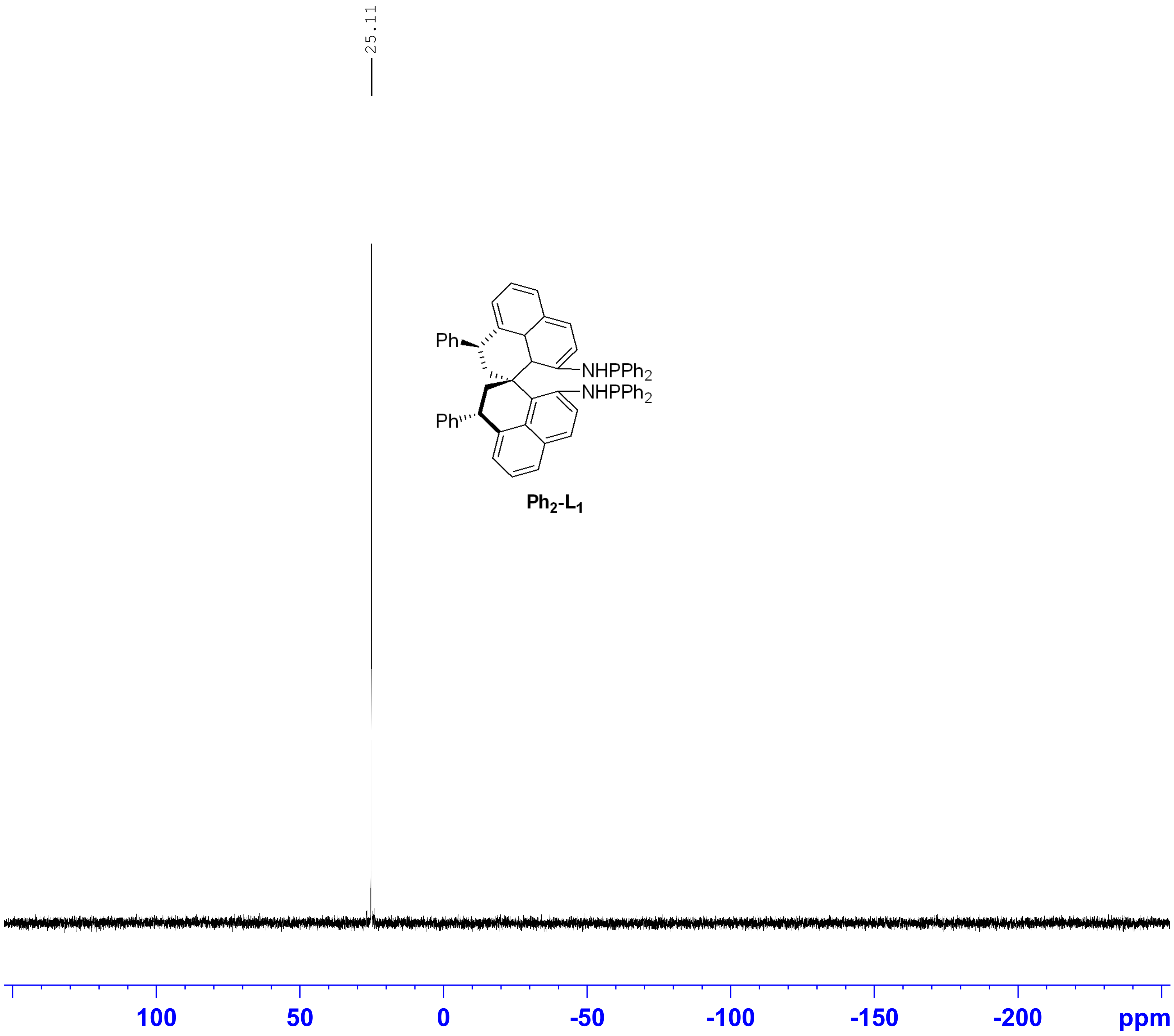

Ph_2-L_1

Current Data Parameters
NAME zmh-0909-3
EXPNO 2
PROCNO 1

F2 - Acquisition Parameters
Date_ 20250908
Time 22.21 h
INSTRUM AvanceNeo 400MHz
PROBHD Z163739_0629 (
PULPROG zg30
TD 65536
SOLVENT CDCl3
NS 32
DS 4
SWH 65789.477 Hz
FIDRES 2.007735 Hz
AQ 0.4980736 sec
RG 101
DW 7.600 usec
DE 6.50 usec
TE 297.6 K
D1 2.00000000 sec
TD0 1
SFO1 161.9877335 MHz
NUC1 31P
P0 2.67 usec
P1 8.00 usec
PLW1 56.00000000 W

F2 - Processing parameters
SI 32768
SF 161.9958333 MHz
WDW EM
SSB 0
LB 1.00 Hz
GB 0
PC 1.40

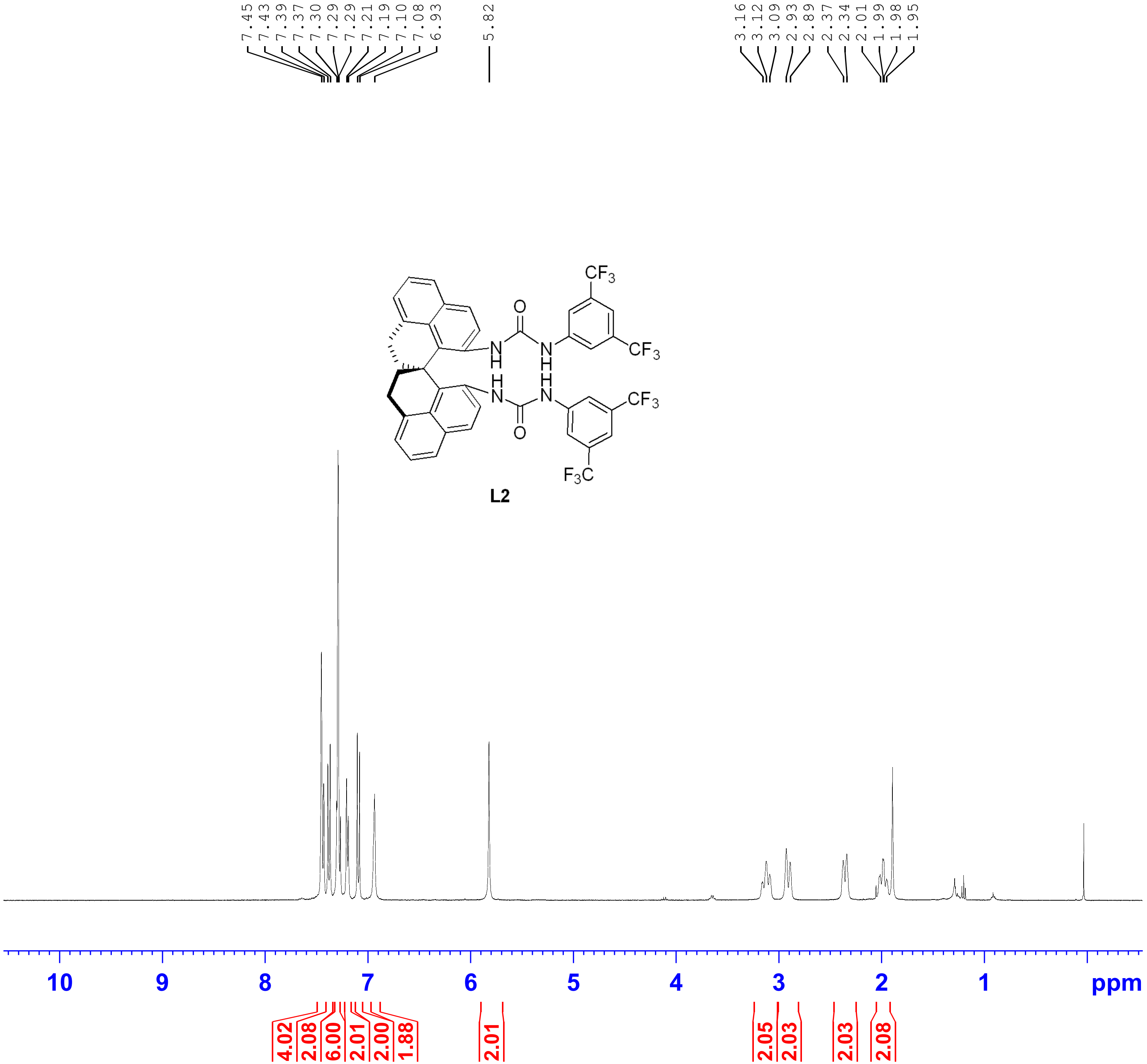


```
Current Data Parameters
NAME           zrh-8-124-2-h
EXPNO                      1
PROCNO                     1

F2 - Acquisition Parameters
Date_               20230109
Time                   19.33
INSTRUM                spect
PROBHD     5 mm PABBO BB/
PULPROG                 zg30
TD                     65536
SOLVENT                CDCl3
NS                         5
DS                         2
SWH                 8012.820 Hz
FIDRES              0.122266 Hz
AQ                 4.0894465 sec
RG                     82.92
DW                    62.400 usec
DE                      6.50 usec
TE                     295.0 K
D1                1.00000000 sec
TD0                        1

======== CHANNEL f1 ========
SFO1             400.1324710 MHz
NUC1                      1H
P1                     14.50 usec
PLW1             11.99499989 W

F2 - Processing parameters
SI                     65536
SF               400.1300000 MHz
WDW                       EM
SSB                        0
LB                      0.30 Hz
GB                         0
PC                      1.00
```

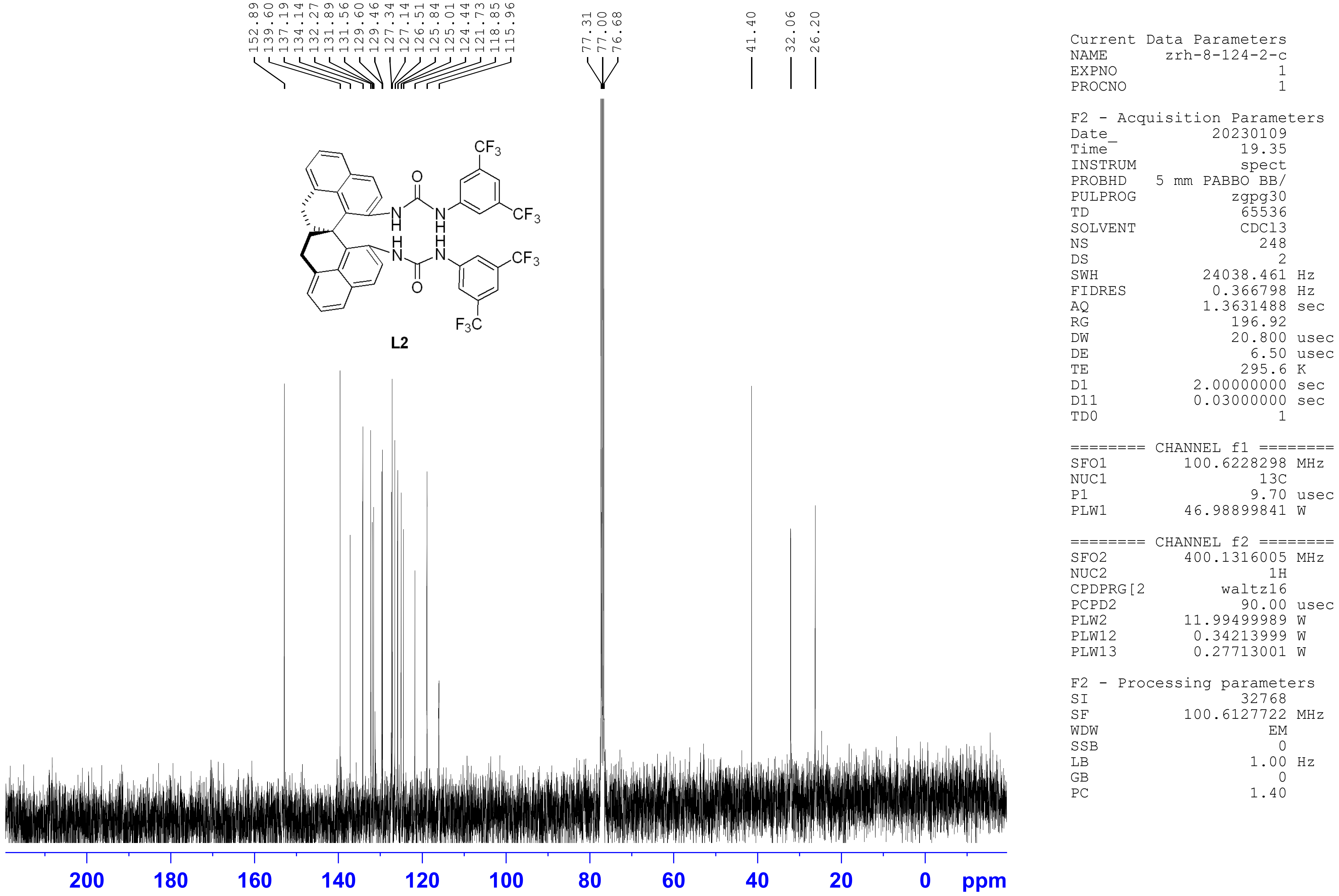
152.89
139.60
137.19
134.14
132.27
131.89
131.56
129.60
129.46
127.34
127.14
126.51
125.84
125.01
124.44
121.73
118.85
115.96
77.31
77.00
76.68
41.40
32.06
26.20
L2
200
180
160
140
120
100
80
60
40
20
0
ppm
Current Data Parameters
NAME zrh-8-124-2-c
EXPNO 1
PROCNO 1
F2 - Acquisition Parameters
Date_ 20230109
Time 19.35
INSTRUM spect
PROBHD 5 mm PABBO BB/
PULPROG zgpg30
TD 65536
SOLVENT CDCl3
NS 248
DS 2
SWH 24038.461 Hz
FIDRES 0.366798 Hz
AQ 1.3631488 sec
RG 196.92
DW 20.800 usec
DE 6.50 usec
TE 295.6 K
D1 2.00000000 sec
D11 0.03000000 sec
TD0 1
======== CHANNEL f1 ========
SFO1 100.6228298 MHz
NUC1 13C
P1 9.70 usec
PLW1 46.98899841 W
======== CHANNEL f2 ========
SFO2 400.1316005 MHz
NUC2 1H
CPDPRG[2 waltz16
PCPD2 90.00 usec
PLW2 11.99499989 W
PLW12 0.34213999 W
PLW13 0.27713001 W
F2 - Processing parameters
SI 32768
SF 100.6127722 MHz
WDW EM
SSB 0
LB 1.00 Hz
GB 0
PC 1.40

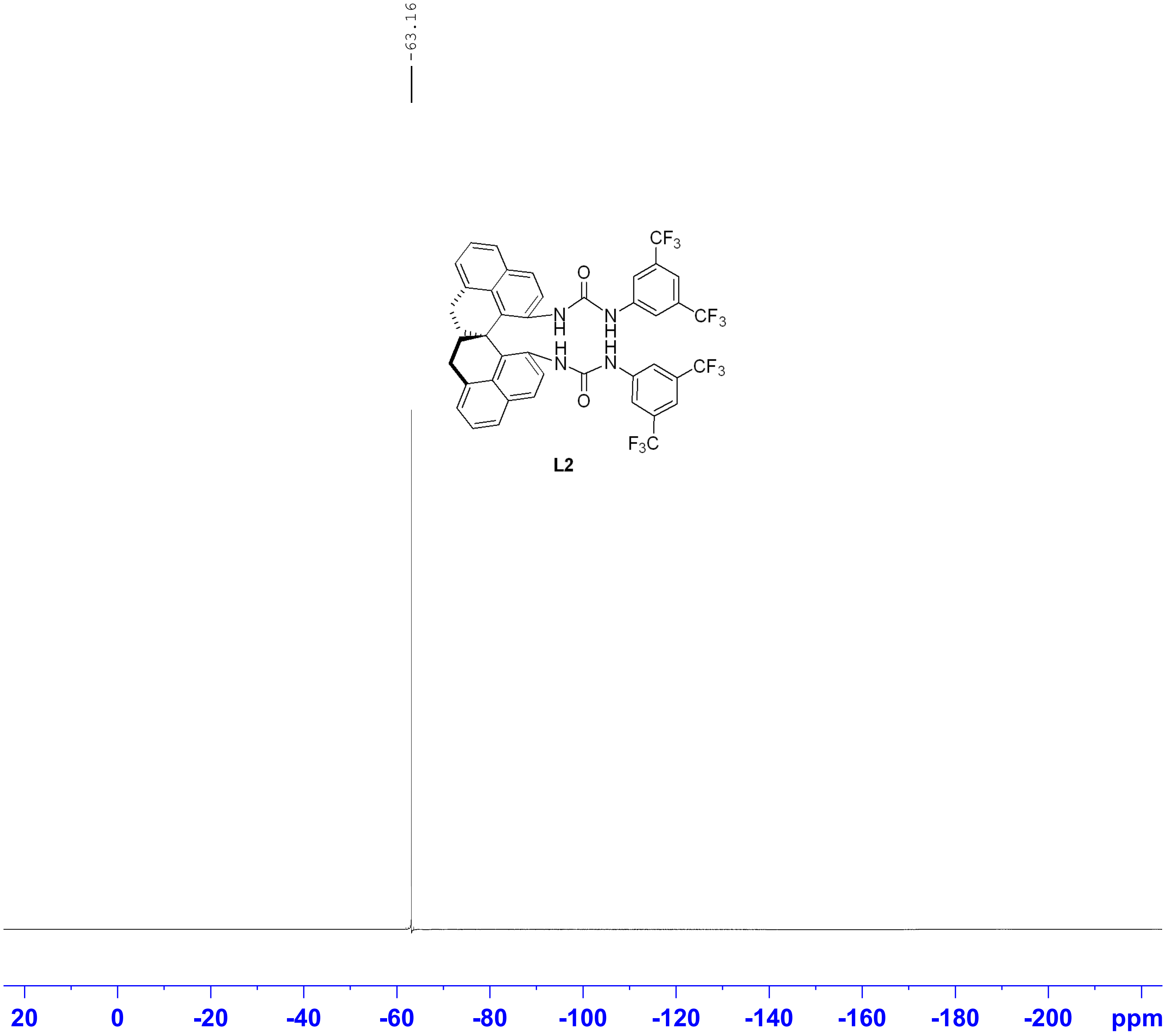

L2

Current Data Parameters

NAME	zrh-8-124-2-f
EXPNO	2
PROCNO	1

F2 - Acquisition Parameters

Date_	20230109
Time	19.50
INSTRUM	spect
PROBHD	5 mm PABBO BB/
PULPROG	zgpg30
TD	65536
SOLVENT	CDCl3
NS	10
DS	2
SWH	93750.000 Hz
FIDRES	1.430511 Hz
AQ	0.3495253 sec
RG	196.92
DW	5.333 usec
DE	6.50 usec
TE	295.6 K
D1	2.00000000 sec
D11	0.03000000 sec
TD0	1

======== CHANNEL f1 ========

SFO1	376.4607162 MHz
NUC1	19F
P1	14.70 usec
PLW1	15.99600029 W

======== CHANNEL f2 ========

SFO2	400.1316005 MHz
NUC2	1H
CPDPRG[2	waltz16
PCPD2	90.00 usec
PLW2	11.99499989 W
PLW12	0.34213999 W
PLW13	0.27713001 W

F2 - Processing parameters

SI	32768
SF	376.4983660 MHz
WDW	EM
SSB	0
LB	1.00 Hz
GB	0
PC	1.40

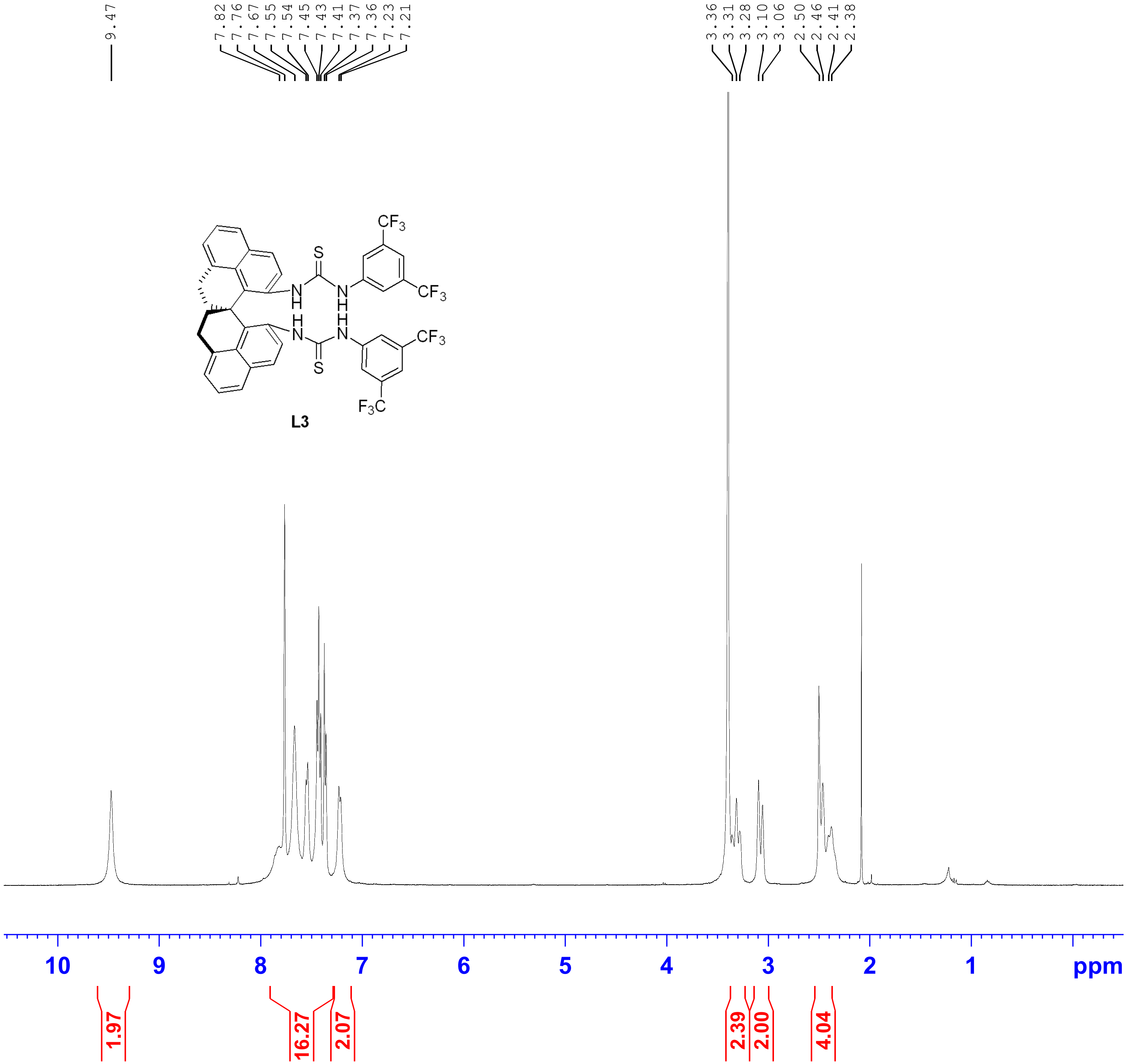

L3

Current Data Parameters
NAME zrh-8-123-re-h
EXPNO 11
PROCNO 1

F2 - Acquisition Parameters
Date_ 20230110
Time 19.38 h
INSTRUM AvanceNeo 400MHz
PROBHD Z163739_0629 (
PULPROG zg30
TD 65536
SOLVENT DMSO
NS 4
DS 2
SWH 8196.722 Hz
FIDRES 0.250144 Hz
AQ 3.9976959 sec
RG 101
DW 61.000 usec
DE 13.89 usec
TE 295.4 K
D1 1.00000000 sec
TD0 1
SFO1 400.1824711 MHz
NUC1 1H
P0 2.67 usec
P1 8.00 usec
PLW1 21.26700020 W

F2 - Processing parameters
SI 65536
SF 400.1800025 MHz
WDW EM
SSB 0
LB 0.30 Hz
GB 0
PC 1.00

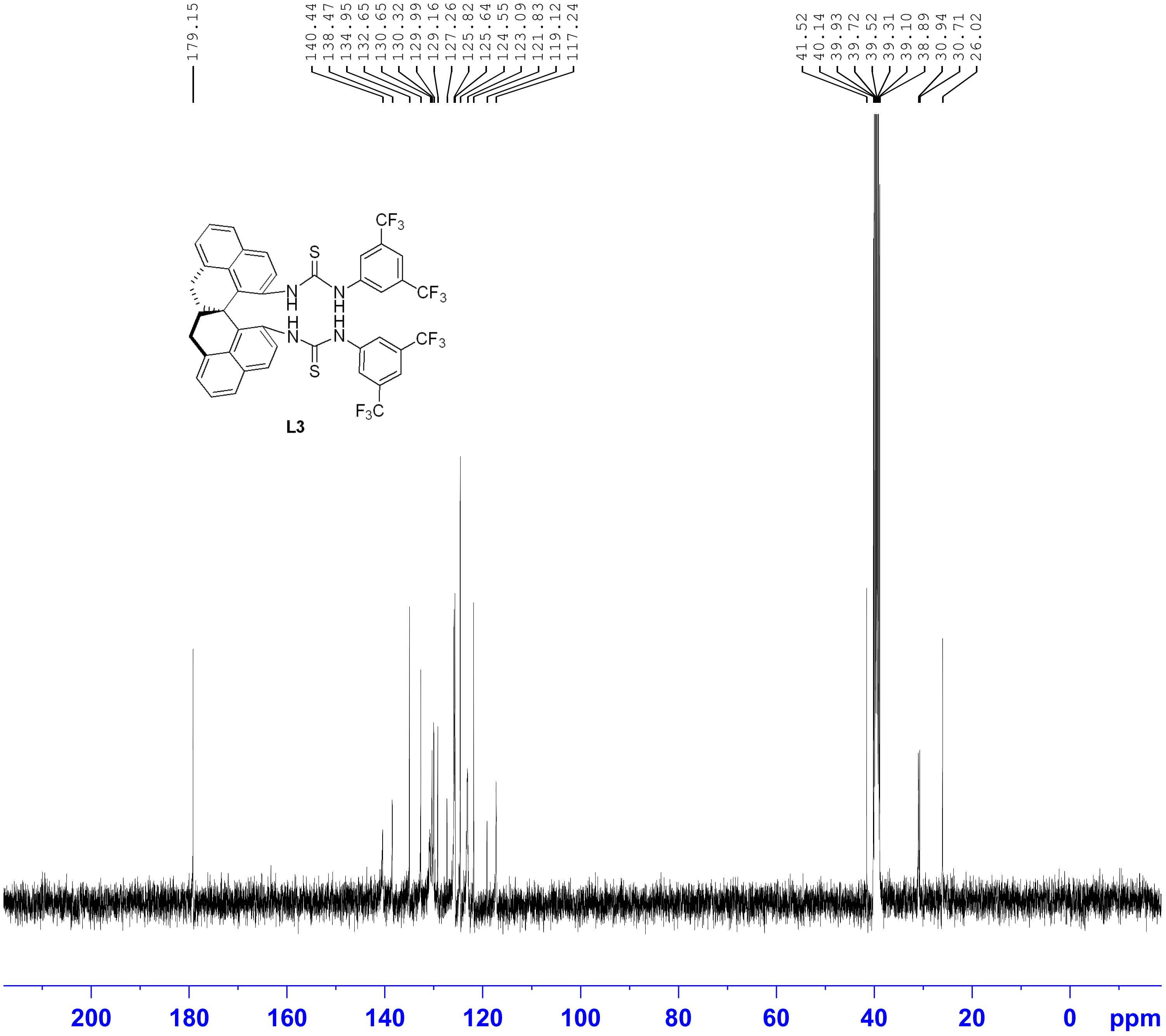


```
Current Data Parameters
NAME      zrh-8-123-re-c
EXPNO                 16
PROCNO                 1

F2 - Acquisition Parameters
Date_           20230110
Time               19.56 h
INSTRUM  AvanceNeo 400MHz
PROBHD   Z163739_0629 (
PULPROG           zgpg30
TD                 65536
SOLVENT             DMSO
NS                   230
DS                     4
SWH            23809.523 Hz
FIDRES          0.726609 Hz
AQ             1.3762560 sec
RG                    10
DW                21.000 usec
DE                  6.50 usec
TE                 295.7 K
D1            2.00000000 sec
D11           0.03000000 sec
TD0                    1
SFO1         100.6354036 MHz
NUC1                 13C
P0                  2.67 usec
P1                  8.00 usec
PLW1         85.25399780 W
SFO2         400.1816007 MHz
NUC2                  1H
CPDPRG[2         waltz65
PCPD2              90.00 usec
PLW2         21.26700020 W
PLW12         0.16802999 W
PLW13         0.08452000 W

F2 - Processing parameters
SI                 32768
SF           100.6253822 MHz
WDW                   EM
SSB                    0
LB                  1.00 Hz
GB                     0
PC                  1.40
```

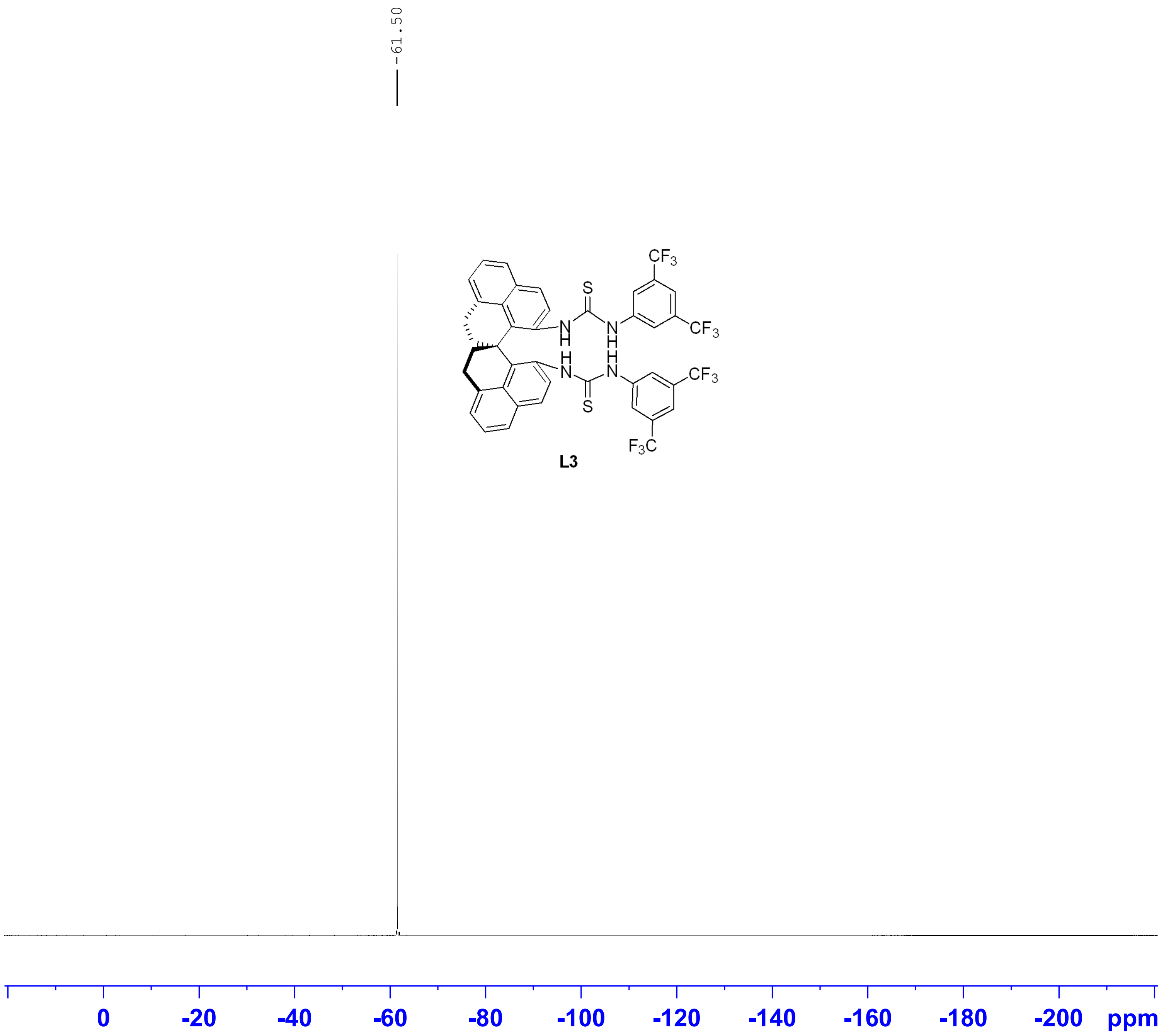

L3

```
Current Data Parameters
NAME      zrh-8-123-re-f
EXPNO                 14
PROCNO                 1

F2 - Acquisition Parameters
Date_           20230110
Time               19.40 h
INSTRUM   AvanceNeo 400MHz
PROBHD    Z163739_0629 (
PULPROG               zg
TD                131072
SOLVENT             DMSO
NS                    10
DS                     4
SWH            90909.094 Hz
FIDRES          1.387163 Hz
AQ             0.7208960 sec
RG                   101
DW                 5.500 usec
DE                  6.50 usec
TE                 295.4 K
D1            1.00000000 sec
TD0                    1
SFO1         376.5077587 MHz
NUC1                 19F
P1                 12.00 usec
PLW1         33.72800064 W

F2 - Processing parameters
SI                 65536
SF           376.5454132 MHz
WDW                   EM
SSB                    0
LB                  0.30 Hz
GB                     0
PC                  1.00
```

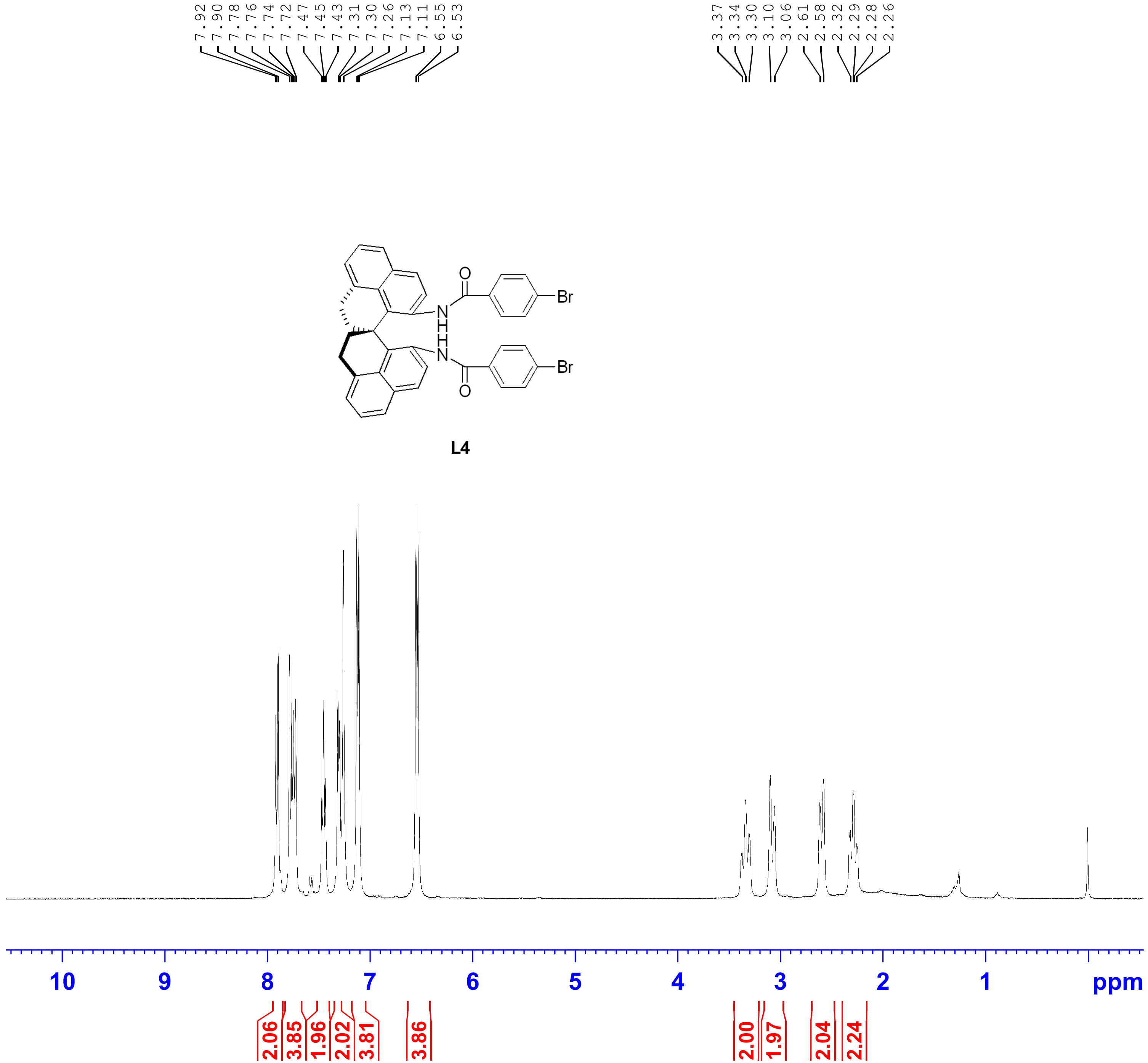

Current Data Parameters
NAME zrh-8-124-1-h
EXPNO 1
PROCNO 1

F2 - Acquisition Parameters
Date_ 20230109
Time 19.22
INSTRUM spect
PROBHD 5 mm PABBO BB/
PULPROG zg30
TD 65536
SOLVENT CDCl3
NS 12
DS 2
SWH 8012.820 Hz
FIDRES 0.122266 Hz
AQ 4.0894465 sec
RG 103.52
DW 62.400 usec
DE 6.50 usec
TE 294.9 K
D1 1.00000000 sec
TD0 1

======== CHANNEL f1 ========
SFO1 400.1324710 MHz
NUC1 1H
P1 14.50 usec
PLW1 11.99499989 W

F2 - Processing parameters
SI 65536
SF 400.1300095 MHz
WDW EM
SSB 0
LB 0.30 Hz
GB 0
PC 1.00

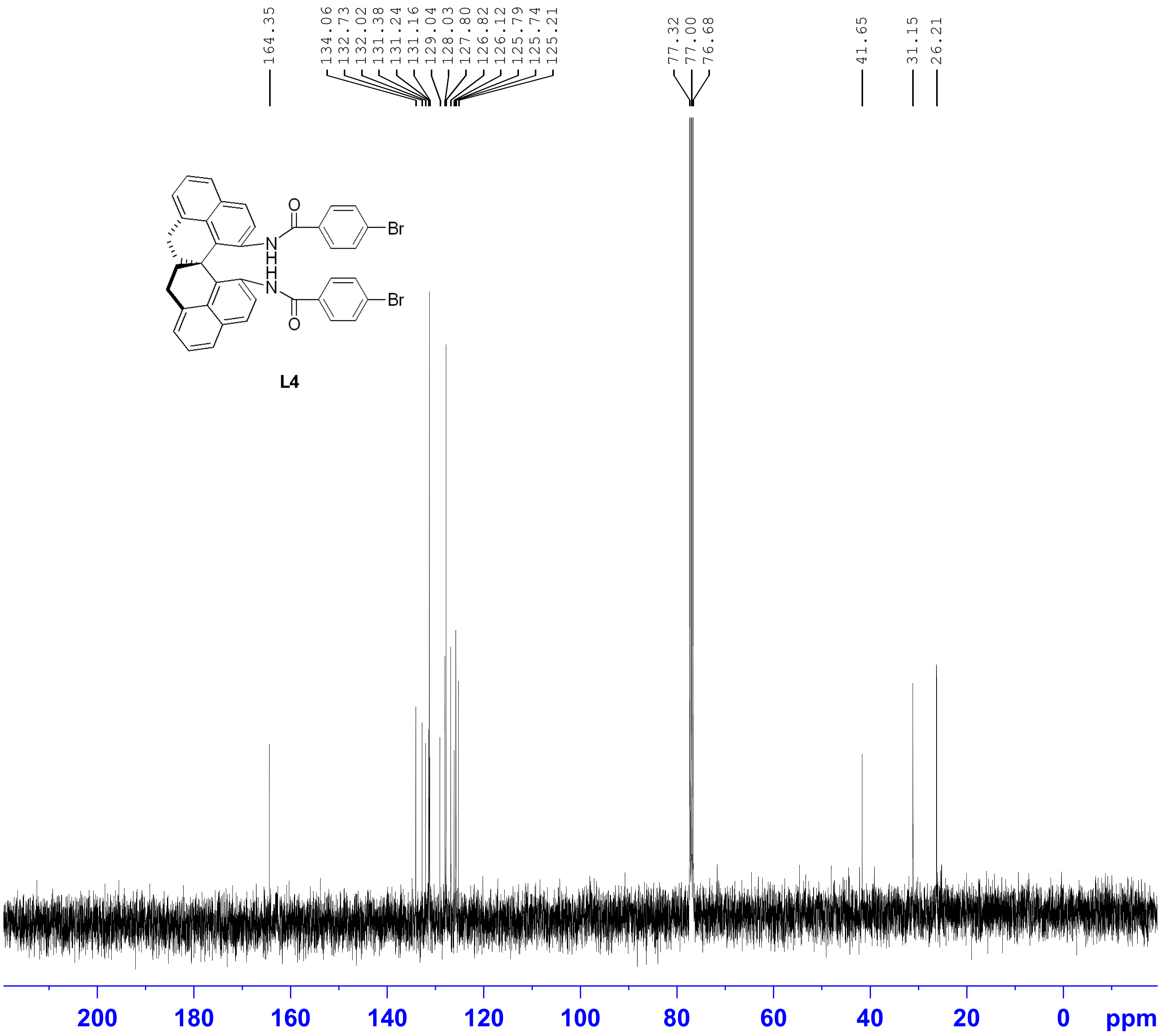

Current Data Parameters
NAME zrh-8-124-1-c
EXPNO 1
PROCNO 1

F2 - Acquisition Parameters
Date_ 20230109
Time 19.24
INSTRUM spect
PROBHD 5 mm PABBO BB/
PULPROG zgpg30
TD 65536
SOLVENT CDCl3
NS 80
DS 2
SWH 24038.461 Hz
FIDRES 0.366798 Hz
AQ 1.3631488 sec
RG 196.92
DW 20.800 usec
DE 6.50 usec
TE 295.2 K
D1 2.00000000 sec
D11 0.03000000 sec
TD0 1

======== CHANNEL f1 ========
SFO1 100.6228298 MHz
NUC1 13C
P1 9.70 usec
PLW1 46.98899841 W

======== CHANNEL f2 ========
SFO2 400.1316005 MHz
NUC2 1H
CPDPRG[2 waltz16
PCPD2 90.00 usec
PLW2 11.99499989 W
PLW12 0.34213999 W
PLW13 0.27713001 W

F2 - Processing parameters
SI 32768
SF 100.6127746 MHz
WDW EM
SSB 0
LB 1.00 Hz
GB 0
PC 1.40

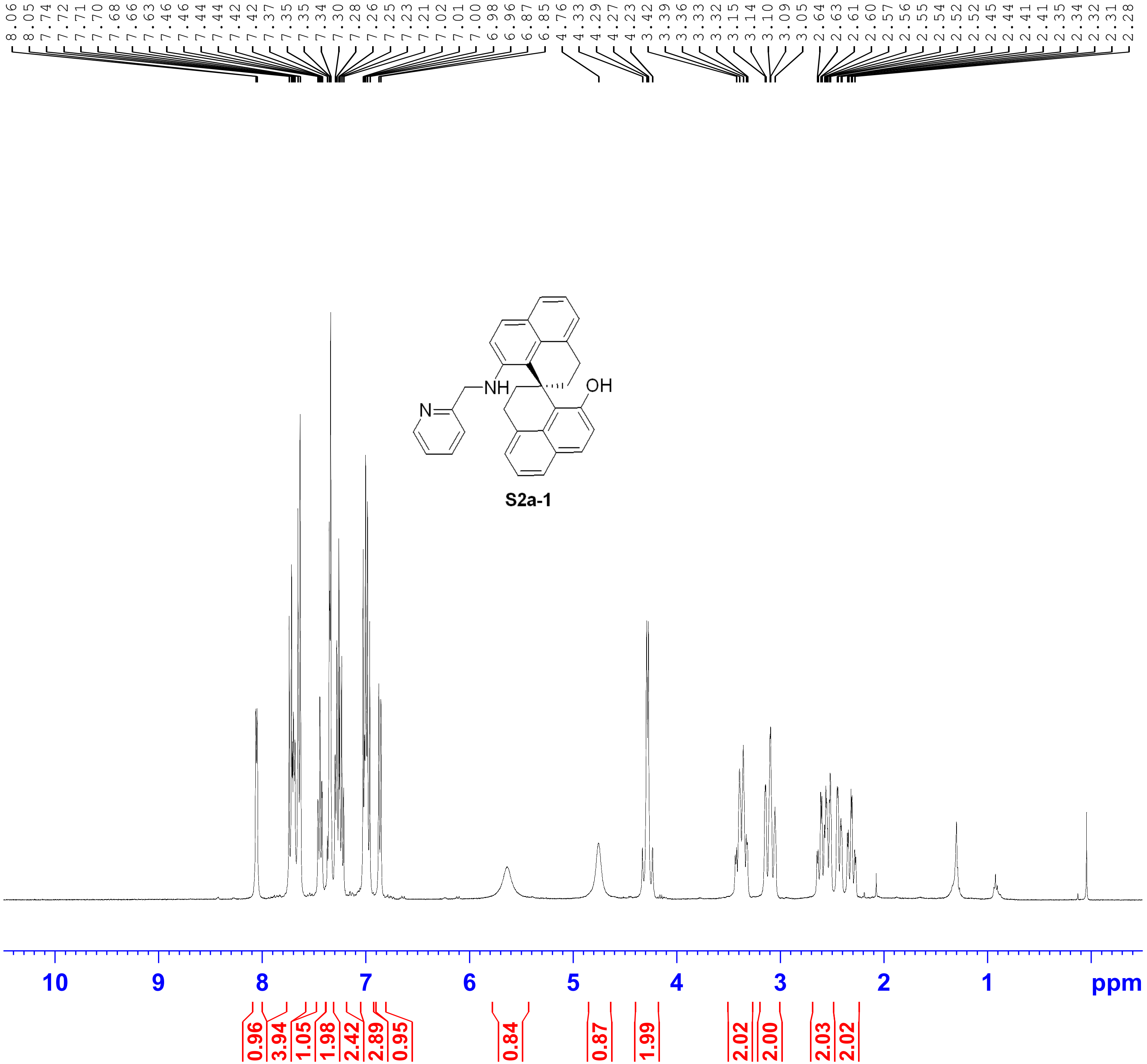

S2a-1

Current Data Parameters
NAME zrh-9-137-h
EXPNO 1
PROCNO 1

F2 - Acquisition Parameters
Date_ 20230531
Time 11.22
INSTRUM spect
PROBHD 5 mm PABBO BB/
PULPROG zg30
TD 65536
SOLVENT CDCl3
NS 3
DS 2
SWH 8012.820 Hz
FIDRES 0.122266 Hz
AQ 4.0894465 sec
RG 31.55
DW 62.400 usec
DE 6.50 usec
TE 296.3 K
D1 1.00000000 sec
TD0 1

======== CHANNEL f1 ========
SFO1 400.1324710 MHz
NUC1 1H
P1 14.50 usec
PLW1 11.99499989 W

F2 - Processing parameters
SI 65536
SF 400.1300099 MHz
WDW EM
SSB 0
LB 0.30 Hz
GB 0
PC 1.00

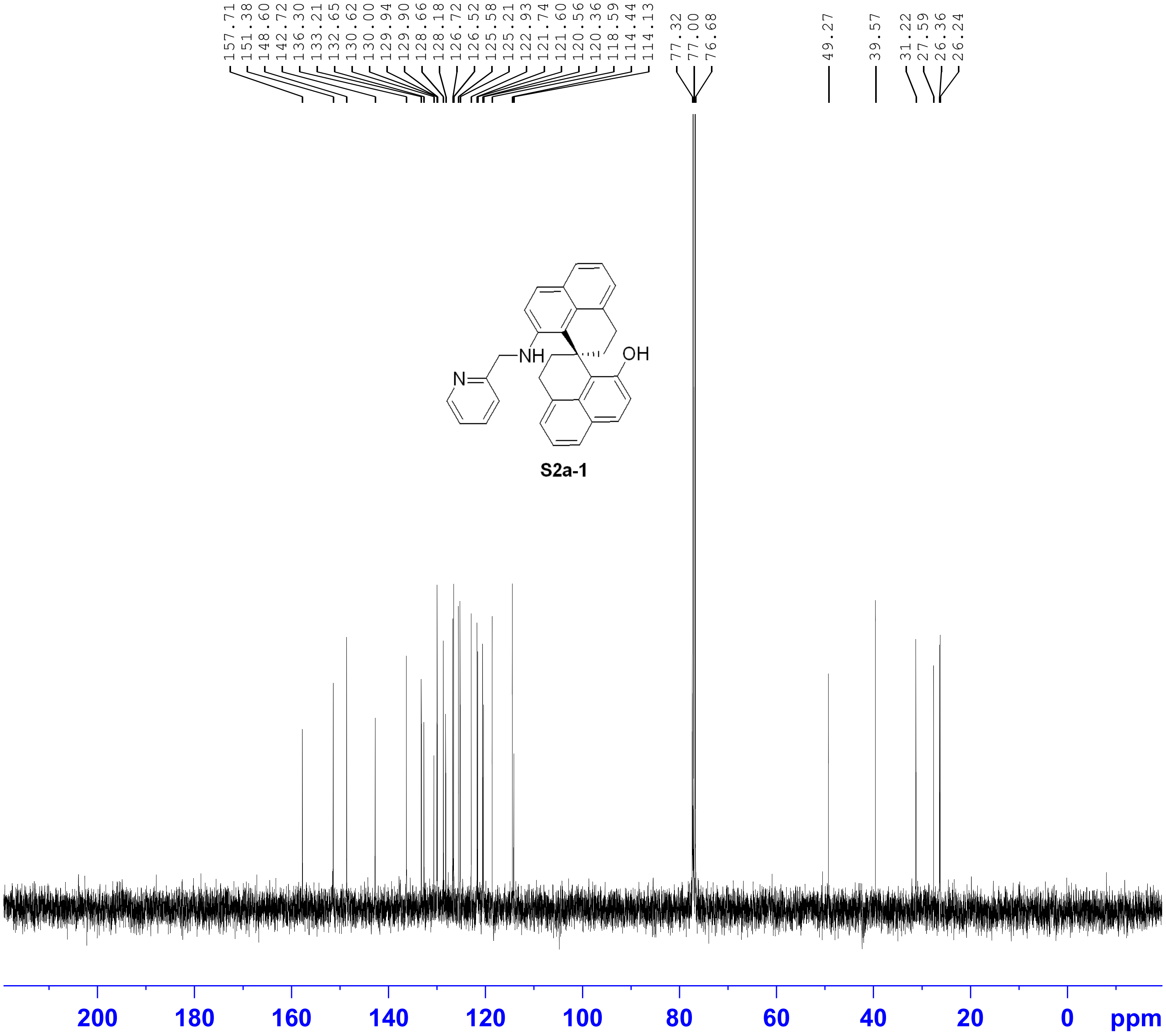

Current Data Parameters
NAME zrh-9-137-c
EXPNO 1
PROCNO 1

F2 - Acquisition Parameters
Date_ 20230531
Time 11.24
INSTRUM spect
PROBHD 5 mm PABBO BB/
PULPROG zgpg30
TD 65536
SOLVENT CDCl3
NS 53
DS 2
SWH 24038.461 Hz
FIDRES 0.366798 Hz
AQ 1.3631488 sec
RG 196.92
DW 20.800 usec
DE 6.50 usec
TE 296.9 K
D1 2.00000000 sec
D11 0.03000000 sec
TD0 1

======== CHANNEL f1 ========
SFO1 100.6228298 MHz
NUC1 13C
P1 9.70 usec
PLW1 46.98899841 W

======== CHANNEL f2 ========
SFO2 400.1316005 MHz
NUC2 1H
CPDPRG[2 waltz16
PCPD2 90.00 usec
PLW2 11.99499989 W
PLW12 0.34213999 W
PLW13 0.27713001 W

F2 - Processing parameters
SI 32768
SF 100.6127792 MHz
WDW EM
SSB 0
LB 1.00 Hz
GB 0
PC 1.40

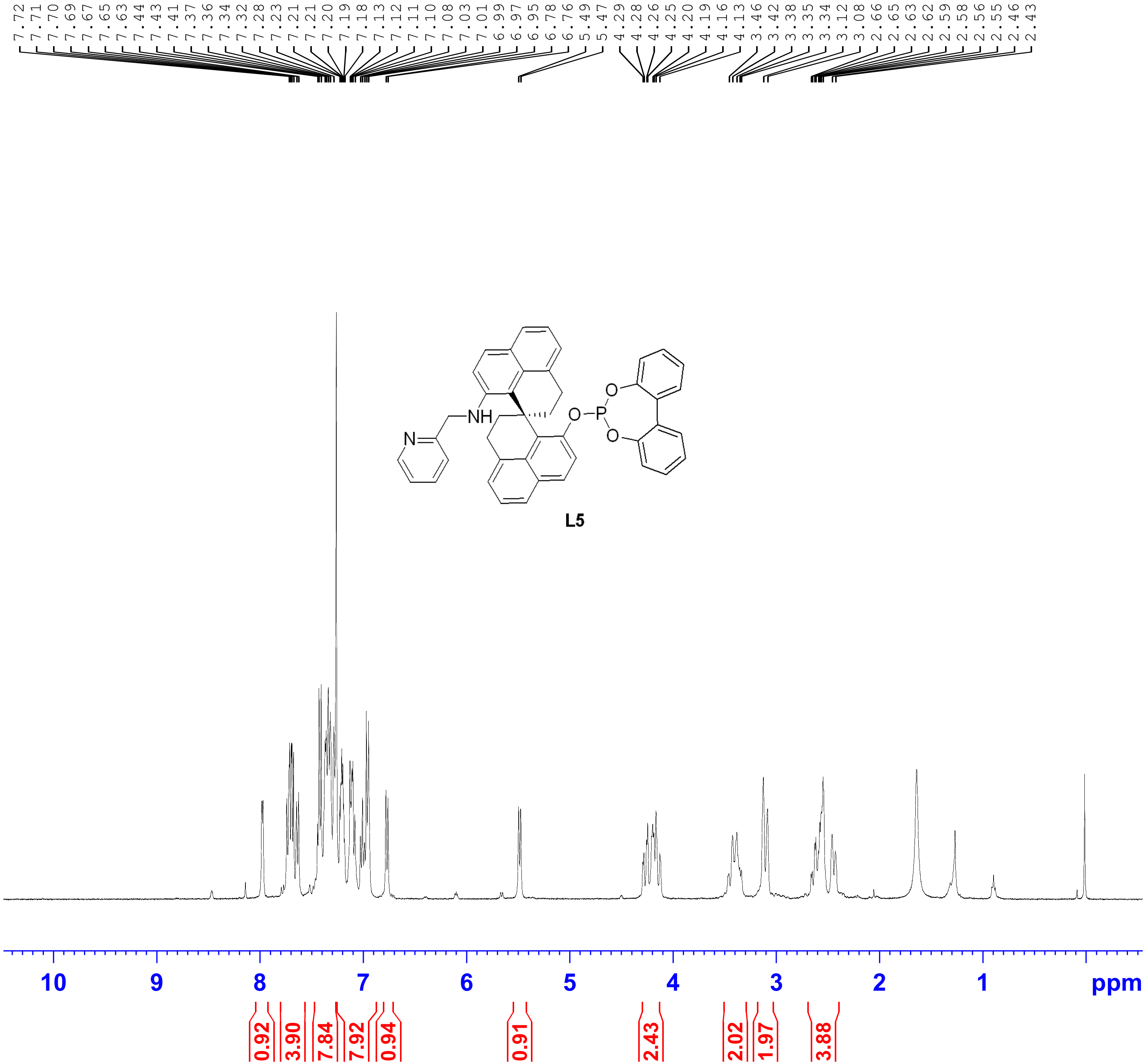

Current Data Parameters
NAME zrh-9-166-1-h
EXPNO 1
PROCNO 1

F2 - Acquisition Parameters
Date_ 20230621
Time 21.31
INSTRUM spect
PROBHD 5 mm PABBO BB/
PULPROG zg30
TD 65536
SOLVENT CDCl3
NS 5
DS 2
SWH 8012.820 Hz
FIDRES 0.122266 Hz
AQ 4.0894465 sec
RG 103.52
DW 62.400 usec
DE 6.50 usec
TE 296.3 K
D1 1.00000000 sec
TD0 1

======== CHANNEL f1 ========
SFO1 400.1324710 MHz
NUC1 1H
P1 14.50 usec
PLW1 11.99499989 W

F2 - Processing parameters
SI 65536
SF 400.1300104 MHz
WDW EM
SSB 0
LB 0.30 Hz
GB 0
PC 1.00

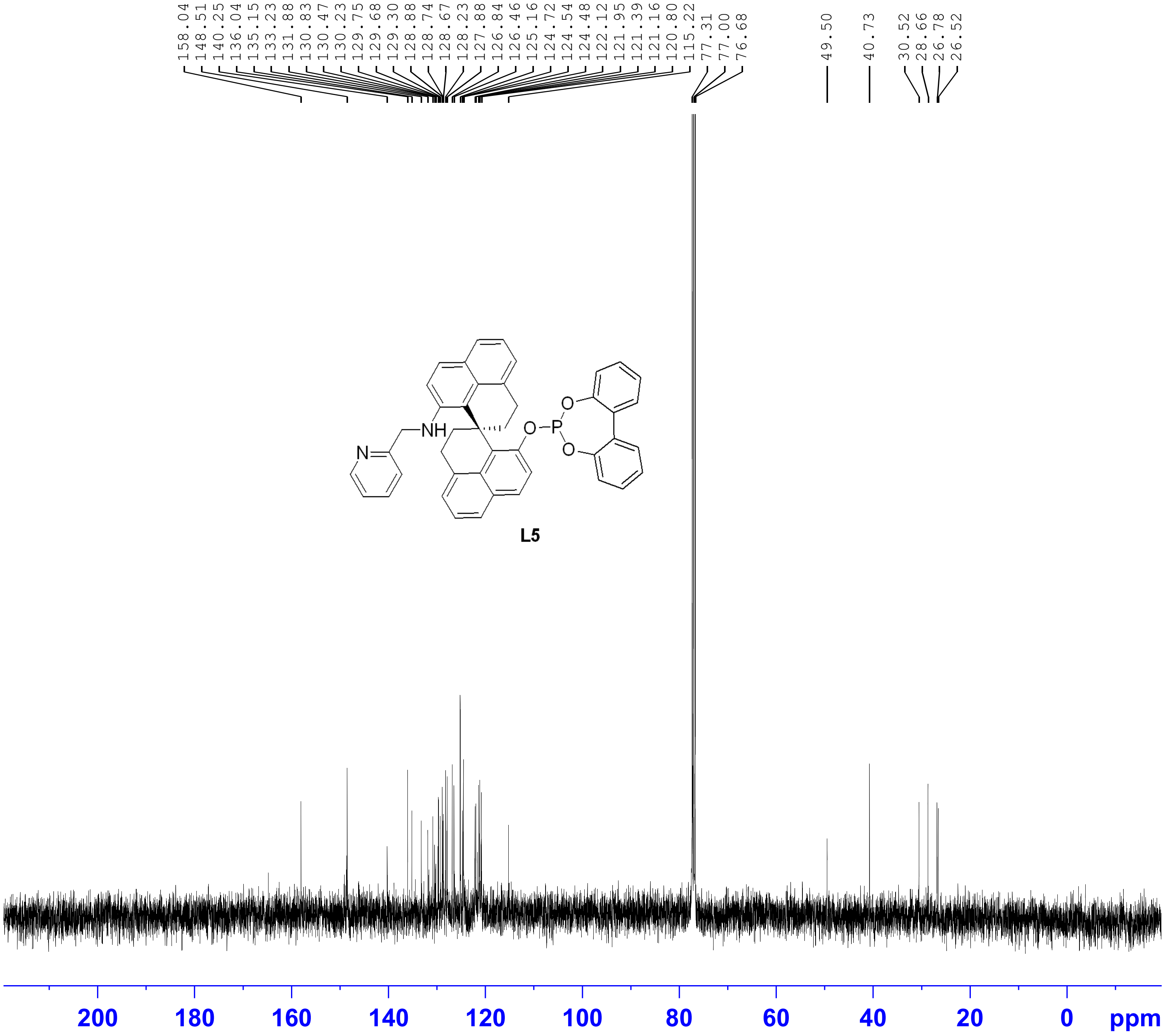

Current Data Parameters
NAME zrh-9-166-1-c
EXPNO 1
PROCNO 1

F2 - Acquisition Parameters
Date_ 20230621
Time 21.34
INSTRUM spect
PROBHD 5 mm PABBO BB/
PULPROG zgpg30
TD 65536
SOLVENT CDCl3
NS 134
DS 2
SWH 24038.461 Hz
FIDRES 0.366798 Hz
AQ 1.3631488 sec
RG 196.92
DW 20.800 usec
DE 6.50 usec
TE 296.7 K
D1 2.00000000 sec
D11 0.03000000 sec
TD0 1

======== CHANNEL f1 ========
SFO1 100.6228298 MHz
NUC1 13C
P1 9.70 usec
PLW1 46.98899841 W

======== CHANNEL f2 ========
SFO2 400.1316005 MHz
NUC2 1H
CPDPRG[2 waltz16
PCPD2 90.00 usec
PLW2 11.99499989 W
PLW12 0.34213999 W
PLW13 0.27713001 W

F2 - Processing parameters
SI 32768
SF 100.6127736 MHz
WDW EM
SSB 0
LB 1.00 Hz
GB 0
PC 1.40

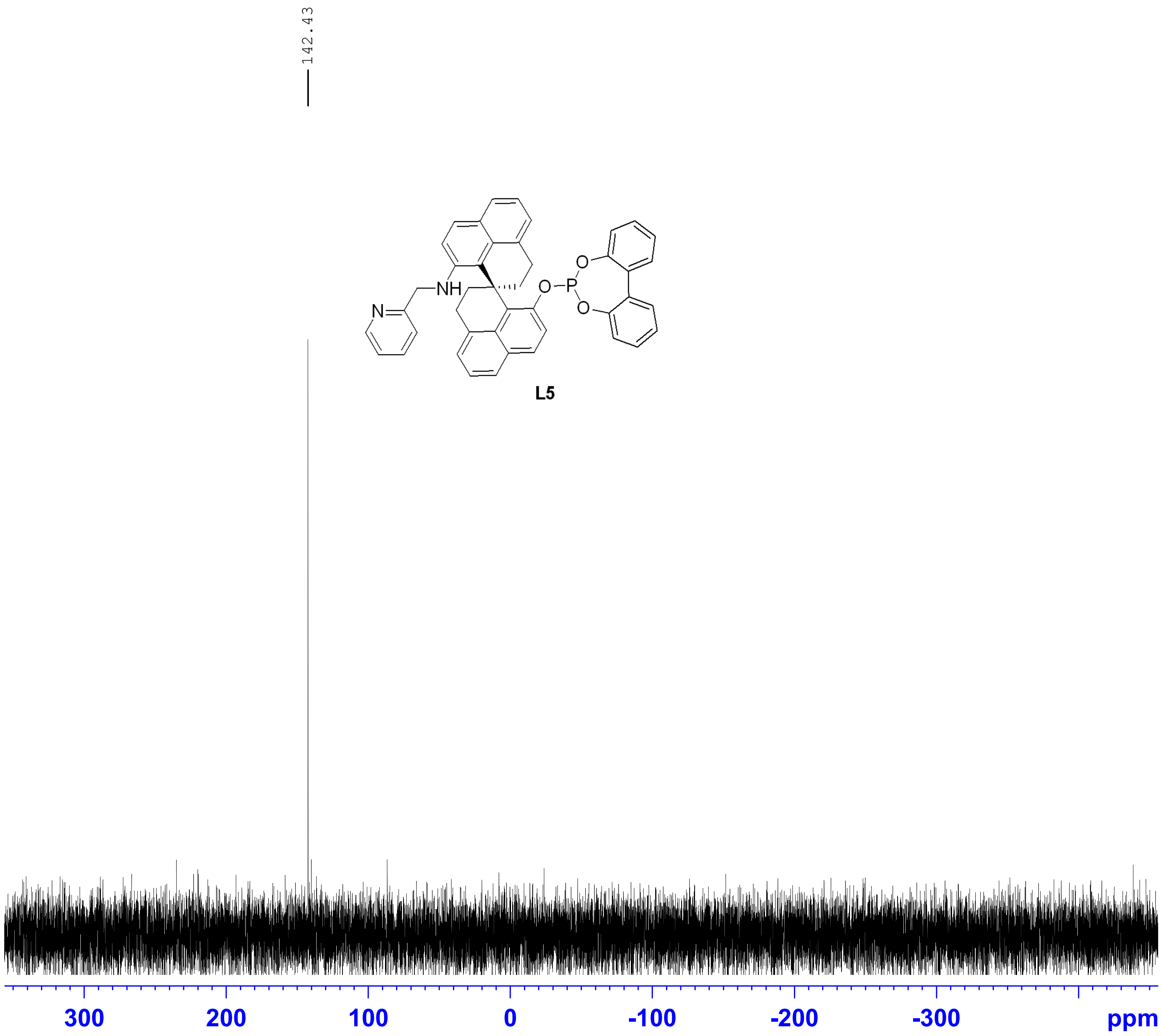

Current Data Parameters
NAME zmh-zrh-p
EXPNO 1
PROCNO 1

F2 - Acquisition Parameters
Date_ 20250913
Time 8.07 h
INSTRUM AvanceNeo 400MHz
PROBHD Z163739_0629 (
PULPROG zg30
TD 65536
SOLVENT CDCl3
NS 15
DS 4
SWH 131578.953 Hz
FIDRES 4.015471 Hz
AQ 0.2490368 sec
RG 101
DW 3.800 usec
DE 6.50 usec
TE 297.9 K
D1 2.00000000 sec
TD0 1
SFO1 161.9877335 MHz
NUC1 31P
P0 2.67 usec
P1 8.00 usec
PLW1 56.00000000 W

F2 - Processing parameters
SI 32768
SF 161.9958333 MHz
WDW EM
SSB 0
LB 1.00 Hz
GB 0
PC 1.40

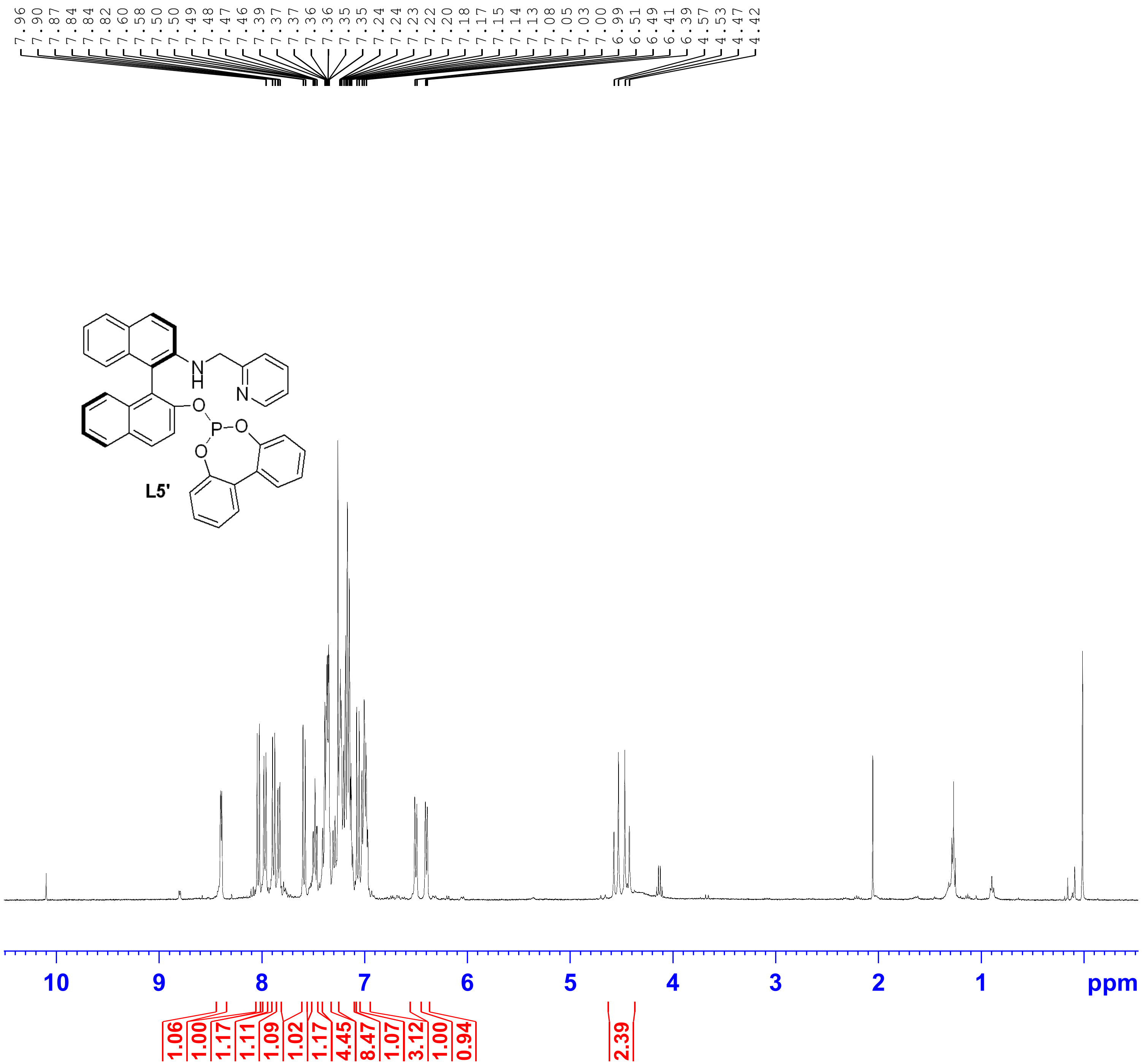


```
Current Data Parameters
NAME          zmh-0909-2-re
EXPNO                     4
PROCNO                    1

F2 - Acquisition Parameters
Date_              20250909
Time                   2.37
INSTRUM               spect
PROBHD     5 mm PABBO BB/
PULPROG                zg30
TD                    65536
SOLVENT               CDCl3
NS                        8
DS                        2
SWH                8012.820 Hz
FIDRES             0.122266 Hz
AQ                4.0894465 sec
RG                    88.84
DW                   62.400 usec
DE                     6.50 usec
TE                    296.4 K
D1               1.00000000 sec
TD0                       1

======== CHANNEL f1 ========
SFO1            400.1324710 MHz
NUC1                     1H
P1                    14.50 usec
PLW1            11.99499989 W

F2 - Processing parameters
SI                    65536
SF              400.1300096 MHz
WDW                      EM
SSB                       0
LB                     0.30 Hz
GB                        0
PC                     1.00
```

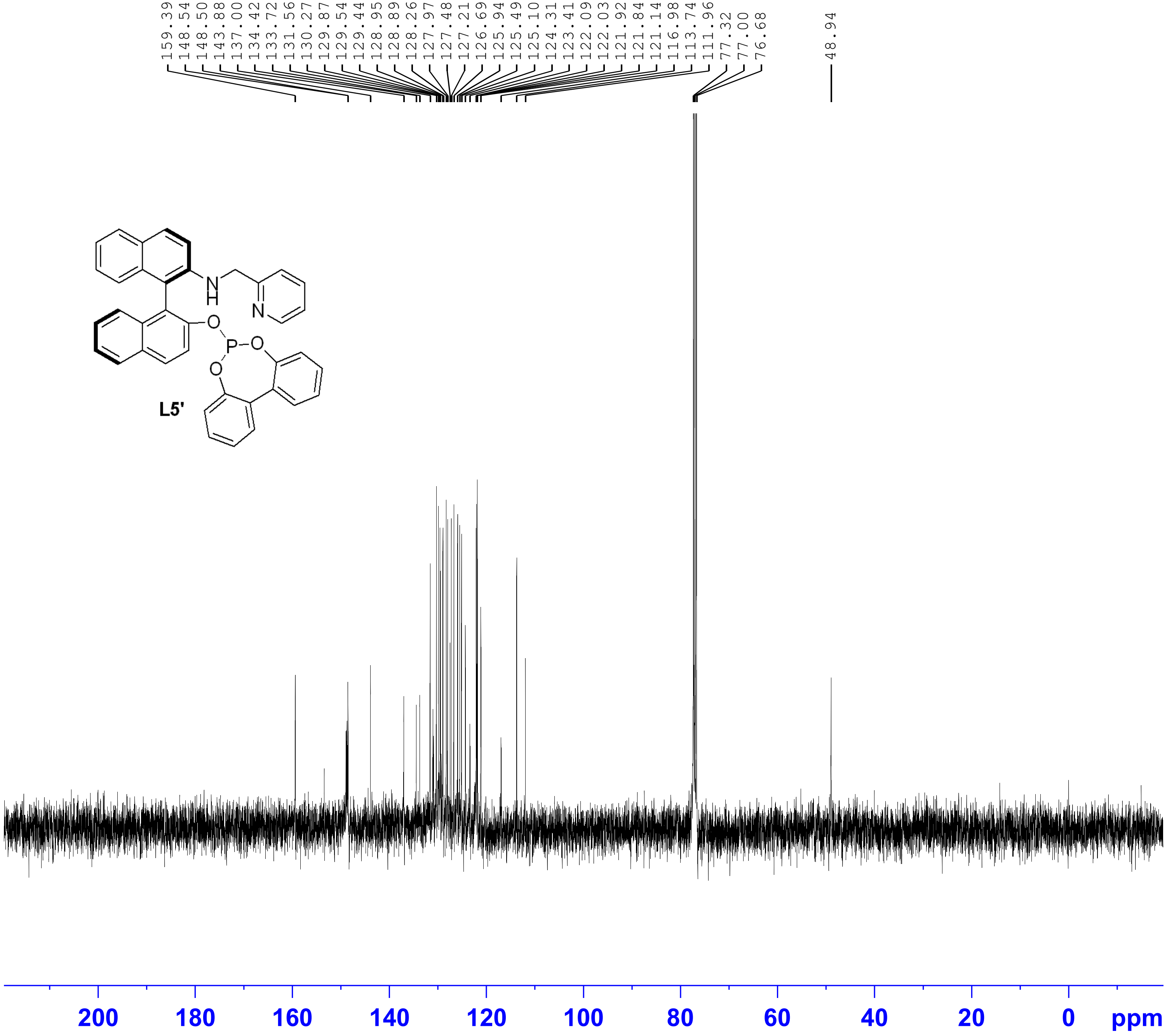

Current Data Parameters
NAME zmh-0909-2-re
EXPNO 6
PROCNO 1

F2 - Acquisition Parameters
Date_ 20250909
Time 2.42
INSTRUM spect
PROBHD 5 mm PABBO BB/
PULPROG zgpg30
TD 65536
SOLVENT CDCl3
NS 341
DS 2
SWH 24038.461 Hz
FIDRES 0.366798 Hz
AQ 1.3631488 sec
RG 196.92
DW 20.800 usec
DE 6.50 usec
TE 297.0 K
D1 2.00000000 sec
D11 0.03000000 sec
TD0 1

======== CHANNEL f1 ========
SFO1 100.6228298 MHz
NUC1 13C
P1 9.70 usec
PLW1 46.98899841 W

======== CHANNEL f2 ========
SFO2 400.1316005 MHz
NUC2 1H
CPDPRG[2 waltz16
PCPD2 90.00 usec
PLW2 11.99499989 W
PLW12 0.34213999 W
PLW13 0.27713001 W

F2 - Processing parameters
SI 32768
SF 100.6127734 MHz
WDW EM
SSB 0
LB 1.00 Hz
GB 0
PC 1.40

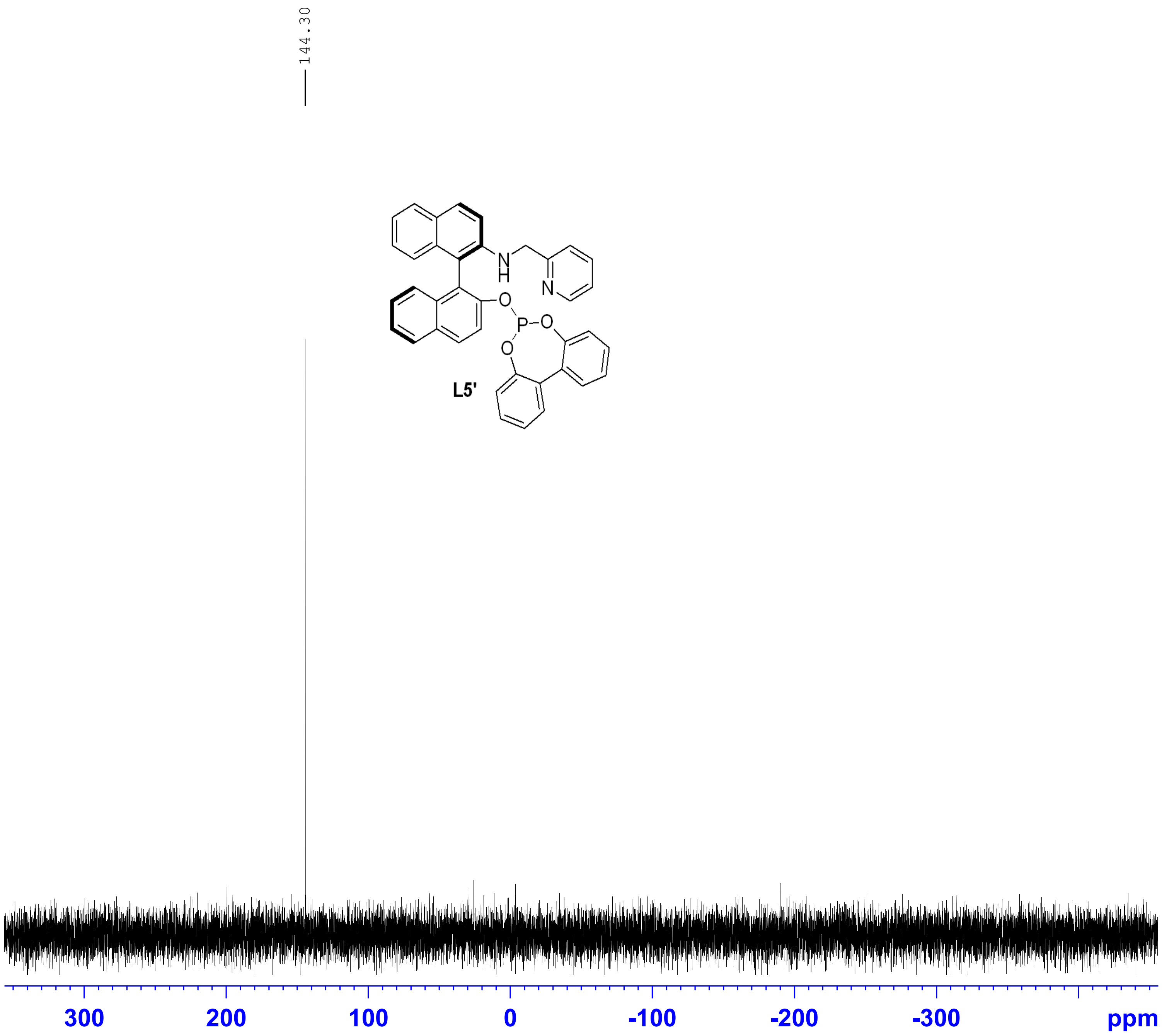

Current Data Parameters
NAME zmh-0913
EXPNO 3
PROCNO 1

F2 - Acquisition Parameters
Date_ 20250913
Time 4.25 h
INSTRUM AvanceNeo 400MHz
PROBHD Z163739_0629 (
PULPROG zg30
TD 65536
SOLVENT CDCl3
NS 18
DS 4
SWH 131578.953 Hz
FIDRES 4.015471 Hz
AQ 0.2490368 sec
RG 101
DW 3.800 usec
DE 6.50 usec
TE 297.1 K
D1 2.00000000 sec
TD0 1
SFO1 161.9877335 MHz
NUC1 31P
P0 2.67 usec
P1 8.00 usec
PLW1 56.00000000 W

F2 - Processing parameters
SI 32768
SF 161.9958333 MHz
WDW EM
SSB 0
LB 1.00 Hz
GB 0
PC 1.40

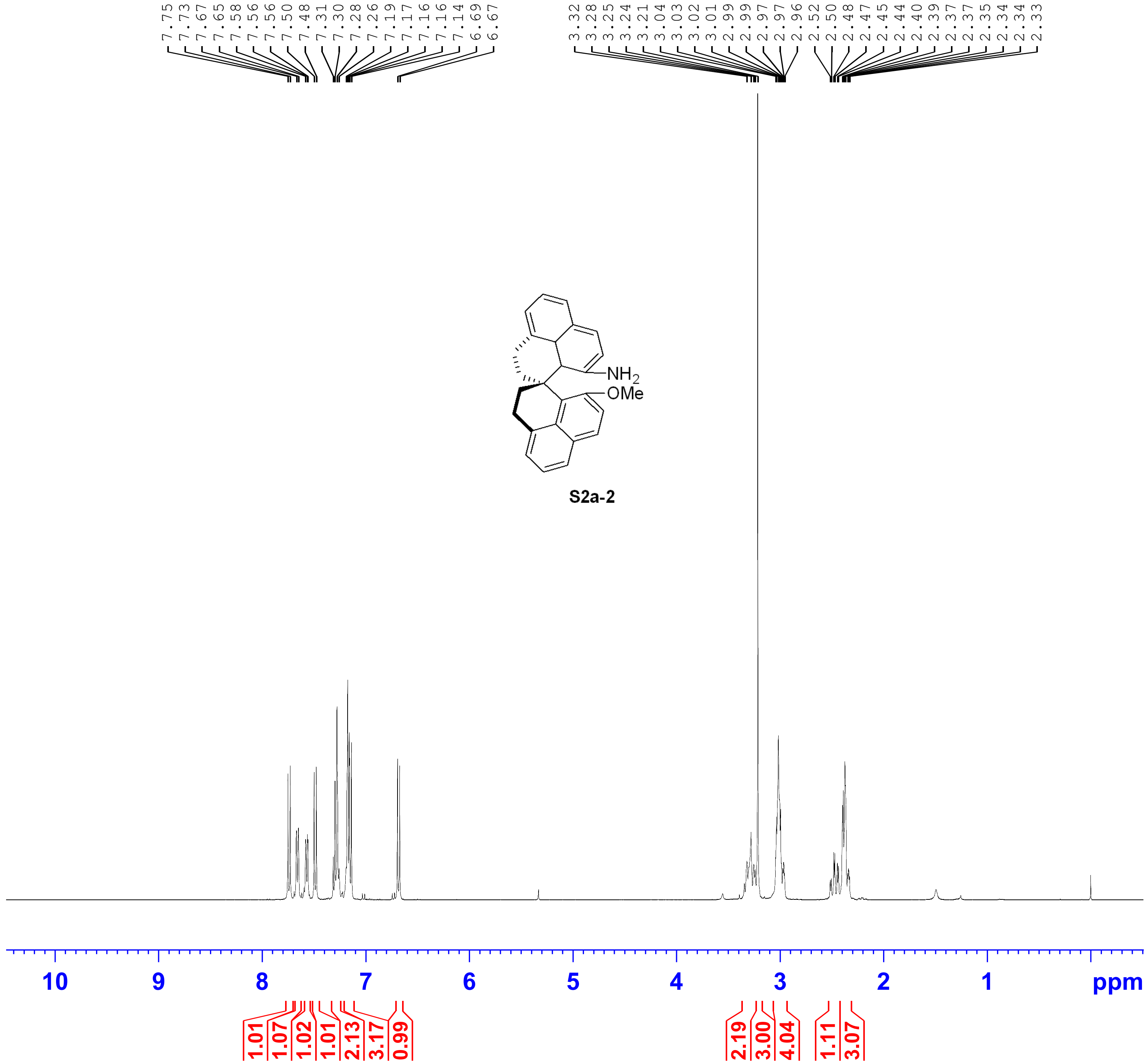

S2a-2

```
Current Data Parameters
NAME             zrh-7-62-h
EXPNO                     1
PROCNO                    1

F2 - Acquisition Parameters
Date_              20220729
Time                  14.19
INSTRUM               spect
PROBHD     5 mm DUL 13C-1
PULPROG                zg30
TD                    65536
SOLVENT               CDCl3
NS                        2
DS                        0
SWH                8223.685 Hz
FIDRES             0.125483 Hz
AQ                3.9845889 sec
RG                      128
DW                   60.800 usec
DE                     6.00 usec
TE                    293.0 K
D1               1.00000000 sec
TD0                       1

======== CHANNEL f1 ========
NUC1                     1H
P1                    15.80 usec
PL1                   -1.00 dB
PL1W            12.17476940 W
SFO1            400.1324710 MHz

F2 - Processing parameters
SI                    32768
SF              400.1300408 MHz
WDW                      EM
SSB                       0
LB                     0.30 Hz
GB                        0
PC                     1.00
```

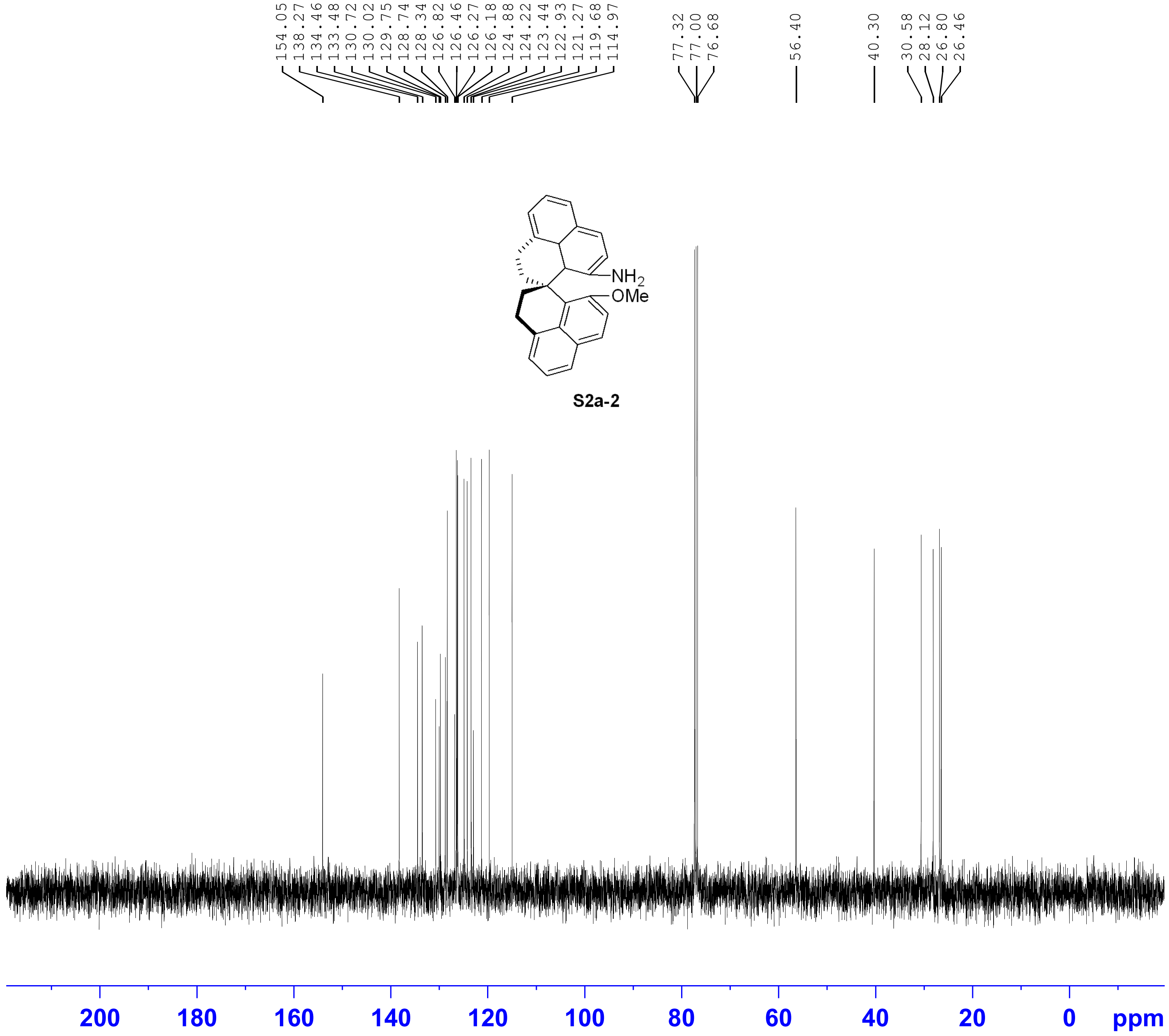

S2a-2

Current Data Parameters
NAME zrh-7-62-c
EXPNO 1
PROCNO 1

F2 - Acquisition Parameters
Date_ 20220729
Time 14.21
INSTRUM spect
PROBHD 5 mm DUL 13C-1
PULPROG zgpg30
TD 65536
SOLVENT CDCl3
NS 37
DS 0
SWH 24038.461 Hz
FIDRES 0.366798 Hz
AQ 1.3631488 sec
RG 2050
DW 20.800 usec
DE 6.00 usec
TE 293.2 K
D1 2.00000000 sec
D11 0.03000000 sec
TD0 1

======== CHANNEL f1 ========
NUC1 13C
P1 40.00 usec
PL1 -3.00 dB
PL1W 60.64365387 W
SFO1 100.6228298 MHz

======== CHANNEL f2 ========
CPDPRG[2 waltz16
NUC2 1H
PCPD2 80.00 usec
PL2 -1.00 dB
PL12 14.39 dB
PL13 18.00 dB
PL2W 12.17476940 W
PL12W 0.35193357 W
PL13W 0.15327126 W
SFO2 400.1316005 MHz

F2 - Processing parameters
SI 32768
SF 100.6127833 MHz
WDW EM
SSB 0
LB 1.00 Hz
GB 0
PC 1.40

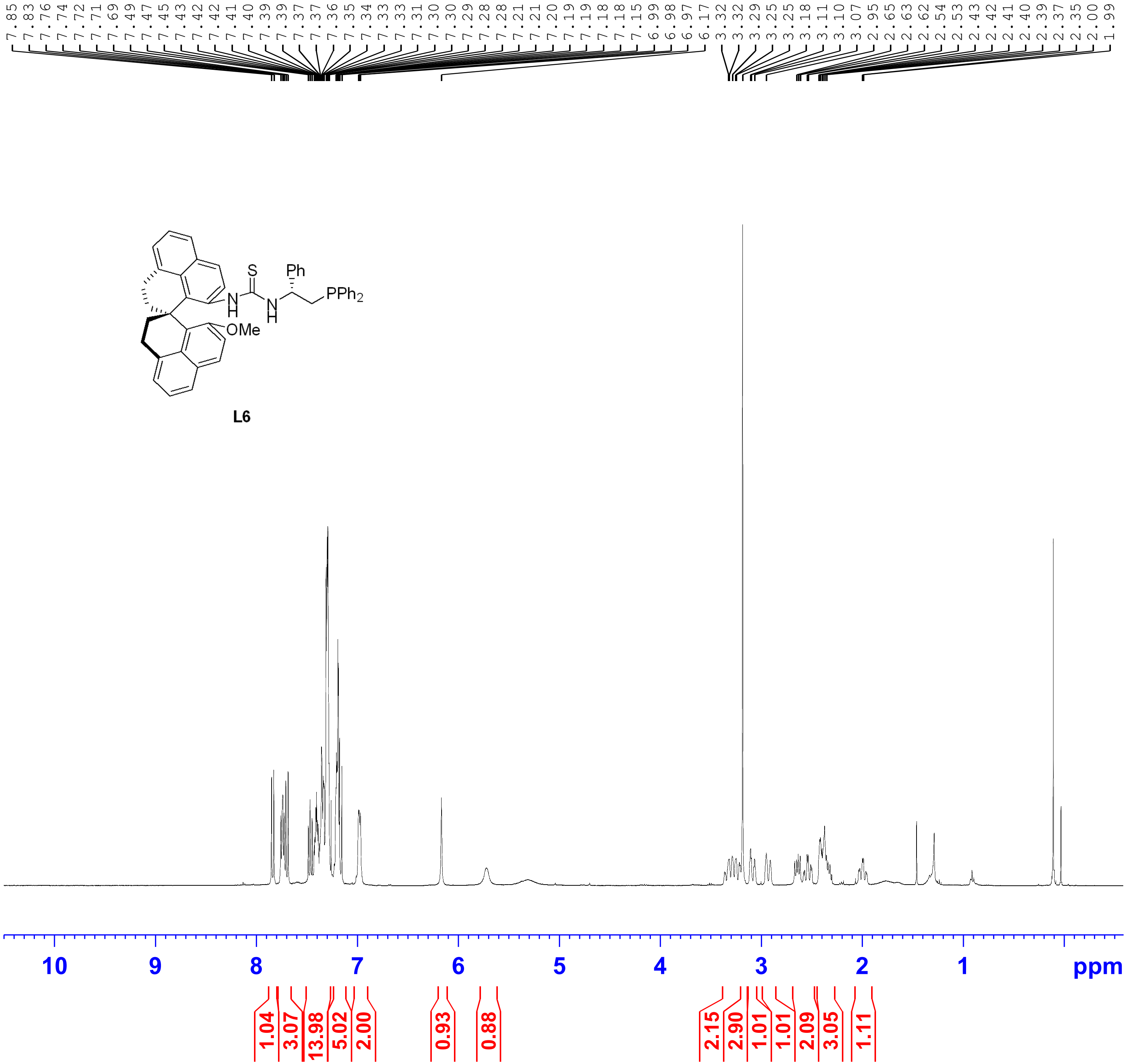


```
Current Data Parameters
NAME      zrh-7-79-SR-re-h
EXPNO                    1
PROCNO                   1

F2 - Acquisition Parameters
Date_             20230603
Time               11.31 h
INSTRUM   AvanceNeo 400MHz
PROBHD    Z163739_0629 (
PULPROG               zg30
TD                   65536
SOLVENT              CDCl3
NS                       5
DS                       2
SWH               8196.722 Hz
FIDRES            0.250144 Hz
AQ               3.9976959 sec
RG                     101
DW                  61.000 usec
DE                   13.89 usec
TE                   296.7 K
D1              1.00000000 sec
TD0                      1
SFO1           400.1824711 MHz
NUC1                    1H
P0                    2.67 usec
P1                    8.00 usec
PLW1           21.26700020 W

F2 - Processing parameters
SI                   65536
SF             400.1800095 MHz
WDW                     EM
SSB                      0
LB                    0.30 Hz
GB                       0
PC                    1.00
```

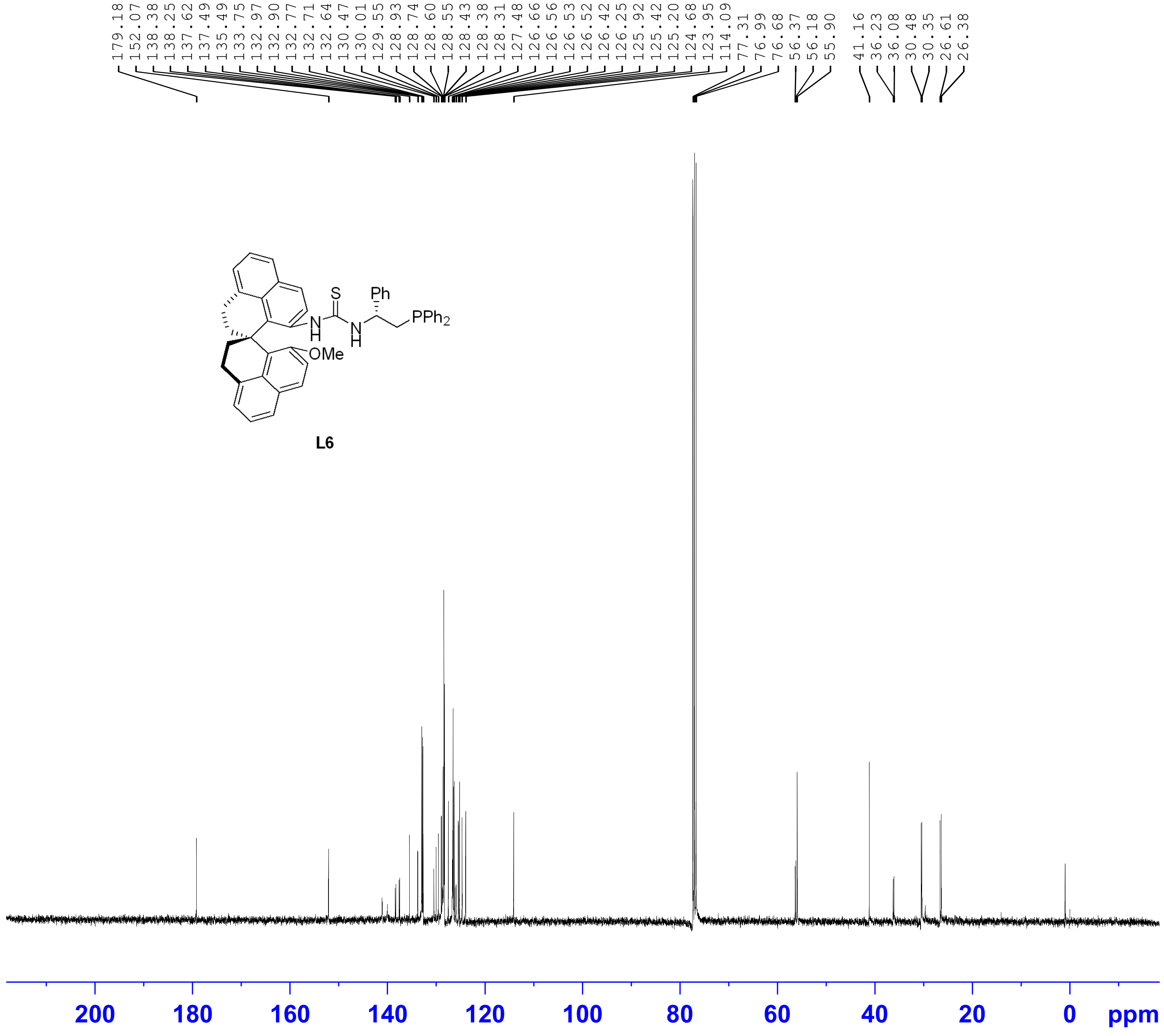

Current Data Parameters
NAME zrh-7-79-SR-c
EXPNO 5
PROCNO 1

F2 - Acquisition Parameters
Date_ 20230603
Time 11.58 h
INSTRUM AvanceNeo 400MHz
PROBHD Z163739_0629 (
PULPROG zgpg30
TD 65536
SOLVENT CDCl3
NS 400
DS 4
SWH 23809.523 Hz
FIDRES 0.726609 Hz
AQ 1.3762560 sec
RG 10
DW 21.000 usec
DE 6.50 usec
TE 297.1 K
D1 2.00000000 sec
D11 0.03000000 sec
TD0 1
SFO1 100.6354036 MHz
NUC1 13C
P0 2.67 usec
P1 8.00 usec
PLW1 85.25399780 W
SFO2 400.1816007 MHz
NUC2 1H
CPDPRG[2 waltz65
PCPD2 90.00 usec
PLW2 21.26700020 W
PLW12 0.16802999 W
PLW13 0.08452000 W

F2 - Processing parameters
SI 32768
SF 100.6253514 MHz
WDW EM
SSB 0
LB 1.00 Hz
GB 0
PC 1.40

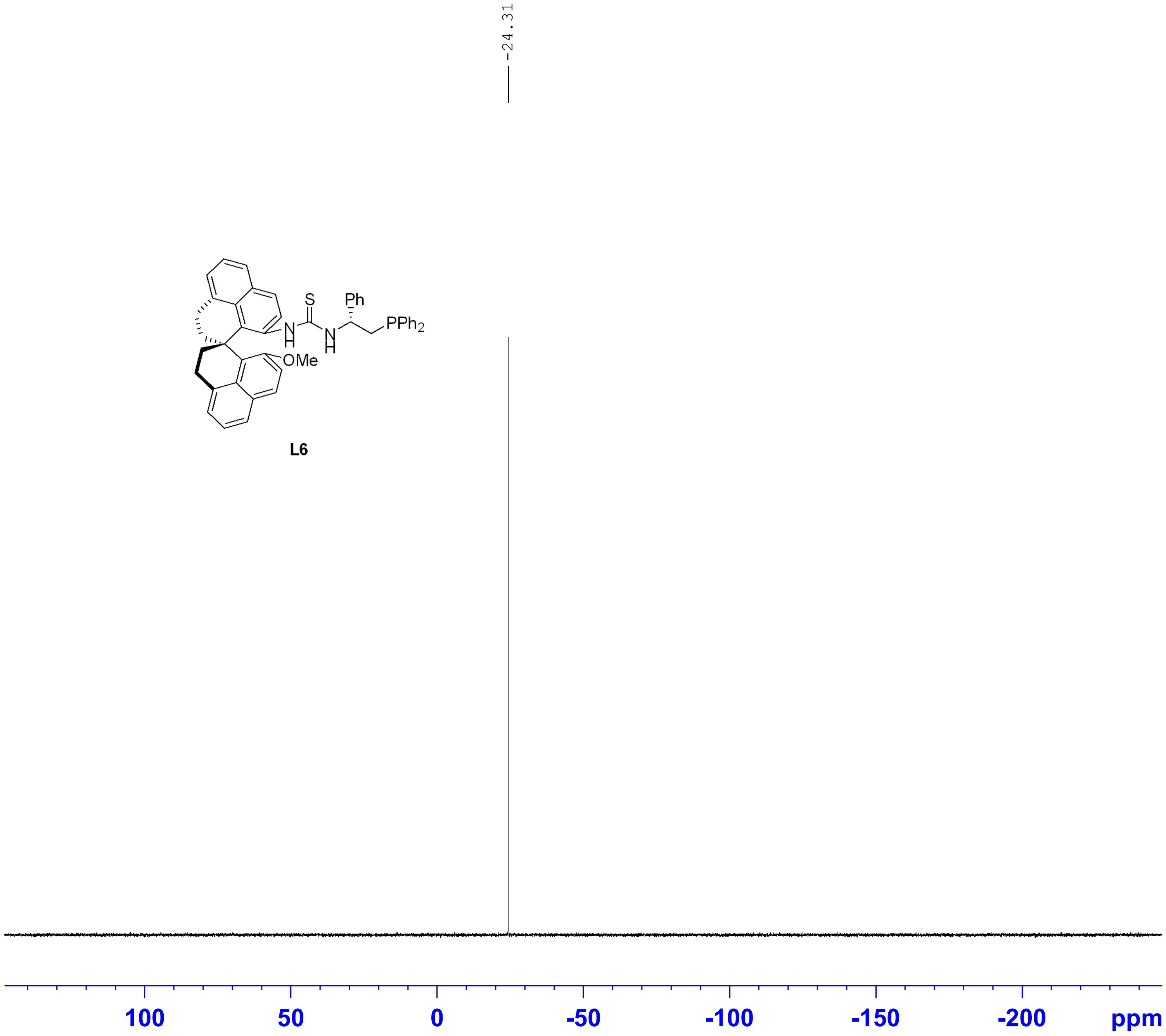

Current Data Parameters
NAME zrh-7-79-SR-p
EXPNO 2
PROCNO 1

F2 - Acquisition Parameters
Date_ 20220810
Time 16.15
INSTRUM spect
PROBHD 5 mm PABBO BB/
PULPROG zgpg30
TD 65536
SOLVENT CDCl3
NS 4
DS 0
SWH 64102.563 Hz
FIDRES 0.978127 Hz
AQ 0.5111808 sec
RG 196.92
DW 7.800 usec
DE 6.50 usec
TE 296.6 K
D1 2.00000000 sec
D11 0.03000000 sec
TD0 1

======== CHANNEL f1 ========
SFO1 161.9674942 MHz
NUC1 31P
P1 14.70 usec
PLW1 11.99499989 W

======== CHANNEL f2 ========
SFO2 400.1316005 MHz
NUC2 1H
CPDPRG[2 waltz16
PCPD2 90.00 usec
PLW2 11.99499989 W
PLW12 0.34213999 W
PLW13 0.27713001 W

F2 - Processing parameters
SI 32768
SF 161.9755930 MHz
WDW EM
SSB 0
LB 1.00 Hz
GB 0
PC 1.40

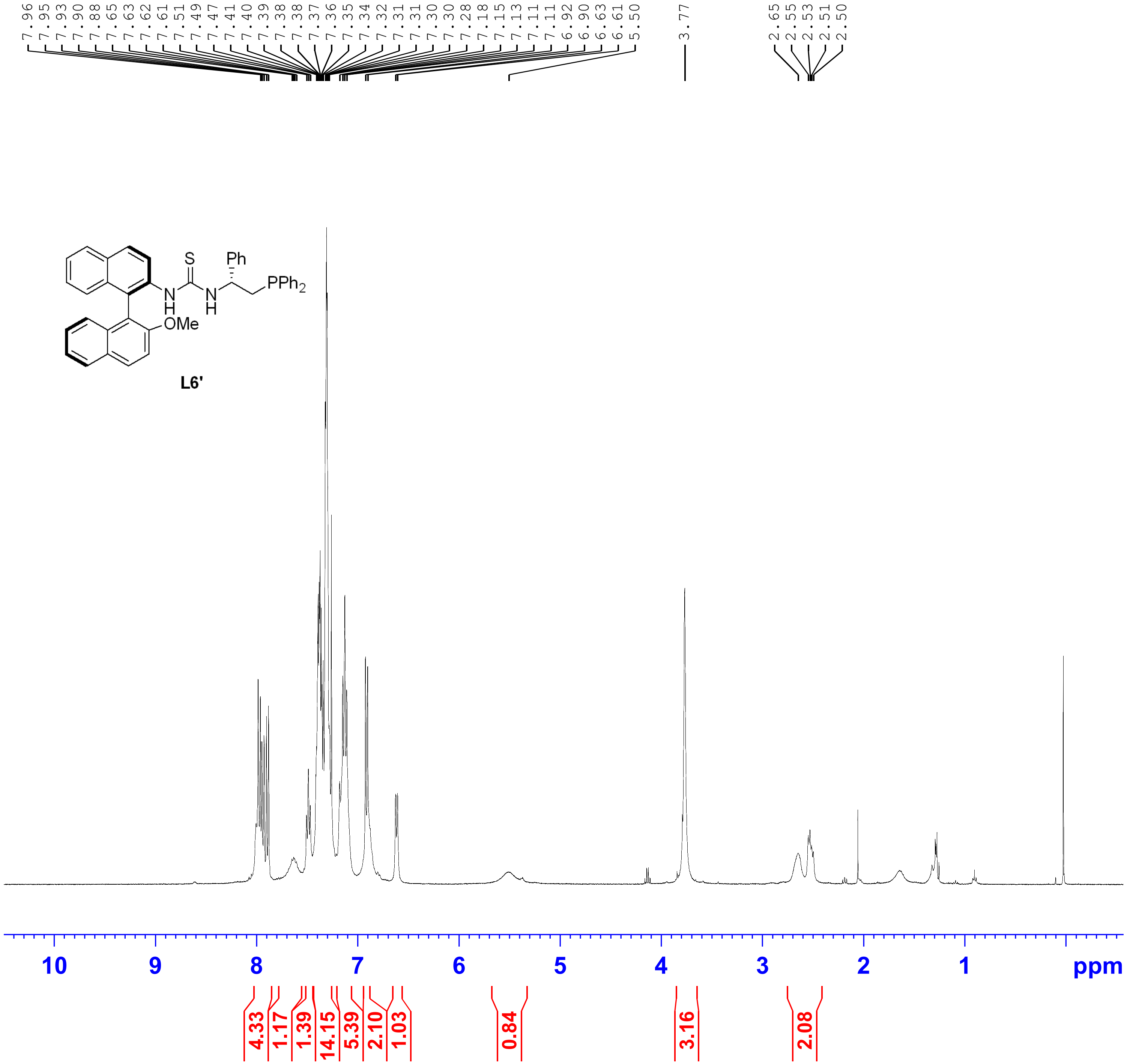


```
Current Data Parameters
NAME             zmh-0909-1
EXPNO                     1
PROCNO                    1

F2 - Acquisition Parameters
Date_              20250908
Time                  22.07
INSTRUM               spect
PROBHD     5 mm PABBO BB/
PULPROG                zg30
TD                    65536
SOLVENT               CDCl3
NS                        8
DS                        2
SWH               8012.820 Hz
FIDRES            0.122266 Hz
AQ               4.0894465 sec
RG                    62.93
DW                   62.400 usec
DE                     6.50 usec
TE                    296.8 K
D1               1.00000000 sec
TD0                       1

======== CHANNEL f1 ========
SFO1           400.1324710 MHz
NUC1                     1H
P1                    14.50 usec
PLW1            11.99499989 W

F2 - Processing parameters
SI                    65536
SF             400.1300098 MHz
WDW                      EM
SSB                       0
LB                     0.30 Hz
GB                        0
PC                     1.00
```

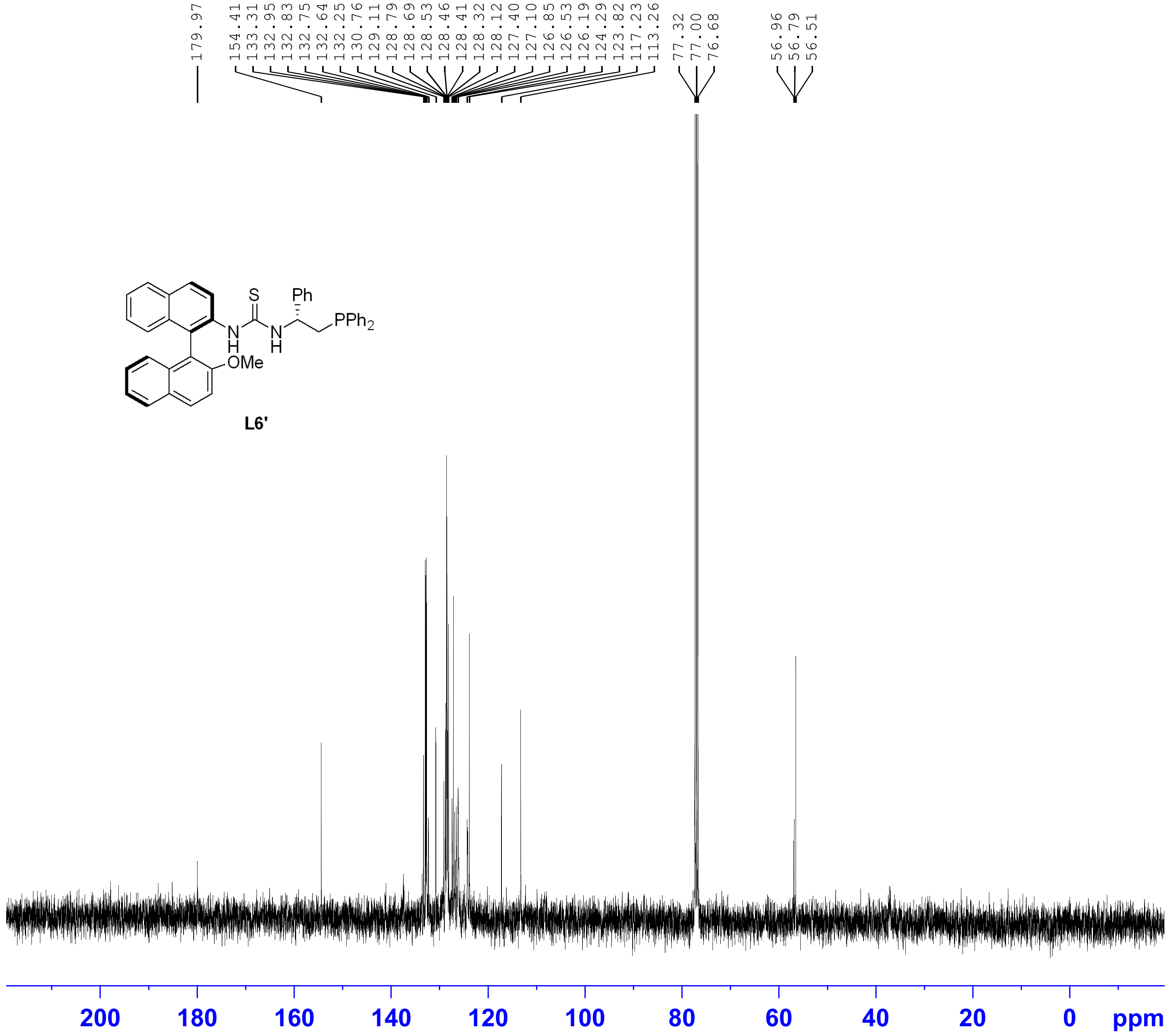

L6'

Current Data Parameters
NAME zmh-0909-1
EXPNO 3
PROCNO 1

F2 - Acquisition Parameters
Date_ 20250908
Time 22.11
INSTRUM spect
PROBHD 5 mm PABBO BB/
PULPROG zgpg30
TD 65536
SOLVENT CDCl3
NS 131
DS 2
SWH 24038.461 Hz
FIDRES 0.366798 Hz
AQ 1.3631488 sec
RG 196.92
DW 20.800 usec
DE 6.50 usec
TE 297.2 K
D1 2.00000000 sec
D11 0.03000000 sec
TD0 1

======== CHANNEL f1 ========
SFO1 100.6228298 MHz
NUC1 13C
P1 9.70 usec
PLW1 46.98899841 W

======== CHANNEL f2 ========
SFO2 400.1316005 MHz
NUC2 1H
CPDPRG[2 waltz16
PCPD2 90.00 usec
PLW2 11.99499989 W
PLW12 0.34213999 W
PLW13 0.27713001 W

F2 - Processing parameters
SI 32768
SF 100.6127777 MHz
WDW EM
SSB 0
LB 1.00 Hz
GB 0
PC 1.40

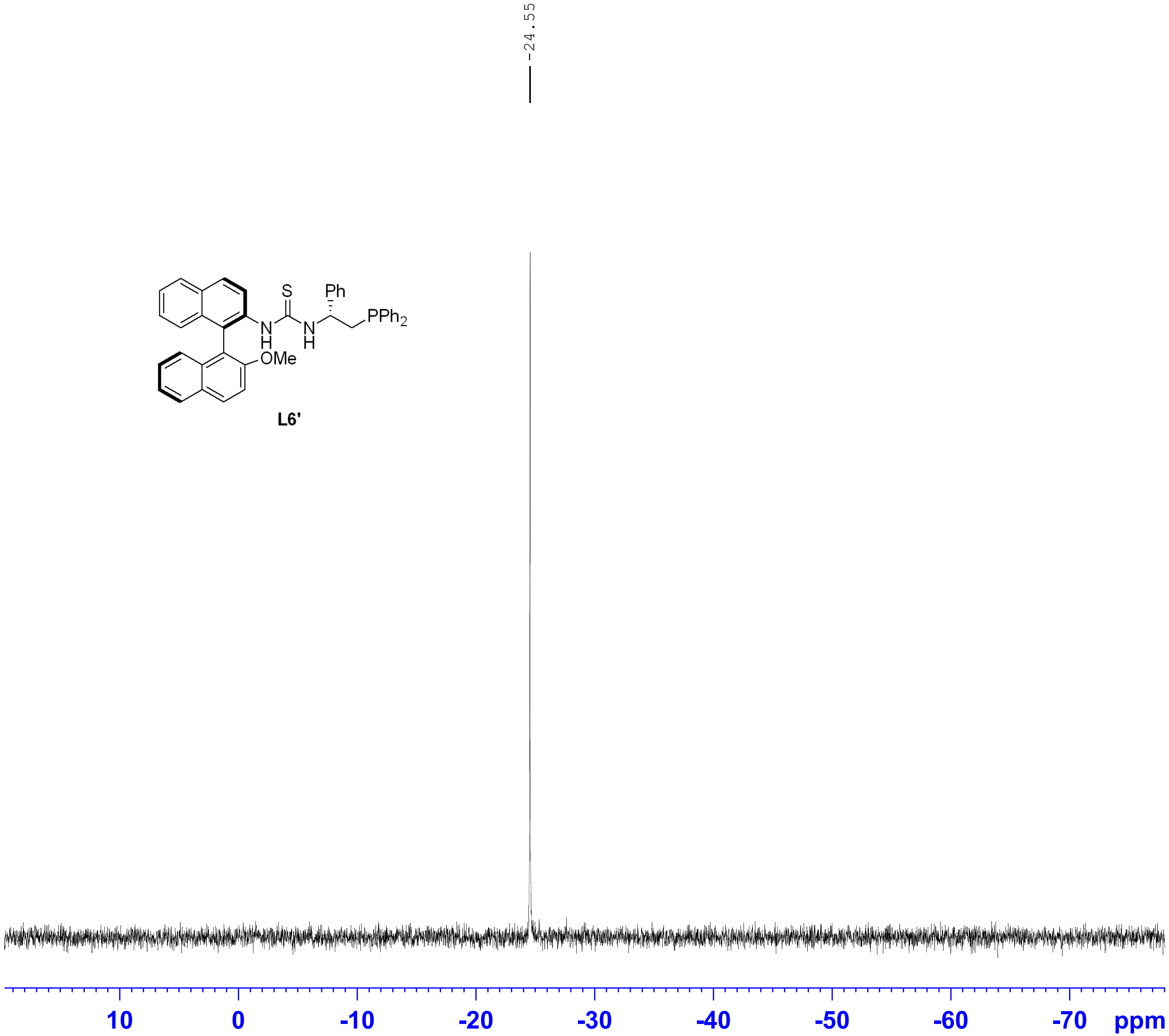


```
Current Data Parameters
NAME          zmh-0909-1
EXPNO                  2
PROCNO                 1

F2 - Acquisition Parameters
Date_           20250908
Time               22.09
INSTRUM            spect
PROBHD   5 mm PABBO BB/
PULPROG           zgpg30
TD                 65536
SOLVENT            CDCl3
NS                    11
DS                     4
SWH            64102.563 Hz
FIDRES          0.978127 Hz
AQ             0.5111808 sec
RG                196.92
DW                 7.800 usec
DE                  6.50 usec
TE                 297.0 K
D1            2.00000000 sec
D11           0.03000000 sec
TD0                    1

======== CHANNEL f1 ========
SFO1         161.9674942 MHz
NUC1                 31P
P1                 14.70 usec
PLW1         11.99499989 W

======== CHANNEL f2 ========
SFO2         400.1316005 MHz
NUC2                  1H
CPDPRG[2         waltz16
PCPD2              90.00 usec
PLW2         11.99499989 W
PLW12         0.34213999 W
PLW13         0.27713001 W

F2 - Processing parameters
SI                 32768
SF           161.9755930 MHz
WDW                   EM
SSB                    0
LB                  1.00 Hz
GB                     0
PC                  1.40
```

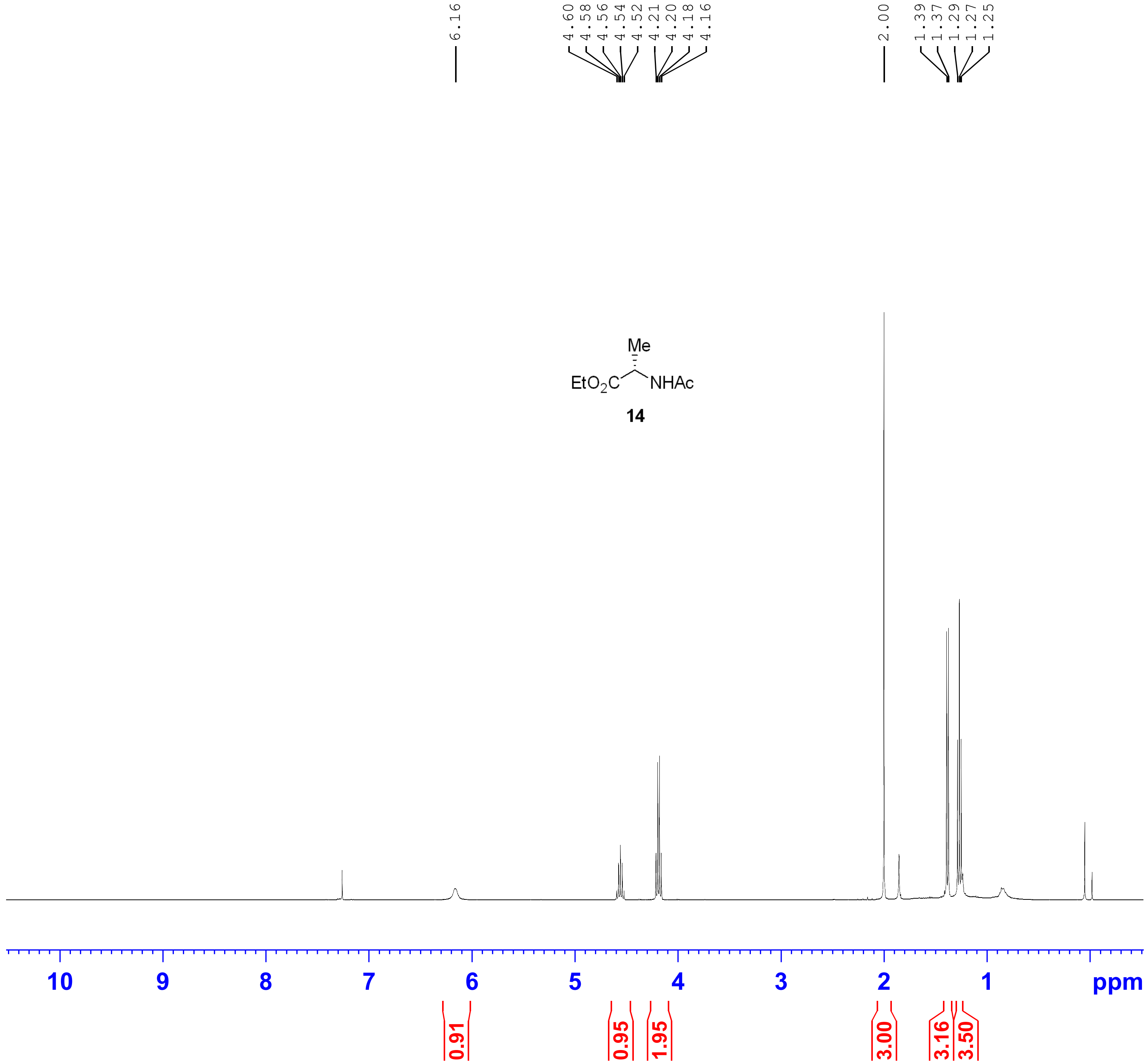


```
Current Data Parameters
NAME          zrh-5-35-h
EXPNO                  1
PROCNO                 1

F2 - Acquisition Parameters
Date_           20211221
Time               20.20
INSTRUM            spect
PROBHD   5 mm DUL 13C-1
PULPROG             zg30
TD                 65536
SOLVENT            CDCl3
NS                     4
DS                     0
SWH             8223.685 Hz
FIDRES          0.125483 Hz
AQ             3.9845889 sec
RG                   228
DW                60.800 usec
DE                  6.00 usec
TE                 293.5 K
D1            1.00000000 sec
TD0                    1

======== CHANNEL f1 ========
NUC1                  1H
P1                 15.80 usec
PL1                -1.00 dB
PL1W         12.17476940 W
SFO1         400.1324710 MHz

F2 - Processing parameters
SI                 32768
SF           400.1300099 MHz
WDW                   EM
SSB                    0
LB                  0.30 Hz
GB                     0
PC                  1.00
```

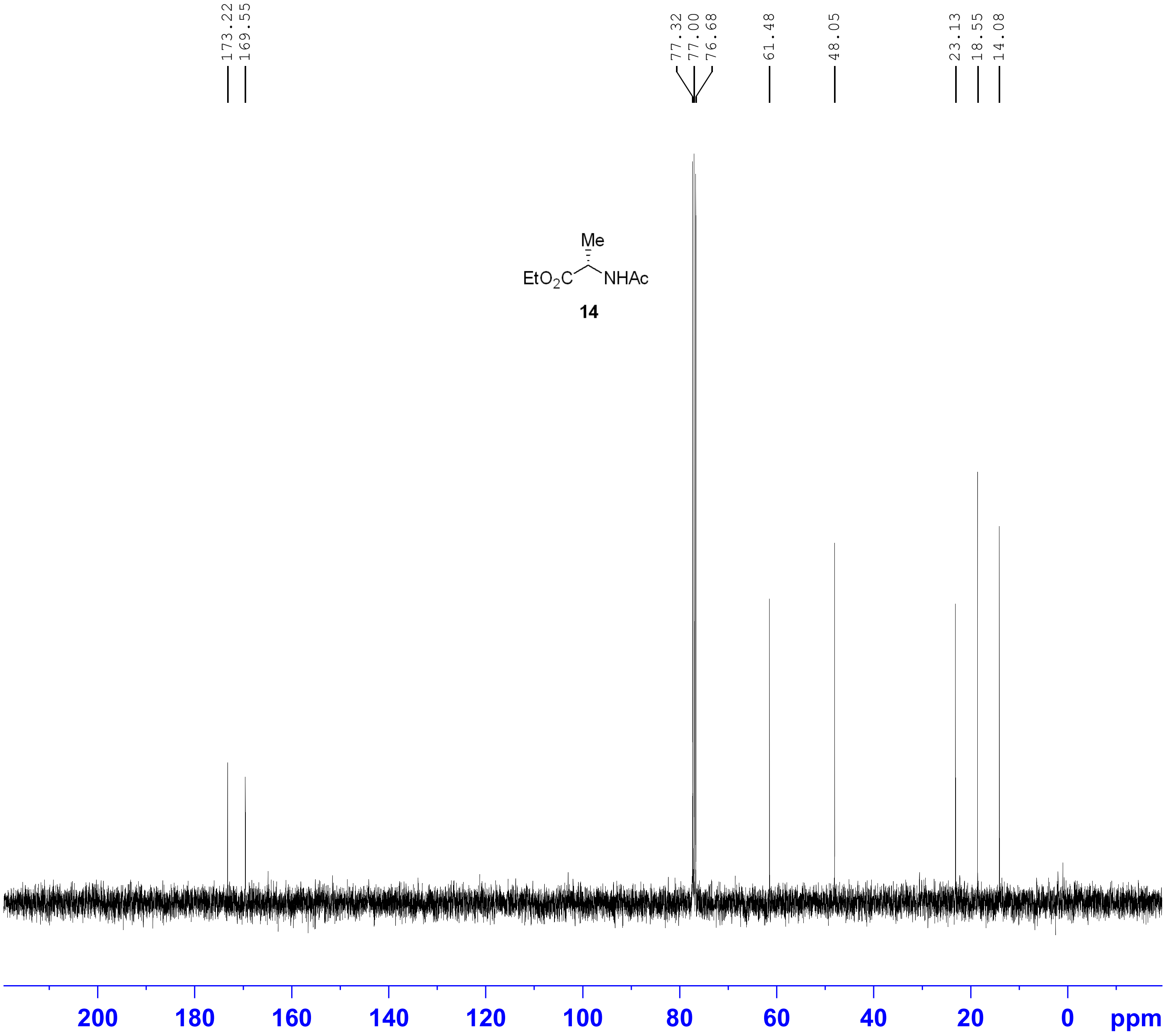

Current Data Parameters
NAME zrh-5-35-c
EXPNO 1
PROCNO 1

F2 - Acquisition Parameters
Date_ 20211221
Time 20.21
INSTRUM spect
PROBHD 5 mm DUL 13C-1
PULPROG zgpg30
TD 65536
SOLVENT CDCl3
NS 167
DS 0
SWH 24038.461 Hz
FIDRES 0.366798 Hz
AQ 1.3631488 sec
RG 2050
DW 20.800 usec
DE 6.00 usec
TE 293.7 K
D1 2.00000000 sec
D11 0.03000000 sec
TD0 1

======== CHANNEL f1 ========
NUC1 13C
P1 40.00 usec
PL1 -3.00 dB
PL1W 60.64365387 W
SFO1 100.6228298 MHz

======== CHANNEL f2 ========
CPDPRG[2 waltz16
NUC2 1H
PCPD2 80.00 usec
PL2 -1.00 dB
PL12 14.39 dB
PL13 18.00 dB
PL2W 12.17476940 W
PL12W 0.35193357 W
PL13W 0.15327126 W
SFO2 400.1316005 MHz

F2 - Processing parameters
SI 32768
SF 100.6127737 MHz
WDW EM
SSB 0
LB 1.00 Hz
GB 0
PC 1.40

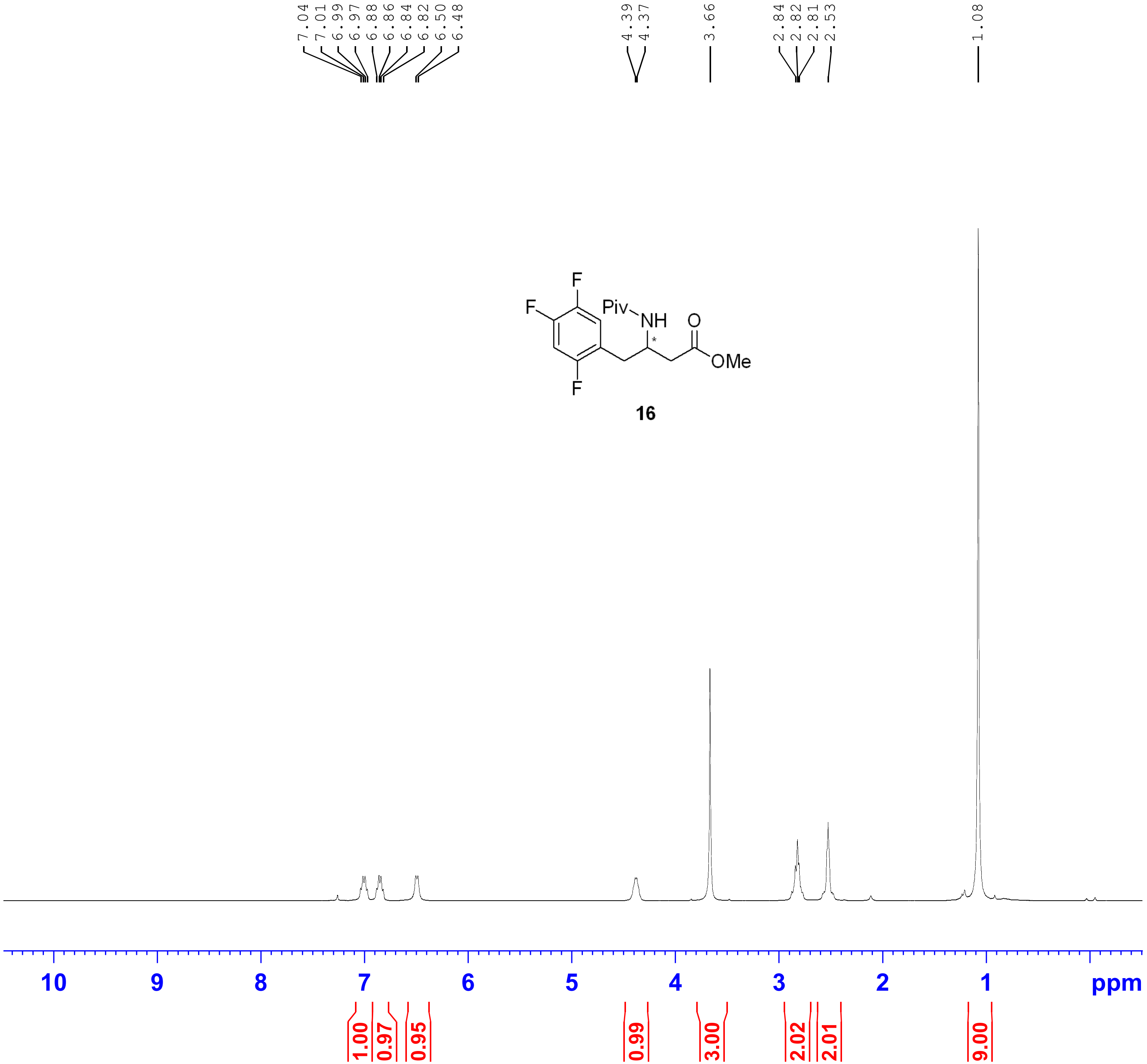

16

```
Current Data Parameters
NAME          zrh-8-103-h
EXPNO                   1
PROCNO                  1

F2 - Acquisition Parameters
Date_            20221231
Time                18.40
INSTRUM             spect
PROBHD     5 mm PABBO BB/
PULPROG              zg30
TD                  65536
SOLVENT             CDCl3
NS                      3
DS                      2
SWH              8012.820 Hz
FIDRES           0.122266 Hz
AQ              4.0894465 sec
RG                  22.47
DW                 62.400 usec
DE                   6.50 usec
TE                  293.7 K
D1             1.00000000 sec
TD0                     1

======== CHANNEL f1 ========
SFO1          400.1324710 MHz
NUC1                   1H
P1                  14.50 usec
PLW1          11.99499989 W

F2 - Processing parameters
SI                  65536
SF            400.1300099 MHz
WDW                    EM
SSB                     0
LB                   0.30 Hz
GB                      0
PC                   1.00
```

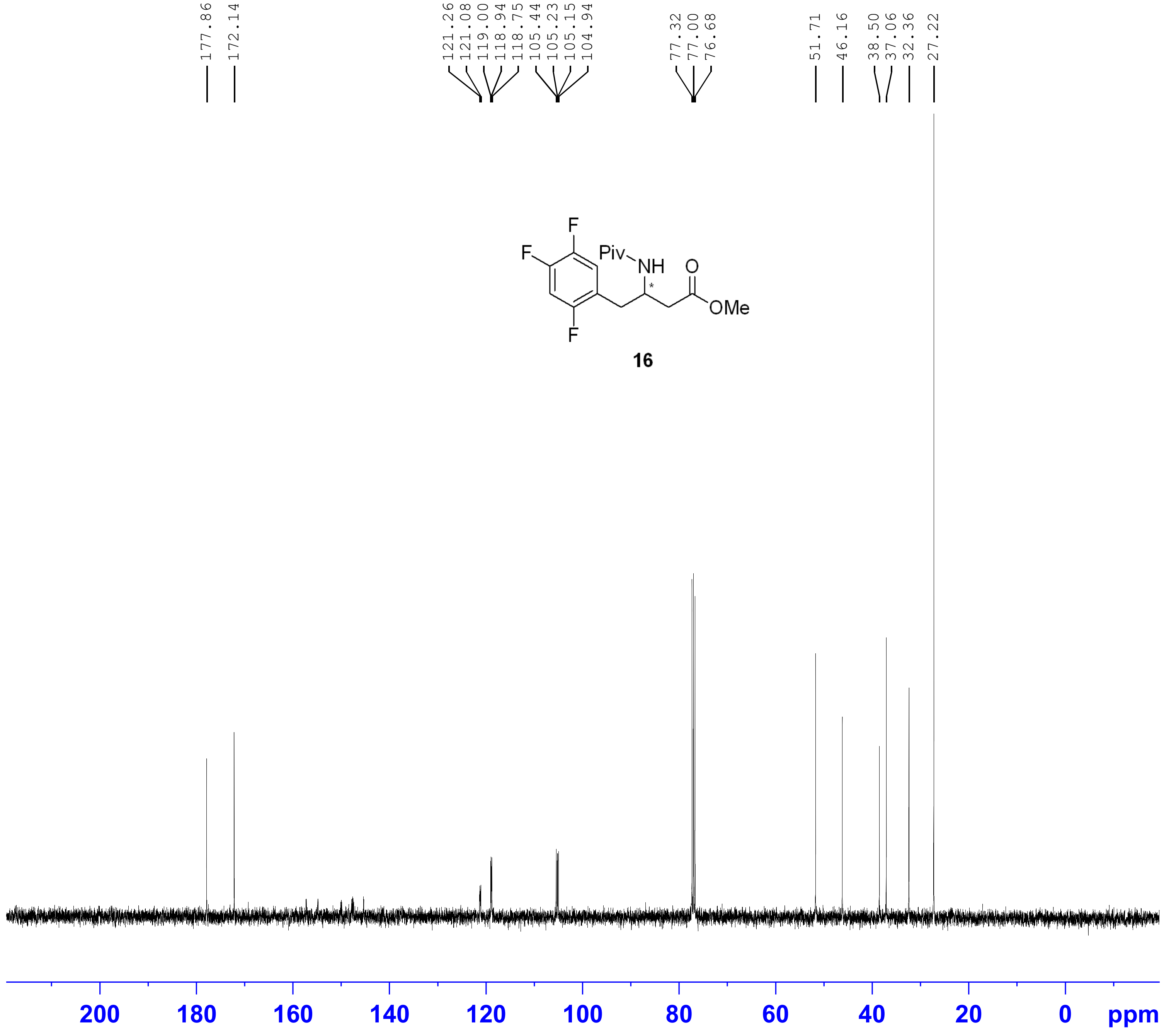


```
Current Data Parameters
NAME          zrh-8-103-c2
EXPNO                    1
PROCNO                   1

F2 - Acquisition Parameters
Date_             20221231
Time                 18.47
INSTRUM              spect
PROBHD    5 mm PABBO BB/
PULPROG             zgpg30
TD                   65536
SOLVENT              CDCl3
NS                      27
DS                       2
SWH              24038.461 Hz
FIDRES            0.366798 Hz
AQ               1.3631488 sec
RG                  196.92
DW                  20.800 usec
DE                    6.50 usec
TE                   294.4 K
D1              2.00000000 sec
D11             0.03000000 sec
TD0                      1

======== CHANNEL f1 ========
SFO1          100.6228298 MHz
NUC1                   13C
P1                    9.70 usec
PLW1           46.98899841 W

======== CHANNEL f2 ========
SFO2          400.1316005 MHz
NUC2                    1H
CPDPRG[2           waltz16
PCPD2                90.00 usec
PLW2           11.99499989 W
PLW12           0.34213999 W
PLW13           0.27713001 W

F2 - Processing parameters
SI                   32768
SF            100.6127765 MHz
WDW                     EM
SSB                      0
LB                    1.00 Hz
GB                       0
PC                    1.40
```

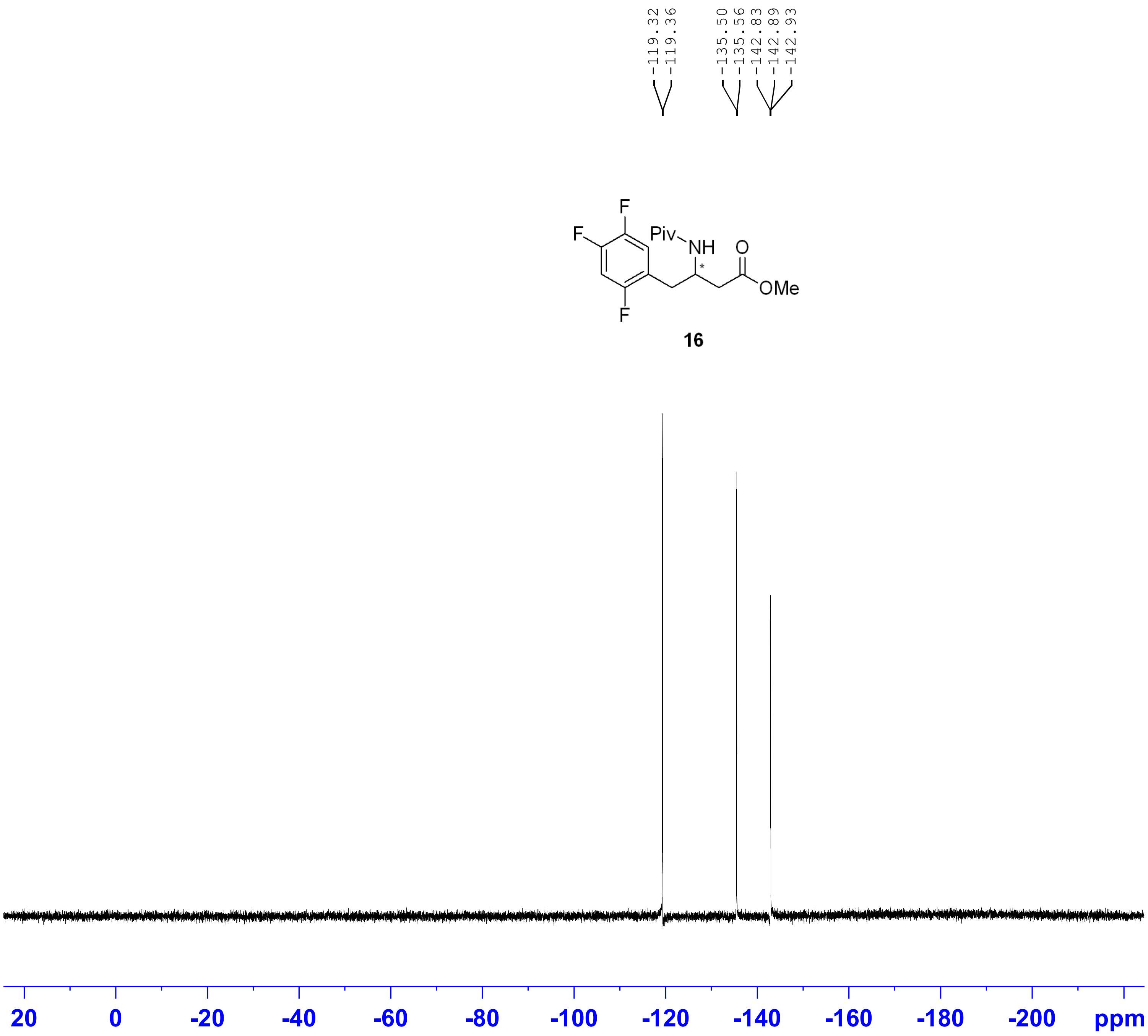


```
Current Data Parameters
NAME          zrh-8-103-f
EXPNO                   1
PROCNO                  1

F2 - Acquisition Parameters
Date_            20221231
Time                18.41
INSTRUM             spect
PROBHD   5 mm PABBO BB/
PULPROG           zgpg30
TD                  65536
SOLVENT             CDCl3
NS                      3
DS                      2
SWH             93750.000 Hz
FIDRES           1.430511 Hz
AQ              0.3495253 sec
RG                 196.92
DW                  5.333 usec
DE                   6.50 usec
TE                  293.9 K
D1             2.00000000 sec
D11            0.03000000 sec
TD0                     1

======== CHANNEL f1 ========
SFO1          376.4607162 MHz
NUC1                  19F
P1                  14.70 usec
PLW1          15.99600029 W

======== CHANNEL f2 ========
SFO2          400.1316005 MHz
NUC2                   1H
CPDPRG[2          waltz16
PCPD2               90.00 usec
PLW2          11.99499989 W
PLW12          0.34213999 W
PLW13          0.27713001 W

F2 - Processing parameters
SI                  32768
SF            376.4983660 MHz
WDW                    EM
SSB                     0
LB                   1.00 Hz
GB                      0
PC                   1.40
```

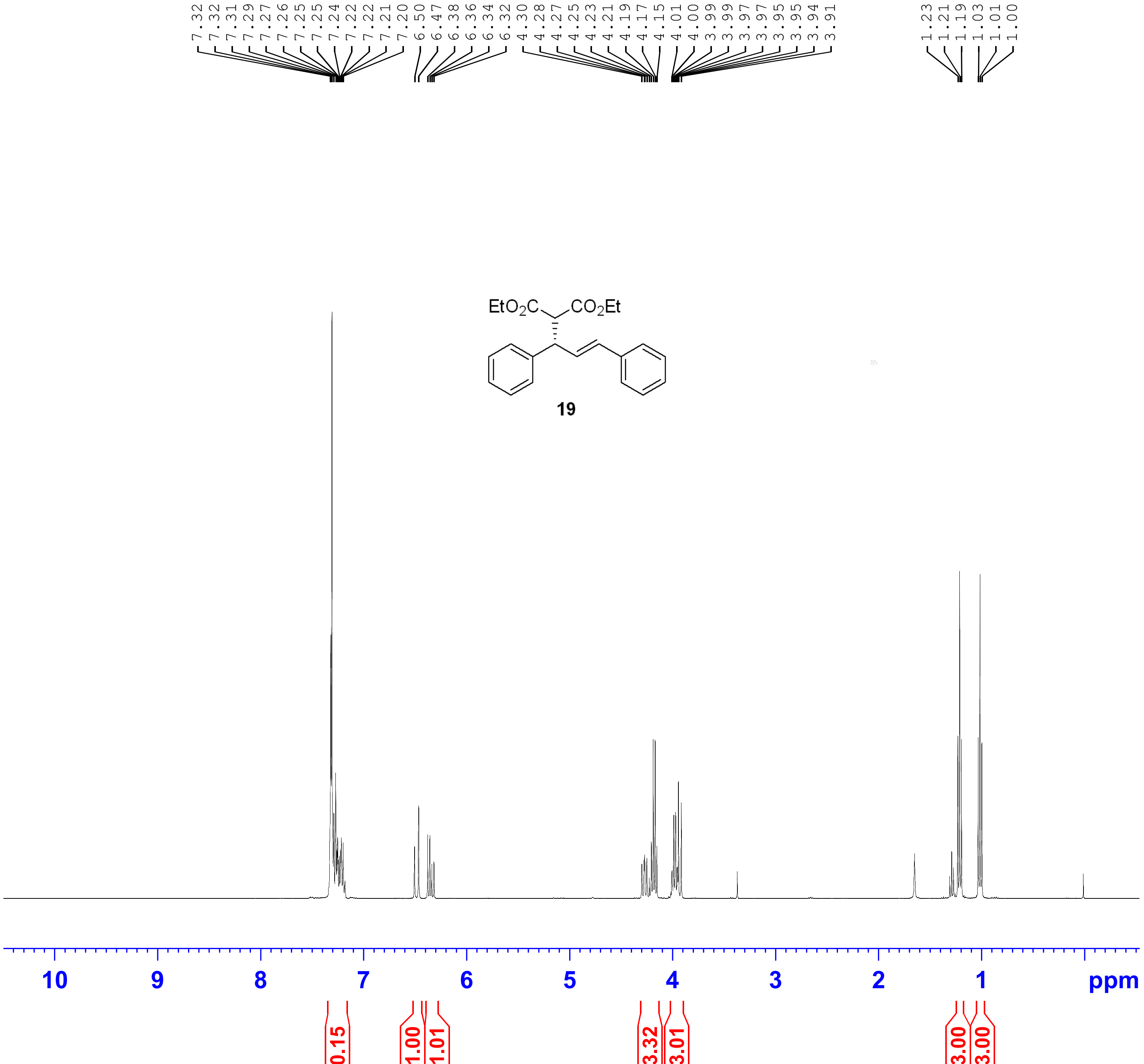

Current Data Parameters
NAME zrh-9-135-et-h
EXPNO 1
PROCNO 1

F2 - Acquisition Parameters
Date_ 20230528
Time 21.01
INSTRUM spect
PROBHD 5 mm PABBO BB/
PULPROG zg30
TD 65536
SOLVENT CDCl3
NS 11
DS 2
SWH 8012.820 Hz
FIDRES 0.122266 Hz
AQ 4.0894465 sec
RG 34.77
DW 62.400 usec
DE 6.50 usec
TE 296.5 K
D1 1.00000000 sec
TD0 1

======== CHANNEL f1 ========
SFO1 400.1324710 MHz
NUC1 1H
P1 14.50 usec
PLW1 11.99499989 W

F2 - Processing parameters
SI 65536
SF 400.1300100 MHz
WDW EM
SSB 0
LB 0.30 Hz
GB 0
PC 1.00

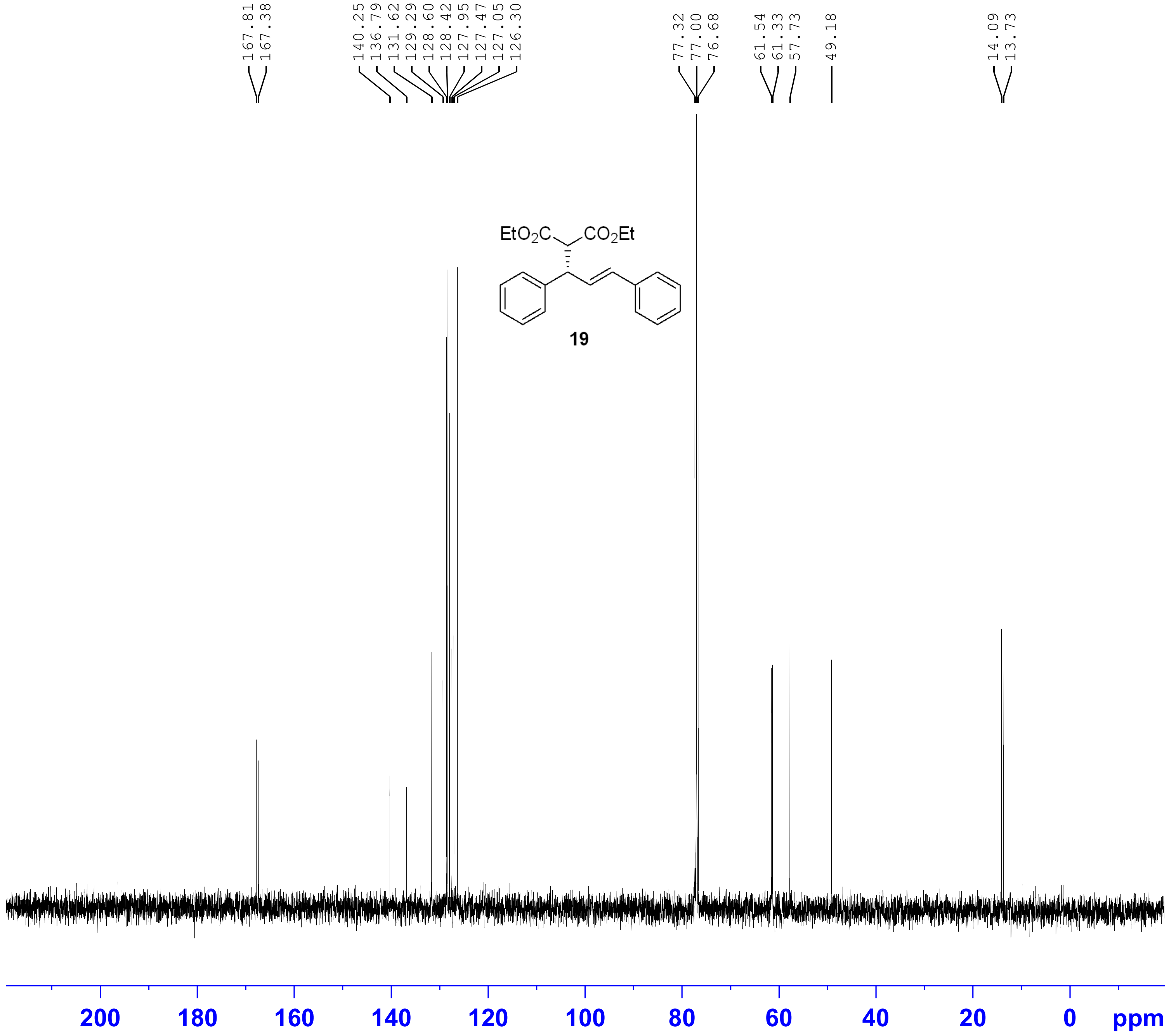

19

Current Data Parameters
NAME zrh-9-135-et-c
EXPNO 1
PROCNO 1

F2 - Acquisition Parameters
Date_ 20230528
Time 21.03
INSTRUM spect
PROBHD 5 mm PABBO BB/
PULPROG zgpg30
TD 65536
SOLVENT CDCl3
NS 32
DS 2
SWH 24038.461 Hz
FIDRES 0.366798 Hz
AQ 1.3631488 sec
RG 196.92
DW 20.800 usec
DE 6.50 usec
TE 297.1 K
D1 2.00000000 sec
D11 0.03000000 sec
TD0 1

======== CHANNEL f1 ========
SFO1 100.6228298 MHz
NUC1 13C
P1 9.70 usec
PLW1 46.98899841 W

======== CHANNEL f2 ========
SFO2 400.1316005 MHz
NUC2 1H
CPDPRG[2 waltz16
PCPD2 90.00 usec
PLW2 11.99499989 W
PLW12 0.34213999 W
PLW13 0.27713001 W

F2 - Processing parameters
SI 32768
SF 100.6127758 MHz
WDW EM
SSB 0
LB 1.00 Hz
GB 0
PC 1.40

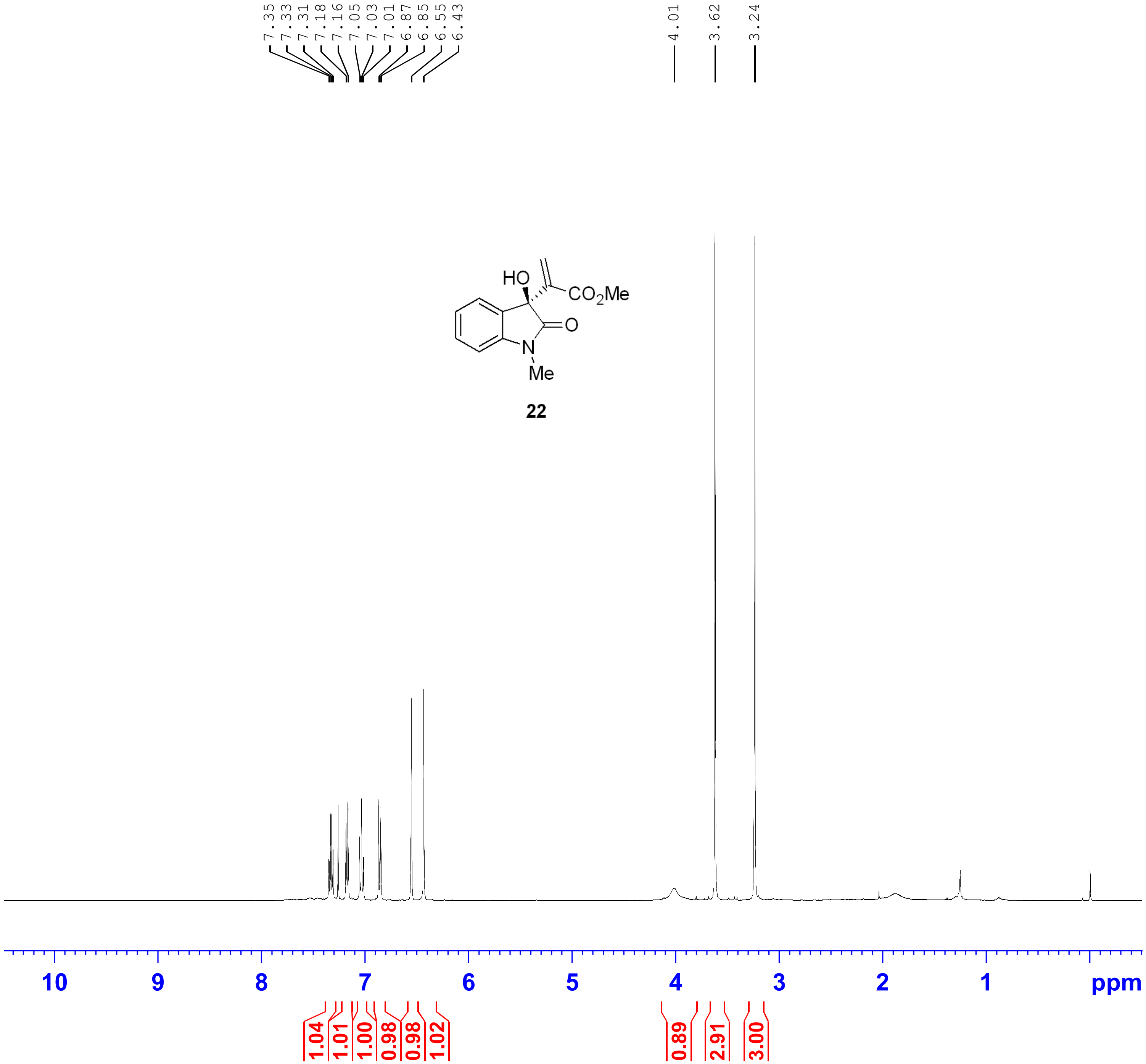

Current Data Parameters
NAME zrh-9-67-3-h
EXPNO 1
PROCNO 1

F2 - Acquisition Parameters
Date_ 20230507
Time 16.41 h
INSTRUM AvanceNeo 400MHz
PROBHD Z163739_0629 (
PULPROG zg30
TD 65536
SOLVENT CDCl3
NS 4
DS 2
SWH 8196.722 Hz
FIDRES 0.250144 Hz
AQ 3.9976959 sec
RG 101
DW 61.000 usec
DE 13.89 usec
TE 296.5 K
D1 1.00000000 sec
TD0 1
SFO1 400.1824711 MHz
NUC1 1H
P0 2.67 usec
P1 8.00 usec
PLW1 21.26700020 W

F2 - Processing parameters
SI 65536
SF 400.1800094 MHz
WDW EM
SSB 0
LB 0.30 Hz
GB 0
PC 1.00

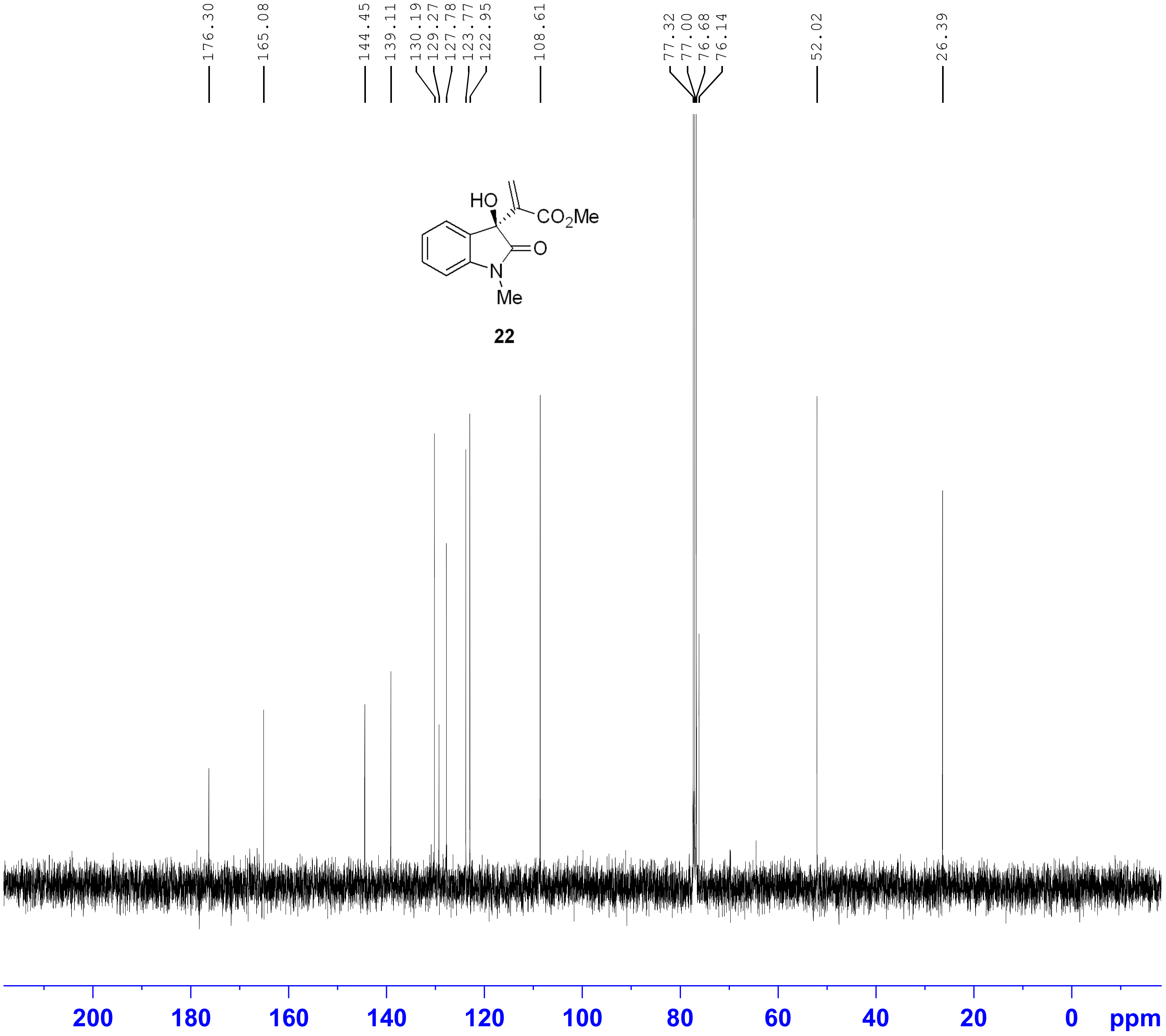

Current Data Parameters
NAME zrh-9-67-3-c
EXPNO 3
PROCNO 1

F2 - Acquisition Parameters
Date_ 20230507
Time 16.47 h
INSTRUM AvanceNeo 400MHz
PROBHD Z163739_0629 (
PULPROG zgpg30
TD 65536
SOLVENT CDCl3
NS 60
DS 4
SWH 23809.523 Hz
FIDRES 0.726609 Hz
AQ 1.3762560 sec
RG 11.3
DW 21.000 usec
DE 6.50 usec
TE 297.0 K
D1 2.00000000 sec
D11 0.03000000 sec
TD0 1
SFO1 100.6354036 MHz
NUC1 13C
P0 2.67 usec
P1 8.00 usec
PLW1 85.25399780 W
SFO2 400.1816007 MHz
NUC2 1H
CPDPRG[2 waltz65
PCPD2 90.00 usec
PLW2 21.26700020 W
PLW12 0.16802999 W
PLW13 0.08452000 W

F2 - Processing parameters
SI 32768
SF 100.6253470 MHz
WDW EM
SSB 0
LB 1.00 Hz
GB 0
PC 1.40

===

Acq. Operator :	Seq. Line : 10
Acq. Instrument : Instrument 1	Location : Vial 43
Injection Date : 3/17/2021 3:34:18 PM	Inj : 1
	Inj Volume : 5.000 µl

Acq. Method : C:\CHEM32\1\DATA\QDY 2021-03-17 13-07-57\AD-30-20.M
Last changed : 3/17/2021 2:51:23 PM
(modified after loading)
Analysis Method : C:\CHEM32\1\DATA\SUN_12 2023-05-12 21-33-41\OD-10-30.M
Last changed : 6/20/2023 7:52:55 PM
(modified after loading)
Additional Info : Peak(s) manually integrated

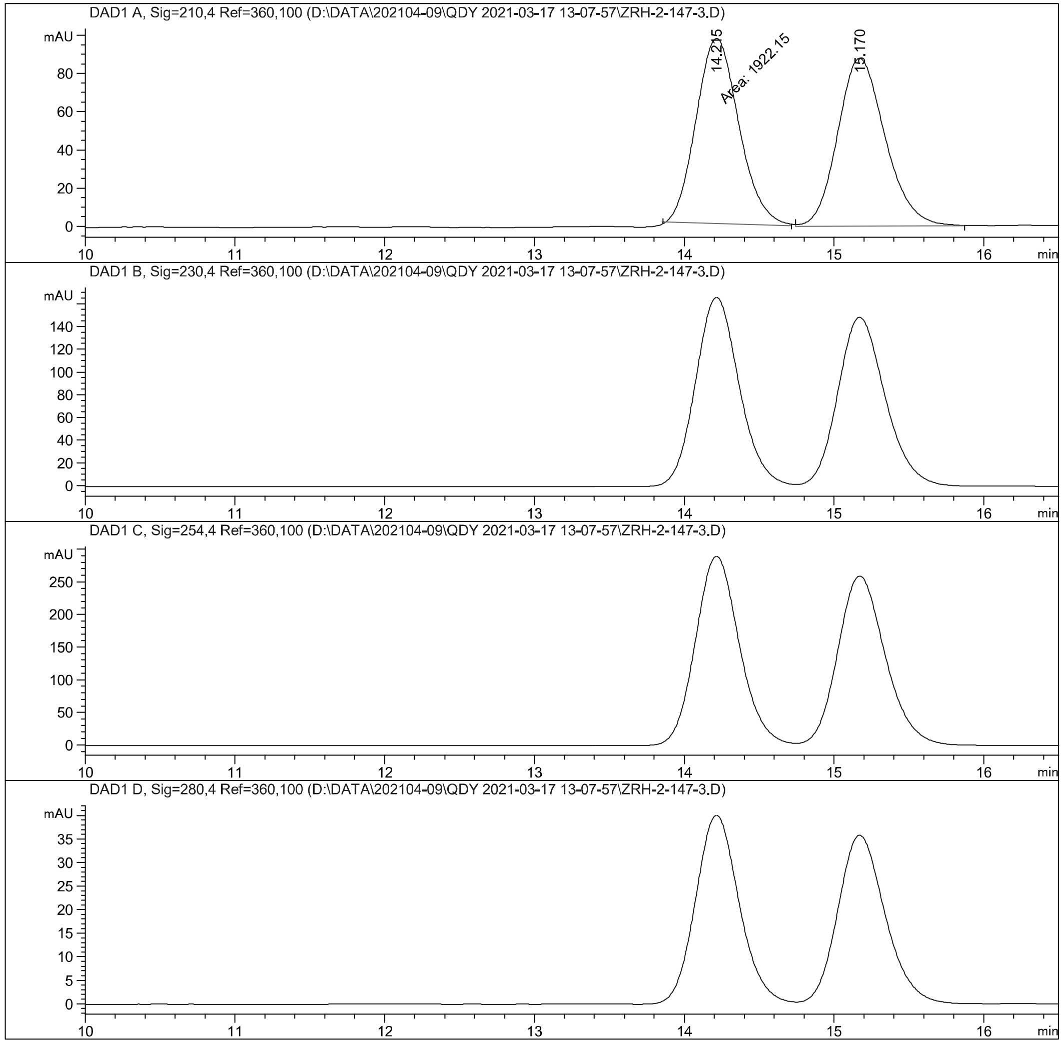

Data File D:\DATA\202104-09\QDY 2021-03-17 13-07-57\ZRH-2-147-3.D
Sample Name:

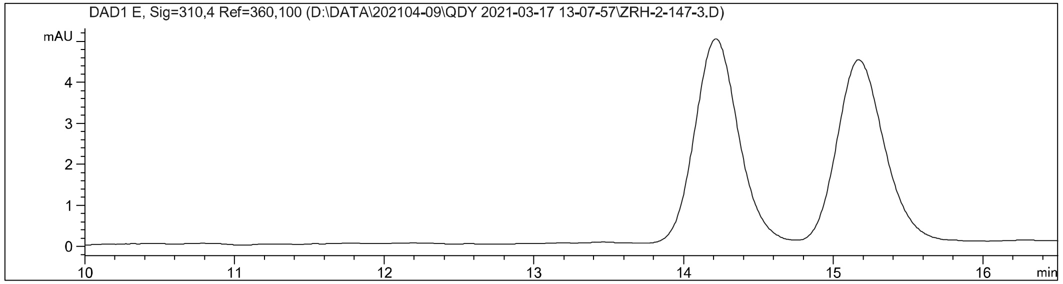

===
Area Percent Report
===

Sorted By : Signal
Multiplier : 1.0000
Dilution : 1.0000
Use Multiplier & Dilution Factor with ISTDs

Signal 1: DAD1 A, Sig=210,4 Ref=360,100

Peak #	RetTime [min]	Type	Width [min]	Area [mAU*s]	Height [mAU]	Area %
1	14.215	MM	0.3301	1922.14746	97.04505	49.8400
2	15.170	VB	0.3387	1934.49109	87.91220	50.1600

Totals : 3856.63855 184.95725

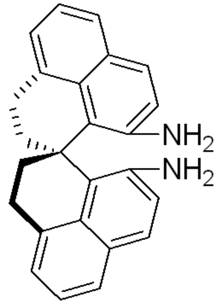

1a
(racemic)

Signal 2: DAD1 B, Sig=230,4 Ref=360,100

Signal 3: DAD1 C, Sig=254,4 Ref=360,100

Signal 4: DAD1 D, Sig=280,4 Ref=360,100

Signal 5: DAD1 E, Sig=310,4 Ref=360,100

===
*** End of Report ***

===

Acq. Operator : Seq. Line : 38
Acq. Instrument : Instrument 1 Location : Vial 41
Injection Date : 6/22/2021 8:35:30 PM Inj : 1
Inj Volume : 5.000 µl
Different Inj Volume from Sequence ! Actual Inj Volume : 10.000 µl
Acq. Method : C:\CHEM32\1\DATA\SUN_12 2021-06-22 09-29-00\AD-30-20.M
Last changed : 6/22/2021 8:34:37 PM
(modified after loading)
Analysis Method : C:\CHEM32\1\DATA\SUN_12 2023-05-12 21-33-41\OD-10-30.M
Last changed : 6/20/2023 8:06:40 PM
(modified after loading)
Additional Info : Peak(s) manually integrated

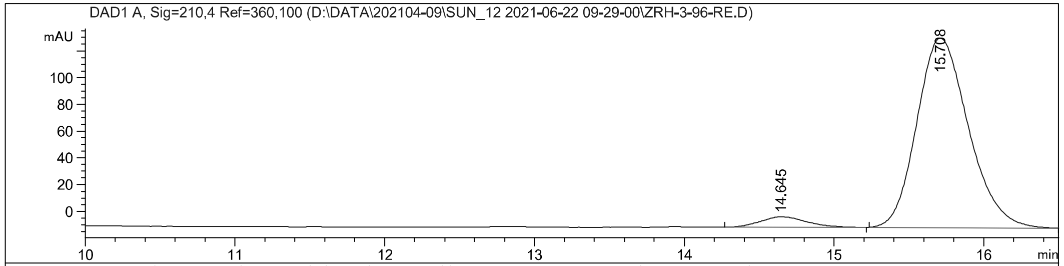

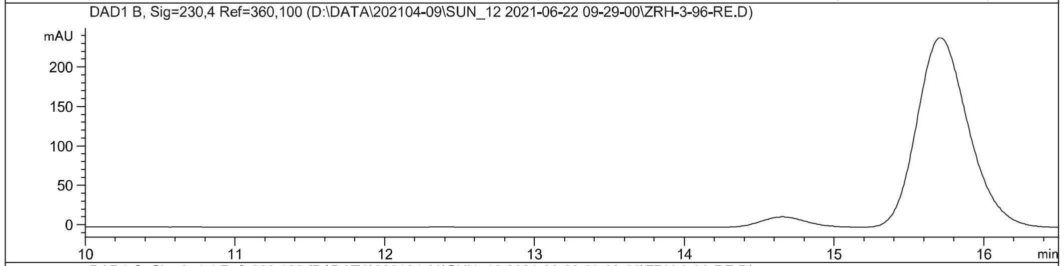

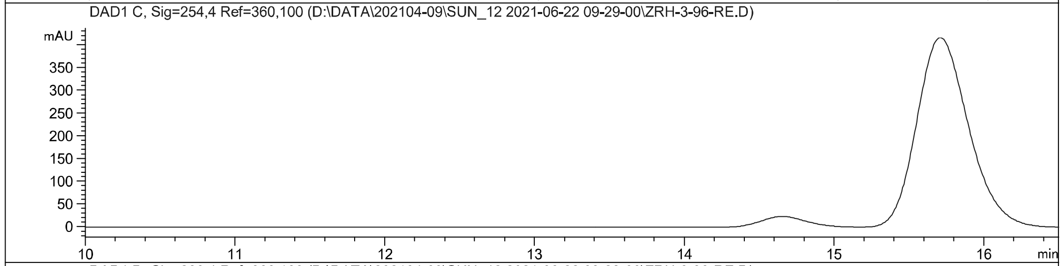

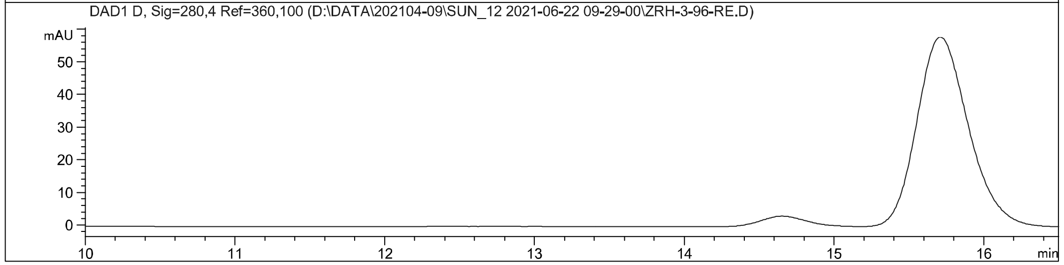

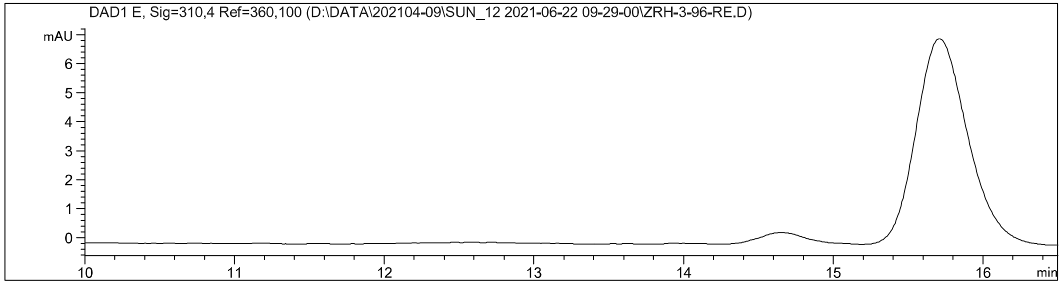

===

Area Percent Report

===

Sorted By : Signal
Multiplier : 1.0000
Dilution : 1.0000
Use Multiplier & Dilution Factor with ISTDs

Signal 1: DAD1 A, Sig=210,4 Ref=360,100

Peak #	RetTime [min]	Type	Width [min]	Area [mAU*s]	Height [mAU]	Area %
1	14.645	BB	0.3089	173.61374	7.88506	4.8483
2	15.708	BB	0.3680	3407.30151	141.95224	95.1517

Totals : 3580.91525 149.83730

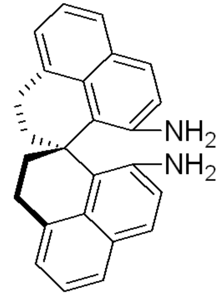

1a
(enantioenriched)

Signal 2: DAD1 B, Sig=230,4 Ref=360,100

Signal 3: DAD1 C, Sig=254,4 Ref=360,100

Signal 4: DAD1 D, Sig=280,4 Ref=360,100

Signal 5: DAD1 E, Sig=310,4 Ref=360,100

===

*** End of Report ***

===

```
Acq. Operator   :                                  Seq. Line :   2
Acq. Instrument : Instrument 1                      Location : Vial 41
Injection Date  : 8/28/2021 3:19:03 PM                   Inj :   1
                                                  Inj Volume : 5.000 µl
Different Inj Volume from Sequence !     Actual Inj Volume : 10.000 µl
Acq. Method     : C:\CHEM32\1\DATA\SUN_12 2021-08-28 15-11-07\AD-30-20.M
Last changed    : 8/28/2021 3:34:59 PM
                  (modified after loading)
Analysis Method : C:\CHEM32\1\DATA\SUN_12 2023-05-12 21-33-41\OD-10-30.M
Last changed    : 6/20/2023 7:50:57 PM
                  (modified after loading)
Additional Info : Peak(s) manually integrated
```

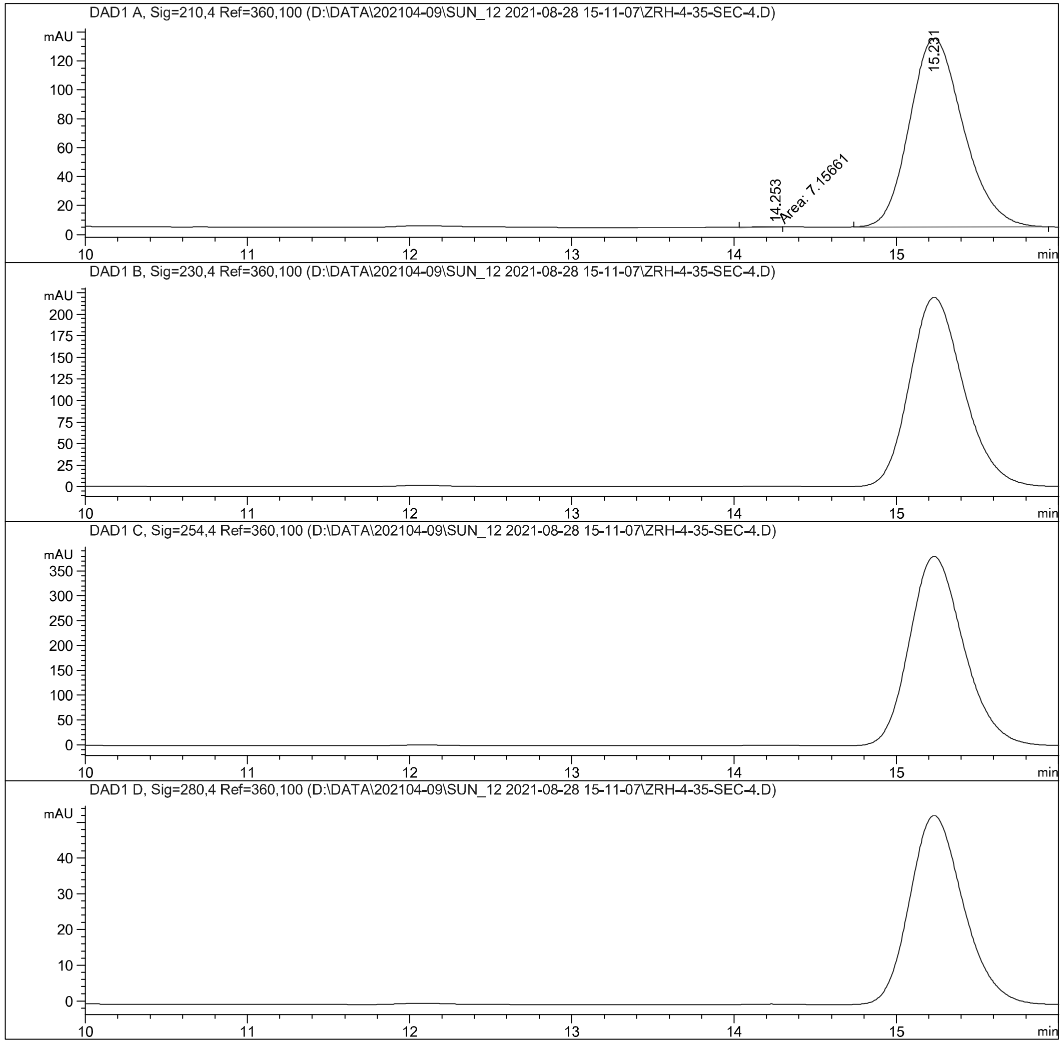

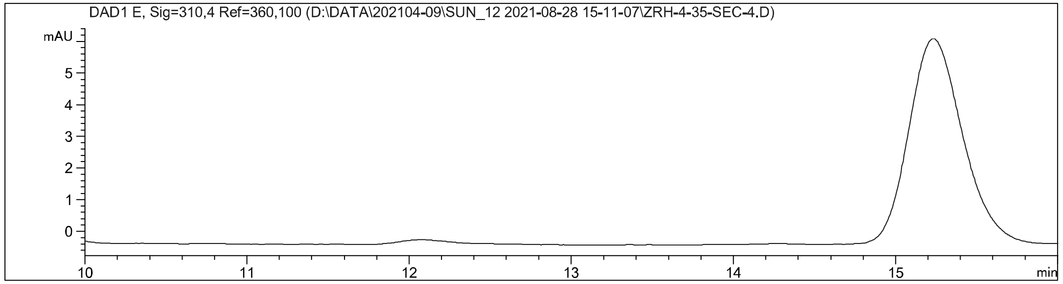

===
Area Percent Report
===

```
Sorted By             :      Signal
Multiplier            :      1.0000
Dilution              :      1.0000
Use Multiplier & Dilution Factor with ISTDs
```

Signal 1: DAD1 A, Sig=210,4 Ref=360,100

Peak #	RetTime [min]	Type	Width [min]	Area [mAU*s]	Height [mAU]	Area %
1	14.253	MM	0.2370	7.15661	5.03280e-1	0.2377
2	15.231	BB	0.3545	3003.68872	130.51895	99.7623

Totals : 3010.84533 131.02223

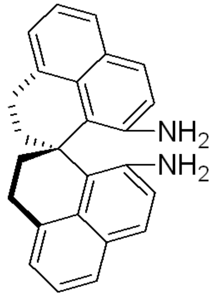

1a
(enantioenriched)

Signal 2: DAD1 B, Sig=230,4 Ref=360,100

Signal 3: DAD1 C, Sig=254,4 Ref=360,100

Signal 4: DAD1 D, Sig=280,4 Ref=360,100

Signal 5: DAD1 E, Sig=310,4 Ref=360,100

===
*** End of Report ***

===

Acq. Operator : Seq. Line : 16
Acq. Instrument : Instrument 1 Location : Vial 31
Injection Date : 6/8/2022 3:51:28 PM Inj : 1
Inj Volume : 5.000 µl
Different Inj Volume from Sequence ! Actual Inj Volume : 7.000 µl
Acq. Method : C:\CHEM32\1\DATA\SUN_12 2022-06-08 09-43-18\OD-20-40.M
Last changed : 6/8/2022 3:50:36 PM
(modified after loading)
Analysis Method : C:\CHEM32\1\DATA\SUN_12 2022-12-29 09-07-21\2.D\DA.M (OD-05-10.M)
Last changed : 1/12/2023 9:14:44 PM
(modified after loading)
Additional Info : Peak(s) manually integrated

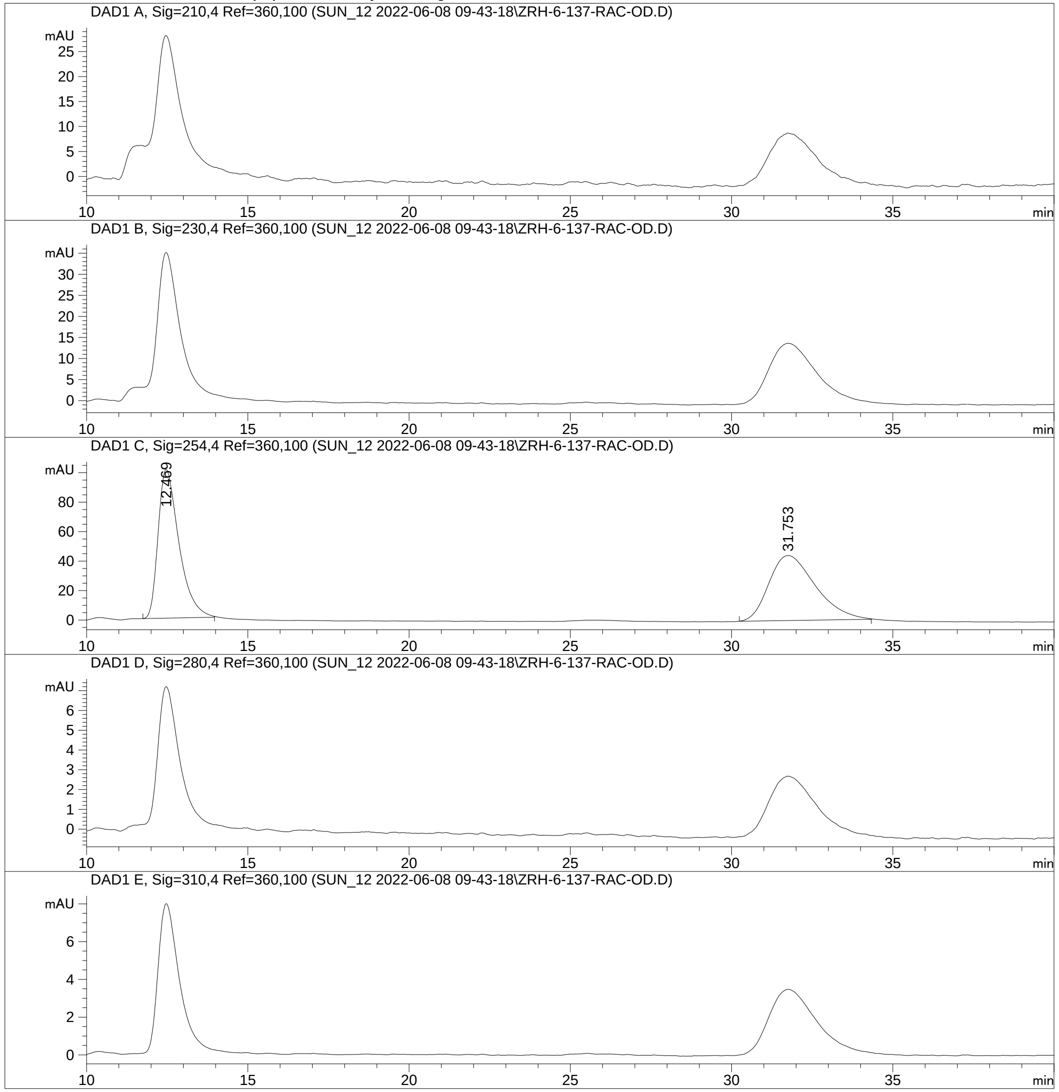

Data File C:\CHEM32\1\DATA\SUN_12 2022-06-08 09-43-18\ZRH-6-137-RAC-OD.D
Sample Name:

===

Area Percent Report

===

Sorted By : Signal
Multiplier : 1.0000
Dilution : 1.0000
Use Multiplier & Dilution Factor with ISTDs

Signal 1: DAD1 A, Sig=210,4 Ref=360,100

Signal 2: DAD1 B, Sig=230,4 Ref=360,100

Signal 3: DAD1 C, Sig=254,4 Ref=360,100

Peak #	RetTime [min]	Type	Width [min]	Area [mAU*s]	Height [mAU]	Area %
1	12.469	BB	0.6862	4530.90820	100.84908	50.6184
2	31.753	BB	1.4626	4420.20313	44.12996	49.3816

Totals : 8951.11133 144.97904

Signal 4: DAD1 D, Sig=280,4 Ref=360,100

Signal 5: DAD1 E, Sig=310,4 Ref=360,100

===

*** End of Report ***

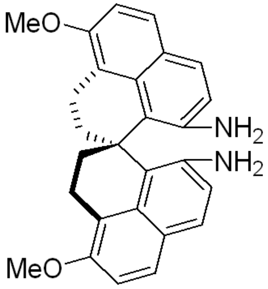

1b
(racemic)

===

Acq. Operator   :   Seq. Line :   9
Acq. Instrument : Instrument 1   Location : Vial 31
Injection Date : 6/9/2022 1:28:39 PM   Inj :   1
                                               Inj Volume : 5.000 µl
Different Inj Volume from Sequence !   Actual Inj Volume : 8.000 µl
Acq. Method   : C:\CHEM32\1\DATA\SUN_12 2022-06-09 10-18-41\OD-20-40.M
Last changed   : 6/9/2022 1:27:47 PM
                  (modified after loading)
Analysis Method : C:\CHEM32\1\DATA\SUN_12 2022-12-29 09-07-21\2.D\DA.M (OD-05-10.M)
Last changed   : 1/12/2023 9:16:19 PM
                  (modified after loading)
Additional Info : Peak(s) manually integrated

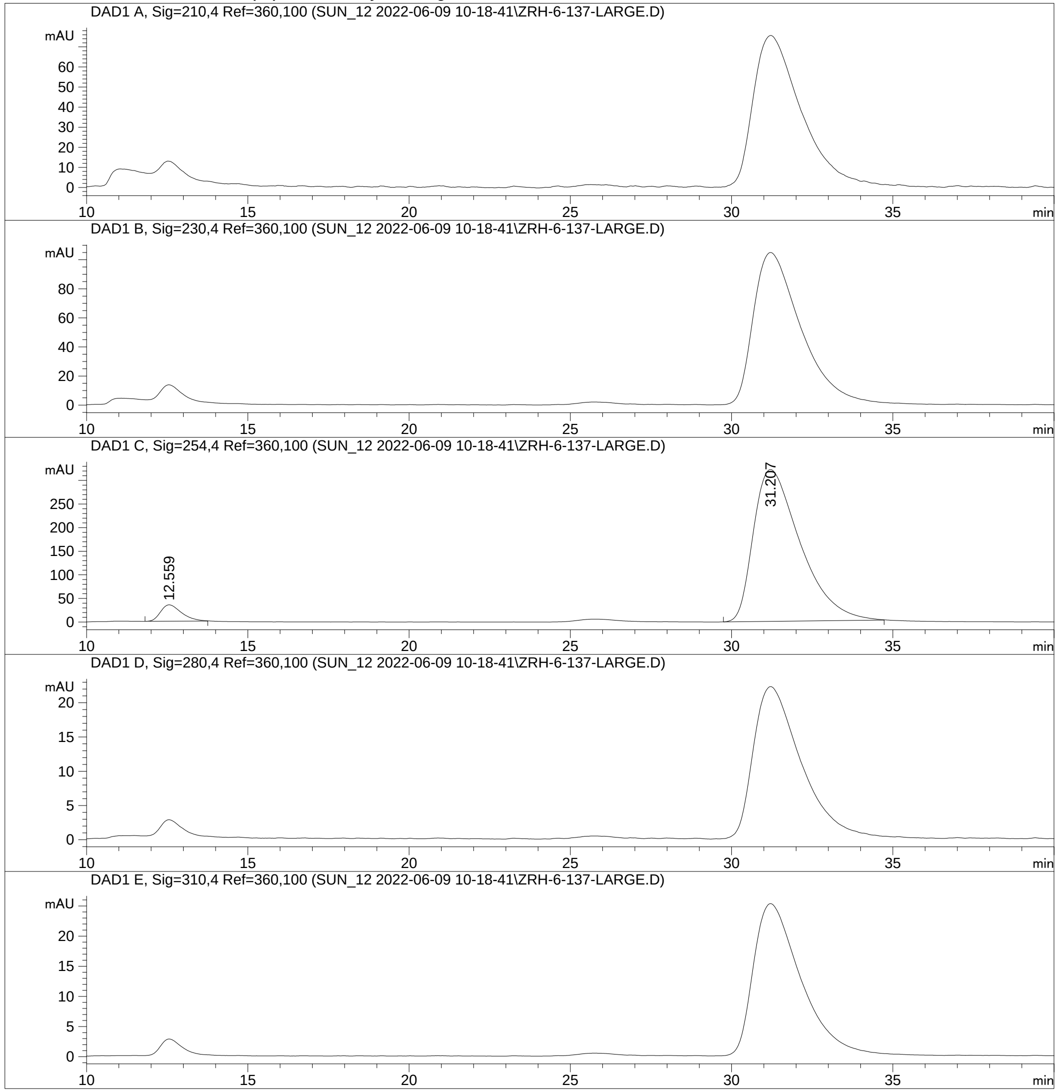

Data File C:\CHEM32\1\DATA\SUN_12 2022-06-09 10-18-41\ZRH-6-137-LARGE.D
Sample Name:

===

Area Percent Report

===

Sorted By   :   Signal
Multiplier   :   1.0000
Dilution   :   1.0000
Use Multiplier & Dilution Factor with ISTDs

Signal 1: DAD1 A, Sig=210,4 Ref=360,100

Signal 2: DAD1 B, Sig=230,4 Ref=360,100

Signal 3: DAD1 C, Sig=254,4 Ref=360,100

Peak #	RetTime [min]	Type	Width [min]	Area [mAU*s]	Height [mAU]	Area %
1	12.559	BB	0.6953	1569.22180	34.72053	4.6096
2	31.207	BB	1.5155	3.24732e4	321.73401	95.3904

Totals :   3.40424e4   356.45454

Signal 4: DAD1 D, Sig=280,4 Ref=360,100

Signal 5: DAD1 E, Sig=310,4 Ref=360,100

===

*** End of Report ***

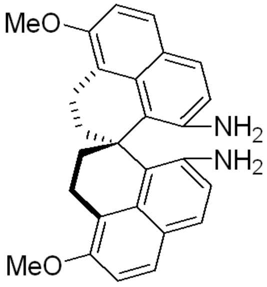

1b
(enantioenriched)

Data File C:\CHEM32\1\DATA\SUN_12 2022-07-01 14-53-17\ZRH-7-4-RAC.D
Sample Name:

===

Acq. Operator   :   Seq. Line : 26
Acq. Instrument : Instrument 1   Location : Vial 33
Injection Date : 7/2/2022 12:56:18 AM   Inj :   1
                                              Inj Volume : 5.000 µl
Different Inj Volume from Sequence !   Actual Inj Volume : 10.000 µl
Acq. Method   : C:\CHEM32\1\DATA\SUN_12 2022-07-01 14-53-17\AD-20-30.M
Last changed   : 6/15/2018 10:29:43 AM
Analysis Method : C:\CHEM32\1\DATA\SUN_12 2022-12-29 09-07-21\2.D\DA.M (OD-05-10.M)
Last changed   : 1/12/2023 9:44:10 PM
                  (modified after loading)
Additional Info : Peak(s) manually integrated

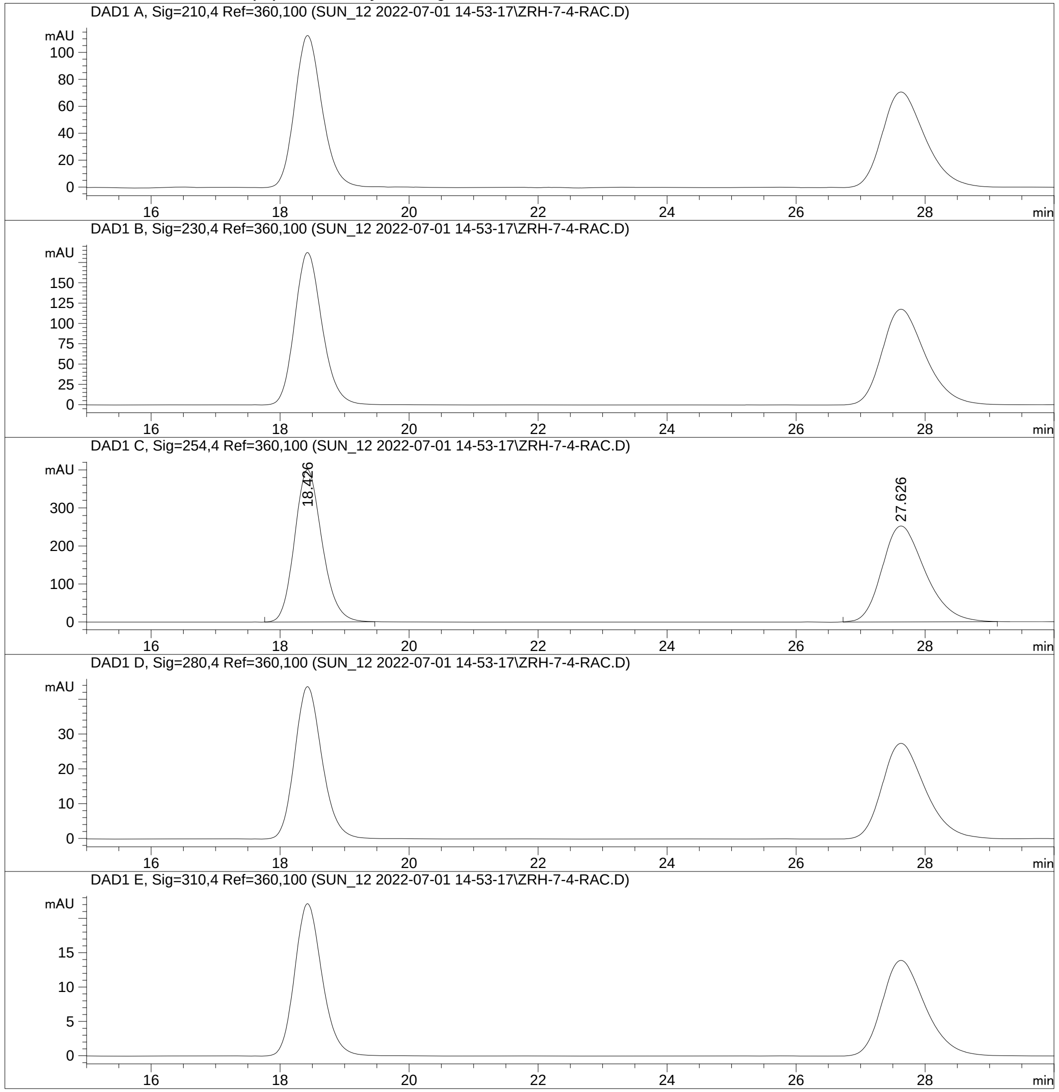

===

Area Percent Report

===

Sorted By   :   Signal
Multiplier   :   1.0000
Dilution   :   1.0000
Use Multiplier & Dilution Factor with ISTDs

Signal 1: DAD1 A, Sig=210,4 Ref=360,100

Signal 2: DAD1 B, Sig=230,4 Ref=360,100

Signal 3: DAD1 C, Sig=254,4 Ref=360,100

Peak #	RetTime [min]	Type	Width [min]	Area [mAU*s]	Height [mAU]	Area %
1	18.426	BB	0.4545	1.18403e4	400.96924	50.2248
2	27.626	BB	0.7141	1.17343e4	252.55818	49.7752

Totals :   2.35746e4   653.52742

Signal 4: DAD1 D, Sig=280,4 Ref=360,100

Signal 5: DAD1 E, Sig=310,4 Ref=360,100

===

*** End of Report ***

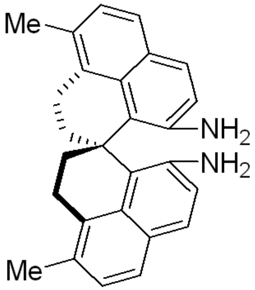

1c
(racemic)

===

Acq. Operator : Seq. Line : 28
Acq. Instrument : Instrument 1 Location : Vial 34
Injection Date : 7/2/2022 2:28:30 AM Inj : 1
Inj Volume : 5.000 µl
Different Inj Volume from Sequence ! Actual Inj Volume : 10.000 µl
Acq. Method : C:\CHEM32\1\DATA\SUN_12 2022-07-01 14-53-17\AD-20-30.M
Last changed : 6/15/2018 10:29:43 AM
Analysis Method : C:\CHEM32\1\DATA\SUN_12 2022-12-29 09-07-21\2.D\DA.M (OD-05-10.M)
Last changed : 1/12/2023 9:44:10 PM
(modified after loading)
Additional Info : Peak(s) manually integrated

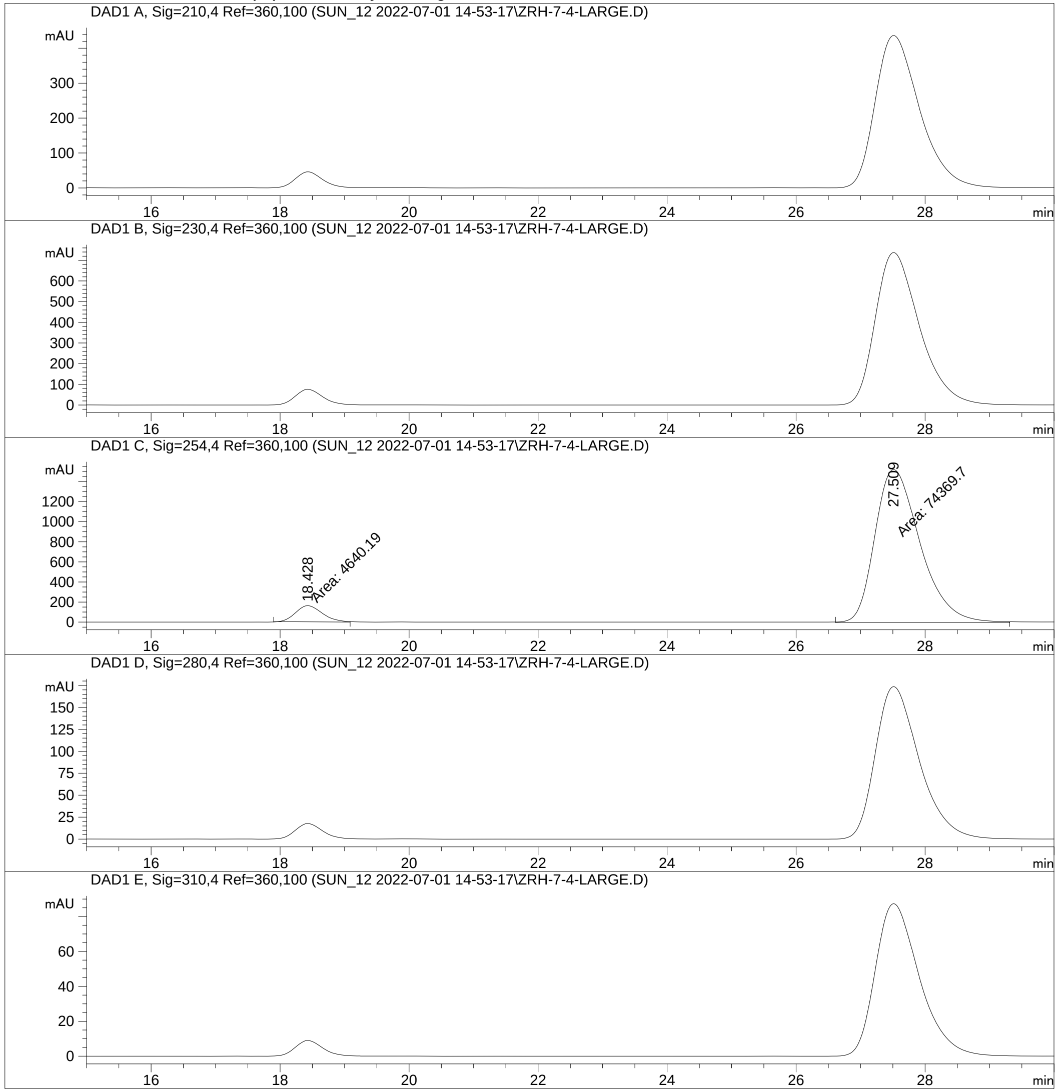

Data File C:\CHEM32\1\DATA\SUN_12 2022-07-01 14-53-17\ZRH-7-4-LARGE.D
Sample Name:

===

Area Percent Report

===

Sorted By : Signal
Multiplier : 1.0000
Dilution : 1.0000
Use Multiplier & Dilution Factor with ISTDs

Signal 1: DAD1 A, Sig=210,4 Ref=360,100

Signal 2: DAD1 B, Sig=230,4 Ref=360,100

Signal 3: DAD1 C, Sig=254,4 Ref=360,100

Peak #	RetTime [min]	Type	Width [min]	Area [mAU*s]	Height [mAU]	Area %
1	18.428	MM	0.4794	4640.19141	161.33424	5.8729
2	27.509	MM	0.8128	7.43697e4	1524.94055	94.1271

Totals : 7.90099e4 1686.27480

Signal 4: DAD1 D, Sig=280,4 Ref=360,100

Signal 5: DAD1 E, Sig=310,4 Ref=360,100

===

*** End of Report ***

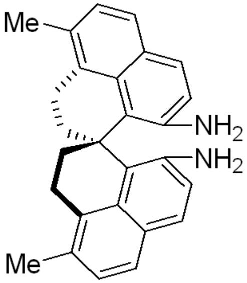

1c
(enantioenriched)

```
=====================================================================
Acq. Operator   :                               Seq. Line :   6
Acq. Instrument : Instrument 1                   Location : Vial 31
Injection Date  : 7/26/2022 2:12:21 PM                Inj :   1
                                               Inj Volume : 5.000 µl
Different Inj Volume from Sequence !     Actual Inj Volume : 12.000 µl
Acq. Method     : C:\CHEM32\1\DATA\SUN_12 2022-07-26 12-19-15\AD-30-40.M
Last changed    : 7/26/2022 1:30:23 PM
                  (modified after loading)
Analysis Method : C:\CHEM32\1\DATA\SUN_12 2022-12-29 09-07-21\2.D\DA.M (OD-05-10.M)
Last changed    : 1/12/2023 10:07:57 PM
                  (modified after loading)
Additional Info : Peak(s) manually integrated
```

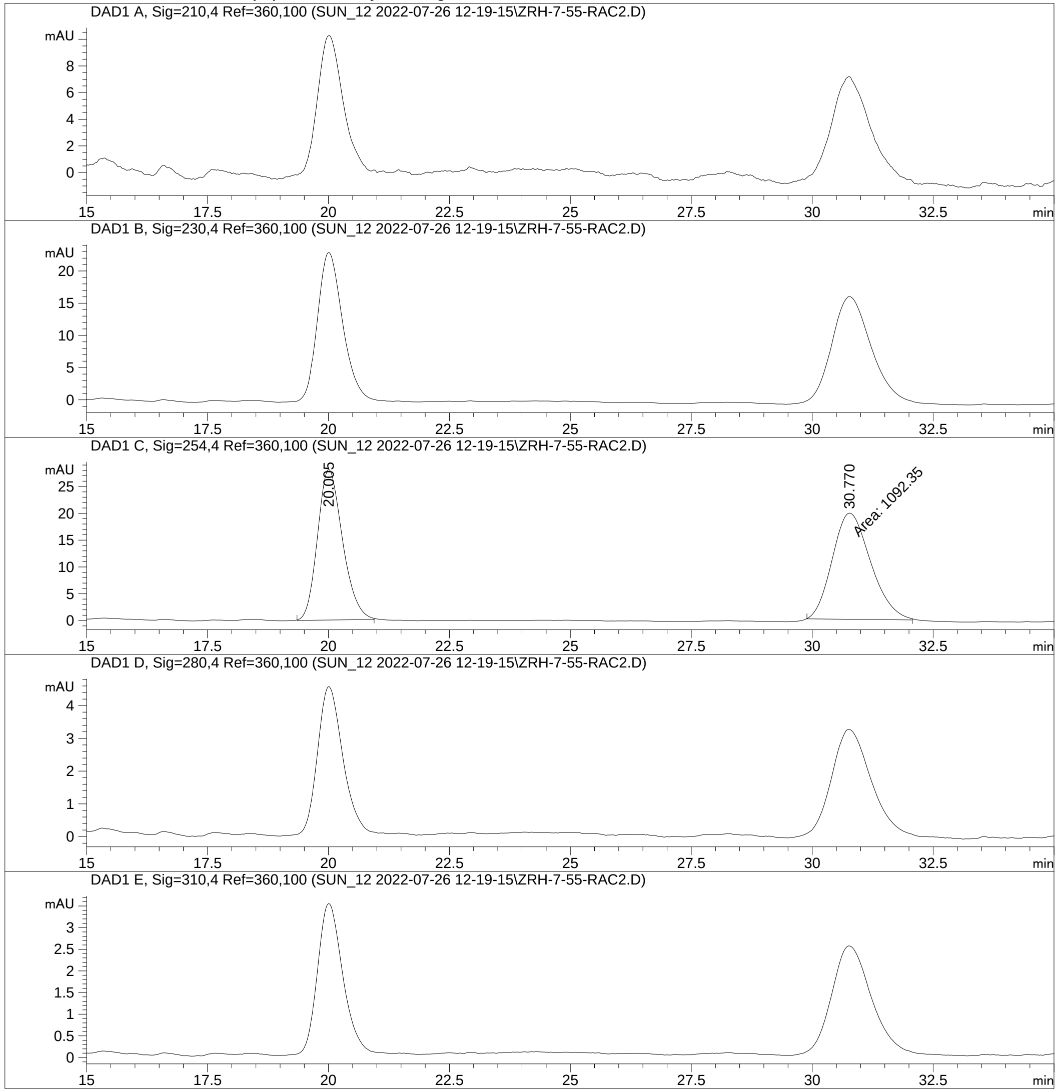


```
=====================================================================
                         Area Percent Report
=====================================================================

Sorted By             :      Signal
Multiplier            :      1.0000
Dilution              :      1.0000
Use Multiplier & Dilution Factor with ISTDs


Signal 1: DAD1 A, Sig=210,4 Ref=360,100


Signal 2: DAD1 B, Sig=230,4 Ref=360,100


Signal 3: DAD1 C, Sig=254,4 Ref=360,100

Peak RetTime Type  Width     Area      Height     Area
  #   [min]        [min]   [mAU*s]     [mAU]        %
----|-------|----|-------|----------|----------|--------|
   1  20.005 BB    0.5473 1008.57690   28.06460  48.0063
   2  30.770 MM    0.9196 1092.34875   19.79685  51.9937

Totals :                  2100.92566   47.86145



Signal 4: DAD1 D, Sig=280,4 Ref=360,100


Signal 5: DAD1 E, Sig=310,4 Ref=360,100

=====================================================================
                          *** End of Report ***
```

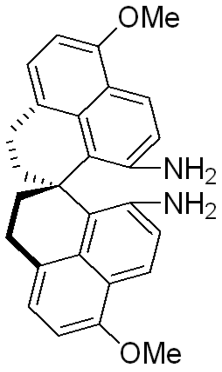

1d
(racemic)

```
=====================================================================
Acq. Operator   :                               Seq. Line :   2
Acq. Instrument : Instrument 1                   Location : Vial 31
Injection Date  : 7/27/2022 1:17:02 PM                Inj :   1
                                               Inj Volume : 5.000 µl
Acq. Method     : C:\CHEM32\1\DATA\SUN_12 2022-07-27 13-09-17\AD-30-40.M
Last changed    : 7/27/2022 1:49:30 PM
                  (modified after loading)
Analysis Method : C:\CHEM32\1\DATA\SUN_12 2022-12-29 09-07-21\2.D\DA.M (OD-05-10.M)
Last changed    : 1/12/2023 10:07:57 PM
                  (modified after loading)
Additional Info : Peak(s) manually integrated
```

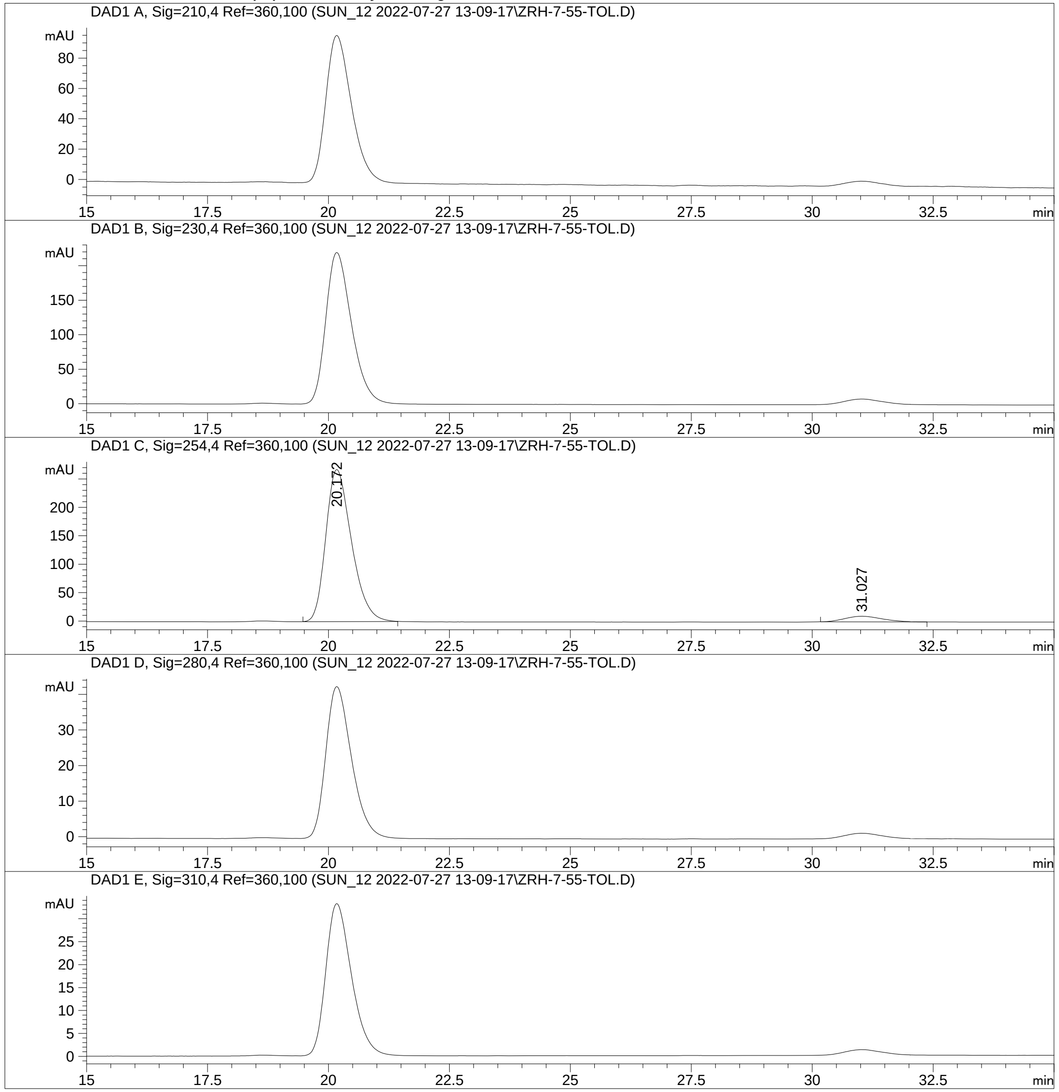

Data File C:\CHEM32\1\DATA\SUN_12 2022-07-27 13-09-17\ZRH-7-55-TOL.D
Sample Name:

```
=====================================================================
                          Area Percent Report
=====================================================================

Sorted By             :      Signal
Multiplier            :      1.0000
Dilution              :      1.0000
Use Multiplier & Dilution Factor with ISTDs


Signal 1: DAD1 A, Sig=210,4 Ref=360,100


Signal 2: DAD1 B, Sig=230,4 Ref=360,100


Signal 3: DAD1 C, Sig=254,4 Ref=360,100
```

Peak #	RetTime [min]	Type	Width [min]	Area [mAU*s]	Height [mAU]	Area %
1	20.172	BB	0.5716	9902.68457	267.66055	94.8215
2	31.027	BB	0.7947	540.82092	9.97183	5.1785

Totals : 1.04435e4 277.63238

```
Signal 4: DAD1 D, Sig=280,4 Ref=360,100


Signal 5: DAD1 E, Sig=310,4 Ref=360,100

=====================================================================
                          *** End of Report ***
```

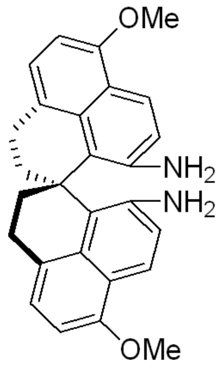

1d
(enantioenriched)

===

Acq. Operator : Seq. Line : 2
Acq. Instrument : Instrument 1 Location : Vial 31
Injection Date : 6/14/2022 7:53:50 PM Inj : 1
Inj Volume : 5.000 µl
Different Inj Volume from Sequence ! Actual Inj Volume : 9.000 µl
Acq. Method : C:\CHEM32\1\DATA\SUN_12 2022-06-14 19-46-03\AD-30-40.M
Last changed : 6/14/2022 8:06:21 PM
(modified after loading)
Analysis Method : C:\CHEM32\1\DATA\SUN_12 2022-12-29 09-07-21\2.D\DA.M (OD-05-10.M)
Last changed : 1/12/2023 9:07:28 PM
(modified after loading)
Additional Info : Peak(s) manually integrated

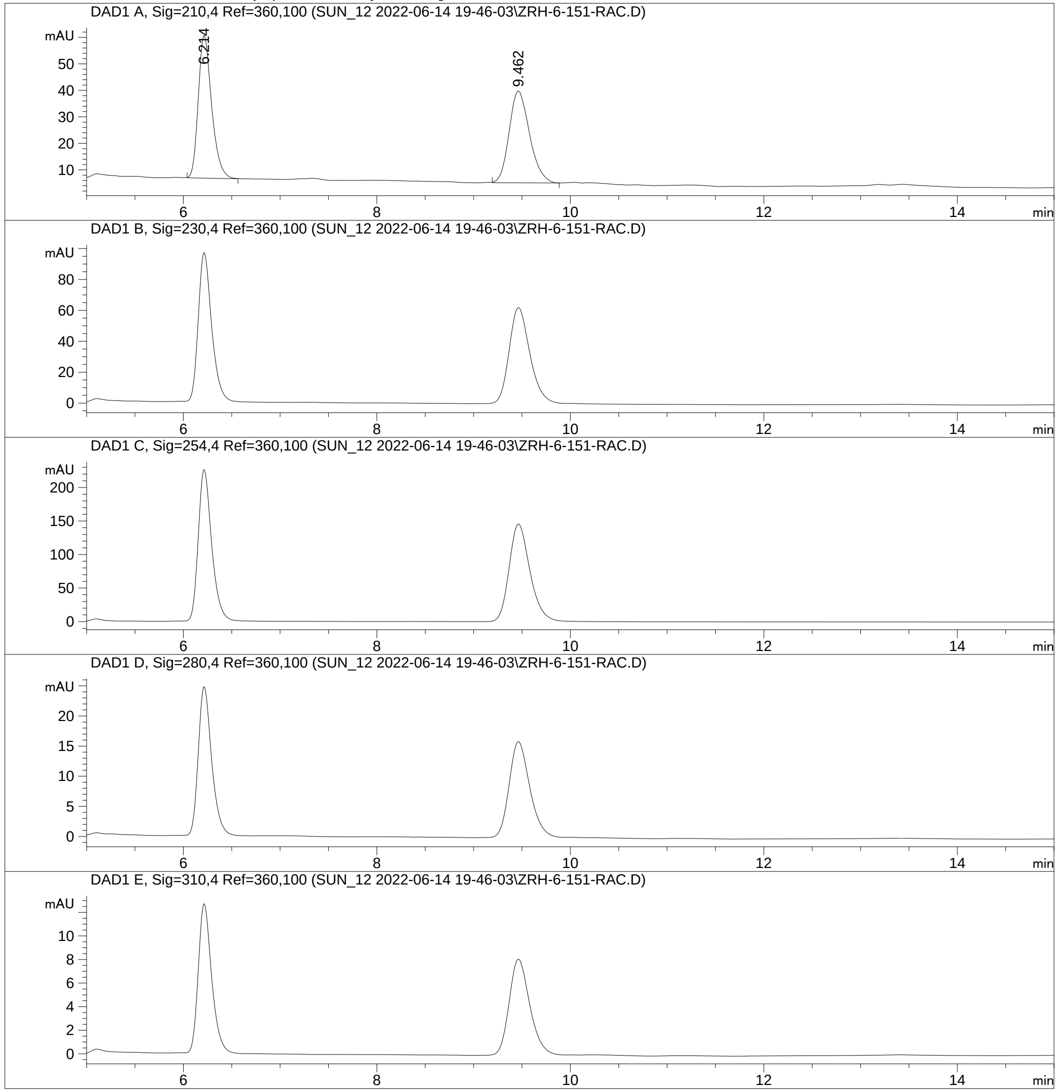

===

Area Percent Report

===

Sorted By : Signal
Multiplier : 1.0000
Dilution : 1.0000
Use Multiplier & Dilution Factor with ISTDs

Signal 1: DAD1 A, Sig=210,4 Ref=360,100

Peak #	RetTime [min]	Type	Width [min]	Area [mAU*s]	Height [mAU]	Area %
1	6.214	BB	0.1458	516.07013	53.87353	50.3769
2	9.462	BB	0.2291	508.34781	34.65858	49.6231

Totals : 1024.41794 88.53211

Signal 2: DAD1 B, Sig=230,4 Ref=360,100

Signal 3: DAD1 C, Sig=254,4 Ref=360,100

Signal 4: DAD1 D, Sig=280,4 Ref=360,100

Signal 5: DAD1 E, Sig=310,4 Ref=360,100

===

*** End of Report ***

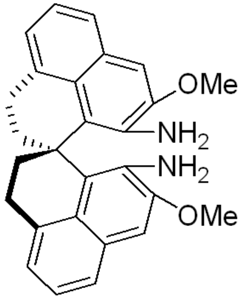

1e
(racemic)

===

Acq. Operator : Seq. Line : 2
Acq. Instrument : Instrument 1 Location : Vial 32
Injection Date : 6/14/2022 9:51:47 PM Inj : 1
Inj Volume : 5.000 µl
Acq. Method : C:\CHEM32\1\DATA\SUN_12 2022-06-14 21-43-58\AD-30-20.M
Last changed : 6/14/2022 9:50:54 PM
(modified after loading)
Analysis Method : C:\CHEM32\1\DATA\SUN_12 2022-12-29 09-07-21\2.D\DA.M (OD-05-10.M)
Last changed : 1/12/2023 9:07:28 PM
(modified after loading)
Additional Info : Peak(s) manually integrated

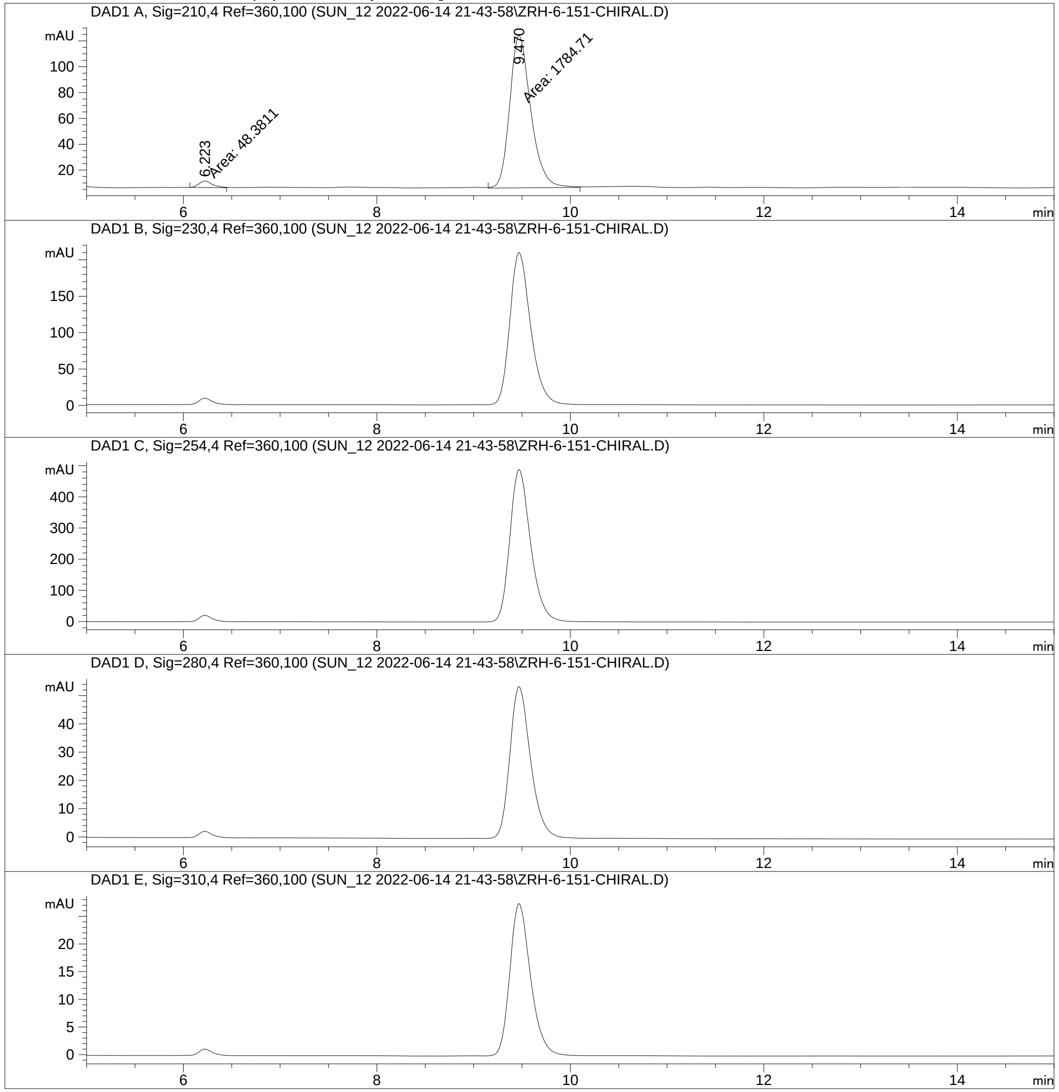

Data File C:\CHEM32\1\DATA\SUN_12 2022-06-14 21-43-58\ZRH-6-151-CHIRAL.D
Sample Name:

===

Area Percent Report

===

Sorted By : Signal
Multiplier : 1.0000
Dilution : 1.0000
Use Multiplier & Dilution Factor with ISTDs

Signal 1: DAD1 A, Sig=210,4 Ref=360,100

Peak #	RetTime [min]	Type	Width [min]	Area [mAU*s]	Height [mAU]	Area %
1	6.223	MM	0.1596	48.38110	5.05259	2.6393
2	9.470	MM	0.2520	1784.70740	118.02903	97.3607

Totals : 1833.08850 123.08162

Signal 2: DAD1 B, Sig=230,4 Ref=360,100

Signal 3: DAD1 C, Sig=254,4 Ref=360,100

Signal 4: DAD1 D, Sig=280,4 Ref=360,100

Signal 5: DAD1 E, Sig=310,4 Ref=360,100

===

*** End of Report ***

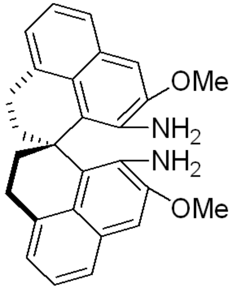

1e
(enantioenriched)

```
=====================================================================
Acq. Operator   :                               Seq. Line :  38
Acq. Instrument : Instrument 1                   Location : Vial 31
Injection Date  : 6/23/2022 8:25:51 PM                Inj :   1
                                               Inj Volume : 5.000 µl
Different Inj Volume from Sequence !     Actual Inj Volume : 9.000 µl
Acq. Method     : C:\CHEM32\1\DATA\SUN_12 2022-06-23 09-42-20\AD-30-60.M
Last changed    : 6/23/2022 8:59:30 PM
                  (modified after loading)
Analysis Method : C:\CHEM32\1\DATA\SUN_12 2022-12-29 09-07-21\2.D\DA.M (OD-05-10.M)
Last changed    : 1/12/2023 9:19:45 PM
                  (modified after loading)
Additional Info : Peak(s) manually integrated
```

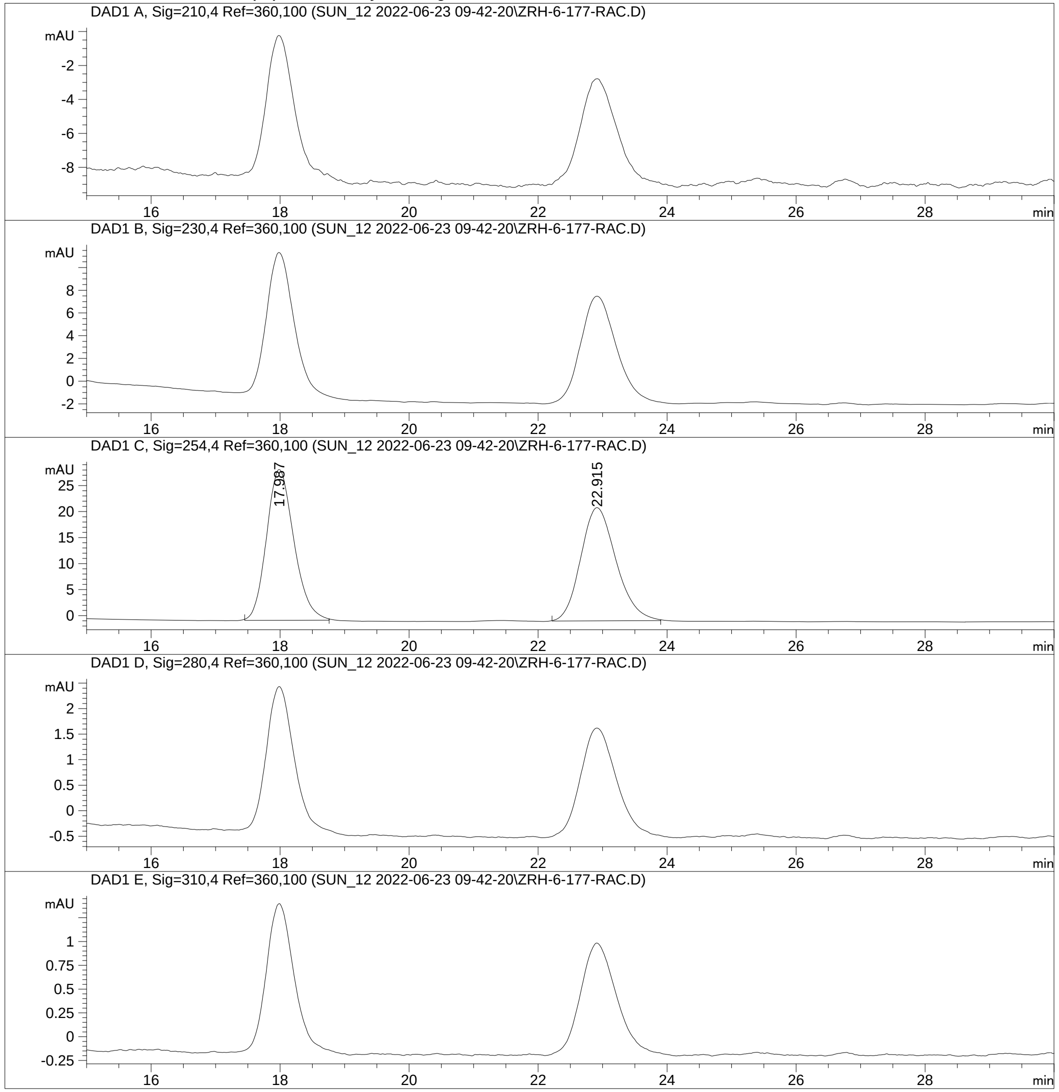

===
Area Percent Report
===

Sorted By : Signal
Multiplier : 1.0000
Dilution : 1.0000
Use Multiplier & Dilution Factor with ISTDs

Signal 1: DAD1 A, Sig=210,4 Ref=360,100

Signal 2: DAD1 B, Sig=230,4 Ref=360,100

Signal 3: DAD1 C, Sig=254,4 Ref=360,100

Peak #	RetTime [min]	Type	Width [min]	Area [mAU*s]	Height [mAU]	Area %
1	17.987	BB	0.4540	854.47266	28.97742	50.7253
2	22.915	BB	0.5909	830.03650	21.76639	49.2747

Totals : 1684.50916 50.74381

Signal 4: DAD1 D, Sig=280,4 Ref=360,100

Signal 5: DAD1 E, Sig=310,4 Ref=360,100

===
*** End of Report ***

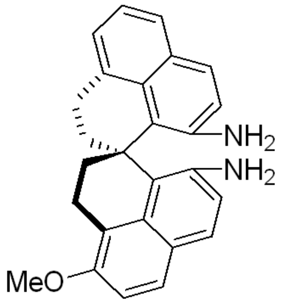

1f
(racemic)

Data File C:\CHEM32\1\DATA\SUN_12 2022-06-23 09-42-20\ZRH-6-177-CHIRAL.D
Sample Name:

===

Acq. Operator : Seq. Line : 39
Acq. Instrument : Instrument 1 Location : Vial 32
Injection Date : 6/23/2022 9:00:34 PM Inj : 1
Inj Volume : 5.000 µl
Different Inj Volume from Sequence ! Actual Inj Volume : 9.000 µl
Acq. Method : C:\CHEM32\1\DATA\SUN_12 2022-06-23 09-42-20\AD-30-60.M
Last changed : 6/23/2022 8:59:30 PM
(modified after loading)
Analysis Method : C:\CHEM32\1\DATA\SUN_12 2022-12-29 09-07-21\2.D\DA.M (OD-05-10.M)
Last changed : 1/12/2023 9:22:42 PM
(modified after loading)
Additional Info : Peak(s) manually integrated

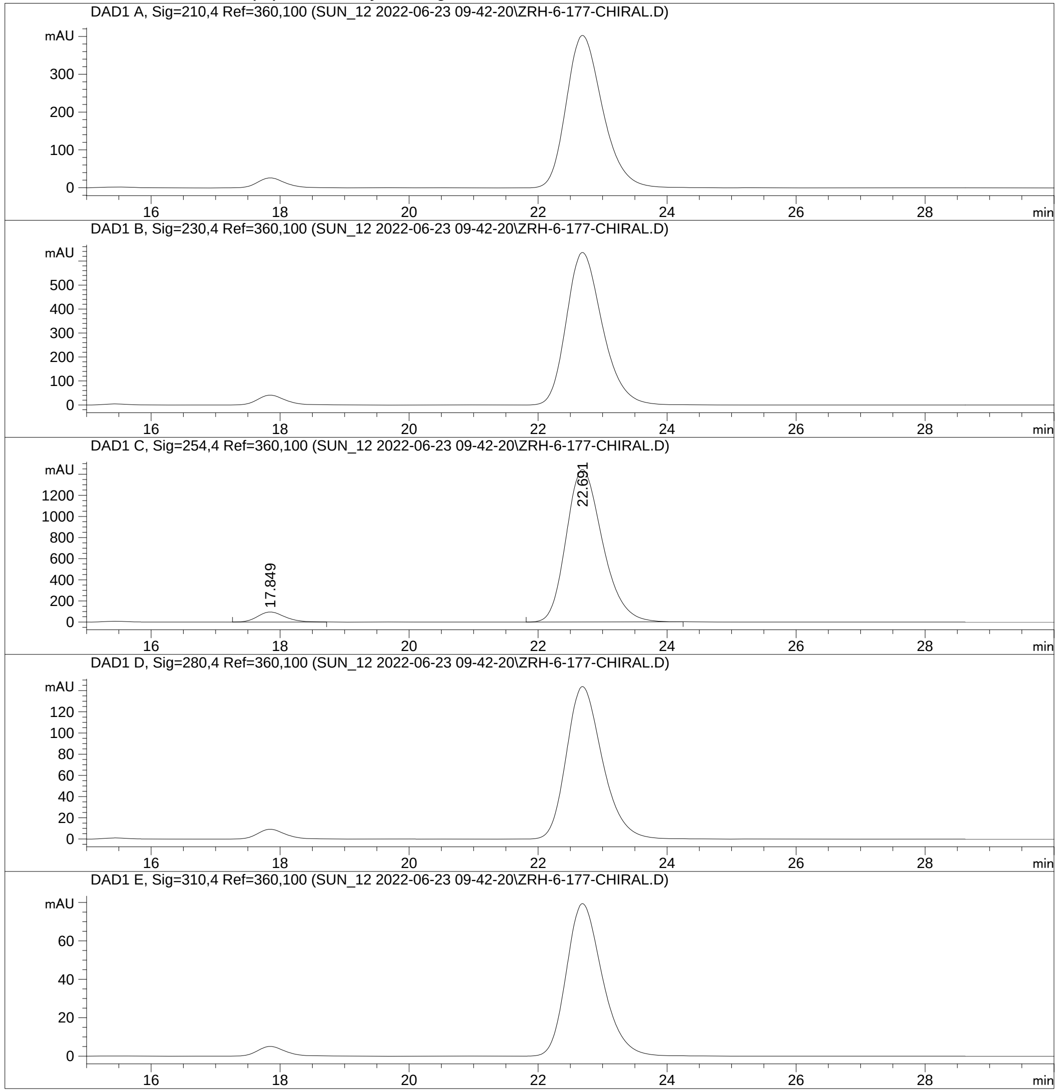

Data File C:\CHEM32\1\DATA\SUN_12 2022-06-23 09-42-20\ZRH-6-177-CHIRAL.D
Sample Name:

===
Area Percent Report
===

Sorted By : Signal
Multiplier : 1.0000
Dilution : 1.0000
Use Multiplier & Dilution Factor with ISTDs

Signal 1: DAD1 A, Sig=210,4 Ref=360,100

Signal 2: DAD1 B, Sig=230,4 Ref=360,100

Signal 3: DAD1 C, Sig=254,4 Ref=360,100

Peak #	RetTime [min]	Type	Width [min]	Area [mAU*s]	Height [mAU]	Area %
1	17.849	BB	0.4503	2805.51538	95.61735	4.7296
2	22.691	BB	0.6027	5.65127e4	1443.51599	95.2704

Totals : 5.93182e4 1539.13334

Signal 4: DAD1 D, Sig=280,4 Ref=360,100

Signal 5: DAD1 E, Sig=310,4 Ref=360,100

===
*** End of Report ***

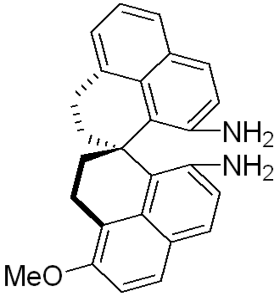

1f
(enantioenriched)

===

Acq. Operator : SYSTEM Seq. Line : 11
Sample Operator : SYSTEM
Acq. Instrument : HPLC Location : P2-C-01
Injection Date : 30/12/2022 1:46:33 pm Inj : 1
Inj Volume : 2.000 µl
Different Inj Volume from Sample Entry! Actual Inj Volume : 10.000 µl
Acq. Method : C:\Users\Public\Documents\ChemStation\1\Data\SUN\SUN 2022-12-30 09-51-19\AD3-20-40.M
Last changed : 30/12/2022 1:12:17 pm by SYSTEM
Analysis Method : C:\Users\Public\Documents\ChemStation\1\Data\SUN\SUN 2022-12-30 09-51-19\AD3-20-40.M (Sequence Method)
Last changed : 12/1/2023 10:46:29 pm by SYSTEM
(modified after loading)
Additional Info : Peak(s) manually integrated

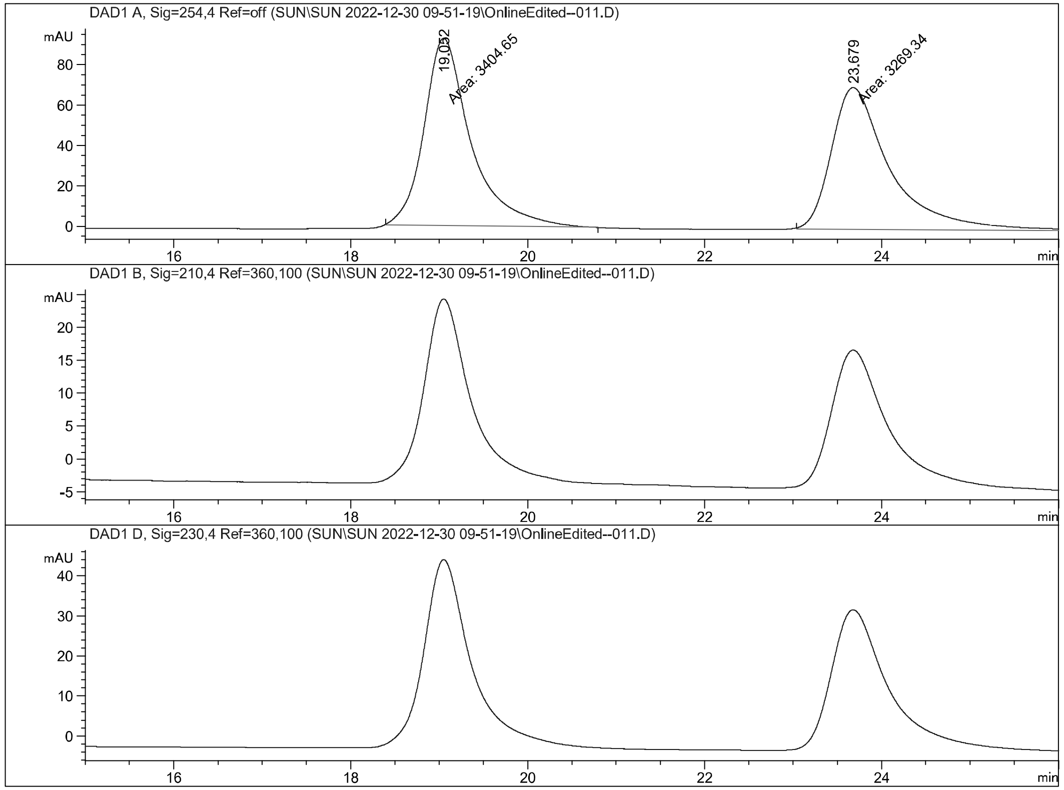

Data File C:\Users\P...\ChemStation\1\Data\SUN\SUN 2022-12-30 09-51-19\OnlineEdited--011.D
Sample Name: zrh-8-101-1-rac

===

Area Percent Report

===

Sorted By : Signal
Multiplier : 1.0000
Dilution : 1.0000
Use Multiplier & Dilution Factor with ISTDs

Signal 1: DAD1 A, Sig=254,4 Ref=off

Peak #	RetTime [min]	Type	Width [min]	Area [mAU*s]	Height [mAU]	Area %
1	19.052	MM	0.6122	3404.65479	92.69099	51.0137
2	23.679	MM	0.7747	3269.34399	70.33273	48.9863

Totals : 6673.99878 163.02371

Signal 2: DAD1 B, Sig=210,4 Ref=360,100

Signal 3: DAD1 D, Sig=230,4 Ref=360,100

===

*** End of Report ***

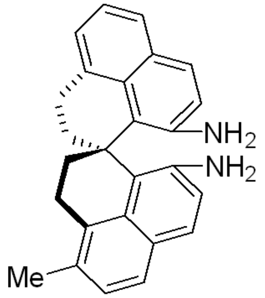

1g
(racemic)

```
=====================================================================
Acq. Operator   : SYSTEM                        Seq. Line :    7
Sample Operator : SYSTEM
Acq. Instrument : HPLC                           Location :   P2-C-02
Injection Date  : 29/12/2022 1:20:44 pm               Inj :    1
                                               Inj Volume : 2.000 µl
Different Inj Volume from Sample Entry!  Actual Inj Volume : 10.000 µl
Acq. Method     : C:\Users\Public\Documents\ChemStation\1\Data\SUN\SUN 2022-12-29 10-12-37
                  \AD3-20-60.M
Last changed    : 29/12/2022 1:50:16 pm by SYSTEM
                  (modified after loading)
Analysis Method : C:\Users\Public\Documents\ChemStation\1\Data\SUN\SUN 2022-12-29 10-12-37
                  \AD3-20-60.M (Sequence Method)
Last changed    : 12/1/2023 10:43:47 pm by SYSTEM
                  (modified after loading)
Additional Info : Peak(s) manually integrated
```

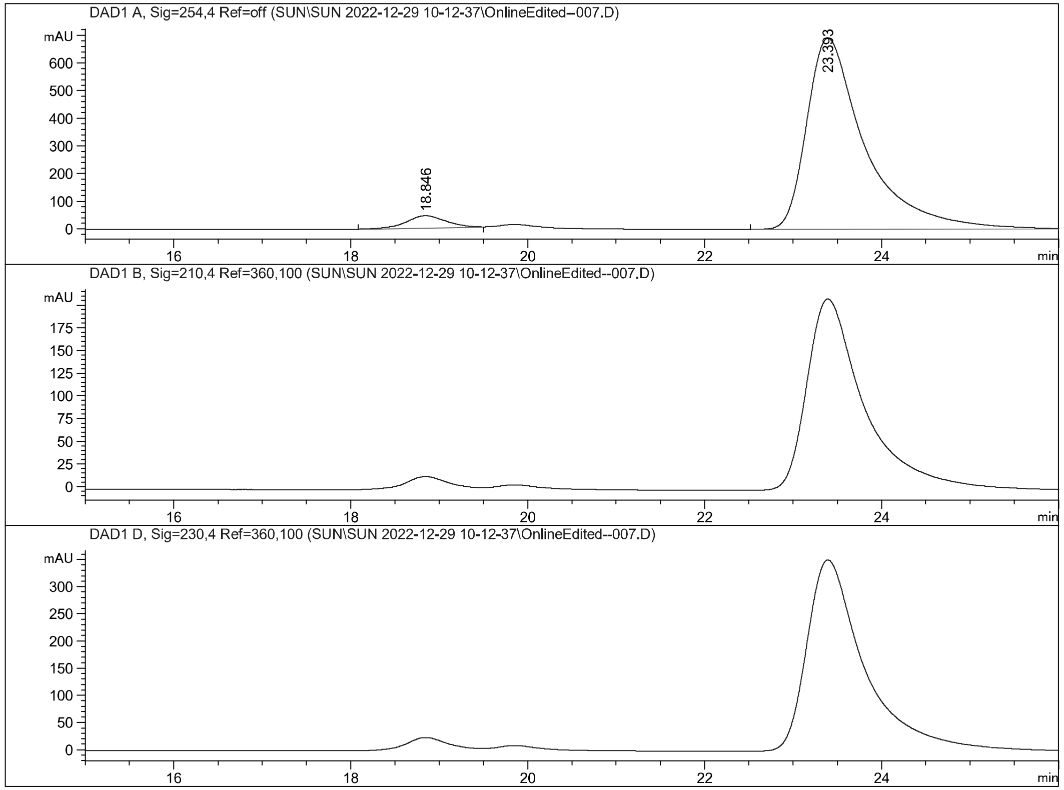

Data File C:\Users\P...\ChemStation\1\Data\SUN\SUN 2022-12-29 10-12-37\OnlineEdited--007.D
Sample Name: zrh-8-101-1

```
=====================================================================
                         Area Percent Report
=====================================================================

Sorted By             :      Signal
Multiplier            :      1.0000
Dilution              :      1.0000
Use Multiplier & Dilution Factor with ISTDs


Signal 1: DAD1 A, Sig=254,4 Ref=off

Peak RetTime Type  Width     Area      Height     Area
  #   [min]        [min]   [mAU*s]     [mAU]        %
----|-------|----|-------|----------|----------|--------|
   1  18.846 BB    0.4501 1366.28638   44.33143   4.1509
   2  23.393 BB    0.6626 3.15494e4   691.10938  95.8491

Totals :                  3.29157e4   735.44081



Signal 2: DAD1 B, Sig=210,4 Ref=360,100


Signal 3: DAD1 D, Sig=230,4 Ref=360,100

=====================================================================
                          *** End of Report ***
```

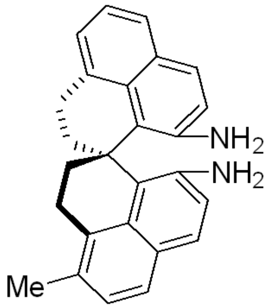

1g
(enantioenriched)

```
=====================================================================
Acq. Operator   :                                   Seq. Line :  25
Acq. Instrument : Instrument 1                       Location : Vial 31
Injection Date  : 7/1/2022 11:55:12 PM                    Inj :   1
                                                   Inj Volume : 5.000 µl
Different Inj Volume from Sequence !     Actual Inj Volume : 10.000 µl
Acq. Method     : C:\CHEM32\1\DATA\SUN_12 2022-07-01 14-53-17\AD-20-60.M
Last changed    : 7/1/2022 11:54:20 PM
                  (modified after loading)
Analysis Method : C:\CHEM32\1\DATA\SUN_12 2023-05-12 21-33-41\OD-10-30.M
Last changed    : 6/4/2023 6:41:11 PM
                  (modified after loading)
Additional Info : Peak(s) manually integrated
```

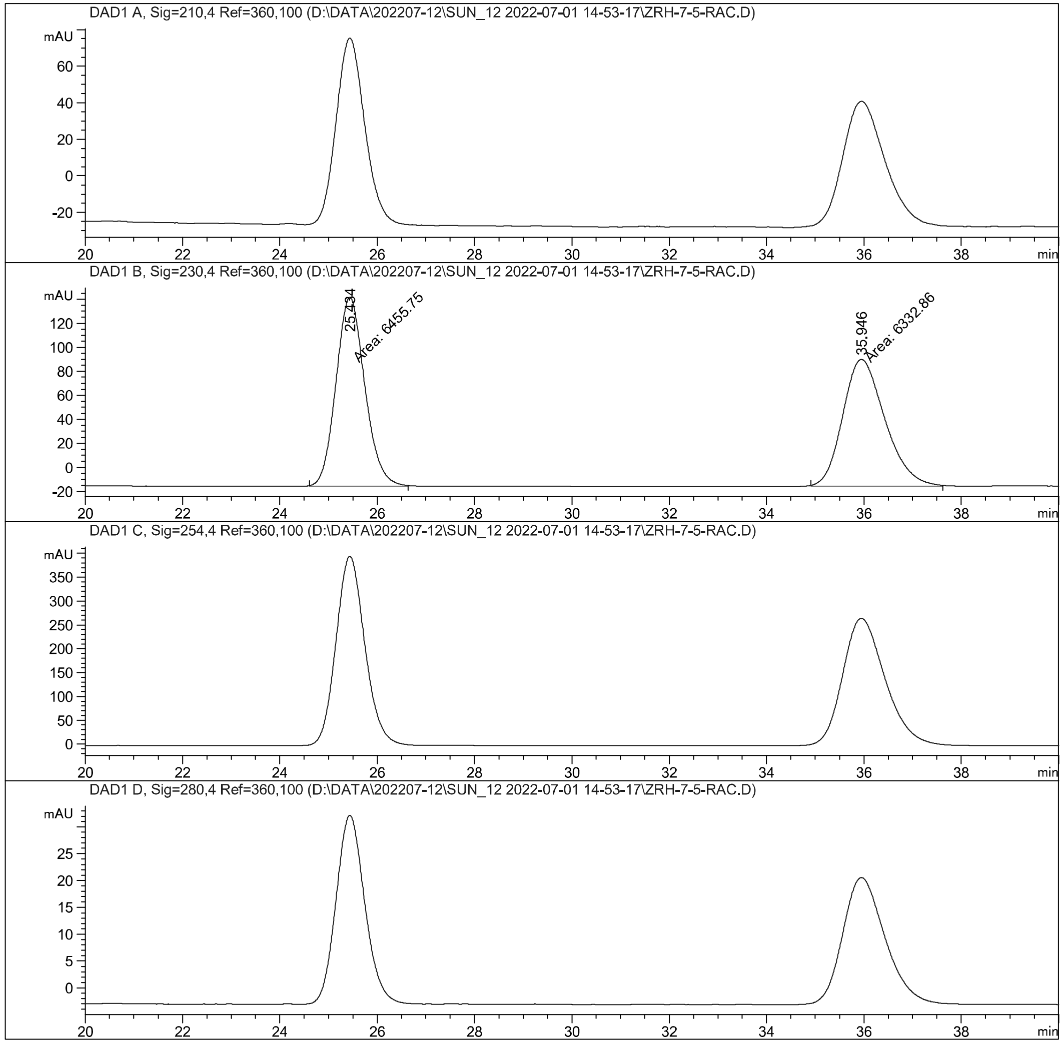

Data File D:\DATA\202207-12\SUN_12 2022-07-01 14-53-17\ZRH-7-5-RAC.D
Sample Name:

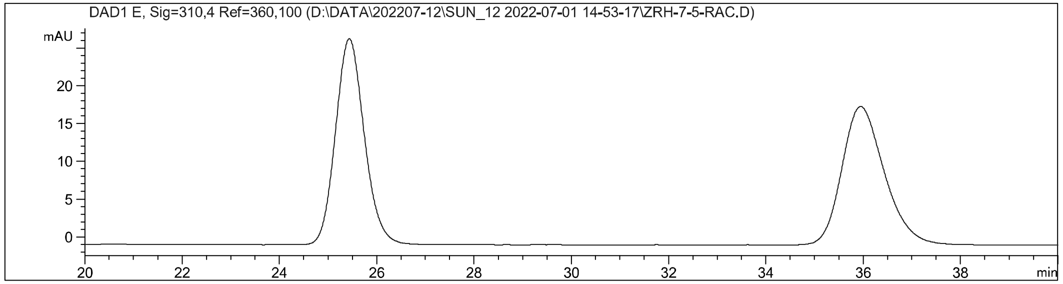


```
=====================================================================
                         Area Percent Report
=====================================================================

Sorted By             :      Signal
Multiplier            :      1.0000
Dilution              :      1.0000
Use Multiplier & Dilution Factor with ISTDs


Signal 1: DAD1 A, Sig=210,4 Ref=360,100


Signal 2: DAD1 B, Sig=230,4 Ref=360,100

Peak RetTime Type  Width     Area      Height     Area
  #   [min]        [min]   [mAU*s]     [mAU]        %
----|-------|----|-------|----------|----------|--------|
   1  25.434 MM    0.6840 6455.74902  157.30208  50.4804
   2  35.946 MM    1.0014 6332.86475  105.40359  49.5196

Totals :                  1.27886e4   262.70567



Signal 3: DAD1 C, Sig=254,4 Ref=360,100


Signal 4: DAD1 D, Sig=280,4 Ref=360,100


Signal 5: DAD1 E, Sig=310,4 Ref=360,100

=====================================================================
                          *** End of Report ***
```

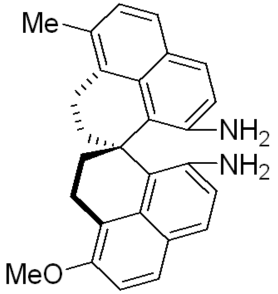

1h
(racemic)

===

Acq. Operator   :   Seq. Line : 27
Acq. Instrument : Instrument 1   Location : Vial 32
Injection Date : 7/2/2022 1:27:26 AM   Inj : 1
                                                  Inj Volume : 5.000 µl
Different Inj Volume from Sequence !   Actual Inj Volume : 10.000 µl
Acq. Method   : C:\CHEM32\1\DATA\SUN_12 2022-07-01 14-53-17\AD-20-60.M
Last changed   : 5/8/2013 10:15:30 PM
Analysis Method : C:\CHEM32\1\DATA\SUN_12 2023-05-12 21-33-41\OD-10-30.M
Last changed   : 6/4/2023 6:41:11 PM
                  (modified after loading)
Additional Info : Peak(s) manually integrated

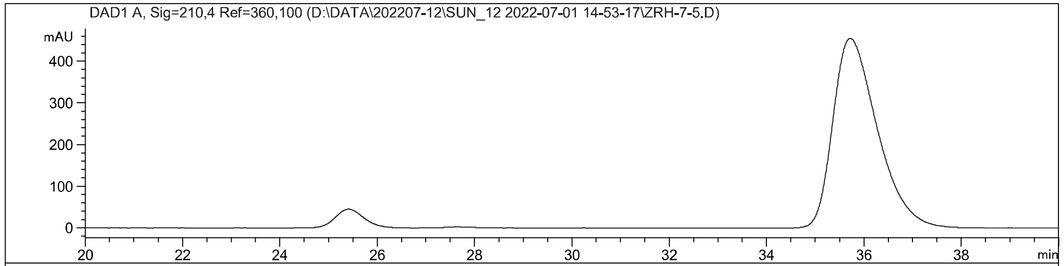

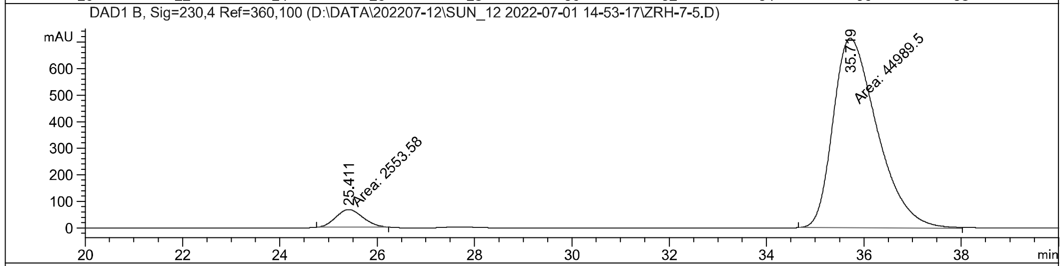

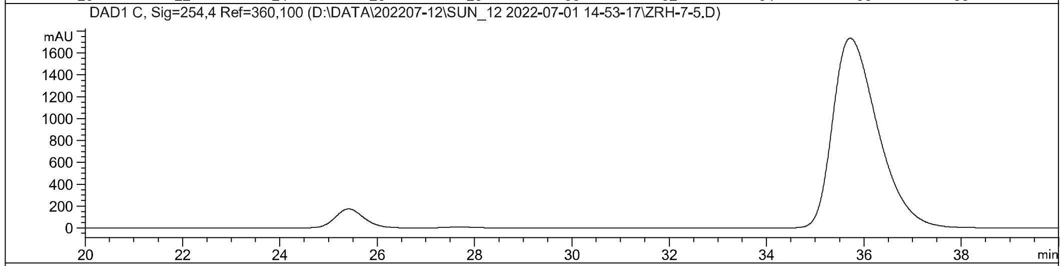

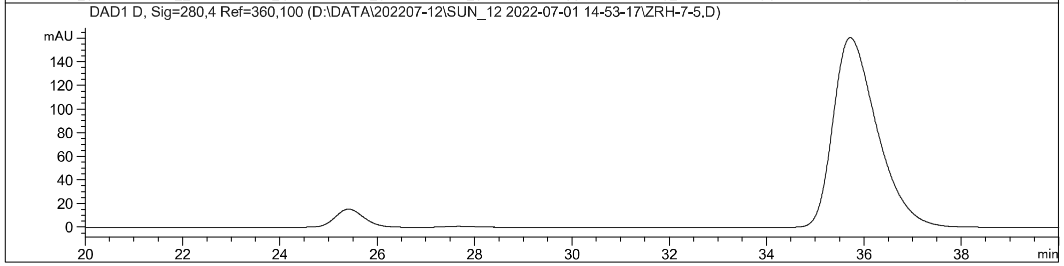

Data File D:\DATA\202207-12\SUN_12 2022-07-01 14-53-17\ZRH-7-5.D
Sample Name:

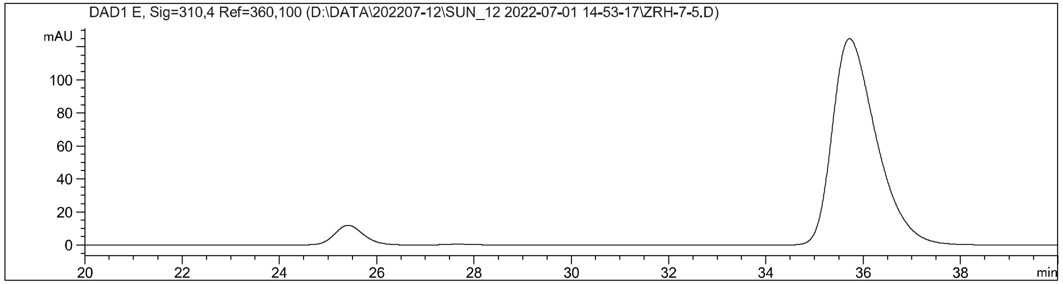

===

Area Percent Report

===

Sorted By   :   Signal
Multiplier   :   1.0000
Dilution   :   1.0000
Use Multiplier & Dilution Factor with ISTDs

Signal 1: DAD1 A, Sig=210,4 Ref=360,100

Signal 2: DAD1 B, Sig=230,4 Ref=360,100

Peak #	RetTime [min]	Type	Width [min]	Area [mAU*s]	Height [mAU]	Area %
1	25.411	MM	0.6421	2553.58350	66.28671	5.3711
2	35.719	MM	1.0502	4.49895e4	713.95972	94.6289

Totals :   4.75431e4   780.24643

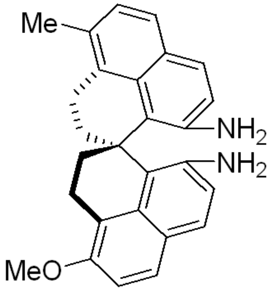

1h
(enantioenriched)

Signal 3: DAD1 C, Sig=254,4 Ref=360,100

Signal 4: DAD1 D, Sig=280,4 Ref=360,100

Signal 5: DAD1 E, Sig=310,4 Ref=360,100

===

*** End of Report ***

```
Data File C:\CHEM32\1\DATA\SUN_12 2022-06-23 09-42-20\ZRH-6-175-RAC-8020.D
Sample Name:

    =====================================================================
    Acq. Operator   :                                 Seq. Line :   2
    Acq. Instrument : Instrument 1                     Location : Vial 31
    Injection Date  : 6/23/2022 9:47:11 AM                  Inj :   1
                                                     Inj Volume : 5.000 µl
    Different Inj Volume from Sequence !     Actual Inj Volume : 9.000 µl
    Acq. Method     : C:\CHEM32\1\DATA\SUN_12 2022-06-23 09-42-20\AD-20-60.M
    Last changed    : 6/23/2022 9:46:26 AM
                      (modified after loading)
    Analysis Method : C:\CHEM32\1\DATA\SUN_12 2022-12-29 09-07-21\2.D\DA.M (OD-05-10.M)
    Last changed    : 1/12/2023 9:27:46 PM
                      (modified after loading)
    Additional Info : Peak(s) manually integrated
```

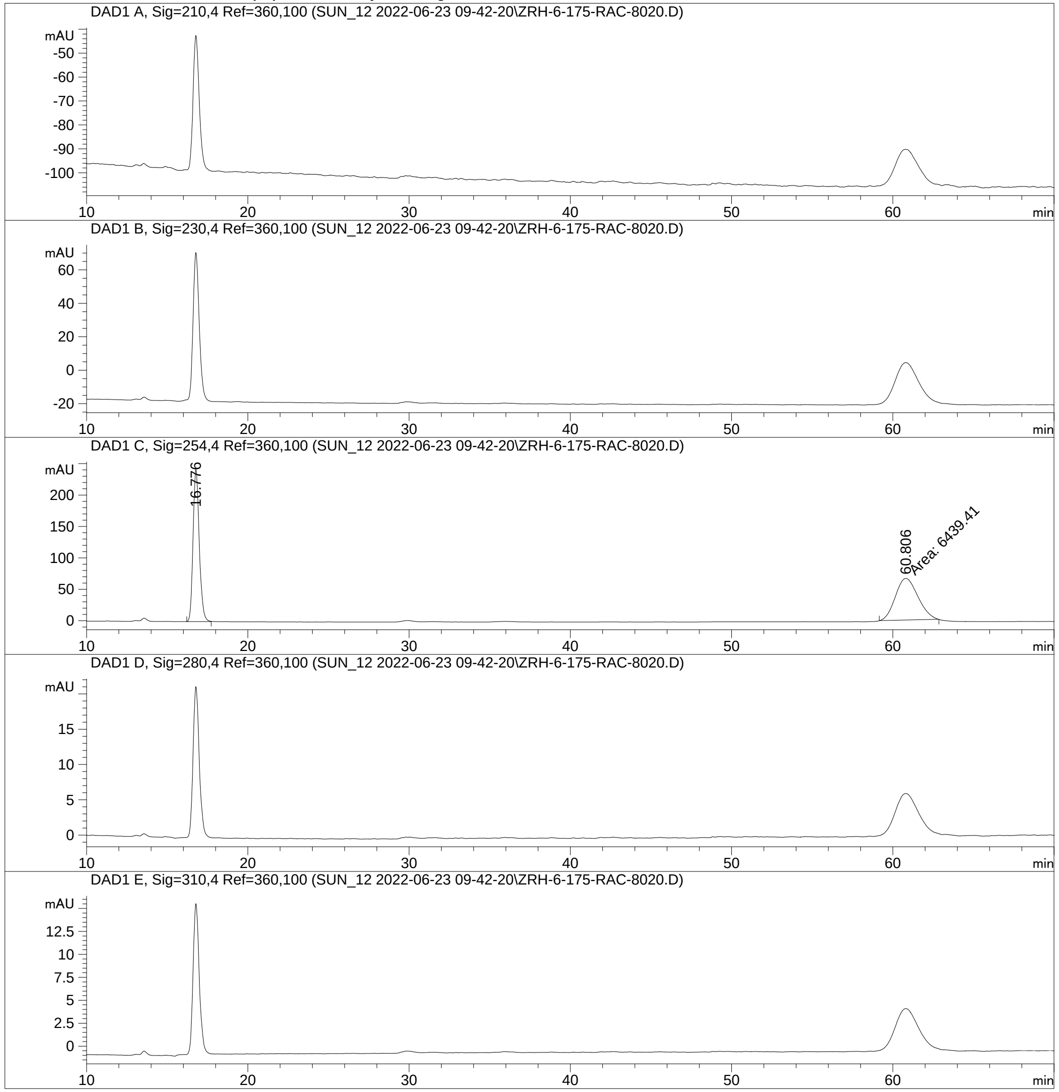

Data File C:\CHEM32\1\DATA\SUN_12 2022-06-23 09-42-20\ZRH-6-175-RAC-8020.D
Sample Name:


```
    =====================================================================
                             Area Percent Report
    =====================================================================

    Sorted By             :      Signal
    Multiplier            :      1.0000
    Dilution              :      1.0000
    Use Multiplier & Dilution Factor with ISTDs


    Signal 1: DAD1 A, Sig=210,4 Ref=360,100


    Signal 2: DAD1 B, Sig=230,4 Ref=360,100


    Signal 3: DAD1 C, Sig=254,4 Ref=360,100
```

Peak #	RetTime [min]	Type	Width [min]	Area [mAU*s]	Height [mAU]	Area %
1	16.776	BB	0.4089	6448.62061	241.99883	50.0357
2	60.806	MM	1.6199	6439.40625	66.25186	49.9643

```
    Totals :                  1.28880e4   308.25069



    Signal 4: DAD1 D, Sig=280,4 Ref=360,100


    Signal 5: DAD1 E, Sig=310,4 Ref=360,100

    =====================================================================
                             *** End of Report ***
```

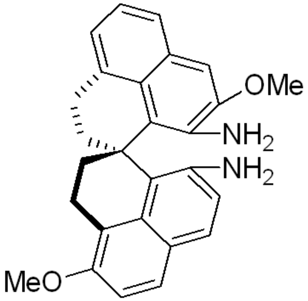

1i
(racemic)

Data File C:\CHEM32\1\DATA\SUN_12 2022-06-23 09-42-20\ZRH-6-175-L.D
Sample Name:

```
=====================================================================
Acq. Operator   :                               Seq. Line :  51
Acq. Instrument : Instrument 1                   Location : Vial 33
Injection Date  : 6/24/2022 12:26:50 AM               Inj :   1
                                              Inj Volume : 5.000 µl
Acq. Method     : C:\CHEM32\1\DATA\SUN_12 2022-06-23 09-42-20\AD-20-60.M
Last changed    : 5/8/2013 10:15:30 PM
Analysis Method : C:\CHEM32\1\DATA\SUN_12 2022-12-29 09-07-21\2.D\DA.M (OD-05-10.M)
Last changed    : 1/12/2023 9:27:46 PM
                  (modified after loading)
Additional Info : Peak(s) manually integrated
```

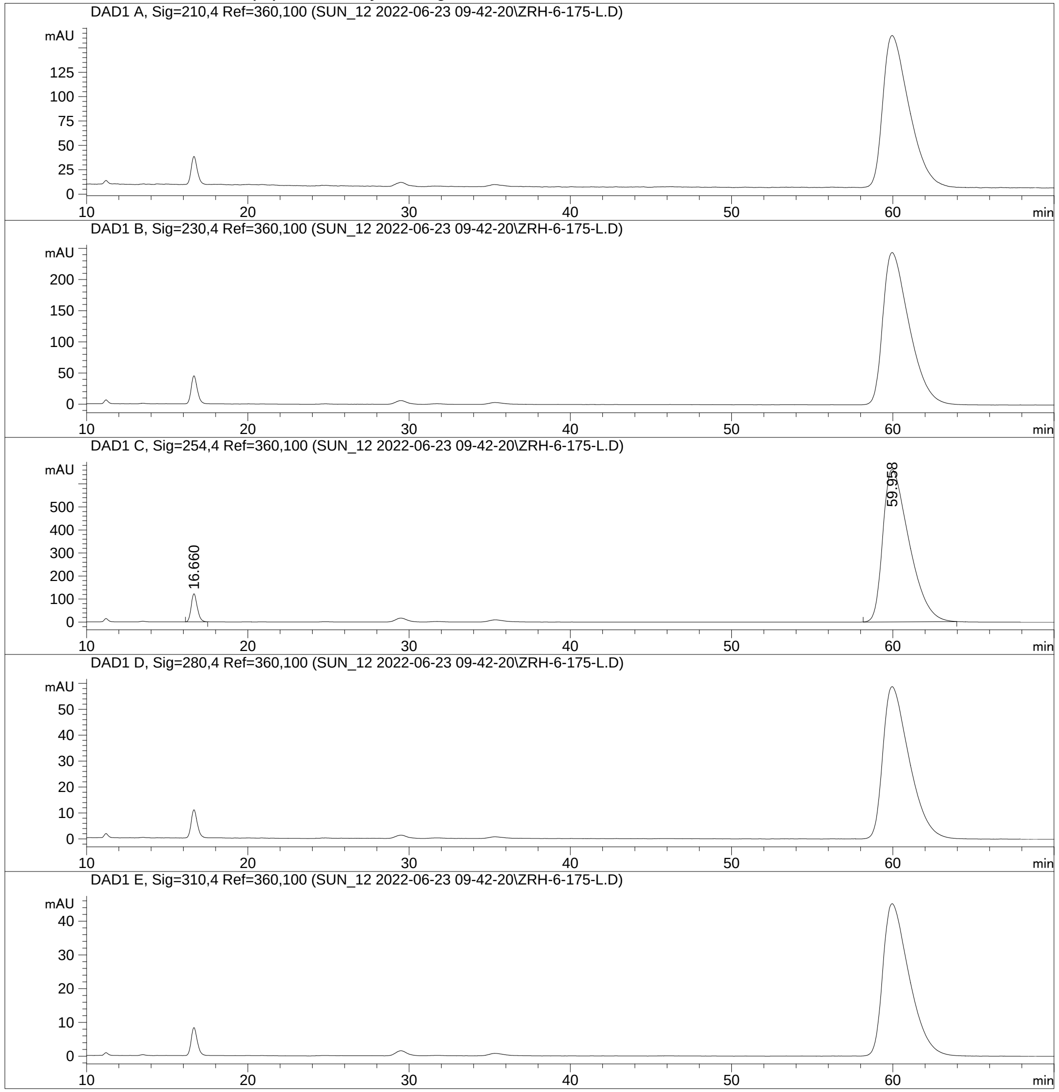

Data File C:\CHEM32\1\DATA\SUN_12 2022-06-23 09-42-20\ZRH-6-175-L.D
Sample Name:

```
=====================================================================
                         Area Percent Report
=====================================================================

Sorted By             :      Signal
Multiplier            :      1.0000
Dilution              :      1.0000
Use Multiplier & Dilution Factor with ISTDs


Signal 1: DAD1 A, Sig=210,4 Ref=360,100


Signal 2: DAD1 B, Sig=230,4 Ref=360,100


Signal 3: DAD1 C, Sig=254,4 Ref=360,100

Peak RetTime Type  Width     Area      Height     Area
  #   [min]        [min]   [mAU*s]     [mAU]        %
----|-------|----|-------|----------|----------|--------|
   1  16.660 BB    0.4068 3228.03809  121.96296   4.1455
   2  59.958 BB    1.6864 7.46406e4   661.48450  95.8545

Totals :                  7.78687e4   783.44746



Signal 4: DAD1 D, Sig=280,4 Ref=360,100


Signal 5: DAD1 E, Sig=310,4 Ref=360,100

=====================================================================
                          *** End of Report ***
```

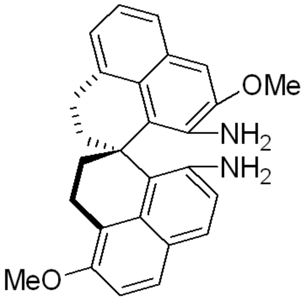

1i
(enantioenriched)

```
=====================================================================
Acq. Operator   :                               Seq. Line :   2
Acq. Instrument : Instrument 1                   Location : Vial 31
Injection Date  : 7/28/2022 4:45:04 PM                Inj :   1
                                               Inj Volume : 5.000 µl
Different Inj Volume from Sequence !     Actual Inj Volume : 6.000 µl
Acq. Method     : C:\CHEM32\1\DATA\SUN_12 2022-07-28 16-35-18\AD-30-60.M
Last changed    : 5/7/2016 9:30:19 AM
Analysis Method : C:\CHEM32\1\DATA\SUN_12 2022-12-29 09-07-21\2.D\DA.M (OD-05-10.M)
Last changed    : 1/12/2023 10:01:26 PM
                  (modified after loading)
Additional Info : Peak(s) manually integrated
```

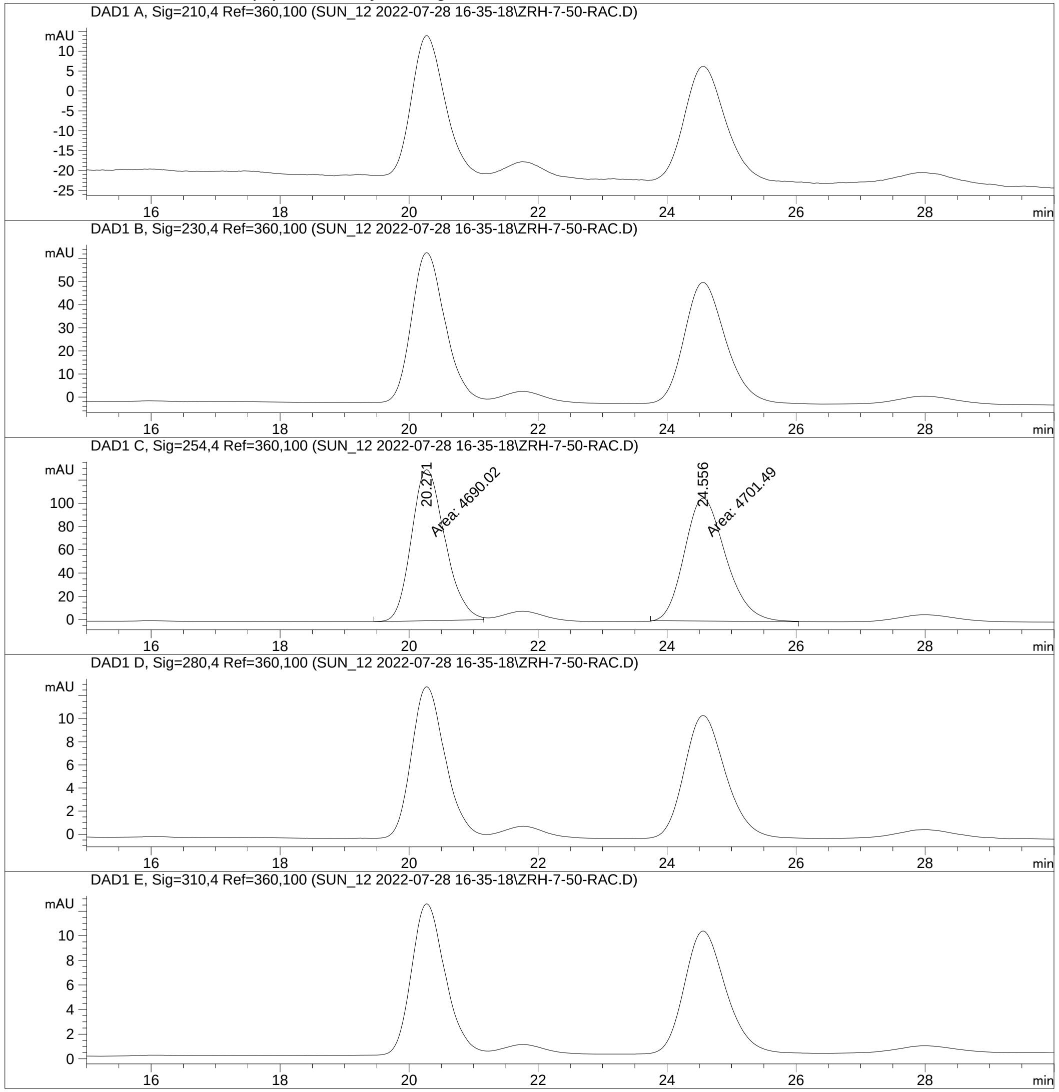


```
=====================================================================
                         Area Percent Report
=====================================================================

Sorted By             :      Signal
Multiplier            :      1.0000
Dilution              :      1.0000
Use Multiplier & Dilution Factor with ISTDs


Signal 1: DAD1 A, Sig=210,4 Ref=360,100


Signal 2: DAD1 B, Sig=230,4 Ref=360,100


Signal 3: DAD1 C, Sig=254,4 Ref=360,100
```

Peak #	RetTime [min]	Type	Width [min]	Area [mAU*s]	Height [mAU]	Area %
1	20.271	MM	0.6011	4690.02100	130.04813	49.9389
2	24.556	MM	0.7413	4701.49268	105.69769	50.0611

Totals : 9391.51367 235.74582

```
Signal 4: DAD1 D, Sig=280,4 Ref=360,100


Signal 5: DAD1 E, Sig=310,4 Ref=360,100

=====================================================================
                         *** End of Report ***
```

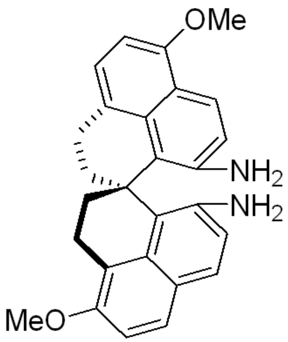

1j
(racemic)

Data File C:\CHEM32\1\DATA\SUN_12 2022-07-30 19-58-02\ZRH-7-60-LARGE.D
Sample Name:

```
=====================================================================
Acq. Operator   :                               Seq. Line :   9
Acq. Instrument : Instrument 1                   Location : Vial 32
Injection Date  : 7/30/2022 11:33:09 PM               Inj :   1
                                              Inj Volume : 5.000 µl
Acq. Method     : C:\CHEM32\1\DATA\SUN_12 2022-07-30 19-58-02\AD-30-40.M
Last changed    : 7/30/2022 10:51:18 PM
                  (modified after loading)
Analysis Method : C:\CHEM32\1\DATA\SUN_12 2022-12-29 09-07-21\2.D\DA.M (OD-05-10.M)
Last changed    : 1/12/2023 10:01:26 PM
                  (modified after loading)
Additional Info : Peak(s) manually integrated
```

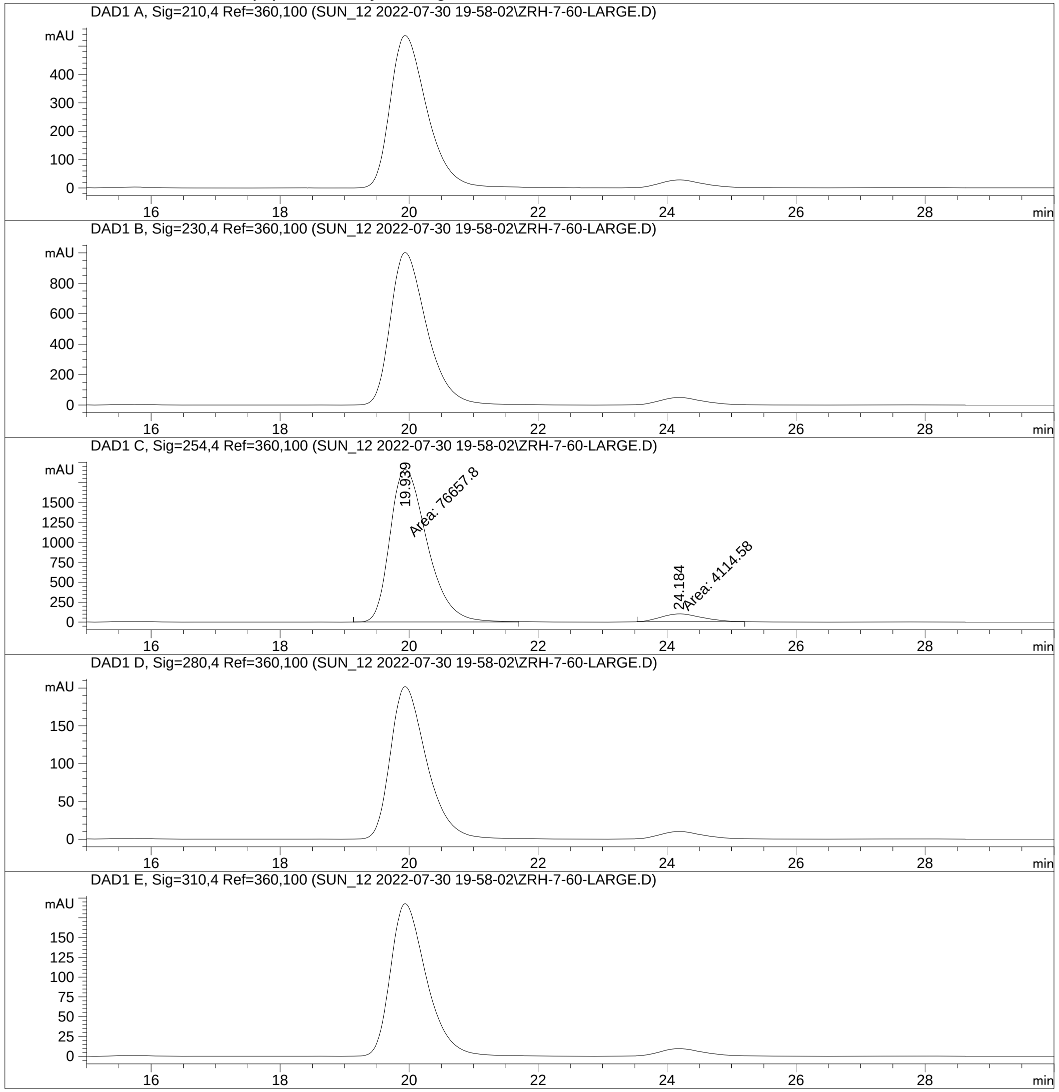

Data File C:\CHEM32\1\DATA\SUN_12 2022-07-30 19-58-02\ZRH-7-60-LARGE.D
Sample Name:

Area Percent Report

```
Sorted By             :      Signal
Multiplier            :      1.0000
Dilution              :      1.0000
Use Multiplier & Dilution Factor with ISTDs
```

Signal 1: DAD1 A, Sig=210,4 Ref=360,100

Signal 2: DAD1 B, Sig=230,4 Ref=360,100

Signal 3: DAD1 C, Sig=254,4 Ref=360,100

Peak #	RetTime [min]	Type	Width [min]	Area [mAU*s]	Height [mAU]	Area %
1	19.939	MM	0.6681	7.66578e4	1912.47180	94.9060
2	24.184	MM	0.7164	4114.58203	95.72015	5.0940

Totals : 8.07724e4 2008.19196

Signal 4: DAD1 D, Sig=280,4 Ref=360,100

Signal 5: DAD1 E, Sig=310,4 Ref=360,100

*** End of Report ***

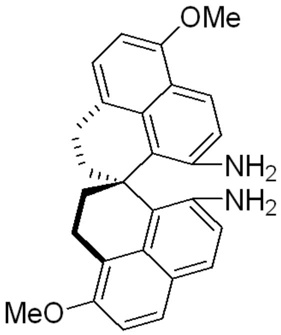

1j
(enantioenriched)

===

Acq. Operator : SYSTEM Seq. Line : 2
Sample Operator : SYSTEM
Acq. Instrument : HPLC Location : P2-C-01
Injection Date : 26/12/2022 5:18:07 pm Inj : 1
Inj Volume : 2.000 µl
Different Inj Volume from Sample Entry! Actual Inj Volume : 10.000 µl
Acq. Method : C:\Users\Public\Documents\ChemStation\1\Data\SUN\SUN 2022-12-26 17-10-27\AD3-20-30.M
Last changed : 26/12/2022 5:52:16 pm by SYSTEM
(modified after loading)
Analysis Method : C:\Users\Public\Documents\ChemStation\1\Data\SUN\SUN 2022-12-26 17-10-27\AD3-20-30.M (Sequence Method)
Last changed : 12/1/2023 10:35:07 pm by SYSTEM
(modified after loading)
Additional Info : Peak(s) manually integrated

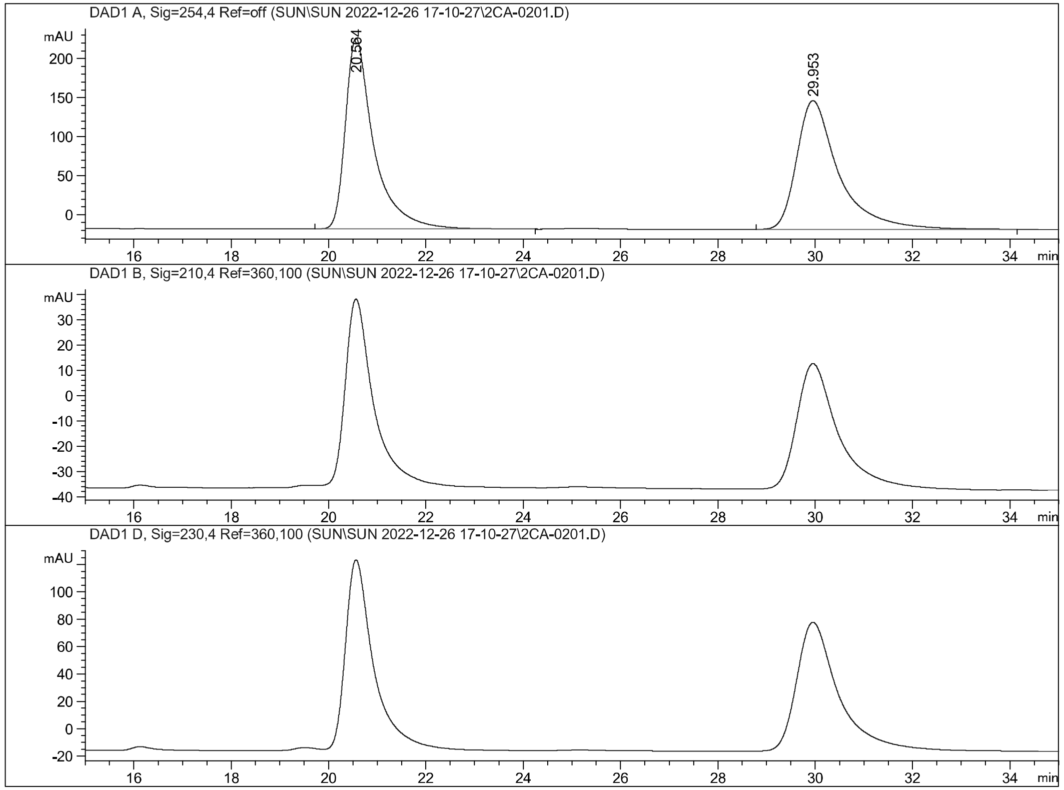

Data File C:\Users\P...Documents\ChemStation\1\Data\SUN\SUN 2022-12-26 17-10-27\2CA-0201.D
Sample Name: zrh-8-98-rac

===

Area Percent Report

===

Sorted By : Signal
Multiplier : 1.0000
Dilution : 1.0000
Use Multiplier & Dilution Factor with ISTDs

Signal 1: DAD1 A, Sig=254,4 Ref=off

Peak #	RetTime [min]	Type	Width [min]	Area [mAU*s]	Height [mAU]	Area %
1	20.564	BB	0.5953	9963.54492	244.57271	50.1716
2	29.953	BB	0.8682	9895.38867	164.88127	49.8284

Totals : 1.98589e4 409.45398

Signal 2: DAD1 B, Sig=210,4 Ref=360,100

Signal 3: DAD1 D, Sig=230,4 Ref=360,100

===

*** End of Report ***

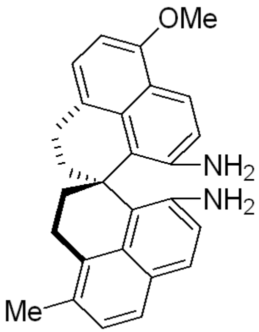

1k
(racemic)

Data File C:\Users\P...\ChemStation\1\Data\SUN\SUN 2022-12-29 10-12-37\OnlineEdited--008.D
Sample Name: zrh-8-101-2

===

Acq. Operator : SYSTEM Seq. Line : 8
Sample Operator : SYSTEM
Acq. Instrument : HPLC Location : P2-C-03
Injection Date : 29/12/2022 1:51:44 pm Inj : 1
Inj Volume : 2.000 µl
Different Inj Volume from Sample Entry! Actual Inj Volume : 10.000 µl
Acq. Method : C:\Users\Public\Documents\ChemStation\1\Data\SUN\SUN 2022-12-29 10-12-37\AD3-20-60.M
Last changed : 29/12/2022 1:51:19 pm by SYSTEM
(modified after loading)
Analysis Method : C:\Users\Public\Documents\ChemStation\1\Data\SUN\SUN 2022-12-29 10-12-37\AD3-20-60.M (Sequence Method)
Last changed : 12/1/2023 10:32:00 pm by SYSTEM
(modified after loading)
Additional Info : Peak(s) manually integrated

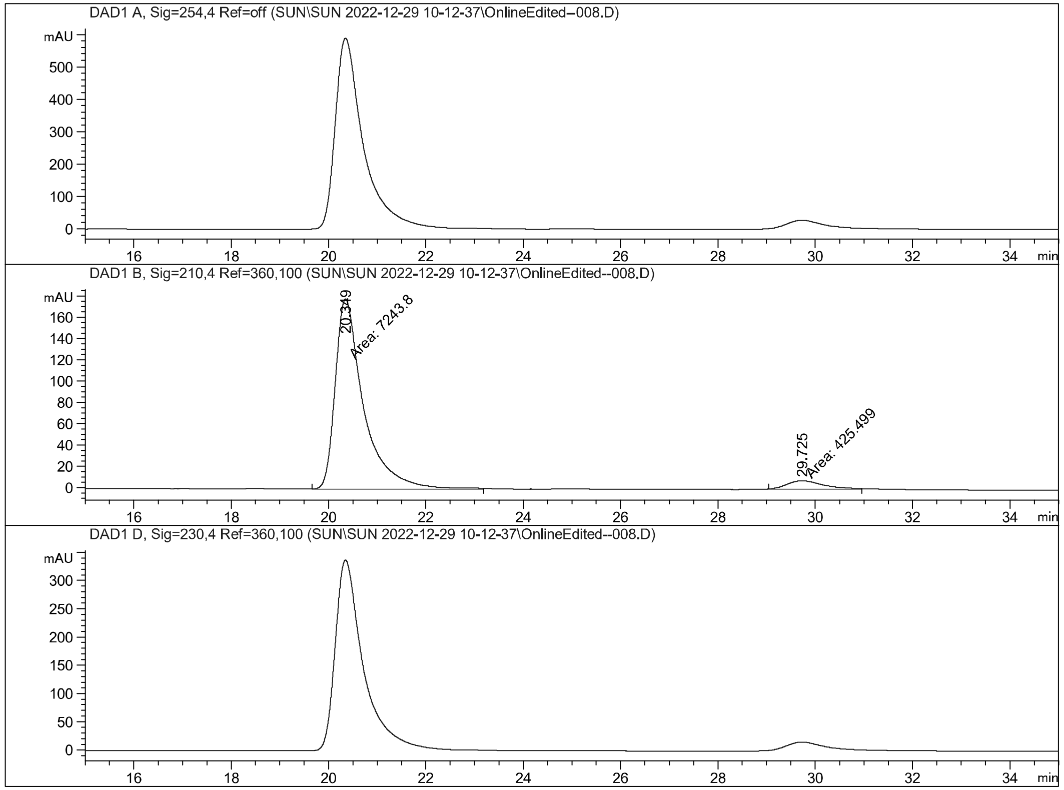

Data File C:\Users\P...\ChemStation\1\Data\SUN\SUN 2022-12-29 10-12-37\OnlineEdited--008.D
Sample Name: zrh-8-101-2

===

Area Percent Report

===

Sorted By : Signal
Multiplier : 1.0000
Dilution : 1.0000
Use Multiplier & Dilution Factor with ISTDs

Signal 1: DAD1 A, Sig=254,4 Ref=off

Signal 2: DAD1 B, Sig=210,4 Ref=360,100

Peak #	RetTime [min]	Type	Width [min]	Area [mAU*s]	Height [mAU]	Area %
1	20.349	MM	0.6762	7243.79785	178.55380	94.4519
2	29.725	MM	0.8863	425.49939	8.00151	5.5481

Totals : 7669.29724 186.55531

Signal 3: DAD1 D, Sig=230,4 Ref=360,100

===

*** End of Report ***

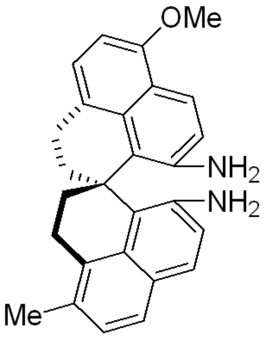

1k
(enantioenriched)

===

Acq. Operator : Seq. Line : 21
Acq. Instrument : Instrument 1 Location : Vial 31
Injection Date : 2/24/2023 5:43:02 PM Inj : 1
Inj Volume : 5.000 µl
Different Inj Volume from Sequence ! Actual Inj Volume : 10.000 µl
Acq. Method : C:\CHEM32\1\DATA\SUN_12 2023-02-24 09-22-47\AD-05-40.M
Last changed : 2/24/2023 5:42:10 PM
(modified after loading)
Analysis Method : C:\CHEM32\1\DATA\SUN_12 2023-02-24 09-22-47\OD-02-40-0.5.M (Sequence Method)
Last changed : 3/7/2023 8:34:08 PM
(modified after loading)
Additional Info : Peak(s) manually integrated

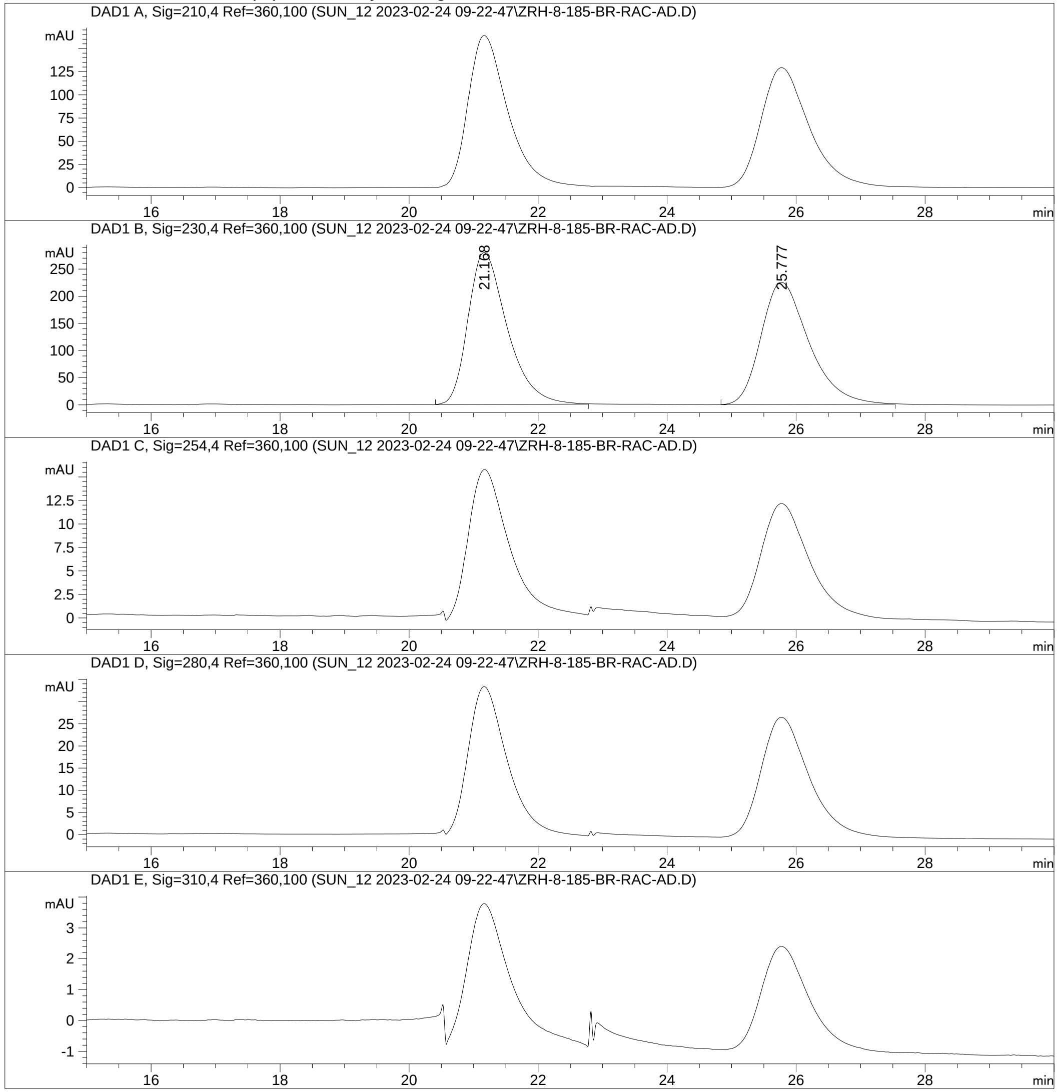

===
Area Percent Report
===

Sorted By : Signal
Multiplier : 1.0000
Dilution : 1.0000
Use Multiplier & Dilution Factor with ISTDs

Signal 1: DAD1 A, Sig=210,4 Ref=360,100

Signal 2: DAD1 B, Sig=230,4 Ref=360,100

Peak #	RetTime [min]	Type	Width [min]	Area [mAU*s]	Height [mAU]	Area %
1	21.168	BV	0.6417	1.17908e4	279.77090	50.3657
2	25.777	BB	0.7840	1.16196e4	225.19731	49.6343
Totals :				2.34105e4	504.96822	

Signal 3: DAD1 C, Sig=254,4 Ref=360,100

Signal 4: DAD1 D, Sig=280,4 Ref=360,100

Signal 5: DAD1 E, Sig=310,4 Ref=360,100

===
*** End of Report ***

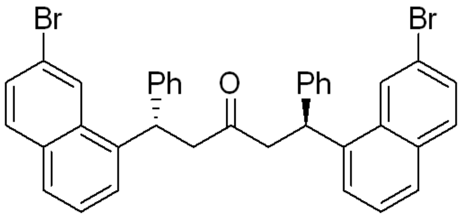

7a
(racemic)

Data File C:\CHEM32\1\DATA\SUN_12 2023-02-24 09-22-47\ZRH-8-185-BR-CHIRAL-AD.D
Sample Name:

===

```
Acq. Operator   :                               Seq. Line :  25
Acq. Instrument : Instrument 1                   Location : Vial 32
Injection Date  : 2/24/2023 7:12:16 PM                Inj :   1
                                               Inj Volume : 5.000 µl
Different Inj Volume from Sequence !     Actual Inj Volume : 10.000 µl
Acq. Method     : C:\CHEM32\1\DATA\SUN_12 2023-02-24 09-22-47\AD-05-40.M
Last changed    : 9/18/2012 9:41:46 AM
Analysis Method : C:\CHEM32\1\DATA\SUN_12 2023-02-24 09-22-47\OD-02-40-0.5.M (Sequence Method
                  )
Last changed    : 3/7/2023 8:34:08 PM
                  (modified after loading)
Additional Info : Peak(s) manually integrated
```

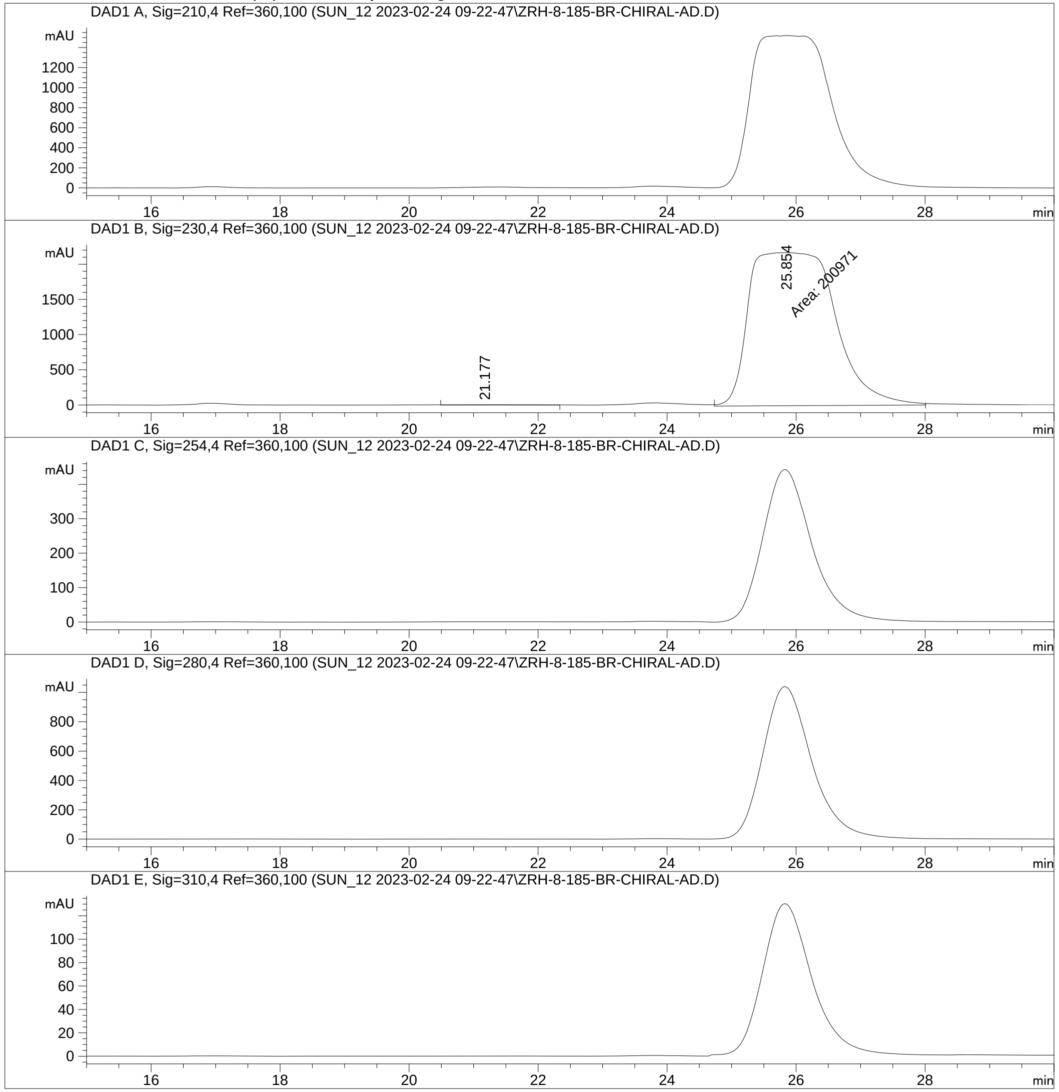

Data File C:\CHEM32\1\DATA\SUN_12 2023-02-24 09-22-47\ZRH-8-185-BR-CHIRAL-AD.D
Sample Name:

===
Area Percent Report
===

```
Sorted By             :      Signal
Multiplier            :      1.0000
Dilution              :      1.0000
Use Multiplier & Dilution Factor with ISTDs
```

Signal 1: DAD1 A, Sig=210,4 Ref=360,100

Signal 2: DAD1 B, Sig=230,4 Ref=360,100

Peak #	RetTime [min]	Type	Width [min]	Area [mAU*s]	Height [mAU]	Area %
1	21.177	BB	0.6411	416.37619	8.71991	0.2068
2	25.854	MM	1.5390	2.00971e5	2176.36743	99.7932
Totals :				2.01388e5	2185.08734	

Signal 3: DAD1 C, Sig=254,4 Ref=360,100

Signal 4: DAD1 D, Sig=280,4 Ref=360,100

Signal 5: DAD1 E, Sig=310,4 Ref=360,100

===
*** End of Report ***

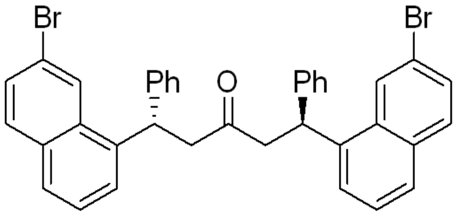

7a
(enantioenriched)

===

Acq. Operator	:	Seq. Line :	30
Acq. Instrument	: Instrument 1	Location :	Vial 31
Injection Date	: 3/11/2023 11:03:09 PM	Inj :	1
		Inj Volume :	5.000 µl

Different Inj Volume from Sequence !   Actual Inj Volume : 20.000 µl
Acq. Method   : C:\CHEM32\1\DATA\SUN_12 2023-03-11 13-29-47\AD-15-65.M
Last changed   : 3/11/2023 11:02:15 PM
                  (modified after loading)
Analysis Method : C:\CHEM32\1\METHODS\OD-15-10.M
Last changed   : 3/13/2023 10:03:24 AM
                  (modified after loading)
Additional Info : Peak(s) manually integrated

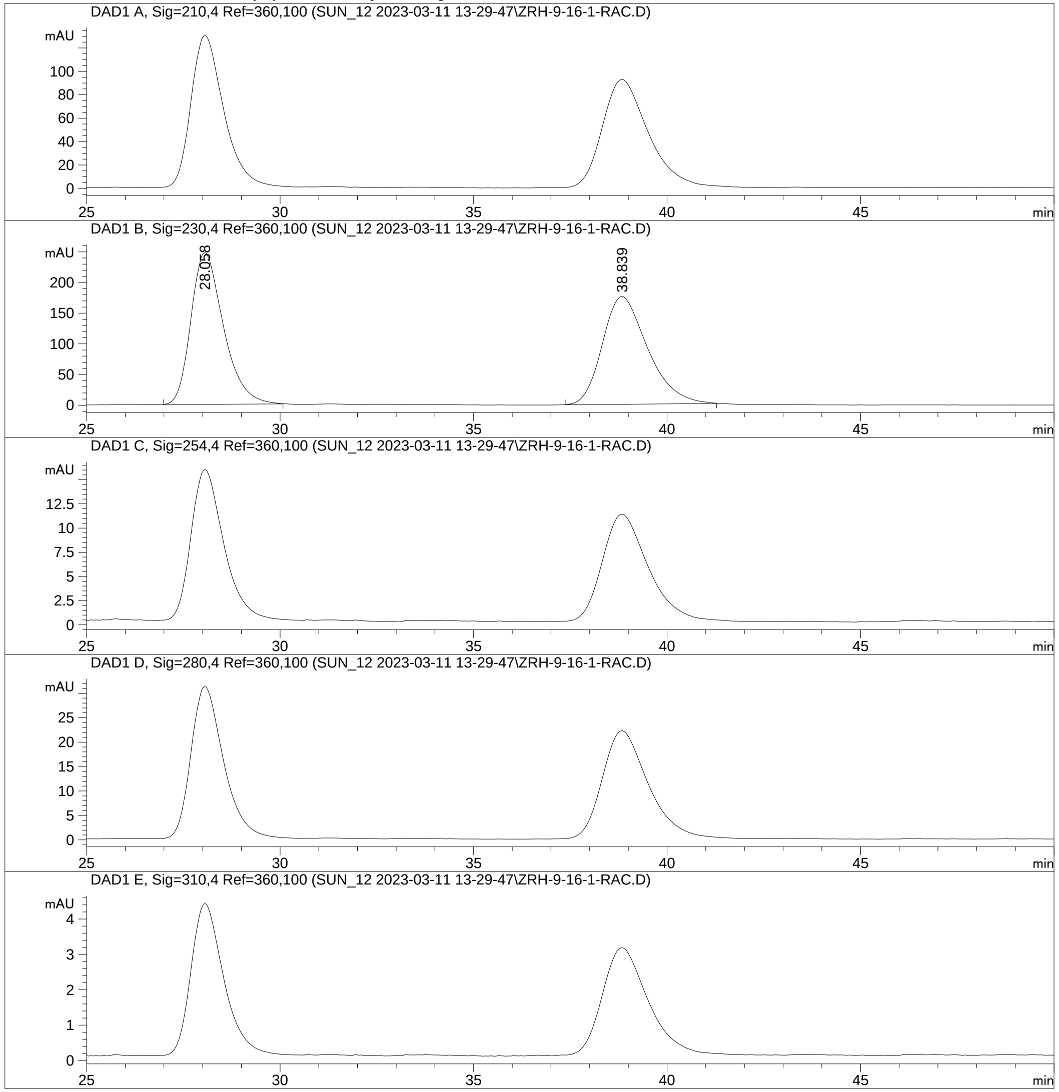

Data File C:\CHEM32\1\DATA\SUN_12 2023-03-11 13-29-47\ZRH-9-16-1-RAC.D
Sample Name:

===
Area Percent Report
===

Sorted By : Signal
Multiplier : 1.0000
Dilution : 1.0000
Use Multiplier & Dilution Factor with ISTDs

Signal 1: DAD1 A, Sig=210,4 Ref=360,100

Signal 2: DAD1 B, Sig=230,4 Ref=360,100

Peak #	RetTime [min]	Type	Width [min]	Area [mAU*s]	Height [mAU]	Area %
1	28.058	BB	0.8832	1.44534e4	247.54831	50.2873
2	38.839	BB	1.2264	1.42883e4	175.75740	49.7127

Totals : 2.87417e4   423.30571

Signal 3: DAD1 C, Sig=254,4 Ref=360,100

Signal 4: DAD1 D, Sig=280,4 Ref=360,100

Signal 5: DAD1 E, Sig=310,4 Ref=360,100

===
*** End of Report ***

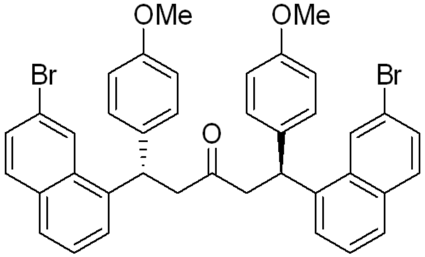

7b
(racemic)

Data File C:\CHEM32\1\DATA\SUN_12 2023-03-13 09-00-12\ZRH-9-16-1-CHIRAL.D
Sample Name:

===

```
Acq. Operator   :                               Seq. Line :  23
Acq. Instrument : Instrument 1                   Location : Vial 31
Injection Date  : 3/13/2023 4:23:14 PM                Inj :   1
                                               Inj Volume : 5.000 µl
Different Inj Volume from Sequence !     Actual Inj Volume : 10.000 µl
Acq. Method     : C:\CHEM32\1\DATA\SUN_12 2023-03-13 09-00-12\AD-15-65.M
Last changed    : 3/13/2023 4:22:22 PM
                  (modified after loading)
Analysis Method : C:\CHEM32\1\METHODS\OD-15-10.M
Last changed    : 3/13/2023 6:11:43 PM
                  (modified after loading)
Additional Info : Peak(s) manually integrated
```

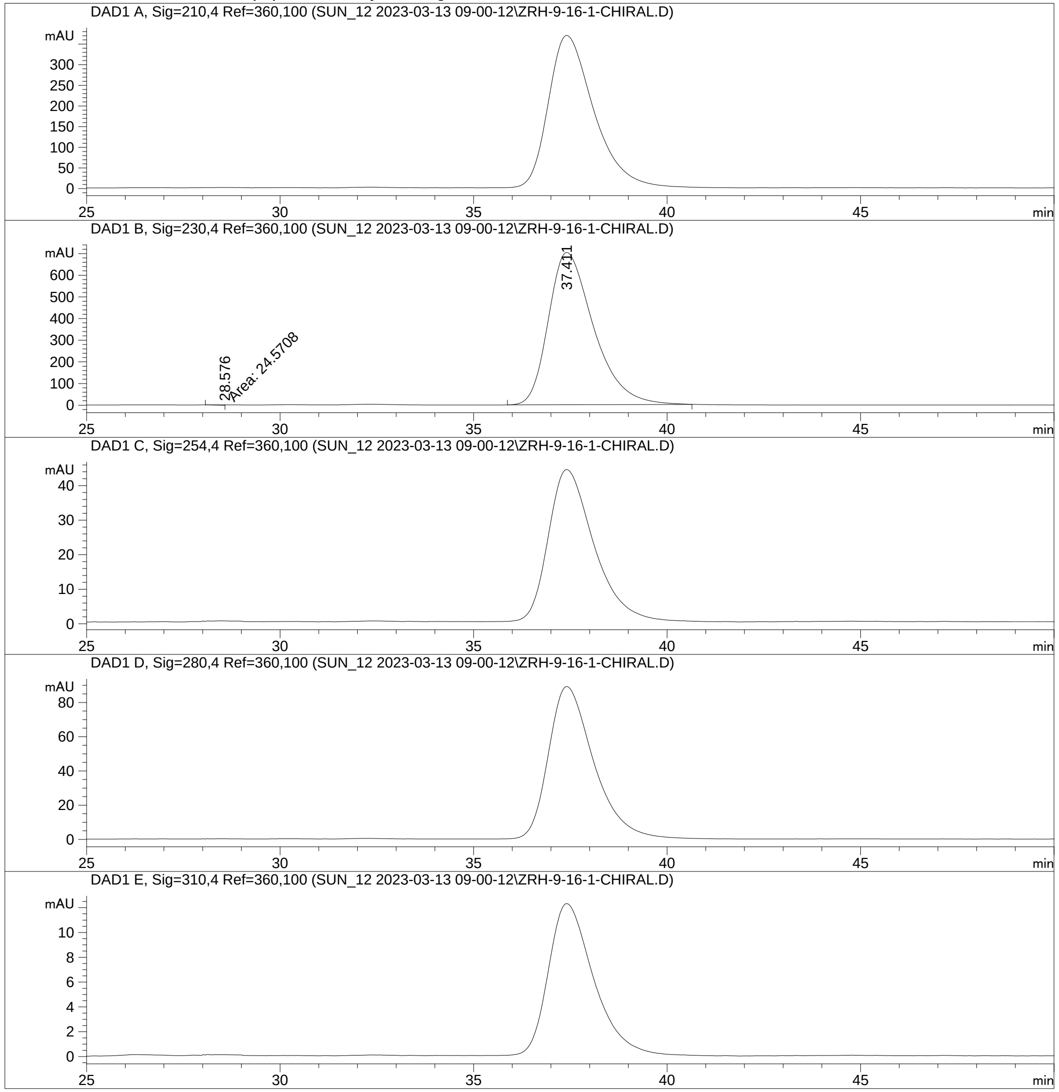

Data File C:\CHEM32\1\DATA\SUN_12 2023-03-13 09-00-12\ZRH-9-16-1-CHIRAL.D
Sample Name:

===
Area Percent Report
===

```
Sorted By             :      Signal
Multiplier            :      1.0000
Dilution              :      1.0000
Use Multiplier & Dilution Factor with ISTDs
```

Signal 1: DAD1 A, Sig=210,4 Ref=360,100

Signal 2: DAD1 B, Sig=230,4 Ref=360,100

Peak #	RetTime [min]	Type	Width [min]	Area [mAU*s]	Height [mAU]	Area %
1	28.576	MM	0.2081	24.57083	1.96750	0.0430
2	37.411	BB	1.2301	5.71414e4	703.16467	99.9570

Totals : 5.71660e4 705.13217

Signal 3: DAD1 C, Sig=254,4 Ref=360,100

Signal 4: DAD1 D, Sig=280,4 Ref=360,100

Signal 5: DAD1 E, Sig=310,4 Ref=360,100

===
*** End of Report ***

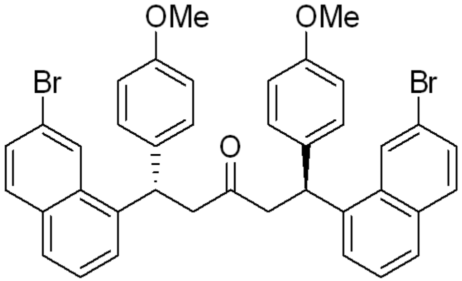

7b
(enantioenriched)

===

Acq. Operator : Seq. Line : 23
Acq. Instrument : Instrument 1 Location : Vial 37
Injection Date : 3/11/2023 7:49:48 PM Inj : 1
Inj Volume : 5.000 µl
Different Inj Volume from Sequence ! Actual Inj Volume : 20.000 µl
Acq. Method : C:\CHEM32\1\DATA\SUN_12 2023-03-11 13-29-47\AD-02-60.M
Last changed : 3/11/2023 7:48:02 PM
(modified after loading)
Analysis Method : C:\CHEM32\1\METHODS\OD-15-10.M
Last changed : 3/11/2023 8:51:15 PM
(modified after loading)
Additional Info : Peak(s) manually integrated

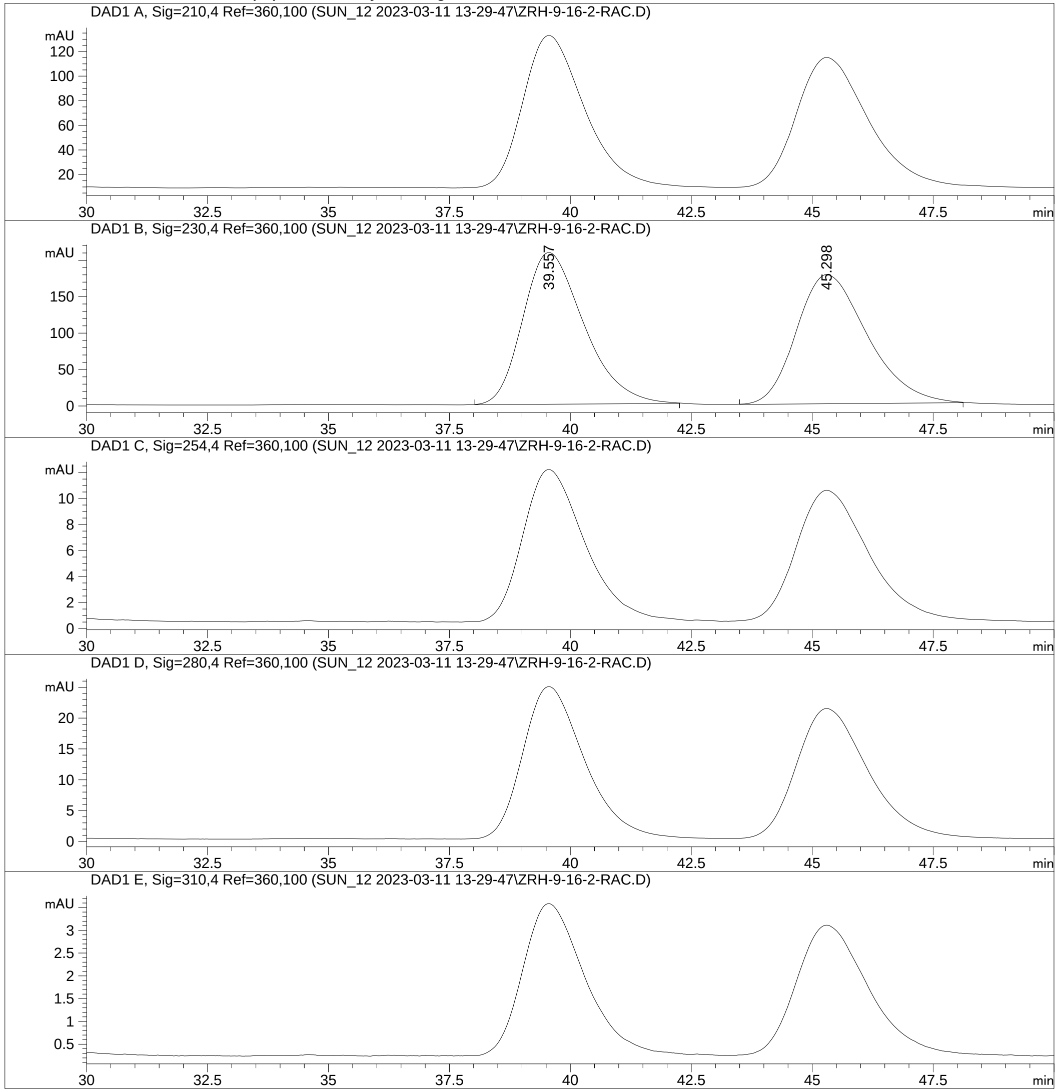

Data File C:\CHEM32\1\DATA\SUN_12 2023-03-11 13-29-47\ZRH-9-16-2-RAC.D
Sample Name:

===

Area Percent Report

===

Sorted By : Signal
Multiplier : 1.0000
Dilution : 1.0000
Use Multiplier & Dilution Factor with ISTDs

Signal 1: DAD1 A, Sig=210,4 Ref=360,100

Signal 2: DAD1 B, Sig=230,4 Ref=360,100

Peak #	RetTime [min]	Type	Width [min]	Area [mAU*s]	Height [mAU]	Area %
1	39.557	BB	1.3348	1.83353e4	208.33049	50.4046
2	45.298	BB	1.5423	1.80410e4	177.41779	49.5954

Totals : 3.63763e4 385.74828

Signal 3: DAD1 C, Sig=254,4 Ref=360,100

Signal 4: DAD1 D, Sig=280,4 Ref=360,100

Signal 5: DAD1 E, Sig=310,4 Ref=360,100

===

*** End of Report ***

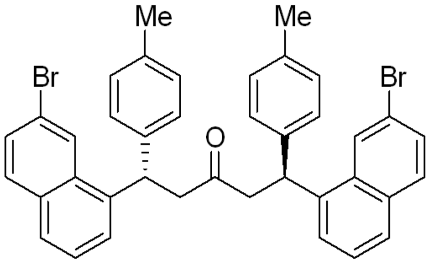

7c
(racemic)

===

Acq. Operator : Seq. Line : 33
Acq. Instrument : Instrument 1 Location : Vial 32
Injection Date : 3/13/2023 8:20:10 PM Inj : 1
Inj Volume : 5.000 µl
Different Inj Volume from Sequence ! Actual Inj Volume : 10.000 µl
Acq. Method : C:\CHEM32\1\DATA\SUN_12 2023-03-13 09-00-12\AD-02-60.M
Last changed : 3/12/2023 2:18:56 PM
Analysis Method : C:\CHEM32\1\DATA\SUN_12 2023-05-12 21-33-41\OD-10-30.M
Last changed : 6/8/2023 10:51:29 AM
(modified after loading)
Additional Info : Peak(s) manually integrated

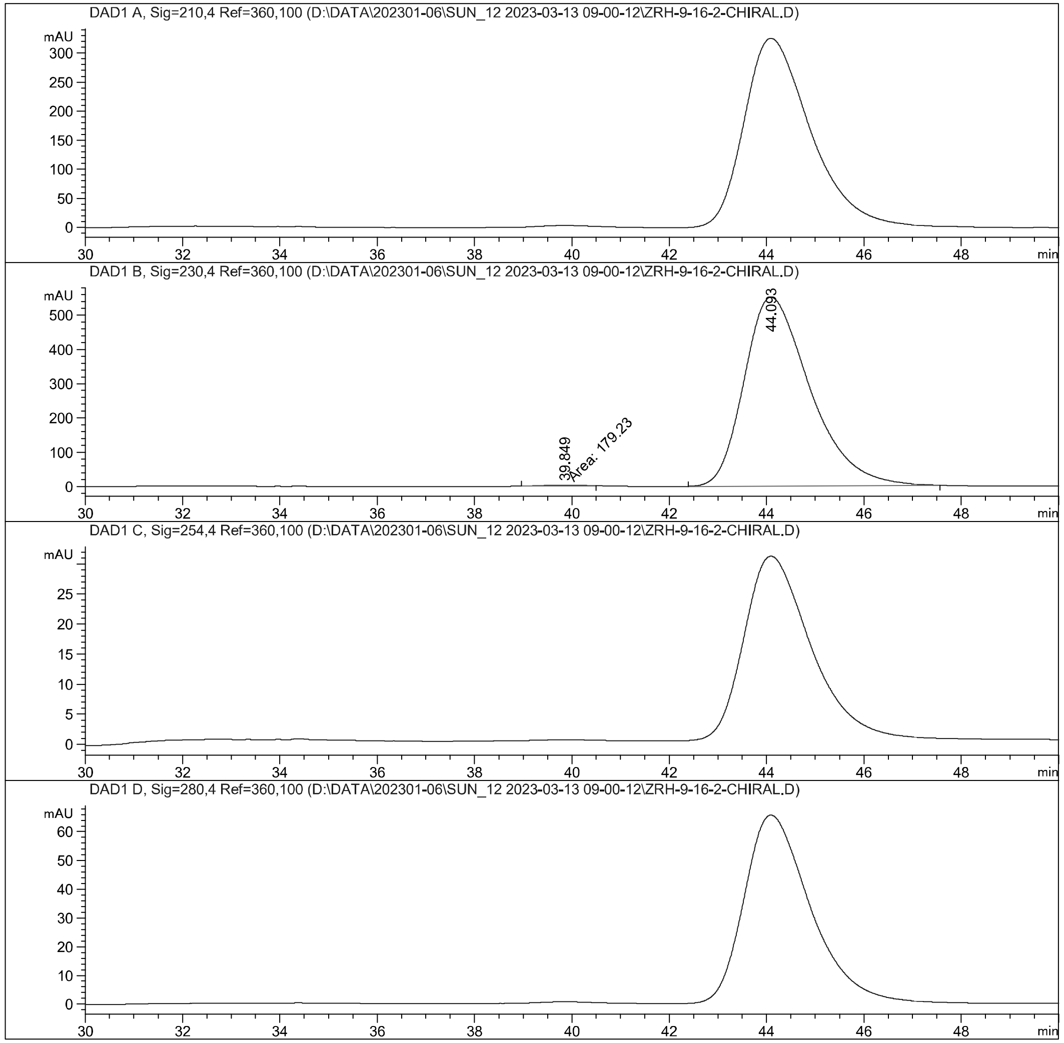

Data File D:\DATA\202301-06\SUN_12 2023-03-13 09-00-12\ZRH-9-16-2-CHIRAL.D
Sample Name:

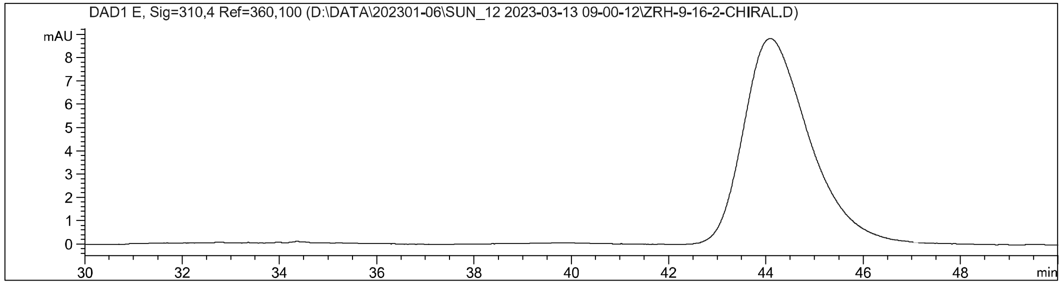

===

Area Percent Report

===

Sorted By : Signal
Multiplier : 1.0000
Dilution : 1.0000
Use Multiplier & Dilution Factor with ISTDs

Signal 1: DAD1 A, Sig=210,4 Ref=360,100

Signal 2: DAD1 B, Sig=230,4 Ref=360,100

Peak #	RetTime [min]	Type	Width [min]	Area [mAU*s]	Height [mAU]	Area %
1	39.849	MM	0.9387	179.23044	3.18226	0.3433
2	44.093	BB	1.4388	5.20321e4	551.44751	99.6567

Totals : 5.22114e4 554.62977

Signal 3: DAD1 C, Sig=254,4 Ref=360,100

Signal 4: DAD1 D, Sig=280,4 Ref=360,100

Signal 5: DAD1 E, Sig=310,4 Ref=360,100

===

*** End of Report ***

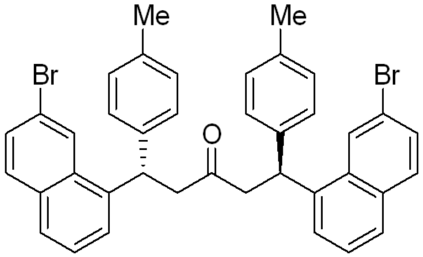

7c
(enantioenriched)

===

Acq. Operator : Seq. Line : 29
Acq. Instrument : Instrument 1 Location : Vial 32
Injection Date : 3/9/2023 7:43:20 PM Inj : 1
Inj Volume : 5.000 µl
Different Inj Volume from Sequence ! Actual Inj Volume : 30.000 µl
Acq. Method : C:\CHEM32\1\DATA\SUN_12 2023-03-09 11-51-16\AD-05-40.M
Last changed : 3/9/2023 8:36:31 PM
(modified after loading)
Analysis Method : C:\CHEM32\1\DATA\SUN_12 2023-05-12 21-33-41\OD-10-30.M
Last changed : 6/8/2023 12:08:29 PM
(modified after loading)
Additional Info : Peak(s) manually integrated

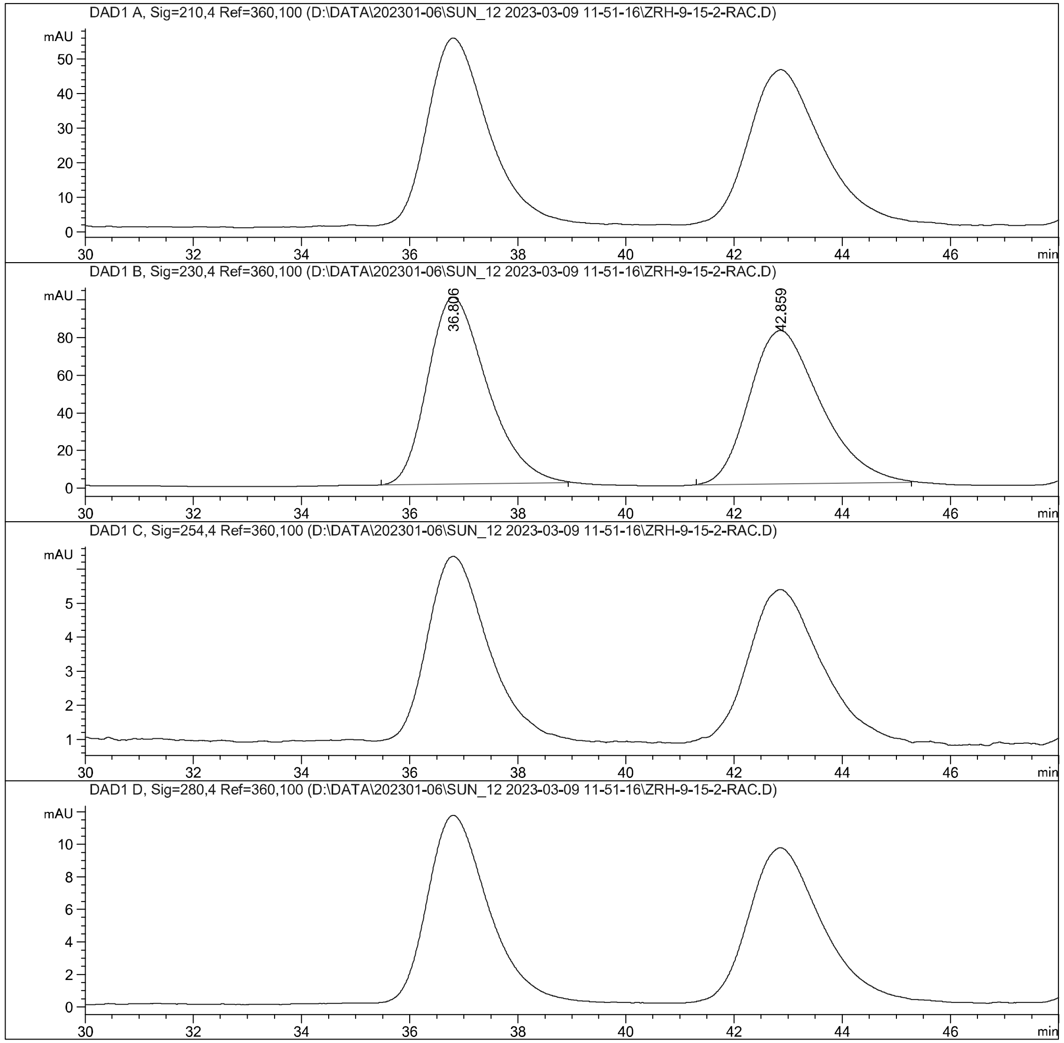

Data File D:\DATA\202301-06\SUN_12 2023-03-09 11-51-16\ZRH-9-15-2-RAC.D
Sample Name:

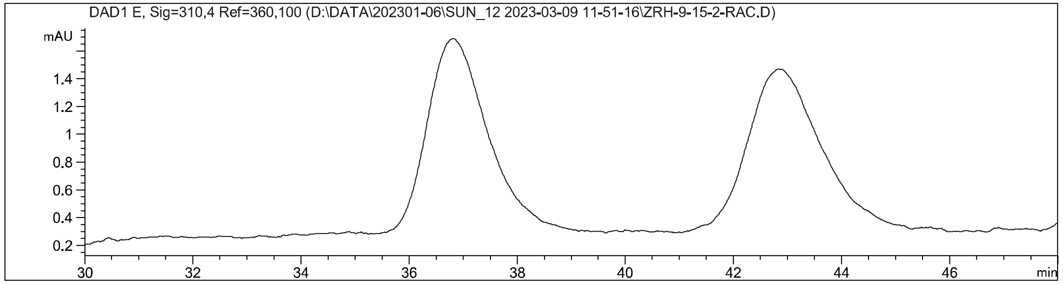

===

Area Percent Report

===

Sorted By : Signal
Multiplier : 1.0000
Dilution : 1.0000
Use Multiplier & Dilution Factor with ISTDs

Signal 1: DAD1 A, Sig=210,4 Ref=360,100

Signal 2: DAD1 B, Sig=230,4 Ref=360,100

Peak #	RetTime [min]	Type	Width [min]	Area [mAU*s]	Height [mAU]	Area %
1	36.806	BB	1.1721	7617.08447	99.38887	50.4584
2	42.859	BB	1.3637	7478.68213	81.56501	49.5416

Totals : 1.50958e4 180.95388

Signal 3: DAD1 C, Sig=254,4 Ref=360,100

Signal 4: DAD1 D, Sig=280,4 Ref=360,100

Signal 5: DAD1 E, Sig=310,4 Ref=360,100

===

*** End of Report ***

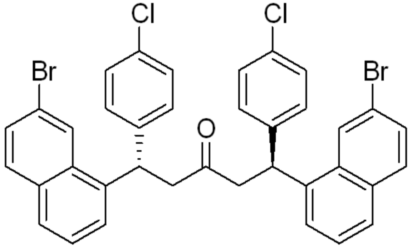

7d
(racemic)

Data File D:\DATA\202301-06\SUN_12 2023-03-09 11-51-16\ZRH-9-15-2.D
Sample Name:

===

Acq. Operator : Seq. Line : 59
Acq. Instrument : Instrument 1 Location : Vial 32
Injection Date : 3/10/2023 7:38:24 AM Inj : 1
Inj Volume : 5.000 µl
Different Inj Volume from Sequence ! Actual Inj Volume : 10.000 µl
Acq. Method : C:\CHEM32\1\DATA\SUN_12 2023-03-09 11-51-16\AD-05-60.M
Last changed : 3/10/2023 7:37:30 AM
(modified after loading)
Analysis Method : C:\CHEM32\1\DATA\SUN_12 2023-05-12 21-33-41\OD-10-30.M
Last changed : 6/8/2023 12:08:29 PM
(modified after loading)
Additional Info : Peak(s) manually integrated

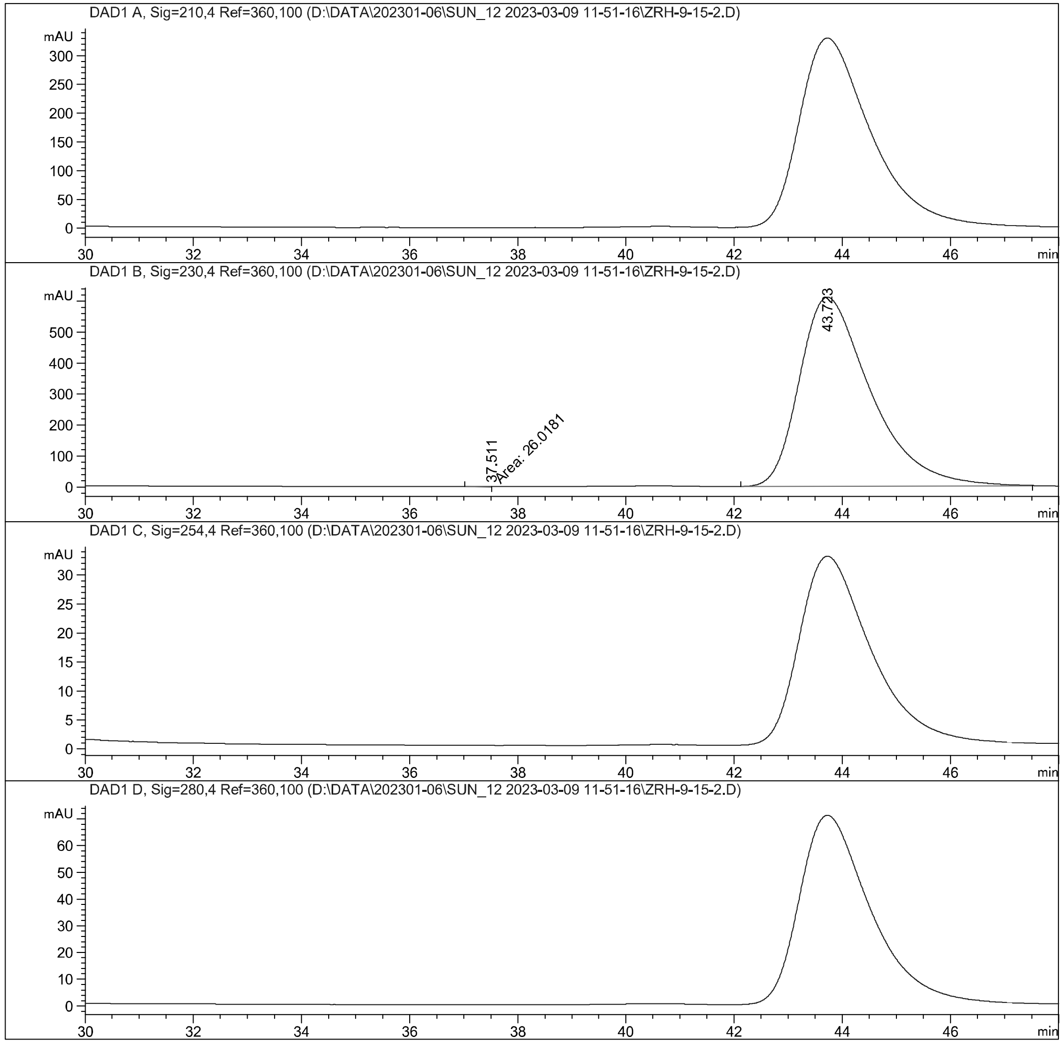

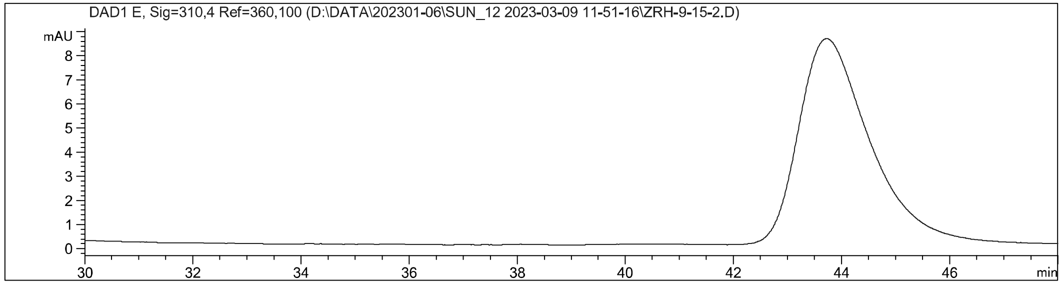

===

Area Percent Report

===

Sorted By : Signal
Multiplier : 1.0000
Dilution : 1.0000
Use Multiplier & Dilution Factor with ISTDs

Signal 1: DAD1 A, Sig=210,4 Ref=360,100

Signal 2: DAD1 B, Sig=230,4 Ref=360,100

Peak #	RetTime [min]	Type	Width [min]	Area [mAU*s]	Height [mAU]	Area %
1	37.511	MM	0.3122	26.01809	1.38876	0.0464
2	43.723	BB	1.3807	5.60142e4	610.35065	99.9536

Totals : 5.60402e4 611.73941

Signal 3: DAD1 C, Sig=254,4 Ref=360,100

Signal 4: DAD1 D, Sig=280,4 Ref=360,100

Signal 5: DAD1 E, Sig=310,4 Ref=360,100

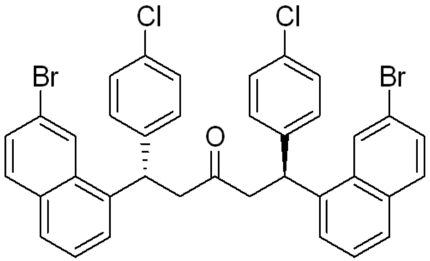

7d
(enantioenriched)

===

*** End of Report ***

===

Acq. Operator : Seq. Line : 80
Acq. Instrument : Instrument 1 Location : Vial 34
Injection Date : 3/10/2023 4:07:32 PM Inj : 1
Inj Volume : 5.000 µl
Different Inj Volume from Sequence ! Actual Inj Volume : 30.000 µl
Acq. Method : C:\CHEM32\1\DATA\SUN_12 2023-03-09 11-51-16\AD-10-30.M
Last changed : 1/18/2023 12:20:36 AM
Analysis Method : C:\CHEM32\1\DATA\SUN_12 2023-05-12 21-33-41\OD-10-30.M
Last changed : 6/8/2023 12:13:42 PM
(modified after loading)
Additional Info : Peak(s) manually integrated

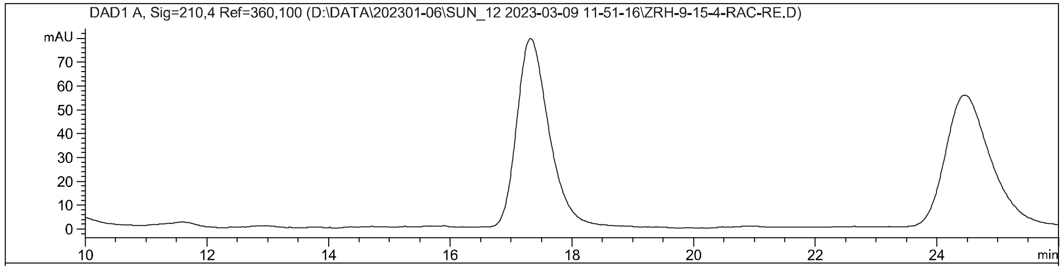

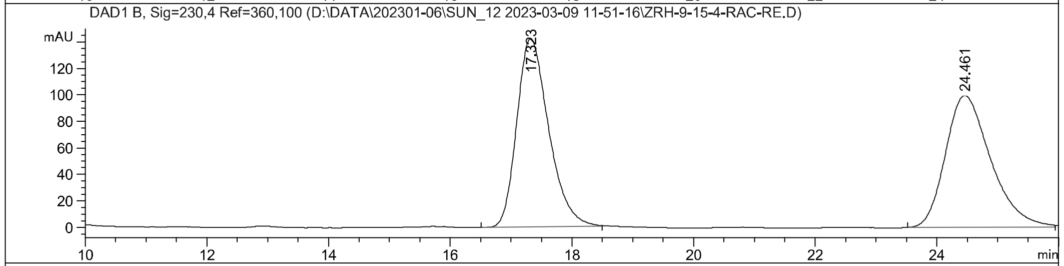

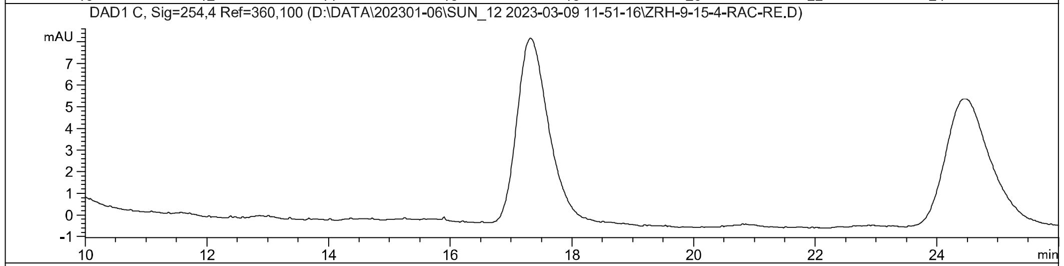

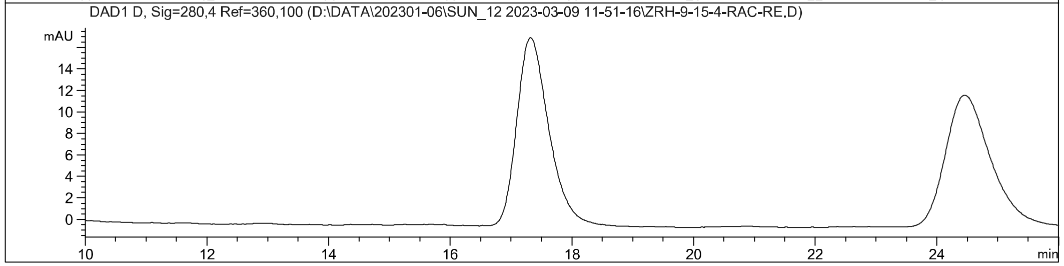

Data File D:\DATA\202301-06\SUN_12 2023-03-09 11-51-16\ZRH-9-15-4-RAC-RE.D
Sample Name:

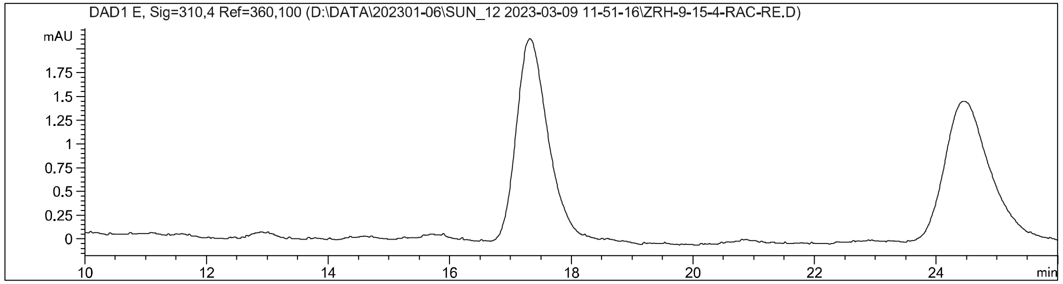

===

Area Percent Report

===

Sorted By : Signal
Multiplier : 1.0000
Dilution : 1.0000
Use Multiplier & Dilution Factor with ISTDs

Signal 1: DAD1 A, Sig=210,4 Ref=360,100

Signal 2: DAD1 B, Sig=230,4 Ref=360,100

Peak #	RetTime [min]	Type	Width [min]	Area [mAU*s]	Height [mAU]	Area %
1	17.323	BB	0.5478	5170.45752	142.32698	50.0407
2	24.461	BB	0.7866	5162.05371	99.61770	49.9593

Totals : 1.03325e4 241.94468

Signal 3: DAD1 C, Sig=254,4 Ref=360,100

Signal 4: DAD1 D, Sig=280,4 Ref=360,100

Signal 5: DAD1 E, Sig=310,4 Ref=360,100

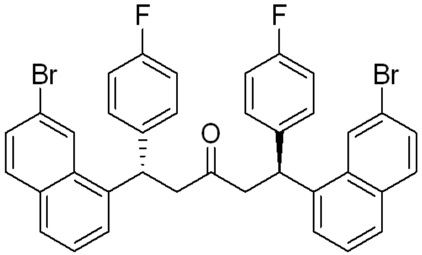

7e
(racemic)

===

*** End of Report ***

```
=====================================================================
Acq. Operator   :                                  Seq. Line :  57
Acq. Instrument : Instrument 1                      Location : Vial 34
Injection Date  : 3/10/2023 6:58:15 AM                   Inj :   1
                                                  Inj Volume : 5.000 µl
Different Inj Volume from Sequence !     Actual Inj Volume : 10.000 µl
Acq. Method     : C:\CHEM32\1\DATA\SUN_12 2023-03-09 11-51-16\AD-10-30.M
Last changed    : 1/18/2023 12:20:36 AM
Analysis Method : C:\CHEM32\1\METHODS\IH3-30-40.M
Last changed    : 6/20/2023 7:33:45 PM
                  (modified after loading)
Additional Info : Peak(s) manually integrated
```

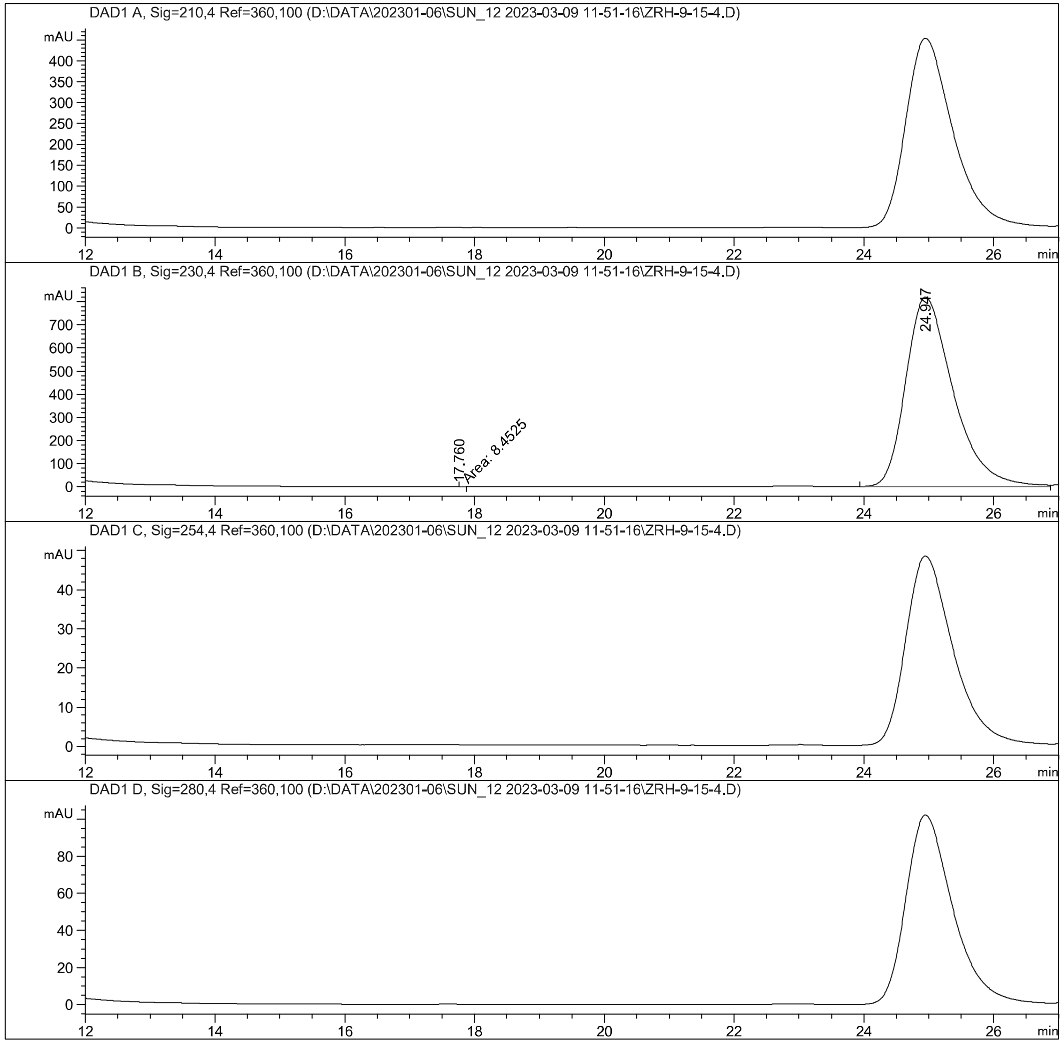

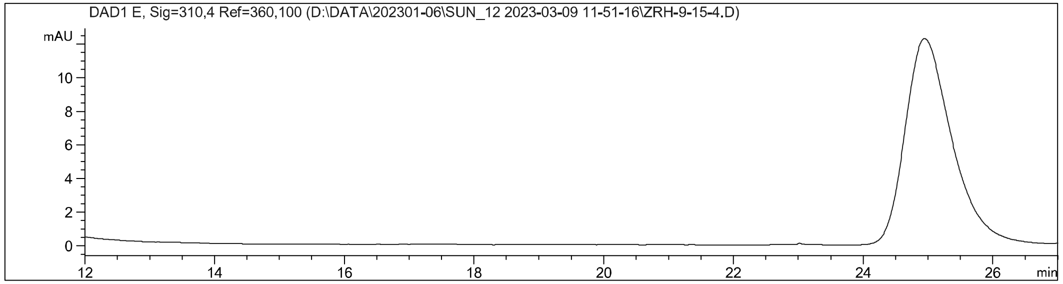


```
=====================================================================
                         Area Percent Report
=====================================================================

Sorted By             :      Signal
Multiplier            :      1.0000
Dilution              :      1.0000
Use Multiplier & Dilution Factor with ISTDs


Signal 1: DAD1 A, Sig=210,4 Ref=360,100


Signal 2: DAD1 B, Sig=230,4 Ref=360,100

Peak RetTime Type  Width     Area      Height     Area
  #   [min]        [min]   [mAU*s]     [mAU]        %
----|-------|----|-------|----------|----------|--------|
   1  17.760 MM    0.1101    8.45250    1.27960   0.0203
   2  24.947 BB    0.7702 4.15922e4  819.44794  99.9797

Totals :                  4.16007e4  820.72753


Signal 3: DAD1 C, Sig=254,4 Ref=360,100


Signal 4: DAD1 D, Sig=280,4 Ref=360,100


Signal 5: DAD1 E, Sig=310,4 Ref=360,100

=====================================================================
                         *** End of Report ***
```

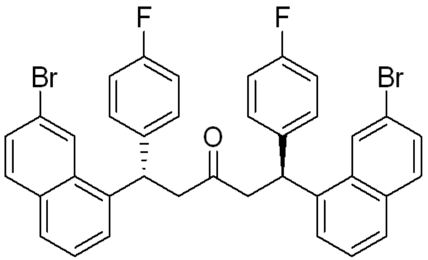

7e
(enantioenriched)

Data File C:\Users\P...emStation\1\Data\SUN\SUN 2025-08-25 21-14-37\016-P1-F1-zmh-rac-ia.D
Sample Name: zmh-rac-ia

===

Acq. Operator : SYSTEM Seq. Line : 16
Sample Operator : SYSTEM
Acq. Instrument : HPLC Location : P1-F-01
Injection Date : 26/8/2025 1:24:44 am Inj : 1
Inj Volume : 2.000 µl
Different Inj Volume from Sample Entry! Actual Inj Volume : 10.000 µl
Acq. Method : C:\Users\Public\Documents\ChemStation\1\Data\SUN\SUN 2025-08-25 21-14-37\IA3-10-20.M
Last changed : 15/8/2022 10:26:10 pm by SYSTEM
Analysis Method : C:\Users\Public\Documents\ChemStation\1\Data\SUN\SUN 2025-08-25 21-14-37\IA3-10-20.M (Sequence Method)
Last changed : 26/8/2025 11:13:15 am by SYSTEM
(modified after loading)
Additional Info : Peak(s) manually integrated

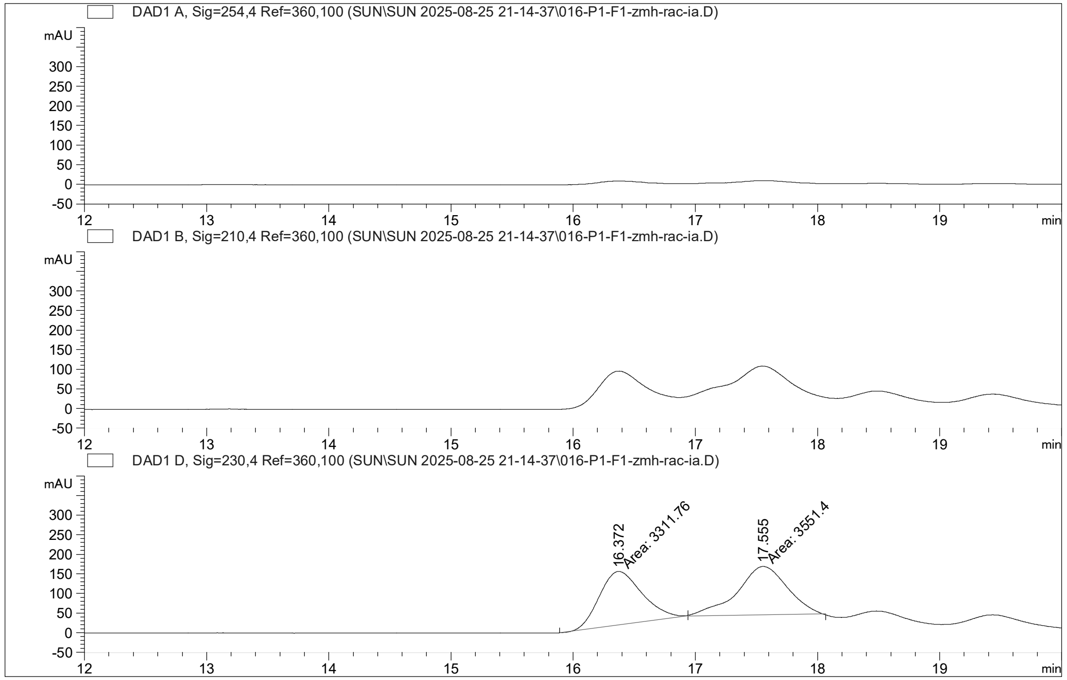

===

Area Percent Report

===

Sorted By : Signal
Multiplier : 1.0000
Dilution : 1.0000
Use Multiplier & Dilution Factor with ISTDs

Data File C:\Users\P...emStation\1\Data\SUN\SUN 2025-08-25 21-14-37\016-P1-F1-zmh-rac-ia.D
Sample Name: zmh-rac-ia

Signal 1: DAD1 A, Sig=254,4 Ref=360,100

Signal 2: DAD1 B, Sig=210,4 Ref=360,100

Signal 3: DAD1 D, Sig=230,4 Ref=360,100

Peak #	RetTime [min]	Type	Width [min]	Area [mAU*s]	Height [mAU]	Area %
1	16.372	MM	0.4041	3311.76465	136.57603	48.2542
2	17.555	MM	0.4773	3551.39771	124.01162	51.7458
Totals :				6863.16235	260.58765	

===

*** End of Report ***

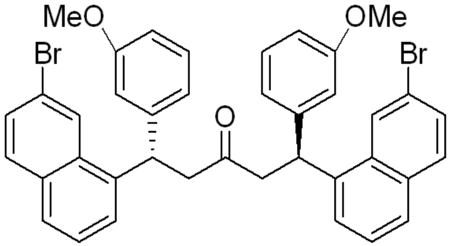

7f
(racemic)

===

Acq. Operator : SYSTEM   Seq. Line : 2
Sample Operator : SYSTEM
Acq. Instrument : HPLC   Location : P1-F-02
Injection Date : 26/8/2025 10:43:04 am   Inj : 1
Inj Volume : 2.000 µl
Different Inj Volume from Sample Entry! Actual Inj Volume : 10.000 µl
Acq. Method : C:\Users\Public\Documents\ChemStation\1\Data\SUN\SUN 2025-08-26 10-29-25\IA3-10-20.M
Last changed : 15/8/2022 10:26:10 pm by SYSTEM
Analysis Method : C:\Users\Public\Documents\ChemStation\1\Data\SUN\SUN 2025-08-26 10-29-25\IA3-10-20.M (Sequence Method)
Last changed : 26/8/2025 11:17:09 am by SYSTEM
(modified after loading)
Additional Info : Peak(s) manually integrated

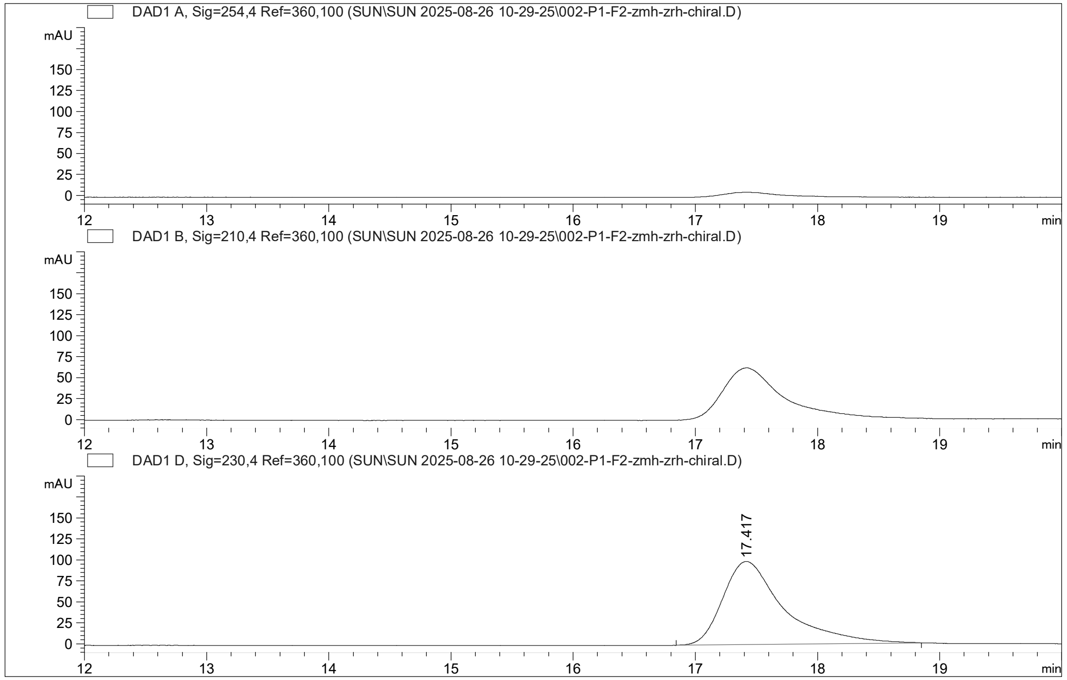

===

Area Percent Report

===

Sorted By : Signal
Multiplier : 1.0000
Dilution : 1.0000
Use Multiplier & Dilution Factor with ISTDs

Data File C:\Users\P...ation\1\Data\SUN\SUN 2025-08-26 10-29-25\002-P1-F2-zmh-zrh-chiral.D
Sample Name: zmh-zrh-chiral

Signal 1: DAD1 A, Sig=254,4 Ref=360,100

Signal 2: DAD1 B, Sig=210,4 Ref=360,100

Signal 3: DAD1 D, Sig=230,4 Ref=360,100

Peak #	RetTime [min]	Type	Width [min]	Area [mAU*s]	Height [mAU]	Area %
1	17.417	BV R	0.4088	3429.39404	99.08686	100.0000
Totals :				3429.39404	99.08686	

===

*** End of Report ***

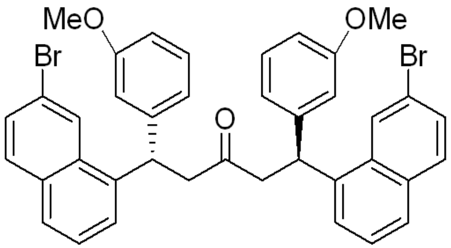

7f
(enantioenriched)

Data File C:\CHEM32\1\DATA\SUN_12 2023-03-09 11-51-16\ZRH-9-15-5-RAC9802.D
Sample Name:

===

```
Acq. Operator   :                              Seq. Line :  35
Acq. Instrument : Instrument 1                  Location : Vial 35
Injection Date  : 3/9/2023 11:05:29 PM               Inj :   1
                                              Inj Volume : 5.000 µl
Different Inj Volume from Sequence !     Actual Inj Volume : 10.000 µl
Acq. Method     : C:\CHEM32\1\DATA\SUN_12 2023-03-09 11-51-16\AD-02-60.M
Last changed    : 9/23/2020 10:59:45 PM
Analysis Method : C:\CHEM32\1\METHODS\OD-15-10.M
Last changed    : 3/10/2023 9:29:40 AM
                  (modified after loading)
Additional Info : Peak(s) manually integrated
```

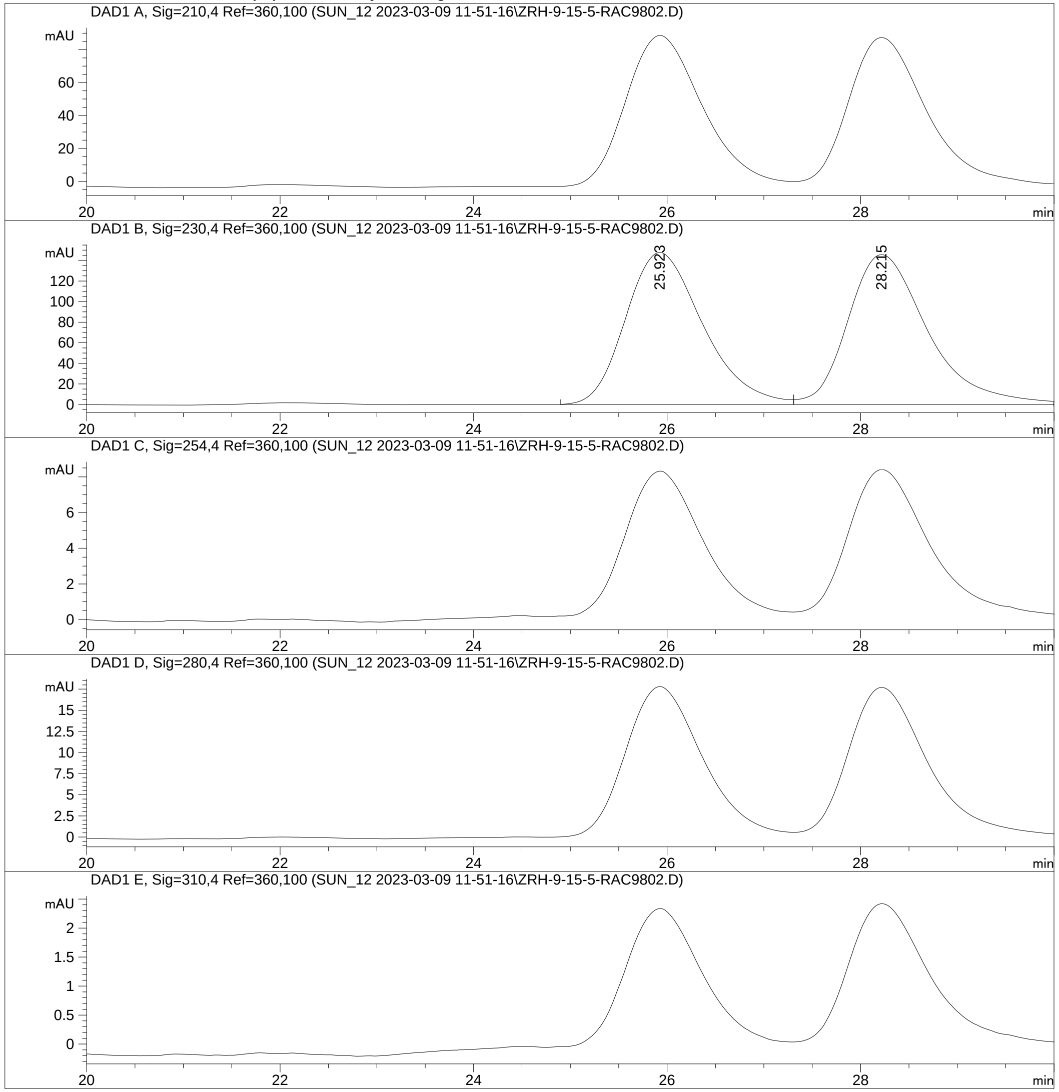

Data File C:\CHEM32\1\DATA\SUN_12 2023-03-09 11-51-16\ZRH-9-15-5-RAC9802.D
Sample Name:

===

Area Percent Report

===

```
Sorted By             :      Signal
Multiplier            :      1.0000
Dilution              :      1.0000
Use Multiplier & Dilution Factor with ISTDs
```

Signal 1: DAD1 A, Sig=210,4 Ref=360,100

Signal 2: DAD1 B, Sig=230,4 Ref=360,100

Peak #	RetTime [min]	Type	Width [min]	Area [mAU*s]	Height [mAU]	Area %
1	25.923	BV	0.8566	8214.72168	147.75970	49.6230
2	28.215	VBA	0.8816	8339.52637	145.74196	50.3770

Totals : 1.65542e4 293.50166

Signal 3: DAD1 C, Sig=254,4 Ref=360,100

Signal 4: DAD1 D, Sig=280,4 Ref=360,100

Signal 5: DAD1 E, Sig=310,4 Ref=360,100

===

*** End of Report ***

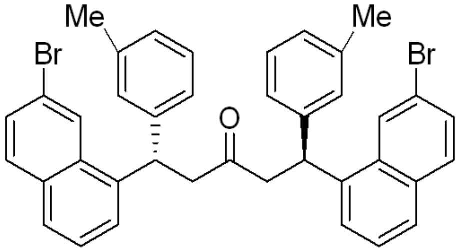

7g
(racemic)

===

Acq. Operator   :   Seq. Line : 88
Acq. Instrument : Instrument 1   Location : Vial 35
Injection Date : 3/10/2023 7:23:39 PM   Inj :   1
                                              Inj Volume : 5.000 µl
Different Inj Volume from Sequence !   Actual Inj Volume : 10.000 µl
Acq. Method   : C:\CHEM32\1\DATA\SUN_12 2023-03-09 11-51-16\AD-02-30.M
Last changed   : 3/10/2023 6:51:48 PM
                  (modified after loading)
Analysis Method : C:\CHEM32\1\METHODS\OD-15-10.M
Last changed   : 3/10/2023 8:26:10 PM
                  (modified after loading)
Additional Info : Peak(s) manually integrated

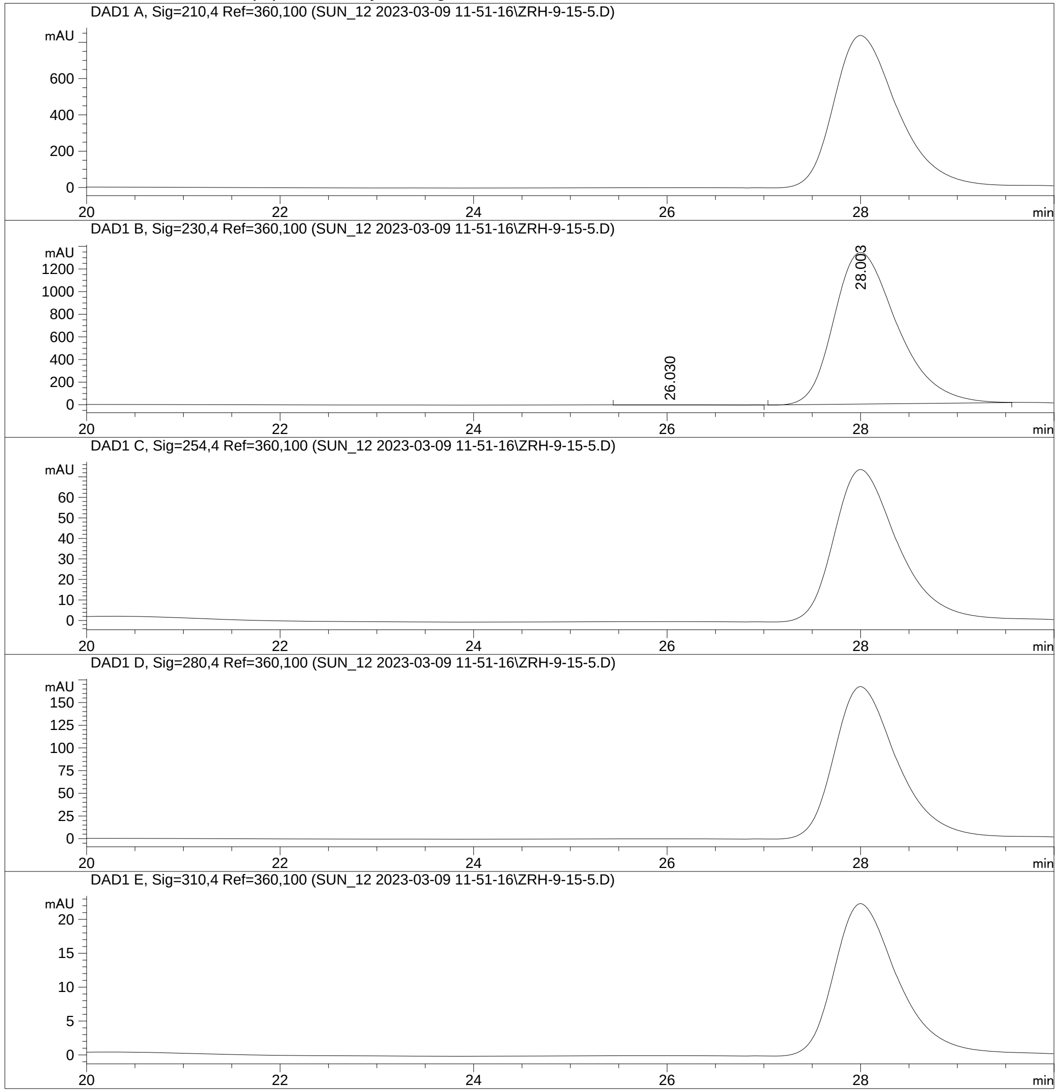

Data File C:\CHEM32\1\DATA\SUN_12 2023-03-09 11-51-16\ZRH-9-15-5.D
Sample Name:

===

Area Percent Report

===

Sorted By   :   Signal
Multiplier   :   1.0000
Dilution   :   1.0000
Use Multiplier & Dilution Factor with ISTDs

Signal 1: DAD1 A, Sig=210,4 Ref=360,100

Signal 2: DAD1 B, Sig=230,4 Ref=360,100

Peak #	RetTime [min]	Type	Width [min]	Area [mAU*s]	Height [mAU]	Area %
1	26.030	BB	0.5596	135.09174	3.03343	0.2213
2	28.003	BB	0.6966	6.09090e4	1339.21826	99.7787

Totals :   6.10441e4 1342.25170

Signal 3: DAD1 C, Sig=254,4 Ref=360,100

Signal 4: DAD1 D, Sig=280,4 Ref=360,100

Signal 5: DAD1 E, Sig=310,4 Ref=360,100

===

*** End of Report ***

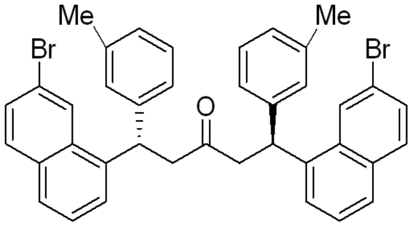

7g
(enantioenriched)

Acq. Operator :
Seq. Line : 82
Acq. Instrument : Instrument 1
Location : Vial 36
Injection Date : 3/10/2023 4:50:28 PM
Inj : 1
Inj Volume : 5.000 µl
Different Inj Volume from Sequence ! Actual Inj Volume : 30.000 µl
Acq. Method : C:\CHEM32\1\DATA\SUN_12 2023-03-09 11-51-16\AD-05-40.M
Last changed : 9/18/2012 9:41:46 AM
Analysis Method : C:\CHEM32\1\METHODS\OD-15-10.M
Last changed : 3/10/2023 6:01:41 PM
(modified after loading)
Additional Info : Peak(s) manually integrated

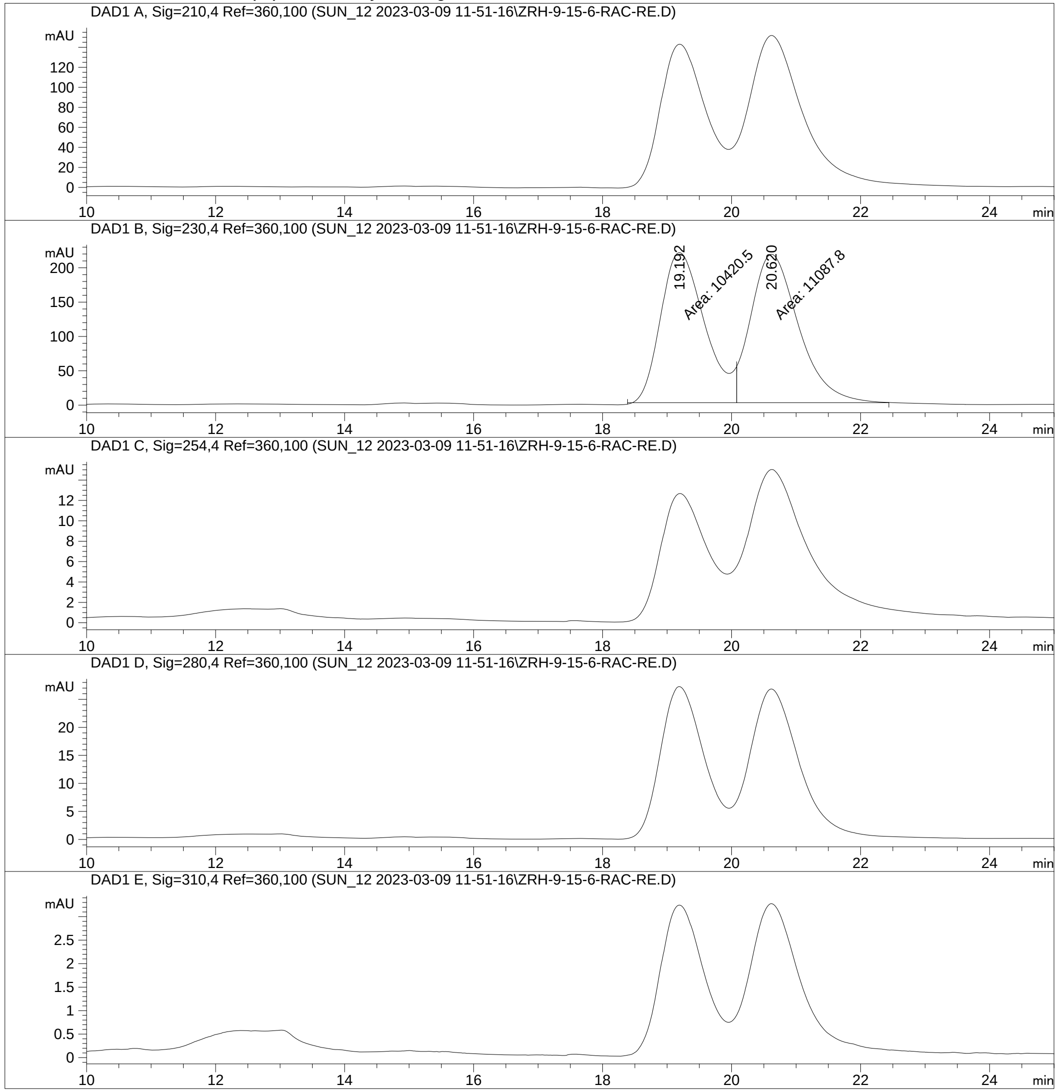

Data File C:\CHEM32\1\DATA\SUN_12 2023-03-09 11-51-16\ZRH-9-15-6-RAC-RE.D
Sample Name:

Area Percent Report

Sorted By : Signal
Multiplier : 1.0000
Dilution : 1.0000
Use Multiplier & Dilution Factor with ISTDs

Signal 1: DAD1 A, Sig=210,4 Ref=360,100

Signal 2: DAD1 B, Sig=230,4 Ref=360,100

Peak #	RetTime [min]	Type	Width [min]	Area [mAU*s]	Height [mAU]	Area %
1	19.192	MF	0.7938	1.04205e4	218.79633	48.4486
2	20.620	FM	0.8545	1.10878e4	216.25494	51.5514

Totals : 2.15083e4 435.05127

Signal 3: DAD1 C, Sig=254,4 Ref=360,100

Signal 4: DAD1 D, Sig=280,4 Ref=360,100

Signal 5: DAD1 E, Sig=310,4 Ref=360,100

*** End of Report ***

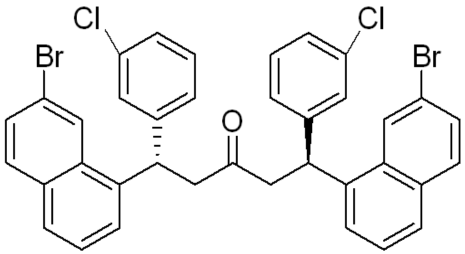

7h
(racemic)

```
=====================================================================
Acq. Operator   :                            Seq. Line :  83
Acq. Instrument : Instrument 1                Location : Vial 31
Injection Date  : 3/10/2023 5:41:33 PM             Inj :   1
                                            Inj Volume : 5.000 µl
Different Inj Volume from Sequence !     Actual Inj Volume : 10.000 µl
Acq. Method     : C:\CHEM32\1\DATA\SUN_12 2023-03-09 11-51-16\AD-05-40.M
Last changed    : 3/10/2023 6:13:08 PM
                  (modified after loading)
Analysis Method : C:\CHEM32\1\METHODS\OD-15-10.M
Last changed    : 3/10/2023 6:15:09 PM
                  (modified after loading)
Additional Info : Peak(s) manually integrated
```

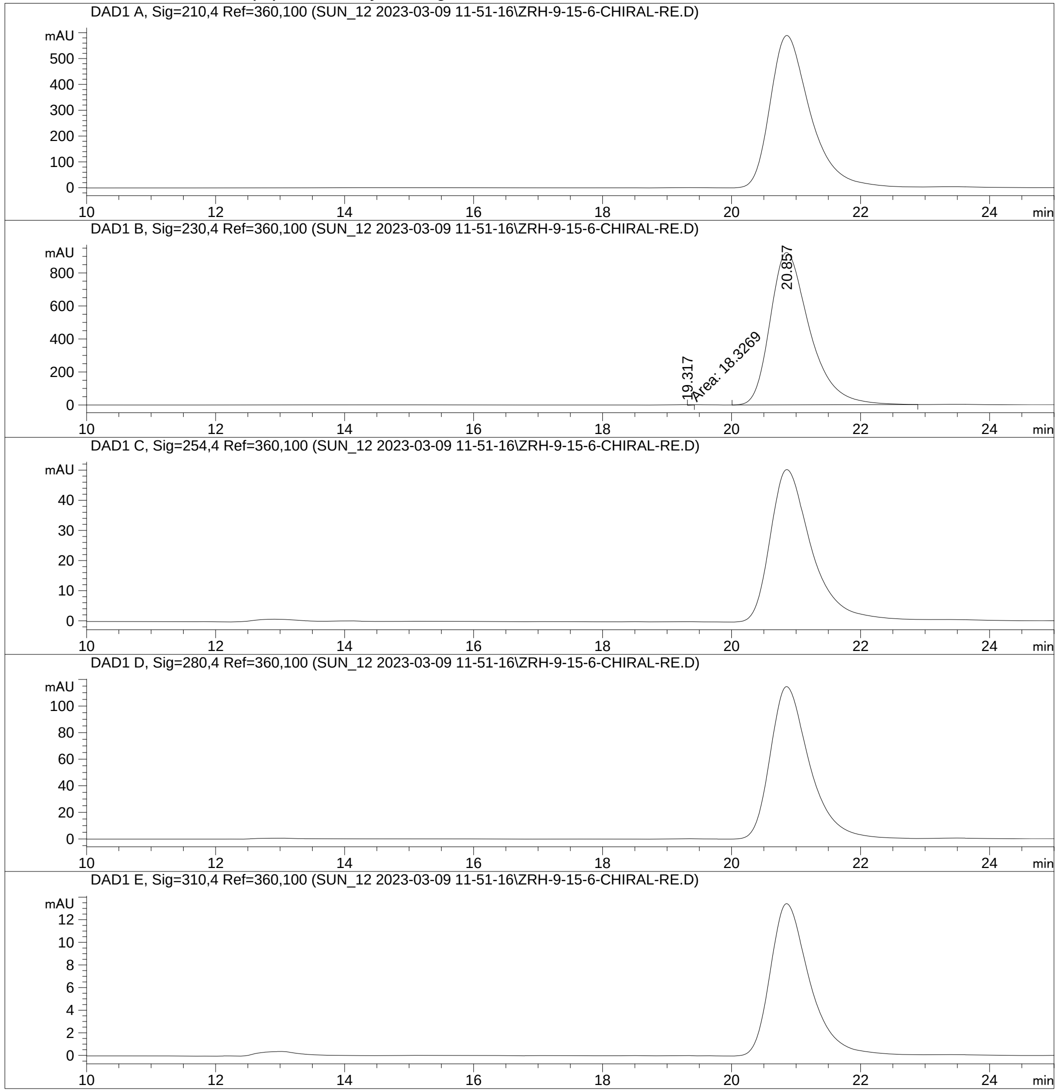

Data File C:\CHEM32\1\DATA\SUN_12 2023-03-09 11-51-16\ZRH-9-15-6-CHIRAL-RE.D
Sample Name:

```
=====================================================================
                        Area Percent Report
=====================================================================

Sorted By             :      Signal
Multiplier            :      1.0000
Dilution              :      1.0000
Use Multiplier & Dilution Factor with ISTDs


Signal 1: DAD1 A, Sig=210,4 Ref=360,100


Signal 2: DAD1 B, Sig=230,4 Ref=360,100
```

Peak #	RetTime [min]	Type	Width [min]	Area [mAU*s]	Height [mAU]	Area %
1	19.317	MM	0.0785	18.32687	3.89060	0.0468
2	20.857	BB	0.6407	3.91338e4	922.92322	99.9532

Totals : 3.91521e4   926.81382

```
Signal 3: DAD1 C, Sig=254,4 Ref=360,100


Signal 4: DAD1 D, Sig=280,4 Ref=360,100


Signal 5: DAD1 E, Sig=310,4 Ref=360,100

=====================================================================
                        *** End of Report ***
```

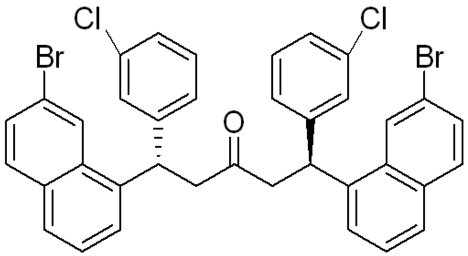

7h
(enantioenriched)

Data File C:\CHEM32\1\DATA\SUN_12 2023-03-09 11-51-16\ZRH-9-15-7-RAC.D
Sample Name:

===

Acq. Operator : Seq. Line : 8
Acq. Instrument : Instrument 1 Location : Vial 32
Injection Date : 3/9/2023 1:14:27 PM Inj : 1
Inj Volume : 5.000 µl
Different Inj Volume from Sequence ! Actual Inj Volume : 20.000 µl
Acq. Method : C:\CHEM32\1\DATA\SUN_12 2023-03-09 11-51-16\AD-02-60.M
Last changed : 3/9/2023 1:44:57 PM
(modified after loading)
Analysis Method : C:\CHEM32\1\METHODS\OD-15-10.M
Last changed : 3/9/2023 1:49:04 PM
(modified after loading)
Additional Info : Peak(s) manually integrated

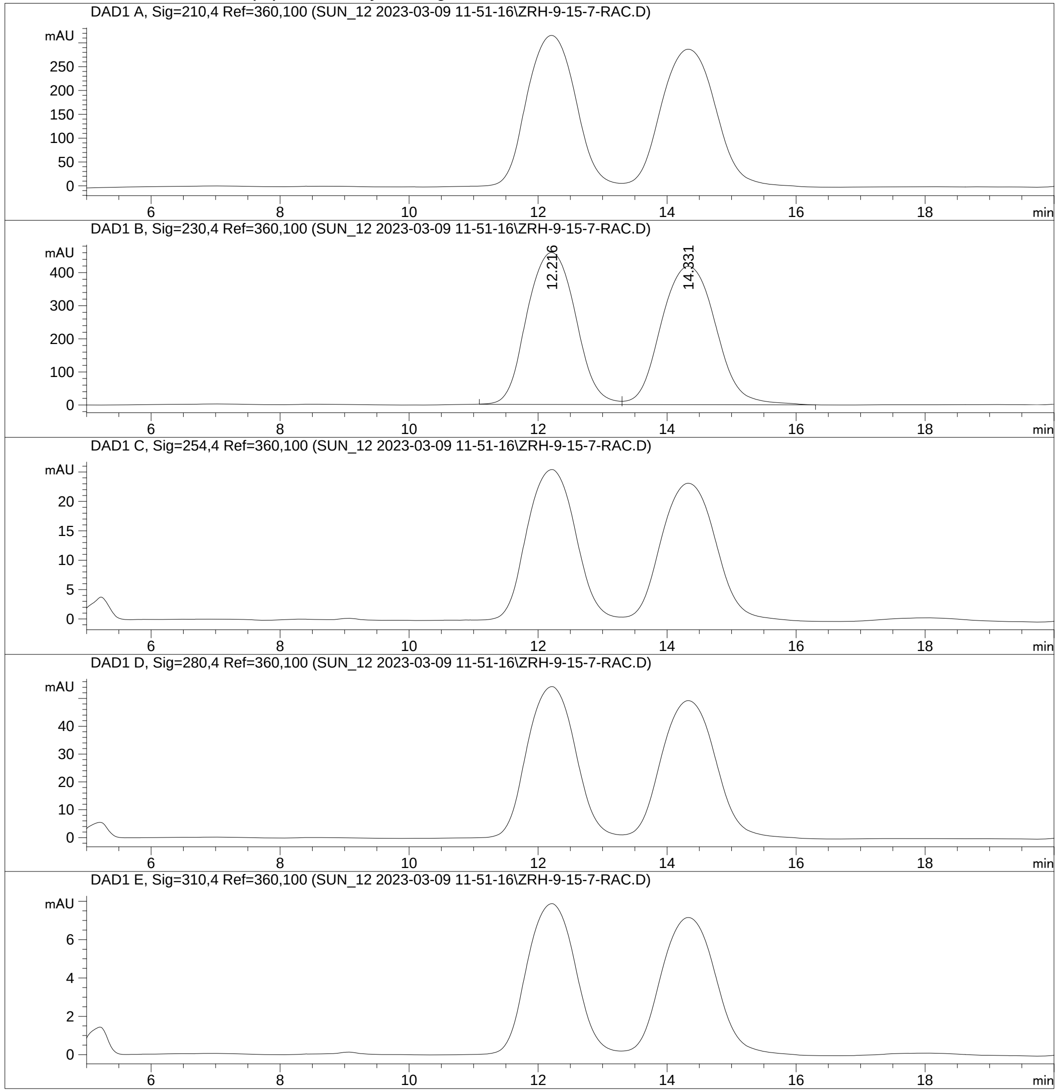

Data File C:\CHEM32\1\DATA\SUN_12 2023-03-09 11-51-16\ZRH-9-15-7-RAC.D
Sample Name:

===

Area Percent Report

===

Sorted By : Signal
Multiplier : 1.0000
Dilution : 1.0000
Use Multiplier & Dilution Factor with ISTDs

Signal 1: DAD1 A, Sig=210,4 Ref=360,100

Signal 2: DAD1 B, Sig=230,4 Ref=360,100

Peak #	RetTime [min]	Type	Width [min]	Area [mAU*s]	Height [mAU]	Area %
1	12.216	BV	0.8566	2.39479e4	459.08542	49.3708
2	14.331	VB	0.9574	2.45583e4	418.03079	50.6292

Totals : 4.85062e4 877.11621

Signal 3: DAD1 C, Sig=254,4 Ref=360,100

Signal 4: DAD1 D, Sig=280,4 Ref=360,100

Signal 5: DAD1 E, Sig=310,4 Ref=360,100

===

*** End of Report ***

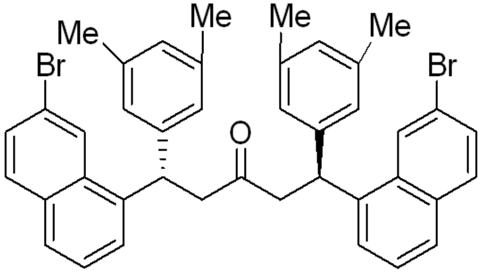

7i
(racemic)

===

Acq. Operator : Seq. Line : 87
Acq. Instrument : Instrument 1 Location : Vial 33
Injection Date : 3/10/2023 6:52:41 PM Inj : 1
Inj Volume : 5.000 µl
Acq. Method : C:\CHEM32\1\DATA\SUN_12 2023-03-09 11-51-16\AD-02-30.M
Last changed : 3/10/2023 6:51:48 PM
(modified after loading)
Analysis Method : C:\CHEM32\1\METHODS\OD-15-10.M
Last changed : 3/10/2023 8:23:15 PM
(modified after loading)
Additional Info : Peak(s) manually integrated

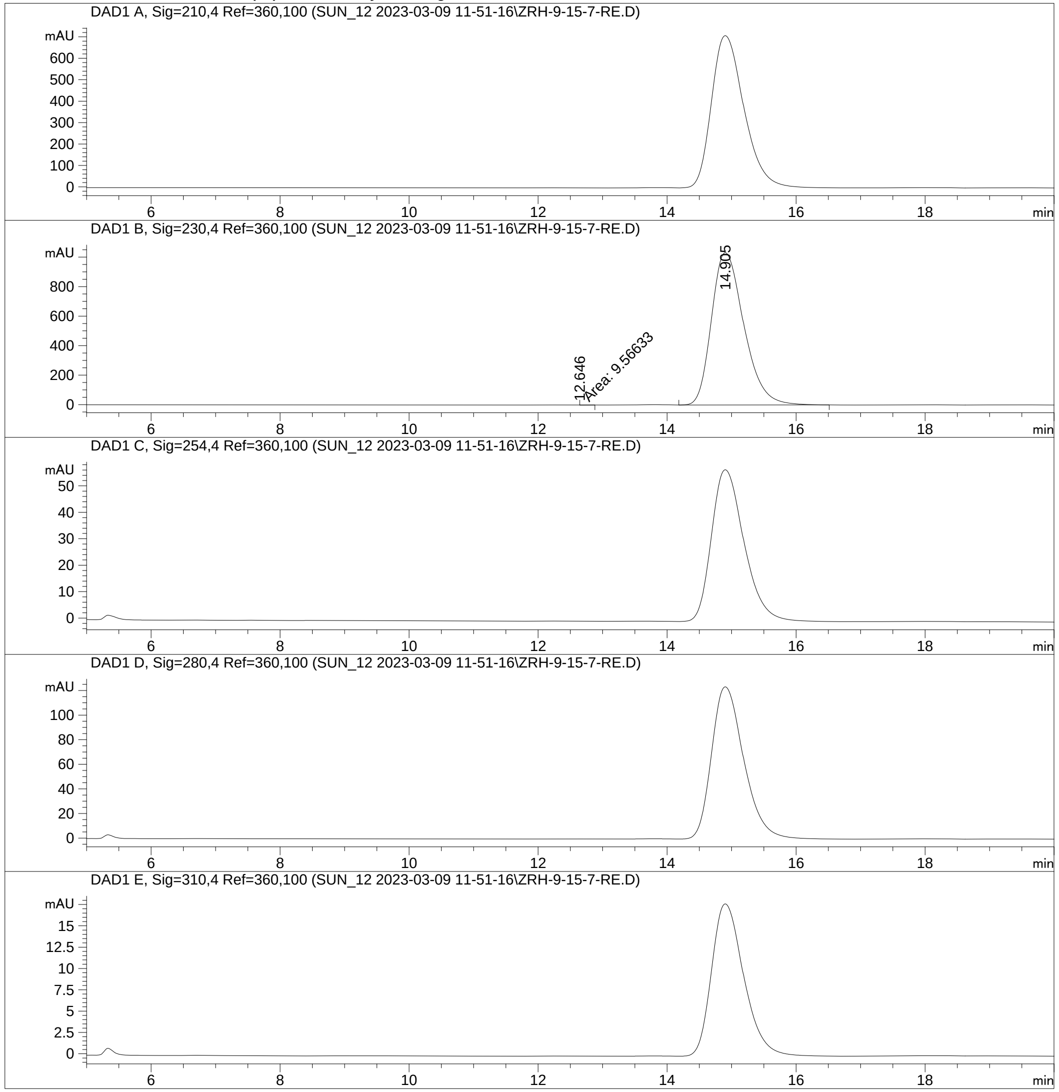

===

Area Percent Report

===

Sorted By : Signal
Multiplier : 1.0000
Dilution : 1.0000
Use Multiplier & Dilution Factor with ISTDs

Signal 1: DAD1 A, Sig=210,4 Ref=360,100

Signal 2: DAD1 B, Sig=230,4 Ref=360,100

Peak #	RetTime [min]	Type	Width [min]	Area [mAU*s]	Height [mAU]	Area %
1	12.646	MM	0.1297	9.56633	1.22920	0.0265
2	14.905	VB	0.5410	3.60516e4	1033.53735	99.9735

Totals : 3.60612e4 1034.76655

Signal 3: DAD1 C, Sig=254,4 Ref=360,100

Signal 4: DAD1 D, Sig=280,4 Ref=360,100

Signal 5: DAD1 E, Sig=310,4 Ref=360,100

===

*** End of Report ***

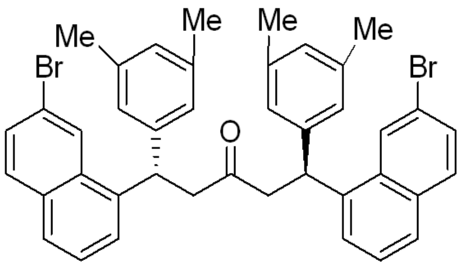

7i
(enantioenriched)

===

```
Acq. Operator   :                              Seq. Line :  14
Acq. Instrument : Instrument 1                  Location : Vial 32
Injection Date  : 6/6/2023 4:12:59 PM                 Inj :   1
                                             Inj Volume : 5.000 µl
Different Inj Volume from Sequence !     Actual Inj Volume : 15.000 µl
Acq. Method     : C:\CHEM32\1\DATA\SUN_12 2023-06-06 09-47-41\OD-10-60.M
Last changed    : 6/6/2023 4:56:18 PM
                  (modified after loading)
Analysis Method : C:\CHEM32\1\DATA\SUN_12 2023-05-12 21-33-41\OD-10-30.M
Last changed    : 6/8/2023 12:34:30 PM
                  (modified after loading)
Additional Info : Peak(s) manually integrated
```

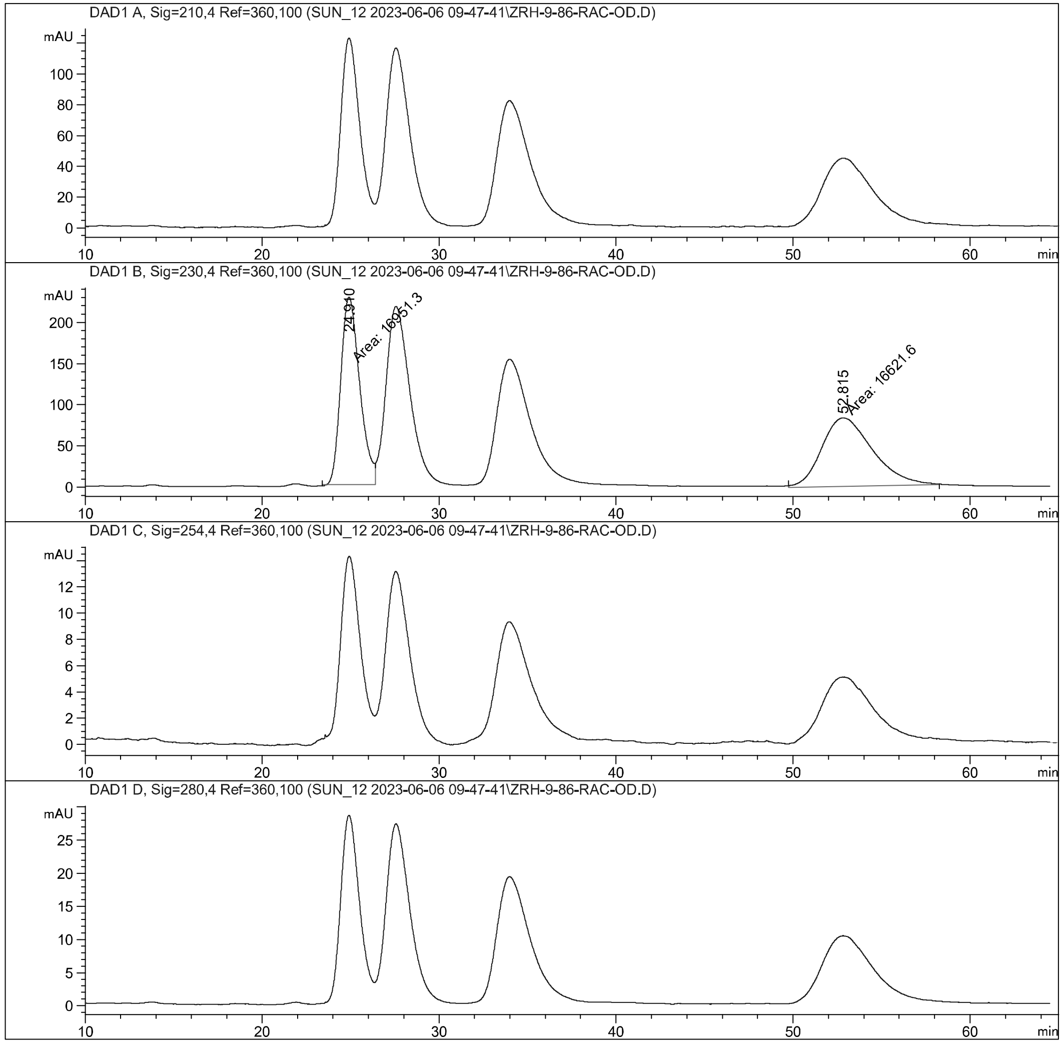

Data File C:\CHEM32\1\DATA\SUN_12 2023-06-06 09-47-41\ZRH-9-86-RAC-OD.D
Sample Name:

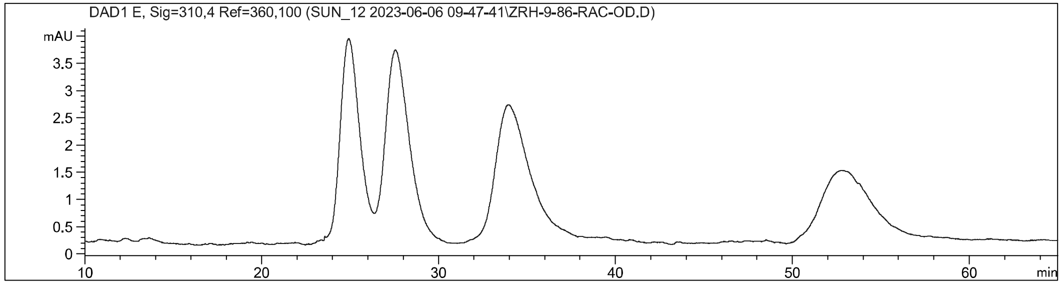

===

Area Percent Report

===

```
Sorted By             :      Signal
Multiplier            :      1.0000
Dilution              :      1.0000
Use Multiplier & Dilution Factor with ISTDs


Signal 1: DAD1 A, Sig=210,4 Ref=360,100


Signal 2: DAD1 B, Sig=230,4 Ref=360,100

Peak RetTime Type  Width     Area      Height     Area
  #   [min]        [min]   [mAU*s]     [mAU]        %
----|-------|----|-------|----------|----------|--------|
   1  24.910 MF    1.2420 1.69512e4  227.47314  50.4910
   2  52.815 MM    3.3361 1.66216e4   83.03809  49.5090

Totals :                  3.35728e4  310.51123


Signal 3: DAD1 C, Sig=254,4 Ref=360,100


Signal 4: DAD1 D, Sig=280,4 Ref=360,100


Signal 5: DAD1 E, Sig=310,4 Ref=360,100
```

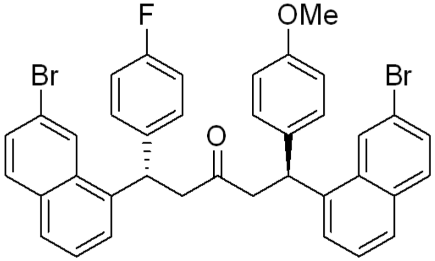

7j
(racemic)

===

*** End of Report ***

Data File C:\CHEM32\1\DATA\SUN_12 2023-06-06 09-47-41\ZRH-9-86-CHIRAL-OD.D
Sample Name:

===

```
Acq. Operator   :                               Seq. Line :  18
Acq. Instrument : Instrument 1                   Location : Vial 31
Injection Date  : 6/6/2023 6:38:36 PM                 Inj :   1
                                               Inj Volume : 5.000 µl
Different Inj Volume from Sequence !     Actual Inj Volume : 20.000 µl
Acq. Method     : C:\CHEM32\1\DATA\SUN_12 2023-06-06 09-47-41\OD-10-60.M
Last changed    : 3/26/2015 10:27:02 AM
Analysis Method : C:\CHEM32\1\DATA\SUN_12 2023-05-12 21-33-41\OD-10-30.M
Last changed    : 6/6/2023 7:48:43 PM
                  (modified after loading)
Additional Info : Peak(s) manually integrated
```

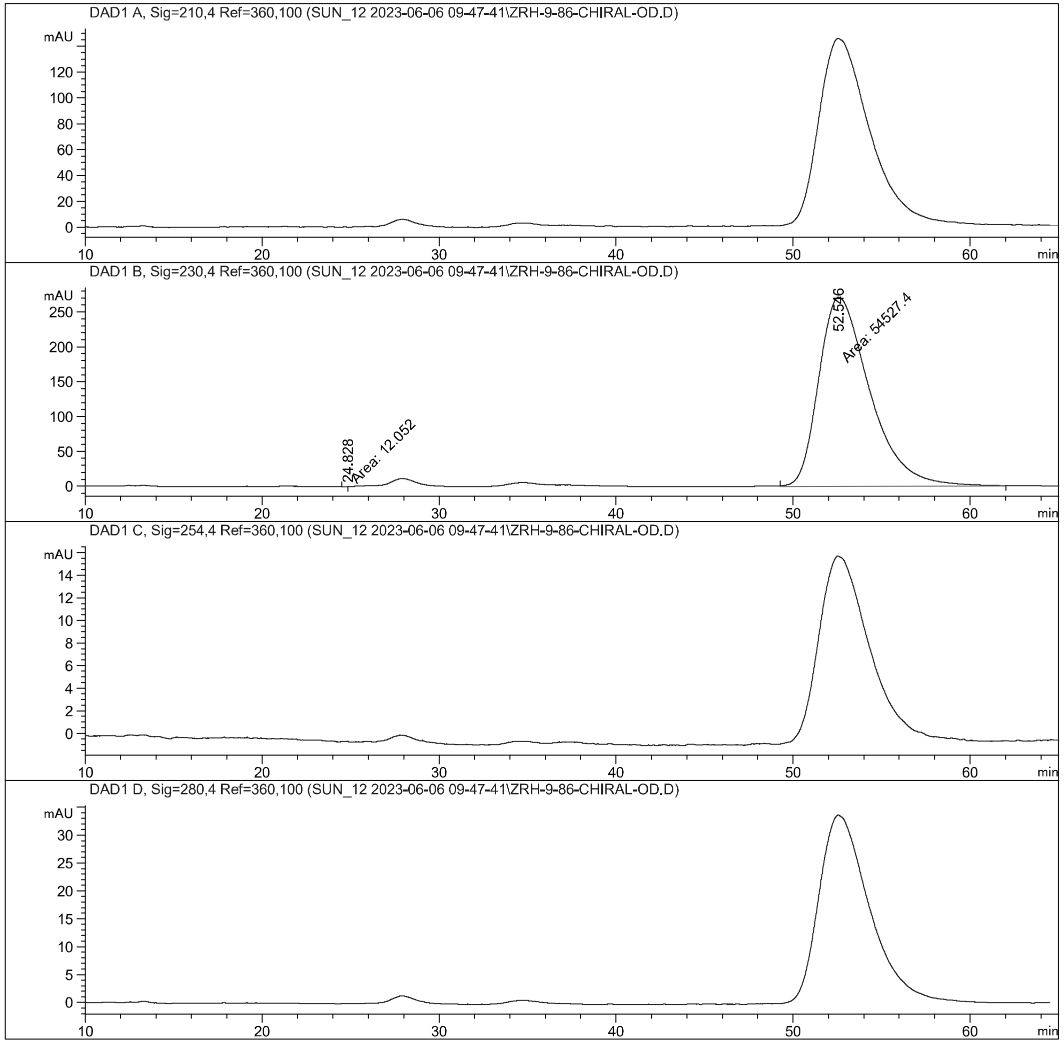

Data File C:\CHEM32\1\DATA\SUN_12 2023-06-06 09-47-41\ZRH-9-86-CHIRAL-OD.D
Sample Name:

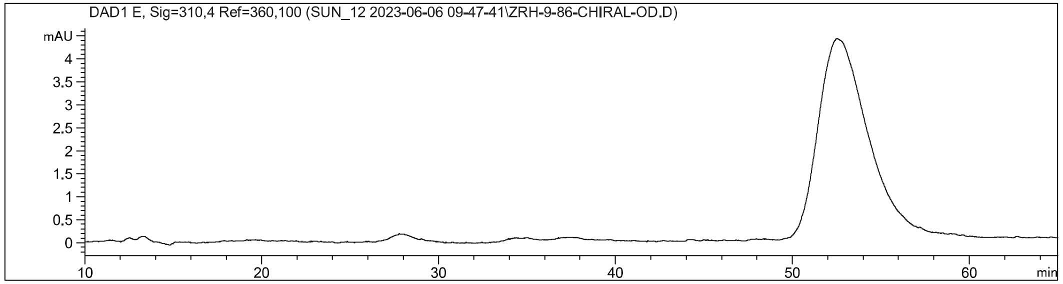

===

Area Percent Report

===

```
Sorted By             :      Signal
Multiplier            :      1.0000
Dilution              :      1.0000
Use Multiplier & Dilution Factor with ISTDs
```

Signal 1: DAD1 A, Sig=210,4 Ref=360,100

Signal 2: DAD1 B, Sig=230,4 Ref=360,100

Peak #	RetTime [min]	Type	Width [min]	Area [mAU*s]	Height [mAU]	Area %
1	24.828	MM	0.2681	12.05200	5.56242e-1	0.0221
2	52.546	MM	3.3470	5.45274e4	271.52014	99.9779

Totals : 5.45395e4 272.07638

Signal 3: DAD1 C, Sig=254,4 Ref=360,100

Signal 4: DAD1 D, Sig=280,4 Ref=360,100

Signal 5: DAD1 E, Sig=310,4 Ref=360,100

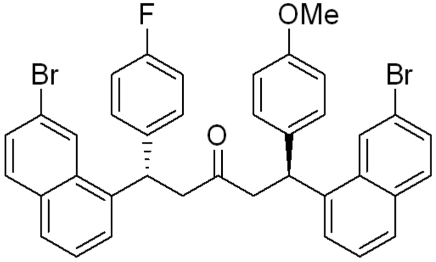

7j
(enantioenriched)

===

*** End of Report ***

===

Acq. Operator : SYSTEM Seq. Line : 2
Sample Operator : SYSTEM
Acq. Instrument : HPLC Location : P2-F-01
Injection Date : 21/6/2023 10:30:21 am Inj : 1
Inj Volume : 2.000 µl
Different Inj Volume from Sample Entry! Actual Inj Volume : 20.000 µl
Acq. Method : C:\Users\Public\Documents\ChemStation\1\Data\SUN\SUN 2023-06-21 10-17-30\OD3-30-80.M
Last changed : 21/6/2023 11:19:06 am by SYSTEM
(modified after loading)
Analysis Method : C:\Users\Public\Documents\ChemStation\1\Data\SUN\SUN 2023-06-21 10-17-30\OD3-30-80.M (Sequence Method)
Last changed : 21/6/2023 12:45:02 pm by SYSTEM
(modified after loading)
Additional Info : Peak(s) manually integrated

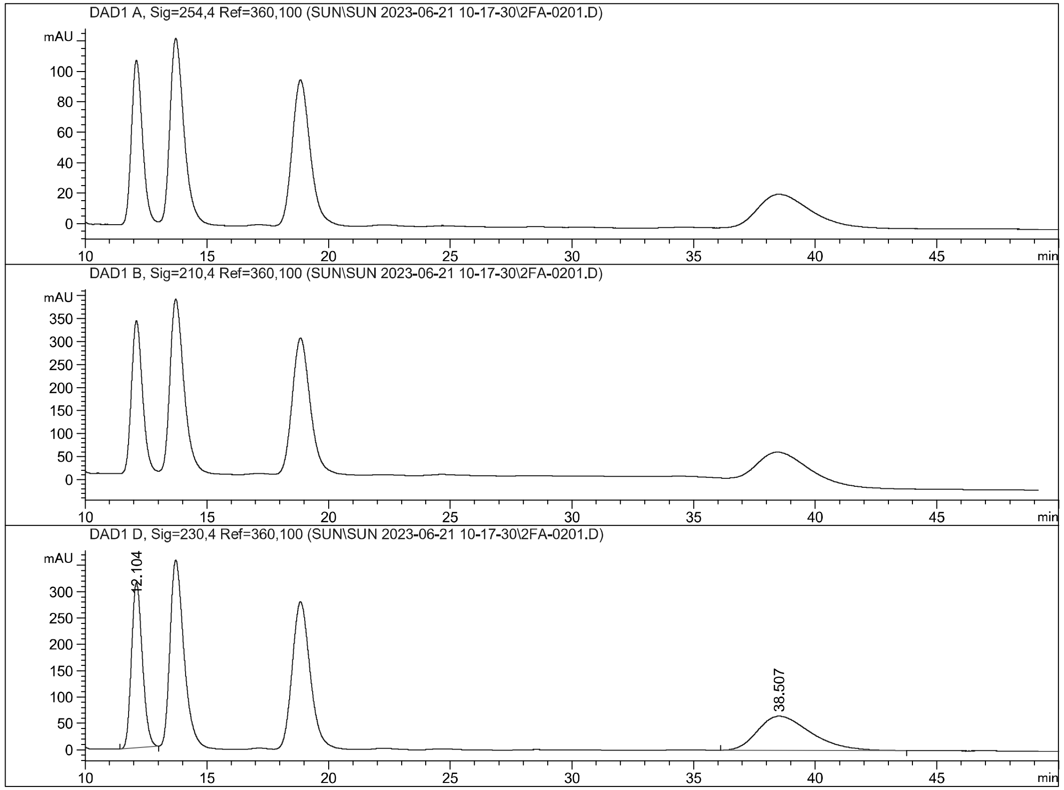

Data File C:\Users\P...Documents\ChemStation\1\Data\SUN\SUN 2023-06-21 10-17-30\2FA-0201.D
Sample Name: zrh-9-133-rac

===

Area Percent Report

===

Sorted By : Signal
Multiplier : 1.0000
Dilution : 1.0000
Use Multiplier & Dilution Factor with ISTDs

Signal 1: DAD1 A, Sig=254,4 Ref=360,100

Signal 2: DAD1 B, Sig=210,4 Ref=360,100

Signal 3: DAD1 D, Sig=230,4 Ref=360,100

Peak #	RetTime [min]	Type	Width [min]	Area [mAU*s]	Height [mAU]	Area %
1	12.104	BB	0.4858	9801.02148	313.47159	49.9829
2	38.507	BB	1.7735	9807.70898	64.73768	50.0171

Totals : 1.96087e4 378.20927

===

*** End of Report ***

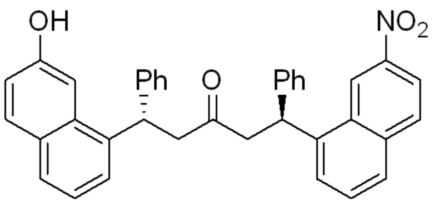

S12
(racemic)

===

Acq. Operator : SYSTEM Seq. Line : 3
Sample Operator : SYSTEM
Acq. Instrument : HPLC Location : P2-F-02
Injection Date : 21/6/2023 11:21:30 am Inj : 1
Inj Volume : 2.000 µl
Different Inj Volume from Sample Entry! Actual Inj Volume : 20.000 µl
Acq. Method : C:\Users\Public\Documents\ChemStation\1\Data\SUN\SUN 2023-06-21 10-17-30
\OD3-30-80.M
Last changed : 21/6/2023 11:19:06 am by SYSTEM
Analysis Method : C:\Users\Public\Documents\ChemStation\1\Data\SUN\SUN 2023-06-21 10-17-30
\OD3-30-80.M (Sequence Method)
Last changed : 21/6/2023 12:45:02 pm by SYSTEM
(modified after loading)
Additional Info : Peak(s) manually integrated

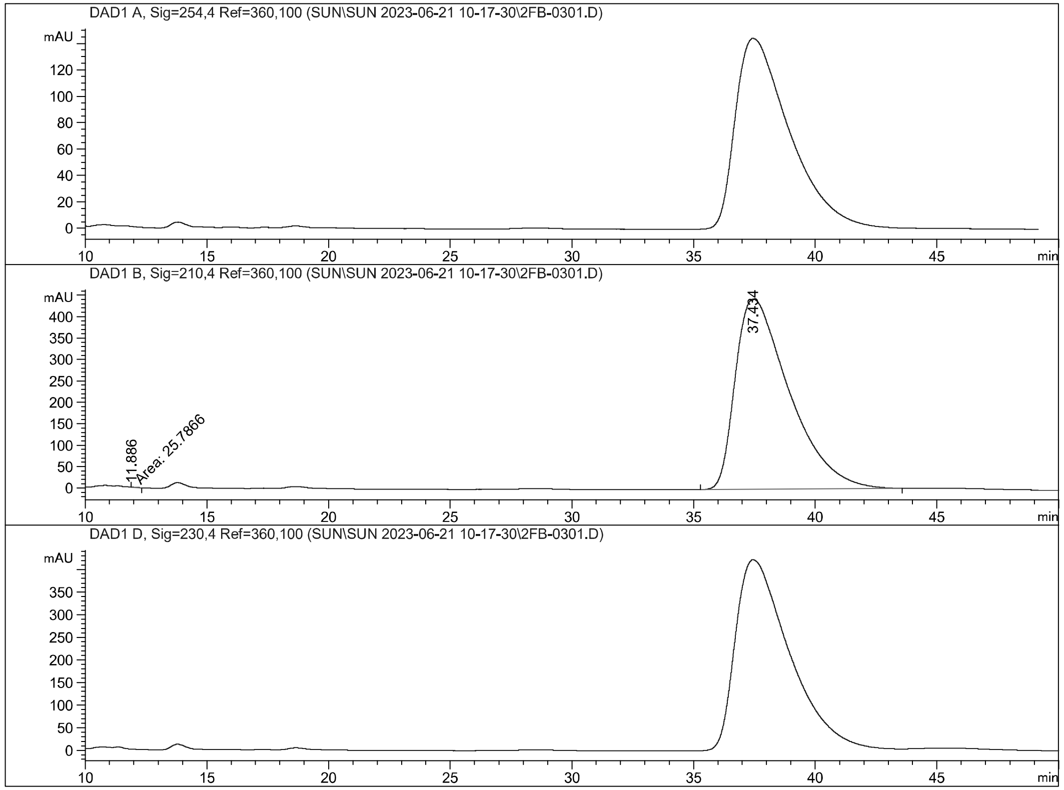

Data File C:\Users\P...Documents\ChemStation\1\Data\SUN\SUN 2023-06-21 10-17-30\2FB-0301.D
Sample Name: zrh-9-133-chiral

===

Area Percent Report

===

Sorted By : Signal
Multiplier : 1.0000
Dilution : 1.0000
Use Multiplier & Dilution Factor with ISTDs

Signal 1: DAD1 A, Sig=254,4 Ref=360,100

Signal 2: DAD1 B, Sig=210,4 Ref=360,100

Peak #	RetTime [min]	Type	Width [min]	Area [mAU*s]	Height [mAU]	Area %
1	11.886	MM	0.2990	25.78662	1.43733	0.0377
2	37.434	BB	1.8033	6.84268e4	444.05551	99.9623

Totals : 6.84526e4 445.49284

Signal 3: DAD1 D, Sig=230,4 Ref=360,100

===

*** End of Report ***

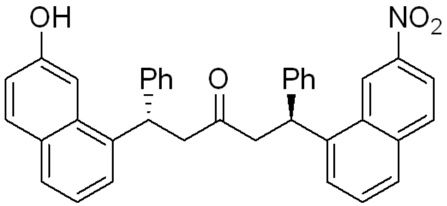

S12
(enantioenriched)

```
=====================================================================
Acq. Operator   :                                Seq. Line :   6
Acq. Instrument : Instrument 1                    Location : Vial 31
Injection Date  : 12/15/2021 10:27:47 AM               Inj :   1
                                                Inj Volume : 5.000 µl
Acq. Method     : C:\CHEM32\1\DATA\SUN_12 2021-12-15 09-26-00\OD-05-30.M
Last changed    : 12/15/2021 10:26:56 AM
                  (modified after loading)
Analysis Method : C:\CHEM32\1\DATA\SUN_12 2022-12-29 09-07-21\2.D\DA.M (OD-05-10.M)
Last changed    : 1/13/2023 9:51:38 AM
                  (modified after loading)
Additional Info : Peak(s) manually integrated
```

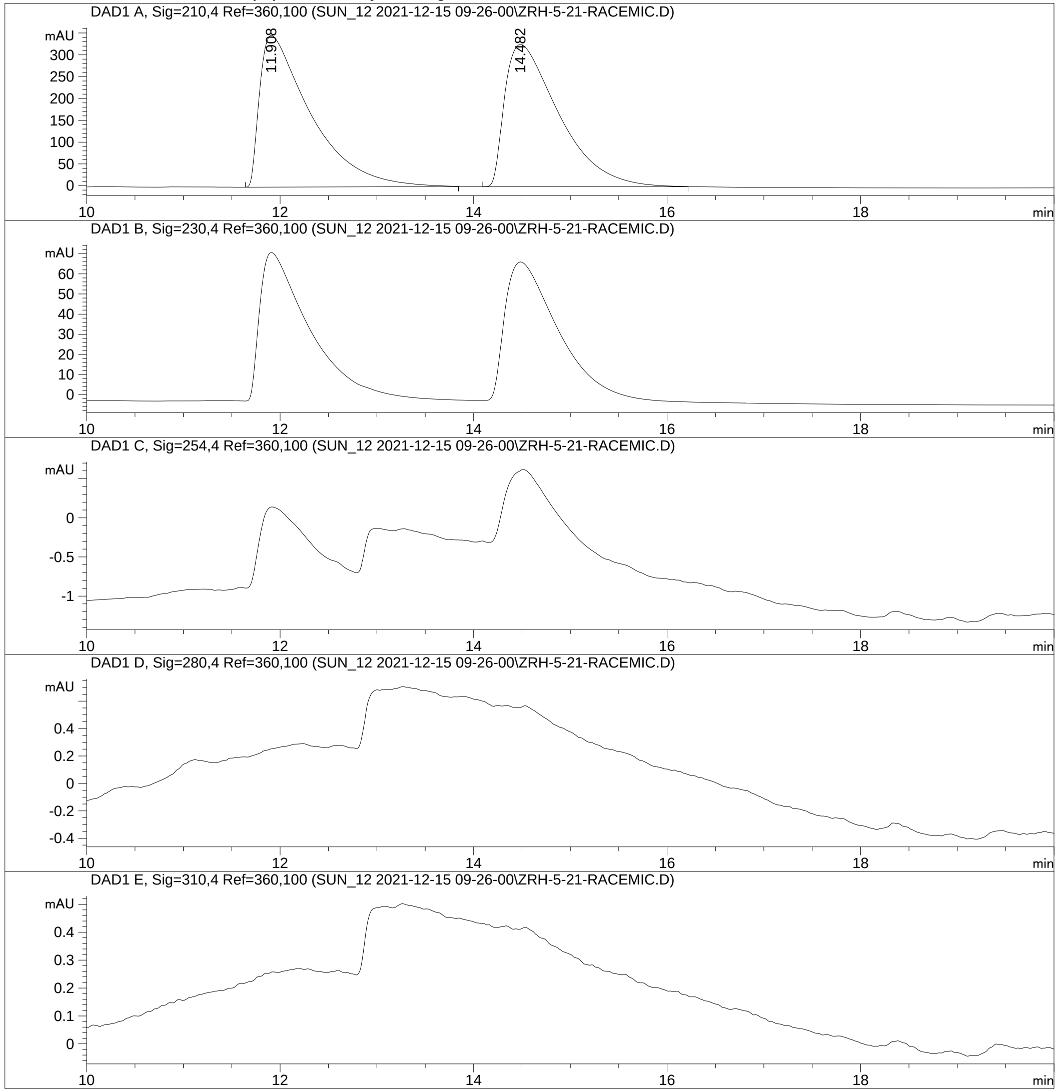

Data File C:\CHEM32\1\DATA\SUN_12 2021-12-15 09-26-00\ZRH-5-21-RACEMIC.D
Sample Name:

```
=====================================================================
                         Area Percent Report
=====================================================================

Sorted By             :      Signal
Multiplier            :      1.0000
Dilution              :      1.0000
Use Multiplier & Dilution Factor with ISTDs


Signal 1: DAD1 A, Sig=210,4 Ref=360,100

Peak RetTime Type  Width     Area      Height     Area
  #   [min]        [min]   [mAU*s]     [mAU]        %
----|-------|----|-------|----------|----------|--------|
   1  11.908 BB    0.5317 1.27400e4  347.48819  50.0596
   2  14.482 BB    0.5951 1.27097e4  325.82605  49.9404

Totals :                  2.54497e4  673.31424



Signal 2: DAD1 B, Sig=230,4 Ref=360,100


Signal 3: DAD1 C, Sig=254,4 Ref=360,100


Signal 4: DAD1 D, Sig=280,4 Ref=360,100


Signal 5: DAD1 E, Sig=310,4 Ref=360,100

=====================================================================
                         *** End of Report ***
```

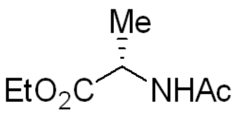

14
(racemic)

===

Acq. Operator : Seq. Line : 2
Acq. Instrument : Instrument 1 Location : Vial 31
Injection Date : 12/21/2021 10:15:17 AM Inj : 1
Inj Volume : 5.000 µl
Acq. Method : C:\CHEM32\1\DATA\SUN_12 2021-12-21 10-06-29\OD-05-25.M
Last changed : 12/21/2021 10:15:02 AM
(modified after loading)
Analysis Method : C:\CHEM32\1\DATA\SUN_12 2022-12-29 09-07-21\2.D\DA.M (OD-05-10.M)
Last changed : 1/13/2023 9:51:38 AM
(modified after loading)
Additional Info : Peak(s) manually integrated

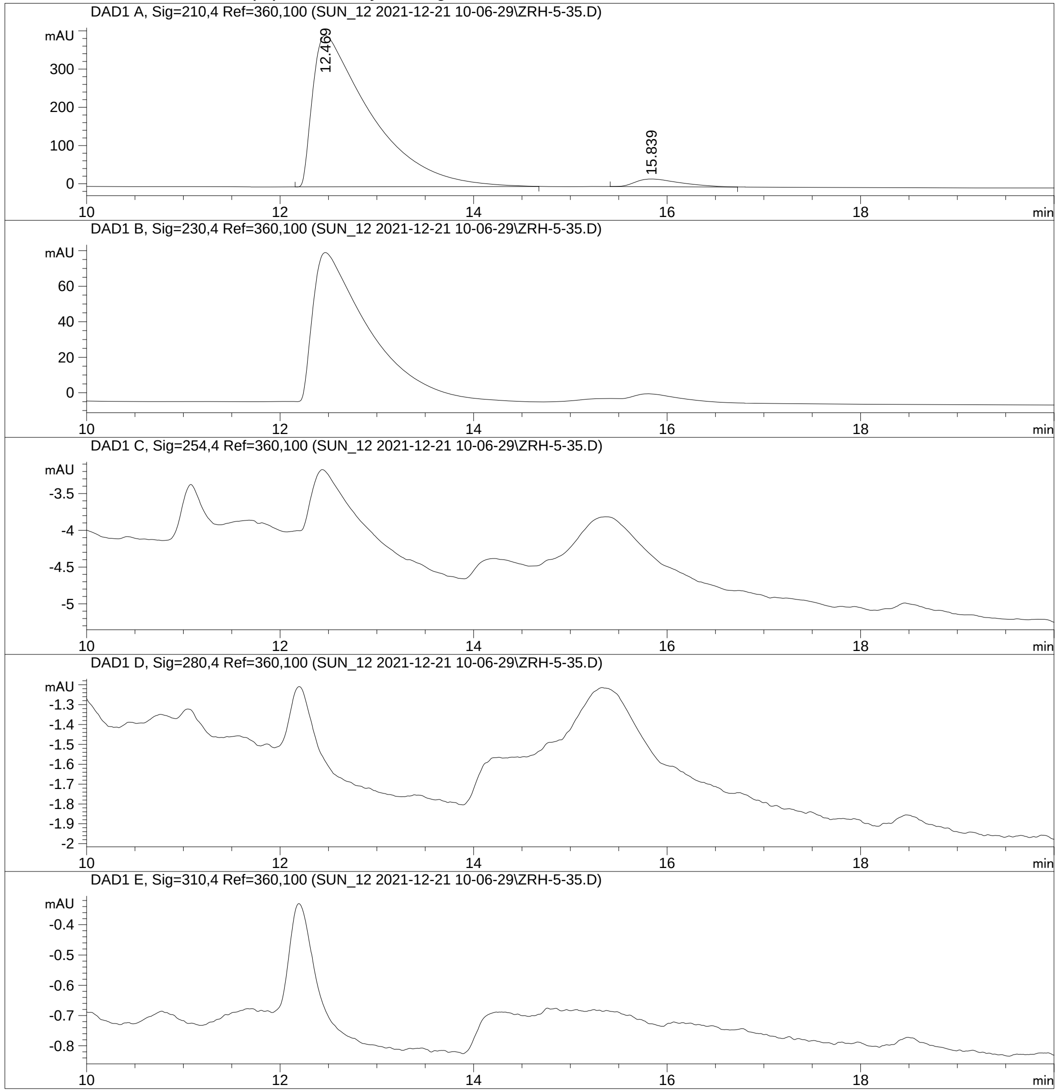

Data File C:\CHEM32\1\DATA\SUN_12 2021-12-21 10-06-29\ZRH-5-35.D
Sample Name:

===
Area Percent Report
===

Sorted By : Signal
Multiplier : 1.0000
Dilution : 1.0000
Use Multiplier & Dilution Factor with ISTDs

Signal 1: DAD1 A, Sig=210,4 Ref=360,100

Peak #	RetTime [min]	Type	Width [min]	Area [mAU*s]	Height [mAU]	Area %
1	12.469	BB	0.5991	1.65288e4	395.87329	96.1887
2	15.839	BB	0.4805	654.92065	20.18723	3.8113

Totals : 1.71837e4 416.06052

Signal 2: DAD1 B, Sig=230,4 Ref=360,100

Signal 3: DAD1 C, Sig=254,4 Ref=360,100

Signal 4: DAD1 D, Sig=280,4 Ref=360,100

Signal 5: DAD1 E, Sig=310,4 Ref=360,100

===
*** End of Report ***

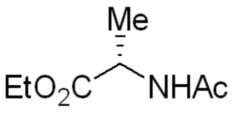

14
(enantioenriched)

===

Acq. Operator : Seq. Line : 2
Acq. Instrument : Instrument 1 Location : Vial 32
Injection Date : 12/31/2022 3:50:55 PM Inj : 1
Inj Volume : 5.000 µl
Different Inj Volume from Sequence ! Actual Inj Volume : 10.000 µl
Acq. Method : C:\CHEM32\1\DATA\SUN_11 2022-12-31 15-42-59\OD-02-30.M
Last changed : 12/31/2022 3:50:02 PM
(modified after loading)
Analysis Method : C:\CHEM32\1\DATA\SUN_12 2022-12-29 09-07-21\2.D\DA.M (OD-05-10.M)
Last changed : 1/13/2023 10:25:47 AM
(modified after loading)
Additional Info : Peak(s) manually integrated

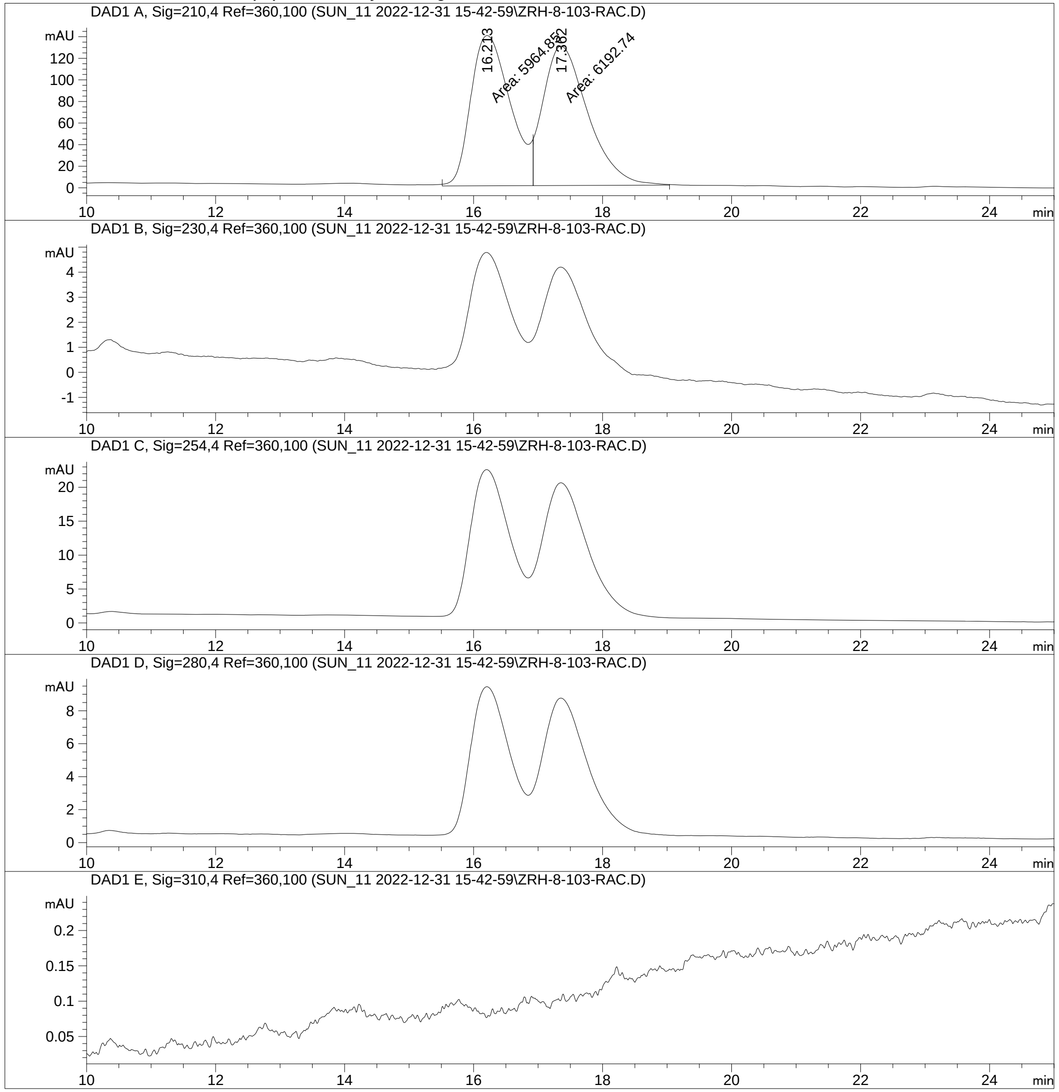

Data File C:\CHEM32\1\DATA\SUN_11 2022-12-31 15-42-59\ZRH-8-103-RAC.D
Sample Name:

===
Area Percent Report
===

Sorted By : Signal
Multiplier : 1.0000
Dilution : 1.0000
Use Multiplier & Dilution Factor with ISTDs

Signal 1: DAD1 A, Sig=210,4 Ref=360,100

Peak #	RetTime [min]	Type	Width [min]	Area [mAU*s]	Height [mAU]	Area %
1	16.213	MF	0.7132	5964.84766	139.39209	49.0627
2	17.362	FM	0.7984	6192.74316	129.27165	50.9373

Totals : 1.21576e4 268.66374

Signal 2: DAD1 B, Sig=230,4 Ref=360,100

Signal 3: DAD1 C, Sig=254,4 Ref=360,100

Signal 4: DAD1 D, Sig=280,4 Ref=360,100

Signal 5: DAD1 E, Sig=310,4 Ref=360,100

===
*** End of Report ***

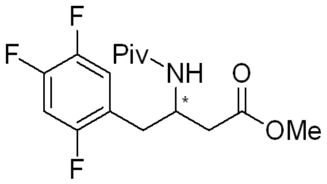

16
(racemic)

===

Acq. Operator : Seq. Line : 26
Acq. Instrument : Instrument 1 Location : Vial 31
Injection Date : 10/6/2022 10:02:12 PM Inj : 1
Inj Volume : 5.000 µl
Different Inj Volume from Sequence ! Actual Inj Volume : 10.000 µl
Acq. Method : C:\CHEM32\1\DATA\SUN_12 2022-10-06 13-48-32\OD-02-30.M
Last changed : 10/6/2022 10:01:20 PM
(modified after loading)
Analysis Method : C:\CHEM32\1\DATA\SUN_12 2022-12-29 09-07-21\2.D\DA.M (OD-05-10.M)
Last changed : 1/13/2023 10:25:47 AM
(modified after loading)
Additional Info : Peak(s) manually integrated

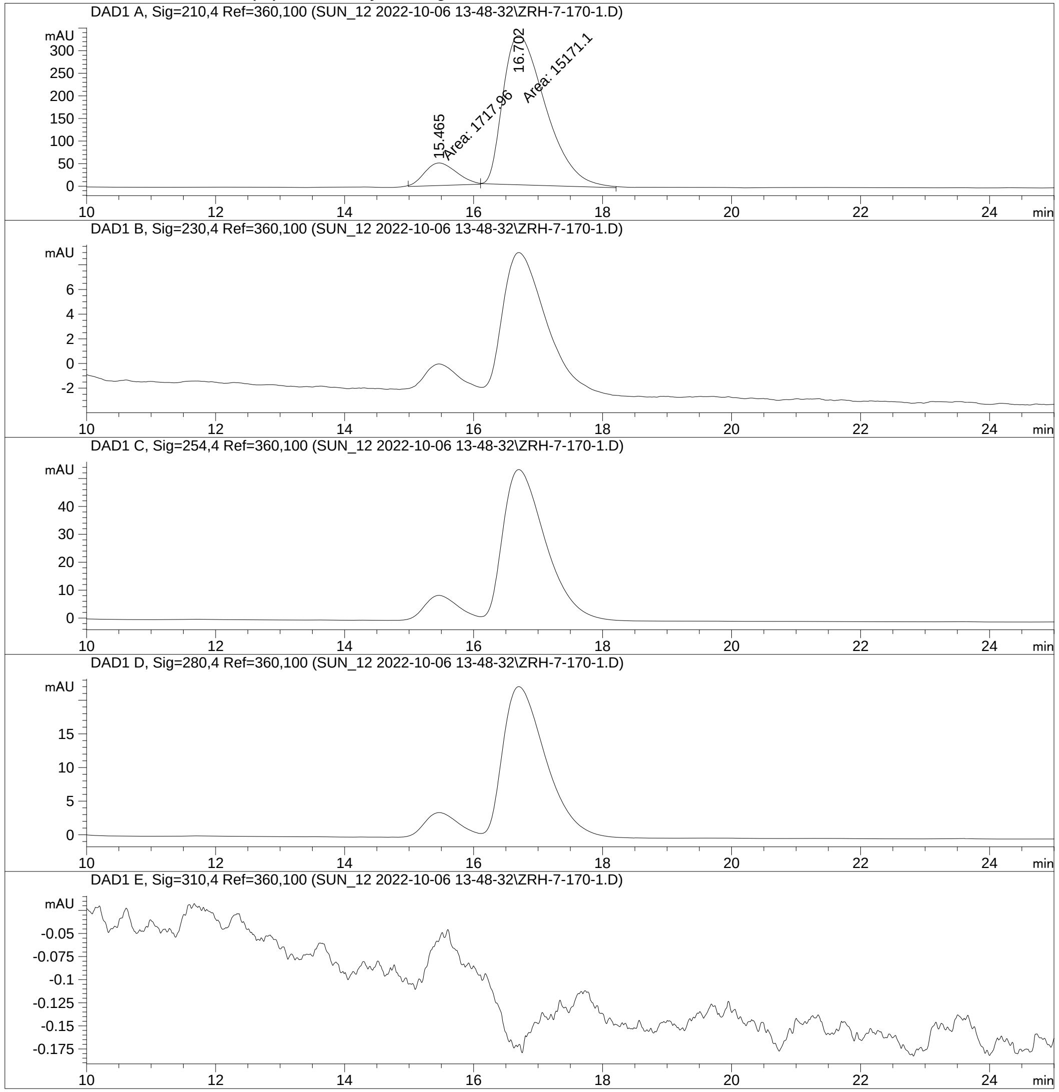

Data File C:\CHEM32\1\DATA\SUN_12 2022-10-06 13-48-32\ZRH-7-170-1.D
Sample Name:

===

Area Percent Report

===

Sorted By : Signal
Multiplier : 1.0000
Dilution : 1.0000
Use Multiplier & Dilution Factor with ISTDs

Signal 1: DAD1 A, Sig=210,4 Ref=360,100

Peak #	RetTime [min]	Type	Width [min]	Area [mAU*s]	Height [mAU]	Area %
1	15.465	MM	0.5733	1717.96265	49.94256	10.1720
2	16.702	MM	0.7649	1.51711e4	330.56140	89.8280

Totals : 1.68891e4 380.50396

Signal 2: DAD1 B, Sig=230,4 Ref=360,100

Signal 3: DAD1 C, Sig=254,4 Ref=360,100

Signal 4: DAD1 D, Sig=280,4 Ref=360,100

Signal 5: DAD1 E, Sig=310,4 Ref=360,100

===

*** End of Report ***

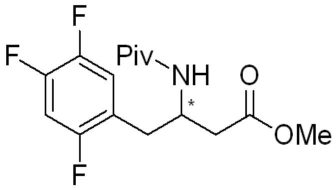

16
(enantioenriched)

===

Acq. Operator : Seq. Line : 4
Acq. Instrument : Instrument 1 Location : Vial 33
Injection Date : 5/28/2023 2:04:55 PM Inj : 1
Inj Volume : 5.000 µl
Different Inj Volume from Sequence ! Actual Inj Volume : 10.000 µl
Acq. Method : C:\CHEM32\1\DATA\SUN_12 2023-05-28 12-51-56\AD-05-30.M
Last changed : 7/17/2016 12:02:24 PM
Analysis Method : C:\CHEM32\1\DATA\SUN_12 2023-05-12 21-33-41\OD-10-30.M
Last changed : 5/28/2023 3:03:36 PM
(modified after loading)
Additional Info : Peak(s) manually integrated

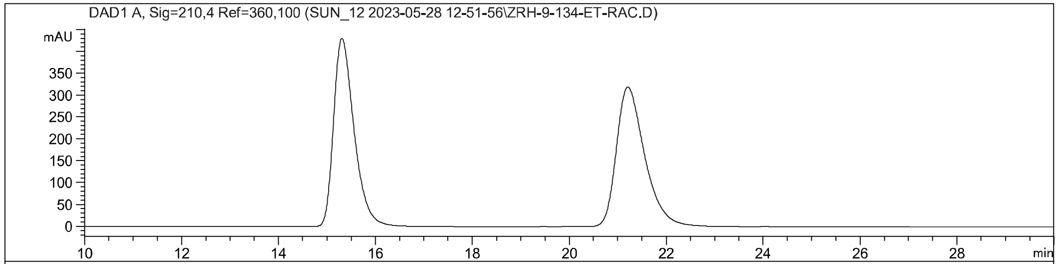

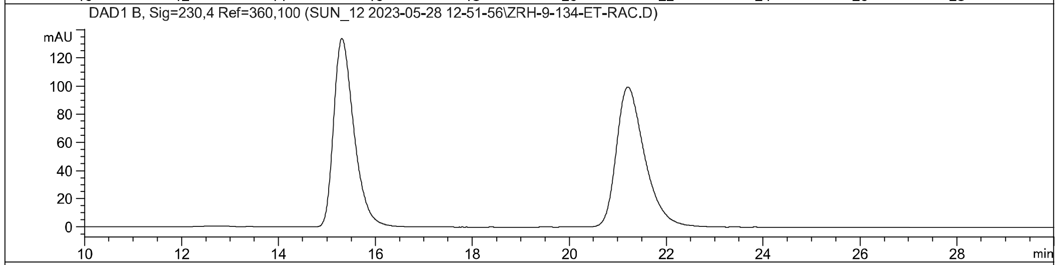

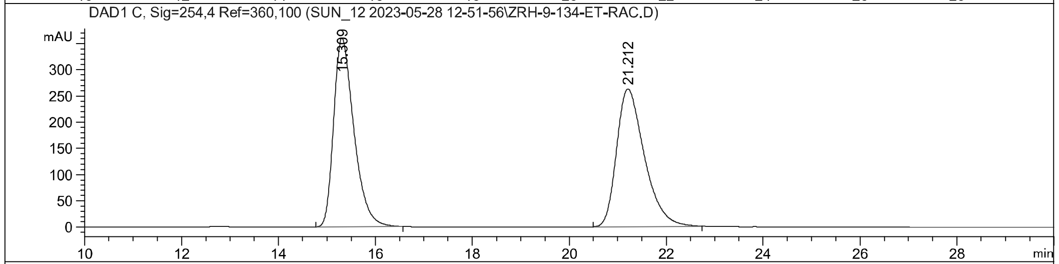

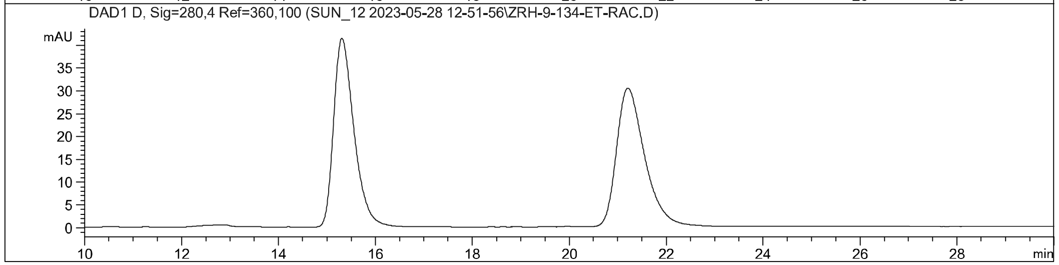

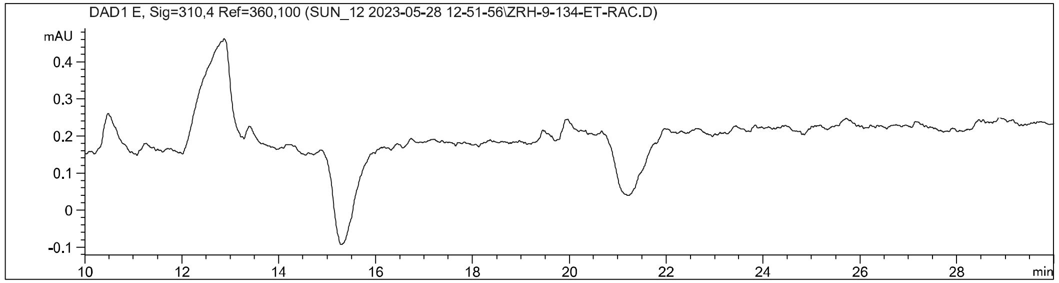

===

Area Percent Report

===

Sorted By : Signal
Multiplier : 1.0000
Dilution : 1.0000
Use Multiplier & Dilution Factor with ISTDs

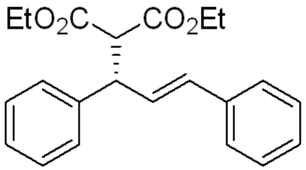

19
(racemic)

Signal 1: DAD1 A, Sig=210,4 Ref=360,100

Signal 2: DAD1 B, Sig=230,4 Ref=360,100

Signal 3: DAD1 C, Sig=254,4 Ref=360,100

Peak #	RetTime [min]	Type	Width [min]	Area [mAU*s]	Height [mAU]	Area %
1	15.309	BB	0.4420	1.04273e4	359.95279	49.9394
2	21.212	BB	0.6062	1.04526e4	262.72134	50.0606

Totals : 2.08799e4 622.67413

Signal 4: DAD1 D, Sig=280,4 Ref=360,100

Signal 5: DAD1 E, Sig=310,4 Ref=360,100

===

*** End of Report ***

===

```
Acq. Operator   :                               Seq. Line :   2
Acq. Instrument : Instrument 1                   Location : Vial 31
Injection Date  : 5/28/2023 1:02:49 PM                Inj :   1
                                               Inj Volume : 5.000 µl
Different Inj Volume from Sequence !     Actual Inj Volume : 10.000 µl
Acq. Method     : C:\CHEM32\1\DATA\SUN_12 2023-05-28 12-51-56\AD-05-30.M
Last changed    : 7/17/2016 12:02:24 PM
Analysis Method : C:\CHEM32\1\DATA\SUN_12 2023-05-12 21-33-41\OD-10-30.M
Last changed    : 5/28/2023 3:03:36 PM
                  (modified after loading)
Additional Info : Peak(s) manually integrated
```

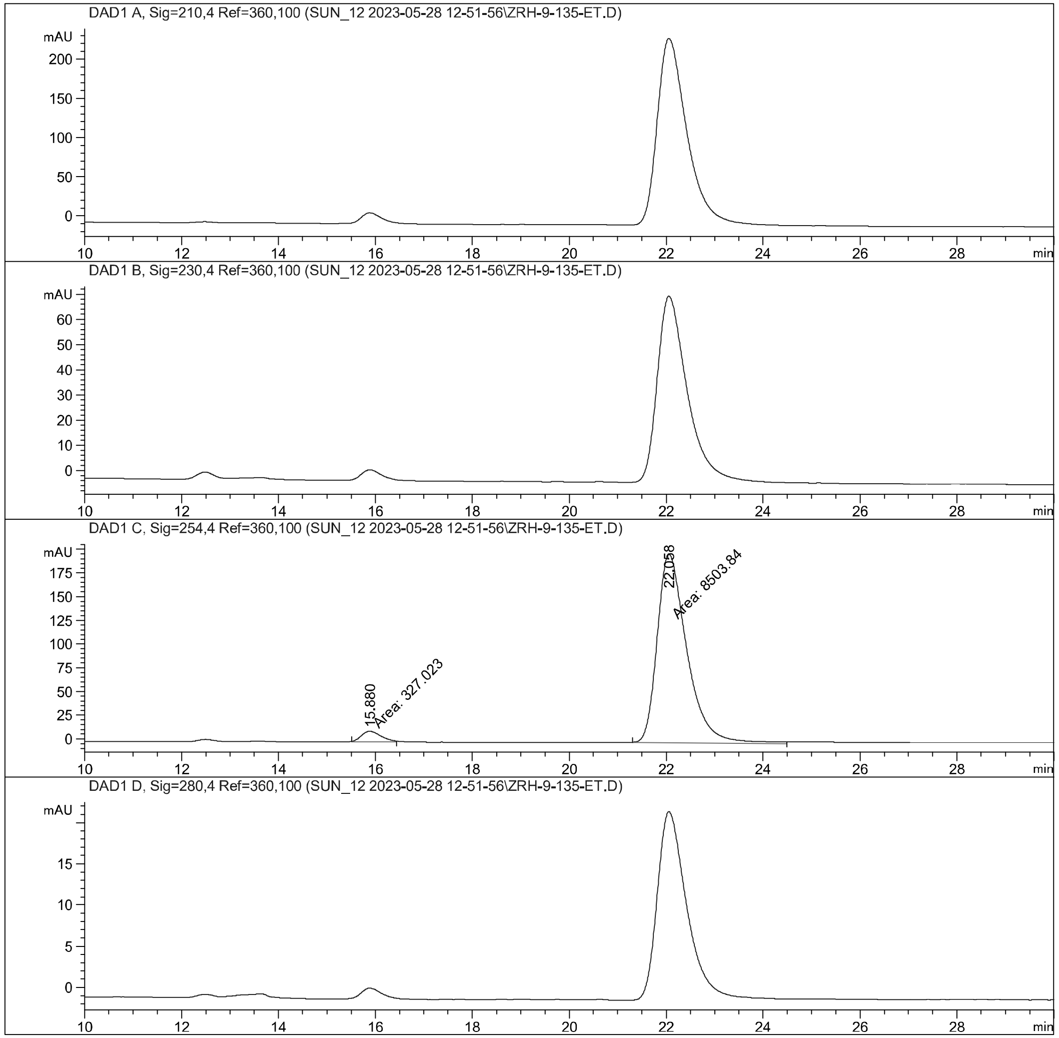

Data File C:\CHEM32\1\DATA\SUN_12 2023-05-28 12-51-56\ZRH-9-135-ET.D
Sample Name:

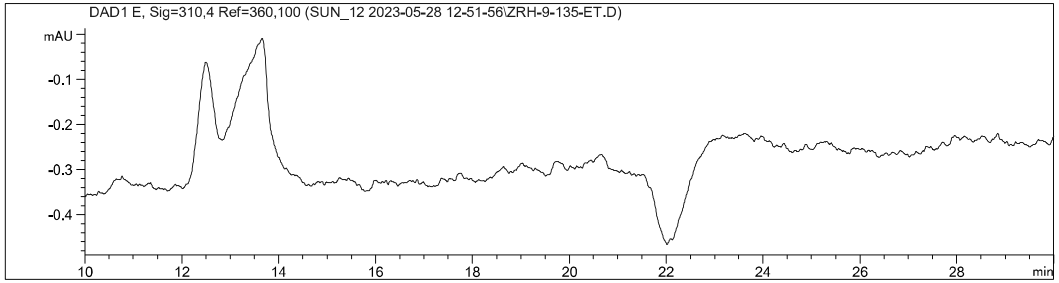

===

Area Percent Report

===

```
Sorted By             :      Signal
Multiplier            :      1.0000
Dilution              :      1.0000
Use Multiplier & Dilution Factor with ISTDs
```

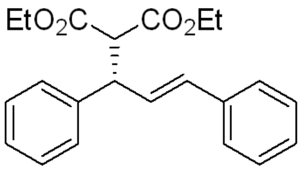

19
(enantioenriched)

Signal 1: DAD1 A, Sig=210,4 Ref=360,100

Signal 2: DAD1 B, Sig=230,4 Ref=360,100

Signal 3: DAD1 C, Sig=254,4 Ref=360,100

Peak #	RetTime [min]	Type	Width [min]	Area [mAU*s]	Height [mAU]	Area %
1	15.880	MM	0.4793	327.02338	11.37136	3.7032
2	22.058	MM	0.7116	8503.84375	199.16219	96.2968

Totals : 8830.86713 210.53354

Signal 4: DAD1 D, Sig=280,4 Ref=360,100

Signal 5: DAD1 E, Sig=310,4 Ref=360,100

===

*** End of Report ***

===

Acq. Operator	:	Seq. Line :	2
Acq. Instrument	: Instrument 1	Location :	Vial 31
Injection Date	: 6/7/2023 7:44:31 PM	Inj :	1
		Inj Volume :	5.000 µl

Different Inj Volume from Sequence ! Actual Inj Volume : 20.000 µl

Acq. Method : C:\CHEM32\1\DATA\SUN_12 2023-06-07 19-36-15\OD-10-30.M
Last changed : 5/8/2023 11:10:02 AM
Analysis Method : C:\CHEM32\1\DATA\SUN_12 2023-05-12 21-33-41\OD-10-30.M
Last changed : 5/8/2023 11:10:02 AM
Additional Info : Peak(s) manually integrated

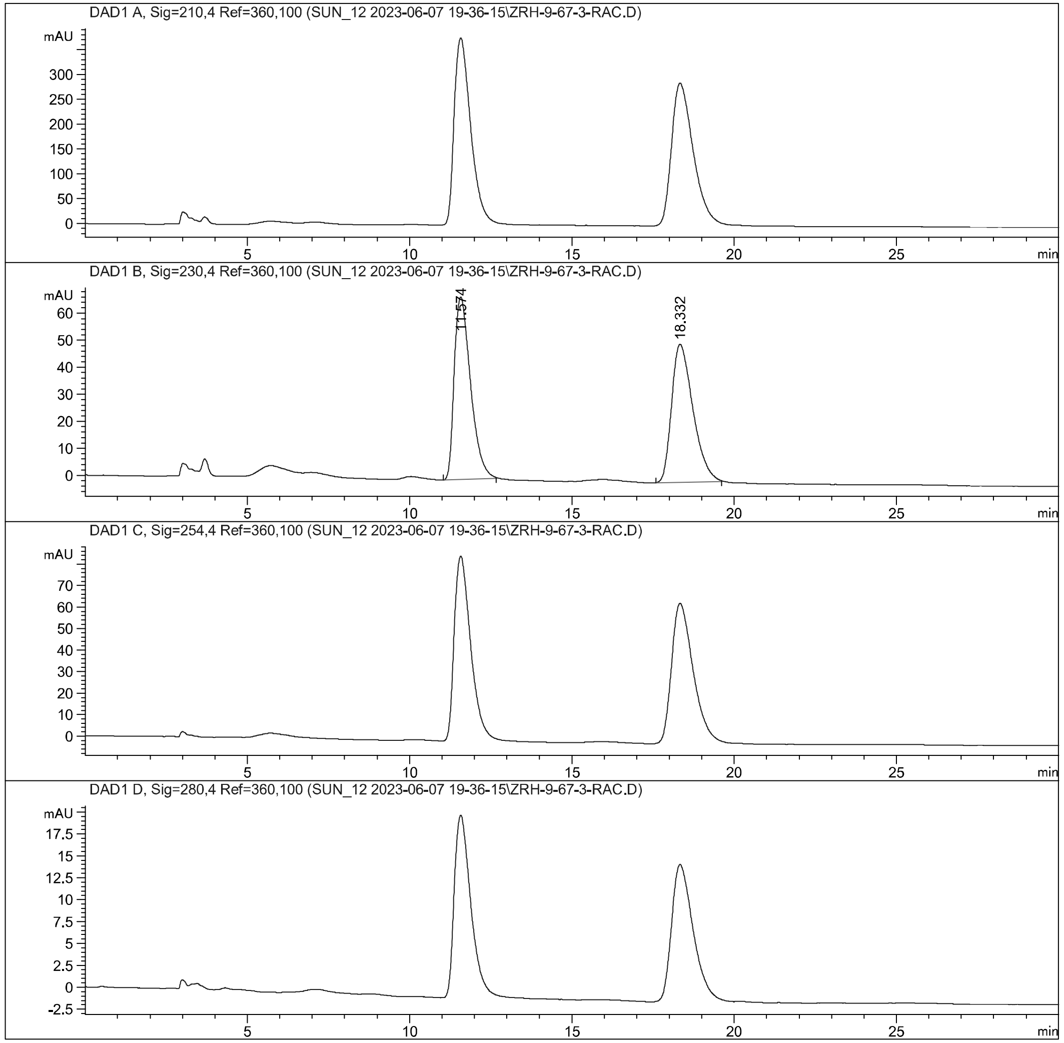

Data File C:\CHEM32\1\DATA\SUN_12 2023-06-07 19-36-15\ZRH-9-67-3-RAC.D
Sample Name:

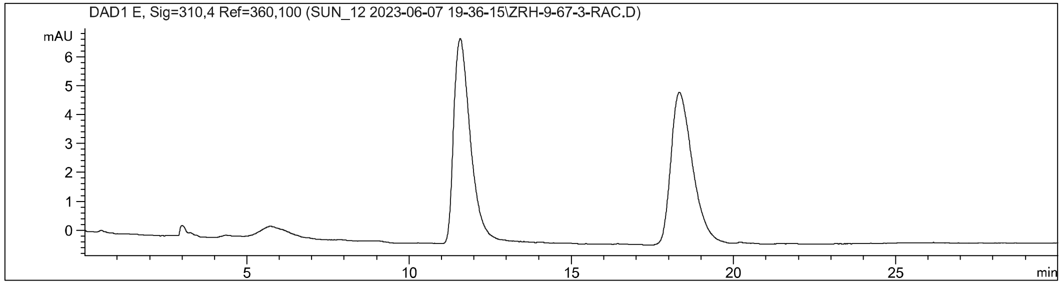

===
Area Percent Report
===

Sorted By : Signal
Multiplier : 1.0000
Dilution : 1.0000
Use Multiplier & Dilution Factor with ISTDs

Signal 1: DAD1 A, Sig=210,4 Ref=360,100

Signal 2: DAD1 B, Sig=230,4 Ref=360,100

Peak #	RetTime [min]	Type	Width [min]	Area [mAU*s]	Height [mAU]	Area %
1	11.574	BB	0.5425	2364.31104	67.53097	50.2165
2	18.332	BB	0.6968	2343.92383	51.13400	49.7835
Totals :				4708.23486	118.66497	

Signal 3: DAD1 C, Sig=254,4 Ref=360,100

Signal 4: DAD1 D, Sig=280,4 Ref=360,100

Signal 5: DAD1 E, Sig=310,4 Ref=360,100

===
*** End of Report ***

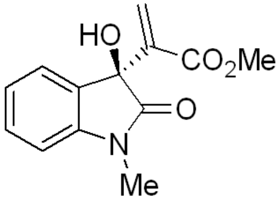

22
(racemic)

===

Acq. Operator	:	Seq. Line :	13
Acq. Instrument	: Instrument 1	Location :	Vial 31
Injection Date	: 5/3/2023 11:46:48 PM	Inj :	1
		Inj Volume :	5.000 µl

Different Inj Volume from Sequence ! Actual Inj Volume : 10.000 µl
Acq. Method : C:\CHEM32\1\DATA\SUN_12 2023-05-03 18-36-45\OD-10-30.M
Last changed : 5/3/2023 10:12:31 PM
(modified after loading)
Analysis Method : C:\CHEM32\1\DATA\SUN_12 2023-05-12 21-33-41\OD-10-30.M
Last changed : 6/6/2023 8:14:28 PM
(modified after loading)
Additional Info : Peak(s) manually integrated

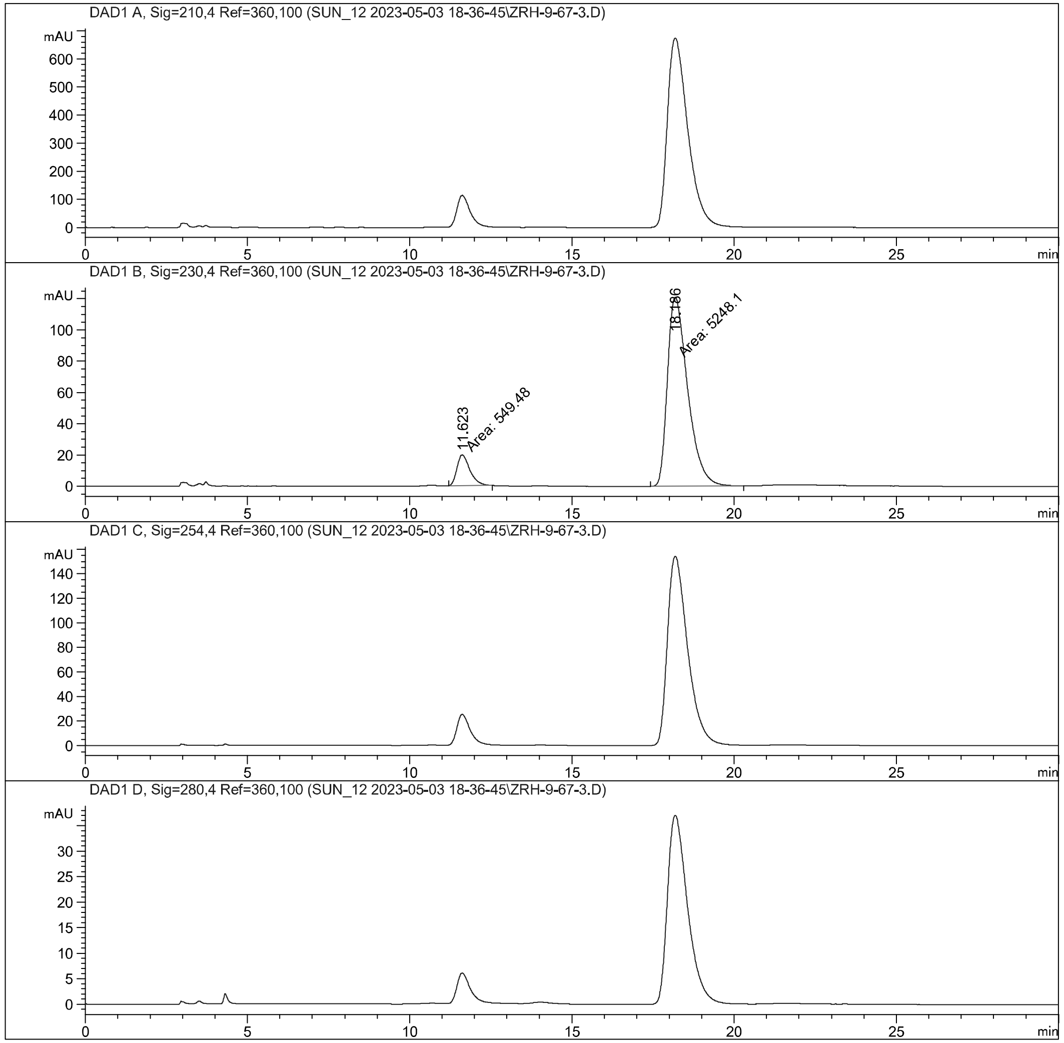

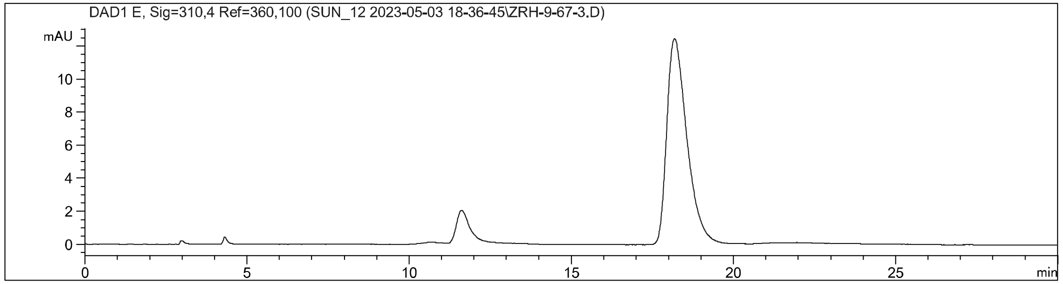

===

Area Percent Report

===

Sorted By : Signal
Multiplier : 1.0000
Dilution : 1.0000
Use Multiplier & Dilution Factor with ISTDs

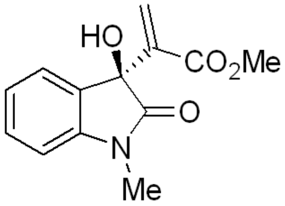

22
(enantioenriched)

Signal 1: DAD1 A, Sig=210,4 Ref=360,100

Signal 2: DAD1 B, Sig=230,4 Ref=360,100

Peak #	RetTime [min]	Type	Width [min]	Area [mAU*s]	Height [mAU]	Area %
1	11.623	MM	0.4649	549.48016	19.70002	9.4778
2	18.186	MM	0.7229	5248.09717	121.00443	90.5222

Totals : 5797.57733 140.70445

Signal 3: DAD1 C, Sig=254,4 Ref=360,100

Signal 4: DAD1 D, Sig=280,4 Ref=360,100

Signal 5: DAD1 E, Sig=310,4 Ref=360,100

===

*** End of Report ***
